# MicroRNAs and Their Inhibition in Modulating *SLC5A8* Expression in the Context of Papillary Thyroid Carcinoma

**DOI:** 10.3390/ijms26167889

**Published:** 2025-08-15

**Authors:** Wojciech Gierlikowski, Jowita Grzędzicka, Katarzyna Konieczek, Marta Kotlarek-Łysakowska

**Affiliations:** 1 Department of Internal Medicine and Endocrinology, Medical University of Warsaw, Banacha 1a, 02-097 Warsaw, Poland; 2 Department of Genomic Medicine, Medical University of Warsaw, Nielubowicza 5, 02-097 Warsaw, Poland; 3 Department of Large Animal Diseases and Clinic, Institute of Veterinary Medicine, Warsaw University of Life Sciences, Nowoursynowska 166, 02-787 Warsaw, Poland; jowita_grzedzicka@sggw.edu.pl; 4 Department of Pediatrics, Medical University of Warsaw, Żwirki i Wigury 63A, 02-091 Warsaw, Poland; katarzyna.konieczek@uckwum.pl; 5 Warsaw Genomics, Żwirki i Wigury 101, 02-089 Warsaw, Poland; marta.kotlarek-lysakowska@warsawgenomics.pl

**Keywords:** SLC5A8, AIT, microRNA, papillary thyroid carcinoma, thyroid carcinoma, PTC

## Abstract

SLC5A8 is a protein coded by the *SLC5A8* gene, and has been proposed as a tumor suppressor and iodide transporter. Its expression is reduced in papillary thyroid carcinoma (PTC), yet the mechanisms underlying this phenomenon are largely unknown. We hypothesized that *SLC5A8* expression in PTC is reduced by microRNAs and can be modulated by their inhibition. We used real-time PCR to analyze the expression of *SLC5A8* and the microRNAs of interest in a set of 49 PTC/normal tissue pairs. We used an in silico approach to identify microRNAs upregulated in PTC and putatively binding to the *SLC5A8* transcript. Luciferase assays were performed to confirm the direct binding of synthetic microRNAs to the 3′UTR of *SLC5A8*. Subsequently, using mir-expressing plasmids and microRNA sponges, including a microRNA sponge designed to simultaneously inhibit three selected microRNAs, we checked the impact of the modulation of microRNAs on endogenous *SLC5A8*. Finally, we investigated if modulation of SLC5A8 induces changes in transcriptomes. We confirmed the downregulation of *SLC5A8* in PTC. In silico analysis revealed microRNAs potentially targeting *SLC5A8*. Luciferase assay confirmed direct binding between the 3′UTR of *SLC5A8* and miR-181a-5p, miR-182-5p, and miR-494-3p. MiR-181a-5p and miR-182-5p were upregulated in PTC. In HEK293 cell lines, transfection with mir-181a- and mir-182-expressing plasmids decreased endogenous *SLC5A8* mRNA, while silencing of miR-181a-5p, miR-182-5p, miR-494-3p, and all three microRNAs simultaneously increased *SLC5A8* expression; however, only simultaneous inhibition was able to induce changes visible for SLC5A8 protein. Changes in *SLC5A8* expression did not alter the whole transcriptome significantly. This study shows microRNA-dependent regulation of *SLC5A8* expression and underlines the potential effectiveness of simultaneous inhibition of a few microRNAs to derepress their common target.

## 1. Introduction

The *SLC5A8* gene encodes for a multi-pass membrane protein, initially described as apical iodide transporter (AIT, SLC5A8) or sodium-coupled monocarboxylate transporter 1 (SMCT1), responsible for the transport of monocarboxylates and nicotinate [1]. It is expressed in virtually all human tissues, but its expression in the thyroid gland is 10-fold higher than elsewhere [2].

*SLC5A8* was postulated as a potent tumor suppressor [3] and its lowered levels were shown in numerous malignancies [4,5,6,7,8,9], including follicular thyroid carcinoma [10] and papillary thyroid carcinoma [11], where its suppression seems to be BRAF-dependent [12] and is related to tumor aggressiveness [13]. It was also shown that its expression is reduced in a mice model of ALK-driven thyroid carcinoma [14]. In colorectal cancer, SLC5A8 expression positively correlates with disease-free survival [15]. Additional confirmation of the tumor-suppressive function of SLC5A8 was indicated by the fact that re-expression of SLC5A8 leads to the suppression of colony formation in colon cancer cell lines [3]. This role of SLC5A8 is possibly mediated by its ability to transport histone deacetylases inhibitors [16] and by its role in depleting the antiapoptotic Survivin, encoded by the *BIRC5* gene [17].

In the thyroid gland, SLC5A8 initially was proposed as a transporter that mediates iodide efflux from thyrocytes into the follicle lumen and was named the apical iodide transporter (AIT) [2]. However, further studies reported conflicting results. A number of electrophysiological studies did not show evidence for iodide transport by SLC5A8 [15,18,19], but later studies performed by Coady et al. [20], as well as molecular modeling studies [21], supported this function. SLC5A8 was also shown to transport putative anti-cancer drugs 3-bromopyruvate [22] and dichloroacetate [23].

Regulation of *SLC5A8* expression has not been well established. The gene’s promoter sequence [24] and a targeting transcription factor were identified [25], but thorough studies characterizing the regulatory mechanisms are lacking. In the thyroid gland, SLC5A8 levels are independent of the TSH concentration in plasma [11]. The presence of the BRAF^V600E^ mutation, occurring in approximately 30% of PTCs, causes hypermethylation of exon 1 and leads to reduced expression of *SLC5A8* [12]. However, the lack of a significant correlation between the gene methylation and mRNA levels [5] suggests the role of post-transcriptional regulation, such as that mediated by microRNAs.

MicroRNAs (miRNAs, miRs) are short (19–25 nucleotides) non-coding RNA molecules that typically function as negative regulators of the expression of protein-coding genes. It is speculated that microRNAs altogether regulate around 60% of the human genome, which highlights their potential importance as global regulators of gene expression. Mature microRNAs target and inhibit translation or promote mRNA degradation by annealing to complementary sequences in mRNA 3′ untranslated regions (3′UTRs) [26]. Many cancers, including papillary thyroid carcinoma, exhibit aberrant expression of microRNAs [27], which leads to the deregulation of the expression of numerous protein-coding genes.

Changes in microRNA expression patterns are typical for different pathological conditions and can be used to diagnose thyroid lesions (as revised in [28]). Aberrant expression of microRNAs leads to altered expression of their target genes, contributing to the promotion and progression of cancer. Since the expression of microRNAs can be modulated, this phenomenon can be further used for tailoring adjuvant cancer therapies [29]. Lowered levels of SLC5A8 potentially lead to the promotion of carcinogenesis and hypothetically may affect the efficiency of radioactive iodine treatment of cancer patients.

Since the mechanisms regulating *SLC5A8* expression have not been thoroughly studied, we hypothesized that the lower expression of *SLC5A8* in papillary thyroid carcinoma results from overexpression of its regulatory microRNAs. Thus, the aim of this study was to identify microRNAs that bind to the *SLC5A8* transcript, regulate its expression, and contribute to its aberrant levels in PTCs, as we previously suggested [30], and to verify if microRNA silencing using microRNA sponges, including constructs targeting a few microRNAs simultaneously, would result in a restoration of *SLC5A8* expression and changes in the whole transcriptome.

## 2. Results

### 2.1. The Expression of SLC5A8 Is Lowered in PTC

**Figure 1:**
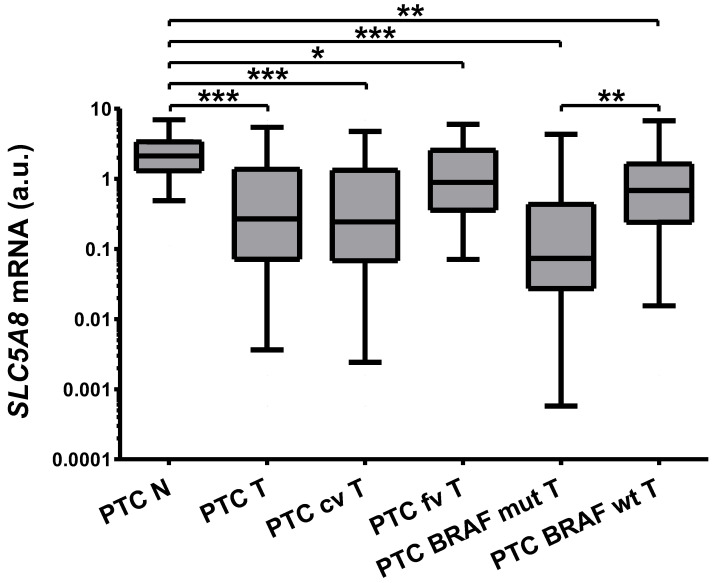
The expression of *SLC5A8* is 7.87-fold (*p* < 0.001) reduced in PTC (PTC T, n = 49) compared with normal adjacent thyroid tissue obtained simultaneously from the contralateral lobe of the same patient (PTC N, n = 49). The reduction was more profound in BRAF-mutated (PTC BRAF mut T, n = 19) than in the wild-type BRAF group (PTC BRAF wt T, n = 23, 9-fold, *p* = 0.0047) and in the classic variant of PTC (PTC cv T, n = 43) compared with the follicular variant (PTC fv T, n = 6). Expression in all healthy tissue is plotted as it includes the other control subset, which statistically do not differ. The graph shows the expression of *SLC5A8* in thyroid tissue samples normalized against *HPRT*. Data are expressed as median, interquartile range, and a 5–95 percentile. A logarithmic scale was used. Statistical analysis was performed with a Wilcoxon matched-pairs signed-ranks test and Mann–Whitney U test (the latter to compare SLC5A8 expression in tumor tissue depending on BRAF status. * *p* < 0.05, ** *p* < 0.01, *** *p* < 0.001).

In 43/49 (88%) tissue pairs, the expression of *SLC5A8* was lower in tumor than control tissue. The median decrease was 7.87-fold (*p* < 0.001). Importantly, we observed a 9-fold difference in the median expression between BRAF^V600E^ wild-type (T, n = 23) and mutated (T/A, n = 19) group (3.18 vs. 29 median decrease, *p* = 0.0047), and the decrease was also more profound in PTC classic variant (PTC cv) than in PTC follicular variant (PTC fv, Figure 1). There was no correlation between the expression of *SLC5A8* and tumor aggressiveness, defined as local or vascular infiltration or lymph node metastases.

### 2.2. In Silico Analysis Reveals Candidate MicroRNAs Regulating the Expression of SLC5A8

**Figure 2:**
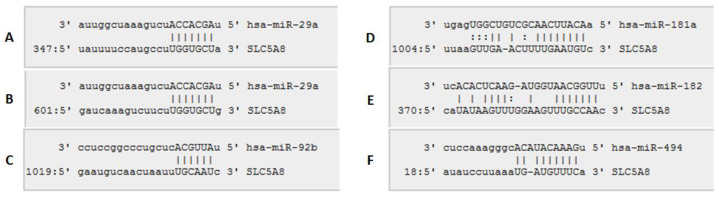
Putative binding sites of selected microRNAs: miR-29a-3p (**A**,**B**), miR-92b-3p (**C**), miR-181a-5p (**D**), miR-182-5p (**E**), and miR-494-3p (**F**) in *SLC5A8* 3′UTR according to in silico analysis. Numbers in the bottom-left corner of each panel represent the position within the 3′UTR sequence (microRNA.org).

Among microRNAs with putative binding sites in the 3′UTR of *SLC5A8*, miR-29a-3p, miR-92b-3p, miR-181a-5p, miR-182-5p, and miR-494-3p showed the highest upregulation and/or expression level in tumor tissue [27], and were chosen for further analyses (Figure 2).

### 2.3. Luciferase Assay Confirms Binding of miR-181a-5p, miR-182-5p, and miR-494-3p to the 3′UTR of SLC5A8

**Figure 3:**
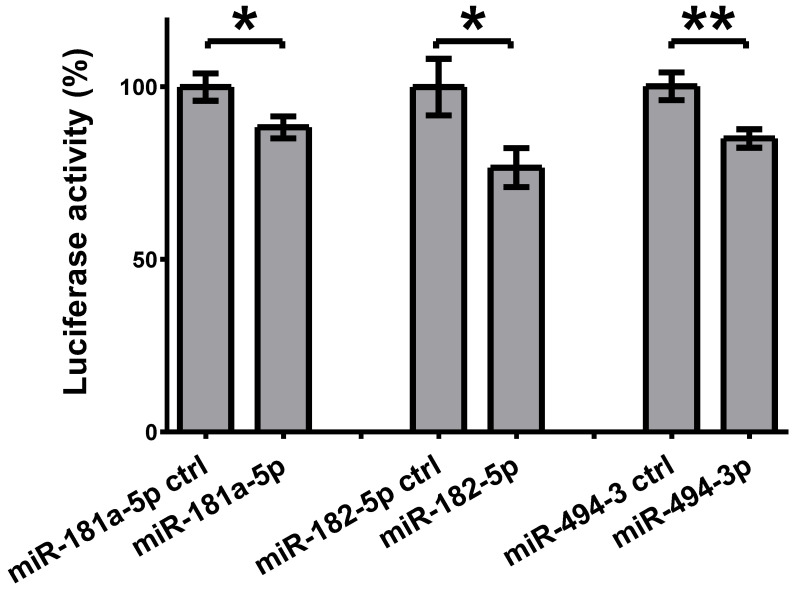
Binding of microRNAs with 3′UTR of *SLC5A8* using luciferase reporter assay. Luciferase activity is reduced upon transfection with miR-181a-5p (by 12%, *p* = 0.04), miR-182-5p (by 23%, *p* = 0.03), and miR-494-3p (by 15%, *p* = 0.007). Luciferase activity is shown as a percentage relative to the control (cells transfected with a scrambled control microRNA). The graph shows the mean along with deviations from mean (SEM). Statistical analysis was performed using an unpaired *t* test (* *p* < 0.05, ** *p* < 0.01).

To analyze the direct binding of the selected microRNAs to the 3′UTR of *SLC5A8* transcript, the HeLa cell line was co-transfected with a pEZX-*SLC5A8* reporter vector and synthetic microRNAs. Transfection of cells with synthetic miR-181a-5p, miR-182-5p, and miR-494-3p resulted in a 12% (*p* = 0.04), 23% (*p* = 0.03), and 15% (*p* = 0.007) decrease in luminescence, respectively (Figure 3). 

### 2.4. The Expression of Identified MicroRNAs Is Deregulated in Tumor Tissue

**Figure 4:**
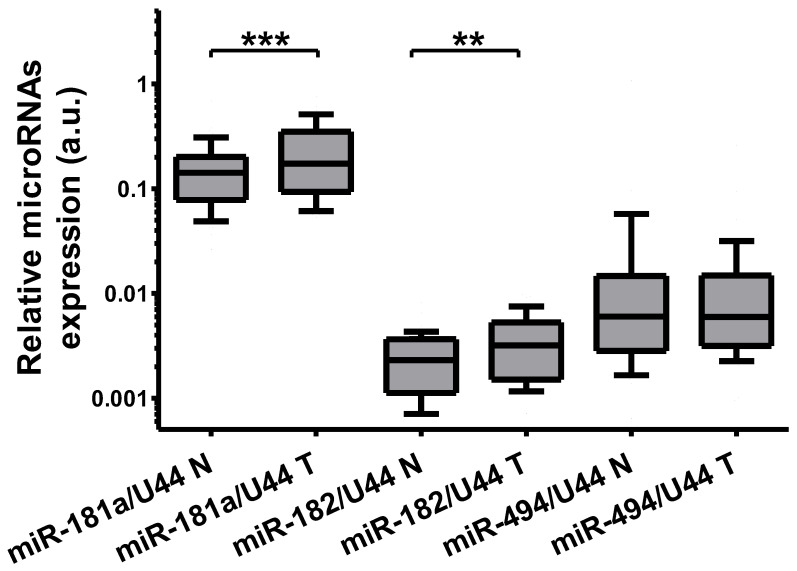
The ratio of expression of the analyzed microRNAs in a tumor (PTC-T) compared to normal adjacent thyroid tissue obtained simultaneously from the contralateral lobe of the same patient (PTC-N, N = 49). The mean expression difference was 1.22 for miR-181a-5p (*p* = 0.0007) and 1.38-fold (*p* = 0.002) for miR-182-5p. Data are expressed as median, interquartile range, and 10–90 percentile. Statistical analysis was performed with a Wilcoxon matched-pairs signed-ranks test to compare expression of each microRNA in PTC-T vs. PTC-N tissue (** *p* < 0.01, *** *p* < 0.001). A logarithmic scale was used.

The expression of microRNAs binding to the 3′UTR of SLC5A8 was further analyzed in thyroid tissue pairs (N = 49) using real-time PCR. The expression of miR-181a-5p was increased 1.22-fold (*p* = 0.0007) and that of miR-182-5p was increased 1.38-fold (*p* = 0.002). The expression of miR-494-3p was not increased (Figure 4).

### 2.5. Plasmid Functionality Verification

**Figure 5:**
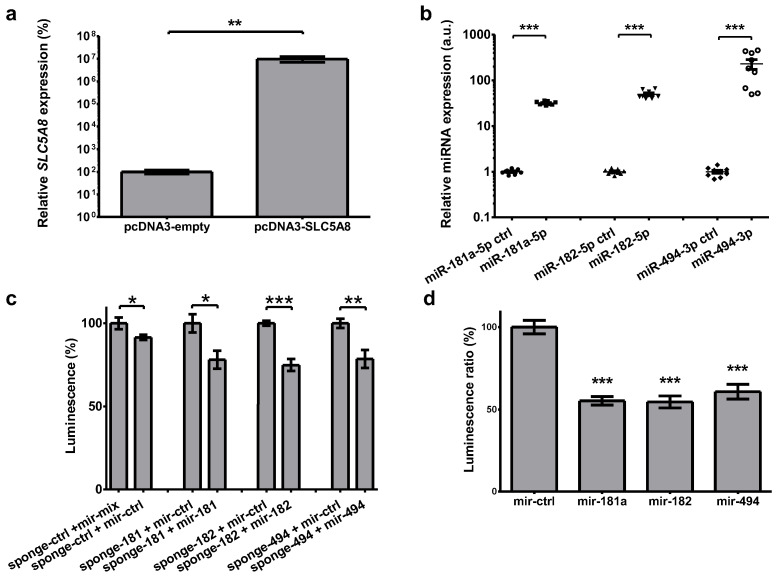
Verification of plasmids functionality. (**a**) Transfection of HEK293 cells with SCL5A8-expressing plasmid led to 10^5^-fold increase in its expression (*p* = 0.004). The results were normalized against *HPRT* expression. (**b**) Expression of microRNAs upon the transfection with a microRNA-expressing plasmids. Transfection of HeLa cells with mir-181a, -182, and -494 expressing plasmids led to a 32-, 50-, and 229-fold increase in their expression, respectively (all *p* < 0.001). The results were normalized to U44 expression. (**c**) As sponge is cloned downstream of the luciferase gene, the activity of luciferase expressed from the sponge plasmids is reduced upon transfection with a corresponding microRNA. Luminescence mediated by the sponge ctrl is reduced upon co-transfection with the mir–ctrl-expressing plasmid in comparison to co-transfection with a mix of mir-181a-, mir-182-, and mir-494-expressing plasmids by 8% (*p* = 0.045). Similarly, co-transfection with sponge-181 and mir-181-expressing plasmids led to reduction in luciferase activity by 22% (*p* = 0.012), whilst for sponge-182 and mir-182 it was by 25% (*p* < 0.0001), and for sponge-494 and mir-494 it was by 21% (*p* = 0.003). (**d**) Sponge–mix plasmid was designed to bind with each of miR-181a-5p, miR-182, and miR-494-3p. Co-transfection of HeLa cells with the sponge–mix plasmid with microRNA-expressing plasmids led to decrease in luciferase activity by 45% in the case of mir-181a, 45% for mir-182, and 31% for mir-494 (all *p* < 0.001). The results were normalized to *Renilla* luciferase. The graphs show the mean along with deviations from mean (SEM). Statistical analysis was performed using an unpaired *t* test (* *p* < 0.05, ** *p* < 0.01, *** *p* < 0.001). A logarithmic scale was used in (**a**,**b**).

Functionality of the plasmids used was verified in vivo in a way similar to that described previously [31]. Briefly, transfection of HEK293 cells with SLC5A8-expressing plasmid increased its level 10^5^-fold (*p* = 0.004, Figure 5a). Transfection of HEK293 with mir-expressing plasmids (i.e., expressing precursors of miR-181a-5p, miR-182-5p, and miR-494-3p) led to a 32-229-fold increase in microRNA expression (Figure 5b). Functionality of sponge-expressing plasmids was verified by co-transfecting HeLa cells with sponge and tested or control microRNA-expression plasmids. In the case of each sponge, co-transfection with a relevant microRNA-expressing plasmid led to a reduction in luciferase activity when compared with cells co-transfected with a plasmid expressing the control pre-microRNA (Figure 5c). In the case of sponge–mix (designed to bind with miR-181a-5p, miR-182, and miR-494-3p), co-transfection with microRNA-expressing plasmids decreased luciferase activity by 31–45% (Figure 5d).

### 2.6. Modulation of Selected MicroRNAs Affects SLC5A8 Expression

**Figure 6:**
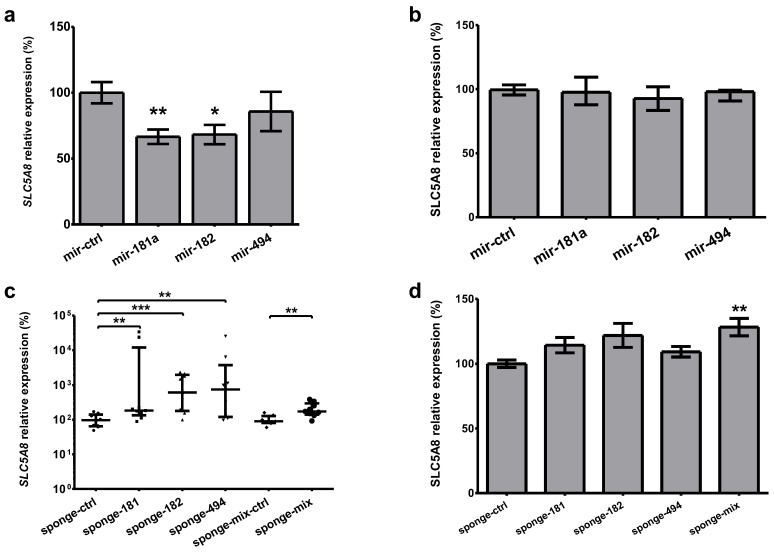
Expression of endogenous *SLC5A8* in the HEK293 cell line transfected with microRNA- or sponge-expressing plasmids. (**a**) Transfection with mir-181a and mir-182 resulted in a reduction in SLC5A8 mRNA measured in real-time PCR assay by 33% (*p* = 0.003) and 32% (*p* = 0.01), respectively. (**b**) The effect of microRNAs overexpression was not visible on the protein level. (**c**) Silencing of miR-181a-5p increased *SLC5A8* mRNA expression 2-fold (*p* = 0.004), for miR-182-5p it was 6-fold (*p* = 0.001), and for miR-494-3p it was 7-fold (*p* = 0.007), whereas simultaneous inhibition of these microRNAs by sponge–mix increased *SLC5A8* expression 2-fold (*p* = 0.006). (**d**) SLC5A8 protein expression was increased by 28% (*p* = 0.002) only upon simultaneous inhibition of the microRNAs by the sponge–mix, whereas the differences in expression of SLC5A8 upon transfection with the other sponges are not significant. Data are expressed as mean values +/− SEM (**a**,**d**) or median +/− interquartile range (**b**,**c**). Statistical analysis was performed using an unpaired *t* test (**a**,**d**) or a Mann–Whitney test ((**b**,**c**) * *p* < 0.05, ** *p* < 0.01, *** *p* < 0.001). A logarithmic scale in (**c**) was used.

Transfection of the HEK293 cell line, exhibiting relatively high mRNA level of *SLC5A8* mRNA, with mir-181a- and mir-182-expression plasmids (i.e., expressing precursors of miR-181a-5p and miR-182-5p, respectively) resulted in a reduction in SLC5A8 mRNA by 33% (*p* = 0.003), and 32% (*p* = 0.01), respectively, whereas transfection with mir-494-expressing plasmid did not alter the *SLC5A8* (Figure 6a). We were not able to show the downregulation of SLC5A8 protein by the overexpression of microRNAs (Figure 6b). Accordingly, transfection with sponge-expressing plasmids led to an increase in *SLC5A8* mRNA expression 2-fold for sponge-181a-5p (*p* = 0.004), 6-fold for sponge-182-5p (*p* = 0.001), and 7-fold for sponge-494-3p (*p* = 0.007), whereas for sponge–mix the increase was 2-fold (*p* = 0.006, Figure 6c). Upon microRNA-sponges transfection, protein expression measured using ELISA was increased by 28% (*p* = 0.002, Figure 6d).

Total RNA isolated from HEK293 cells with the SLC5A8 level modulated through overexpression from plasmids or silencing of three microRNAs using sponge–mix was submitted for whole-transcriptome sequencing, but no significant results were found.

## 3. Discussion

The role of microRNA-mediated gene regulation is a growing matter of interest in thyroid carcinomas. In this study, we show, for the first time, that the microRNAs miR-181a-5p, miR-182-5p, and miR-494-3p directly regulate expression of *SLC5A8*.

Downregulation of *SLC5A8* is related to the process of carcinogenesis and was reported in colon [3], thyroid [10,11,14], prostate [9], pancreas [5], squamous cell head, neck [6], breast [7], lung [8], and cervical [32,33] cancers, as well as in gliomas [4] and acute myeloid leukemia [34]. In colon cancer, a proteomic study suggested a causative role of SLC5A8 [35], that downregulation of *SLC5A8* is an early event in carcinogenesis [36], and that a low protein level is a marker of poor prognosis [15]. In PTC, downregulation of SLC5A8 was demonstrated not only using real-time PCR but also employing immunohistochemistry [11]. Additionally, there were suggestions to employ measuring methylation of *SLC5A8* promoter region in circulating cell-free DNA as a component of diagnostic panels [37,38].

The reasons for the deregulation of *SLC5A8* in differentiated thyroid carcinomas are largely unknown. The promoter methylation depends on BRAF^V600E^ status but is not the only factor affecting *SLC5A8* expression [12]. Methylation of *SLC5A8* is more frequent in the classic than follicular variant of PTC and may be related to extrathyroid invasion and multifocality [13]. Our study confirms that the levels of *SLC5A8* are severely lowered in the presence of the BRAF^V600E^ mutation, which potentially results from the gene’s promoter hypermethylation. *SLC5A8* expression is also lower in the classic compared with the follicular variant of PTC. We could not detect a correlation between *SLC5A8* expression and tumor focality or invasion, which was reported previously [39], nor with tumor size.

We hypothesized that overexpression of microRNAs may contribute to repression of *SLC5A8* in PTC and used in silico and in vitro approaches to test the hypothesis within this study. We confirm that miR-181a-5p, miR-182-5p, and miR-494-3p bind to *SLC5A8* 3′UTR using luciferase assay, and that miR-181a-5p and miR-182-5p are indeed upregulated in PTC compared with normal thyroid tissue obtained from the same patient. We were not able to confirm direct binding of miR-29a/b/c with 3′UTR of *SLC5A8*, which was reported previously [40], probably due to different experimental settings. We do not see any relation between the expression of miR-181a-5p, miR-182-5p, and miR-494-3p and the BRAF^V600E^ mutation status. The result for miR-181a-5p is inconsistent with a previous report which showed higher miR-181a-5p levels in BRAF^V600E^ PTC tumors in the Chinese population [41]. Similarly, miR-182-5p was reported to be BRAF-dependent based on The Cancer Genome Atlas data [42]. The discrepancies between the studies might result from populational (such as iodine nutritional status) or histological differences between the analyzed materials. We did not find a correlation between the expression of *SLC5A8* and any of the investigated microRNAs in our tissue set.

We demonstrate that overexpression of the identified microRNAs suppresses expression of *SLC5A8* mRNA in cell lines, whereas silencing of the microRNAs using microRNA sponges increases *SLC5A8* mRNA levels. We are not able to show statistically significant microRNA-induced deregulation of the SLC5A8 protein, which may result from suboptimal experimental design such as the determination of time points. Nevertheless, in mammals mRNA destabilization is the main effect of microRNA action [43] and mRNA changes are believed to provide a nearly quantitative readout of the microRNA-mediated repression [44], which is also in line with our previous research [31]. Also, inhibition of single-tested microRNA does not increase the protein level, but such an effect is observed upon simultaneous inhibition of miR-181a-5p, miR-182-5p, and miR-494-3p. Such a cumulative effect was reported previously [45], and remains one of the most important findings of this study.

A number of studies showed the ability of SLC5A8 to transport short-chain fatty acids and suggested the role of this transport in tumor suppression through the modulation of histone deacetylases (HDACs) [16], and, consequently, the alteration of the expression of numerous genes including p53, Bax, Bak, TRAIL, TRAILR1, TRAILR2, Survivin, Bcl2, and Bcl-W [46]. Despite the fact that real-time PCR results usually match those obtained using RNA sequencing [47], in this case after correction for multiple comparisons the results of RNA-Seq are not significant. One of the reasons may be that we used embryonic cells, not a tumor cell line. The overall effects of SLC5A8 manipulation on cell function and possible use as a therapeutic agent remains to be elucidated for example, starting with viability or proliferation assays.

There are some other limitations of this study. As we are mainly interested in a PTC context, the main drawback is most likely due to the use of HEK293 cells rather than the thyroid cancer-derived cell line. Human thyroid cancer cell lines exhibit barely detectable levels of *SLC5A8*, similarly to the tumor tissue. It was also not possible to use cell lines derived from other species, such as FRTL5, as the 3′UTR sequences are not conserved between orthologs and microRNA expression varies among species. The function of SLC5A8 in the kidney is well characterized—it is responsible for the sodium-coupled reabsorption of lactate [25,48] and its expression is relatively high. Since the principle of action of microRNAs on the target gene does not depend on cell type, the finding that miR-181a-5p, miR-182-5p, and miR-494-3p directly reduce *SLC5A8* expression in HEK293 cells could be extended to suggest that the overexpression of miR-181a-5p, miR-182-5p, and miR-494-3p account for the reduced expression of *SLC5A8* in PTC, at least in part, and that the inhibition of these microRNAs may partially restore *SLC5A8* expression.

## 4. Materials and Methods

### 4.1. Tissue Samples

Tissue samples were obtained with the permission of the Bioethics Committee of the Medical University of Warsaw (no. KB/184/2009) from patients with papillary thyroid carcinoma and collected at the Department of Genomic Medicine, Medical University of Warsaw. Each patient provided informed consent prior to surgery. The samples consisted of two groups, cancer tissue (n = 49, PTC-T) and control tissue (paired normal tissue from the same patient; n = 49, PTC-N). PTC was diagnosed by histology according to the WHO 2004 criteria. The patients represented all stages of the disease excluding IVB, and 43 (88%) tumors represented the classic variant of PTC (PTC cv) and 6 (12%) were of the follicular variant of PTC (PTC fv). The median tumor diameter was 12 mm (range 1–73 mm). BRAF^V600E^ status was established as described previously [27] in 42 tumors, identifying the mutation in 19 of them. Patient characteristics are summarized in Table 1.

### 4.2. In Silico Identification of MicroRNAs Targeting SLC5A8 Transcripts

MicroRNAs potentially binding *SLC5A8* 3′UTR were predicted using the miRanda (http://microrna.org/, 19 October 2018) algorithm [49] (on 19 October 2018), TargetRank (http://hollywood.mit.edu/targetrank/, results verified on 14 March 2025) [50], DIANA microT (http://diana.imis.athena-innovation.gr/DianaTools/index.php?r=MicroT_CDS, results verified on 14 March 2025 using version 5.0) [51], and TargetScan (https://www.targetscan.org/vert_80/, results verified on 14 March 2025 using version 8.0) [52] (all results last verified on 14 March 2025, besides miRanda). The results were subsequently compared with our previous study, which revealed deregulation of microRNAs in PTC [27]. Only microRNAs with both increased expression in PTC and potentially targeting the *SLC5A8* transcript were considered for further analysis.

### 4.3. MicroRNA Cloning and MicroRNA-Sponges Preparation

The influence of microRNAs on endogenous *SLC5A8* levels was analyzed using microRNA- and microRNA sponge-expressing plasmids. The procedure was similar to that reported in our previous report [31]. Briefly, the sequences encoding the precursors (referred to as mirs) for miR-181a-5p, miR-182-5p, and miR-494-3p were identified based on the Ensembl Database (www.ensembl.org, accessed on 2 May 2014). Primer pairs (Table S1) were created using Clone Manager Professional Suite 8 (Sci-Ed Software, Denver, CO, USA). The specificity of primers was verified using Primer-BLAST (https://www.ncbi.nlm.nih.gov/tools/primer-blast/, last on 14 March 2025). Formation of a hairpin structure by the product was confirmed with RNA Shapes software [53] (https://bibiserv.cebitec.uni-bielefeld.de/rnashapes, on 2 May 2014). Precursors were amplified on the template of DNA isolated from leukocytes of healthy donors and cloned into pcDNA3 plasmid (Life Technologies, Thermo Fisher Scientific, Waltham, MA, USA). As the cloned sequences contained microRNAs precursors, the plasmids are referred to as “mir-expressing”.

Tandem sequences complementary to the recognition sites of selected microRNAs were synthetized, amplified, and cloned into pGL3-MCS plasmid downstream of the luciferase gene [31,54], resulting in microRNA sponges [55]. Additionally, a sequence designed to bind miR-181a-5p, miR-182-5p, and miR-494-3p, having 2 binding sites for each microRNA, was cloned into PGL3-MCS plasmid (Table S1). Their specificity was confirmed using the MirTarget algorithm [56] via www.mirdb.org.

All constructs were sequenced using the Sanger technique. MicroRNA overexpression upon transfection with expressing plasmids was confirmed in HeLa cells using TaqMan kits. MicroRNA sponges were validated in HeLa cells co-transfected with a relevant microRNA-expressing and sponge-expressing plasmid [31].

### 4.4. Analysis of MicroRNA-Mediated Regulation of SLC5A8

Direct binding of microRNAs to SLC5A8 was analyzed in a luciferase assay using the pEZX-SLC5A8 reporter vector (GeneCopoeia, Rockville, MD, USA, cat. no. HmiT003953-MT01) containing the 3′UTR of SLC5A8 cloned downstream of the coding sequence of the firefly luciferase. Constitutively expressed Renilla luciferase served as the internal control. HeLa cells (obtained from ATCC, passage 7th to 12th), which exhibit low expression of all tested microRNAs, were seeded on 12-well plates using 1 × 10^5^ cells per well in 1 mL of Dulbecco’s Modified Eagle’s Medium (DMEM, Lonza, Basel, Switzerland) supplemented with 10% Fetal Bovine Serum (FBS, Biowest, Nuaillé, France) and Penicillin/Streptomycin (P/S, Lonza). After 24 h cells were transfected with 500 ng of the pEZX-SLC5A8 plasmid using polyethylenimine (PEI, Polysciences, Warrington, PA, USA) in DMEM. Transfection mix was prepared as follows: (1) 4 µL PEI (1 µg/µL) was mixed with 125 µL Opti-MEM (Gibco, Thermo Fisher Scientific, Waltham, MA, USA) and (2) 500 ng pEZX-SLC5A8 plasmid was mixed with 125 µL Opti-MEM. After 20 min of incubation at room temperature the solutions were combined, incubated for an additional 20 min, and added to culture wells containing about 740 µL medium without FBS (up to total volume of 1 mL). After an additional 6 h, cells were transfected with synthetic microRNA miR-29a-3p, miR-92b-3p, miR-181a-5p, miR-182-5p, miR-494-3p (Life Technologies, Thermo Fisher Scientific, Waltham, MA, USA), and scrambled negative control (Life Technologies) using Lipofectamie2000 (Life Technologies). A total of 24 h after pEZX-SLC5A8 transfection, cells were subjected to luciferase assay (Promega, Madison, WI, USA) using a GloMax-Multi Detection System (Promega) according to the manufacturer’s instruction.

The effect of microRNAs on endogenous SLC5A8 expression was analyzed through transfection with relevant expression plasmids or sponges. HEK293 cells were seeded in a DMEM medium onto 12-well plates using 2 × 10^5^ cells per well and transfected 24 h later with 400 ng of microRNA-expressing plasmid or 100 ng of sponge-expressing plasmid. After a 96 h of cell incubation, RNA was extracted for gene expression quantification.

### 4.5. Real-Time PCR

Total RNA was extracted as described previously [31]. To establish *SLC5A8* mRNA level, RNAs were reverse transcribed using a SuperScript kit (Life Technologies) according to the manufacturer’s protocol, and gene expression was analyzed in a real-time PCR assay using a Light Cycler 480 (Roche, Basel, Switzerland) using the manufacturer’s protocol and primers listed in Table S1. *HPRT* served as the internal control. Reverse transcription and real-time PCR of microRNAs were performed using TaqMan probes specifically for miR-181a-5p, miR-182-5p, and miR-494-3p, with U44 as an internal control (Life Technologies, cat. no. 000480, 002334, 002365, and 001094, respectively) according to the manufacturer’s protocol. Relative quantification of each expressed microRNA was calculated using the standard 2^−ΔCt^ method. Association between the microRNAs and *SLC5A8* expression in tissue samples was calculated using multiple correlation analysis. BRAF^V600E^ mutation status was checked using a high-resolution melting method as described before [27].

### 4.6. Protein Quantification

In parallel to the transfections described above, cells were seeded onto 6-well plates and transfected using microRNA- or sponge-expressing plasmids using 2-fold higher amounts of all the reagents. Subsequently, the protein was obtained as described previously [31]. A total of 20 μg of total protein was subjected to SLC5A8 quantification using a Human SLC5A8 ELISA kit (Wuhan Fine Biotech, Wuhan, China, cat. no. EH12382), according to the manufacturer’s protocol.

### 4.7. Transcriptome Sequencing

Total RNA isolated from HEK293 cells transfected with pcDNA3-*SLC5A8* [34] or the sponge–mix along with appropriate controls was subjected to transcriptome sequencing (RNA-Seq; performed by Warsaw Genomics, Warsaw, Poland). The Illumina platform was used and sequencing yielded libraries of at least 27 million reads. Reads were demultiplexed and aligned using the Ensembl gene annotation. Log fold change values were compared using false discovery rate correction.

### 4.8. Statistical Analysis

Each experimental series was compared with an appropriate control set. Normally distributed data were analyzed using Student’s *t*-test, non-normally distributed data were analyzed using Wilcoxon and Mann–Whitney U tests, and correlation analysis was performed using Spearman’s rank correlation coefficient (r). An ANOVA test was used for multiple groups comparisons. Statistical analysis was performed using GraphPad Prism 5 (GraphPad Software, La Jolla, CA, USA). *p*-values < 0.05 were considered significant.

## 5. Conclusions

Our data shows that in papillary thyroid cancer (PTC) the overexpression of microRNAs, including miR-181a-5p, miR-182-5p, and miR-494-3p, may contribute to a reduced expression of the tumor suppressor *SLC5A8*. Modulation of those microRNAs may derepress *SLC5A8*, however, further studies are needed to test the in vivo effects of such intervention.

## Figures and Tables

**Table 1:** Characteristics of PTC patients.

Feature		n	(%)
Sex	Female	44	90%
	Male	5	10%
Histopathological subtype	PTC cf	43	88%
	PTC fv	6	12%
No. of foci	Single	39	80%
	Multiple	10	20%
Tumor diameter	Average	12	mm
	Range	1–73	mm
pT feature	pT1a	21	43%
	pT1b	12	24%
	pT2	6	12%
	pT3	10	20%
	pT4	0	0%
pN feature	N0	39	80%
	N1a	5	10%
	N1b	5	10%
cM feature	M0	48	98%
	M1	1	2%
Vascular invasion	No	44	90%
	Yes	5	10%
Local invasion	No	32	65%
	Capsule only	10	20%
	Extrathyroid	7	14%
Stage	I	40	82%
	II	1	2%
	III	5	10%
	IVA	2	4%
	IVB	0	0%
	IVC	1	2%
BRAF status *	T (wild-type)	23	55%
	T/A (mutated)	19	45%

* BRAF status was established in case of 42 patients.

## Data Availability

Data are contained within the article or Supplementary Materials.

## Supplementary Materials

The following supporting information can be downloaded at: https://www.mdpi.com/article/10.3390/ijms26167889/s1.
