# Supplementary material for: MicroRNAs and Their Inhibition in Modulating SLC5A8 Expression in the Context of Papillary Thyroid Carcinoma
Source: Int J Mol Sci. 2025 Aug 15;26(16):7889. doi: 10.3390/ijms26167889 (PMC12386254; doi:10.3390/ijms26167889)
Supplement: Supplementary file 1 [file ijms-26-07889-s001.zip › ijms-3558049-supplementary/Manuscript data/Fig1 data/Data/2013-02-25 1507TN 100ng raport.PDF]

LightCycler® 480 Software
Report

Wojtek_2013-02-25_HPRT AIT BIRC5 NIS

Experiment

Creation Date	2013-02-25 08:24:25	Last Modified Date	2013-02-25 11:16:32
Operator	Genomowa	Owner	System Admin
Start Time	2013-02-25 08:28:46	End Time	2013-02-25 10:01:13
Run State	Completed	Software Version	LCS480 1.5.1.62
Macro		Macro Owner	
Macro Status			
Templates	SYBR Green_AIT_WG	Plate ID	03304775
Test ID		Lot ID	
Color Comp ID			
Run Notes			

Programs

Program Name	pre-incubation		
Cycles	1	Analysis Mode	None

Target (°C)	Acquisition Mode	Hold (hh:mm:ss)	Ramp Rate (°C/s)	Acquisitions (per °C)	Sec Target (°C)	Step size (°C)	Step Delay (cycles)
95	None	00:10:00	4,40		0	0	0

Program Name	amplification		
Cycles	55	Analysis Mode	Quantification

Target (°C)	Acquisition Mode	Hold (hh:mm:ss)	Ramp Rate (°C/s)	Acquisitions (per °C)	Sec Target (°C)	Step size (°C)	Step Delay (cycles)
95	None	00:00:15	4,40		0	0	0
57	None	00:00:15	2,20		0	0	0
72	Single	00:00:15	4,40		0	0	0

Program Name	melting curve		
Cycles	1	Analysis Mode	Melting Curves

Target (°C)	Acquisition Mode	Hold (hh:mm:ss)	Ramp Rate (°C/s)	Acquisitions (per °C)	Sec Target (°C)	Step size (°C)	Step Delay (cycles)
95	None	00:00:05	4,40		0	0	0
65	None	00:01:00	2,20		0	0	0
97	Continuous		0,11	5	0	0	0

Program Name	cooling		
Cycles	1	Analysis Mode	None

Target (°C)	Acquisition Mode	Hold (hh:mm:ss)	Ramp Rate (°C/s)	Acquisitions (per °C)	Sec Target (°C)	Step size (°C)	Step Delay (cycles)

Target (°C)	Acquisition Mode	Hold (hh:mm:ss)	Ramp Rate (°C/s)	Acquisitions (per °C)	Sec Target (°C)	Step size (°C)	Step Delay (cycles)
40	None	00:00:30	2,20		0	0	0

Abs Quant/2nd Derivative Max for All (Abs Quant/2nd Derivative Max)

Settings

Channel	465-510		
Color Compensation	Off		
Program	amplification	Units	
Mode	High Confidence		

Subset Name	All

Results

Inc	Pos	Name	Type	CP	Concentration	Standard	Status
☑	E4	1507T	Unknown	29,04			
☑	E5	1507T	Unknown	28,94			
☑	E6	1507T	Unknown	29,27			
☑	E7	1507T	Unknown	29,47			
☑	E8	1507T	Unknown	29,58			
☑	E9	1507T	Unknown	28,98			
☑	E10	1507T	Unknown	38,11			
☑	E11	1507T	Unknown	35,32			
☑	E12	1507T	Unknown	35,51			
☑	F4	1507T	Unknown	31,07			
☑	F5	1507T	Unknown	31,43			
☑	F6	1507T	Unknown	31,09			
☑	G4	1507N	Unknown	29,67			
☑	G5	1507N	Unknown	30,60			
☑	G6	1507N	Unknown	30,75			
☑	G7	1507N	Unknown	28,88			
☑	G8	1507N	Unknown	29,18			
☑	G9	1507N	Unknown	28,84			
☑	G10	1507N	Unknown	30,23			
☑	G11	1507N	Unknown				
☑	G12	1507N	Unknown	50,00			>
☑	H4	1507N	Unknown	30,01			
☑	H5	1507N	Unknown	29,84			
☑	H6	1507N	Unknown	29,88			

> - Late Cp call (last five cycles) has higher uncertainty

Statistics

Samples	Mean Cp	Std Cp	Mean conc	Std conc
E4, E5, E6	29,08	0,17		
E7, E8, E9	29,34	0,32		
E10, E11, E12	36,31	1,56		
F4, F5, F6	31,20	0,20		
G4, G5, G6	30,34	0,58		
G7, G8, G9	28,97	0,19		
G10, G11, G12	40,12	13,98		
H4, H5, H6	29,91	0,09		

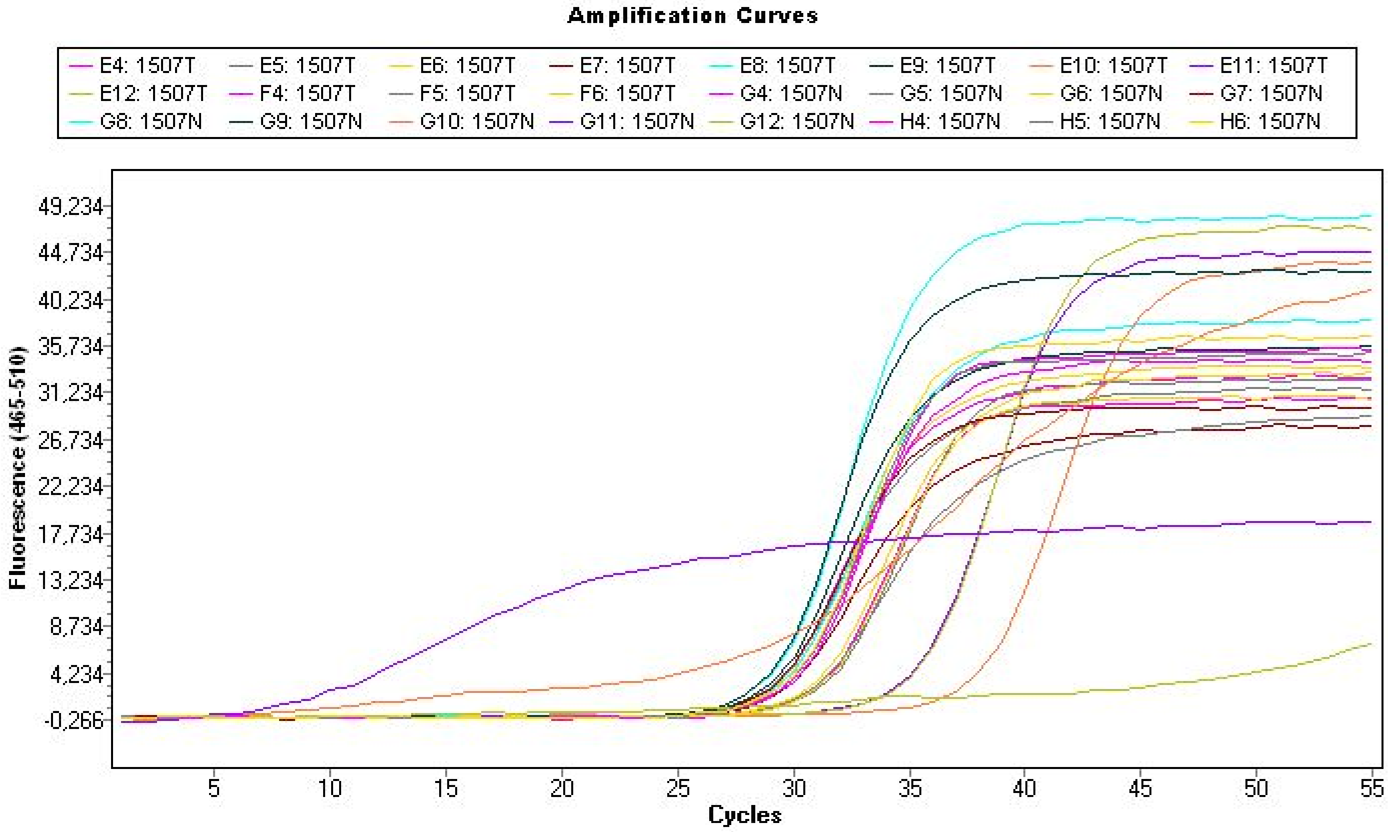

Advanced Relative Quantification for All (Relative Quantification)

Settings

Program Name	amplification		
Subset	All		
AbsQuant Type	Abs Quant/2nd Derivative Max		
Subordinate Analysis Setting	Analyze by Target Name	Mean/Median	Mean
Reference Experiment	In-run		

Target Names

Target ID	Filter Combination	Standards/Efficiency	Efficiency Value
HPRT	465-510	Efficiency	2,00

Target Names

Target ID	Filter Combination	Standards/Efficiency	Efficiency Value
AIT	465-510	Efficiency	2,00
BIRC5	465-510	Efficiency	2,00
NIS	465-510	Efficiency	2,00

Results

Bar Chart	Pairing	Sample Name	Target Name		Tgt Cp	Ref. Cp	Ratios		Corr/Multi Factor	Status
			Targets	References	Mean	Mean	Tgt/Ref.	Norm		
☑	E7/E4	1507T	AIT	HPRT	29,34	29,08	0,8335		1/1	
☑	G7/G4	1507N	AIT	HPRT	28,97	30,34	2,592		1/1	
☑	E10/E4	1507T	BIRC5	HPRT	36,31	29,08	6,65E-3		1/1	
☑	G10/G4	1507N	BIRC5	HPRT	26,74	30,34	Invalid		1/1	
☑	F4/E4	1507T	NIS	HPRT	31,20	29,08	0,2306		1/1	
☑	H4/G4	1507N	NIS	HPRT	29,91	30,34	1,346		1/1	

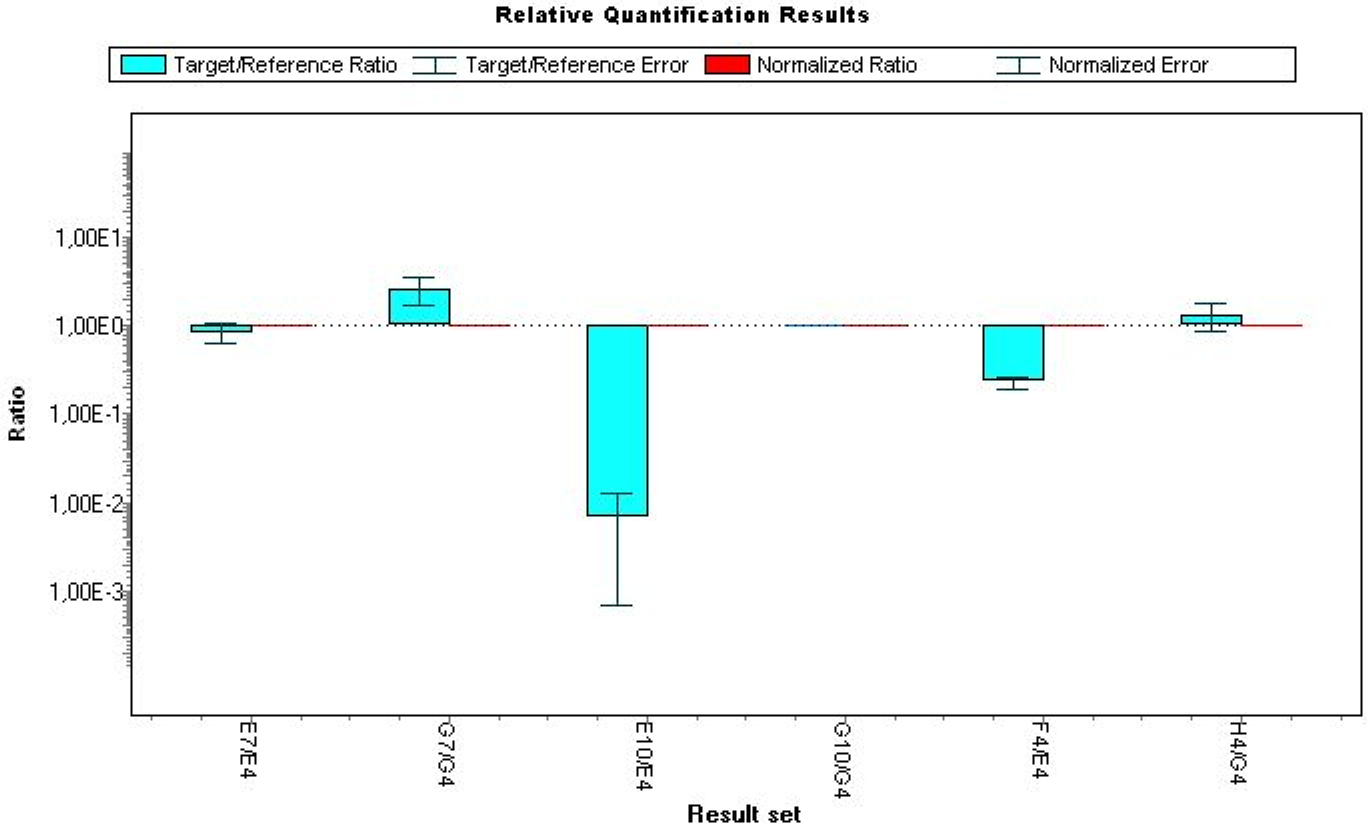

Melt Curve Genotyping for All (Melt Curve Genotyping)

Channel	465-510	Color Compensation	Off				
Progam Name	melting curve						
Stds Settings	Auto-Group						
Sensitivity	Normal	Temp Range	73-91	Score	0,7	Res	0,1

Subset Name	All

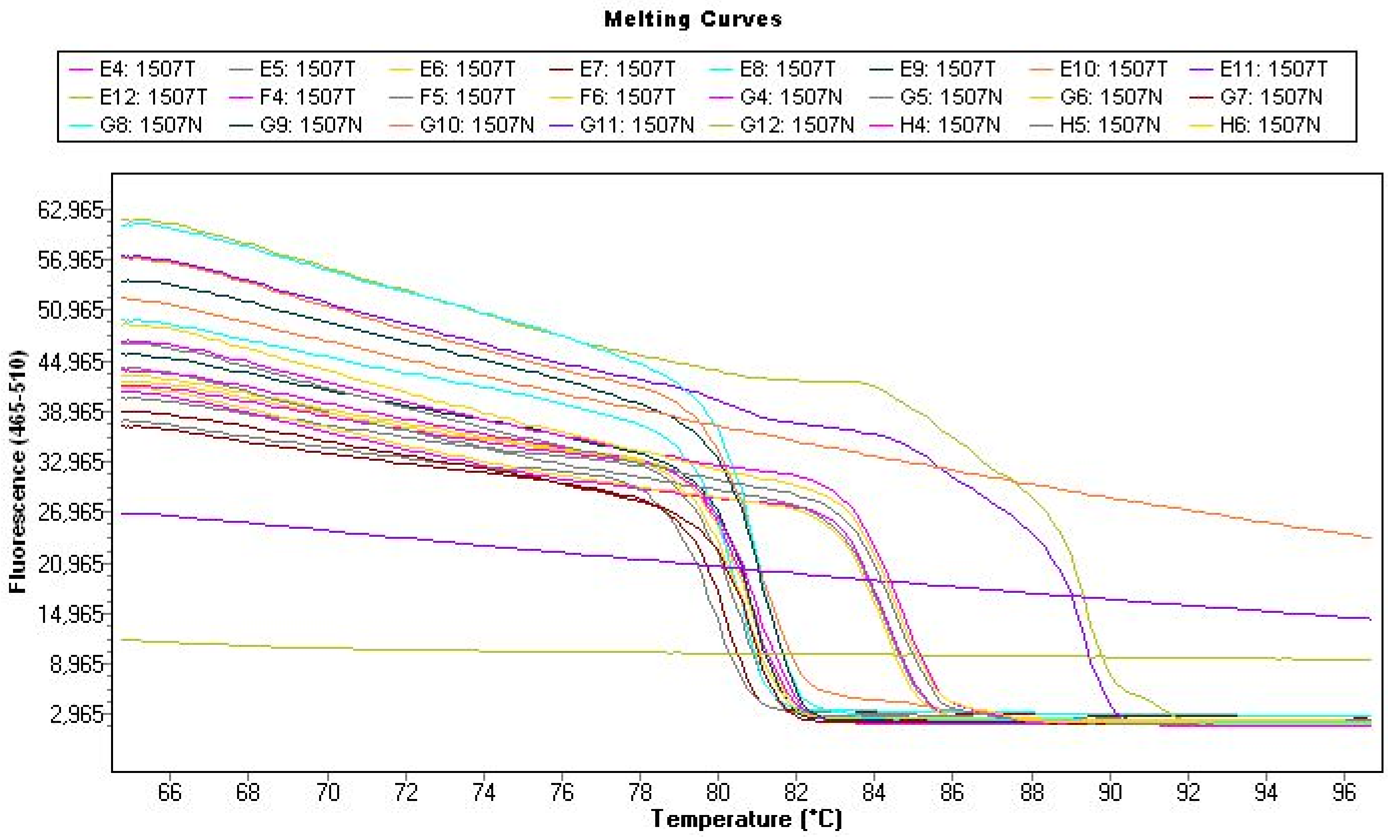
Melting Curves
E4: 1507T
E5: 1507T
E6: 1507T
E7: 1507T
E8: 1507T
E9: 1507T
E10: 1507T
E11: 1507T
E12: 1507T
F4: 1507T
F5: 1507T
F6: 1507T
G4: 1507N
G5: 1507N
G6: 1507N
G7: 1507N
G8: 1507N
G9: 1507N
G10: 1507N
G11: 1507N
G12: 1507N
H4: 1507N
H5: 1507N
H6: 1507N
Fluorescence (465-510)
Temperature (°C)

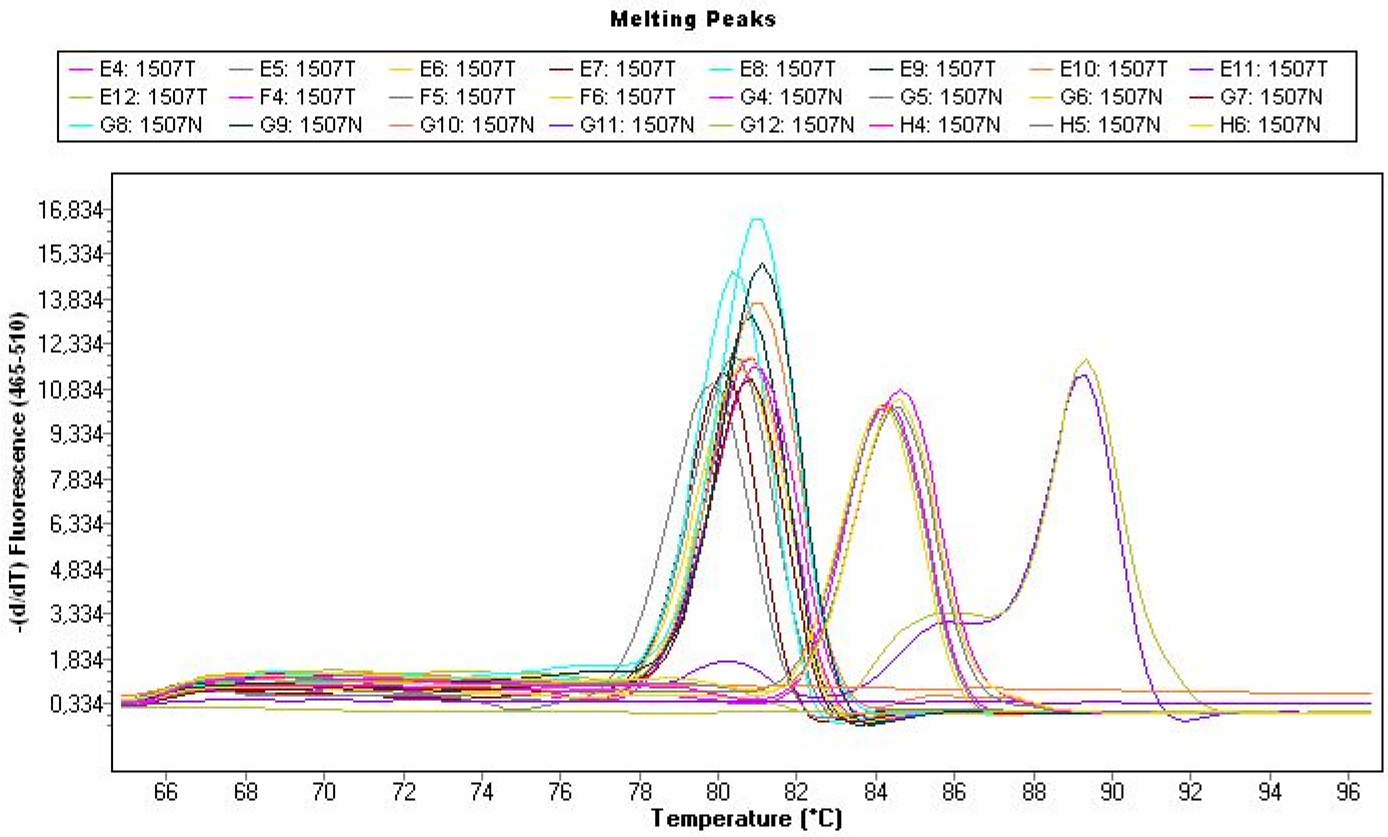
Melting Peaks
E4: 1507T
E5: 1507T
E6: 1507T
E7: 1507T
E8: 1507T
E9: 1507T
E10: 1507T
E11: 1507T
E12: 1507T
F4: 1507T
F5: 1507T
F6: 1507T
G4: 1507N
G5: 1507N
G6: 1507N
G7: 1507N
G8: 1507N
G9: 1507N
G10: 1507N
G11: 1507N
G12: 1507N
H4: 1507N
H5: 1507N
H6: 1507N
-(d/dT) Fluorescence (465-510)
Temperature (°C)
