# Supplementary material for: MicroRNAs and Their Inhibition in Modulating SLC5A8 Expression in the Context of Papillary Thyroid Carcinoma
Source: Int J Mol Sci. 2025 Aug 15;26(16):7889. doi: 10.3390/ijms26167889 (PMC12386254; doi:10.3390/ijms26167889)
Supplement: Supplementary file 1 [file ijms-26-07889-s001.zip › ijms-3558049-supplementary/Manuscript data/Fig1 data/Data/2013-02-25 1507TN 100ng.PDF]

LightCycler® 480 Software

Report

Wojtek_2013-02-25_HPRT AIT BIRC5 NIS

Experiment

Creation Date	2013-02-25 08:24:25	Last Modified Date	2013-02-25 10:37:32
Operator	Genomowa	Owner	System Admin
Start Time	2013-02-25 08:28:46	End Time	2013-02-25 10:01:13
Run State	Completed	Software Version	LCS480 1.5.1.62
Macro		Macro Owner	
Macro Status			
Templates	SYBR Green_AIT_WG	Plate ID	03304775
Test ID		Lot ID	
Color Comp ID			
Run Notes			

Programs

Program Name	pre-incubation		
Cycles	1	Analysis Mode	None

Target (°C)	Acquisition Mode	Hold (hh:mm:ss)	Ramp Rate (°C/s)	Acquisitions (per °C)	Sec Target (°C)	Step size (°C)	Step Delay (cycles)
95	None	00:10:00	4,40		0	0	0

Program Name	amplification		
Cycles	55	Analysis Mode	Quantification

Target (°C)	Acquisition Mode	Hold (hh:mm:ss)	Ramp Rate (°C/s)	Acquisitions (per °C)	Sec Target (°C)	Step size (°C)	Step Delay (cycles)
95	None	00:00:15	4,40		0	0	0
57	None	00:00:15	2,20		0	0	0
72	Single	00:00:15	4,40		0	0	0

Program Name	melting curve		
Cycles	1	Analysis Mode	Melting Curves

Target (°C)	Acquisition Mode	Hold (hh:mm:ss)	Ramp Rate (°C/s)	Acquisitions (per °C)	Sec Target (°C)	Step size (°C)	Step Delay (cycles)
95	None	00:00:05	4,40		0	0	0
65	None	00:01:00	2,20		0	0	0
97	Continuous		0,11	5	0	0	0

Program Name	cooling		
Cycles	1	Analysis Mode	None

Target (°C)	Acquisition Mode	Hold (hh:mm:ss)	Ramp Rate (°C/s)	Acquisitions (per °C)	Sec Target (°C)	Step size (°C)	Step Delay (cycles)

Target (°C)	Acquisition Mode	Hold (hh:mm:ss)	Ramp Rate (°C/s)	Acquisitions (per °C)	Sec Target (°C)	Step size (°C)	Step Delay (cycles)
40	None	00:00:30	2,20		0	0	0

Abs Quant/2nd Derivative Max for All (Abs Quant/2nd Derivative Max)

Results

Inc	Pos	Name	Type	CP	Concentration	Standard	Status
☑	E4	1507T	Unknown	29,04			
☑	E5	1507T	Unknown	28,94			
☑	E6	1507T	Unknown	29,27			
☑	E7	1507T	Unknown	29,47			
☑	E8	1507T	Unknown	29,58			
☑	E9	1507T	Unknown	28,98			
☑	E10	1507T	Unknown	38,11			
☑	E11	1507T	Unknown	35,32			
☑	E12	1507T	Unknown	35,51			
☑	F4	1507T	Unknown	31,07			
☑	F5	1507T	Unknown	31,43			
☑	F6	1507T	Unknown	31,09			
☑	G4	1507N	Unknown	29,67			
☑	G5	1507N	Unknown	30,60			
☑	G6	1507N	Unknown	30,75			
☑	G7	1507N	Unknown	28,88			
☑	G8	1507N	Unknown	29,18			
☑	G9	1507N	Unknown	28,84			
☑	G10	1507N	Unknown	30,23			
☑	G11	1507N	Unknown				
☑	G12	1507N	Unknown	50,00			>
☑	H4	1507N	Unknown	30,01			
☑	H5	1507N	Unknown	29,84			
☑	H6	1507N	Unknown	29,88			

> - Late Cp call (last five cycles) has higher uncertainty

Abs Quant/Fit Points for All (Abs Quant/Fit Points)

Advanced Relative Quantification for All (Relative Quantification)

Melt Curve Genotyping for All (Melt Curve Genotyping)
