# Supplementary material for: MicroRNAs and Their Inhibition in Modulating SLC5A8 Expression in the Context of Papillary Thyroid Carcinoma
Source: Int J Mol Sci. 2025 Aug 15;26(16):7889. doi: 10.3390/ijms26167889 (PMC12386254; doi:10.3390/ijms26167889)
Supplement: Supplementary file 1 [file ijms-26-07889-s001.zip › ijms-3558049-supplementary/Manuscript data/Fig1 data/Data/2013-02-26 1521, 1531, 1534 TN.PDF]

Roche

Wojtek_2013-02-26_HPRT AIT BIRC5 NIS

Programs

Program Name	pre-incubation		
Cycles	1	Analysis Mode	None

Target (°C)	Acquisition Mode	Hold (hh:mm:ss)	Ramp Rate (°C/s)	Acquisitions (per °C)	Sec Target (°C)	Step size (°C)	Step Delay (cycles)
95	None	00:10:00	4,40		0	0	0

Program Name	amplification		
Cycles	50	Analysis Mode	Quantification

Target (°C)	Acquisition Mode	Hold (hh:mm:ss)	Ramp Rate (°C/s)	Acquisitions (per °C)	Sec Target (°C)	Step size (°C)	Step Delay (cycles)
95	None	00:00:15	4,40		0	0	0
57	None	00:00:15	2,20		0	0	0
72	Single	00:00:15	4,40		0	0	0

Program Name	melting curve		
Cycles	1	Analysis Mode	Melting Curves

Target (°C)	Acquisition Mode	Hold (hh:mm:ss)	Ramp Rate (°C/s)	Acquisitions (per °C)	Sec Target (°C)	Step size (°C)	Step Delay (cycles)
95	None	00:00:05	4,40		0	0	0
65	None	00:01:00	2,20		0	0	0
97	Continuous		0,11	5	0	0	0

Program Name	cooling		
Cycles	1	Analysis Mode	None

Target (°C)	Acquisition Mode	Hold (hh:mm:ss)	Ramp Rate (°C/s)	Acquisitions (per °C)	Sec Target (°C)	Step size (°C)	Step Delay (cycles)
40	None	00:00:30	2,20		0	0	0

Abs Quant/2nd Derivative Max for All (Abs Quant/2nd Derivative Max)

Settings

Channel	465-510		
Color Compensation	Off		
Program	amplification	Units	
Mode	High Confidence		

Subset Name	All

Statistics

Samples	Mean Cp	Std Cp	Mean conc	Std conc

Statistics

Samples	Mean Cp	Std Cp	Mean conc	Std conc
A1, A2, A3	29,05	0,95		
A4, A5, A6	31,65	0,31		
A7, A8, A9	34,70	0,50		
B1, B2, B3	28,43	0,89		
B4, B5, B6	28,63	0,51		
B7, B8, B9	32,61	1,12		
C1, C2, C3	26,20	1,27		
C4, C5, C6	29,86	1,54		
C7, C8, C9	37,45	3,42		
D1, D2, D3	28,35	0,61		
D4, D5, D6	27,32	1,28		
D7, D8, D9	33,61	2,32		
E1, E2, E3	25,42	0,98		
E4, E5, E6	27,24	0,58		
E7, E8, E9	27,18	1,07		
F1, F2, F3	27,57	1,14		
F4, F5, F6	25,86	1,29		
F7, F8, F9	29,25	1,06		
G1, G2, G3	28,59	1,69		
G4, G5, G6	26,11	1,20		
G7, G8, G9	31,08	0,39		
H1, H2, H3	26,95	1,76		
H4, H5, H6	26,11	0,56		
H7, H8, H9	26,99	1,20		

Advanced Relative Quantification for All (Relative Quantification)

Settings

Program Name	amplification		
Subset	All		
AbsQuant Type	Abs Quant/2nd Derivative Max		
Subordinate Analysis Setting	Analyze by Target Name	Mean/Median	Mean
Reference Experiment	In-run		

Results

Bar Chart	Pairing	Sample Name	Target Name		Tgt Cp	Ref. Cp	Ratios		Corr/Multi Factor	Status
			Targets	References	Mean	Mean	Tgt/Ref.	Norm		
☑	A4/A1	1557T	AIT	HPRT	31,65	29,05	0,1660		1/1	
☑	B4/B1	1557N	AIT	HPRT	28,63	28,43	0,8696		1/1	
☑	C4/C1	1521T	AIT	HPRT	29,86	26,20	7,90E-2		1/1	

☑	D4/D1	1521N	AIT	HPRT	27,32	28,35	2,041		1/1	
☑	E4/E1	1531T	AIT	HPRT	27,24	25,42	0,2826		1/1	
☑	F4/F1	1531N	AIT	HPRT	25,86	27,57	3,283		1/1	
☑	G4/G1	1534T	AIT	HPRT	26,11	28,59	5,576		1/1	
☑	H4/H1	1534N	AIT	HPRT	26,11	26,95	1,781		1/1	
☑	A7/A1	1557T	NIS	HPRT	34,70	29,05	2,00E-2		1/1	
☑	B7/B1	1557N	NIS	HPRT	32,61	28,43	5,52E-2		1/1	
☑	C7/C1	1521T	NIS	HPRT	37,45	26,20	4,11E-4		1/1	
☑	D7/D1	1521N	NIS	HPRT	33,61	28,35	2,60E-2		1/1	
☑	E7/E1	1531T	NIS	HPRT	27,18	25,42	0,2941		1/1	
☑	F7/F1	1531N	NIS	HPRT	29,25	27,57	0,3128		1/1	
☑	G7/G1	1534T	NIS	HPRT	31,08	28,59	0,1774		1/1	
☑	H7/H1	1534N	NIS	HPRT	26,99	26,95	0,9732		1/1	

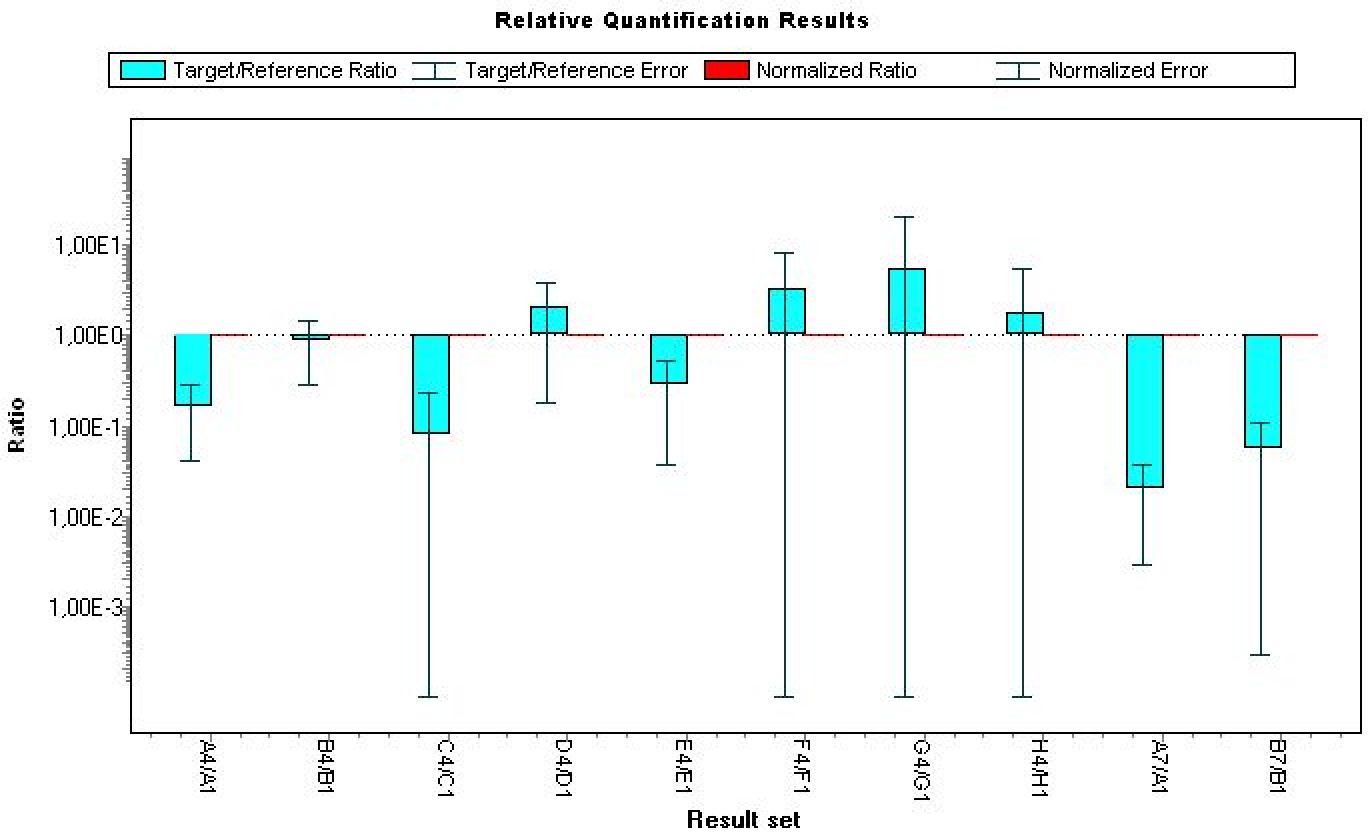

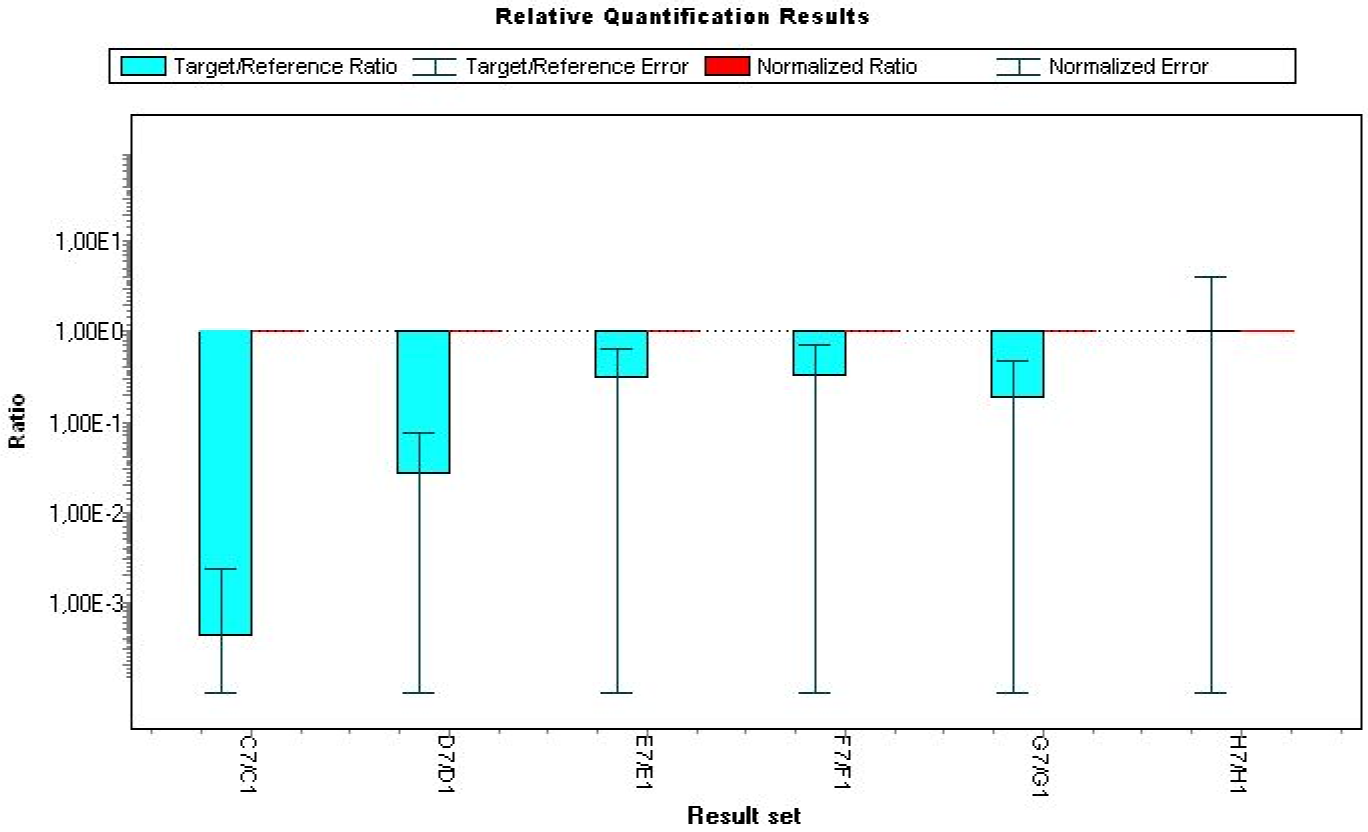

Melt Curve Genotyping for All (Melt Curve Genotyping)

Channel	465-510	Color Compensation	Off				
Progam Name	melting curve						
Stds Settings	Auto-Group						
Sensitivity	Normal	Temp Range	73-88	Score	0,7	Res	0,1

Subset Name	All

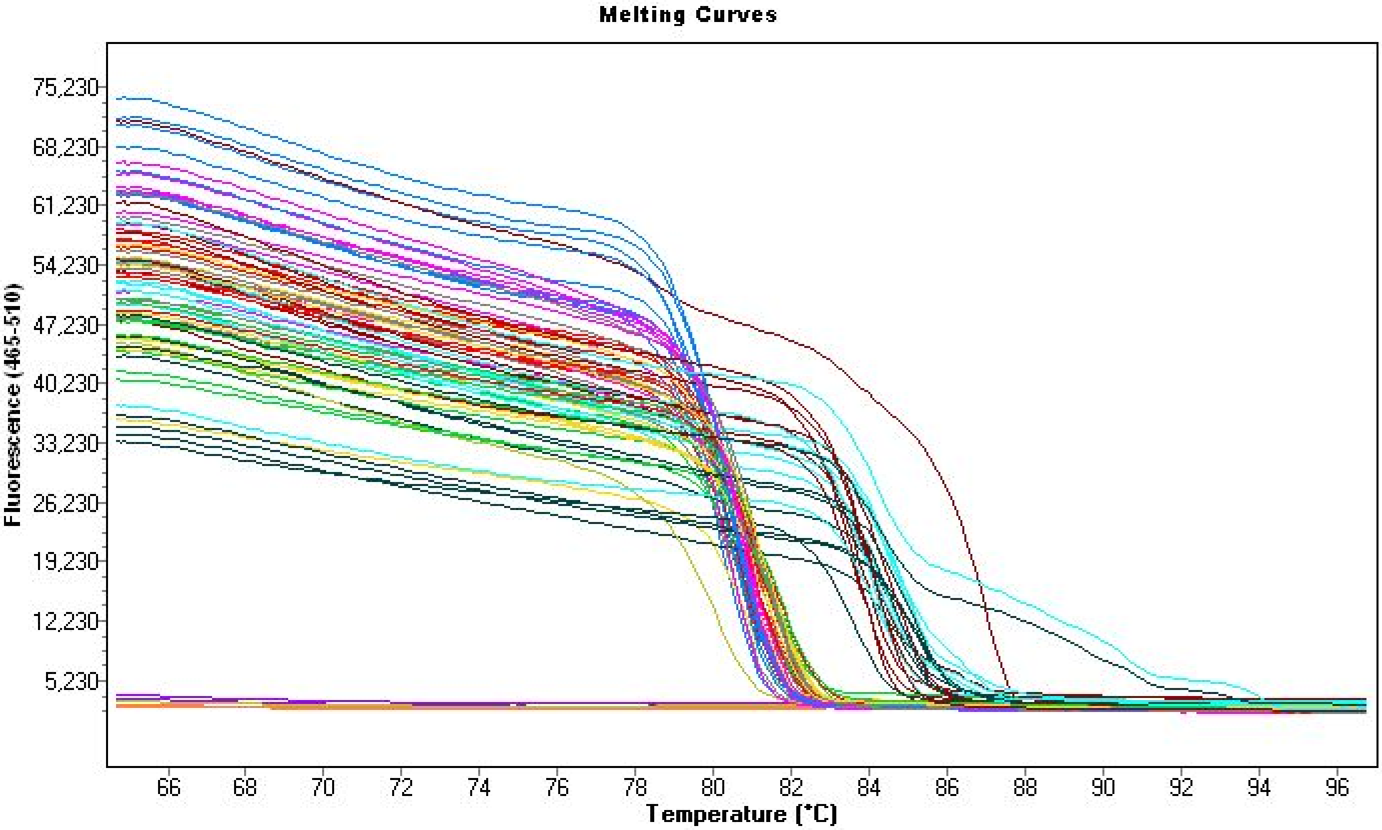
Melting Curves
75,230
68,230
61,230
54,230
47,230
40,230
33,230
26,230
19,230
12,230
5,230
Fluorescence (465-510)
66 68 70 72 74 76 78 80 82 84 86 88 90 92 94 96
Temperature (°C)

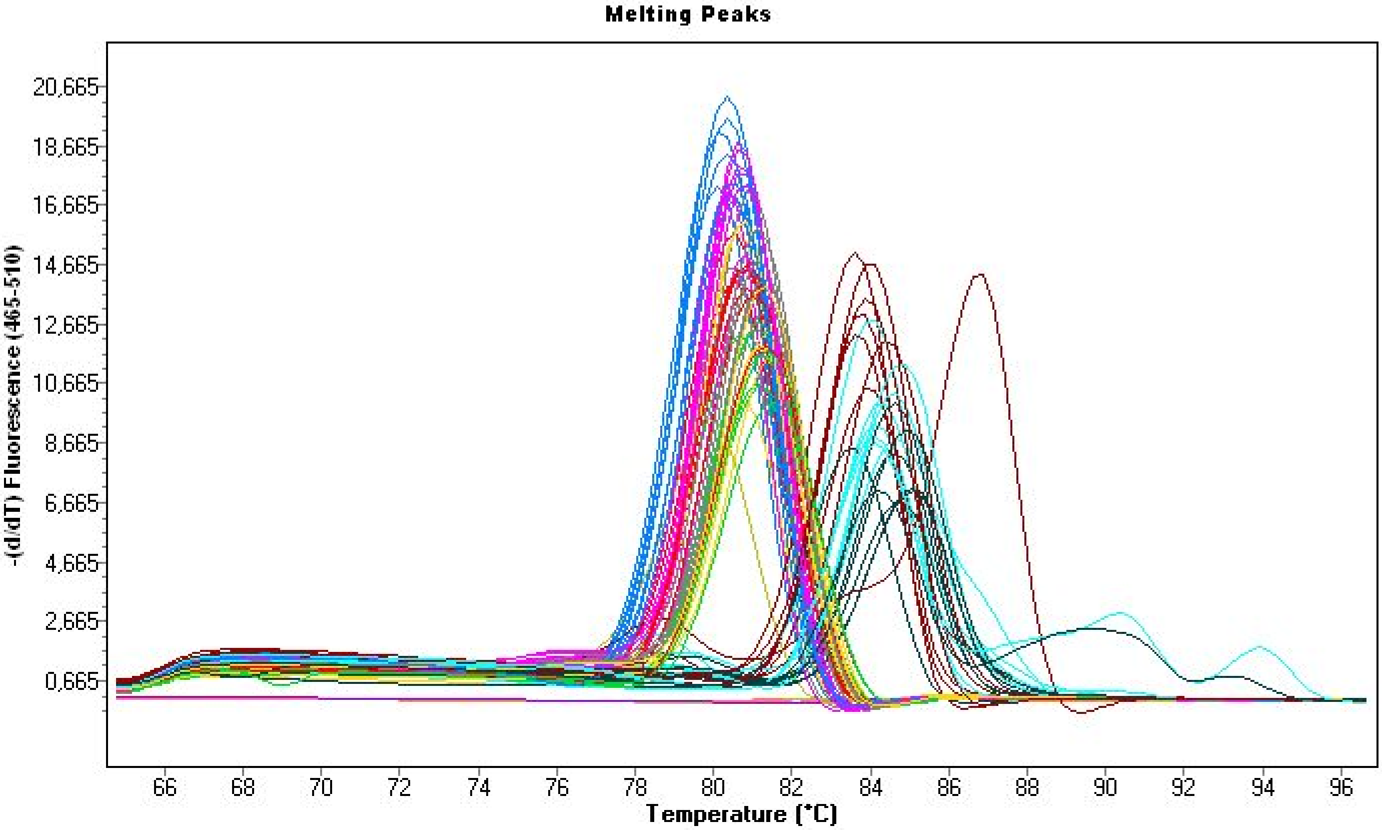
Melting Peaks
20,665
18,665
16,665
14,665
12,665
10,665
8,665
6,665
4,665
2,665
0,665
-(d/dT) Fluorescence (465-510)
66 68 70 72 74 76 78 80 82 84 86 88 90 92 94 96
Temperature (°C)
