# Supplementary material for: MicroRNAs and Their Inhibition in Modulating SLC5A8 Expression in the Context of Papillary Thyroid Carcinoma
Source: Int J Mol Sci. 2025 Aug 15;26(16):7889. doi: 10.3390/ijms26167889 (PMC12386254; doi:10.3390/ijms26167889)
Supplement: Supplementary file 1 [file ijms-26-07889-s001.zip › ijms-3558049-supplementary/Manuscript data/Fig1 data/Data/2013-02-27 HPRT AIT NIS 1507-1534 (2).PDF]

27.02.2013 ii

Programs

Program Name	pre-incubation		
Cycles	1	Analysis Mode	None

Target (°C)	Acquisition Mode	Hold (hh:mm:ss)	Ramp Rate (°C/s)	Acquisitions (per °C)	Sec Target (°C)	Step size (°C)	Step Delay (cycles)
95	None	00:10:00	4,40		0	0	0

Program Name	amplification		
Cycles	50	Analysis Mode	Quantification

Target (°C)	Acquisition Mode	Hold (hh:mm:ss)	Ramp Rate (°C/s)	Acquisitions (per °C)	Sec Target (°C)	Step size (°C)	Step Delay (cycles)
95	None	00:00:15	4,40		0	0	0
57	None	00:00:15	2,20		0	0	0
72	Single	00:00:15	4,40		0	0	0

Program Name	melting curve		
Cycles	1	Analysis Mode	Melting Curves

Target (°C)	Acquisition Mode	Hold (hh:mm:ss)	Ramp Rate (°C/s)	Acquisitions (per °C)	Sec Target (°C)	Step size (°C)	Step Delay (cycles)
95	None	00:00:05	4,40		0	0	0
65	None	00:01:00	2,20		0	0	0
97	Continuous		0,11	5	0	0	0

Program Name	cooling		
Cycles	1	Analysis Mode	None

Target (°C)	Acquisition Mode	Hold (hh:mm:ss)	Ramp Rate (°C/s)	Acquisitions (per °C)	Sec Target (°C)	Step size (°C)	Step Delay (cycles)
40	None	00:00:30	2,20		0	0	0

Melt Curve Genotyping for All Samples (Melt Curve Genotyping)

Results

Inc	Pos.	Sample Name	Sample Type	Group	Score	Resolution	Status
☑	A1	1507T	Unknown	1	0,97	0,59	
☑	A2	1507T	Unknown	1	0,96	0,59	
☑	A3	1507T	Unknown	1	0,96	0,58	
☑	A4	1507N	Unknown	1	0,97	0,57	
☑	A5	1507N	Unknown	1	0,97	0,60	
☑	A6	1507N	Unknown	1	0,96	0,60	

Results

Inc	Pos.	Sample Name	Sample Type	Group	Score	Resolution	Status
☑	A7	1521T	Unknown	Unknown	0,04	0,04	
☑	A8	1521T	Unknown	4	1,00	0,97	
☑	A9	1521T	Unknown	1	0,98	0,59	
☑	A10	1521N	Unknown	1	0,98	0,58	
☑	A11	1521N	Unknown	1	0,97	0,56	
☑	A12	1521N	Unknown	1	0,95	0,60	
☑	B1	1531T	Unknown	1	0,97	0,60	
☑	B2	1531T	Unknown	1	0,97	0,58	
☑	B3	1531T	Unknown	1	0,97	0,59	
☑	B4	1531N	Unknown	1	0,97	0,55	
☑	B5	1531N	Unknown	1	0,98	0,58	
☑	B6	1531N	Unknown	1	0,97	0,55	
☑	B7	1534T	Unknown	1	0,98	0,55	
☑	B8	1534T	Unknown	1	0,98	0,57	
☑	B9	1534T	Unknown	1	0,98	0,57	
☑	B10	1534N	Unknown	1	0,98	0,58	
☑	B11	1534N	Unknown	1	0,97	0,56	
☑	B12	1534N	Unknown	1	0,96	0,56	
☑	C1	1541T	Unknown	1	0,98	0,58	
☑	C2	1541T	Unknown	1	0,97	0,61	
☑	C3	1541T	Unknown	1	0,98	0,57	
☑	C4	1541N	Unknown	1	0,98	0,58	
☑	C5	1541N	Unknown	1	0,97	0,62	
☑	C6	1541N	Unknown	1	0,97	0,56	
☑	C7	H20	Unknown	3	1,00	0,97	
☑	C8	H20	Unknown	3	0,73	0,69	
☑	C9	H20	Unknown	5	1,00	0,51	
☑	C10	H20	Unknown	1	0,91	0,37	
☑	C11	H20	Unknown	2	0,83	0,36	
☑	C12	H20	Unknown	6	1,00	0,51	
☑	D1	1507T	Unknown	1	0,99	0,54	
☑	D2	1507T	Unknown	1	0,98	0,51	
☑	D3	1507T	Unknown	1	0,99	0,56	
☑	D4	1507N	Unknown	1	0,98	0,60	
☑	D5	1507N	Unknown	1	0,99	0,55	
☑	D6	1507N	Unknown	1	0,98	0,53	
☑	D7	1521T	Unknown	1	0,99	0,56	
☑	D8	1521T	Unknown	1	0,98	0,53	

Results

Inc	Pos.	Sample Name	Sample Type	Group	Score	Resolution	Status
☑	D9	1521T	Unknown	1	0,99	0,54	
☑	D10	1521N	Unknown	1	0,99	0,56	
☑	D11	1521N	Unknown	1	1,00	0,57	
☑	D12	1521N	Unknown	1	0,99	0,56	
☑	E1	1531T	Unknown	1	0,99	0,52	
☑	E2	1531T	Unknown	1	0,99	0,51	
☑	E3	1531T	Unknown	1	0,99	0,52	
☑	E4	1531N	Unknown	1	0,99	0,55	
☑	E5	1531N	Unknown	1	0,98	0,53	
☑	E6	1531N	Unknown	1	0,98	0,50	
☑	E7	1534T	Unknown	1	0,98	0,51	
☑	E8	1534T	Unknown	1	0,99	0,54	
☑	E9	1534T	Unknown	1	0,99	0,51	
☑	E10	1534N	Unknown	1	0,99	0,53	
☑	E11	1534N	Unknown	1	0,99	0,51	
☑	E12	1534N	Unknown	1	0,99	0,54	
☑	F1	1541T	Unknown	1	0,99	0,57	
☑	F2	1541T	Unknown	1	0,99	0,54	
☑	F3	1541T	Unknown	1	0,99	0,51	
☑	F4	1541N	Unknown	1	0,99	0,55	
☑	F5	1541N	Unknown	1	0,99	0,55	
☑	F6	1541N	Unknown	1	0,98	0,52	
☑	F7	1541T	Unknown	2	0,85	0,65	
☑	F8	1541T	Unknown	2	0,87	0,65	
☑	F9	1541T	Unknown	2	0,84	0,67	
☑	F10	1541N	Unknown	2	0,89	0,67	
☑	F11	1541N	Unknown	2	0,90	0,74	
☑	F12	1541N	Unknown	2	0,88	0,71	
☑	G1	1507T	Unknown	2	0,93	0,71	
☑	G2	1507T	Unknown	2	0,93	0,67	
☑	G3	1507T	Unknown	2	0,94	0,72	
☑	G4	1507N	Unknown	2	0,92	0,74	
☑	G5	1507N	Unknown	2	0,93	0,71	
☑	G6	1507N	Unknown	2	0,93	0,70	
☑	G7	1521T	Unknown	2	0,93	0,53	
☑	G8	1521T	Unknown	2	0,87	0,45	
☑	G9	1521T	Unknown	2	0,92	0,61	
☑	G10	1521N	Unknown	2	0,96	0,68	

Results

Inc	Pos.	Sample Name	Sample Type	Group	Score	Resolution	Status
☑	G11	1521N	Unknown	2	0,94	0,75	
☑	G12	1521N	Unknown	2	0,92	0,69	
☑	H1	1531T	Unknown	2	0,97	0,78	
☑	H2	1531T	Unknown	2	0,92	0,77	
☑	H3	1531T	Unknown	2	0,97	0,76	
☑	H4	1531N	Unknown	2	0,91	0,72	
☑	H5	1531N	Unknown	2	0,92	0,71	
☑	H6	1531N	Unknown	2	0,92	0,70	
☑	H7	1534T	Unknown	2	1,00	0,74	
☑	H8	1534T	Unknown	2	0,96	0,70	
☑	H9	1534T	Unknown	2	0,97	0,68	
☑	H10	1534N	Unknown	2	0,96	0,76	
☑	H11	1534N	Unknown	2	0,94	0,76	
☑	H12	1534N	Unknown	2	0,94	0,76	

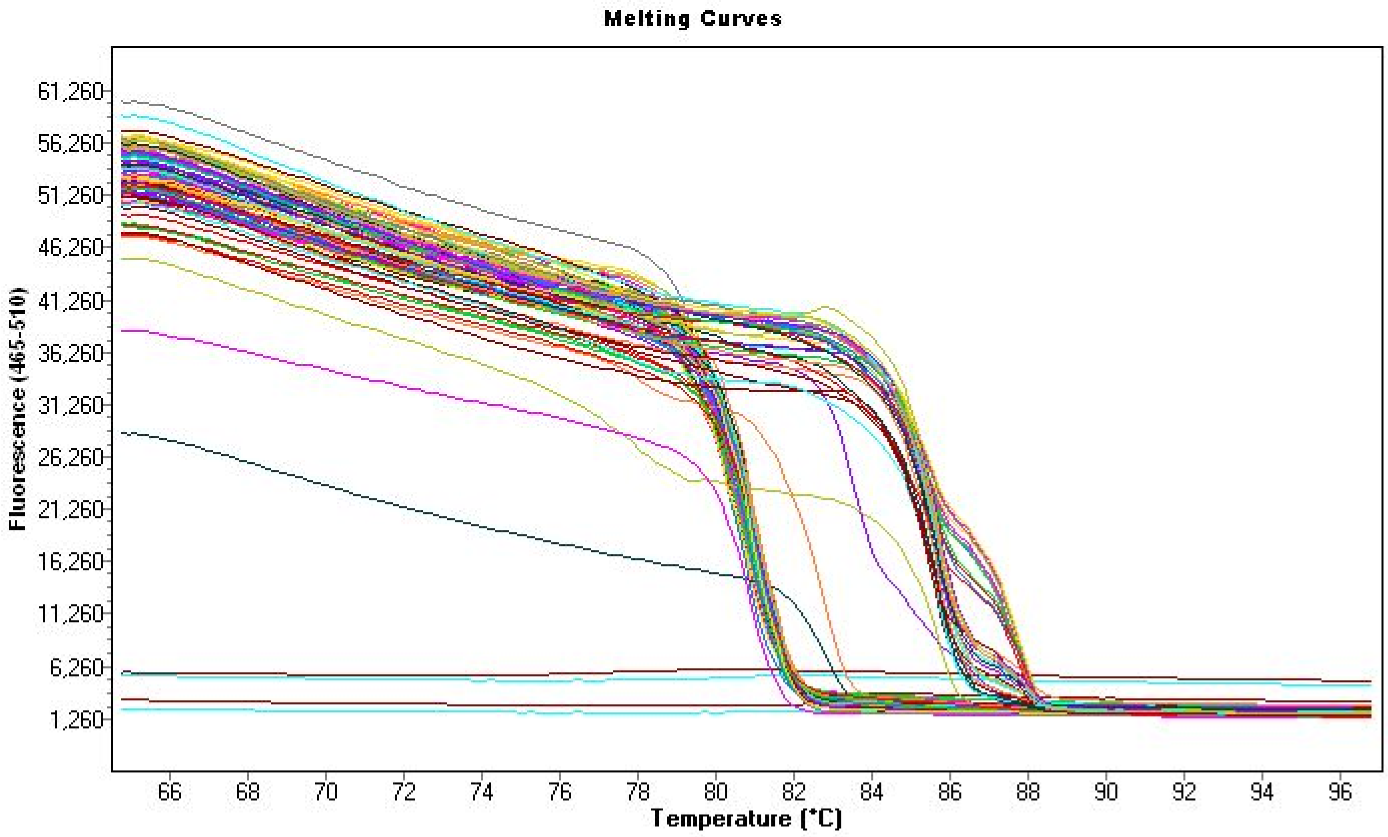

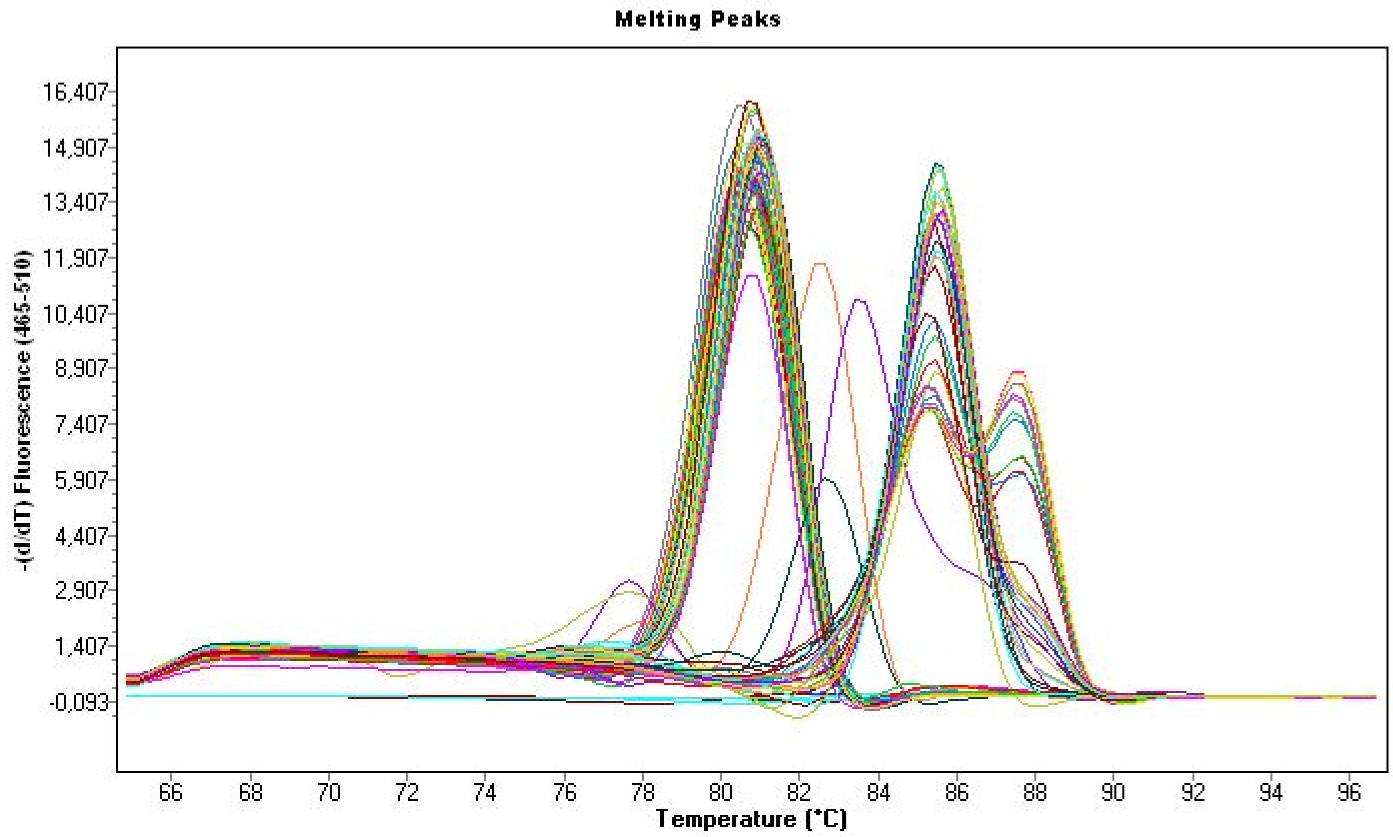

Abs Quant/2nd Derivative Max for All Samples (Abs Quant/2nd Derivative Max)

Statistics

Samples	Mean Cp	Std Cp	Mean conc	Std conc
A1, A2, A3	26,01	0,06		
A4, A5, A6	27,10	0,15		
A7, A8, A9	24,46			
A10, A11, A12	26,92	0,14		
B1, B2, B3	24,08	0,06		
B4, B5, B6	26,08	0,01		
B7, B8, B9	26,99	0,03		
B10, B11, B12	26,38	0,12		
C1, C2, C3	25,39	0,09		
C4, C5, C6	30,55	0,07		
C7, C8				
C10, C9	44,35	0,92		
C11, C12	36,82	2,48		
D1, D2, D3	25,52	0,13		
D4, D5, D6	25,60	0,05		
D7, D8, D9	27,67	0,08		
D10, D11, D12	25,50	0,03		

Statistics

Samples	Mean Cp	Std Cp	Mean conc	Std conc
E1, E2, E3	25,23	0,07		
E4, E5, E6	25,06	0,03		
E7, E8, E9	24,18	0,04		
E10, E11, E12	24,64	0,09		
F1, F2, F3	27,43	0,11		
F4, F5, F6	29,24	0,11		
F7, F8, F9	28,50	0,03		
F10, F11, F12	32,66	0,47		
G1, G2, G3	31,78	0,33		
G4, G5, G6	30,38	0,15		
G7, G8, G9	32,45	0,16		
G10, G11, G12	31,68	0,31		
H1, H2, H3	28,12	0,08		
H4, H5, H6	30,61	0,10		
H7, H8, H9	31,45	0,22		
H10, H11, H12	28,17	0,06		

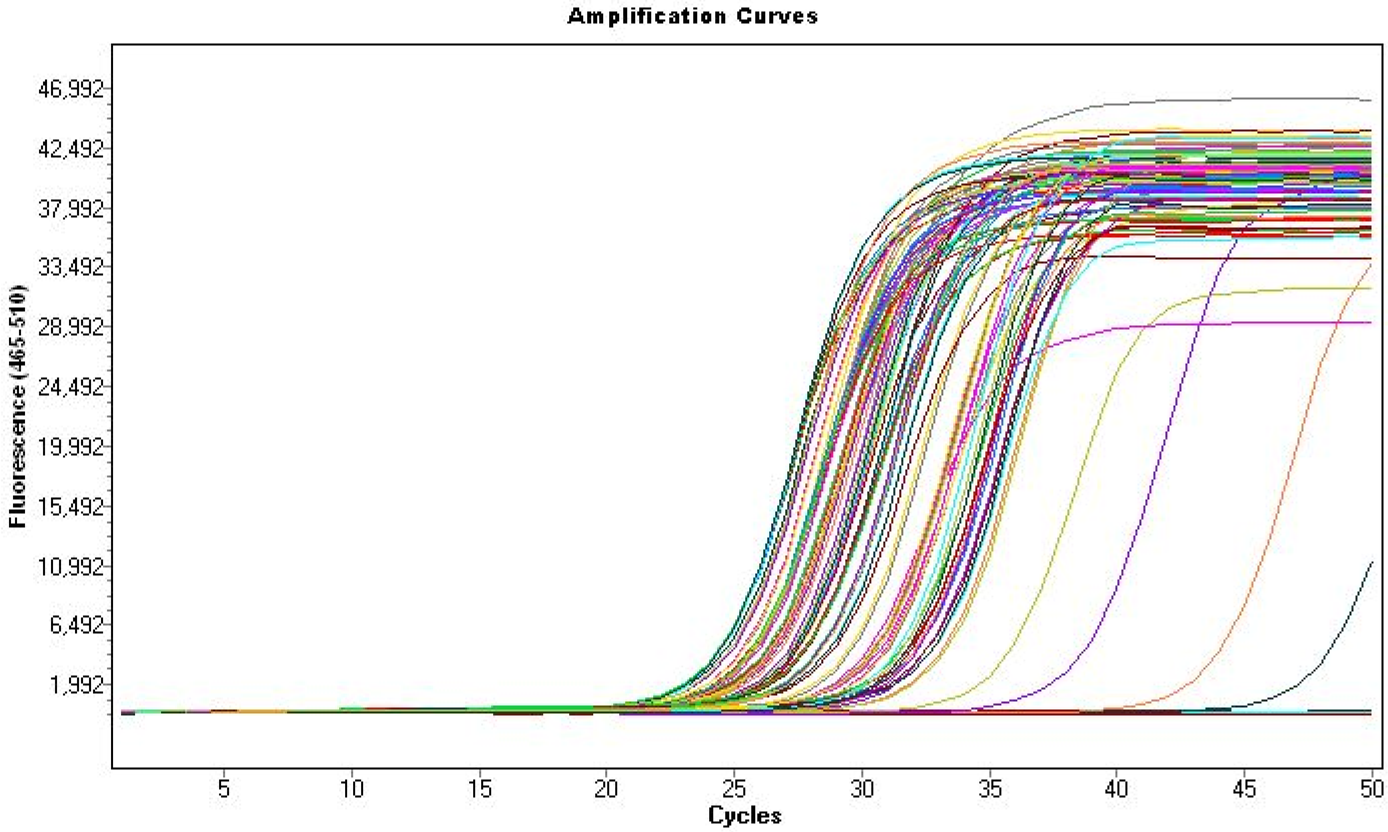

Advanced Relative Quantification for All Samples (Relative Quantification)

Target Names

Target ID	Filter Combination	Standards/Efficiency	Efficiency Value
HPRT	465-510	Efficiency	2,00
AIT	465-510	Efficiency	2,00
NIS	465-510	Efficiency	2,00

Results

Bar Chart	Pairing	Sample Name	Target Name: Targets	Target Name: References	Tgt Cp Mean	Ref. Cp Mean	Ratios: Tgt/Ref.	Ratios: Norm	Corr/Multi Factor	Status
☑	D1/A1	1507T	AIT	HPRT	25,52	26,01	1,405		1/1	
☑	D4/A4	1507N	AIT	HPRT	25,60	27,10	2,843		1/1	
☑	D7/A7	1521T	AIT	HPRT	27,67	8,15	Invalid		1/1	
☑	D10/A10	1521N	AIT	HPRT	25,50	26,92	2,670		1/1	
☑	E1/B1	1531T	AIT	HPRT	25,23	24,08	0,4524		1/1	
☑	E4/B4	1531N	AIT	HPRT	25,06	26,08	2,036		1/1	
☑	E7/B7	1534T	AIT	HPRT	24,18	26,99	7,038		1/1	
☑	E10/B10	1534N	AIT	HPRT	24,64	26,38	3,327		1/1	
☑	F1/C1	1541T	AIT	HPRT	27,43	25,39	0,2426		1/1	
☑	F4/C4	1541N	AIT	HPRT	29,24	30,55	2,477		1/1	
☑	F7/C1	1541T	NIS	HPRT	28,50	25,39	0,1160		1/1	
☑	F10/C4	1541N	NIS	HPRT	32,66	30,55	0,2313		1/1	
☑	G1/A1	1507T	NIS	HPRT	31,78	26,01	1,84E-2		1/1	
☑	G4/A4	1507N	NIS	HPRT	30,38	27,10	0,1034		1/1	
☑	G7/A7	1521T	NIS	HPRT	32,45	8,15	Invalid		1/1	
☑	G10/A10	1521N	NIS	HPRT	31,68	26,92	3,69E-2		1/1	
☑	H1/B1	1531T	NIS	HPRT	28,12	24,08	6,10E-2		1/1	
☑	H4/B4	1531N	NIS	HPRT	30,61	26,08	4,32E-2		1/1	
☑	H7/B7	1534T	NIS	HPRT	31,45	26,99	4,56E-2		1/1	
☑	H10/B10	1534N	NIS	HPRT	28,17	26,38	0,2888		1/1	

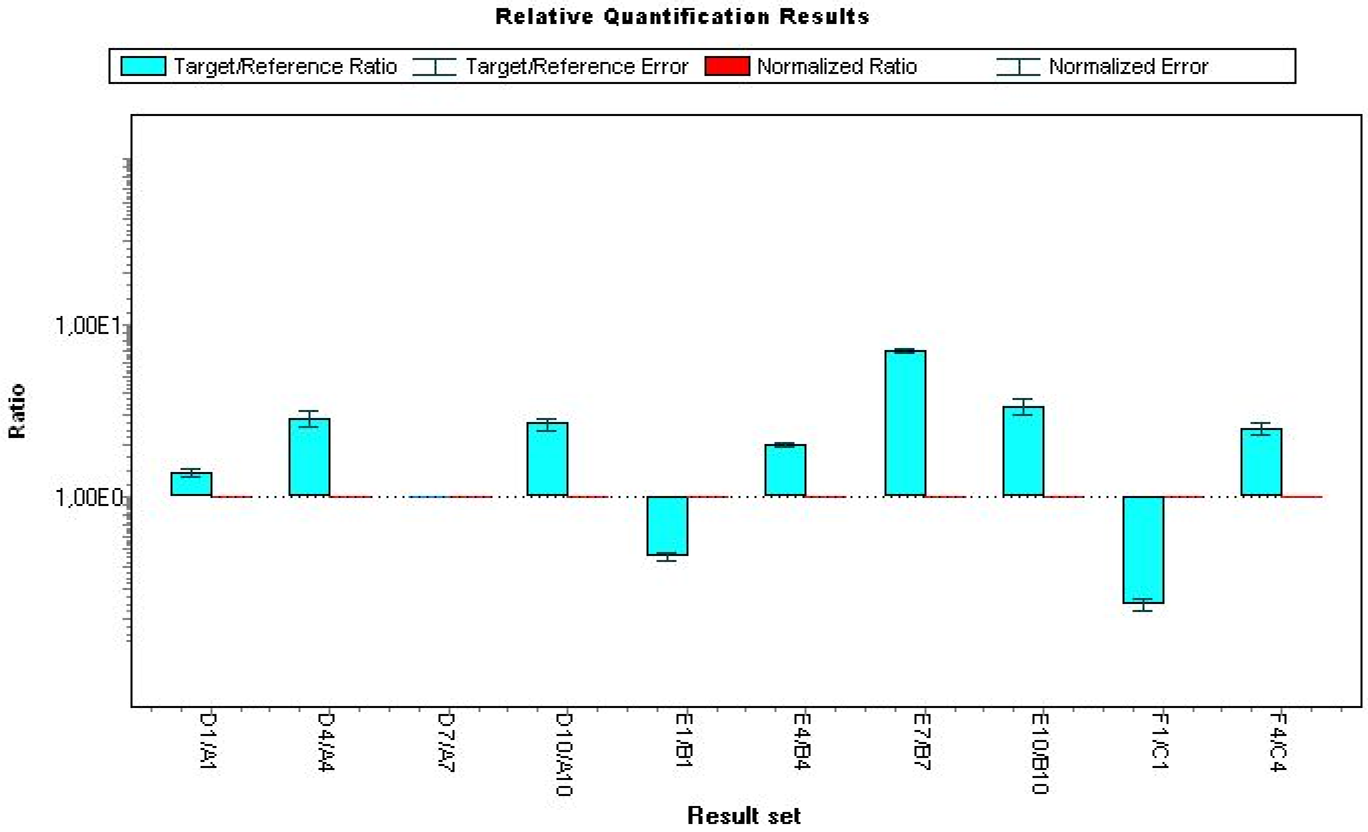
Relative Quantification Results
Target/Reference Ratio
Target/Reference Error
Normalized Ratio
Normalized Error
Ratio
1,00E1
1,00E0
D1/A1
D4/A4
D7/A7
D10/A10
E1/B1
E4/B4
E7/B7
E10/B10
F1/C1
F4/C4
Result set

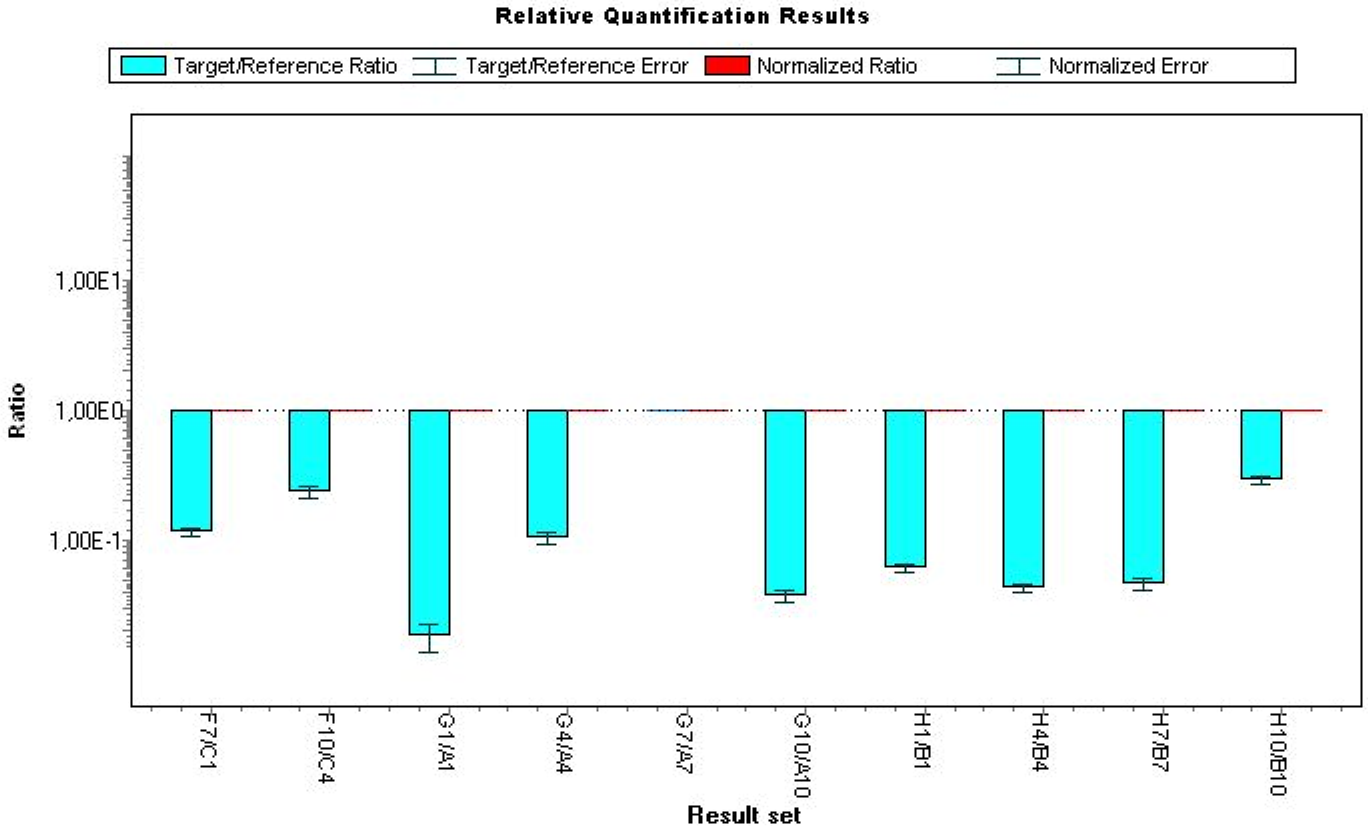
Relative Quantification Results
Target/Reference Ratio
Target/Reference Error
Normalized Ratio
Normalized Error
Ratio
1,00E1
1,00E0
1,00E-1
F7/C1
F10/C4
G1/A1
G4/A4
G7/A7
G10/A10
H1/B1
H4/B4
H7/B7
H10/B10
Result set

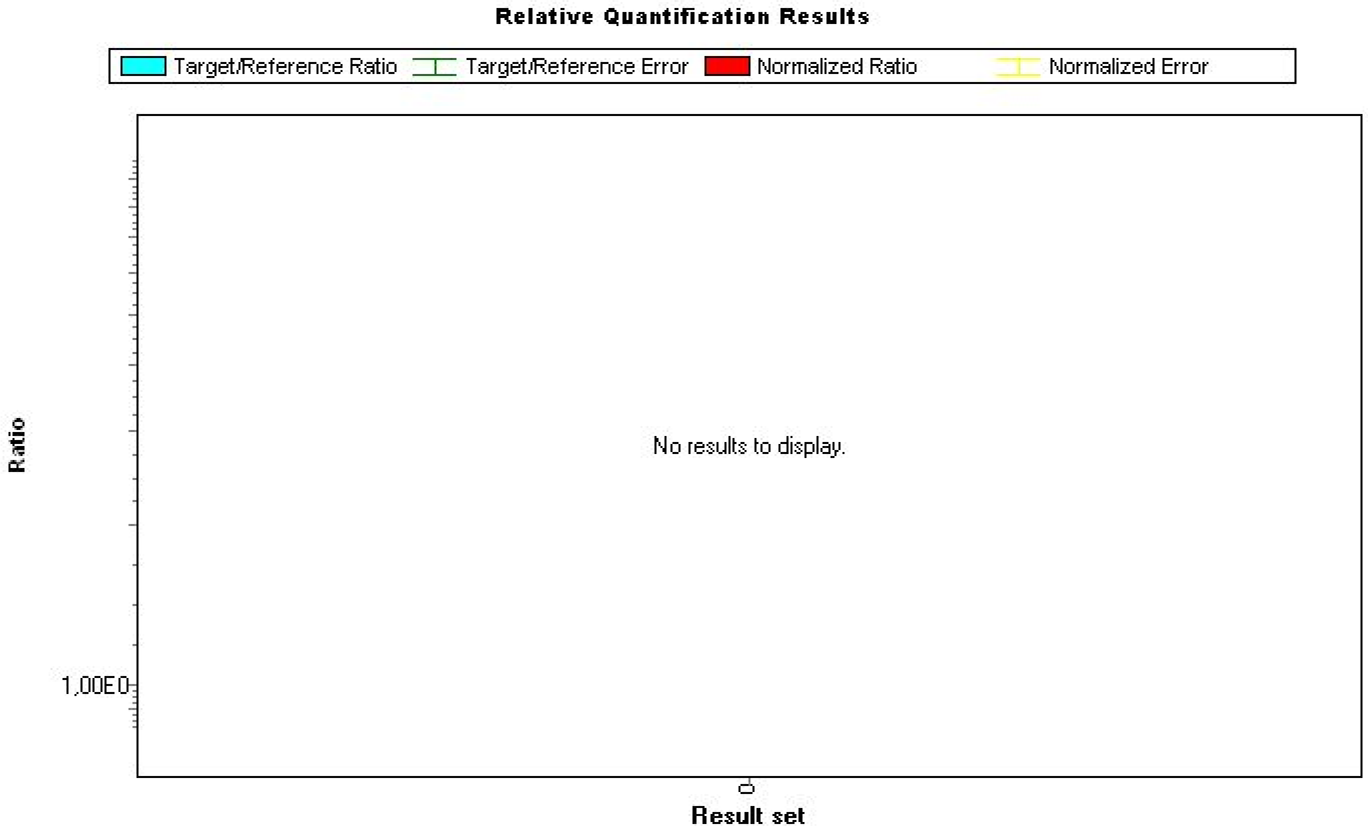
Relative Quantification Results
Target/Reference Ratio
Target/Reference Error
Normalized Ratio
Normalized Error
Ratio
1,00E0
No results to display.
0
Result set
