# Supplementary material for: MicroRNAs and Their Inhibition in Modulating SLC5A8 Expression in the Context of Papillary Thyroid Carcinoma
Source: Int J Mol Sci. 2025 Aug 15;26(16):7889. doi: 10.3390/ijms26167889 (PMC12386254; doi:10.3390/ijms26167889)
Supplement: Supplementary file 1 [file ijms-26-07889-s001.zip › ijms-3558049-supplementary/Manuscript data/Fig1 data/Data/2013-02-28 HPRT AIT NIS 1556-1674 (2).PDF]

Roche

# Wojtek_2013-02-28_HPRT AIT NIS_1

## Programs

| Program Name | pre-incubation | | |
|---|---|---|---|
| Cycles | 1 | Analysis Mode | None |

| Target (°C) | Acquisition Mode | Hold (hh:mm:ss) | Ramp Rate (°C/s) | Acquisitions (per °C) | Sec Target (°C) | Step size (°C) | Step Delay (cycles) |
|---|---|---|---|---|---|---|---|
| 95 | None | 00:10:00 | 4,40 | | 0 | 0 | 0 |

| Program Name | amplification | | |
|---|---|---|---|
| Cycles | 50 | Analysis Mode | Quantification |

| Target (°C) | Acquisition Mode | Hold (hh:mm:ss) | Ramp Rate (°C/s) | Acquisitions (per °C) | Sec Target (°C) | Step size (°C) | Step Delay (cycles) |
|---|---|---|---|---|---|---|---|
| 95 | None | 00:00:15 | 4,40 | | 0 | 0 | 0 |
| 57 | None | 00:00:15 | 2,20 | | 0 | 0 | 0 |
| 72 | Single | 00:00:15 | 4,40 | | 0 | 0 | 0 |

| Program Name | melting curve | | |
|---|---|---|---|
| Cycles | 1 | Analysis Mode | Melting Curves |

| Target (°C) | Acquisition Mode | Hold (hh:mm:ss) | Ramp Rate (°C/s) | Acquisitions (per °C) | Sec Target (°C) | Step size (°C) | Step Delay (cycles) |
|---|---|---|---|---|---|---|---|
| 95 | None | 00:00:05 | 4,40 | | 0 | 0 | 0 |
| 65 | None | 00:01:00 | 2,20 | | 0 | 0 | 0 |
| 97 | Continuous | | 0,11 | 5 | 0 | 0 | 0 |

| Program Name | cooling | | |
|---|---|---|---|
| Cycles | 1 | Analysis Mode | None |

| Target (°C) | Acquisition Mode | Hold (hh:mm:ss) | Ramp Rate (°C/s) | Acquisitions (per °C) | Sec Target (°C) | Step size (°C) | Step Delay (cycles) |
|---|---|---|---|---|---|---|---|
| 40 | None | 00:00:30 | 2,20 | | 0 | 0 | 0 |

## Melt Curve Genotyping for All Samples (Melt Curve Genotyping)

### Results

| Inc | Pos. | Sample Name | Sample Type | Group | Score | Resolution | Status |
|---|---|---|---|---|---|---|---|
| ☑ | A1 | 1556T | Unknown | 1 | 0,97 | 0,53 | |
| ☑ | A2 | 1556T | Unknown | 1 | 0,97 | 0,51 | |
| ☑ | A3 | 1556T | Unknown | 1 | 0,98 | 0,47 | |
| ☑ | A4 | 1556N | Unknown | 1 | 0,97 | 0,48 | |
| ☑ | A5 | 1556N | Unknown | 1 | 0,98 | 0,49 | |
| ☑ | A6 | 1556N | Unknown | 1 | 0,97 | 0,54 | |

**Results**

| Inc | Pos. | Sample Name | Sample Type | Group | Score | Resolution | Status |
|---|---|---|---|---|---|---|---|
| ☑ | A7 | 1557T | Unknown | 1 | 0,97 | 0,51 | |
| ☑ | A8 | 1557T | Unknown | 1 | 0,97 | 0,49 | |
| ☑ | A9 | 1557T | Unknown | 1 | 0,97 | 0,47 | |
| ☑ | A10 | 1557N | Unknown | 1 | 0,96 | 0,51 | |
| ☑ | A11 | 1557N | Unknown | 1 | 0,96 | 0,51 | |
| ☑ | A12 | 1557N | Unknown | 1 | 0,96 | 0,55 | |
| ☑ | B1 | 1596T | Unknown | 1 | 0,97 | 0,54 | |
| ☑ | B2 | 1596T | Unknown | 1 | 0,97 | 0,52 | |
| ☑ | B3 | 1596T | Unknown | 1 | 0,98 | 0,52 | |
| ☑ | B4 | 1596N | Unknown | 1 | 0,98 | 0,50 | |
| ☑ | B5 | 1596N | Unknown | 1 | 0,95 | 0,49 | |
| ☑ | B6 | 1596N | Unknown | 1 | 0,98 | 0,51 | |
| ☑ | B7 | 1560T | Unknown | 1 | 0,97 | 0,51 | |
| ☑ | B8 | 1560T | Unknown | 1 | 0,95 | 0,51 | |
| ☑ | B9 | 1560T | Unknown | 1 | 0,96 | 0,47 | |
| ☑ | B10 | 1560N | Unknown | 1 | 0,93 | 0,50 | |
| ☑ | B11 | 1560N | Unknown | 1 | 0,94 | 0,53 | |
| ☑ | B12 | 1560N | Unknown | 1 | 0,97 | 0,55 | |
| ☑ | C1 | 1674T | Unknown | 1 | 0,97 | 0,51 | |
| ☑ | C2 | 1674T | Unknown | 1 | 0,97 | 0,48 | |
| ☑ | C3 | 1674T | Unknown | 1 | 0,96 | 0,45 | |
| ☑ | C4 | 1674N | Unknown | 1 | 0,95 | 0,31 | |
| ☑ | C5 | 1674N | Unknown | 1 | 0,94 | 0,34 | |
| ☑ | C6 | 1674N | Unknown | 1 | 0,88 | 0,12 | |
| ☑ | C7 | H20 | Unknown | Unknown | | | |
| ☑ | C8 | H20 | Unknown | Unknown | | | |
| ☑ | C9 | H20 | Unknown | Unknown | | | |
| ☑ | C10 | H20 | Unknown | Unknown | | | |
| ☑ | C11 | H20 | Unknown | 4 | 1,00 | 0,62 | |
| ☑ | C12 | H20 | Unknown | 5 | 1,00 | 0,50 | |
| ☑ | D1 | 1556T | Unknown | 1 | 0,96 | 0,36 | |
| ☑ | D2 | 1556T | Unknown | 1 | 0,98 | 0,47 | |
| ☑ | D3 | 1556T | Unknown | 1 | 0,97 | 0,45 | |
| ☑ | D4 | 1556N | Unknown | 1 | 0,98 | 0,49 | |
| ☑ | D5 | 1556N | Unknown | 1 | 0,97 | 0,46 | |
| ☑ | D6 | 1556N | Unknown | 1 | 0,96 | 0,49 | |
| ☑ | D7 | 1557T | Unknown | 1 | 0,95 | 0,46 | |
| ☑ | D8 | 1557T | Unknown | 1 | 0,98 | 0,47 | |

**Results**

| Inc | Pos. | Sample Name | Sample Type | Group | Score | Resolution | Status |
|---|---|---|---|---|---|---|---|
| ☑ | D9 | 1557T | Unknown | 1 | 0,99 | 0,51 | |
| ☑ | D10 | 1557N | Unknown | 1 | 0,98 | 0,55 | |
| ☑ | D11 | 1557N | Unknown | 1 | 0,99 | 0,53 | |
| ☑ | D12 | 1557N | Unknown | 1 | 0,99 | 0,53 | |
| ☑ | E1 | 1596T | Unknown | 1 | 0,97 | 0,48 | |
| ☑ | E2 | 1596T | Unknown | 1 | 0,99 | 0,48 | |
| ☑ | E3 | 1596T | Unknown | 1 | 0,99 | 0,51 | |
| ☑ | E4 | 1596N | Unknown | 1 | 0,98 | 0,42 | |
| ☑ | E5 | 1596N | Unknown | 1 | 1,00 | 0,48 | |
| ☑ | E6 | 1596N | Unknown | 1 | 0,99 | 0,44 | |
| ☑ | E7 | 1560T | Unknown | Unknown | 0,78 | 0,02 | |
| ☑ | E8 | 1560T | Unknown | 3 | 0,74 | 0,15 | |
| ☑ | E9 | 1560T | Unknown | Unknown | 0,76 | 0,08 | |
| ☑ | E10 | 1560N | Unknown | 1 | 0,96 | 0,49 | |
| ☑ | E11 | 1560N | Unknown | 1 | 0,98 | 0,51 | |
| ☑ | E12 | 1560N | Unknown | 1 | 0,95 | 0,55 | |
| ☑ | F1 | 1674T | Unknown | 1 | 0,98 | 0,53 | |
| ☑ | F2 | 1674T | Unknown | 1 | 0,98 | 0,53 | |
| ☑ | F3 | 1674T | Unknown | 1 | 0,99 | 0,51 | |
| ☑ | F4 | 1674N | Unknown | 3 | 1,00 | 0,48 | |
| ☑ | F5 | 1674N | Unknown | 3 | 0,96 | 0,44 | |
| ☑ | F6 | 1674N | Unknown | 6 | 1,00 | 0,55 | |
| ☑ | F7 | 1674T | Unknown | 2 | 0,95 | 0,67 | |
| ☑ | F8 | 1674T | Unknown | 2 | 0,93 | 0,66 | |
| ☑ | F9 | 1674T | Unknown | 2 | 0,94 | 0,55 | |
| ☑ | F10 | 1674N | Unknown | 2 | 0,93 | 0,63 | |
| ☑ | F11 | 1674N | Unknown | 2 | 0,94 | 0,68 | |
| ☑ | F12 | 1674N | Unknown | 2 | 0,97 | 0,70 | |
| ☑ | G1 | 1556T | Unknown | 2 | 0,95 | 0,49 | |
| ☑ | G2 | 1556T | Unknown | 2 | 0,95 | 0,52 | |
| ☑ | G3 | 1556T | Unknown | 2 | 0,93 | 0,44 | |
| ☑ | G4 | 1556N | Unknown | Unknown | 0,67 | 0,40 | |
| ☑ | G5 | 1556N | Unknown | Unknown | 0,69 | 0,34 | |
| ☑ | G6 | 1556N | Unknown | Unknown | 0,69 | 0,45 | |
| ☑ | G7 | 1557T | Unknown | 2 | 0,94 | 0,48 | |
| ☑ | G8 | 1557T | Unknown | 2 | 0,97 | 0,66 | |
| ☑ | G9 | 1557T | Unknown | 2 | 0,94 | 0,71 | |
| ☑ | G10 | 1557N | Unknown | 2 | 0,89 | 0,55 | |

**Results**

| Inc | Pos. | Sample Name | Sample Type | Group | Score | Resolution | Status |
|---|---|---|---|---|---|---|---|
| ☑ | G11 | 1557N | Unknown | 2 | 0,90 | 0,60 | |
| ☑ | G12 | 1557N | Unknown | 2 | 0,87 | 0,59 | |
| ☑ | H1 | 1596T | Unknown | 2 | 0,99 | 0,60 | |
| ☑ | H2 | 1596T | Unknown | 2 | 0,98 | 0,63 | |
| ☑ | H3 | 1596T | Unknown | 2 | 0,98 | 0,70 | |
| ☑ | H4 | 1596N | Unknown | 2 | 0,98 | 0,67 | |
| ☑ | H5 | 1596N | Unknown | 2 | 1,00 | 0,64 | |
| ☑ | H6 | 1596N | Unknown | 2 | 0,99 | 0,67 | |
| ☑ | H7 | 1560T | Unknown | 2 | 0,99 | 0,66 | |
| ☑ | H8 | 1560T | Unknown | 2 | 0,99 | 0,68 | |
| ☑ | H9 | 1560T | Unknown | 2 | 0,96 | 0,72 | |
| ☑ | H10 | 1560N | Unknown | 2 | 0,89 | 0,47 | |
| ☑ | H11 | 1560N | Unknown | 2 | 0,91 | 0,55 | |
| ☑ | H12 | 1560N | Unknown | 2 | 0,90 | 0,55 | |

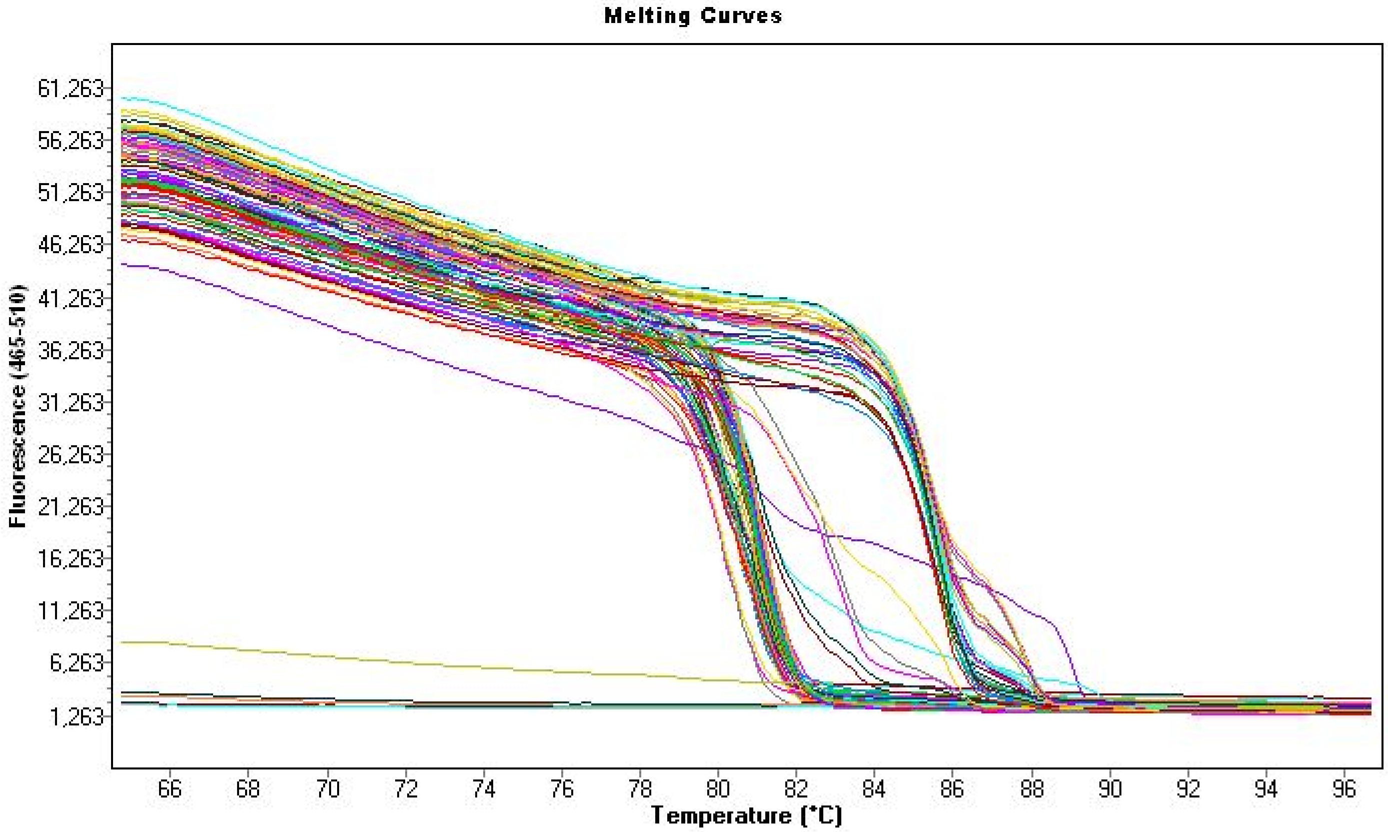

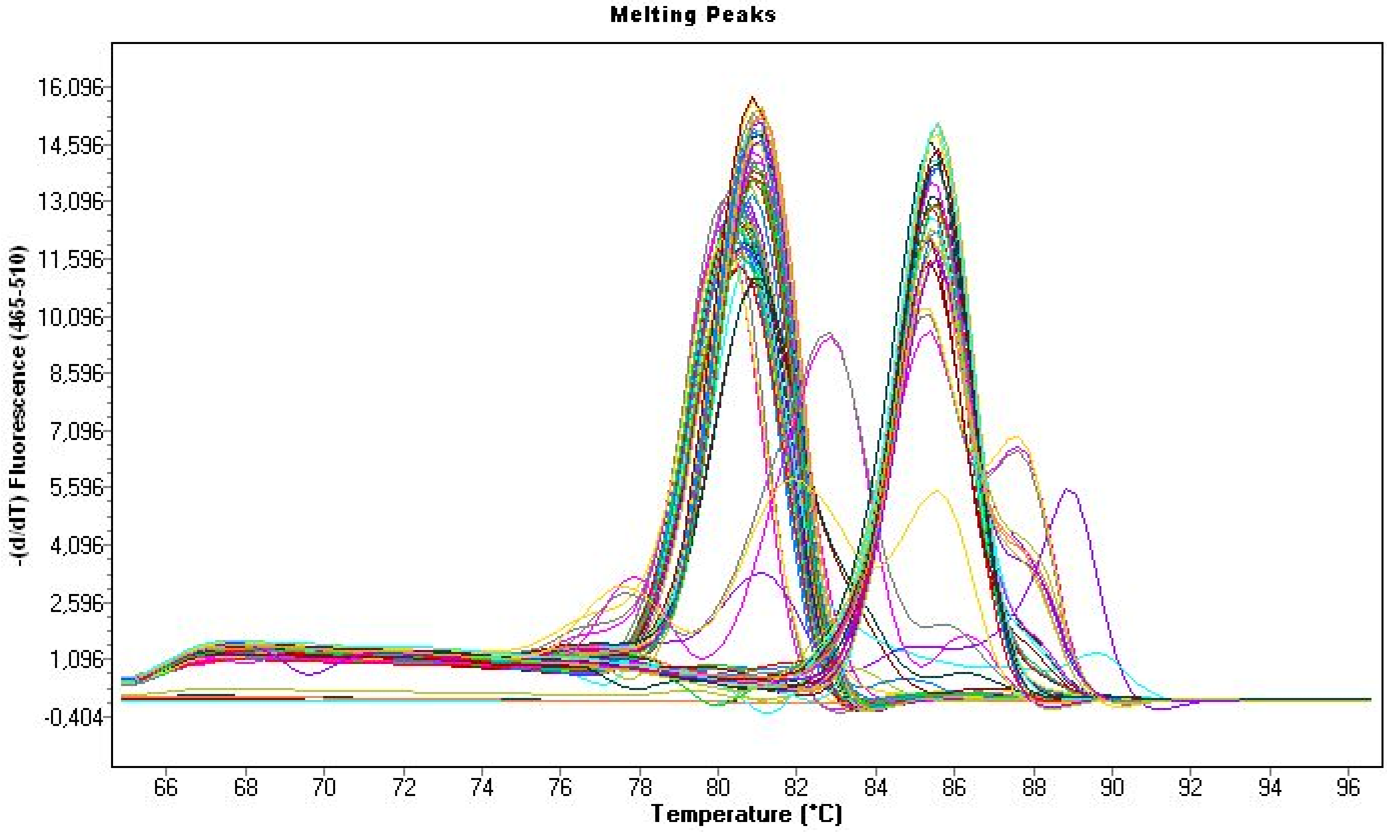


## Abs Quant/2nd Derivative Max for All Samples (Abs Quant/2nd Derivative Max)

### Statistics

| Samples | Mean Cp | Std Cp | Mean conc | Std conc |
|---|---|---|---|---|
| A1, A2, A3 | 26,02 | 0,14 | | |
| A4, A5, A6 | 27,29 | 0,12 | | |
| A7, A8, A9 | 26,13 | 0,05 | | |
| A10, A11, A12 | 26,42 | 0,08 | | |
| B1, B2, B3 | 29,85 | 0,16 | | |
| B4, B5, B6 | 34,11 | 0,94 | | |
| B7, B8, B9 | 26,09 | 0,06 | | |
| B10, B11, B12 | 28,10 | 0,03 | | |
| C1, C2, C3 | 26,43 | 0,09 | | |
| C4, C5, C6 | 35,77 | 0,51 | | |
| C7, C8 | | | | |
| C10, C9 | | | | |
| C11, C12 | 45,00 | | | |
| D1, D2, D3 | 29,84 | 0,04 | | |
| D4, D5, D6 | 26,17 | 0,06 | | |
| D7, D8, D9 | 27,91 | 0,09 | | |
| D10, D11, D12 | 25,95 | 0,06 | | |

**Statistics**

| Samples | Mean Cp | Std Cp | Mean conc | Std conc |
|---|---|---|---|---|
| E1, E2, E3 | 29,14 | 0,20 | | |
| E4, E5, E6 | 32,07 | 0,22 | | |
| E7, E8, E9 | 31,39 | 0,34 | | |
| E10, E11, E12 | 26,97 | 0,07 | | |
| F1, F2, F3 | 25,93 | 0,06 | | |
| F4, F5, F6 | 35,80 | 0,34 | | |
| F7, F8, F9 | 27,40 | 0,27 | | |
| F10, F11, F12 | 29,95 | 0,21 | | |
| G1, G2, G3 | 31,96 | 0,16 | | |
| G4, G5, G6 | 27,65 | 0,09 | | |
| G7, G8, G9 | 31,35 | 0,25 | | |
| G10, G11, G12 | 30,62 | 0,13 | | |
| H1, H2, H3 | 30,33 | 0,11 | | |
| H4, H5, H6 | 31,07 | 0,18 | | |
| H7, H8, H9 | 28,71 | 0,06 | | |
| H10, H11, H12 | 30,59 | 0,09 | | |

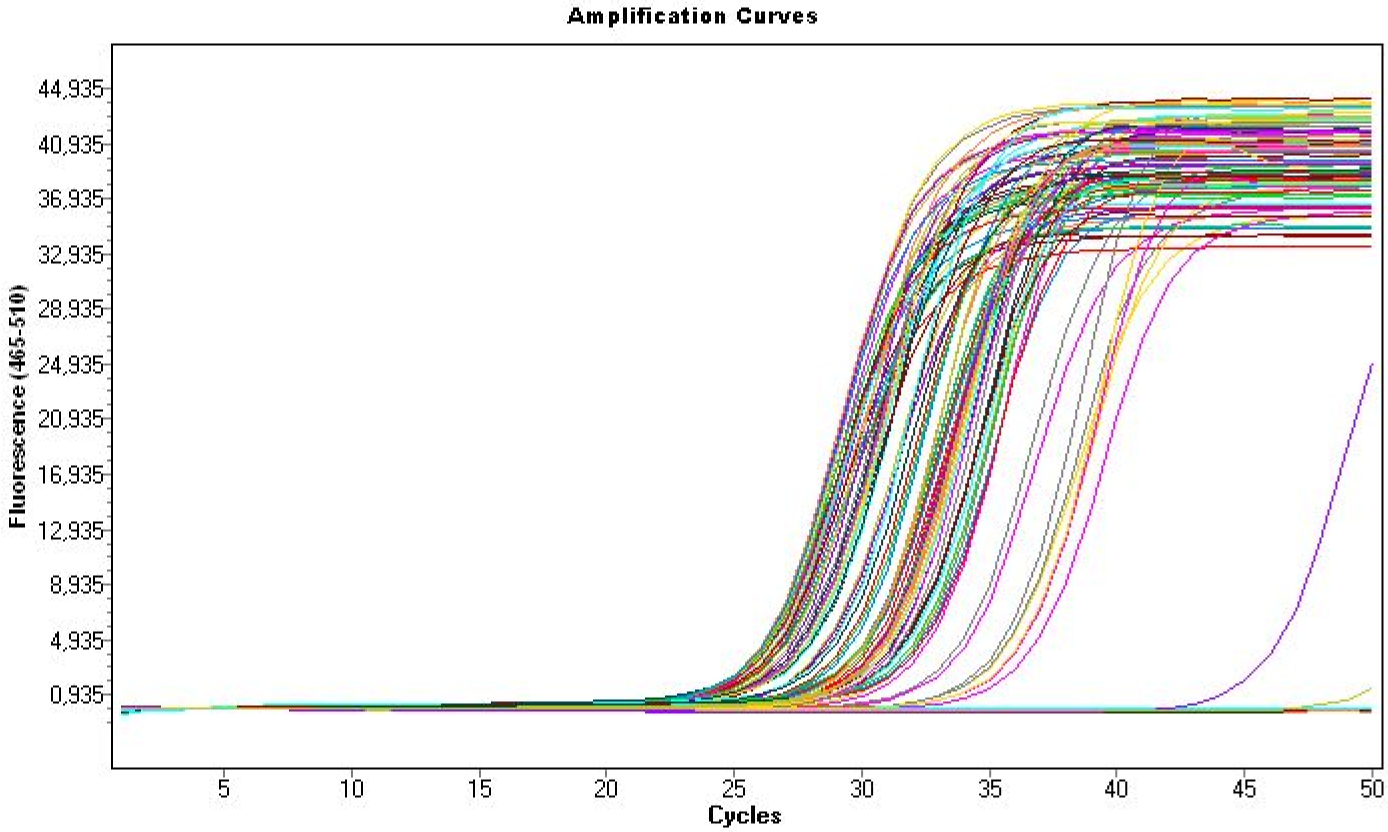


## Advanced Relative Quantification for All Samples (Relative Quantification)

**Target Names**

| Target ID | Filter Combination | Standards/Efficiency | Efficiency Value |
|---|---|---|---|
| HPRT | 465-510 | Efficiency | 2,00 |
| AIT | 465-510 | Efficiency | 2,00 |
| NIS | 465-510 | Efficiency | 2,00 |

**Results**

| Bar Chart | Pairing | Sample Name | Target Name | | Tgt Cp | Ref. Cp | Ratios | | Corr/Multi Factor | Status |
|---|---|---|---|---|---|---|---|---|---|---|
| | | | Targets | References | Mean | Mean | Tgt/Ref. | Norm | | |
| ☑ | D1/A1 | 1556T | AIT | HPRT | 29,84 | 26,02 | 7,08E-2 | | 1/1 | |
| ☑ | D4/A4 | 1556N | AIT | HPRT | 26,17 | 27,29 | 2,170 | | 1/1 | |
| ☑ | D7/A7 | 1557T | AIT | HPRT | 27,91 | 26,13 | 0,2921 | | 1/1 | |
| ☑ | D10/A10 | 1557N | AIT | HPRT | 25,95 | 26,42 | 1,386 | | 1/1 | |
| ☑ | E1/B1 | 1596T | AIT | HPRT | 29,14 | 29,85 | 1,631 | | 1/1 | |
| ☑ | E4/B4 | 1596N | AIT | HPRT | 32,07 | 34,11 | 4,104 | | 1/1 | |
| ☑ | E7/B7 | 1560T | AIT | HPRT | 31,39 | 26,09 | 2,55E-2 | | 1/1 | |
| ☑ | E10/B10 | 1560N | AIT | HPRT | 26,97 | 28,10 | 2,194 | | 1/1 | |
| ☑ | F1/C1 | 1674T | AIT | HPRT | 25,93 | 26,43 | 1,417 | | 1/1 | |
| ☑ | F4/C4 | 1674N | AIT | HPRT | 35,80 | 35,77 | 0,9818 | | 1/1 | |
| ☑ | F7/C1 | 1674T | NIS | HPRT | 27,40 | 26,43 | 0,5107 | | 1/1 | |
| ☑ | F10/C4 | 1674N | NIS | HPRT | 29,95 | 35,77 | 56,35 | | 1/1 | |
| ☑ | G1/A1 | 1556T | NIS | HPRT | 31,96 | 26,02 | 1,62E-2 | | 1/1 | |
| ☑ | G4/A4 | 1556N | NIS | HPRT | 27,65 | 27,29 | 0,7812 | | 1/1 | |
| ☑ | G7/A7 | 1557T | NIS | HPRT | 31,35 | 26,13 | 2,69E-2 | | 1/1 | |
| ☑ | G10/A10 | 1557N | NIS | HPRT | 30,62 | 26,42 | 5,45E-2 | | 1/1 | |
| ☑ | H1/B1 | 1596T | NIS | HPRT | 30,33 | 29,85 | 0,7128 | | 1/1 | |
| ☑ | H4/B4 | 1596N | NIS | HPRT | 31,07 | 34,11 | 8,206 | | 1/1 | |
| ☑ | H7/B7 | 1560T | NIS | HPRT | 28,71 | 26,09 | 0,1627 | | 1/1 | |
| ☑ | H10/B10 | 1560N | NIS | HPRT | 30,59 | 28,10 | 0,1784 | | 1/1 | |

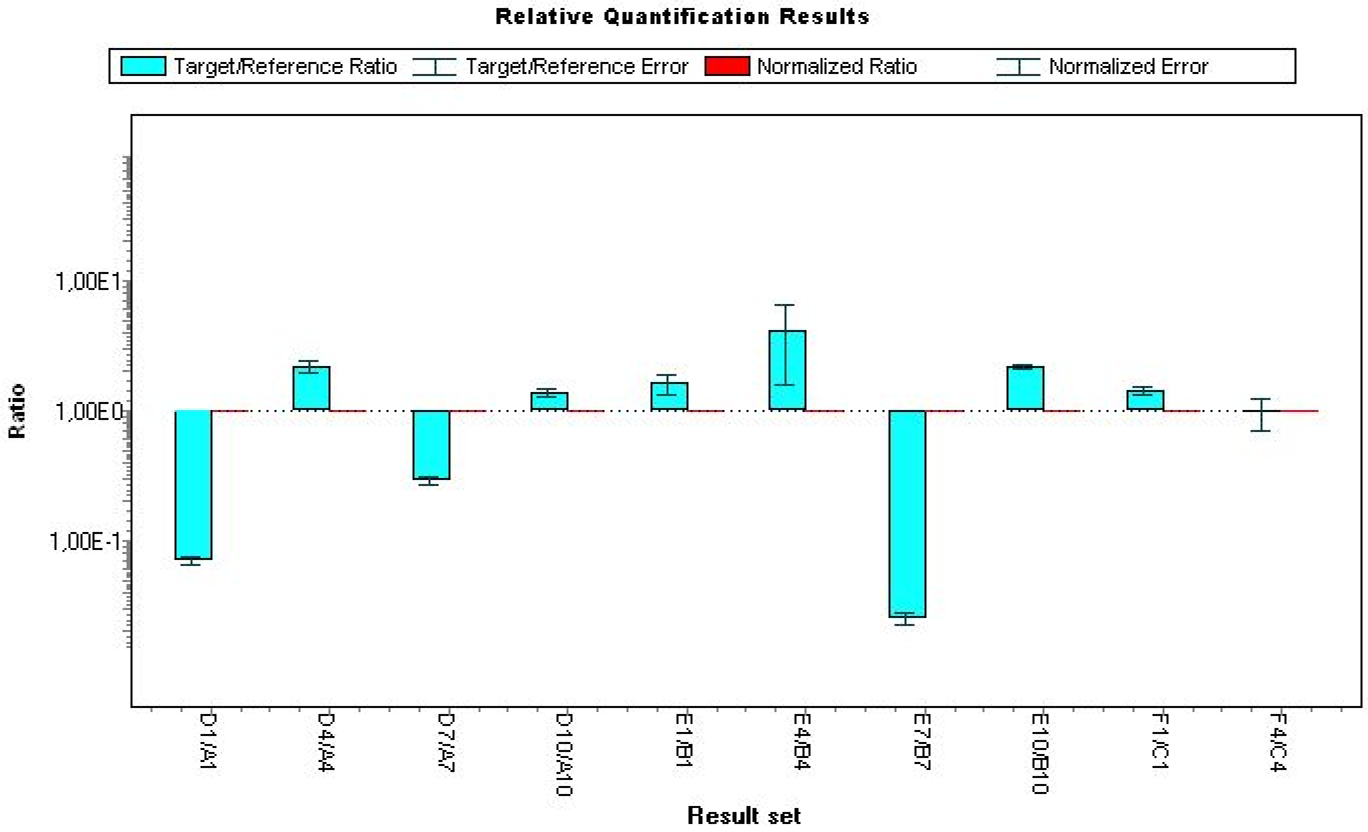
Relative Quantification Results
Target/Reference Ratio
Target/Reference Error
Normalized Ratio
Normalized Error
Ratio
1,00E1
1,00E0
1,00E-1
D1/A1
D4/A4
D7/A7
D10/A10
E1/B1
E4/B4
E7/B7
E10/B10
F1/C1
F4/C4
Result set

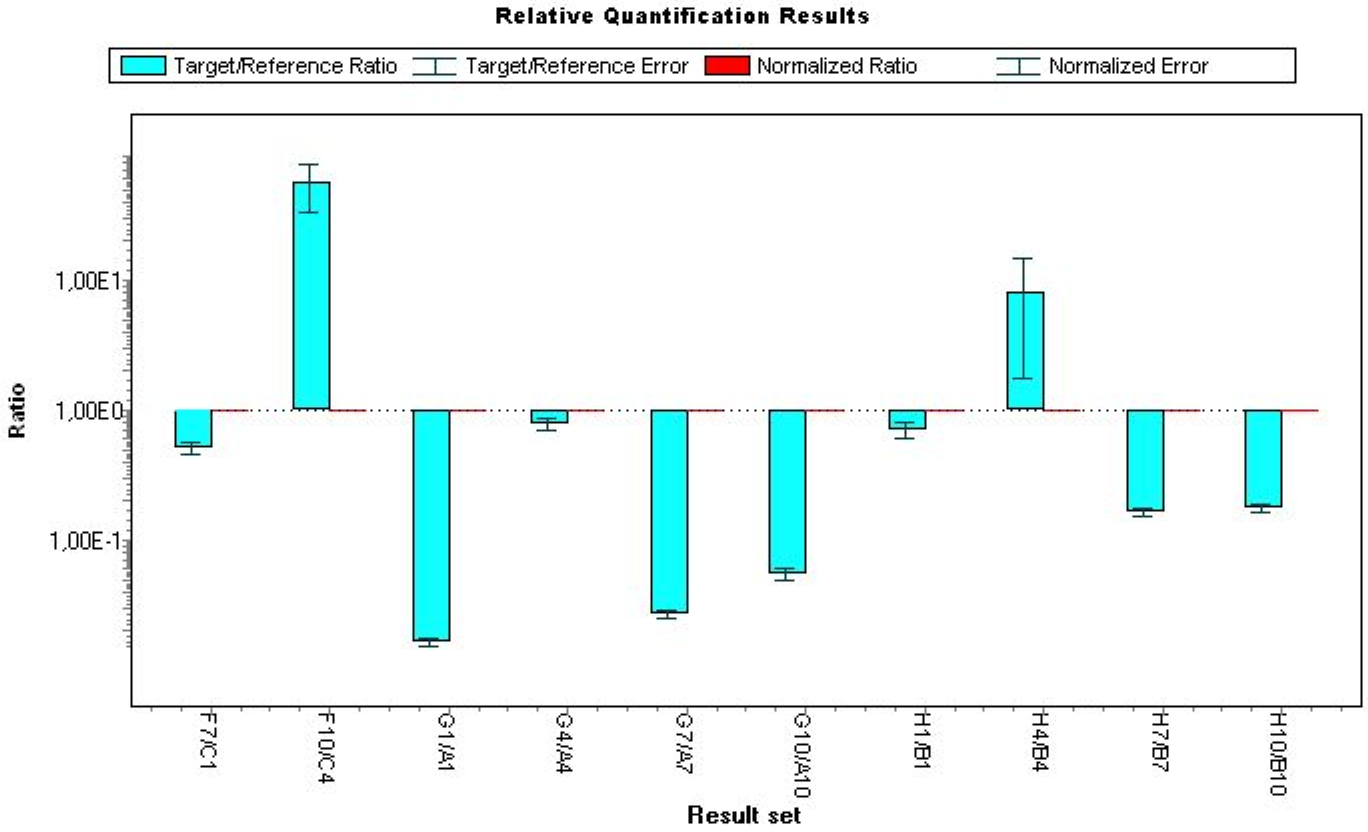
Relative Quantification Results
Target/Reference Ratio
Target/Reference Error
Normalized Ratio
Normalized Error
Ratio
1,00E1
1,00E0
1,00E-1
F7/C1
F10/C4
G1/A1
G4/A4
G7/A7
G10/A10
H1/B1
H4/B4
H7/B7
H10/B10
Result set

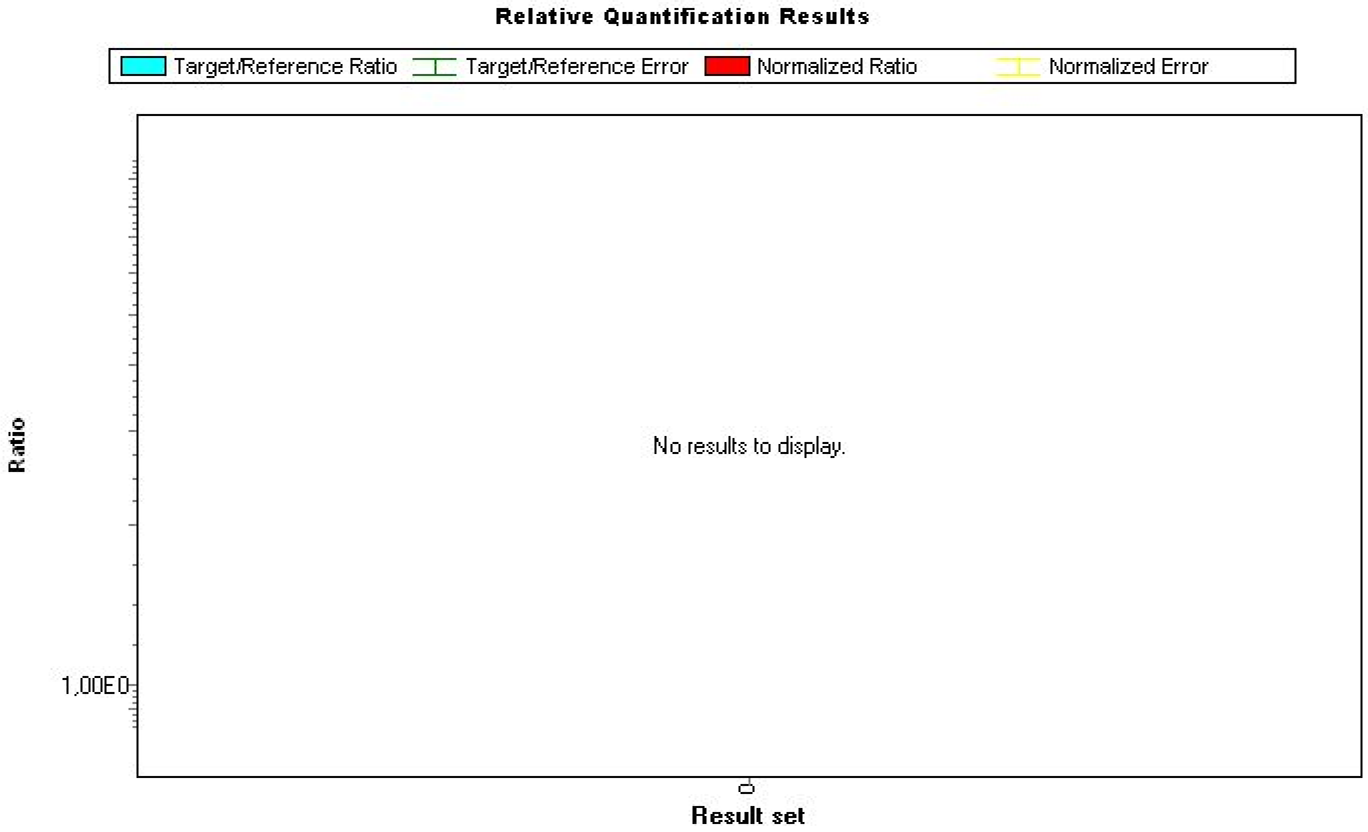

Relative Quantification Results
Target/Reference Ratio
Target/Reference Error
Normalized Ratio
Normalized Error
Ratio
1,00E0
No results to display.
0
Result set
