# Supplementary material for: MicroRNAs and Their Inhibition in Modulating SLC5A8 Expression in the Context of Papillary Thyroid Carcinoma
Source: Int J Mol Sci. 2025 Aug 15;26(16):7889. doi: 10.3390/ijms26167889 (PMC12386254; doi:10.3390/ijms26167889)

LightCycler® 480 Software

Report

Roche

Wojtek_2013-02-28_HPRT AIT NIS_2

Programs

Program Name	pre-incubation		
Cycles	1	Analysis Mode	None

Target (°C)	Acquisition Mode	Hold (hh:mm:ss)	Ramp Rate (°C/s)	Acquisitions (per °C)	Sec Target (°C)	Step size (°C)	Step Delay (cycles)
95	None	00:10:00	4,40		0	0	0

Program Name	amplification		
Cycles	45	Analysis Mode	Quantification

Target (°C)	Acquisition Mode	Hold (hh:mm:ss)	Ramp Rate (°C/s)	Acquisitions (per °C)	Sec Target (°C)	Step size (°C)	Step Delay (cycles)
95	None	00:00:10	4,40		0	0	0
54	None	00:00:20	2,20		0	0	0
72	Single	00:00:15	4,40		0	0	0

Program Name	melting curve		
Cycles	1	Analysis Mode	Melting Curves

Target (°C)	Acquisition Mode	Hold (hh:mm:ss)	Ramp Rate (°C/s)	Acquisitions (per °C)	Sec Target (°C)	Step size (°C)	Step Delay (cycles)
95	None	00:00:05	4,40		0	0	0
65	None	00:01:00	2,20		0	0	0
97	Continuous		0,11	5	0	0	0

Program Name	cooling		
Cycles	1	Analysis Mode	None

Target (°C)	Acquisition Mode	Hold (hh:mm:ss)	Ramp Rate (°C/s)	Acquisitions (per °C)	Sec Target (°C)	Step size (°C)	Step Delay (cycles)
40	None	00:00:30	2,20		0	0	0

Melt Curve Genotyping for All Samples (Melt Curve Genotyping)

Results

Inc	Pos.	Sample Name	Sample Type	Group	Score	Resolution	Status
☑	A1	1584T	Unknown	1	0,94	0,93	
☑	A2	1584T	Unknown	1	0,97	0,95	
☑	A3	1584T	Unknown	1	0,96	0,94	
☑	A4	1584N	Unknown	1	0,98	0,95	
☑	A5	1584N	Unknown	1	0,98	0,95	
☑	A6	1584N	Unknown	1	0,98	0,96	

Results

Inc	Pos.	Sample Name	Sample Type	Group	Score	Resolution	Status
☑	A7	1697T	Unknown	1	0,97	0,95	
☑	A8	1697T	Unknown	1	0,97	0,95	
☑	A9	1697T	Unknown	1	0,96	0,95	
☑	A10	1697N	Unknown	1	0,95	0,94	
☑	A11	1697N	Unknown	1	0,88	0,88	
☑	A12	1697N	Unknown	1	0,95	0,94	
☑	B1	1700T	Unknown	1	0,98	0,96	
☑	B2	1700T	Unknown	1	0,98	0,96	
☑	B3	1700T	Unknown	1	0,98	0,95	
☑	B4	1700N	Unknown	1	0,97	0,93	
☑	B5	1700N	Unknown	1	0,97	0,92	
☑	B6	1700N	Unknown	1	0,97	0,93	
☑	B7	1707T	Unknown	1	0,85	0,77	
☑	B8	1707T	Unknown	1	0,79	0,67	
☑	B9	1707T	Unknown	1	0,79	0,67	
☑	B10	1707N	Unknown	1	0,96	0,94	
☑	B11	1707N	Unknown	1	0,96	0,94	
☑	B12	1707N	Unknown	1	0,94	0,93	
☑	C1	1711T	Unknown	1	0,98	0,96	
☑	C2	1711T	Unknown	1	0,97	0,95	
☑	C3	1711T	Unknown	1	0,97	0,96	
☑	C4	1711N	Unknown	1	0,98	0,96	
☑	C5	1711N	Unknown	1	0,98	0,96	
☑	C6	1711N	Unknown	1	0,98	0,96	
☑	C7	H2O	Unknown	3	1,00	0,50	
☑	C8	H2O	Unknown	4	1,00	0,50	
☑	C9	H2O	Unknown	Unknown	0,49		
☑	C10	H2O	Unknown	5	1,00	0,98	
☑	C11	H20	Unknown	2	0,78	0,74	
☑	C12	H20	Unknown	6	1,00	0,99	
☑	D1	1584T	Unknown	1	0,96	0,94	
☑	D2	1584T	Unknown	1	0,87	0,82	
☑	D3	1584T	Unknown	1	0,94	0,91	
☑	D4	1584N	Unknown	1	0,90	0,86	
☑	D5	1584N	Unknown	1	0,85	0,81	
☑	D6	1584N	Unknown	1	0,92	0,88	
☑	D7	1697T	Unknown	1	0,87	0,81	
☑	D8	1697T	Unknown	1	0,78	0,69	

Results

Inc	Pos.	Sample Name	Sample Type	Group	Score	Resolution	Status
☑	D9	1697T	Unknown	1	0,89	0,83	
☑	D10	1697N	Unknown	1	0,98	0,97	
☑	D11	1697N	Unknown	1	0,97	0,96	
☑	D12	1697N	Unknown	1	0,98	0,97	
☑	E1	1700T	Unknown	Unknown	0,59	0,21	
☑	E2	1700T	Unknown	Unknown	0,58	0,16	
☑	E3	1700T	Unknown	Unknown	0,45	0,03	
☑	E4	1700N	Unknown	1	0,73	0,56	
☑	E5	1700N	Unknown	1	0,70	0,52	
☑	E6	1700N	Unknown	1	0,75	0,61	
☑	E7	1707T	Unknown	1	0,73	0,49	
☑	E8	1707T	Unknown	1	0,80	0,62	
☑	E9	1707T	Unknown	1	0,75	0,54	
☑	E10	1707N	Unknown	1	0,99	0,98	
☑	E11	1707N	Unknown	1	1,00	0,98	
☑	E12	1707N	Unknown	1	0,99	0,98	
☑	F1	1711T	Unknown	1	0,70	0,38	
☑	F2	1711T	Unknown	Unknown	0,65	0,34	
☑	F3	1711T	Unknown	Unknown	0,59	0,13	
☑	F4	1711N	Unknown	1	0,89	0,84	
☑	F5	1711N	Unknown	1	0,80	0,73	
☑	F6	1711N	Unknown	1	0,87	0,81	
☑	F7	1711T	Unknown	2	0,98	0,97	
☑	F8	1711T	Unknown	2	0,98	0,96	
☑	F9	1711T	Unknown	2	0,98	0,96	
☑	F10	1711N	Unknown	2	0,97	0,96	
☑	F11	1711N	Unknown	2	0,93	0,92	
☑	F12	1711N	Unknown	2	0,80	0,80	
☑	G1	1584T	Unknown	2	0,94	0,92	
☑	G2	1584T	Unknown	2	0,94	0,91	
☑	G3	1584T	Unknown	2	0,95	0,93	
☑	G4	1584N	Unknown	2	0,99	0,97	
☑	G5	1584N	Unknown	2	0,95	0,92	
☑	G6	1584N	Unknown	2	0,96	0,94	
☑	G7	1697T	Unknown	2	0,99	0,98	
☑	G8	1697T	Unknown	2	1,00	0,98	
☑	G9	1697T	Unknown	2	0,99	0,98	
☑	G10	1697N	Unknown	2	0,95	0,94	

Results

Inc	Pos.	Sample Name	Sample Type	Group	Score	Resolution	Status
☑	G11	1697N	Unknown	2	0,91	0,90	
☑	G12	1697N	Unknown	2	0,93	0,93	
☑	H1	1700T	Unknown	2	0,94	0,94	
☑	H2	1700T	Unknown	2	0,94	0,93	
☑	H3	1700T	Unknown	2	0,98	0,97	
☑	H4	1700N	Unknown	2	0,99	0,97	
☑	H5	1700N	Unknown	2	0,98	0,96	
☑	H6	1700N	Unknown	2	0,95	0,94	
☑	H7	1707T	Unknown	2	0,97	0,93	
☑	H8	1707T	Unknown	2	0,99	0,95	
☑	H9	1707T	Unknown	2	0,97	0,93	
☑	H10	1707N	Unknown	2	0,98	0,94	
☑	H11	1707N	Unknown	2	0,87	0,82	
☑	H12	1707N	Unknown	2	0,89	0,85	

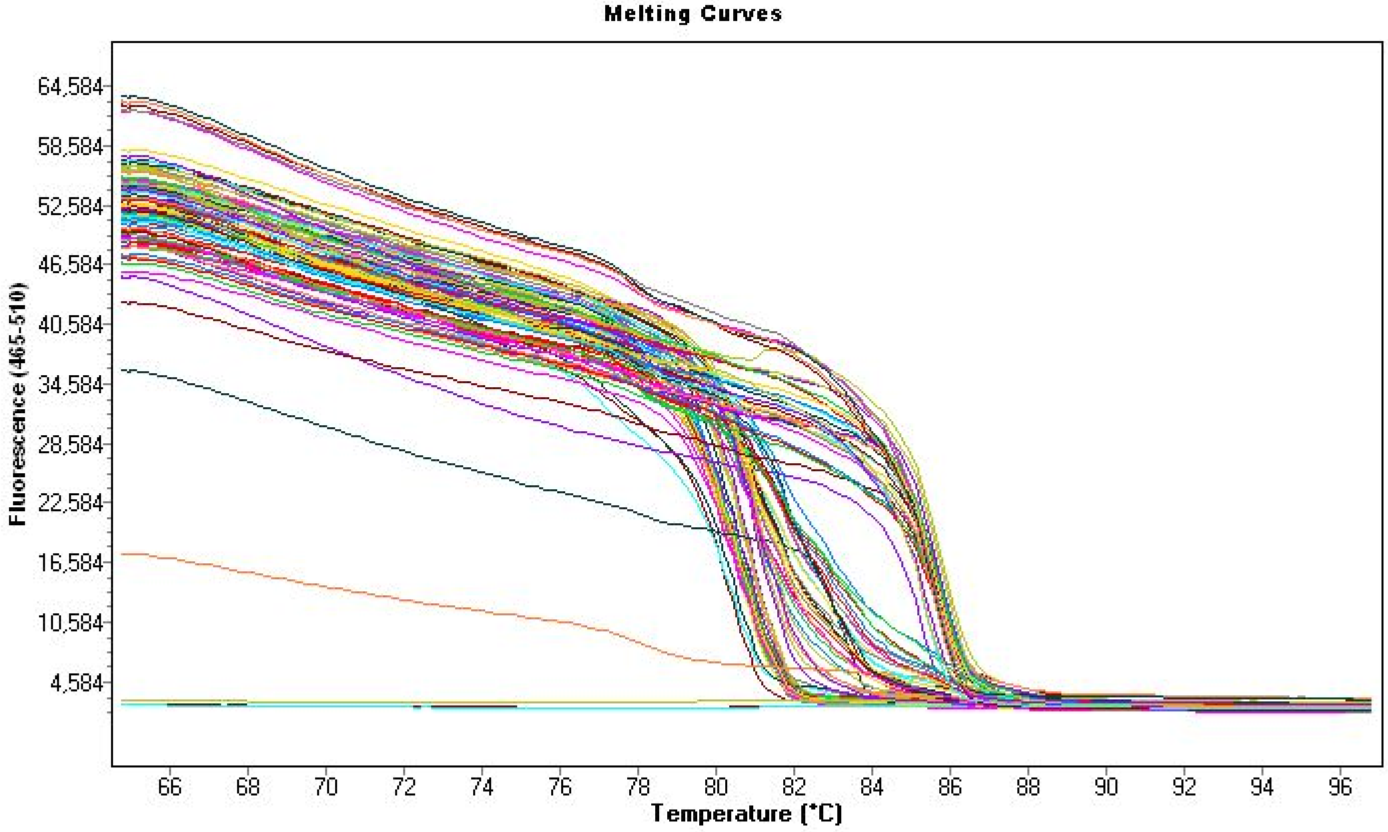

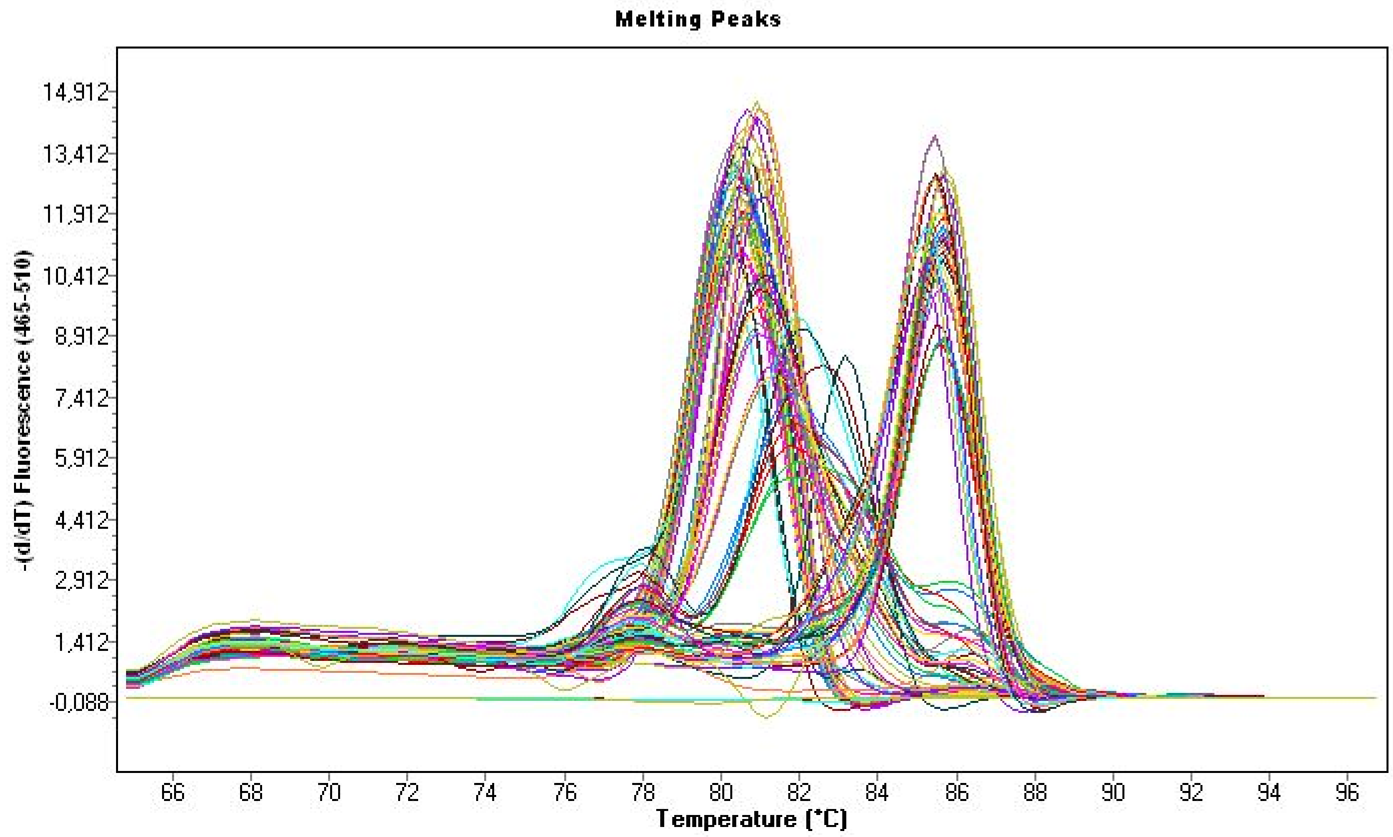

Abs Quant/2nd Derivative Max for All Samples (Abs Quant/2nd Derivative Max)

Statistics

Samples	Mean Cp	Std Cp	Mean conc	Std conc
A1, A2, A3	28,67	0,08		
A4, A5, A6	30,01	0,17		
A7, A8, A9	28,02	0,07		
A10, A11, A12	27,21	0,05		
B1, B2, B3	27,04	0,06		
B4, B5, B6	28,83	0,08		
B7, B8, B9	32,98	0,15		
B10, B11, B12	28,24	0,07		
C1, C2, C3	26,81	0,08		
C4, C5, C6	27,73	0,04		
C7, C8				
C10, C9	40,00			
C11, C12	40,00			
D1, D2, D3	27,00	0,05		
D4, D5, D6	27,16	0,07		
D7, D8, D9	29,57	0,11		
D10, D11, D12	25,28	0,01		

Statistics

Samples	Mean Cp	Std Cp	Mean conc	Std conc
E1, E2, E3	28,62	0,21		
E4, E5, E6	27,29	0,17		
E7, E8, E9	31,93	0,27		
E10, E11, E12	27,16	0,03		
F1, F2, F3	30,26	0,30		
F4, F5, F6	26,96	0,04		
F7, F8, F9	26,06	0,31		
F10, F11, F12	24,61	0,03		
G1, G2, G3	25,30	0,23		
G4, G5, G6	25,30	0,16		
G7, G8, G9	27,07	0,14		
G10, G11, G12	26,71	0,16		
H1, H2, H3	25,37	0,08		
H4, H5, H6	24,90	0,75		
H7, H8, H9	29,10	0,65		
H10, H11, H12	31,31	1,43		

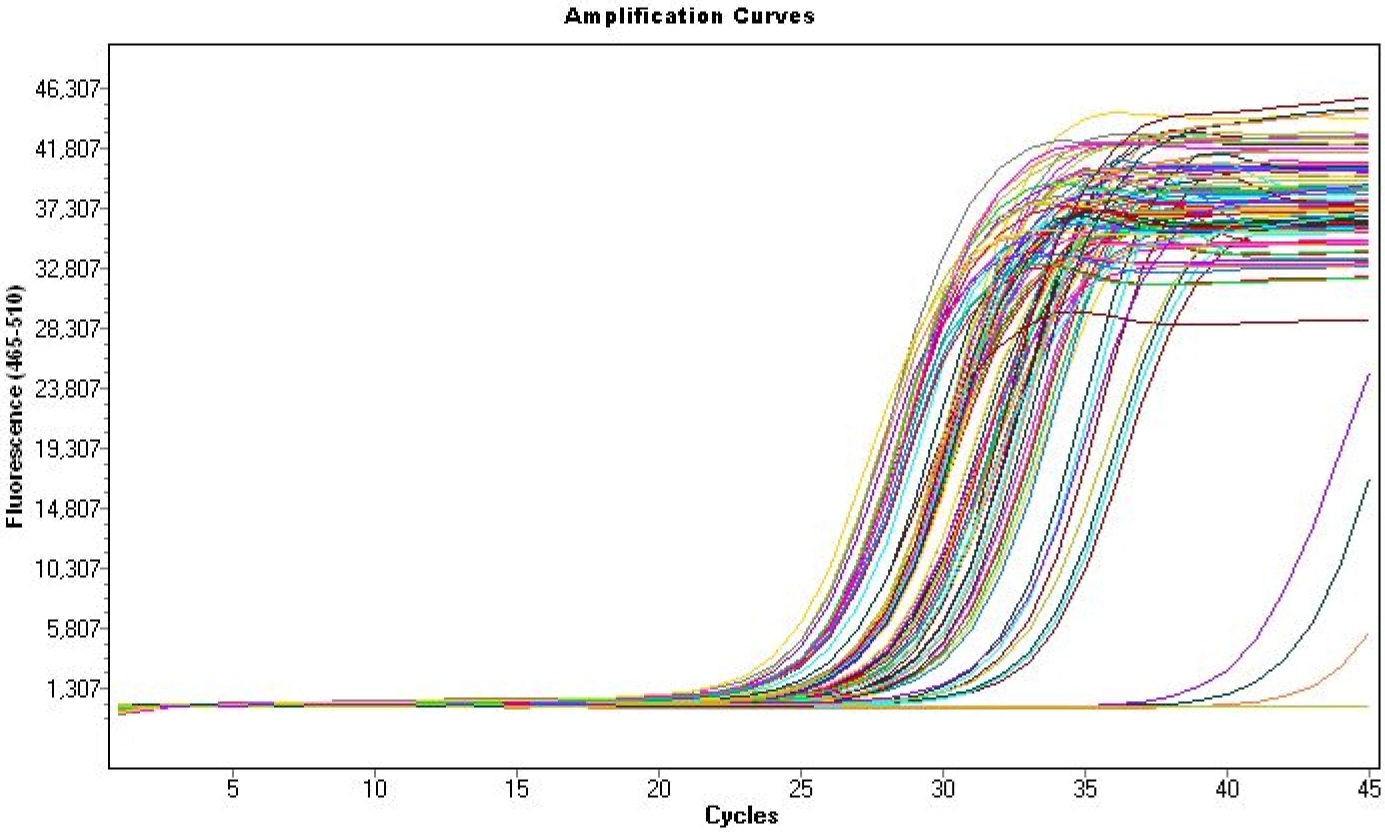

Advanced Relative Quantification for All Samples (Relative Quantification)

Target Names

Target ID	Filter Combination	Standards/Efficiency	Efficiency Value
HPRT	465-510	Efficiency	2,00
AIT	465-510	Efficiency	2,00
NIS	465-510	Efficiency	2,00

Results

Bar Chart	Pairing	Sample Name	Target Name		Tgt Cp	Ref. Cp	Ratios		Corr/Multi Factor	Status
			Targets	References	Mean	Mean	Tgt/Ref.	Norm		
☑	D1/A1	1584T	AIT	HPRT	27,00	28,67	3,166		1/1	
☑	D4/A4	1584N	AIT	HPRT	27,16	30,01	7,212		1/1	
☑	D7/A7	1697T	AIT	HPRT	29,57	28,02	0,3418		1/1	
☑	D10/A10	1697N	AIT	HPRT	25,28	27,21	3,804		1/1	
☑	E1/B1	1700T	AIT	HPRT	28,62	27,04	0,3342		1/1	
☑	E4/B4	1700N	AIT	HPRT	27,29	28,83	2,915		1/1	
☑	E7/B7	1707T	AIT	HPRT	31,93	32,98	2,071		1/1	
☑	E10/B10	1707N	AIT	HPRT	27,16	28,24	2,124		1/1	
☑	F1/C1	1711T	AIT	HPRT	30,26	26,81	9,12E-2		1/1	
☑	F4/C4	1711N	AIT	HPRT	26,96	27,73	1,697		1/1	
☑	F7/C1	1711T	NIS	HPRT	26,06	26,81	1,683		1/1	
☑	F10/C4	1711N	NIS	HPRT	24,61	27,73	8,662		1/1	
☑	G1/A1	1584T	NIS	HPRT	25,30	28,67	10,31		1/1	
☑	G4/A4	1584N	NIS	HPRT	25,30	30,01	26,29		1/1	
☑	G7/A7	1697T	NIS	HPRT	27,07	28,02	1,941		1/1	
☑	G10/A10	1697N	NIS	HPRT	26,71	27,21	1,409		1/1	
☑	H1/B1	1700T	NIS	HPRT	25,37	27,04	3,196		1/1	
☑	H4/B4	1700N	NIS	HPRT	24,90	28,83	15,20		1/1	
☑	H7/B7	1707T	NIS	HPRT	29,10	32,98	14,68		1/1	
☑	H10/B10	1707N	NIS	HPRT	31,31	28,24	0,1195		1/1	

Relative Quantification Results

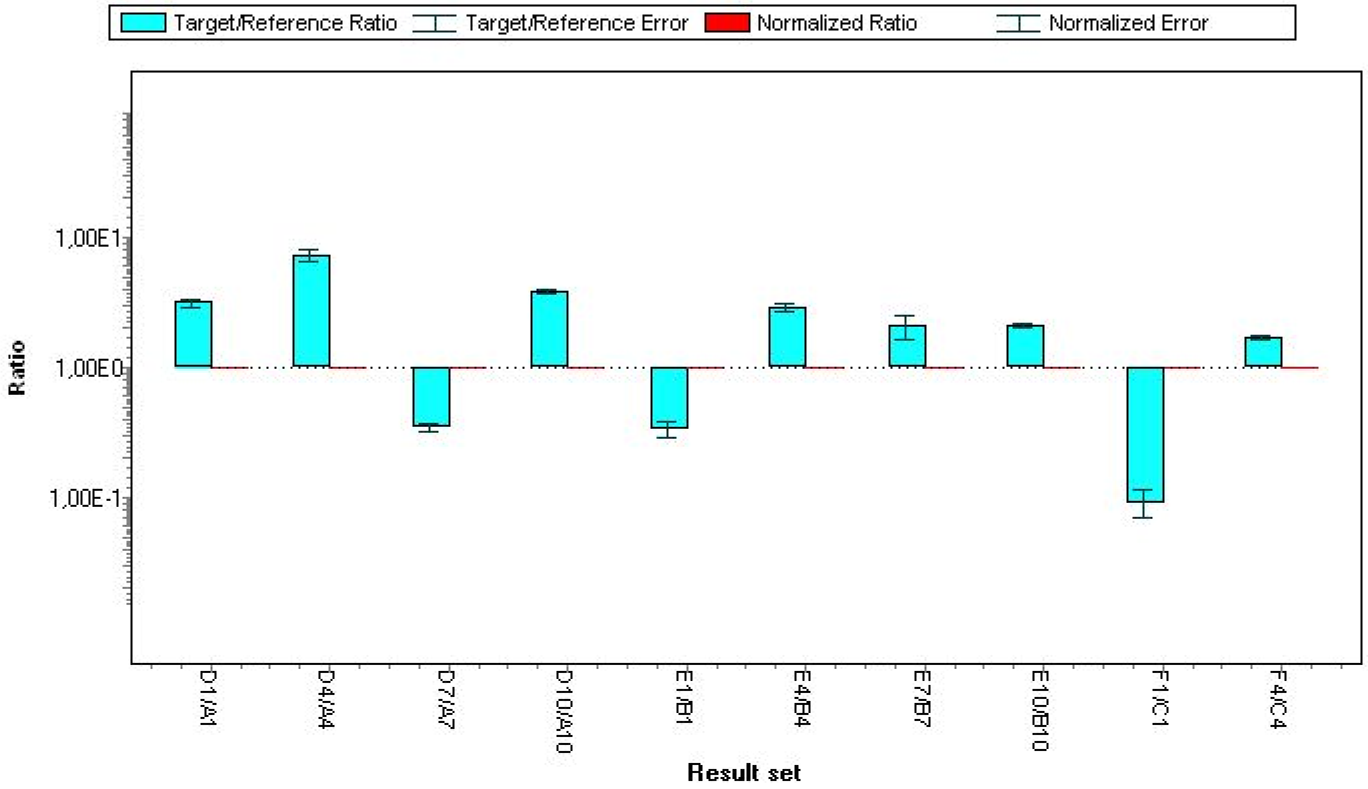

Relative Quantification Results

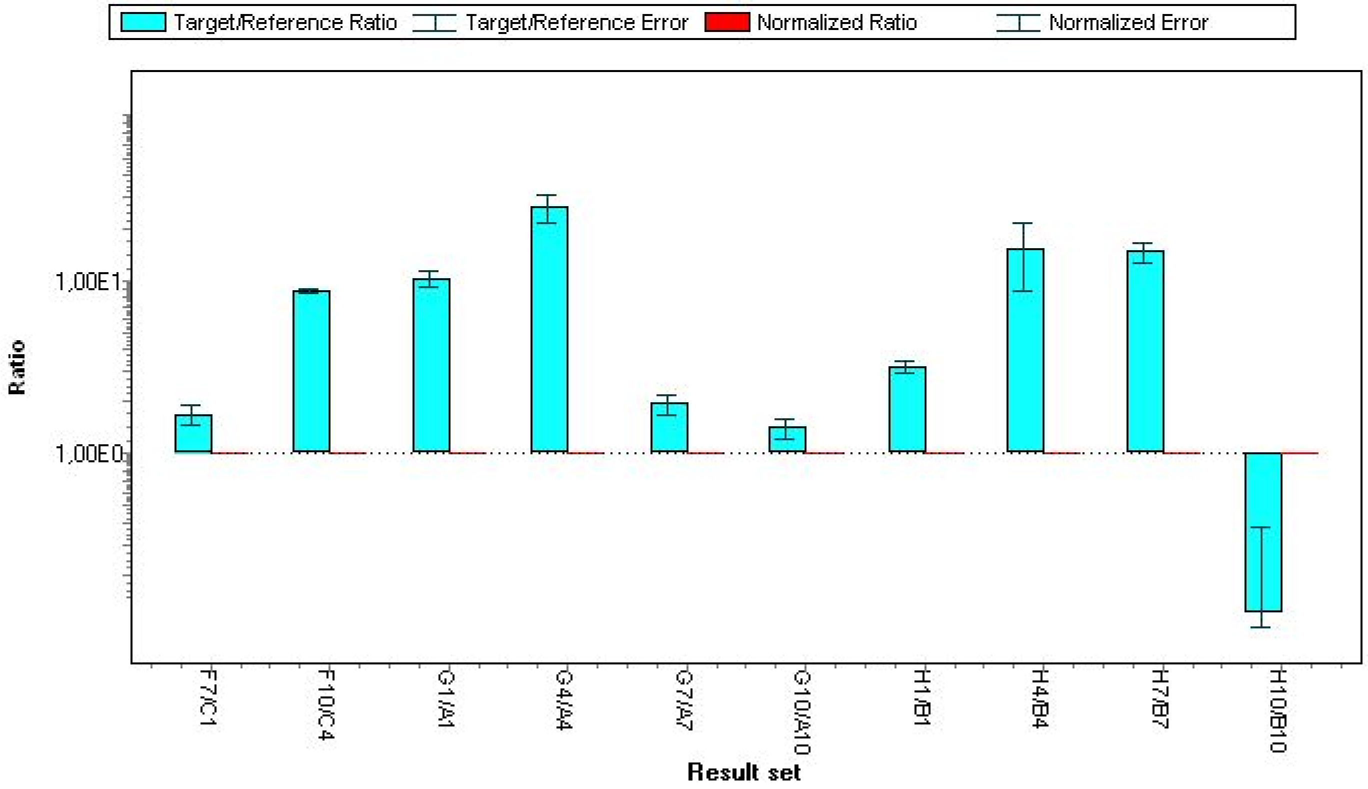

Relative Quantification Results

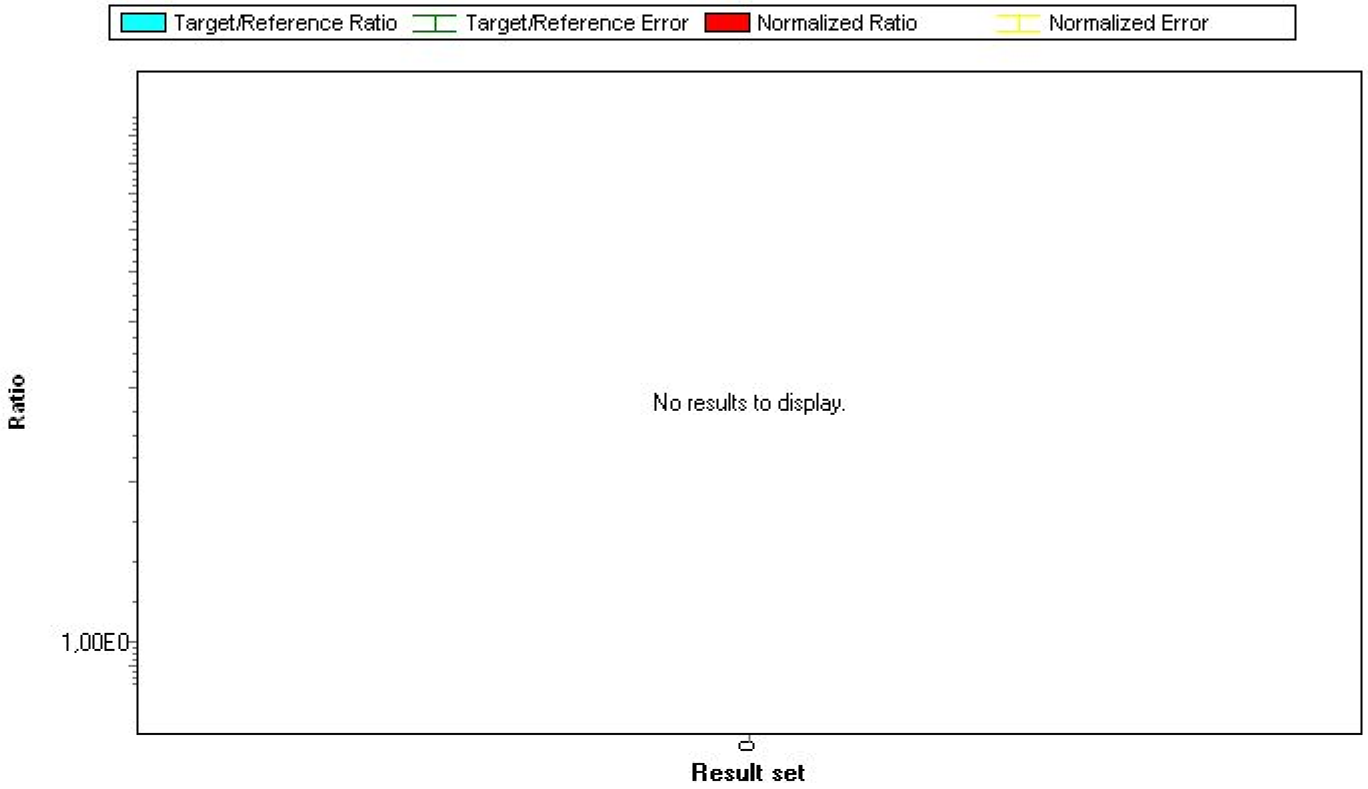

Supplement: Supplementary file 1 [file ijms-26-07889-s001.zip › ijms-3558049-supplementary/Manuscript data/Fig1 data/Data/2013-02-28 HPRT AIT NIS 1584-1711 (2).PDF]
