# Supplementary material for: MicroRNAs and Their Inhibition in Modulating SLC5A8 Expression in the Context of Papillary Thyroid Carcinoma
Source: Int J Mol Sci. 2025 Aug 15;26(16):7889. doi: 10.3390/ijms26167889 (PMC12386254; doi:10.3390/ijms26167889)
Supplement: Supplementary file 1 [file ijms-26-07889-s001.zip › ijms-3558049-supplementary/Manuscript data/Fig1 data/Data/2013-03-04 HPRT AIT NIS.PDF]

Roche

Abs Quant/2nd Derivative Max for All Samples (Abs Quant/2nd Derivative Max)

Results

Inc	Pos	Name	Type	CP	Concentration	Standard	Status
☑	A1	1719T	Unknown	27,47			
☑	A2	1719T	Unknown	27,72			
☑	A3	1719T	Unknown	27,52			
☑	A4	1719N	Unknown	28,54			
☑	A5	1719N	Unknown	28,65			
☑	A6	1719N	Unknown	28,57			
☑	A7	1521T	Unknown	24,75			
☑	A8	1521T	Unknown	24,66			
☑	A9	1521T	Unknown	24,70			
☑	A10	1521N	Unknown	27,22			
☑	A11	1521N	Unknown	27,06			
☑	A12	1521N	Unknown	27,07			
☑	B1	1707T	Unknown	34,85			
☑	B2	1707T	Unknown	34,02			
☑	B3	1707T	Unknown	34,87			
☑	B4	1707N	Unknown	27,55			
☑	B5	1707N	Unknown	27,65			
☑	B6	1707N	Unknown	27,76			
☑	B7	1596T	Unknown	29,51			
☑	B8	1596T	Unknown	29,23			
☑	B9	1596T	Unknown	28,90			
☑	B10	1596N	Unknown	32,89			
☑	B11	1596N	Unknown	33,32			
☑	B12	1596N	Unknown	33,02			
☑	C1	1719T	Unknown	29,56			
☑	C2	1719T	Unknown	29,57			
☑	C3	1719T	Unknown	29,76			
☑	C4	1719N	Unknown	27,23			
☑	C5	1719N	Unknown	27,34			
☑	C6	1719N	Unknown	27,22			
☑	C7	1560T	Unknown	31,62			
☑	C8	1560T	Unknown	31,19			
☑	C9	1560T	Unknown	31,17			

Results

Inc	Pos	Name	Type	CP	Concentration	Standard	Status
☑	C10	1560N	Unknown	27,09			
☑	C11	1560N	Unknown	27,24			
☑	C12	1560N	Unknown	27,27			
☑	D1	1674T	Unknown	26,00			
☑	D2	1674T	Unknown	26,03			
☑	D3	1674T	Unknown	25,96			
☑	D4	1674N	Unknown	36,43			
☑	D5	1674N	Unknown	36,00			
☑	D6	1674N	Unknown	35,42			
☑	D7	1697T	Unknown	29,81			
☑	D8	1697T	Unknown	29,75			
☑	D9	1697T	Unknown	30,06			
☑	D10	1697N	Unknown	25,21			
☑	D11	1697N	Unknown	25,18			
☑	D12	1697N	Unknown	25,51			
☑	E1	1700T	Unknown	31,21			
☑	E2	1700T	Unknown	31,34			
☑	E3	1700T	Unknown	31,84			
☑	E4	1700N	Unknown	28,63			
☑	E5	1700N	Unknown	28,61			
☑	E6	1700N	Unknown	28,77			
☑	E7	1707T	Unknown	36,98			
☑	E8	1707T	Unknown				
☑	E9	1707T	Unknown				
☑	E10	1707N	Unknown	29,10			
☑	E11	1707N	Unknown				
☑	E12	1707N	Unknown				
☑	F1	1711T	Unknown	33,04			
☑	F2	1711T	Unknown	33,31			
☑	F3	1711T	Unknown	32,68			
☑	F4	1711N	Unknown	27,43			
☑	F5	1711N	Unknown	27,69			
☑	F6	1711N	Unknown	27,44			
☑	F7	1719T	Unknown	29,44			
☑	F8	1719T	Unknown	29,15			
☑	F9	1719T	Unknown	29,26			
☑	F10	1719N	Unknown	28,82			

Results

Inc	Pos	Name	Type	CP	Concentration	Standard	Status
☑	F11	1719N	Unknown	28,78			
☑	F12	1719N	Unknown	28,89			
☑	G1	1541T	Unknown	28,90			
☑	G2	1541T	Unknown	29,03			
☑	G3	1541T	Unknown	29,07			
☑	G4	1541N	Unknown	33,92			
☑	G5	1541N	Unknown	34,30			
☑	G6	1541N	Unknown	33,77			
☑	G7	1557T	Unknown	31,61			
☑	G8	1557T	Unknown	31,29			
☑	G9	1557T	Unknown	31,56			
☑	G10	1557N	Unknown	31,28			
☑	G11	1557N	Unknown	30,76			
☑	G12	1557N	Unknown	30,83			
☑	H1	1700T	Unknown	27,42			
☑	H2	1700T	Unknown	27,08			
☑	H3	1700T	Unknown	27,29			
☑	H4	1700N	Unknown	26,17			
☑	H5	1700N	Unknown	26,25			
☑	H6	1700N	Unknown	26,30			
☑	H7	H20	Negative Control				
☑	H8	H20	Negative Control				
☑	H9	H20	Negative Control				
☑	H10	H20	Negative Control				
☑	H11	H20	Negative Control				
☑	H12	H20	Negative Control				
