# Supplementary material for: MicroRNAs and Their Inhibition in Modulating SLC5A8 Expression in the Context of Papillary Thyroid Carcinoma
Source: Int J Mol Sci. 2025 Aug 15;26(16):7889. doi: 10.3390/ijms26167889 (PMC12386254; doi:10.3390/ijms26167889)
Supplement: Supplementary file 1 [file ijms-26-07889-s001.zip › ijms-3558049-supplementary/Manuscript data/Fig1 data/Data/2013-03-11 HPRT AIT NIS Cp.PDF]

Roche

Abs Quant/2nd Derivative Max for All Samples (Abs Quant/2nd Derivative Max)

Results

Inc	Pos	Name	Type	CP	Concentration	Standard	Status
☑	A1	1507T	Unknown	26,49			
☑	A2	1507N	Unknown	26,94			
☑	A3	1531T	Unknown	24,61			
☑	A4	1531N	Unknown	26,54			
☑	A5	1556T	Unknown	26,59			
☑	A6	1556N	Unknown	26,02			
☑	A7	1507T	Unknown	32,68			
☑	A8	1507N	Unknown	31,48			
☑	A9	1531T	Unknown	29,43			
☑	A10	1531N	Unknown	31,84			
☑	A11	1556T	Unknown	32,78			
☑	A12	1556N	Unknown	27,92			
☑	B1	1507T	Unknown	26,16			
☑	B2	1507N	Unknown	26,98			
☑	B3	1531T	Unknown	25,27			
☑	B4	1531N	Unknown	26,33			
☑	B5	1556T	Unknown	25,97			
☑	B6	1556N	Unknown	26,05			
☑	B7	1507T	Unknown	32,89			
☑	B8	1507N	Unknown	31,20			
☑	B9	1531T	Unknown	29,06			
☑	B10	1531N	Unknown	32,04			
☑	B11	1556T	Unknown	32,47			
☑	B12	1556N	Unknown	27,33			
☑	C1	1507T	Unknown	25,29			
☑	C2	1507N	Unknown	26,60			
☑	C3	1531T	Unknown	23,70			
☑	C4	1531N	Unknown	25,93			
☑	C5	1556T	Unknown	24,78			
☑	C6	1556N	Unknown	25,84			
☑	C7	1507T	Unknown	32,02			
☑	C8	1507N	Unknown	29,95			

> - Late Cp call (last five cycles) has higher uncertainty

Results

Inc	Pos	Name	Type	CP	Concentration	Standard	Status
☑	C9	1531T	Unknown	28,85			
☑	C10	1531N	Unknown	31,74			
☑	C11	1556T	Unknown	32,43			
☑	C12	1556N	Unknown	26,95			
☑	D1	1560T	Unknown	25,96			
☑	D2	1560T	Unknown	25,76			
☑	D3	1560T	Unknown	24,95			
☑	D4	1707T	Unknown	35,67			
☑	D5	1707T	Unknown	35,99			
☑	D6	1707T	Unknown	33,89			
☑	D7	1560T	Unknown	30,68			
☑	D8	1560T	Unknown	30,98			
☑	D9	1560T	Unknown	30,93			
☑	D10	1707T	Unknown	35,65			
☑	D11	1707T	Unknown	35,26			
☑	D12	1707T	Unknown	36,24			
☑	E1	1560N	Unknown	26,97			
☑	E2	1560N	Unknown	26,62			
☑	E3	1560N	Unknown	26,66			
☑	E4	1707N	Unknown	26,85			
☑	E5	1707N	Unknown	26,98			
☑	E6	1707N	Unknown	26,44			
☑	E7	1560N	Unknown	26,62			
☑	E8	1560N	Unknown	26,81			
☑	E9	1560N	Unknown	26,95			
☑	E10	1707N	Unknown	26,66			
☑	E11	1707N	Unknown	27,30			
☑	E12	1707N	Unknown	27,45			
☑	F1	1674T	Unknown	26,05			
☑	F2	1674T	Unknown	26,14			
☑	F3	1674T	Unknown	25,57			
☑	F4	1711T	Unknown	25,61			
☑	F5	1711T	Unknown	25,71			
☑	F6	1711T	Unknown	25,49			
☑	F7	1674T	Unknown	25,49			
☑	F8	1674T	Unknown	26,30			
☑	F9	1674T	Unknown	26,24			

> - Late Cp call (last five cycles) has higher uncertainty

Results

Inc	Pos	Name	Type	CP	Concentration	Standard	Status
☑	F10	1711T	Unknown	33,00			
☑	F11	1711T	Unknown	33,17			
☑	F12	1711T	Unknown	33,28			
☑	G1	1674N	Unknown	34,55			
☑	G2	1674N	Unknown	36,91			
☑	G3	1674N	Unknown	34,64			
☑	G4	1711N	Unknown	27,03			
☑	G5	1711N	Unknown	27,49			
☑	G6	1711N	Unknown	26,81			
☑	G7	1674N	Unknown	37,12			
☑	G8	1674N	Unknown	37,59			
☑	G9	1674N	Unknown	35,72			
☑	G10	1711N	Unknown	27,57			
☑	G11	1711N	Unknown	27,22			
☑	G12	1711N	Unknown	28,24			
☑	H1	1700T	Unknown	26,80			
☑	H2	1700T	Unknown	26,81			
☑	H3	1700T	Unknown	26,49			
☑	H4	K-	Negative Control	36,67			
☑	H5	K-	Negative Control				
☑	H6	K-	Negative Control				
☑	H7	1700T	Unknown	31,81			
☑	H8	1700T	Unknown	32,80			
☑	H9	1700T	Unknown	31,82			
☑	H10	RT-	Negative Control	36,77			
☑	H11	RT-	Negative Control				
☑	H12	RT-	Negative Control	42,00			>

> - Late Cp call (last five cycles) has higher uncertainty
