# Supplementary material for: MicroRNAs and Their Inhibition in Modulating SLC5A8 Expression in the Context of Papillary Thyroid Carcinoma
Source: Int J Mol Sci. 2025 Aug 15;26(16):7889. doi: 10.3390/ijms26167889 (PMC12386254; doi:10.3390/ijms26167889)
Supplement: Supplementary file 1 [file ijms-26-07889-s001.zip › ijms-3558049-supplementary/Manuscript data/Fig1 data/Data/2013-03-11 HPRT AIT NIS.PDF]

Wojtek_2013-03-11_HPRT AIT NIS

Programs

Program Name	pre-incubation		
Cycles	1	Analysis Mode	None

Target (°C)	Acquisition Mode	Hold (hh:mm:ss)	Ramp Rate (°C/s)	Acquisitions (per °C)	Sec Target (°C)	Step size (°C)	Step Delay (cycles)
95	None	00:10:00	4,40		0	0	0

Program Name	amplification		
Cycles	55	Analysis Mode	Quantification

Target (°C)	Acquisition Mode	Hold (hh:mm:ss)	Ramp Rate (°C/s)	Acquisitions (per °C)	Sec Target (°C)	Step size (°C)	Step Delay (cycles)
95	None	00:00:15	4,40		0	0	0
57	None	00:00:15	2,20		0	0	0
72	Single	00:00:15	4,40		0	0	0

Program Name	melting curve		
Cycles	1	Analysis Mode	Melting Curves

Target (°C)	Acquisition Mode	Hold (hh:mm:ss)	Ramp Rate (°C/s)	Acquisitions (per °C)	Sec Target (°C)	Step size (°C)	Step Delay (cycles)
95	None	00:00:05	4,40		0	0	0
65	None	00:01:00	2,20		0	0	0
97	Continuous		0,11	5	0	0	0

Program Name	cooling		
Cycles	1	Analysis Mode	None

Target (°C)	Acquisition Mode	Hold (hh:mm:ss)	Ramp Rate (°C/s)	Acquisitions (per °C)	Sec Target (°C)	Step size (°C)	Step Delay (cycles)
40	None	00:00:30	2,20		0	0	0

Abs Quant/2nd Derivative Max for All Samples (Abs Quant/2nd Derivative Max)

Statistics

Samples	Mean Cp	Std Cp	Mean conc	Std conc
A1, B1, C1	25,98	0,62		
A2, B2, C2	26,84	0,21		
A3, B3, C3	24,53	0,79		
A4, B4, C4	26,27	0,31		
A5, B5, C5	25,78	0,92		
A6, B6, C6	25,97	0,11		
A7, B7, C7	32,53	0,46		

Statistics

Samples	Mean Cp	Std Cp	Mean conc	Std conc
A8, B8, C8	30,87	0,81		
A9, B9, C9	29,11	0,29		
A10, B10, C10	31,87	0,15		
A11, B11, C11	32,56	0,19		
A12, B12, C12	27,40	0,49		
D1, D2, D3	25,56	0,53		
D4, D5, D6	35,18	1,13		
D7, D8, D9	30,86	0,16		
D10, D11, D12	35,72	0,50		
E1, E2, E3	26,75	0,19		
E4, E5, E6	26,76	0,28		
E7, E8, E9	26,79	0,17		
E10, E11, E12	27,13	0,42		
F1, F2, F3	25,92	0,31		
F4, F5, F6	25,61	0,11		
F7, F8, F9	26,01	0,45		
F10, F11, F12	33,15	0,14		
G1, G2, G3	35,37	1,34		
G4, G5, G6	27,11	0,35		
G7, G8, G9	36,81	0,98		
G10, G11, G12	27,67	0,52		
H1, H2, H3	26,70	0,18		
H7, H8, H9	32,14	0,57		

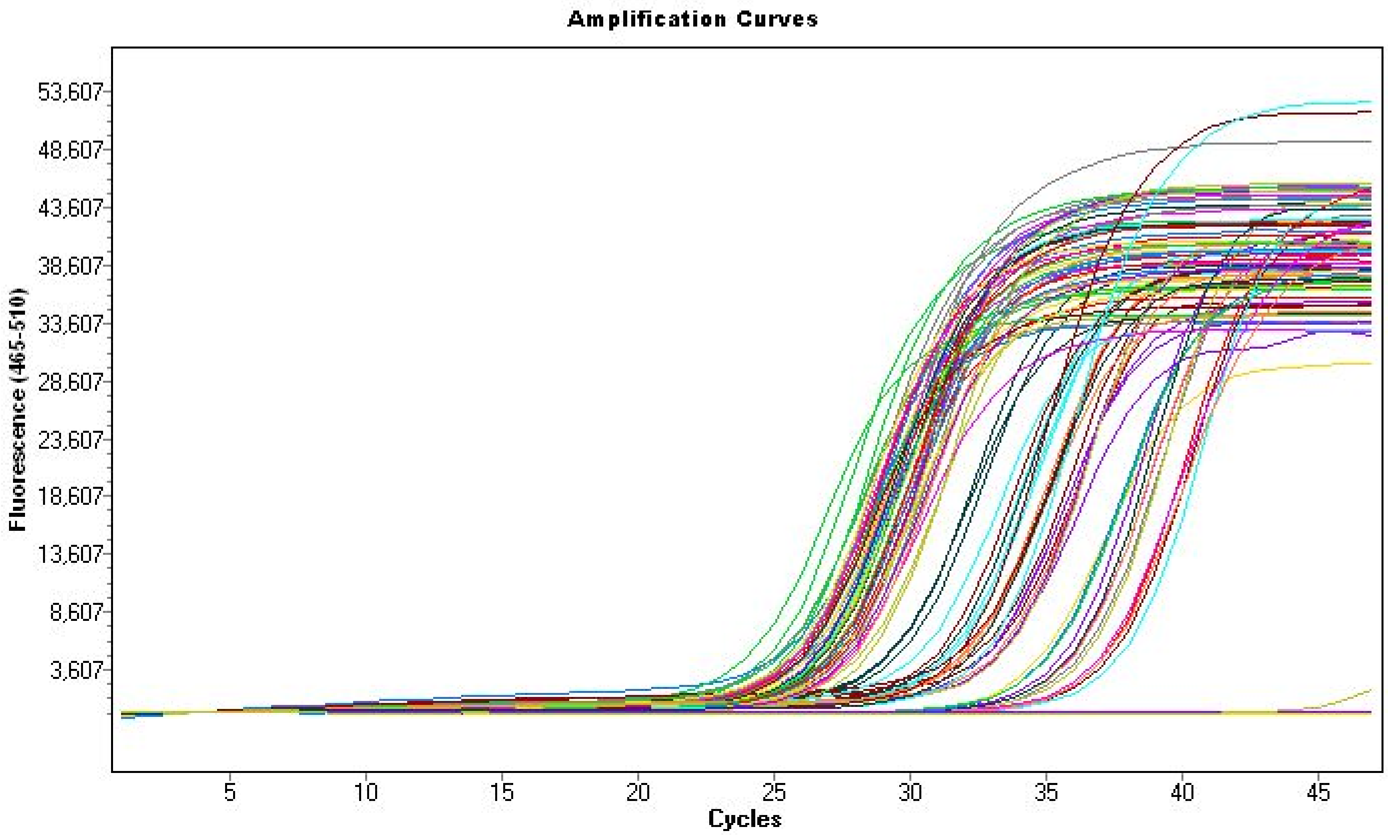

Advanced Relative Quantification for All Samples (Relative Quantification)

Target Names

Target ID	Filter Combination	Standards/Efficiency	Efficiency Value
HPRT	465-510	Efficiency	2,00
NIS	465-510	Efficiency	2,00
AIT	465-510	Efficiency	2,00

Results

Bar			Target Name		Tgt Cp	Ref. Cp	Ratios		Corr/Multi	
Chart	Pairing	Sample Name	Targets	References	Mean	Mean	Tgt/Ref.	Norm	Factor	Status
☑	A7/A1	1507T	NIS	HPRT	32,53	25,98	1,07E-2		1/1	
☑	A8/A2	1507N	NIS	HPRT	30,87	26,84	6,11E-2		1/1	
☑	A9/A3	1531T	NIS	HPRT	29,11	24,53	4,17E-2		1/1	
☑	A10/A4	1531N	NIS	HPRT	31,87	26,27	2,05E-2		1/1	
☑	A11/A5	1556T	NIS	HPRT	32,56	25,78	9,12E-3		1/1	
☑	A12/A6	1556N	NIS	HPRT	27,40	25,97	0,3717		1/1	
☑	D7/D1	1560T	AIT	HPRT	30,86	25,56	2,53E-2		1/1	
☑	D10/D4	1707T	AIT	HPRT	35,72	35,18	0,6913		1/1	
☑	E7/E1	1560N	AIT	HPRT	26,79	26,75	0,9720		1/1	
☑	E10/E4	1707N	AIT	HPRT	27,13	26,76	0,7717		1/1	
☑	F7/F1	1674T	AIT	HPRT	26,01	25,92	0,9393		1/1	
☑	F10/F4	1711T	AIT	HPRT	33,15	25,61	5,35E-3		1/1	
☑	G7/G1	1674N	AIT	HPRT	36,81	35,37	0,3677		1/1	
☑	G10/G4	1711N	AIT	HPRT	27,67	27,11	0,6761		1/1	

☑	H7/H1	1700T	AIT	HPRT	32,14	26,70	2,30E-2		1/1

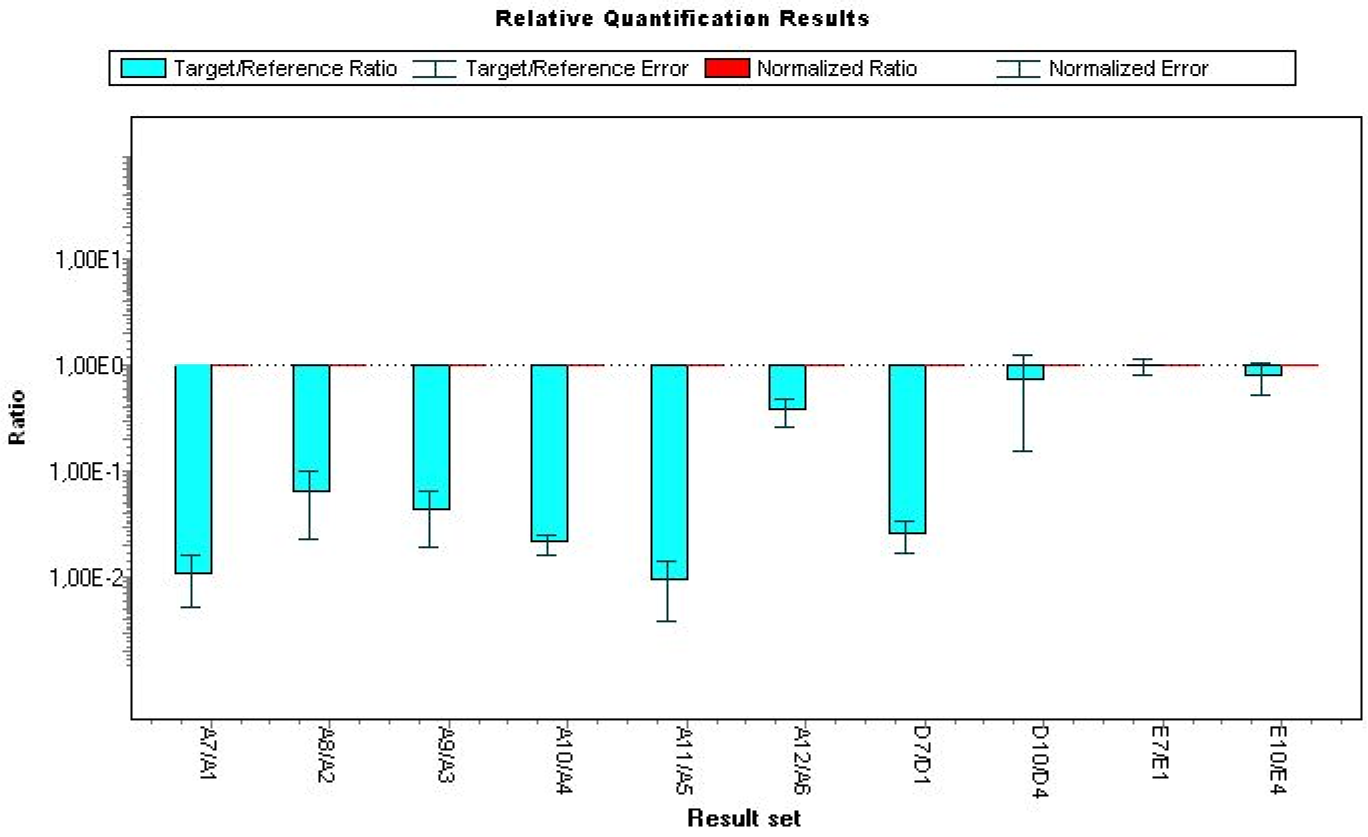

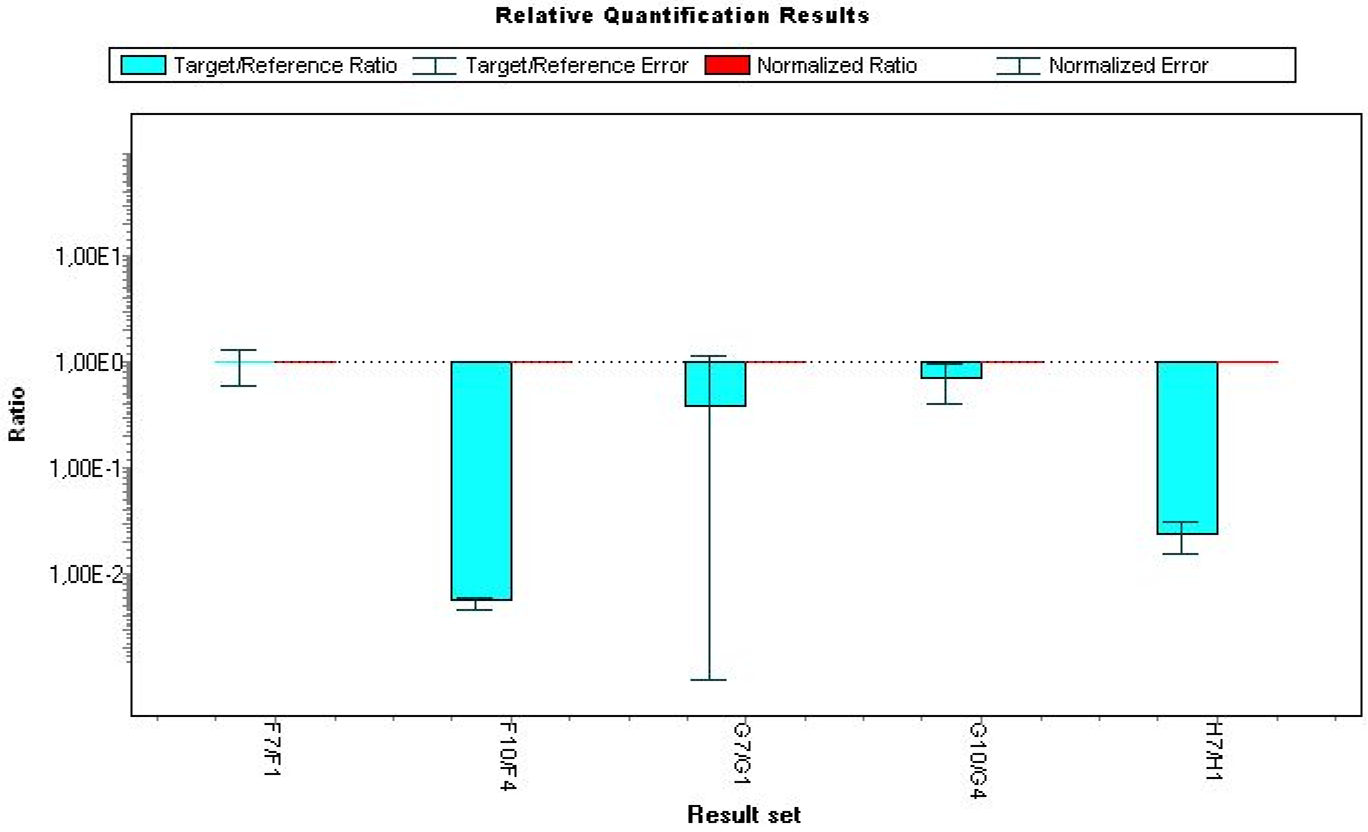

Tm Calling for All Samples (Tm Calling)

Results

			Peak 1				Peak 2				
Inc	Pos	Sample Name	Tm	Area	Width	Height	Tm	Area	Width	Height	Status
☑	A1	1507T	80,28	43,68	2,65	16,51					
☑	A2	1507N	80,54	36,92	2,69	13,72					
☑	A3	1531T	80,58	44,38	2,62	16,93					
☑	A4	1531N	80,52	50,73	2,68	18,93					
☑	A5	1556T	80,12	48,64	2,63	18,47					
☑	A6	1556N	80,51	41,23	2,65	15,54					
☑	A7	1507T	85,39	37,73	4,27	8,84					
☑	A8	1507N	85,48	42,29	4,54	9,31					
☑	A9	1531T	85,42	35,77	3,45	10,38					
☑	A10	1531N	85,32	24,06	2,78	8,67	87,66	11,53	1,69	6,81	
☑	A11	1556T	85,34	31,38	2,54	12,35					
☑	A12	1556N	85,69	40,57	4,17	9,74					
☑	B1	1507T	80,46	39,04	2,68	14,59					
☑	B2	1507N	80,56	36,79	2,68	13,72					
☑	B3	1531T	80,24	43,29	2,69	16,10					
☑	B4	1531N	80,57	42,19	2,76	15,29					
☑	B5	1556T	80,49	42,25	2,71	15,59					
☑	B6	1556N	80,40	37,68	2,57	14,67					
☑	B7	1507T	85,83	34,80	4,29	8,11					
☑	B8	1507N	85,71	39,30	4,47	8,78					
☑	B9	1531T	85,51	37,00	3,46	10,70					
☑	B10	1531N	86,19	37,93	4,41	8,60					
☑	B11	1556T	85,11	31,44	2,29	13,73					
☑	B12	1556N	86,13	36,16	4,18	8,66					
☑	C1	1507T	80,98	30,88	2,65	11,65					
☑	C2	1507N	80,77	34,09	2,69	12,67					
☑	C3	1531T	81,09	31,65	2,74	11,55					
☑	C4	1531N	80,86	35,54	2,74	12,99					
☑	C5	1556T	81,16	31,11	2,73	11,40					
☑	C6	1556N	80,72	35,26	2,71	13,01					
☑	C7	1507T	85,89	35,11	4,12	8,53					
☑	C8	1507N	85,35	12,89	1,71	7,55	87,62	14,95	2,28	6,55	
☑	C9	1531T	85,36	20,42	2,22	9,20	87,76	10,41	1,94	5,38	
☑	C10	1531N	86,16	39,05	4,98	7,84					
☑	C11	1556T	85,42	29,74	2,38	12,50					

Results

Inc	Pos	Sample Name	Peak 1 Tm	Peak 1 Area	Peak 1 Width	Peak 1 Height	Peak 2 Tm	Peak 2 Area	Peak 2 Width	Peak 2 Height	Status
☑	C12	1556N	86,50	33,76	4,41	7,66					
☑	D1	1560T	80,38	43,60	2,67	16,32					
☑	D2	1560T	80,54	39,12	2,70	14,47					
☑	D3	1560T	80,93	33,48	2,72	12,33					
☑	D4	1707T	80,21	36,68	2,64	13,92					
☑	D5	1707T	80,07	36,89	2,95	12,51					
☑	D6	1707T	80,56	25,43	2,81	9,07					
☑	D7	1560T	81,04	32,63	2,63	12,40					
☑	D8	1560T	81,06	29,39	2,41	12,21					
☑	D9	1560T	81,50	33,42	3,69	9,05					
☑	D10	1707T	77,96	8,18	2,47	3,32	82,68	27,39	3,06	8,96	
☑	D11	1707T	84,94	24,27	6,64	3,65	88,39	6,13	1,42	4,30	
☑	D12	1707T	77,63	7,99	1,87	4,28	83,70	28,92	2,95	9,81	
☑	E1	1560N	80,79	37,82	2,71	13,98					
☑	E2	1560N	80,90	36,31	2,73	13,28					
☑	E3	1560N	80,97	37,71	2,74	13,75					
☑	E4	1707N	80,80	41,11	2,68	15,34					
☑	E5	1707N	80,77	41,77	2,73	15,31					
☑	E6	1707N	81,06	37,27	2,70	13,82					
☑	E7	1560N	81,17	36,60	2,40	15,24					
☑	E8	1560N	80,97	38,31	2,37	16,19					
☑	E9	1560N	81,03	38,94	2,38	16,38					
☑	E10	1707N	81,37	32,18	2,42	13,29					
☑	E11	1707N	81,04	38,56	2,43	15,89					
☑	E12	1707N	80,97	41,92	2,47	16,98					
☑	F1	1674T	80,48	37,24	2,66	14,03					
☑	F2	1674T	80,46	40,00	2,67	14,98					
☑	F3	1674T	80,65	35,46	2,65	13,38					
☑	F4	1711T	80,75	37,75	2,70	13,96					
☑	F5	1711T	80,70	42,21	2,73	15,49					
☑	F6	1711T	80,80	38,13	2,67	14,28					
☑	F7	1674T	81,02	30,95	2,31	13,38					
☑	F8	1674T	80,81	37,62	2,32	16,24					
☑	F9	1674T	80,77	37,37	2,32	16,11					
☑	F10	1711T	82,00	34,00	4,09	8,32					
☑	F11	1711T	82,75	34,18	5,09	6,71					

Results

Inc	Pos	Sample Name	Peak 1 Tm	Peak 1 Area	Peak 1 Width	Peak 1 Height	Peak 2 Tm	Peak 2 Area	Peak 2 Width	Peak 2 Height	Status
☑	F12	1711T	81,52	35,78	3,65	9,79					
☑	G1	1674N	80,37	33,83	2,84	11,90					
☑	G2	1674N	80,01	40,75	2,84	14,36					
☑	G3	1674N	80,53	32,76	2,50	13,11					
☑	G4	1711N	80,32	32,35	2,55	12,67					
☑	G5	1711N	80,42	42,14	2,72	15,47					
☑	G6	1711N	80,59	36,72	2,62	14,02					
☑	G7	1674N	83,05	38,06	4,23	9,00					
☑	G8	1674N	82,81	38,41	3,16	12,14					
☑	G9	1674N	82,64	39,83	4,01	9,94					
☑	G10	1711N	80,93	38,28	2,41	15,91					
☑	G11	1711N	81,09	35,49	2,40	14,82					
☑	G12	1711N	80,71	41,49	2,38	17,44					
☑	H1	1700T	80,70	38,05	2,75	13,84					
☑	H2	1700T	80,80	39,58	2,78	14,23					
☑	H3	1700T	80,76	38,64	2,79	13,84					
☑	H4	K-	76,82	42,57	3,97	10,72					
☑	H5	K-									
☑	H6	K-									
☑	H7	1700T	81,04	45,04	3,42	13,15					
☑	H8	1700T	80,87	47,28	3,46	13,66					
☑	H9	1700T	81,35	21,70	3,80	5,71	86,35	16,07	3,73	4,31	
☑	H10	RT-	76,84	40,11	3,79	10,58					
☑	H11	RT-									
☑	H12	RT-	80,82	5,46	1,90	2,88					

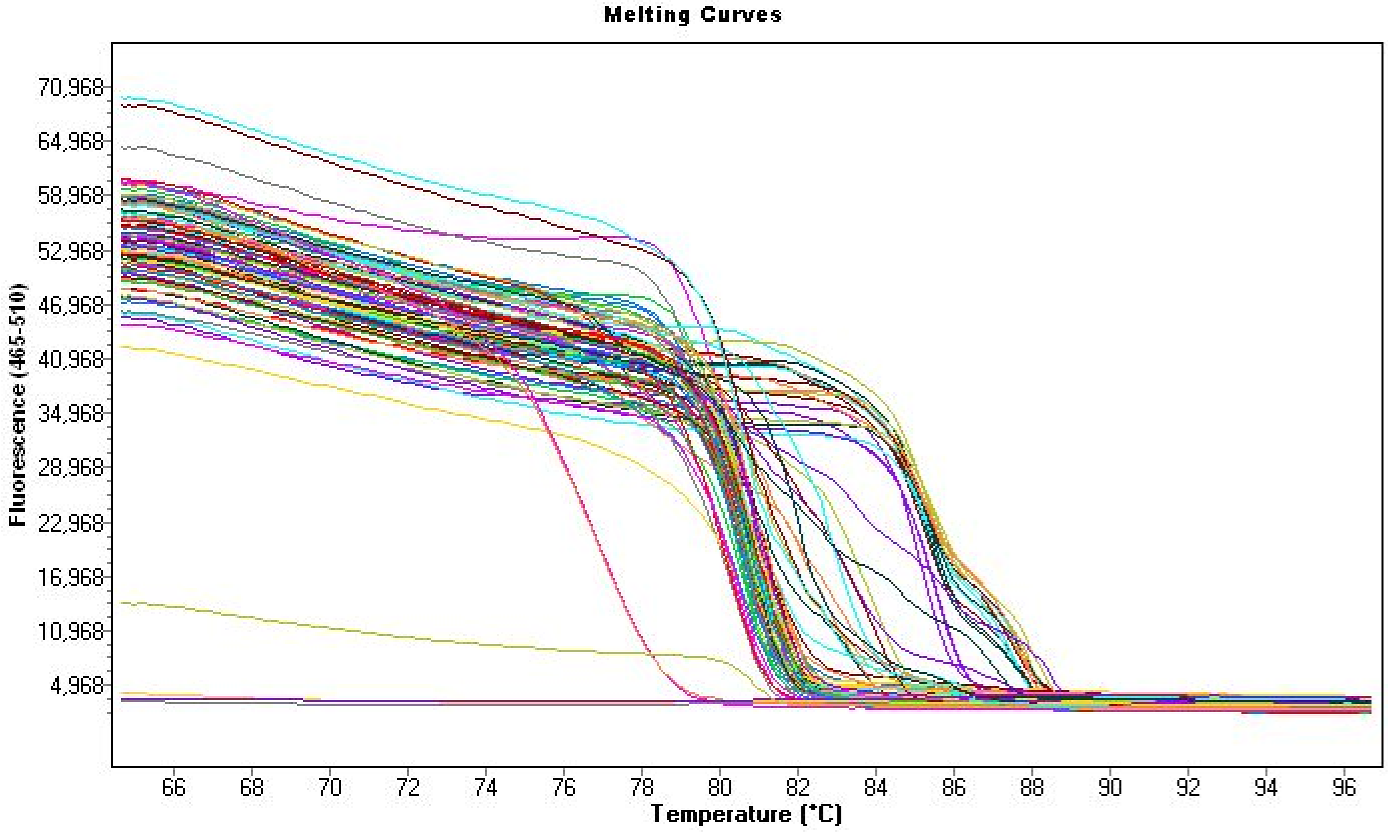
Melting Curves
70,968
64,968
58,968
52,968
46,968
40,968
34,968
28,968
22,968
16,968
10,968
4,968
Fluorescence (465-510)
66
68
70
72
74
76
78
80
82
84
86
88
90
92
94
96
Temperature (°C)

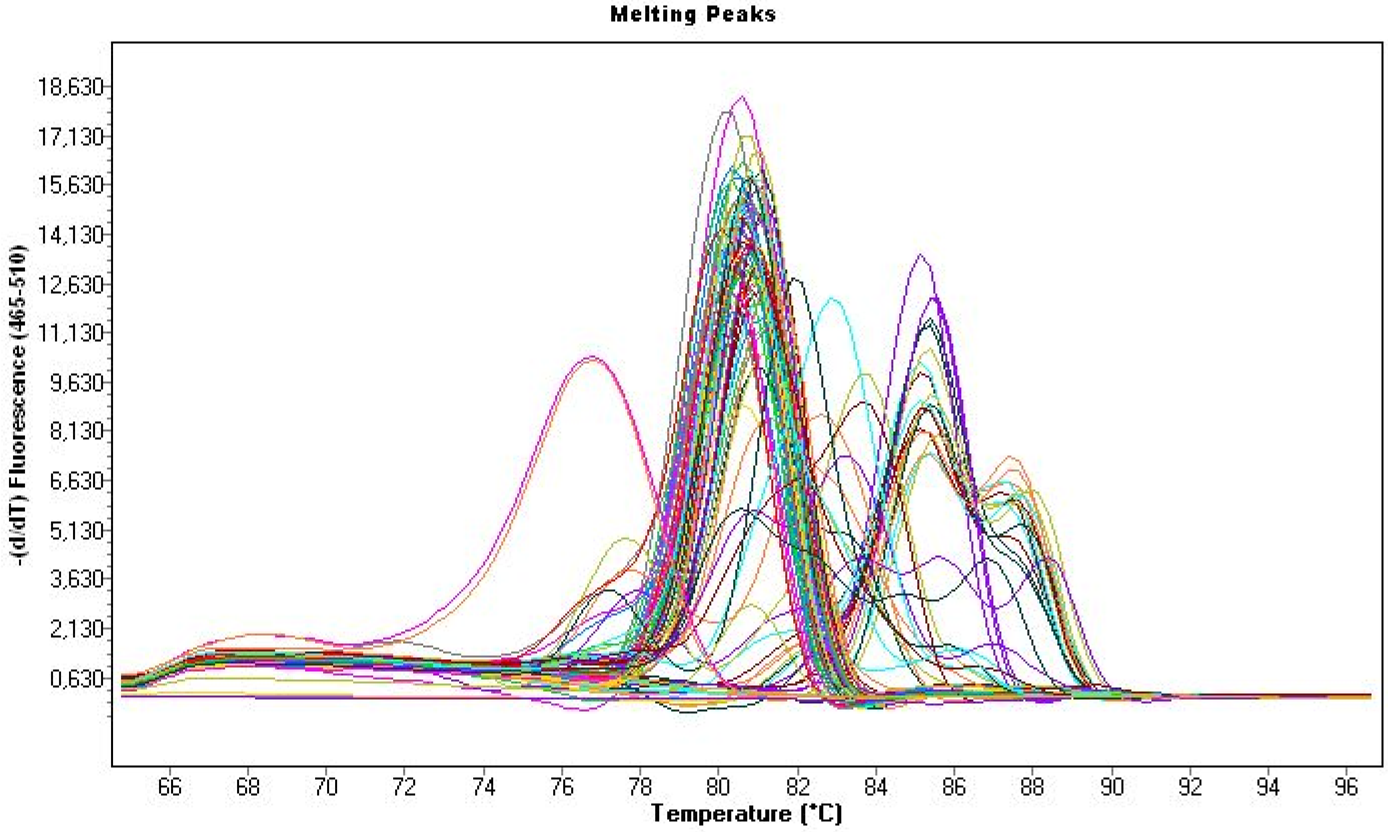
Melting Peaks
18,630
17,130
15,630
14,130
12,630
11,130
9,630
8,130
6,630
5,130
3,630
2,130
0,630
-(d/dT) Fluorescence (465-510)
66
68
70
72
74
76
78
80
82
84
86
88
90
92
94
96
Temperature (°C)
