# Supplementary material for: MicroRNAs and Their Inhibition in Modulating SLC5A8 Expression in the Context of Papillary Thyroid Carcinoma
Source: Int J Mol Sci. 2025 Aug 15;26(16):7889. doi: 10.3390/ijms26167889 (PMC12386254; doi:10.3390/ijms26167889)
Supplement: Supplementary file 1 [file ijms-26-07889-s001.zip › ijms-3558049-supplementary/Manuscript data/Fig1 data/Data/2013-05-07 HPRT AIT 1674 1697.PDF]

Wojtek_2013-05-07_HPRT AIT 1674 1697

Programs

Program Name	pre-incubation		
Cycles	1	Analysis Mode	None

Target (°C)	Acquisition Mode	Hold (hh:mm:ss)	Ramp Rate (°C/s)	Acquisitions (per °C)	Sec Target (°C)	Step size (°C)	Step Delay (cycles)
95	None	00:10:00	4,40		0	0	0

Program Name	amplification		
Cycles	55	Analysis Mode	Quantification

Target (°C)	Acquisition Mode	Hold (hh:mm:ss)	Ramp Rate (°C/s)	Acquisitions (per °C)	Sec Target (°C)	Step size (°C)	Step Delay (cycles)
95	None	00:00:15	4,40		0	0	0
57	None	00:00:15	2,20		0	0	0
72	Single	00:00:15	4,40		0	0	0

Program Name	melting curve		
Cycles	1	Analysis Mode	Melting Curves

Target (°C)	Acquisition Mode	Hold (hh:mm:ss)	Ramp Rate (°C/s)	Acquisitions (per °C)	Sec Target (°C)	Step size (°C)	Step Delay (cycles)
95	None	00:00:05	4,40		0	0	0
65	None	00:01:00	2,20		0	0	0
97	Continuous		0,11	5	0	0	0

Program Name	cooling		
Cycles	1	Analysis Mode	None

Target (°C)	Acquisition Mode	Hold (hh:mm:ss)	Ramp Rate (°C/s)	Acquisitions (per °C)	Sec Target (°C)	Step size (°C)	Step Delay (cycles)
40	None	00:00:30	2,20		0	0	0

Tm Calling for All (Tm Calling)

Results

			Peak 1				Peak 2				
Inc	Pos	Sample Name	Tm	Area	Width	Height	Tm	Area	Width	Height	Status
☑	A1	1674T	80,06	56,43	2,72	20,77					
☑	A2	1674T	80,18	53,29	2,83	18,86					
☑	A3	1674T	80,18	52,90	2,76	19,16					
☑	B1	1674N	79,76	47,46	3,09	15,38					
☑	B2	1674N	79,74	45,51	3,09	14,71					

Results

Inc	Pos	Sample Name	Peak 1 Tm	Peak 1 Area	Peak 1 Width	Peak 1 Height	Peak 2 Tm	Peak 2 Area	Peak 2 Width	Peak 2 Height	Status
☑	B3	1674N	79,72	45,71	3,03	15,08					
☑	C1	1697T	80,20	52,28	2,85	18,37					
☑	C2	1697T	80,24	48,26	2,76	17,51					
☑	C3	1697T	80,26	48,69	2,78	17,49					
☑	D1	1697N	80,25	51,11	2,81	18,22					
☑	D2	1697N	80,29	52,34	2,79	18,75					
☑	D3	1697N	80,35	52,44	2,79	18,80					
☑	E1	1674T	80,70	46,98	2,41	19,46					
☑	E2	1674T	80,67	42,79	2,41	17,77					
☑	E3	1674T	80,69	43,97	2,39	18,36					
☑	F1	1674N	82,59	48,24	2,44	19,76					
☑	F2	1674N	77,69	7,01	1,61	4,35	82,18	38,23	2,85	13,42	
☑	F3	1674N	81,53	36,91	2,25	16,39	85,67	4,50	1,97	2,28	
☑	G1	1697T	80,78	45,52	2,46	18,49					
☑	G2	1697T	80,70	43,46	2,70	16,07					
☑	G3	1697T	80,72	45,43	2,41	18,86					
☑	H1	1697N	80,81	47,56	2,47	19,22					
☑	H2	1697N	80,88	42,06	2,43	17,32					
☑	H3	1697N	80,76	47,92	2,48	19,36					
☑	H4	RT-									
☑	H5	K-	82,39	3,33	1,95	1,71					
☑	H6	RT-									
☑	H7	K-	70,71	0,68	7,55	0,09					

Melting Curves

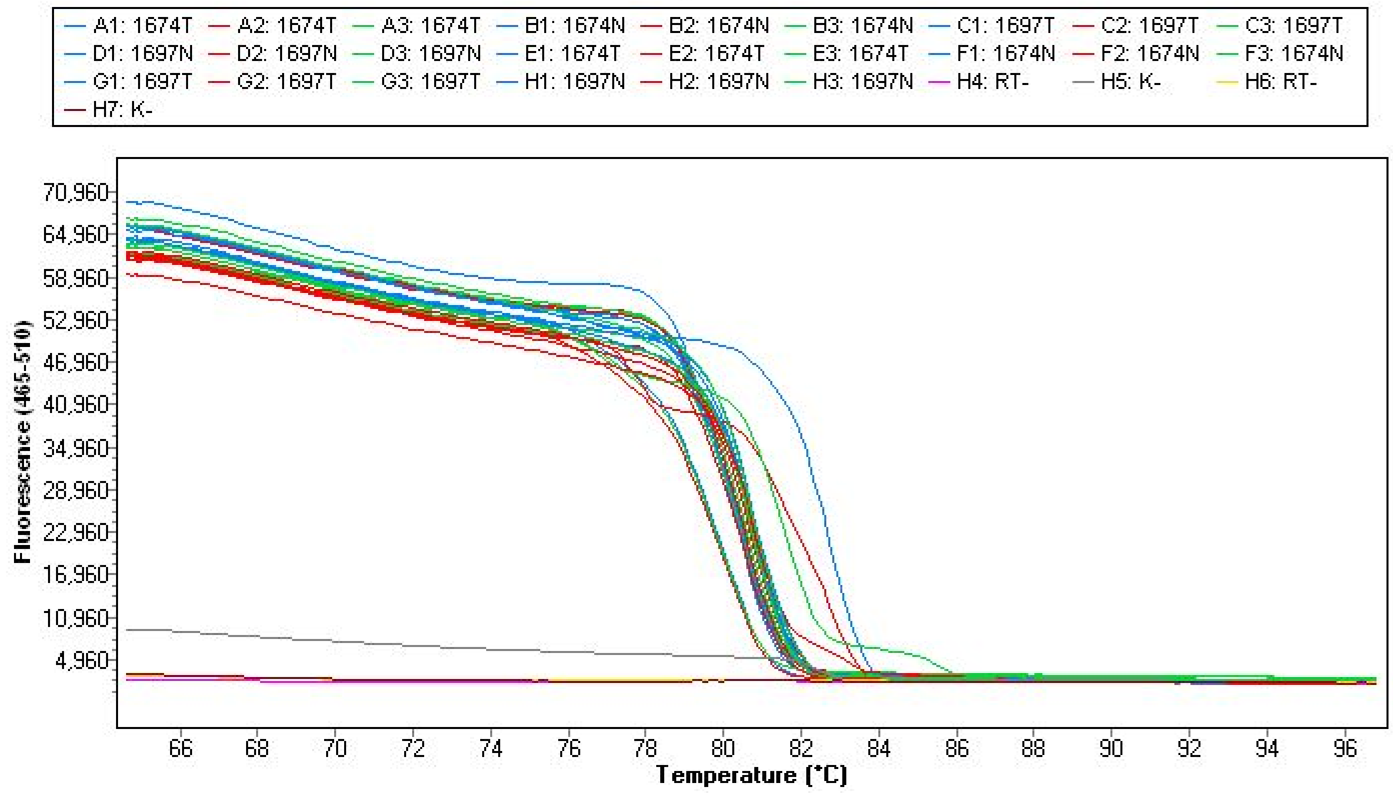

Melting Peaks

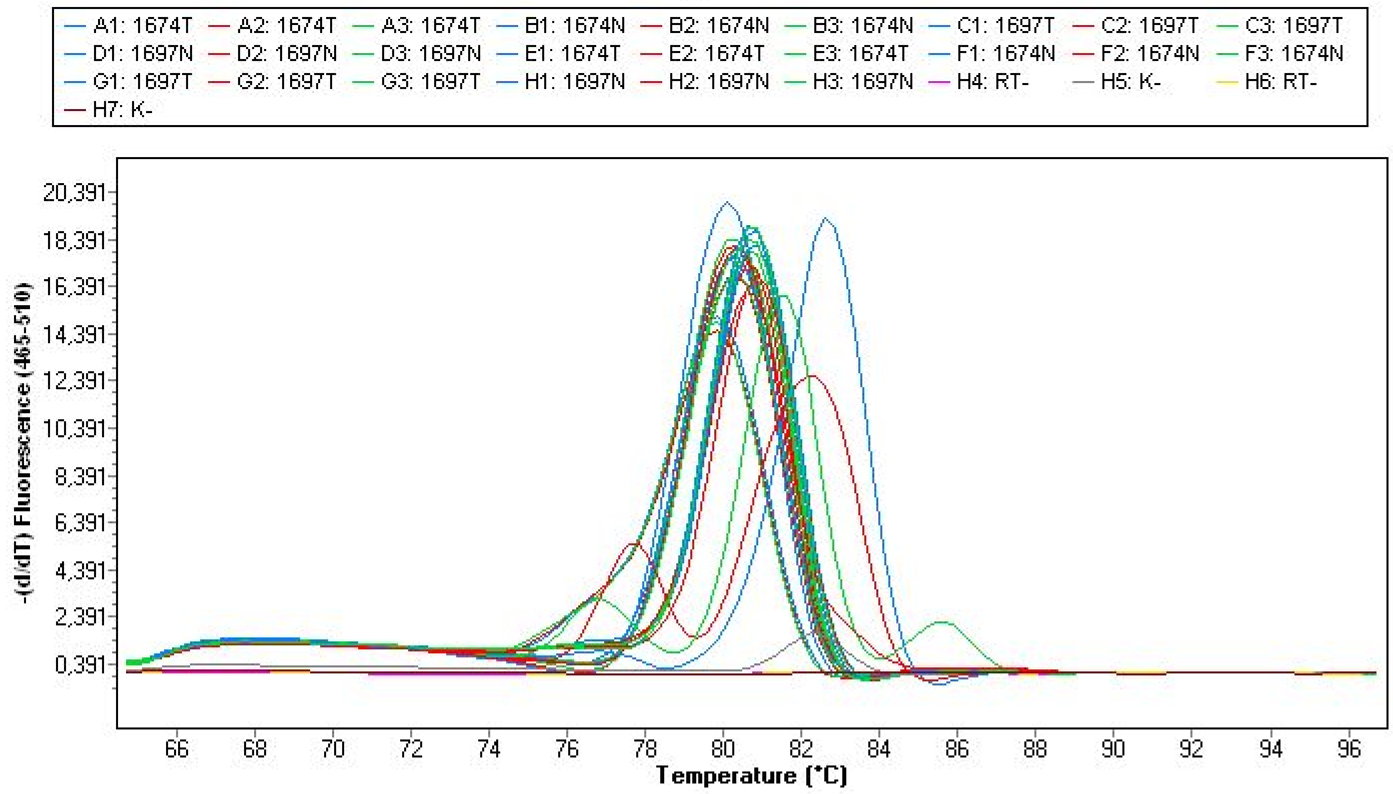

Abs Quant/2nd Derivative Max for All (Abs Quant/2nd Derivative Max)

Statistics

Samples	Mean Cp	Std Cp	Mean conc	Std conc
A1, A2, A3	26,43	0,12		
B1, B2, B3	38,24	0,79		
C1, C2, C3	28,66	0,08		
D1, D2, D3	26,87	0,10		
E1, E2, E3	26,78	0,05		
F1, F2, F3	41,21	1,23		
G1, G2, G3	32,05	0,16		
H1, H2, H3	25,88	0,06		

Amplification Curves

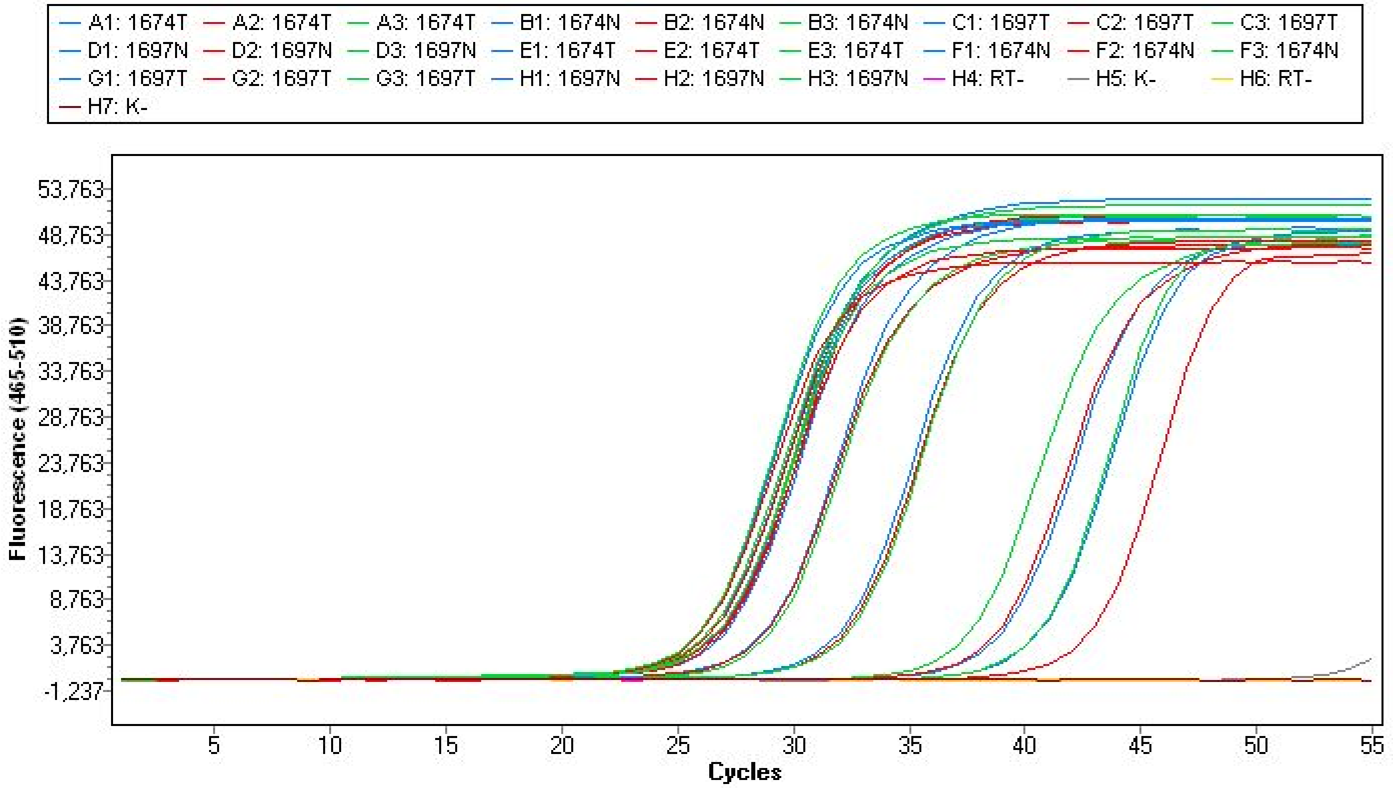

Advanced Relative Quantification for All (Relative Quantification)

Results

Bar Chart	Pairing	Sample Name	Target Name		Tgt Cp	Ref. Cp	Ratios		Corr/Multi Factor	Status
			Targets	References	Mean	Mean	Tgt/Ref.	Norm		
☑	E1/A1	1674T	AIT	HPRT	26,78	26,43	0,7809		1/1	
☑	F1/B1	1674N	AIT	HPRT	41,21	38,24	0,1275		1/1	
☑	G1/C1	1697T	AIT	HPRT	32,05	28,66	9,54E-2		1/1	
☑	H1/D1	1697N	AIT	HPRT	25,88	26,87	1,989		1/1	

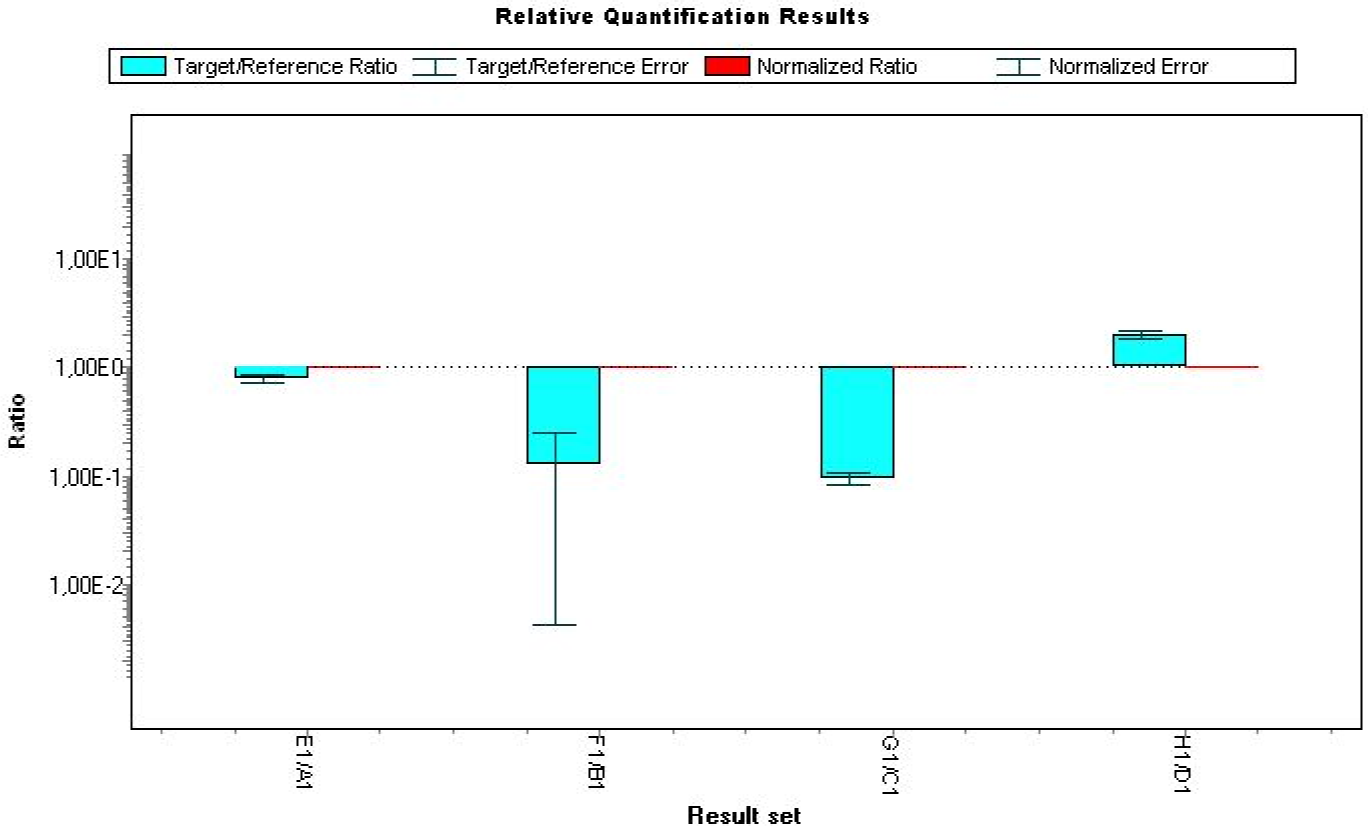
Relative Quantification Results
Target/Reference Ratio
Target/Reference Error
Normalized Ratio
Normalized Error
1,00E1
1,00E0
1,00E-1
1,00E-2
Ratio
E1/A1
F1/B1
G1/C1
H1/D1
Result set
