# Supplementary material for: MicroRNAs and Their Inhibition in Modulating SLC5A8 Expression in the Context of Papillary Thyroid Carcinoma
Source: Int J Mol Sci. 2025 Aug 15;26(16):7889. doi: 10.3390/ijms26167889 (PMC12386254; doi:10.3390/ijms26167889)

LightCycler® 480 Software Report

Wojtek_2013-06-18_HPRT AIT 1534-1778

Programs

Program Name	pre-incubation		
Cycles	1	Analysis Mode	None

Target (°C)	Acquisition Mode	Hold (hh:mm:ss)	Ramp Rate (°C/s)	Acquisitions (per °C)	Sec Target (°C)	Step size (°C)	Step Delay (cycles)
95	None	00:10:00	4,40		0	0	0

Program Name	amplification		
Cycles	45	Analysis Mode	Quantification

Target (°C)	Acquisition Mode	Hold (hh:mm:ss)	Ramp Rate (°C/s)	Acquisitions (per °C)	Sec Target (°C)	Step size (°C)	Step Delay (cycles)
95	None	00:00:15	4,40		0	0	0
57	None	00:00:15	2,20		0	0	0
72	Single	00:00:15	4,40		0	0	0

Program Name	melting curve		
Cycles	1	Analysis Mode	Melting Curves

Target (°C)	Acquisition Mode	Hold (hh:mm:ss)	Ramp Rate (°C/s)	Acquisitions (per °C)	Sec Target (°C)	Step size (°C)	Step Delay (cycles)
95	None	00:00:05	4,40		0	0	0
65	None	00:01:00	2,20		0	0	0
97	Continuous		0,11	5	0	0	0

Program Name	cooling		
Cycles	1	Analysis Mode	None

Target (°C)	Acquisition Mode	Hold (hh:mm:ss)	Ramp Rate (°C/s)	Acquisitions (per °C)	Sec Target (°C)	Step size (°C)	Step Delay (cycles)
40	None	00:00:30	2,20		0	0	0

Tm Calling for All (Tm Calling)

Results

			Peak 1				Peak 2				
Inc	Pos	Sample Name	Tm	Area	Width	Height	Tm	Area	Width	Height	Status
☑	A7	1534T	80,65	45,29	2,61	17,37					
☑	A8	1534T	80,65	46,32	2,61	17,75					
☑	A9	1534T	80,75	48,28	2,76	17,50					
☑	A10	1534T	80,86	44,37	2,46	18,07					
☑	A11	1534T	80,92	44,11	2,45	17,98					

Results

Inc	Pos	Sample Name	Peak 1				Peak 2				Status
			Tm	Area	Width	Height	Tm	Area	Width	Height	
☑	A12	1534T	80,96	41,91	2,49	16,80					
☑	B7	1534N	80,66	43,43	2,61	16,62					
☑	B8	1534N	80,58	44,93	2,62	17,12					
☑	B9	1534N	80,76	47,02	2,77	16,99					
☑	B10	1534N	80,99	40,85	2,38	17,15					
☑	B11	1534N	80,86	44,53	2,46	18,08					
☑	B12	1534N	81,02	39,85	2,46	16,23					
☑	C7	1691T	80,76	41,63	3,94	10,56					
☑	C8	1691T	80,48	39,11	2,66	14,69					
☑	C9	1691T	80,51	41,63	2,86	14,54					
☑	C10	1691T	81,41	36,52	3,56	10,25					
☑	C11	1691T	81,19	37,40	3,31	11,30					
☑	C12	1691T	81,01	37,18	2,71	13,73					
☑	D7	1691N	80,70	44,69	2,70	16,57					
☑	D8	1691N	80,85	45,33	2,72	16,69					
☑	D9	1691N	80,85	44,91	2,71	16,56					
☑	D10	1691N	80,29	10,19	1,92	5,31					
☑	D11	1691N	81,05	39,07	2,47	15,85					
☑	D12	1691N	81,06	37,75	2,45	15,40					
☑	E7	1778T	80,75	44,65	2,72	16,44					
☑	E8	1778T	80,75	46,46	2,80	16,57					
☑	E9	1778T	80,71	47,16	2,67	17,67					
☑	E10	1778T	81,16	38,44	2,54	15,14					
☑	E11	1778T	81,10	39,28	2,51	15,65					
☑	E12	1778T	81,09	40,62	2,51	16,20					
☑	F7	1778N	80,77	43,91	2,71	16,23					
☑	F8	1778N	80,64	44,21	2,65	16,66					
☑	F9	1778N	80,82	47,17	2,71	17,42					
☑	F10	1778N	81,07	38,85	2,42	16,02					
☑	F11	1778N	81,08	40,43	2,52	16,06					
☑	F12	1778N	80,78	54,07	2,30	23,56					
☑	G7	K-									
☑	G8	K-									

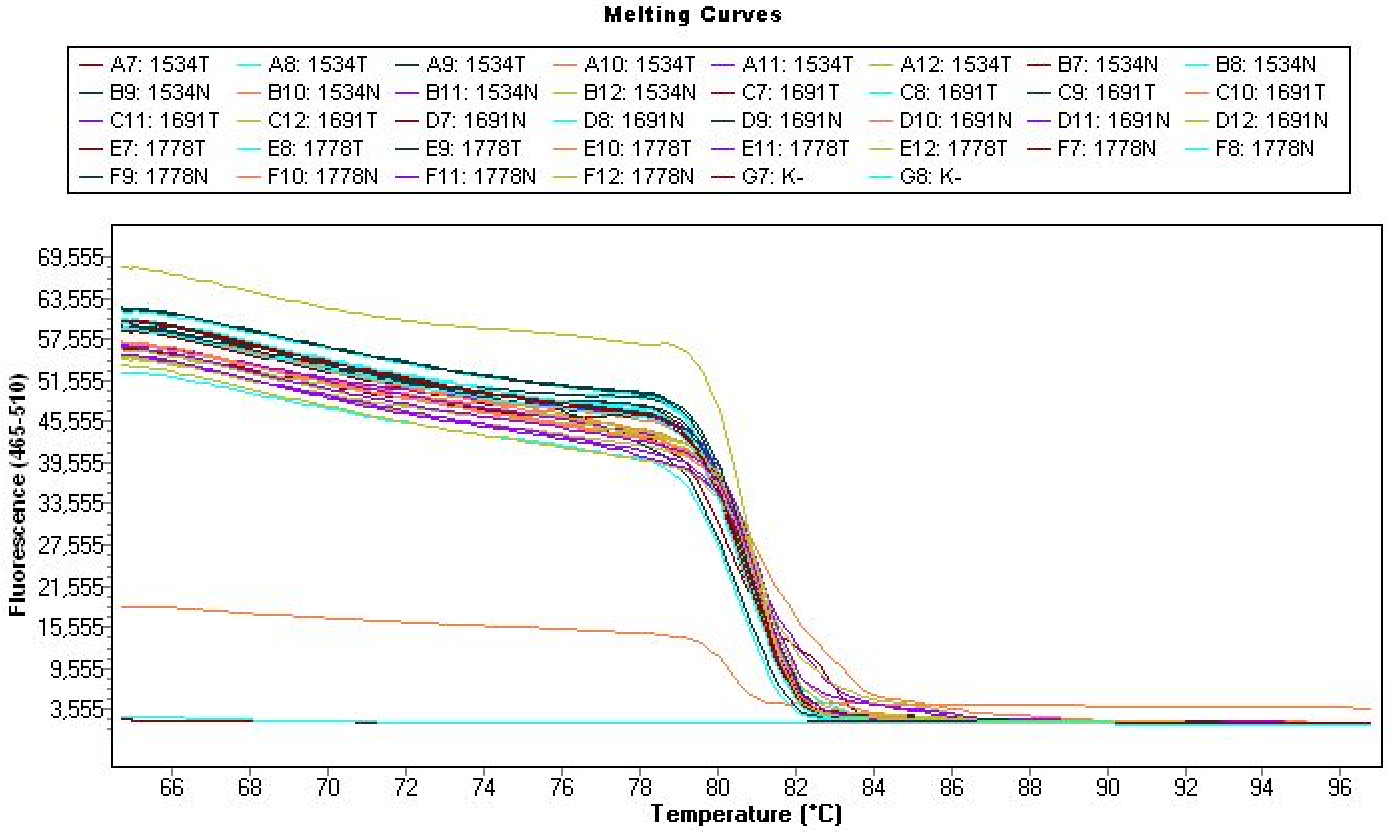

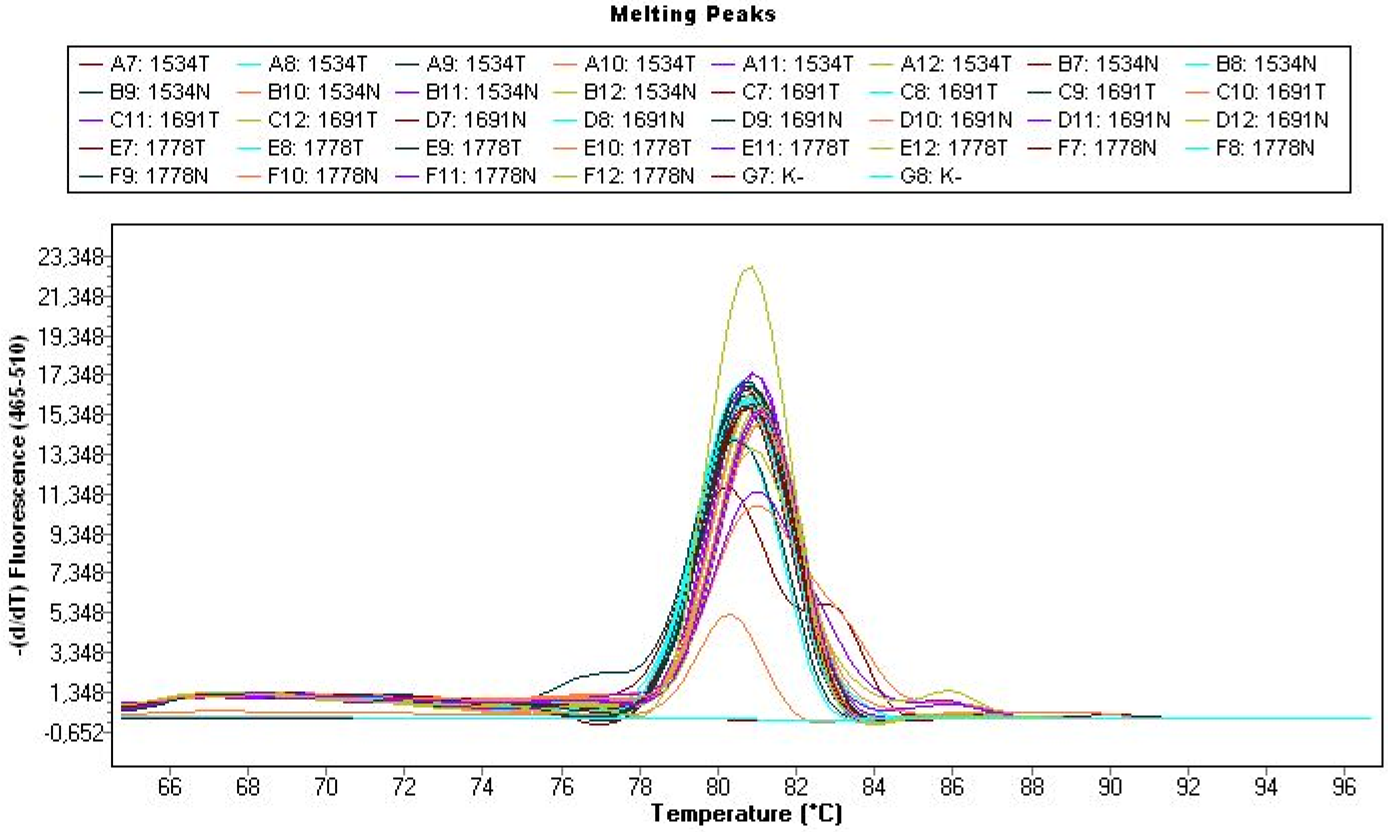

Abs Quant/2nd Derivative Max for All (Abs Quant/2nd Derivative Max)

Statistics

Samples	Mean Cp	Std Cp	Mean conc	Std conc
A7, A8, A9	28,49	0,20		
A10, A11, A12	25,71	0,04		
B7, B8, B9	27,42	0,11		
B10, B11, B12	25,69	0,05		
C7, C8, C9	32,14	0,36		
C10, C11, C12	32,07	0,35		
D7, D8, D9	28,35	0,10		
D10, D11, D12	27,52	0,30		
E7, E8, E9	27,70	0,04		
E10, E11, E12	27,63	0,04		
F7, F8, F9	27,68	0,07		
F10, F11, F12	27,50	0,02		

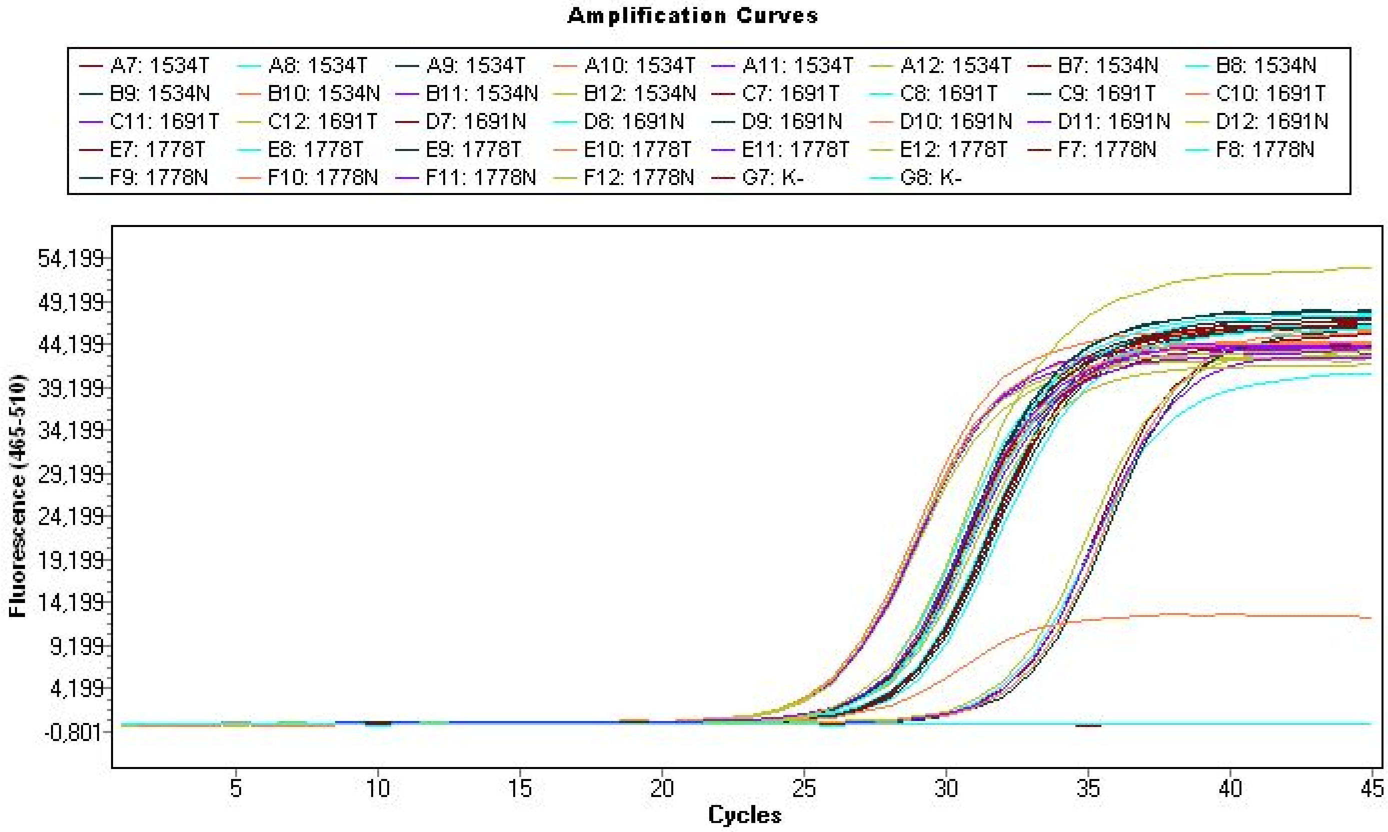

Supplement: Supplementary file 1 [file ijms-26-07889-s001.zip › ijms-3558049-supplementary/Manuscript data/Fig1 data/Data/2013-06-18 HPRT AIT 1534-1778.PDF]
