# Supplementary material for: MicroRNAs and Their Inhibition in Modulating SLC5A8 Expression in the Context of Papillary Thyroid Carcinoma
Source: Int J Mol Sci. 2025 Aug 15;26(16):7889. doi: 10.3390/ijms26167889 (PMC12386254; doi:10.3390/ijms26167889)
Supplement: Supplementary file 1 [file ijms-26-07889-s001.zip › ijms-3558049-supplementary/Manuscript data/Fig1 data/Data/2013-06-21 HPRT AIT 548-1928.PDF]

Roche

Wojtek_2013-06-21_HPRT AIT 548-1928

Programs

Program Name	pre-incubation		
Cycles	1	Analysis Mode	None

Target (°C)	Acquisition Mode	Hold (hh:mm:ss)	Ramp Rate (°C/s)	Acquisitions (per °C)	Sec Target (°C)	Step size (°C)	Step Delay (cycles)
95	None	00:10:00	4,40		0	0	0

Program Name	amplification		
Cycles	45	Analysis Mode	Quantification

Target (°C)	Acquisition Mode	Hold (hh:mm:ss)	Ramp Rate (°C/s)	Acquisitions (per °C)	Sec Target (°C)	Step size (°C)	Step Delay (cycles)
95	None	00:00:15	4,40		0	0	0
57	None	00:00:15	2,20		0	0	0
72	Single	00:00:15	4,40		0	0	0

Program Name	melting curve		
Cycles	1	Analysis Mode	Melting Curves

Target (°C)	Acquisition Mode	Hold (hh:mm:ss)	Ramp Rate (°C/s)	Acquisitions (per °C)	Sec Target (°C)	Step size (°C)	Step Delay (cycles)
95	None	00:00:05	4,40		0	0	0
65	None	00:01:00	2,20		0	0	0
97	Continuous		0,11	5	0	0	0

Program Name	cooling		
Cycles	1	Analysis Mode	None

Target (°C)	Acquisition Mode	Hold (hh:mm:ss)	Ramp Rate (°C/s)	Acquisitions (per °C)	Sec Target (°C)	Step size (°C)	Step Delay (cycles)
40	None	00:00:30	2,20		0	0	0

Tm Calling for All (Tm Calling)

Melting Curves

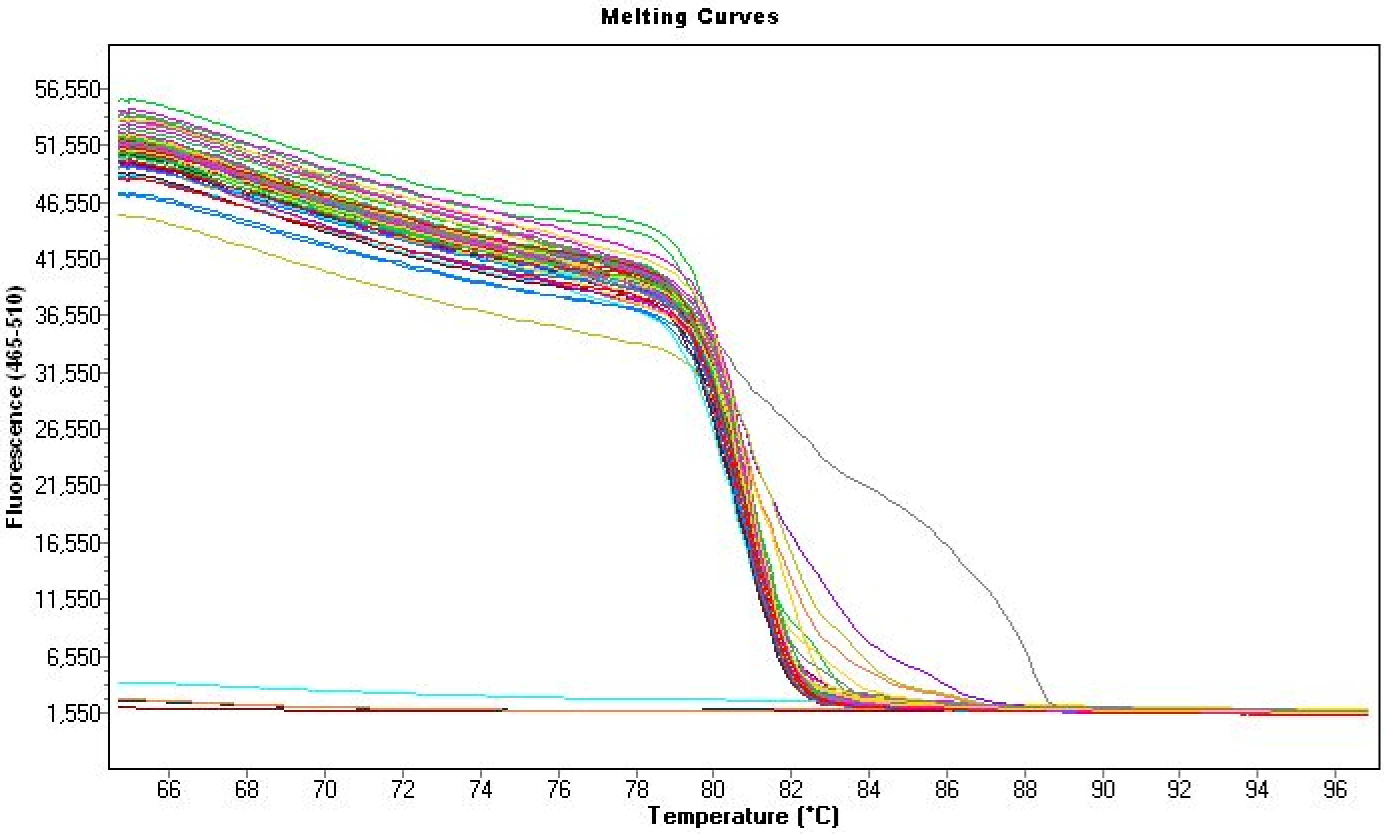

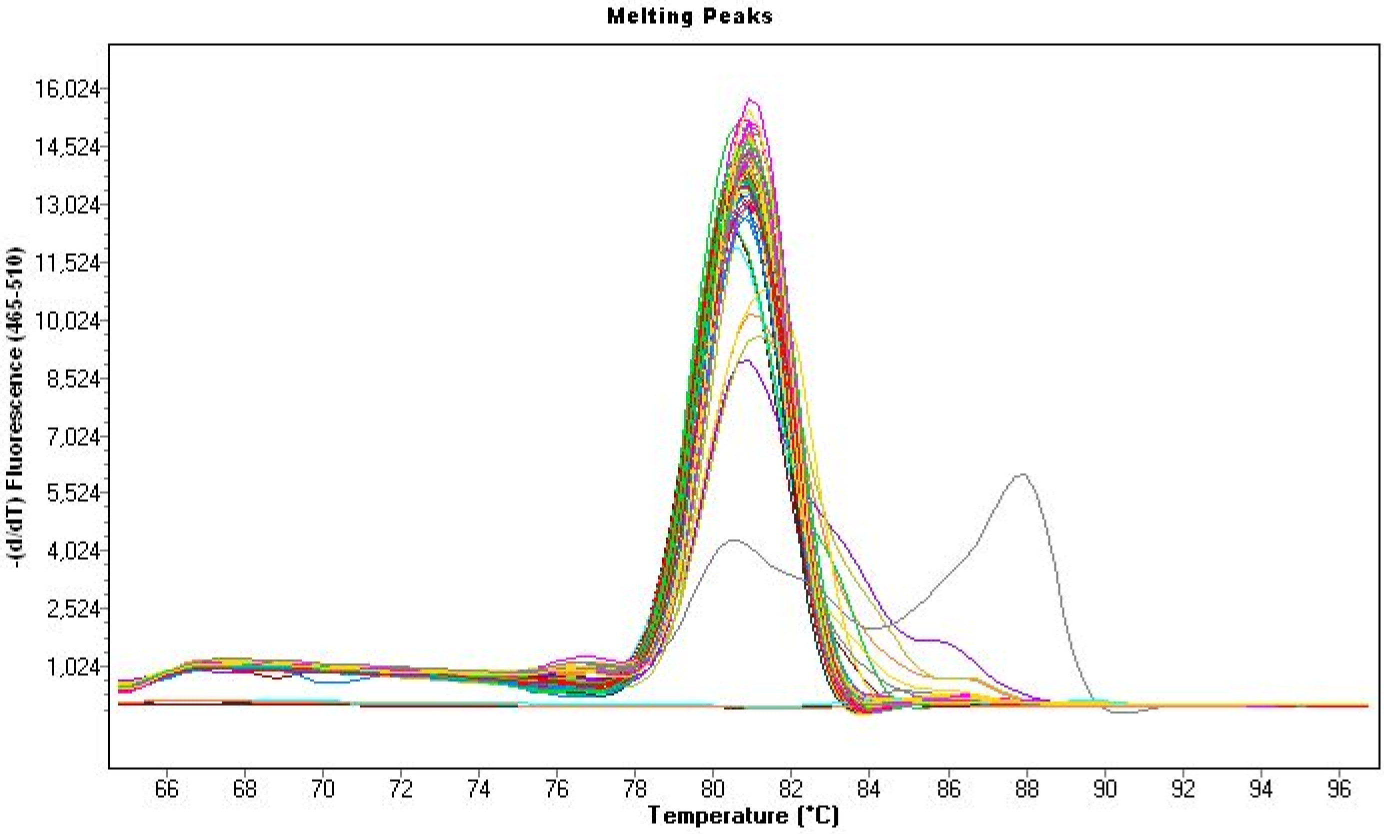

Abs Quant/2nd Derivative Max for All (Abs Quant/2nd Derivative Max)

Statistics

Samples	Mean Cp	Std Cp	Mean conc	Std conc
A1, A2, A3	28,19	0,17		
A4, A5, A6	29,26	0,17		
A7, A8, A9	31,08	0,19		
A10, A11, A12	31,01	0,06		
B1, B2, B3	27,09	0,10		
B4, B5, B6	28,14	0,15		
B7, B8, B9	27,00	0,13		
B10, B11, B12	26,63	0,01		
C1, C2, C3	29,89	0,04		
C4, C5, C6	32,97	0,97		
D1, D2, D3	27,27	0,16		
D4, D5, D6	27,97	0,09		
E1, E2, E3	27,26	0,16		
E4, E5, E6	27,10	0,07		
F1, F2, F3	26,85	0,04		
F4, F5, F6	26,40	0,05		
G1, G2, G3	27,28	0,15		
G4, G5, G6	27,08	0,03		
H1, H2, H3	27,84	0,10		
H4, H5, H6	27,37	0,13		

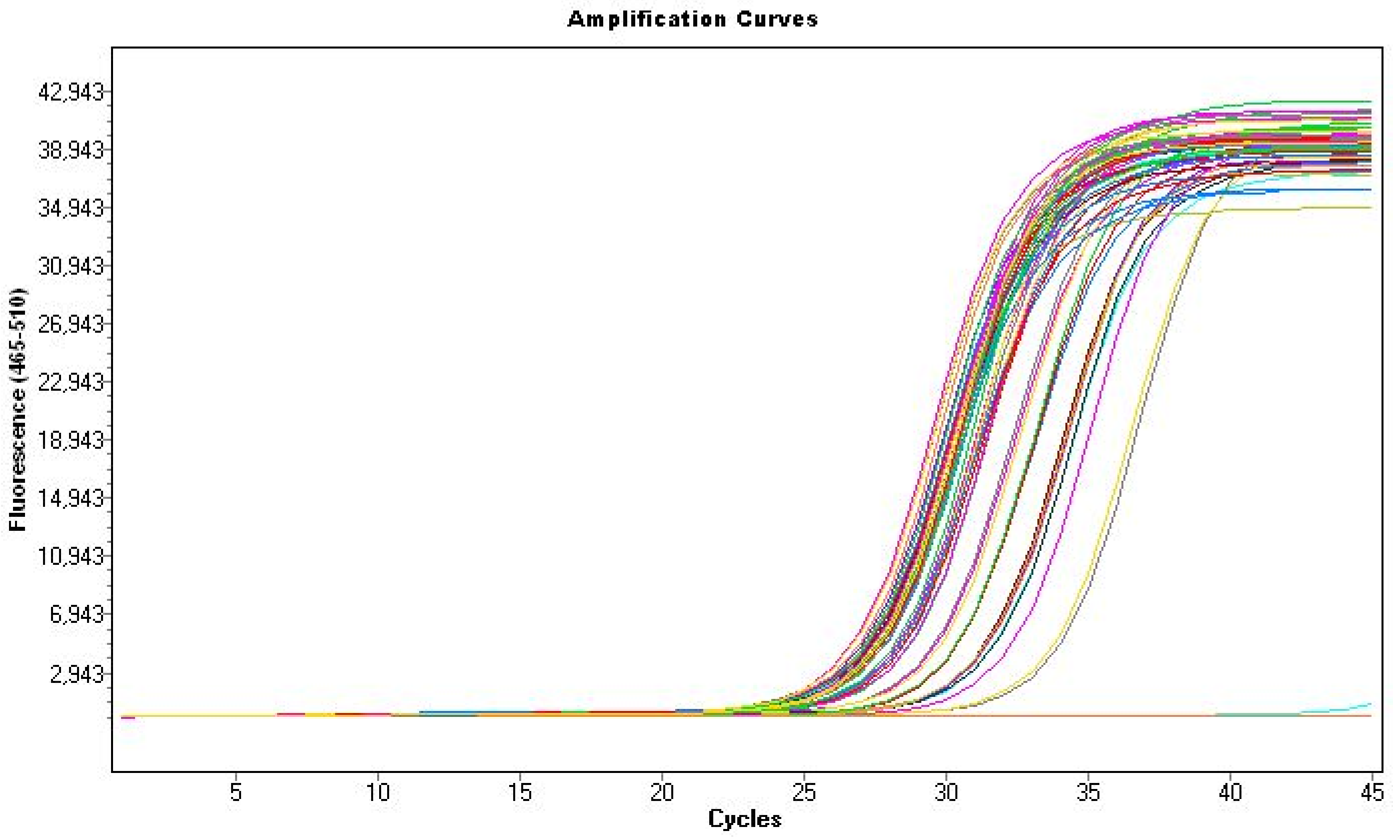
Amplification Curves
Fluorescence (465-510)
42,943
38,943
34,943
30,943
26,943
22,943
18,943
14,943
10,943
6,943
2,943
5
10
15
20
25
30
35
40
45
Cycles
