# Supplementary material for: MicroRNAs and Their Inhibition in Modulating SLC5A8 Expression in the Context of Papillary Thyroid Carcinoma
Source: Int J Mol Sci. 2025 Aug 15;26(16):7889. doi: 10.3390/ijms26167889 (PMC12386254; doi:10.3390/ijms26167889)
Supplement: Supplementary file 1 [file ijms-26-07889-s001.zip › ijms-3558049-supplementary/Manuscript data/Fig1 data/Data/2013-09-16 HPRT AIT 538-1707.PDF]

LightCycler® 480 Software

Report

Wojtek_2013-09-16_HPRT AIT 538 1707

Programs

Program Name	pre-incubation		
Cycles	1	Analysis Mode	None

Target (°C)	Acquisition Mode	Hold (hh:mm:ss)	Ramp Rate (°C/s)	Acquisitions (per °C)	Sec Target (°C)	Step size (°C)	Step Delay (cycles)
95	None	00:10:00	4,40		0	0	0

Program Name	amplification		
Cycles	45	Analysis Mode	Quantification

Target (°C)	Acquisition Mode	Hold (hh:mm:ss)	Ramp Rate (°C/s)	Acquisitions (per °C)	Sec Target (°C)	Step size (°C)	Step Delay (cycles)
95	None	00:00:15	4,40		0	0	0
57	None	00:00:15	2,20		0	0	0
72	Single	00:00:15	4,40		0	0	0

Program Name	melting curve		
Cycles	1	Analysis Mode	Melting Curves

Target (°C)	Acquisition Mode	Hold (hh:mm:ss)	Ramp Rate (°C/s)	Acquisitions (per °C)	Sec Target (°C)	Step size (°C)	Step Delay (cycles)
95	None	00:00:05	4,40		0	0	0
65	None	00:01:00	2,20		0	0	0
97	Continuous		0,11	5	0	0	0

Program Name	cooling		
Cycles	1	Analysis Mode	None

Target (°C)	Acquisition Mode	Hold (hh:mm:ss)	Ramp Rate (°C/s)	Acquisitions (per °C)	Sec Target (°C)	Step size (°C)	Step Delay (cycles)
40	None	00:00:30	2,20		0	0	0

Tm Calling for All (Tm Calling)

Melting Curves

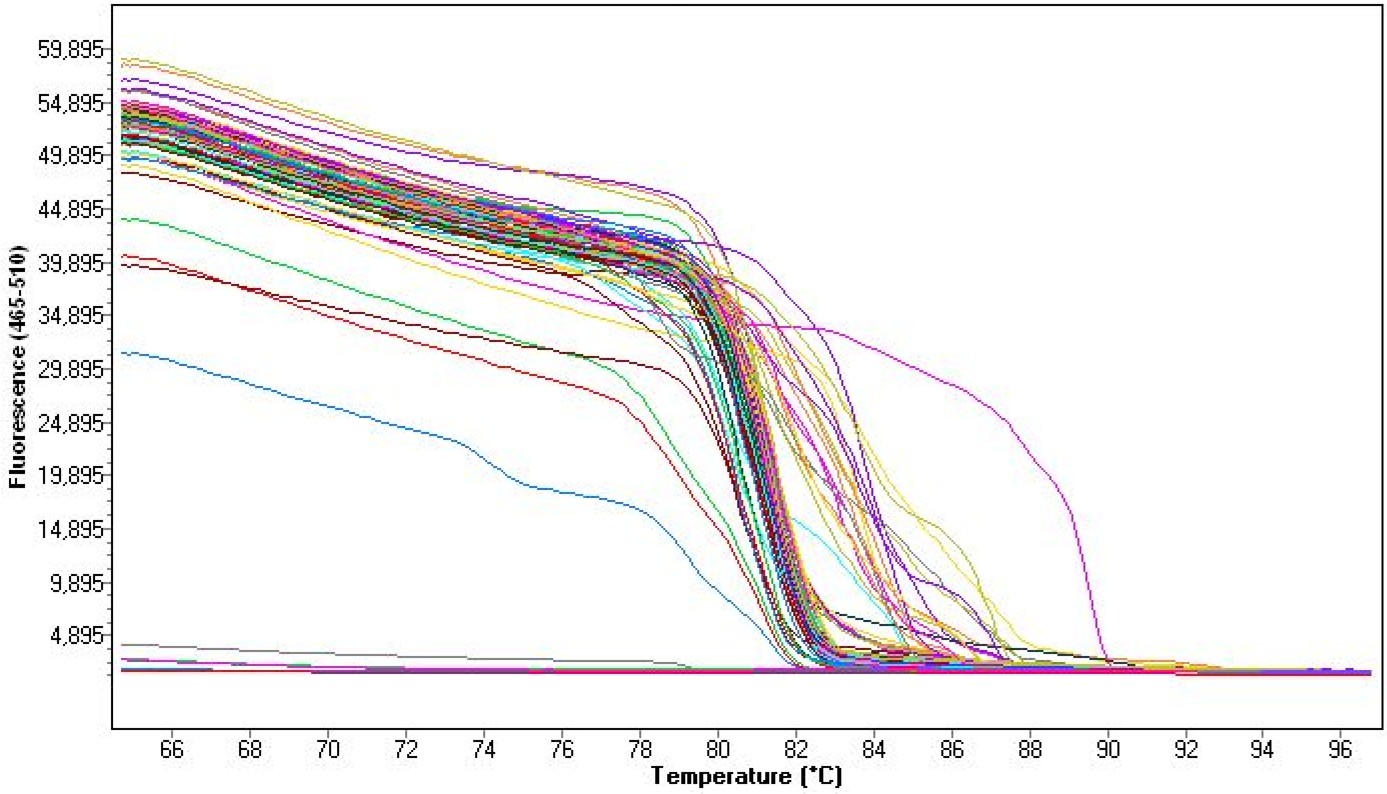

Melting Peaks

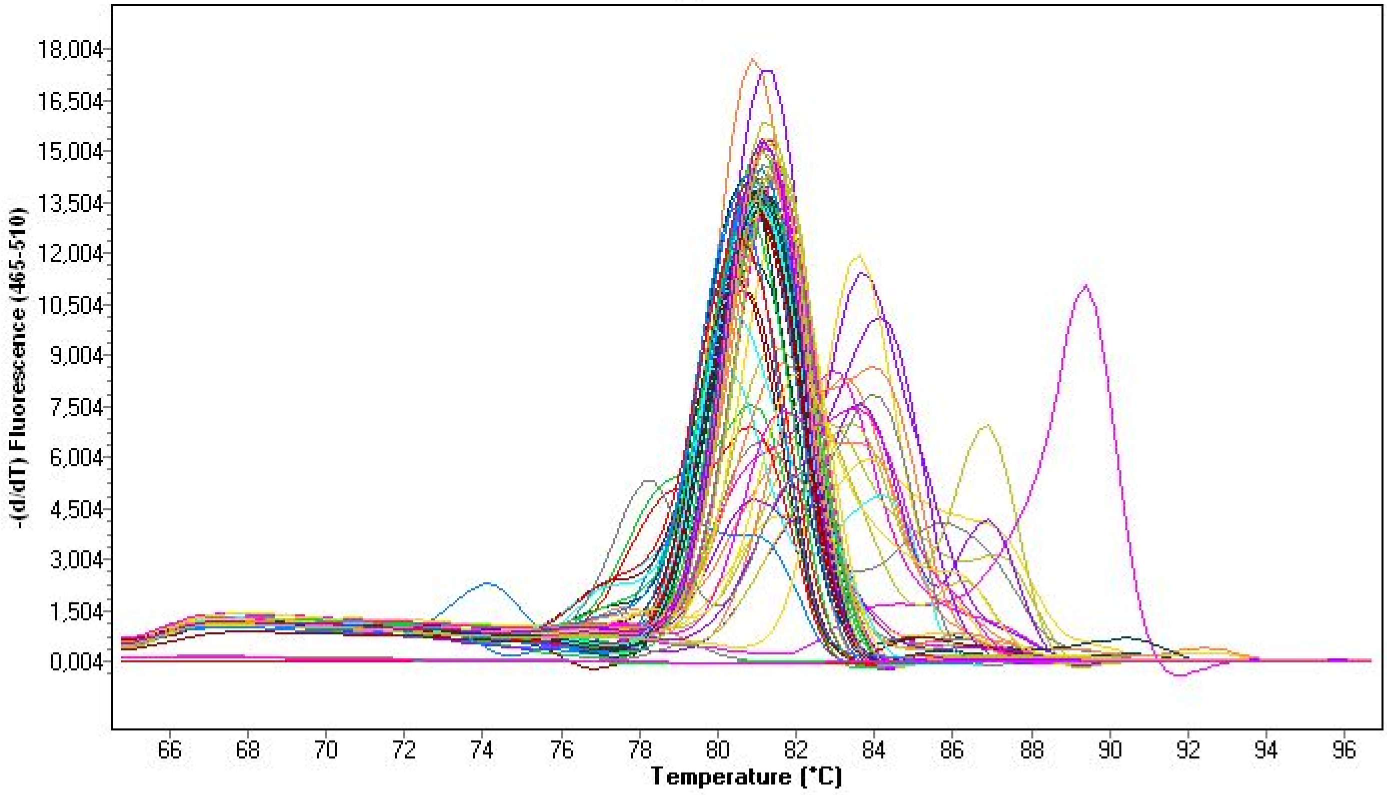

Abs Quant/2nd Derivative Max for All (Abs Quant/2nd Derivative Max)

Statistics

Samples	Mean Cp	Std Cp	Mean conc	Std conc
A1, A2, A3	26,60	0,27		
A4, A5, A6	26,00	0,02		
A7, A8, A9	27,47	0,15		
A10, A11, A12	26,60	0,96		
B1, B2, B3	27,40	0,43		
B4, B5, B6	32,91	0,44		
B7, B8, B9	30,45	0,19		
B10, B11, B12	34,41	0,30		
C1, C2, C3	40,00			
C4, C5, C6	40,00			
C7, C8, C9	33,38	0,60		
C10, C11, C12	33,10	0,41		
D1, D2, D3	26,38	0,29		
D4, D5, D6	27,70	0,08		
D7, D8, D9	27,62	0,44		
D10, D11, D12	27,53	0,06		
E1, E2, E3	26,92	0,01		
E4, E5, E6	27,55	0,13		
E7, E8, E9	33,34	0,13		
E10, E11, E12	34,40	0,16		
F1, F2, F3	28,02	0,89		
F4, F5, F6	27,04	0,07		
F7, F8, F9	25,74	0,01		
F10, F11, F12	24,19	0,01		
G1, G2, G3	33,37	0,19		
G4, G5, G6	34,31	0,43		
G7, G8, G9	26,98	0,04		
G10, G11, G12	26,77	0,59		

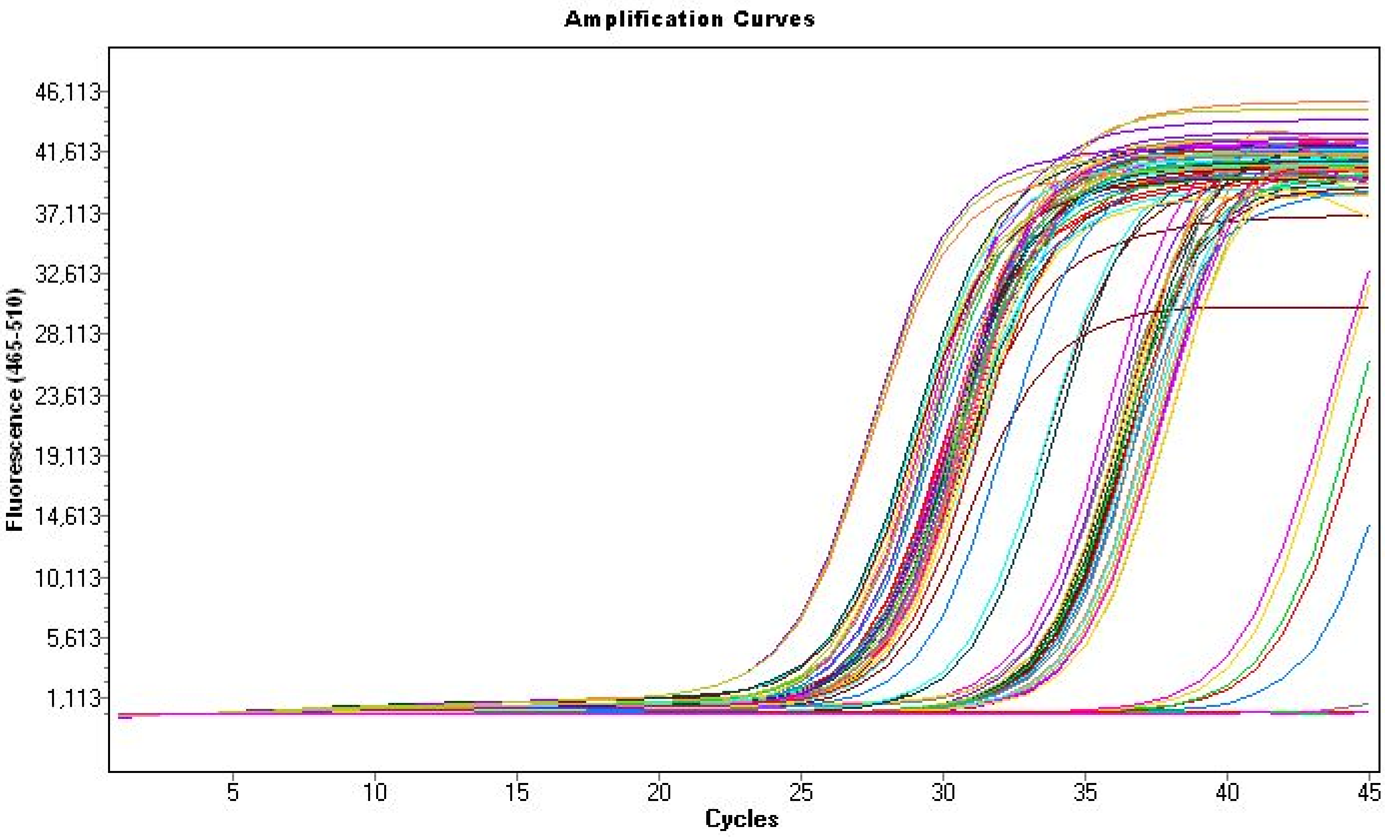
Amplification Curves
46,113
41,613
37,113
32,613
28,113
23,613
19,113
14,613
10,113
5,613
1,113
Fluorescence (465-510)
5
10
15
20
25
30
35
40
45
Cycles
