# Supplementary material for: MicroRNAs and Their Inhibition in Modulating SLC5A8 Expression in the Context of Papillary Thyroid Carcinoma
Source: Int J Mol Sci. 2025 Aug 15;26(16):7889. doi: 10.3390/ijms26167889 (PMC12386254; doi:10.3390/ijms26167889)
Supplement: Supplementary file 1 [file ijms-26-07889-s001.zip › ijms-3558049-supplementary/Manuscript data/Fig1 data/Data/2013-09-17 HPRT AIT 1711-10468.PDF]

Roche

Wojtek_2013-09-17_HPRT AIT 1711-10468

Programs

Program Name	pre-incubation		
Cycles	1	Analysis Mode	None

Target (°C)	Acquisition Mode	Hold (hh:mm:ss)	Ramp Rate (°C/s)	Acquisitions (per °C)	Sec Target (°C)	Step size (°C)	Step Delay (cycles)
95	None	00:10:00	4,40		0	0	0

Program Name	amplification		
Cycles	45	Analysis Mode	Quantification

Target (°C)	Acquisition Mode	Hold (hh:mm:ss)	Ramp Rate (°C/s)	Acquisitions (per °C)	Sec Target (°C)	Step size (°C)	Step Delay (cycles)
95	None	00:00:15	4,40		0	0	0
57	None	00:00:15	2,20		0	0	0
72	Single	00:00:15	4,40		0	0	0

Program Name	melting curve		
Cycles	1	Analysis Mode	Melting Curves

Target (°C)	Acquisition Mode	Hold (hh:mm:ss)	Ramp Rate (°C/s)	Acquisitions (per °C)	Sec Target (°C)	Step size (°C)	Step Delay (cycles)
95	None	00:00:05	4,40		0	0	0
65	None	00:01:00	2,20		0	0	0
97	Continuous		0,11	5	0	0	0

Program Name	cooling		
Cycles	1	Analysis Mode	None

Target (°C)	Acquisition Mode	Hold (hh:mm:ss)	Ramp Rate (°C/s)	Acquisitions (per °C)	Sec Target (°C)	Step size (°C)	Step Delay (cycles)
40	None	00:00:30	2,20		0	0	0

Tm Calling for All (Tm Calling)

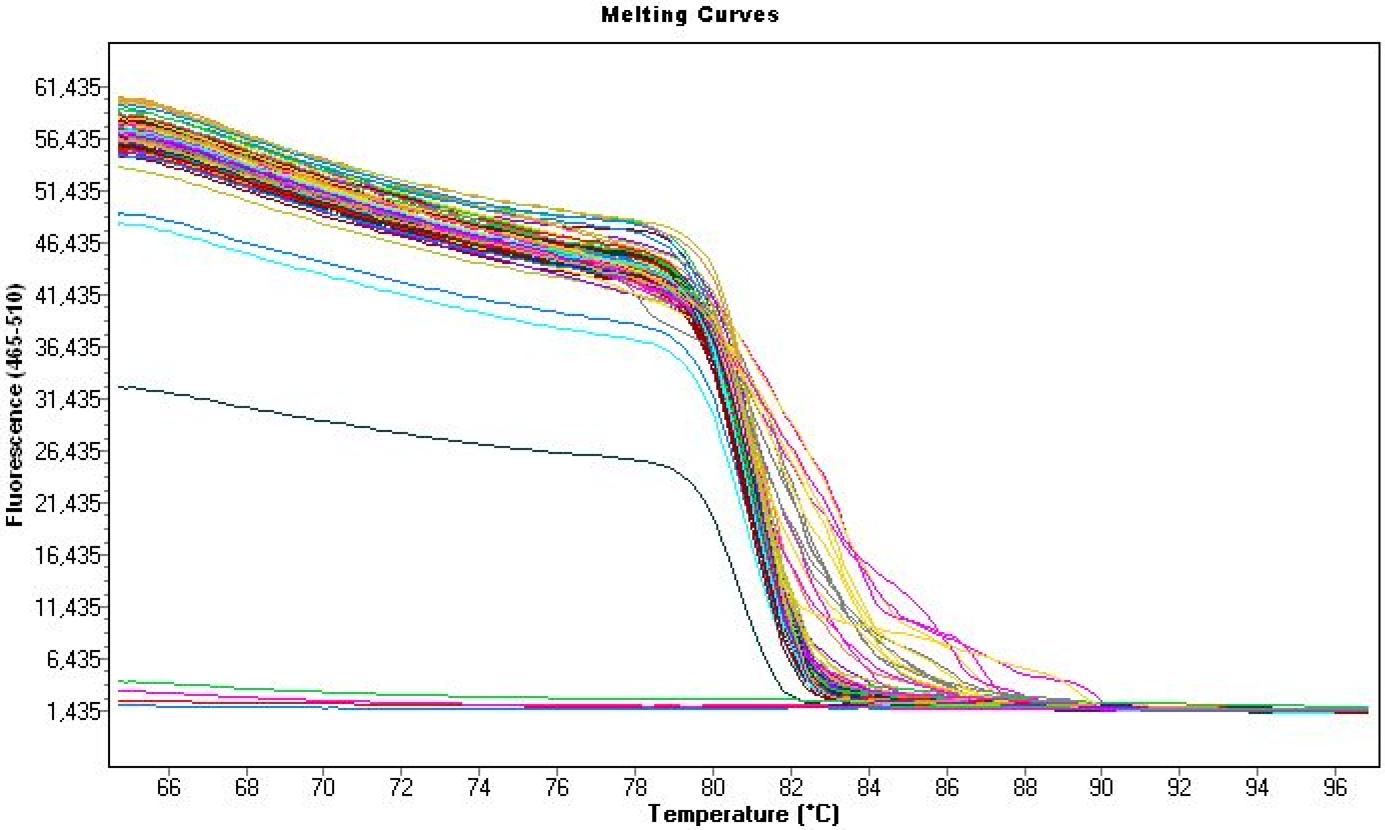

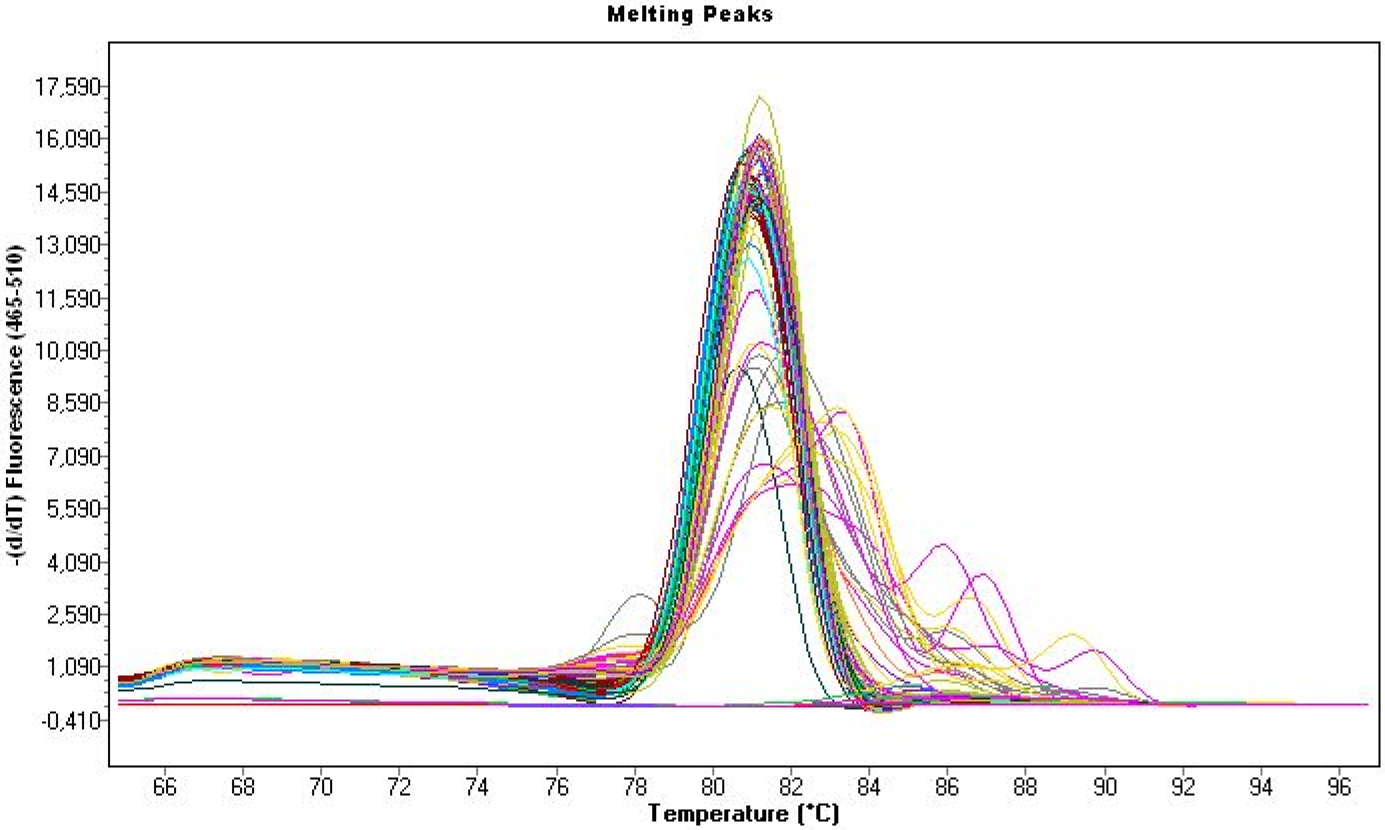

Abs Quant/2nd Derivative Max for All (Abs Quant/2nd Derivative Max)

Statistics

Samples	Mean Cp	Std Cp	Mean conc	Std conc
A1, A2, A3	26,00	0,03		
A4, A5, A6	32,72	0,67		
A7, A8, A9	26,96	0,05		
A10, A11, A12	27,69	0,20		
B1, B2, B3	27,67	0,06		
B4, B5, B6	27,28	0,05		
B7, B8, B9	27,15	0,22		
B10, B11, B12	27,04	0,34		
C1, C2, C3	27,17	0,04		
C4, C5, C6	29,39	0,21		
C7, C8, C9	27,83	0,04		
C10, C11, C12	26,89	0,08		
D1, D2, D3	26,69	0,22		
D4, D5, D6	31,91	0,51		
D7, D8, D9	26,87	0,07		
D10, D11, D12	26,15	0,04		
E1, E2, E3	25,78	0,44		
E4, E5, E6	33,40	0,23		
E7, E8, E9	25,15	0,07		
E10, E11, E12	25,55	0,07		
F1, F2, F3	26,29	0,06		
F4, F5, F6	31,58	0,48		
F7, F8, F9	26,37	0,09		
F10, F11, F12	25,05	0,02		
G1, G2, G3	26,03	0,05		
G4, G5, G6	29,23	0,18		
G7, G8, G9	26,48	0,54		
G10, G11, G12	24,88	0,03		

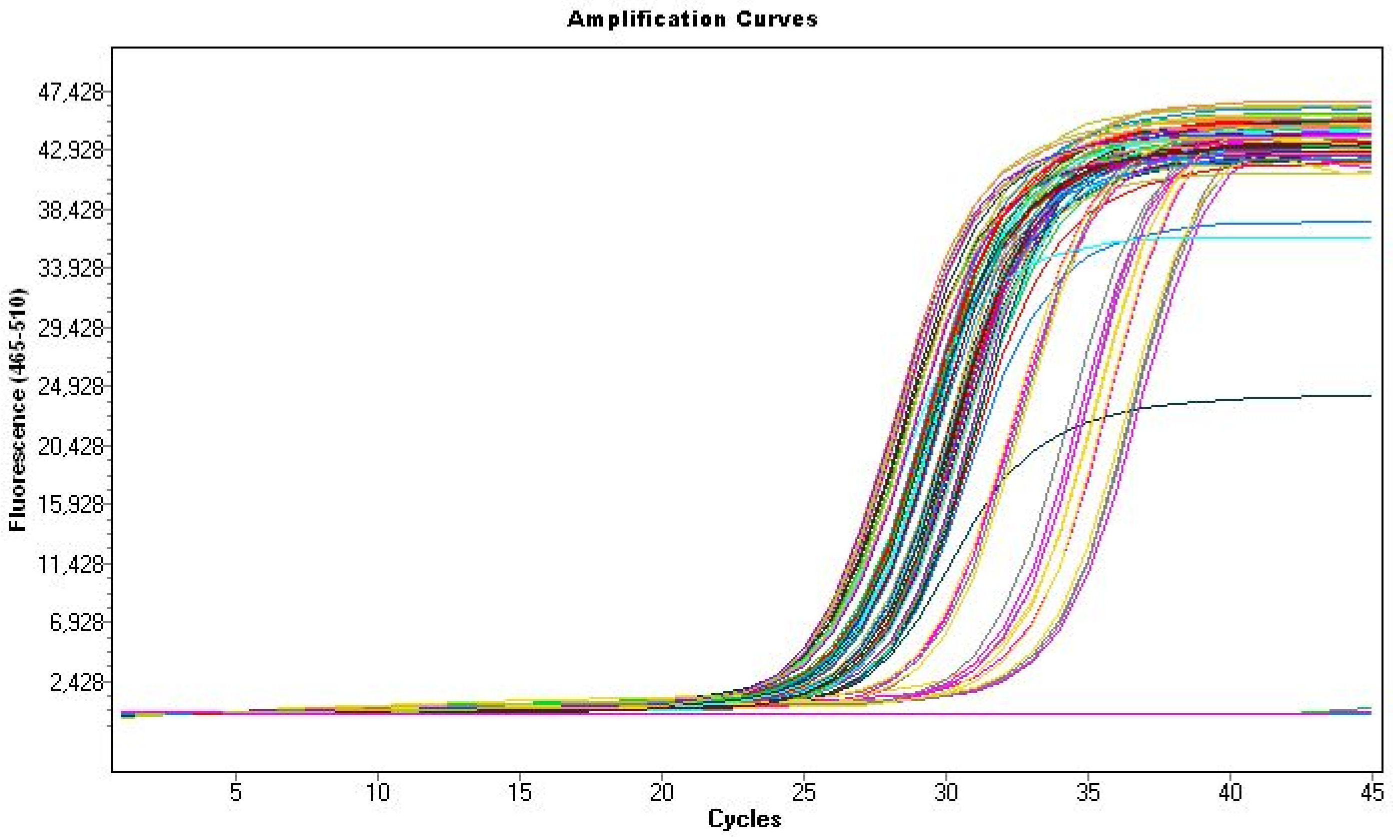
Amplification Curves
47,428
42,928
38,428
33,928
29,428
24,928
20,428
15,928
11,428
6,928
2,428
Fluorescence (465-510)
5
10
15
20
25
30
35
40
45
Cycles
