# Supplementary material for: MicroRNAs and Their Inhibition in Modulating SLC5A8 Expression in the Context of Papillary Thyroid Carcinoma
Source: Int J Mol Sci. 2025 Aug 15;26(16):7889. doi: 10.3390/ijms26167889 (PMC12386254; doi:10.3390/ijms26167889)
Supplement: Supplementary file 1 [file ijms-26-07889-s001.zip › ijms-3558049-supplementary/Manuscript data/Fig1 data/Data/2013-09-18 HPRT AIT 538N-10468N.PDF]

LightCycler® 480 Software
Report

Wojtek_2013-09-18_HPRT AIT 538N-10468N

Programs

Program Name	pre-incubation		
Cycles	1	Analysis Mode	None

Target (°C)	Acquisition Mode	Hold (hh:mm:ss)	Ramp Rate (°C/s)	Acquisitions (per °C)	Sec Target (°C)	Step size (°C)	Step Delay (cycles)
95	None	00:10:00	4,40		0	0	0

Program Name	amplification		
Cycles	45	Analysis Mode	Quantification

Target (°C)	Acquisition Mode	Hold (hh:mm:ss)	Ramp Rate (°C/s)	Acquisitions (per °C)	Sec Target (°C)	Step size (°C)	Step Delay (cycles)
95	None	00:00:15	4,40		0	0	0
57	None	00:00:15	2,20		0	0	0
72	Single	00:00:15	4,40		0	0	0

Program Name	melting curve		
Cycles	1	Analysis Mode	Melting Curves

Target (°C)	Acquisition Mode	Hold (hh:mm:ss)	Ramp Rate (°C/s)	Acquisitions (per °C)	Sec Target (°C)	Step size (°C)	Step Delay (cycles)
95	None	00:00:05	4,40		0	0	0
65	None	00:01:00	2,20		0	0	0
97	Continuous		0,11	5	0	0	0

Program Name	cooling		
Cycles	1	Analysis Mode	None

Target (°C)	Acquisition Mode	Hold (hh:mm:ss)	Ramp Rate (°C/s)	Acquisitions (per °C)	Sec Target (°C)	Step size (°C)	Step Delay (cycles)
40	None	00:00:30	2,20		0	0	0

Tm Calling for All (Tm Calling)

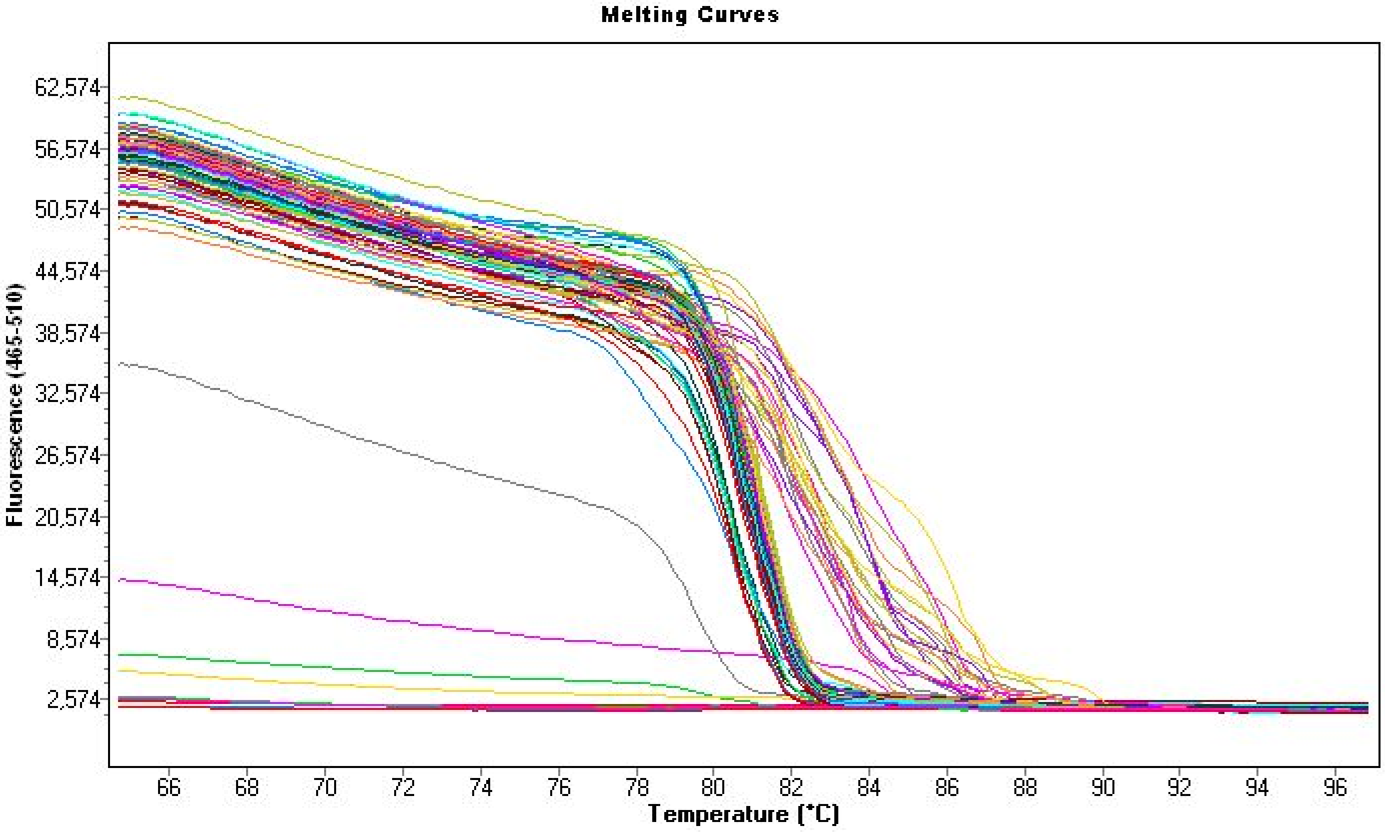

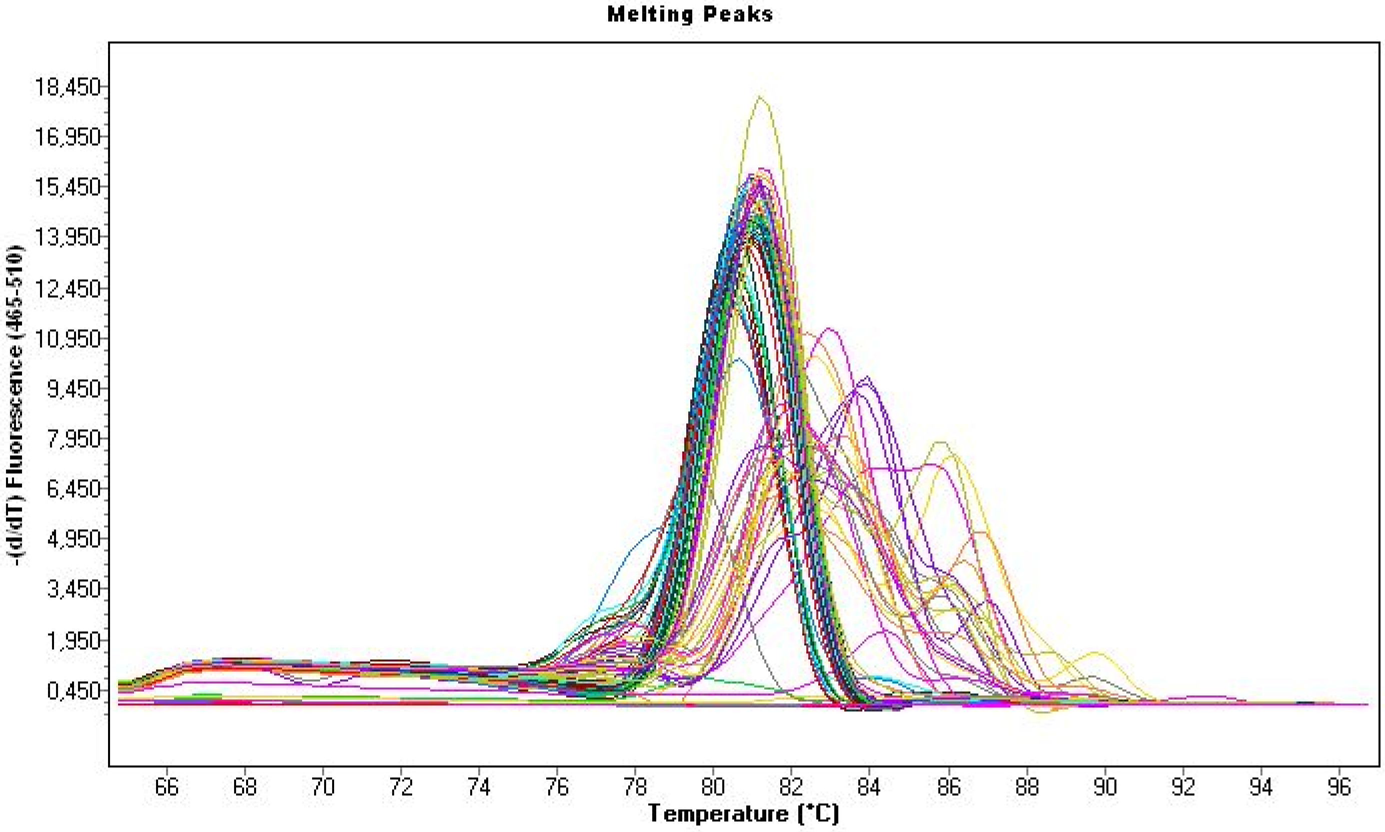

Abs Quant/2nd Derivative Max for All (Abs Quant/2nd Derivative Max)

Statistics

Samples	Mean Cp	Std Cp	Mean conc	Std conc
A1, A2, A3	27,66	0,04		
A4, A5, A6	33,74	0,36		
A7, A8, A9	30,12	0,34		
A10, A11, A12	34,60	0,39		
B1, B2, B3	39,24	1,07		
B4, B5, B6	40,00			
B7, B8, B9	32,89	0,64		
B10, B11, B12	33,47	0,66		
C1, C2, C3	26,93	0,04		
C4, C5, C6	27,19	0,31		
C7, C8, C9	33,31	0,12		
C10, C11, C12	34,33	0,50		
D1, D2, D3	33,57	0,23		
D4, D5, D6	34,43	0,44		
D7, D8, D9	27,22	0,67		
D10, D11, D12	26,92	0,09		
E1, E2, E3	27,58	0,26		
E4, E5, E6	26,49	0,04		
E7, E8, E9	27,30	0,30		
E10, E11, E12	26,66	0,49		
F1, F2, F3	25,85	0,23		
F4, F5, F6	32,44	0,25		
F7, F8, F9	26,59	0,06		
F10, F11, F12	31,32	0,12		
G1, G2, G3	25,93	0,73		
G4, G5, G6	33,06	0,47		
G7, G8, G9	25,70	0,24		
G10, G11, G12	28,84	0,16		

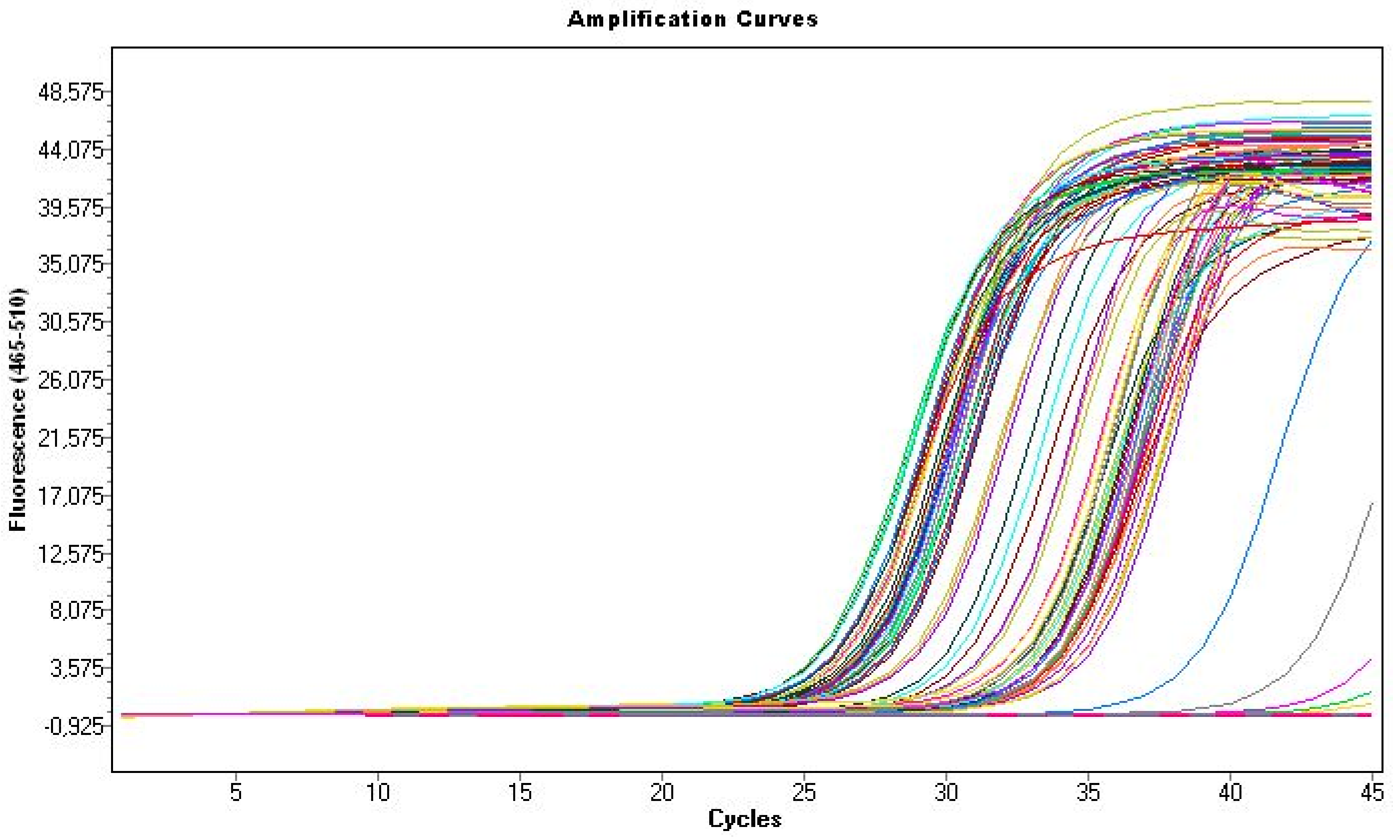
Amplification Curves
Fluorescence (465-510)
48,575
44,075
39,575
35,075
30,575
26,075
21,575
17,075
12,575
8,075
3,575
-0,925
5
10
15
20
25
30
35
40
45
Cycles
