# Supplementary material for: MicroRNAs and Their Inhibition in Modulating SLC5A8 Expression in the Context of Papillary Thyroid Carcinoma
Source: Int J Mol Sci. 2025 Aug 15;26(16):7889. doi: 10.3390/ijms26167889 (PMC12386254; doi:10.3390/ijms26167889)

# Wojtek_2013-09-19 HPRT AIT 1711T-10029T

## Programs

| Program Name | pre-incubation | | |
|---|---|---|---|
| Cycles | 1 | Analysis Mode | None |

| Target (°C) | Acquisition Mode | Hold (hh:mm:ss) | Ramp Rate (°C/s) | Acquisitions (per °C) | Sec Target (°C) | Step size (°C) | Step Delay (cycles) |
|---|---|---|---|---|---|---|---|
| 95 | None | 00:10:00 | 4,40 | | 0 | 0 | 0 |

| Program Name | amplification | | |
|---|---|---|---|
| Cycles | 45 | Analysis Mode | Quantification |

| Target (°C) | Acquisition Mode | Hold (hh:mm:ss) | Ramp Rate (°C/s) | Acquisitions (per °C) | Sec Target (°C) | Step size (°C) | Step Delay (cycles) |
|---|---|---|---|---|---|---|---|
| 95 | None | 00:00:15 | 4,40 | | 0 | 0 | 0 |
| 57 | None | 00:00:15 | 2,20 | | 0 | 0 | 0 |
| 72 | Single | 00:00:15 | 4,40 | | 0 | 0 | 0 |

| Program Name | melting curve | | |
|---|---|---|---|
| Cycles | 1 | Analysis Mode | Melting Curves |

| Target (°C) | Acquisition Mode | Hold (hh:mm:ss) | Ramp Rate (°C/s) | Acquisitions (per °C) | Sec Target (°C) | Step size (°C) | Step Delay (cycles) |
|---|---|---|---|---|---|---|---|
| 95 | None | 00:00:05 | 4,40 | | 0 | 0 | 0 |
| 65 | None | 00:01:00 | 2,20 | | 0 | 0 | 0 |
| 97 | Continuous | | 0,11 | 5 | 0 | 0 | 0 |

| Program Name | cooling | | |
|---|---|---|---|
| Cycles | 1 | Analysis Mode | None |

| Target (°C) | Acquisition Mode | Hold (hh:mm:ss) | Ramp Rate (°C/s) | Acquisitions (per °C) | Sec Target (°C) | Step size (°C) | Step Delay (cycles) |
|---|---|---|---|---|---|---|---|
| 40 | None | 00:00:30 | 2,20 | | 0 | 0 | 0 |

## Tm Calling for All (Tm Calling)

**Melting Curves**

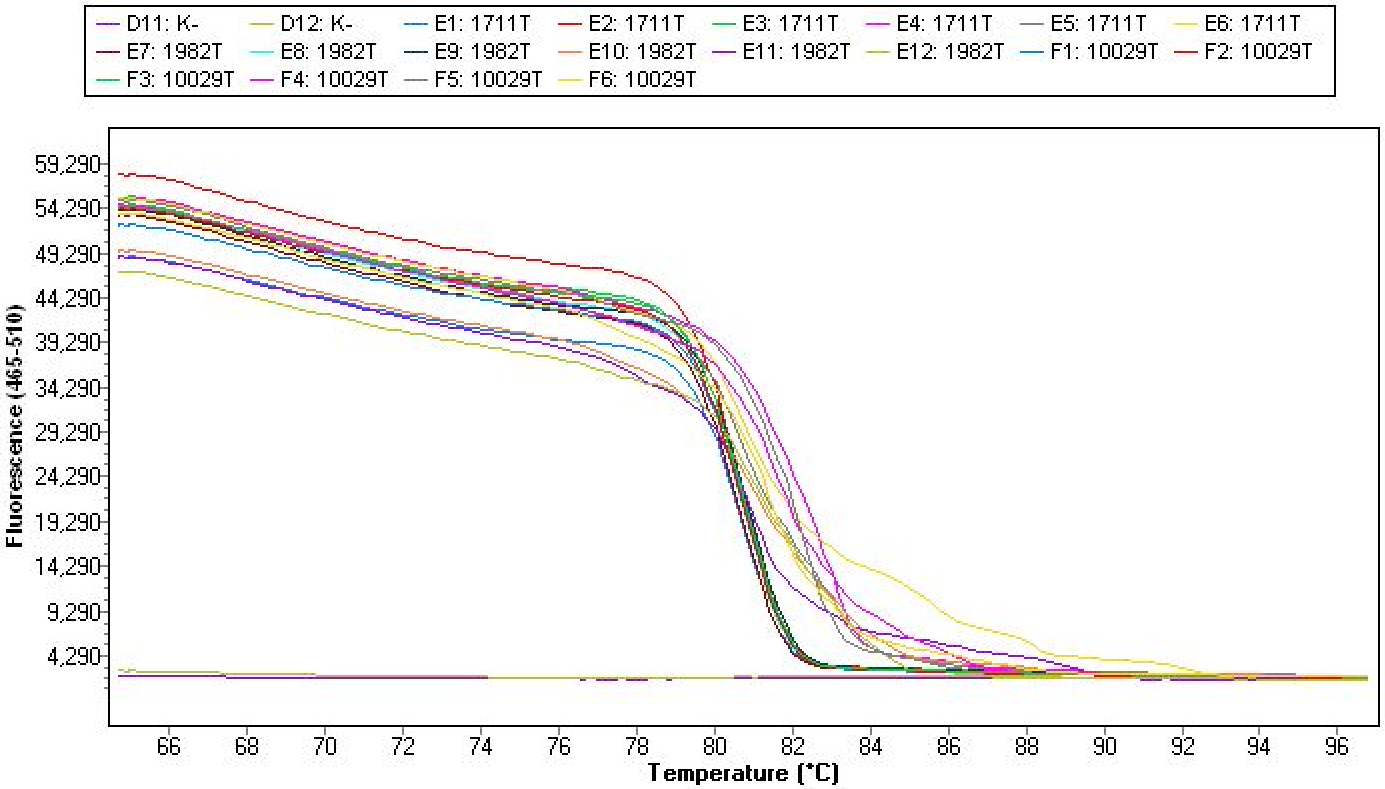


**Melting Peaks**

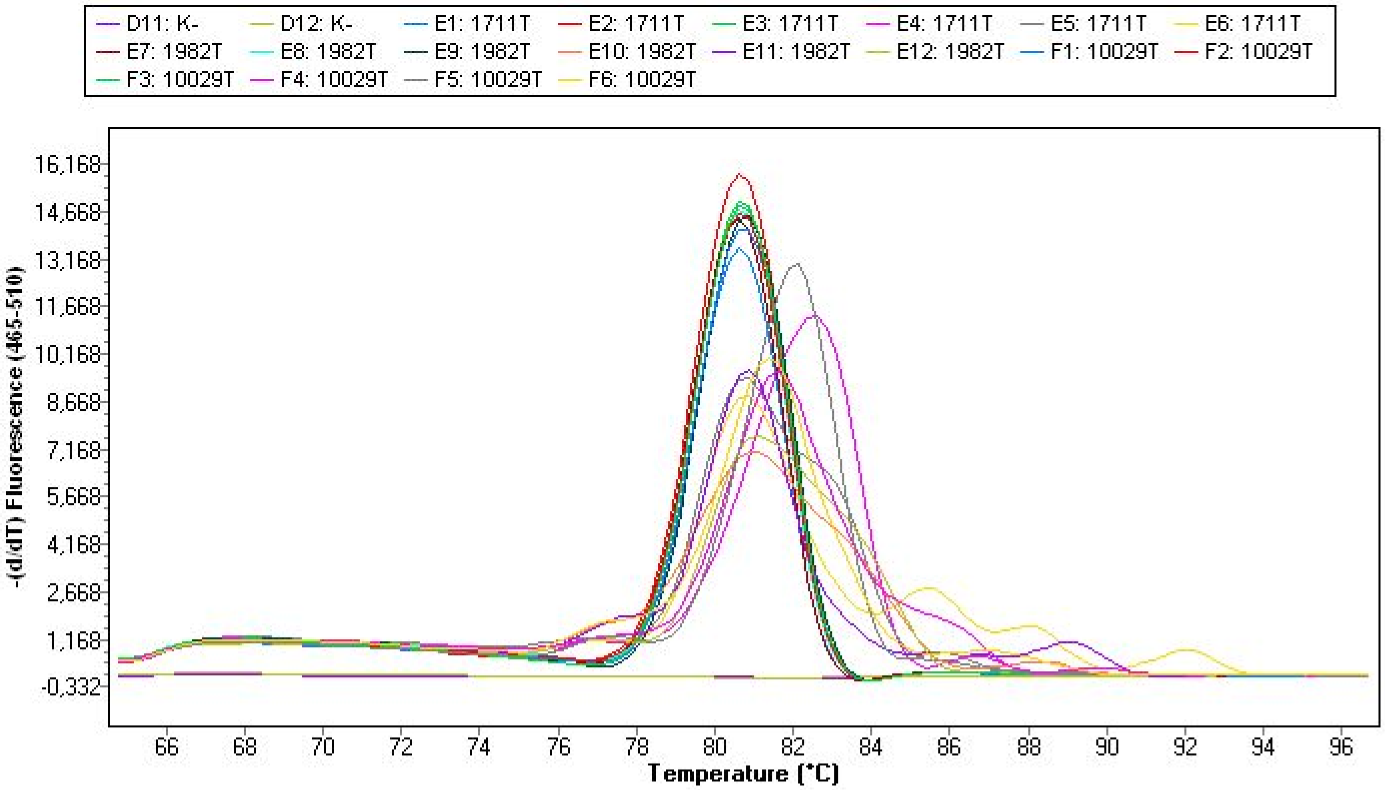


## Abs Quant/2nd Derivative Max for All (Abs Quant/2nd Derivative Max)

### Statistics

| Samples | Mean Cp | Std Cp | Mean conc | Std conc |
|---|---|---|---|---|
| E1, E2, E3 | 25,92 | 0,03 | | |
| E4, E5, E6 | 34,27 | 0,81 | | |
| E7, E8, E9 | 26,99 | 0,10 | | |
| E10, E11, E12 | 32,70 | 0,55 | | |
| F1, F2, F3 | 26,07 | 0,05 | | |
| F4, F5, F6 | 35,14 | 0,23 | | |

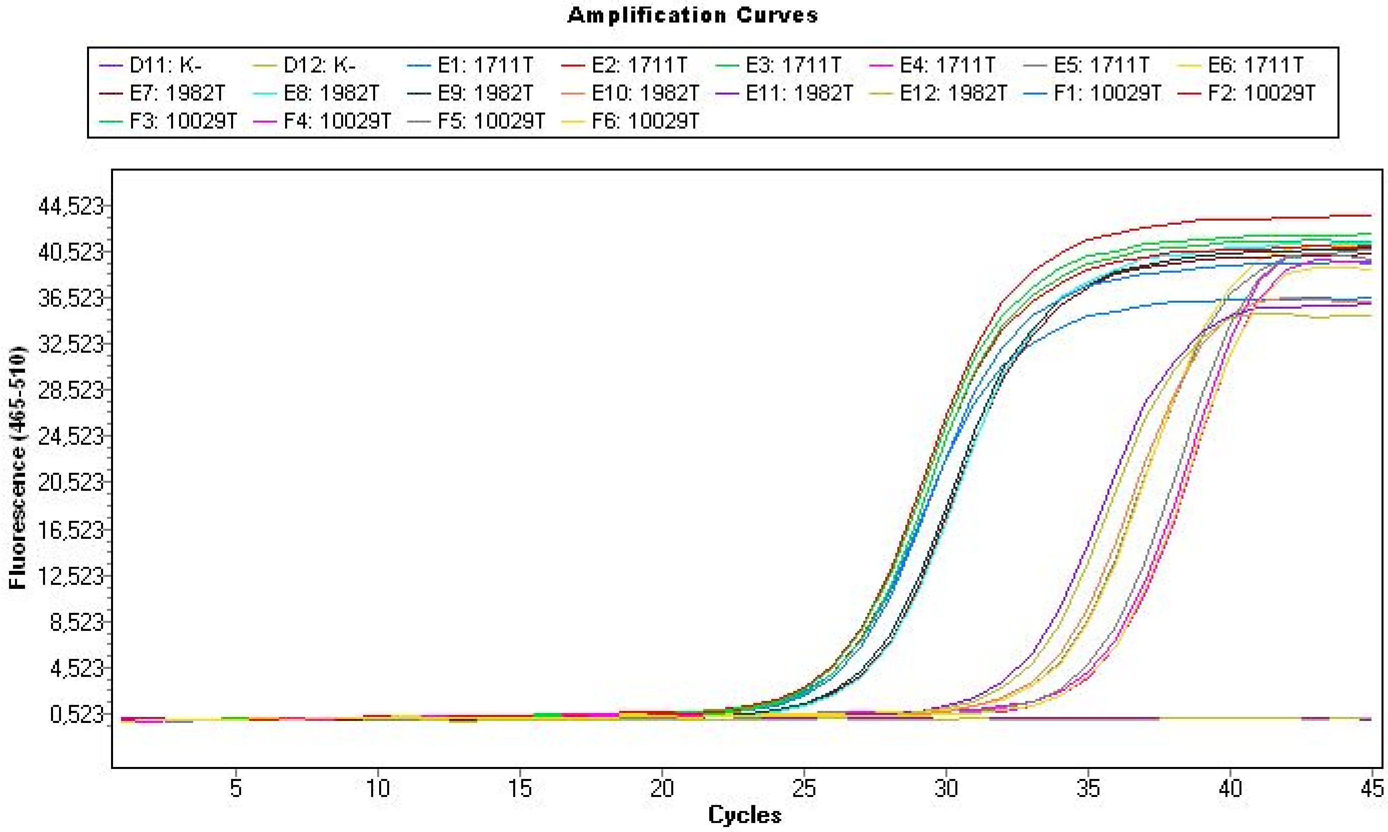

Supplement: Supplementary file 1 [file ijms-26-07889-s001.zip › ijms-3558049-supplementary/Manuscript data/Fig1 data/Data/2013-09-19 HPRT AIT 1711T-10029T.PDF]
