# Supplementary material for: MicroRNAs and Their Inhibition in Modulating SLC5A8 Expression in the Context of Papillary Thyroid Carcinoma
Source: Int J Mol Sci. 2025 Aug 15;26(16):7889. doi: 10.3390/ijms26167889 (PMC12386254; doi:10.3390/ijms26167889)

Wojtek_2013-09-20 HPRT AIT 1711T

Programs

Program Name	pre-incubation		
Cycles	1	Analysis Mode	None

Target (°C)	Acquisition Mode	Hold (hh:mm:ss)	Ramp Rate (°C/s)	Acquisitions (per °C)	Sec Target (°C)	Step size (°C)	Step Delay (cycles)
95	None	00:10:00	4,40		0	0	0

Program Name	amplification		
Cycles	45	Analysis Mode	Quantification

Target (°C)	Acquisition Mode	Hold (hh:mm:ss)	Ramp Rate (°C/s)	Acquisitions (per °C)	Sec Target (°C)	Step size (°C)	Step Delay (cycles)
95	None	00:00:15	4,40		0	0	0
57	None	00:00:15	2,20		0	0	0
72	Single	00:00:15	4,40		0	0	0

Program Name	melting curve		
Cycles	1	Analysis Mode	Melting Curves

Target (°C)	Acquisition Mode	Hold (hh:mm:ss)	Ramp Rate (°C/s)	Acquisitions (per °C)	Sec Target (°C)	Step size (°C)	Step Delay (cycles)
95	None	00:00:05	4,40		0	0	0
65	None	00:01:00	2,20		0	0	0
97	Continuous		0,11	5	0	0	0

Program Name	cooling		
Cycles	1	Analysis Mode	None

Target (°C)	Acquisition Mode	Hold (hh:mm:ss)	Ramp Rate (°C/s)	Acquisitions (per °C)	Sec Target (°C)	Step size (°C)	Step Delay (cycles)
40	None	00:00:30	2,20		0	0	0

Tm Calling for All (Tm Calling)

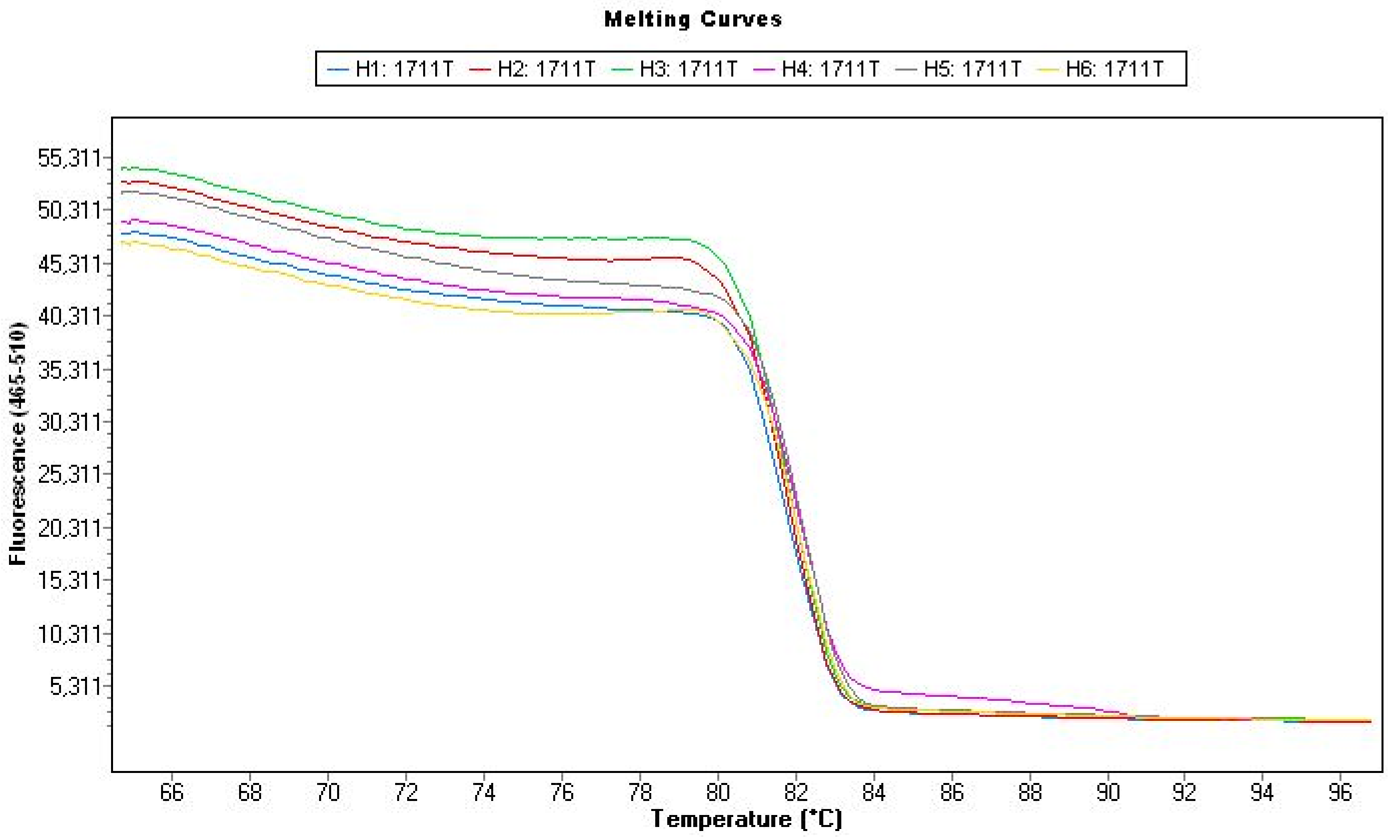

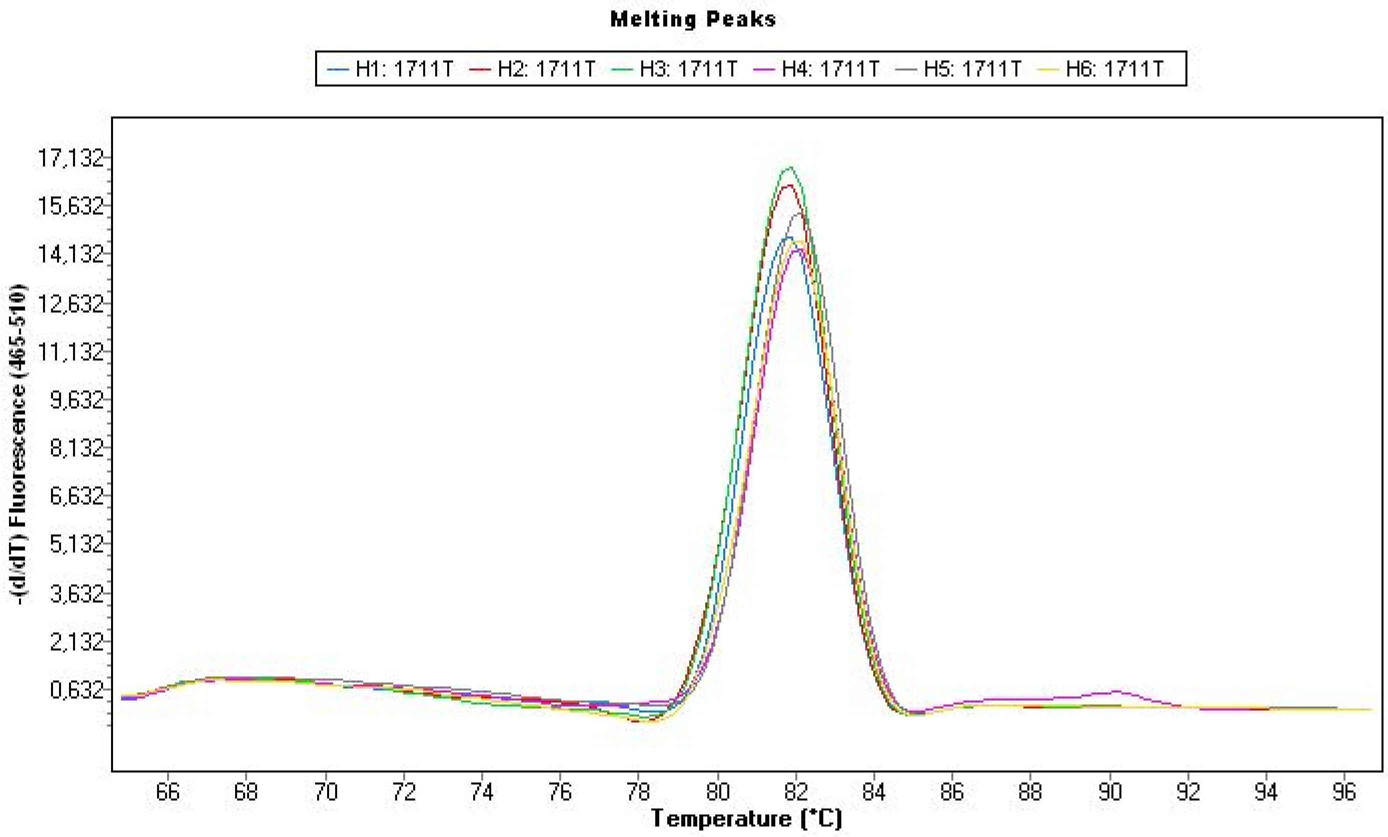

Abs Quant/2nd Derivative Max for All (Abs Quant/2nd Derivative Max)

Results

Inc	Pos	Name	Type	CP	Concentration	Standard	Status
☑	H1	1711T	Unknown	24,81			
☑	H2	1711T	Unknown	24,86			
☑	H3	1711T	Unknown	24,95			
☑	H4	1711T	Unknown	33,91			
☑	H5	1711T	Unknown	34,08			
☑	H6	1711T	Unknown	34,51			

Statistics

Samples	Mean Cp	Std Cp	Mean conc	Std conc
H1, H2, H3	24,87	0,07		
H4, H5, H6	34,17	0,31		

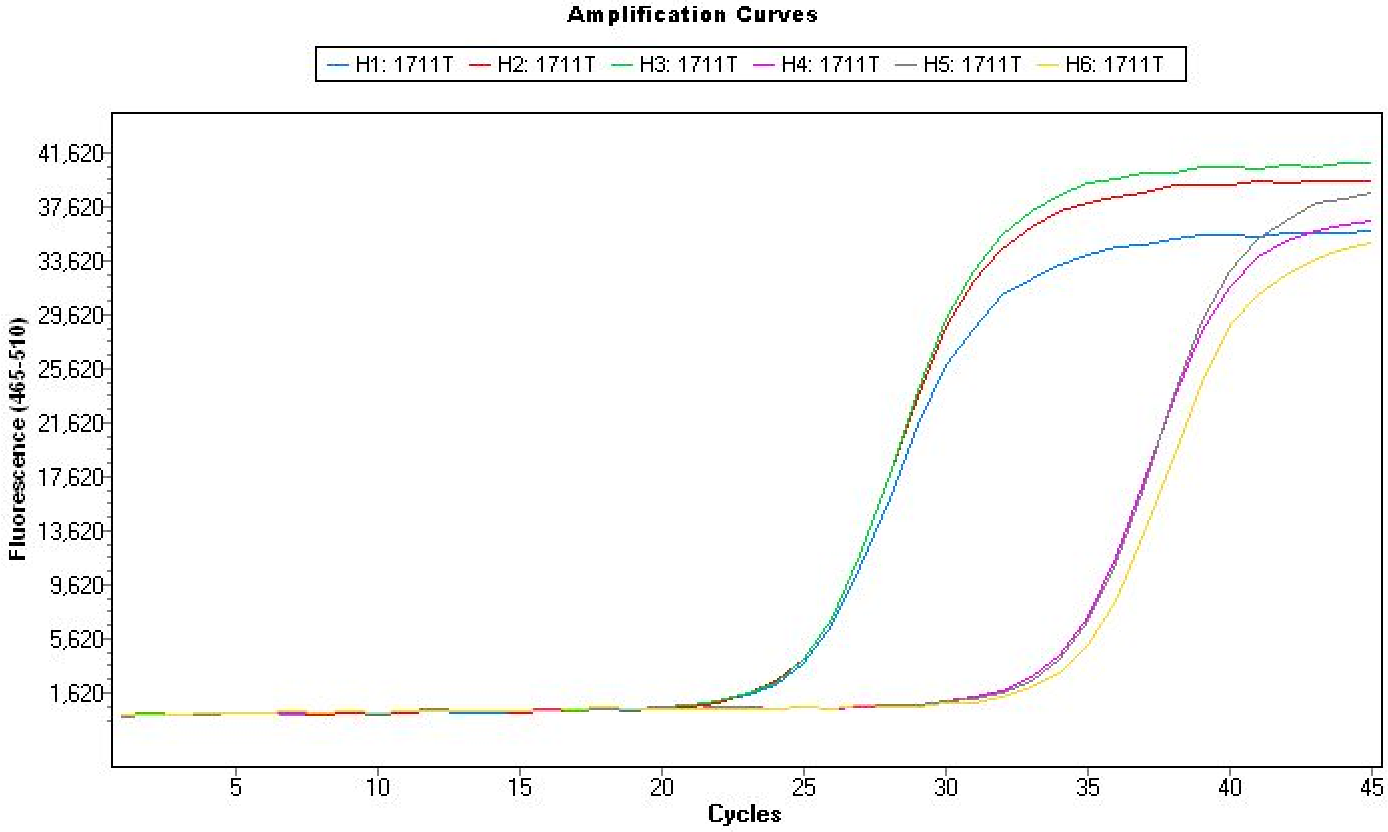

Supplement: Supplementary file 1 [file ijms-26-07889-s001.zip › ijms-3558049-supplementary/Manuscript data/Fig1 data/Data/2013-09-20 HPRT AIT 1711T.PDF]
