# Supplementary material for: MicroRNAs and Their Inhibition in Modulating SLC5A8 Expression in the Context of Papillary Thyroid Carcinoma
Source: Int J Mol Sci. 2025 Aug 15;26(16):7889. doi: 10.3390/ijms26167889 (PMC12386254; doi:10.3390/ijms26167889)
Supplement: Supplementary file 1 [file ijms-26-07889-s001.zip › ijms-3558049-supplementary/Manuscript data/Fig1 data/Data/2013-11-19 HPRT AIT 10001143.PDF]

Roche

Wojtek_2013-11-19 HPRT AIT 10001143

Programs

Program Name	pre-incubation		
Cycles	1	Analysis Mode	None

Target (°C)	Acquisition Mode	Hold (hh:mm:ss)	Ramp Rate (°C/s)	Acquisitions (per °C)	Sec Target (°C)	Step size (°C)	Step Delay (cycles)
95	None	00:10:00	4,40		0	0	0

Program Name	amplification		
Cycles	45	Analysis Mode	Quantification

Target (°C)	Acquisition Mode	Hold (hh:mm:ss)	Ramp Rate (°C/s)	Acquisitions (per °C)	Sec Target (°C)	Step size (°C)	Step Delay (cycles)
95	None	00:00:15	4,40		0	0	0
57	None	00:00:15	2,20		0	0	0
72	Single	00:00:15	4,40		0	0	0

Program Name	melting curve		
Cycles	1	Analysis Mode	Melting Curves

Target (°C)	Acquisition Mode	Hold (hh:mm:ss)	Ramp Rate (°C/s)	Acquisitions (per °C)	Sec Target (°C)	Step size (°C)	Step Delay (cycles)
95	None	00:00:05	4,40		0	0	0
65	None	00:01:00	2,20		0	0	0
97	Continuous		0,11	5	0	0	0

Program Name	cooling		
Cycles	1	Analysis Mode	None

Target (°C)	Acquisition Mode	Hold (hh:mm:ss)	Ramp Rate (°C/s)	Acquisitions (per °C)	Sec Target (°C)	Step size (°C)	Step Delay (cycles)
40	None	00:00:30	2,20		0	0	0

Tm Calling for All (Tm Calling)

Melting Curves

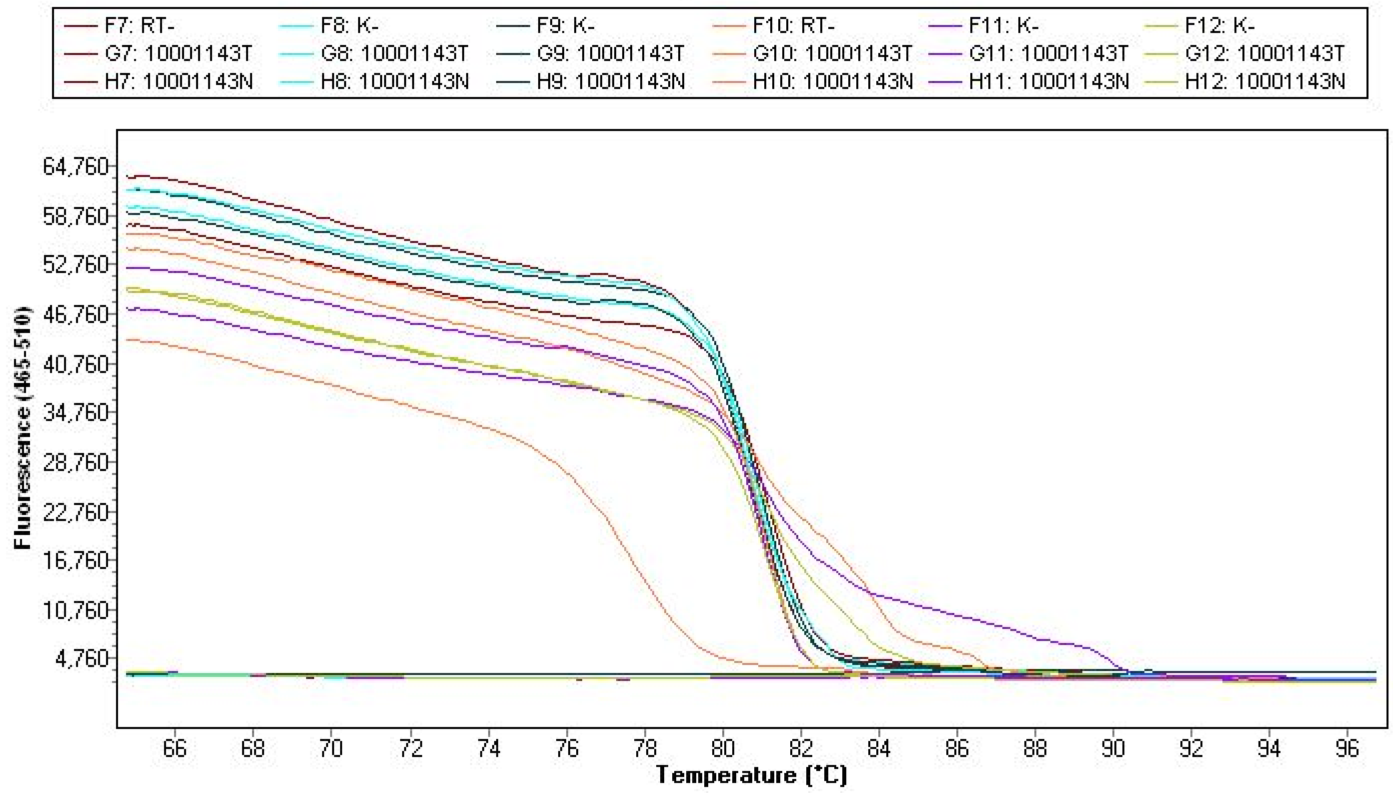

Melting Peaks

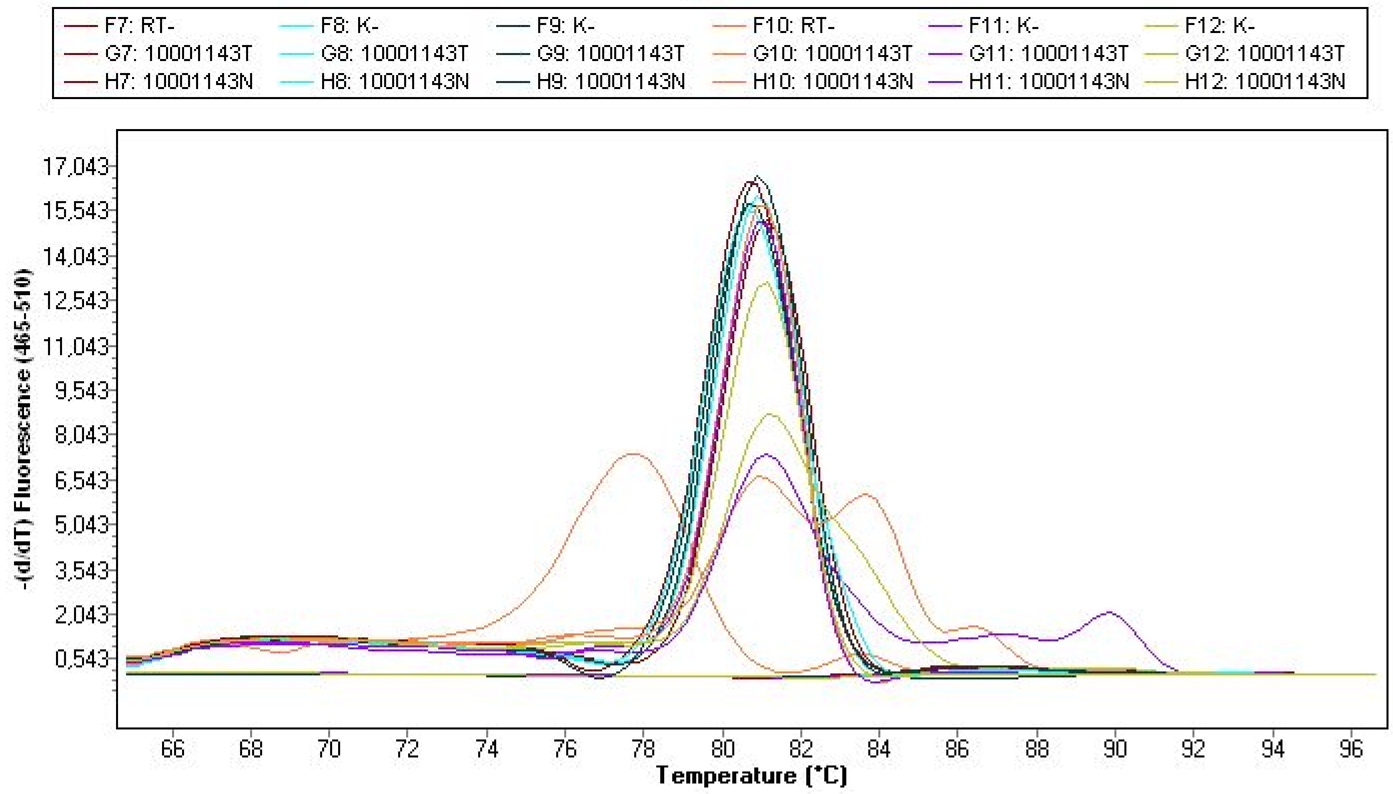

Abs Quant/2nd Derivative Max for All (Abs Quant/2nd Derivative Max)

Results

Inc	Pos	Name	Type	CP	Concentration	Standard	Status
☑	F7	RT-	Negative Control				
☑	F8	K-	Negative Control				
☑	F9	K-	Negative Control				
☑	F10	RT-	Negative Control	37,19			
☑	F11	K-	Negative Control				
☑	F12	K-	Negative Control				
☑	G7	10001143T	Unknown	24,05			
☑	G8	10001143T	Unknown	24,61			
☑	G9	10001143T	Unknown	24,85			
☑	G10	10001143T	Unknown	33,47			
☑	G11	10001143T	Unknown	32,45			
☑	G12	10001143T	Unknown	33,93			
☑	H7	10001143N	Unknown	25,66			
☑	H8	10001143N	Unknown	25,75			
☑	H9	10001143N	Unknown	25,55			
☑	H10	10001143N	Unknown	26,83			
☑	H11	10001143N	Unknown	26,79			
☑	H12	10001143N	Unknown	27,03			

Statistics

Samples	Mean Cp	Std Cp	Mean conc	Std conc
F8, F9				
F11, F12				
G7, G8, G9	24,50	0,41		
G10, G11, G12	33,28	0,75		
H7, H8, H9	25,65	0,10		
H10, H11, H12	26,88	0,13		

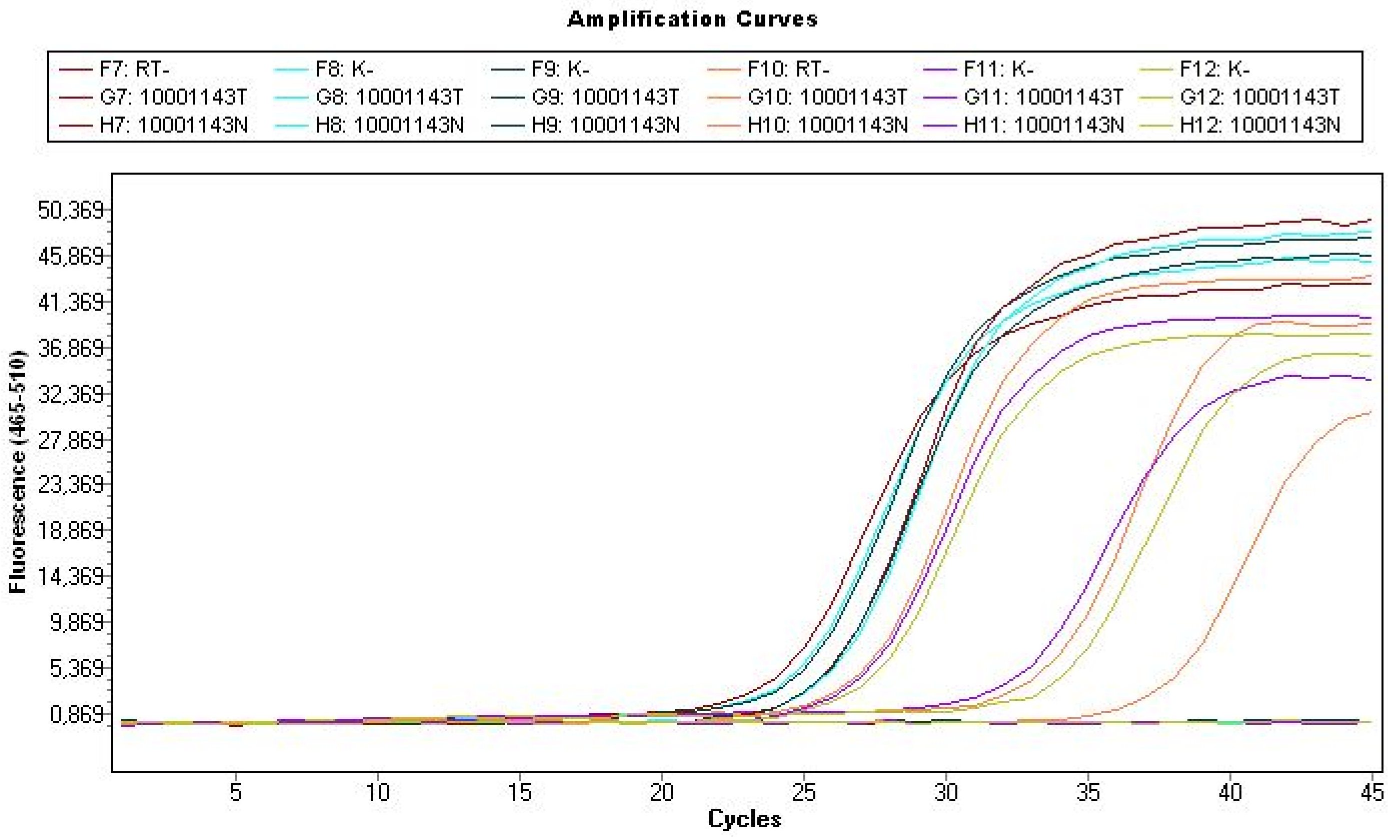
Amplification Curves
F7: RT-
F8: K-
F9: K-
F10: RT-
F11: K-
F12: K-
G7: 10001143T
G8: 10001143T
G9: 10001143T
G10: 10001143T
G11: 10001143T
G12: 10001143T
H7: 10001143N
H8: 10001143N
H9: 10001143N
H10: 10001143N
H11: 10001143N
H12: 10001143N
Fluorescence (465-510)
50,369
45,869
41,369
36,869
32,369
27,869
23,369
18,869
14,369
9,869
5,369
0,869
5
10
15
20
25
30
35
40
45
Cycles
