# Supplementary material for: MicroRNAs and Their Inhibition in Modulating SLC5A8 Expression in the Context of Papillary Thyroid Carcinoma
Source: Int J Mol Sci. 2025 Aug 15;26(16):7889. doi: 10.3390/ijms26167889 (PMC12386254; doi:10.3390/ijms26167889)
Supplement: Supplementary file 1 [file ijms-26-07889-s001.zip › ijms-3558049-supplementary/Manuscript data/Fig1 data/Data/probki_1.PDF]

Roche

Abs Quant/2nd Derivative Max for All Samples (Abs Quant/2nd Derivative Max)

Results

Inc	Pos	Name	Type	CP	Concentration	Standard	Status
☑	A1	Sample 1	Unknown				
☑	A2	Sample 2	Unknown				
☑	A3	Sample 3	Unknown				
☑	A4	probki	Unknown	33,05			
☑	A5	probki	Unknown	33,16			
☑	A6	probki	Unknown	33,86			
☑	A7	Sample 7	Unknown				
☑	A8	Sample 8	Unknown				
☑	A9	Sample 9	Unknown				
☑	A10	Sample 10	Unknown				
☑	A11	Sample 11	Unknown				
☑	A12	Sample 12	Unknown				
☑	B1	Sample 13	Unknown				
☑	B2	Sample 14	Unknown				
☑	B3	Sample 15	Unknown				
☑	B4	probki	Unknown	27,04			
☑	B5	probki	Unknown	27,19			
☑	B6	probki	Unknown	27,12			
☑	B7	Sample 19	Unknown				
☑	B8	Sample 20	Unknown				
☑	B9	Sample 21	Unknown				
☑	B10	Sample 22	Unknown				
☑	B11	Sample 23	Unknown				
☑	B12	Sample 24	Unknown				
☑	C1	Sample 25	Unknown				
☑	C2	Sample 26	Unknown				
☑	C3	Sample 27	Unknown				
☑	C4	probki	Unknown	26,21			
☑	C5	probki	Unknown	26,28			
☑	C6	probki	Unknown	26,70			
☑	C7	Sample 31	Unknown				
☑	C8	Sample 32	Unknown				
☑	C9	Sample 33	Unknown				

Results

Inc	Pos	Name	Type	CP	Concentration	Standard	Status
☑	C10	Sample 34	Unknown				
☑	C11	Sample 35	Unknown				
☑	C12	Sample 36	Unknown				
☑	D1	Sample 37	Unknown				
☑	D2	Sample 38	Unknown				
☑	D3	Sample 39	Unknown				
☑	D4	probki	Unknown	25,72			
☑	D5	probki	Unknown	25,79			
☑	D6	probki	Unknown	26,18			
☑	D7	Sample 43	Unknown				
☑	D8	Sample 44	Unknown				
☑	D9	Sample 45	Unknown				
☑	D10	Sample 46	Unknown				
☑	D11	Sample 47	Unknown				
☑	D12	Sample 48	Unknown				
☑	E1	Sample 49	Unknown				
☑	E2	Sample 50	Unknown				
☑	E3	Sample 51	Unknown				
☑	E4	probki	Unknown	29,35			
☑	E5	probki	Unknown	29,11			
☑	E6	probki	Unknown	29,44			
☑	E7	Sample 55	Unknown				
☑	E8	Sample 56	Unknown				
☑	E9	Sample 57	Unknown				
☑	E10	Sample 58	Unknown				
☑	E11	Sample 59	Unknown				
☑	E12	Sample 60	Unknown				
☑	F1	Sample 61	Unknown				
☑	F2	Sample 62	Unknown				
☑	F3	Sample 63	Unknown				
☑	F4	probki	Unknown	25,43			
☑	F5	probki	Unknown	25,85			
☑	F6	probki	Unknown	26,05			
☑	F7	Sample 67	Unknown				
☑	F8	Sample 68	Unknown				
☑	F9	Sample 69	Unknown				
☑	F10	Sample 70	Unknown				

Results

Inc	Pos	Name	Type	CP	Concentration	Standard	Status
☑	F11	Sample 71	Unknown				
☑	F12	Sample 72	Unknown				
☑	G1	Sample 73	Unknown	30,66			
☑	G2	Sample 74	Unknown	31,34			
☑	G3	Sample 75	Unknown	12,28			
☑	G4	probki	Unknown	26,12			
☑	G5	probki	Unknown	26,15			
☑	G6	probki	Unknown	26,92			
☑	G7	Sample 79	Unknown				
☑	G8	Sample 80	Unknown				
☑	G9	Sample 81	Unknown				
☑	G10	Sample 82	Unknown				
☑	G11	Sample 83	Unknown				
☑	G12	Sample 84	Unknown				
☑	H1	k-	Unknown				
☑	H2	k-	Unknown				
☑	H3	k-	Unknown				
☑	H4	probki	Unknown	26,70			
☑	H5	probki	Unknown	26,73			
☑	H6	probki	Unknown	26,89			
☑	H7	Sample 91	Unknown				
☑	H8	Sample 92	Unknown				
☑	H9	Sample 93	Unknown				
☑	H10	Sample 94	Unknown				
☑	H11	Sample 95	Unknown				
☑	H12	Sample 96	Unknown				
