# Supplementary material for: MicroRNAs and Their Inhibition in Modulating SLC5A8 Expression in the Context of Papillary Thyroid Carcinoma
Source: Int J Mol Sci. 2025 Aug 15;26(16):7889. doi: 10.3390/ijms26167889 (PMC12386254; doi:10.3390/ijms26167889)
Supplement: Supplementary file 1 [file ijms-26-07889-s001.zip › ijms-3558049-supplementary/Manuscript data/Fig1 data/Data/probki_2.PDF]

Roche

Abs Quant/2nd Derivative Max for All Samples (Abs Quant/2nd Derivative Max)

Results

Inc	Pos	Name	Type	CP	Concentration	Standard	Status
☑	A1	probki	Unknown	29,35			
☑	A2	probki	Unknown	28,81			
☑	A3	probki	Unknown	29,03			
☑	A4	probki	Unknown	30,79			
☑	A5	probki	Unknown	30,20			
☑	A6	probki	Unknown	30,74			
☑	A7	Sample 7	Unknown				
☑	A8	Sample 8	Unknown	24,16			
☑	A9	Sample 9	Unknown				
☑	A10	Sample 10	Unknown				
☑	A11	Sample 11	Unknown	15,74			
☑	A12	Sample 12	Unknown				
☑	B1	probki	Unknown	32,07			
☑	B2	probki	Unknown	32,33			
☑	B3	probki	Unknown	32,08			
☑	B4	probki	Unknown	27,83			
☑	B5	probki	Unknown	27,82			
☑	B6	probki	Unknown	27,88			
☑	B7	Sample 19	Unknown				
☑	B8	Sample 20	Unknown	15,81			
☑	B9	Sample 21	Unknown	19,48			
☑	B10	Sample 22	Unknown	19,44			?
☑	B11	Sample 23	Unknown				
☑	B12	Sample 24	Unknown				
☑	C1	probki	Unknown	30,55			
☑	C2	probki	Unknown	30,77			
☑	C3	probki	Unknown	30,66			
☑	C4	probki	Unknown	32,91			
☑	C5	probki	Unknown	32,50			
☑	C6	probki	Unknown	32,81			
☑	C7	Sample 31	Unknown	17,61			?
☑	C8	Sample 32	Unknown	18,10			

? - Detector Call uncertain

Results

Inc	Pos	Name	Type	CP	Concentration	Standard	Status
☑	C9	Sample 33	Unknown				
☑	C10	Sample 34	Unknown				
☑	C11	Sample 35	Unknown				
☑	C12	Sample 36	Unknown				
☑	D1	probki	Unknown	30,41			
☑	D2	probki	Unknown	30,75			
☑	D3	probki	Unknown	30,27			
☑	D4	probki	Unknown	26,32			
☑	D5	probki	Unknown	26,40			
☑	D6	probki	Unknown	26,57			
☑	D7	Sample 43	Unknown				
☑	D8	Sample 44	Unknown				
☑	D9	Sample 45	Unknown				
☑	D10	Sample 46	Unknown				
☑	D11	Sample 47	Unknown	18,84			?
☑	D12	Sample 48	Unknown	19,16			
☑	E1	probki	Unknown	29,30			
☑	E2	probki	Unknown	28,71			
☑	E3	probki	Unknown	29,59			
☑	E4	probki	Unknown	34,05			
☑	E5	probki	Unknown	35,18			
☑	E6	probki	Unknown	35,51			
☑	E7	Sample 55	Unknown	26,39			
☑	E8	Sample 56	Unknown	15,45			
☑	E9	Sample 57	Unknown	17,06			
☑	E10	Sample 58	Unknown				
☑	E11	Sample 59	Unknown				
☑	E12	Sample 60	Unknown	17,09			?
☑	F1	probki	Unknown	30,92			
☑	F2	probki	Unknown	31,11			
☑	F3	probki	Unknown	30,87			
☑	F4	probki	Unknown	27,68			
☑	F5	probki	Unknown	27,67			
☑	F6	probki	Unknown	27,99			
☑	F7	Sample 67	Unknown	26,44			
☑	F8	Sample 68	Unknown	15,25			
☑	F9	Sample 69	Unknown	19,11			

? - Detector Call uncertain

Results

Inc	Pos	Name	Type	CP	Concentration	Standard	Status
☑	F10	Sample 70	Unknown	18,38			
☑	F11	Sample 71	Unknown	19,62			
☑	F12	Sample 72	Unknown				
☑	G1	probki	Unknown	26,13			
☑	G2	probki	Unknown	26,54			
☑	G3	probki	Unknown	26,65			
☑	G4	k-	Unknown				
☑	G5	k-	Unknown				
☑	G6	k-	Unknown				
☑	G7	Sample 79	Unknown				
☑	G8	Sample 80	Unknown	18,72			
☑	G9	Sample 81	Unknown	18,00			
☑	G10	Sample 82	Unknown				
☑	G11	Sample 83	Unknown	19,02			
☑	G12	Sample 84	Unknown	18,26			
☑	H1	probki	Unknown	28,94			
☑	H2	probki	Unknown	28,91			
☑	H3	probki	Unknown	29,16			
☑	H4	Sample 88	Unknown	18,72			
☑	H5	Sample 89	Unknown	27,52			
☑	H6	Sample 90	Unknown				
☑	H7	Sample 91	Unknown				
☑	H8	Sample 92	Unknown	18,62			
☑	H9	Sample 93	Unknown				
☑	H10	Sample 94	Unknown	19,07			
☑	H11	Sample 95	Unknown	17,96			
☑	H12	Sample 96	Unknown	19,82			?

? - Detector Call uncertain
