# Supplementary material for: MicroRNAs and Their Inhibition in Modulating SLC5A8 Expression in the Context of Papillary Thyroid Carcinoma
Source: Int J Mol Sci. 2025 Aug 15;26(16):7889. doi: 10.3390/ijms26167889 (PMC12386254; doi:10.3390/ijms26167889)
Supplement: Supplementary file 1 [file ijms-26-07889-s001.zip › ijms-3558049-supplementary/Manuscript data/Fig1 data/Data/probki_4.PDF]

Roche

Abs Quant/2nd Derivative Max for All Samples (Abs Quant/2nd Derivative Max)

Results

Inc	Pos	Name	Type	CP	Concentration	Standard	Status
☑	A1	1673T	Unknown	26,41			
☑	A2	1673T	Unknown	26,81			
☑	A3	1673T	Unknown				
☑	A4	k-	Unknown	35,50			
☑	A5	k-	Unknown				
☑	A6	k-	Unknown	36,26			
☑	A7	Sample 7	Unknown	21,09			
☑	A8	Sample 8	Unknown	24,54			
☑	A9	Sample 9	Unknown	21,39			
☑	A10	Sample 10	Unknown	25,86			
☑	A11	Sample 11	Unknown	38,36			
☑	A12	Sample 12	Unknown	32,84			
☑	B1	1673N	Unknown	27,20			
☑	B2	1673N	Unknown	26,27			
☑	B3	1673N	Unknown	26,99			
☑	B4	Sample 16	Unknown				
☑	B5	Sample 17	Unknown				
☑	B6	Sample 18	Unknown				
☑	B7	Sample 19	Unknown	23,16			
☑	B8	Sample 20	Unknown	39,36			
☑	B9	Sample 21	Unknown	22,37			
☑	B10	Sample 22	Unknown				
☑	B11	Sample 23	Unknown				
☑	B12	Sample 24	Unknown				
☑	C1	1680T	Unknown	31,57			
☑	C2	1680T	Unknown	32,11			
☑	C3	1680T	Unknown	31,70			
☑	C4	Sample 28	Unknown				
☑	C5	Sample 29	Unknown				
☑	C6	Sample 30	Unknown	29,45			
☑	C7	Sample 31	Unknown	23,64			
☑	C8	Sample 32	Unknown				
☑	C9	Sample 33	Unknown				

Results

Inc	Pos	Name	Type	CP	Concentration	Standard	Status
☑	C10	Sample 34	Unknown				
☑	C11	Sample 35	Unknown	26,29			
☑	C12	Sample 36	Unknown	36,09			
☑	D1	1680N	Unknown	28,93			
☑	D2	1680N	Unknown	29,12			
☑	D3	1680N	Unknown	29,43			
☑	D4	Sample 40	Unknown				
☑	D5	Sample 41	Unknown	36,63			
☑	D6	Sample 42	Unknown	22,11			
☑	D7	Sample 43	Unknown	24,29			
☑	D8	Sample 44	Unknown	22,87			
☑	D9	Sample 45	Unknown	28,72			
☑	D10	Sample 46	Unknown	30,53			
☑	D11	Sample 47	Unknown	21,57			
☑	D12	Sample 48	Unknown				
☑	E1	1693T	Unknown	26,43			
☑	E2	1693T	Unknown	26,26			
☑	E3	1693T	Unknown	26,69			
☑	E4	Sample 52	Unknown				
☑	E5	Sample 53	Unknown				
☑	E6	Sample 54	Unknown				
☑	E7	Sample 55	Unknown				
☑	E8	Sample 56	Unknown	23,29			
☑	E9	Sample 57	Unknown	29,98			
☑	E10	Sample 58	Unknown	24,56			
☑	E11	Sample 59	Unknown				
☑	E12	Sample 60	Unknown				
☑	F1	1693N	Unknown	27,34			
☑	F2	1693N	Unknown	27,18			
☑	F3	1693N	Unknown	27,03			
☑	F4	Sample 64	Unknown				
☑	F5	Sample 65	Unknown				
☑	F6	Sample 66	Unknown				
☑	F7	Sample 67	Unknown	28,49			
☑	F8	Sample 68	Unknown	35,63			
☑	F9	Sample 69	Unknown	21,79			
☑	F10	Sample 70	Unknown				

Results

Inc	Pos	Name	Type	CP	Concentration	Standard	Status
☑	F11	Sample 71	Unknown				
☑	F12	Sample 72	Unknown				
☑	G1	1705T	Unknown	27,98			
☑	G2	1705T	Unknown	27,37			
☑	G3	1705T	Unknown	28,00			
☑	G4	Sample 76	Unknown				
☑	G5	Sample 77	Unknown				
☑	G6	Sample 78	Unknown				
☑	G7	Sample 79	Unknown	34,50			
☑	G8	Sample 80	Unknown				
☑	G9	Sample 81	Unknown				
☑	G10	Sample 82	Unknown	23,19			
☑	G11	Sample 83	Unknown				
☑	G12	Sample 84	Unknown	34,56			
☑	H1	1705N	Unknown	26,08			
☑	H2	1705N	Unknown	25,92			
☑	H3	1705N	Unknown	25,92			
☑	H4	Sample 88	Unknown	29,74			
☑	H5	Sample 89	Unknown				
☑	H6	Sample 90	Unknown	29,87			
☑	H7	Sample 91	Unknown	28,69			
☑	H8	Sample 92	Unknown				
☑	H9	Sample 93	Unknown				
☑	H10	Sample 94	Unknown				
☑	H11	Sample 95	Unknown	32,48			
☑	H12	Sample 96	Unknown				
