# Supplementary material for: MicroRNAs and Their Inhibition in Modulating SLC5A8 Expression in the Context of Papillary Thyroid Carcinoma
Source: Int J Mol Sci. 2025 Aug 15;26(16):7889. doi: 10.3390/ijms26167889 (PMC12386254; doi:10.3390/ijms26167889)
Supplement: Supplementary file 1 [file ijms-26-07889-s001.zip › ijms-3558049-supplementary/Manuscript data/Fig1 data/Data/RQ-08-05-2012.PDF]

Roche

Abs Quant/2nd Derivative Max for All Samples (Abs Quant/2nd Derivative Max)

Results

Inc	Pos	Name	Type	CP	Concentration	Standard	Status
☑	A1	Sample 1	Unknown				
☑	A2	Sample 2	Unknown				
☑	A3	Sample 3	Unknown				
☑	A4	Sample 4	Unknown	33,05			
☑	A5	Sample 5	Unknown	32,63			
☑	A6	Sample 6	Unknown	33,91			
☑	A7	1532T	Unknown	29,97			
☑	A8	1532T	Unknown	29,88			
☑	A9	1532T	Unknown	29,94			
☑	A10	1614T	Unknown	27,83			
☑	A11	1614T	Unknown	27,92			
☑	A12	1614T	Unknown	27,79			
☑	B1	Sample 13	Unknown				
☑	B2	Sample 14	Unknown				
☑	B3	Sample 15	Unknown				
☑	B4	k-	Unknown				
☑	B5	k-	Unknown				
☑	B6	k-	Unknown				
☑	B7	1532N	Unknown	25,48			
☑	B8	1532N	Unknown	25,49			
☑	B9	1532N	Unknown	25,55			
☑	B10	1614N	Unknown	24,72			
☑	B11	1614N	Unknown	24,83			
☑	B12	1614N	Unknown	24,97			
☑	C1	Sample 25	Unknown				
☑	C2	Sample 26	Unknown				
☑	C3	Sample 27	Unknown				
☑	C4	1:1	Unknown	24,55			
☑	C5	1:1	Unknown	24,58			
☑	C6	1:1	Unknown	24,50			
☑	C7	1539T	Unknown	32,40			
☑	C8	1539T	Unknown	33,08			
☑	C9	1539T	Unknown	33,29			

Results

Inc	Pos	Name	Type	CP	Concentration	Standard	Status
☑	C10	1634T	Unknown	30,00			
☑	C11	1634T	Unknown	30,15			
☑	C12	1634T	Unknown	30,17			
☑	D1	Sample 37	Unknown				
☑	D2	Sample 38	Unknown				
☑	D3	Sample 39	Unknown				
☑	D4	1:2	Unknown				
☑	D5	1:2	Unknown	25,10			
☑	D6	1:2	Unknown	25,20			
☑	D7	1539N	Unknown	27,02			
☑	D8	1539N	Unknown	27,07			
☑	D9	1539N	Unknown	27,06			
☑	D10	1634N	Unknown	26,62			
☑	D11	1634N	Unknown	26,72			
☑	D12	1634N	Unknown	26,64			
☑	E1	Sample 49	Unknown				
☑	E2	Sample 50	Unknown				
☑	E3	Sample 51	Unknown				
☑	E4	1:4	Unknown	26,00			
☑	E5	1:4	Unknown	26,14			
☑	E6	1:4	Unknown	26,04			
☑	E7	1543T	Unknown	26,00			
☑	E8	1543T	Unknown	25,94			
☑	E9	1543T	Unknown	26,00			
☑	E10	1643T	Unknown	28,76			
☑	E11	1643T	Unknown	28,61			
☑	E12	1643T	Unknown	29,04			
☑	F1	Sample 61	Unknown				
☑	F2	Sample 62	Unknown				
☑	F3	Sample 63	Unknown				
☑	F4	1:8	Unknown	30,98			
☑	F5	1:8	Unknown	31,20			
☑	F6	1:8	Unknown	30,84			
☑	F7	1543N	Unknown	27,44			
☑	F8	1543N	Unknown	27,51			
☑	F9	1543N	Unknown	27,46			
☑	F10	1643N	Unknown	29,75			

Results

Inc	Pos	Name	Type	CP	Concentration	Standard	Status
☑	F11	1643N	Unknown	29,55			
☑	F12	1643N	Unknown	29,67			
☑	G1	Sample 73	Unknown				
☑	G2	Sample 74	Unknown				
☑	G3	Sample 75	Unknown				
☑	G4	1:16	Unknown	29,42			
☑	G5	1:16	Unknown	29,57			
☑	G6	1:16	Unknown	29,52			
☑	G7	1547T	Unknown	32,76			
☑	G8	1547T	Unknown	32,59			
☑	G9	1547T	Unknown	31,94			
☑	G10	1644T	Unknown	30,60			
☑	G11	1644T	Unknown	30,82			
☑	G12	1644T	Unknown	31,29			
☑	H1	Sample 85	Unknown				
☑	H2	Sample 86	Unknown				
☑	H3	Sample 87	Unknown				
☑	H4	1:32	Unknown	36,64			
☑	H5	1:32	Unknown	35,29			
☑	H6	1:32	Unknown	35,62			
☑	H7	1547N	Unknown	26,19			
☑	H8	1547N	Unknown	26,42			
☑	H9	1547N	Unknown	26,55			
☑	H10	1644N	Unknown	27,79			
☑	H11	1644N	Unknown	28,05			
☑	H12	1644N	Unknown	28,25			
