# Supplementary material for: MicroRNAs and Their Inhibition in Modulating SLC5A8 Expression in the Context of Papillary Thyroid Carcinoma
Source: Int J Mol Sci. 2025 Aug 15;26(16):7889. doi: 10.3390/ijms26167889 (PMC12386254; doi:10.3390/ijms26167889)
Supplement: Supplementary file 1 [file ijms-26-07889-s001.zip › ijms-3558049-supplementary/Manuscript data/Fig1 data/Data/RQ-09-05-2012.PDF]

Roche

Abs Quant/2nd Derivative Max for All Samples (Abs Quant/2nd Derivative Max)

Results

Inc	Pos	Name	Type	CP	Concentration	Standard	Status
☑	A1	1:1	Unknown	24,62			
☑	A2	1:1	Unknown	24,68			
☑	A3	1:1	Unknown	24,67			
☑	A4	1544T	Unknown	29,20			
☑	A5	1544T	Unknown	29,24			
☑	A6	1544T	Unknown	29,23			
☑	A7	1604T	Unknown	24,89			
☑	A8	1604T	Unknown	24,82			
☑	A9	1604T	Unknown	24,80			
☑	A10	1673T	Unknown	25,61			
☑	A11	1673T	Unknown	25,52			
☑	A12	1673T	Unknown	25,69			
☑	B1	1:2	Unknown	25,43			
☑	B2	1:2	Unknown	25,57			
☑	B3	1:2	Unknown	25,58			
☑	B4	1544N	Unknown	25,47			
☑	B5	1544N	Unknown	25,57			
☑	B6	1544N	Unknown	25,56			
☑	B7	1604N	Unknown	24,52			
☑	B8	1604N	Unknown	24,53			
☑	B9	1604N	Unknown	24,54			
☑	B10	1673N	Unknown	26,06			
☑	B11	1673N	Unknown	26,19			
☑	B12	1673N	Unknown	26,15			
☑	C1	1:4	Unknown	26,46			
☑	C2	1:4	Unknown	26,56			
☑	C3	1:4	Unknown	26,51			
☑	C4	1571T	Unknown	31,95			
☑	C5	1571T	Unknown	31,68			
☑	C6	1571T	Unknown	31,17			
☑	C7	1622T	Unknown	27,44			
☑	C8	1622T	Unknown	27,26			
☑	C9	1622T	Unknown	27,56			

Results

Inc	Pos	Name	Type	CP	Concentration	Standard	Status
☑	C10	1680T	Unknown	30,69			
☑	C11	1680T	Unknown	30,61			
☑	C12	1680T	Unknown	30,89			
☑	D1	1:8	Unknown	27,31			
☑	D2	1:8	Unknown	27,12			
☑	D3	1:8	Unknown	27,60			
☑	D4	1571N	Unknown	25,67			
☑	D5	1571N	Unknown	25,75			
☑	D6	1571N	Unknown	25,77			
☑	D7	1622N	Unknown	26,14			
☑	D8	1622N	Unknown	26,19			
☑	D9	1622N	Unknown	26,13			
☑	D10	1680N	Unknown	28,32			
☑	D11	1680N	Unknown	28,16			
☑	D12	1680N	Unknown	28,48			
☑	E1	1:16	Unknown	28,99			
☑	E2	1:16	Unknown	29,19			
☑	E3	1:16	Unknown	29,25			
☑	E4	1579T	Unknown	33,06			
☑	E5	1579T	Unknown	33,52			
☑	E6	1579T	Unknown	35,07			
☑	E7	1629T	Unknown	29,33			
☑	E8	1629T	Unknown	29,59			
☑	E9	1629T	Unknown	29,60			
☑	E10	1693T	Unknown	25,89			
☑	E11	1693T	Unknown	25,95			
☑	E12	1693T	Unknown	26,03			
☑	F1	1:32	Unknown				
☑	F2	1:32	Unknown				
☑	F3	1:32	Unknown				
☑	F4	1579N	Unknown	26,85			
☑	F5	1579N	Unknown	26,96			
☑	F6	1579N	Unknown	26,83			
☑	F7	1629N	Unknown	26,32			
☑	F8	1629N	Unknown	26,13			
☑	F9	1629N	Unknown	26,07			
☑	F10	1693N	Unknown	27,09			

Results

Inc	Pos	Name	Type	CP	Concentration	Standard	Status
☑	F11	1693N	Unknown	27,01			
☑	F12	1693N	Unknown	27,07			
☑	G1	Sample 73	Unknown	32,77			
☑	G2	Sample 74	Unknown	30,81			
☑	G3	Sample 75	Unknown				
☑	G4	1580T	Unknown	31,16			
☑	G5	1580T	Unknown	31,33			
☑	G6	1580T	Unknown	31,95			
☑	G7	1632T	Unknown	30,44			
☑	G8	1632T	Unknown	30,47			
☑	G9	1632T	Unknown	29,83			
☑	G10	1705T	Unknown	26,92			
☑	G11	1705T	Unknown	26,79			
☑	G12	1705T	Unknown	26,92			
☑	H1	k-	Unknown				
☑	H2	k-	Unknown				
☑	H3	k-	Unknown				
☑	H4	1580N	Unknown	26,92			
☑	H5	1580N	Unknown	26,95			
☑	H6	1580N	Unknown	26,84			
☑	H7	1632N	Unknown	25,72			
☑	H8	1632N	Unknown	25,69			
☑	H9	1632N	Unknown	25,85			
☑	H10	1705N	Unknown	25,31			
☑	H11	1705N	Unknown	25,49			
☑	H12	1705N	Unknown	25,74			
