# Supplementary material for: MicroRNAs and Their Inhibition in Modulating SLC5A8 Expression in the Context of Papillary Thyroid Carcinoma
Source: Int J Mol Sci. 2025 Aug 15;26(16):7889. doi: 10.3390/ijms26167889 (PMC12386254; doi:10.3390/ijms26167889)
Supplement: Supplementary file 1 [file ijms-26-07889-s001.zip › ijms-3558049-supplementary/Manuscript data/Fig1 data/Data/RQ-15-05-2012part.PDF]

Abs Quant/2nd Derivative Max for All Samples (Abs Quant/2nd Derivative Max)

Results

Inc	Pos	Name	Type	CP	Concentration	Standard	Status
☑	A1	1:1	Unknown	25,91			
☑	A2	1:1	Unknown	25,97			
☑	A3	1:1	Unknown	25,91			
☑	A4	Sample 4	Unknown	28,48			
☑	A5	Sample 5	Unknown	27,46			
☑	A6	Sample 6	Unknown	31,92			
☑	A7	1532T	Unknown	26,18			
☑	A8	1532T	Unknown	26,06			
☑	A9	1532T	Unknown	26,13			
☑	A10	1614T	Unknown				
☑	A11	1614T	Unknown				
☑	A12	1614T	Unknown				
☑	B1	1:2	Unknown	26,92			
☑	B2	1:2	Unknown	26,67			
☑	B3	1:2	Unknown	26,91			
☑	B4	Sample 16	Unknown	35,13			
☑	B5	Sample 17	Unknown	34,87			
☑	B6	Sample 18	Unknown				
☑	B7	1532N	Unknown	26,52			
☑	B8	1532N	Unknown	26,31			
☑	B9	1532N	Unknown	26,55			
☑	B10	1614N	Unknown	25,87			
☑	B11	1614N	Unknown	25,96			
☑	B12	1614N	Unknown	25,95			
☑	C1	1:4	Unknown	27,62			
☑	C2	1:4	Unknown	27,58			
☑	C3	1:4	Unknown	27,76			
☑	C4	Sample 28	Unknown	34,27			
☑	C5	Sample 29	Unknown	34,18			
☑	C6	Sample 30	Unknown				
☑	C7	1539T	Unknown	26,95			
☑	C8	1539T	Unknown	26,86			

> - Late Cp call (last five cycles) has higher uncertainty

Results

Inc	Pos	Name	Type	CP	Concentration	Standard	Status
☑	C9	1539T	Unknown	26,80			
☑	C10	1634T	Unknown	26,17			
☑	C11	1634T	Unknown	26,09			
☑	C12	1634T	Unknown	26,18			
☑	D1	1:8	Unknown	28,82			
☑	D2	1:8	Unknown	28,92			
☑	D3	1:8	Unknown	28,75			
☑	D4	Sample 40	Unknown	27,02			
☑	D5	Sample 41	Unknown	27,86			
☑	D6	Sample 42	Unknown	35,42			
☑	D7	1539N	Unknown	25,94			
☑	D8	1539N	Unknown	25,90			
☑	D9	1539N	Unknown	25,99			
☑	D10	1634N	Unknown	27,08			
☑	D11	1634N	Unknown	27,03			
☑	D12	1634N	Unknown	27,04			
☑	E1	1:16	Unknown	30,25			
☑	E2	1:16	Unknown	30,49			
☑	E3	1:16	Unknown	30,50			
☑	E4	Sample 52	Unknown	28,03			
☑	E5	Sample 53	Unknown	32,50			
☑	E6	Sample 54	Unknown	45,00			>
☑	E7	1543T	Unknown	27,43			
☑	E8	1543T	Unknown	27,29			
☑	E9	1543T	Unknown	27,05			
☑	E10	1643T	Unknown	25,56			
☑	E11	1643T	Unknown	25,59			
☑	E12	1643T	Unknown	25,67			
☑	F1	1:32	Unknown				
☑	F2	1:32	Unknown				
☑	F3	1:32	Unknown				
☑	F4	Sample 64	Unknown	30,10			
☑	F5	Sample 65	Unknown	31,88			
☑	F6	Sample 66	Unknown	41,42			
☑	F7	1543N	Unknown	28,91			
☑	F8	1543N	Unknown	28,58			
☑	F9	1543N	Unknown	28,55			

> - Late Cp call (last five cycles) has higher uncertainty

Results

Inc	Pos	Name	Type	CP	Concentration	Standard	Status
☑	F10	1643N	Unknown	28,74			
☑	F11	1643N	Unknown	28,54			
☑	F12	1643N	Unknown	28,61			
☑	G1	Sample 73	Unknown				
☑	G2	Sample 74	Unknown				
☑	G3	Sample 75	Unknown	20,67			
☑	G4	Sample 76	Unknown	39,19			
☑	G5	Sample 77	Unknown	41,24			
☑	G6	Sample 78	Unknown				
☑	G7	1547T	Unknown	25,87			
☑	G8	1547T	Unknown	26,09			
☑	G9	1547T	Unknown	25,98			
☑	G10	1644T	Unknown	25,46			
☑	G11	1644T	Unknown	25,64			
☑	G12	1644T	Unknown	26,34			
☑	H1	Sample 85	Unknown				
☑	H2	Sample 86	Unknown				
☑	H3	Sample 87	Unknown				
☑	H4	Sample 88	Unknown				
☑	H5	Sample 89	Unknown				
☑	H6	Sample 90	Unknown				
☑	H7	1547N	Unknown	27,55			
☑	H8	1547N	Unknown	27,30			
☑	H9	1547N	Unknown	27,49			
☑	H10	1644N	Unknown	28,68			
☑	H11	1644N	Unknown	32,73			
☑	H12	1644N	Unknown				

> - Late Cp call (last five cycles) has higher uncertainty
