# Supplementary material for: MicroRNAs and Their Inhibition in Modulating SLC5A8 Expression in the Context of Papillary Thyroid Carcinoma
Source: Int J Mol Sci. 2025 Aug 15;26(16):7889. doi: 10.3390/ijms26167889 (PMC12386254; doi:10.3390/ijms26167889)
Supplement: Supplementary file 1 [file ijms-26-07889-s001.zip › ijms-3558049-supplementary/Manuscript data/Fig1 data/Data/RQ-17-05-2012.PDF]

Roche

Abs Quant/2nd Derivative Max for All Samples (Abs Quant/2nd Derivative Max)

Results

Inc	Pos	Name	Type	CP	Concentration	Standard	Status
☑	A1	1501T	Unknown	27,11			
☑	A2	1501T	Unknown	26,98			
☑	A3	1501T	Unknown	27,00			
☑	A4	1644T	Unknown	26,97			
☑	A5	1644T	Unknown	26,94			
☑	A6	1644T	Unknown	27,17			
☑	A7	1580T	Unknown	28,47			
☑	A8	1580T	Unknown	28,45			
☑	A9	1580T	Unknown	28,33			
☑	A10	1632T	Unknown	27,69			
☑	A11	1632T	Unknown	27,69			
☑	A12	1632T	Unknown	27,69			
☑	B1	1501N	Unknown	28,58			
☑	B2	1501N	Unknown	28,25			
☑	B3	1501N	Unknown	28,01			
☑	B4	1644N	Unknown	29,10			
☑	B5	1644N	Unknown	28,97			
☑	B6	1644N	Unknown	29,23			
☑	B7	1580N	Unknown	28,01			
☑	B8	1580N	Unknown	28,12			
☑	B9	1580N	Unknown	27,74			
☑	B10	1632N	Unknown	27,64			
☑	B11	1632N	Unknown	27,66			
☑	B12	1632N	Unknown	27,57			
☑	C1	1508T	Unknown	27,93			
☑	C2	1508T	Unknown	27,75			
☑	C3	1508T	Unknown	27,87			
☑	C4	1544T	Unknown	27,26			
☑	C5	1544T	Unknown	27,29			
☑	C6	1544T	Unknown	27,58			
☑	C7	1604T	Unknown	27,64			
☑	C8	1604T	Unknown	27,54			
☑	C9	1604T	Unknown	27,08			

Results

Inc	Pos	Name	Type	CP	Concentration	Standard	Status
☑	C10	1673T	Unknown	27,64			
☑	C11	1673T	Unknown	27,71			
☑	C12	1673T	Unknown	27,79			
☑	D1	1508N	Unknown	27,81			
☑	D2	1508N	Unknown	27,72			
☑	D3	1508N	Unknown	27,61			
☑	D4	1544N	Unknown	27,99			
☑	D5	1544N	Unknown	27,93			
☑	D6	1544N	Unknown	27,86			
☑	D7	1604N	Unknown	27,24			
☑	D8	1604N	Unknown	27,52			
☑	D9	1604N	Unknown	27,52			
☑	D10	1673N	Unknown	28,07			
☑	D11	1673N	Unknown	27,76			
☑	D12	1673N	Unknown	28,06			
☑	E1	1510T	Unknown	27,62			
☑	E2	1510T	Unknown	27,62			
☑	E3	1510T	Unknown	27,53			
☑	E4	1571T	Unknown	27,80			
☑	E5	1571T	Unknown	27,74			
☑	E6	1571T	Unknown	27,94			
☑	E7	1622T	Unknown	26,12			
☑	E8	1622T	Unknown	26,20			
☑	E9	1622T	Unknown	26,30			
☑	E10	1680T	Unknown	28,59			
☑	E11	1680T	Unknown	28,63			
☑	E12	1680T	Unknown	28,85			
☑	F1	1510N	Unknown	28,15			
☑	F2	1510N	Unknown	27,95			
☑	F3	1510N	Unknown	27,98			
☑	F4	1571N	Unknown	27,72			
☑	F5	1571N	Unknown	27,73			
☑	F6	1571N	Unknown	27,81			
☑	F7	1622N	Unknown	27,80			
☑	F8	1622N	Unknown	27,99			
☑	F9	1622N	Unknown	27,93			
☑	F10	1680N	Unknown	29,10			

Results

Inc	Pos	Name	Type	CP	Concentration	Standard	Status
☑	F11	1680N	Unknown	29,14			
☑	F12	1680N	Unknown	29,19			
☑	G1	1529T	Unknown	27,54			
☑	G2	1529T	Unknown	27,47			
☑	G3	1529T	Unknown	27,47			
☑	G4	1579T	Unknown	27,27			
☑	G5	1579T	Unknown	27,31			
☑	G6	1579T	Unknown	27,45			
☑	G7	1629T	Unknown	27,75			
☑	G8	1629T	Unknown	27,55			
☑	G9	1629T	Unknown	27,54			
☑	G10	1614T	Unknown	27,34			
☑	G11	1614T	Unknown	27,29			
☑	G12	1614T	Unknown	27,25			
☑	H1	1529N	Unknown	29,12			
☑	H2	1529N	Unknown	29,23			
☑	H3	1529N	Unknown	29,24			
☑	H4	1579N	Unknown	28,04			
☑	H5	1579N	Unknown	28,25			
☑	H6	1579N	Unknown	28,14			
☑	H7	1629N	Unknown	28,29			
☑	H8	1629N	Unknown	28,52			
☑	H9	1629N	Unknown	28,66			
☑	H10	k-	Unknown				
☑	H11	k-	Unknown				
☑	H12	k-	Unknown				
