# Supplementary material for: MicroRNAs and Their Inhibition in Modulating SLC5A8 Expression in the Context of Papillary Thyroid Carcinoma
Source: Int J Mol Sci. 2025 Aug 15;26(16):7889. doi: 10.3390/ijms26167889 (PMC12386254; doi:10.3390/ijms26167889)
Supplement: Supplementary file 1 [file ijms-26-07889-s001.zip › ijms-3558049-supplementary/Manuscript data/Fig1 data/Data/RQ-18-05-2012.PDF]

Roche

Abs Quant/2nd Derivative Max for All Samples (Abs Quant/2nd Derivative Max)

Results

Inc	Pos	Name	Type	CP	Concentration	Standard	Status
☑	A1	1693T	Unknown	26,60			
☑	A2	1693T	Unknown	26,76			
☑	A3	1693T	Unknown	26,67			
☑	A4	Sample 4	Unknown				
☑	A5	Sample 5	Unknown				
☑	A6	Sample 6	Unknown				
☑	A7	Sample 7	Unknown				
☑	A8	Sample 8	Unknown				
☑	A9	Sample 9	Unknown				
☑	A10	Sample 10	Unknown				
☑	A11	Sample 11	Unknown				
☑	A12	Sample 12	Unknown				
☑	B1	1693N	Unknown	26,82			
☑	B2	1693N	Unknown	27,06			
☑	B3	1693N	Unknown	26,94			
☑	B4	Sample 16	Unknown	29,15			
☑	B5	Sample 17	Unknown	40,07			
☑	B6	Sample 18	Unknown				
☑	B7	Sample 19	Unknown	45,00			?, >
☑	B8	Sample 20	Unknown				
☑	B9	Sample 21	Unknown				
☑	B10	Sample 22	Unknown				
☑	B11	Sample 23	Unknown				
☑	B12	Sample 24	Unknown				
☑	C1	1705T	Unknown	26,97			
☑	C2	1705T	Unknown	27,02			
☑	C3	1705T	Unknown	27,06			
☑	C4	Sample 28	Unknown				
☑	C5	Sample 29	Unknown				
☑	C6	Sample 30	Unknown				
☑	C7	Sample 31	Unknown				
☑	C8	Sample 32	Unknown	31,04			?

? - Detector Call uncertain, > - Late Cp call (last five cycles) has higher uncertainty

Results

Inc	Pos	Name	Type	CP	Concentration	Standard	Status
☑	C9	Sample 33	Unknown				
☑	C10	Sample 34	Unknown				
☑	C11	Sample 35	Unknown	29,77			?
☑	C12	Sample 36	Unknown				
☑	D1	1705N	Unknown	26,43			
☑	D2	1705N	Unknown	26,50			
☑	D3	1705N	Unknown	26,62			
☑	D4	Sample 40	Unknown				
☑	D5	Sample 41	Unknown	37,22			?
☑	D6	Sample 42	Unknown				
☑	D7	Sample 43	Unknown	28,43			?
☑	D8	Sample 44	Unknown				
☑	D9	Sample 45	Unknown				
☑	D10	Sample 46	Unknown				
☑	D11	Sample 47	Unknown				
☑	D12	Sample 48	Unknown				
☑	E1	k-	Unknown				
☑	E2	k-	Unknown				
☑	E3	k-	Unknown				
☑	E4	Sample 52	Unknown				
☑	E5	Sample 53	Unknown				
☑	E6	Sample 54	Unknown	20,99			
☑	E7	Sample 55	Unknown	28,30			
☑	E8	Sample 56	Unknown				
☑	E9	Sample 57	Unknown				
☑	E10	Sample 58	Unknown				
☑	E11	Sample 59	Unknown				
☑	E12	Sample 60	Unknown				
☑	F1	Sample 61	Unknown				
☑	F2	Sample 62	Unknown				
☑	F3	Sample 63	Unknown				
☑	F4	Sample 64	Unknown				
☑	F5	Sample 65	Unknown				
☑	F6	Sample 66	Unknown				
☑	F7	Sample 67	Unknown				
☑	F8	Sample 68	Unknown				
☑	F9	Sample 69	Unknown				

? - Detector Call uncertain, > - Late Cp call (last five cycles) has higher uncertainty

Results

Inc	Pos	Name	Type	CP	Concentration	Standard	Status
☑	F10	Sample 70	Unknown				
☑	F11	Sample 71	Unknown	28,76			?
☑	F12	Sample 72	Unknown				
☑	G1	Sample 73	Unknown	39,67			?
☑	G2	Sample 74	Unknown				
☑	G3	Sample 75	Unknown				
☑	G4	Sample 76	Unknown	38,44			?
☑	G5	Sample 77	Unknown				
☑	G6	Sample 78	Unknown				
☑	G7	Sample 79	Unknown				
☑	G8	Sample 80	Unknown				
☑	G9	Sample 81	Unknown				
☑	G10	Sample 82	Unknown				
☑	G11	Sample 83	Unknown	35,62			
☑	G12	Sample 84	Unknown				
☑	H1	Sample 85	Unknown				
☑	H2	Sample 86	Unknown				
☑	H3	Sample 87	Unknown	28,19			
☑	H4	Sample 88	Unknown				
☑	H5	Sample 89	Unknown	32,76			
☑	H6	Sample 90	Unknown				
☑	H7	Sample 91	Unknown				
☑	H8	Sample 92	Unknown	27,15			?
☑	H9	Sample 93	Unknown				
☑	H10	Sample 94	Unknown				
☑	H11	Sample 95	Unknown				
☑	H12	Sample 96	Unknown				

? - Detector Call uncertain, > - Late Cp call (last five cycles) has higher uncertainty
