# Supplementary material for: MicroRNAs and Their Inhibition in Modulating SLC5A8 Expression in the Context of Papillary Thyroid Carcinoma
Source: Int J Mol Sci. 2025 Aug 15;26(16):7889. doi: 10.3390/ijms26167889 (PMC12386254; doi:10.3390/ijms26167889)

Roche

Abs Quant/2nd Derivative Max for All Samples (Abs Quant/2nd Derivative Max)

Results

Inc	Pos	Name	Type	CP	Concentration	Standard	Status
☑	A1	1580T	Unknown	32,75			
☑	A2	1580T	Unknown	33,08			
☑	A3	1580T	Unknown	34,15			
☑	A4	1632T	Unknown	31,23			
☑	A5	1632T	Unknown	31,49			
☑	A6	1632T	Unknown	31,05			
☑	A7	Sample 7	Unknown	15,42			
☑	A8	Sample 8	Unknown	15,07			
☑	A9	Sample 9	Unknown				
☑	A10	Sample 10	Unknown	18,42			
☑	A11	Sample 11	Unknown	23,73			?
☑	A12	Sample 12	Unknown	16,42			
☑	B1	1580N	Unknown	27,18			
☑	B2	1580N	Unknown	27,28			
☑	B3	1580N	Unknown	27,41			
☑	B4	1632N	Unknown	27,08			
☑	B5	1632N	Unknown	26,80			
☑	B6	1632N	Unknown	26,81			
☑	B7	Sample 19	Unknown	36,21			
☑	B8	Sample 20	Unknown				
☑	B9	Sample 21	Unknown	19,14			
☑	B10	Sample 22	Unknown	16,74			
☑	B11	Sample 23	Unknown	18,69			
☑	B12	Sample 24	Unknown	22,52			?
☑	C1	1604T	Unknown	25,62			
☑	C2	1604T	Unknown	25,56			
☑	C3	1604T	Unknown	25,78			
☑	C4	k-	Unknown				
☑	C5	k-	Unknown	38,11			
☑	C6	k-	Unknown				
☑	C7	Sample 31	Unknown				
☑	C8	Sample 32	Unknown	24,63			

? - Detector Call uncertain

Results

Inc	Pos	Name	Type	CP	Concentration	Standard	Status
☑	C9	Sample 33	Unknown	18,02			
☑	C10	Sample 34	Unknown				
☑	C11	Sample 35	Unknown	21,95			
☑	C12	Sample 36	Unknown				
☑	D1	1604N	Unknown	25,10			
☑	D2	1604N	Unknown	25,53			
☑	D3	1604N	Unknown	25,60			
☑	D4	Sample 40	Unknown	19,43			
☑	D5	Sample 41	Unknown	24,08			
☑	D6	Sample 42	Unknown	19,84			
☑	D7	Sample 43	Unknown	15,74			
☑	D8	Sample 44	Unknown	15,61			
☑	D9	Sample 45	Unknown	19,09			
☑	D10	Sample 46	Unknown	19,89			
☑	D11	Sample 47	Unknown	19,95			
☑	D12	Sample 48	Unknown	22,62			
☑	E1	1622T	Unknown	34,53			
☑	E2	1622T	Unknown	28,30			
☑	E3	1622T	Unknown	28,65			
☑	E4	Sample 52	Unknown				
☑	E5	Sample 53	Unknown	24,96			
☑	E6	Sample 54	Unknown	17,94			
☑	E7	Sample 55	Unknown	17,60			
☑	E8	Sample 56	Unknown	17,11			
☑	E9	Sample 57	Unknown	20,62			
☑	E10	Sample 58	Unknown	19,69			
☑	E11	Sample 59	Unknown	16,96			
☑	E12	Sample 60	Unknown	30,50			
☑	F1	1622N	Unknown	26,98			
☑	F2	1622N	Unknown	26,51			
☑	F3	1622N	Unknown	26,79			
☑	F4	Sample 64	Unknown	24,60			
☑	F5	Sample 65	Unknown				
☑	F6	Sample 66	Unknown	18,46			
☑	F7	Sample 67	Unknown				
☑	F8	Sample 68	Unknown	21,81			
☑	F9	Sample 69	Unknown	23,71			

? - Detector Call uncertain

Results

Inc	Pos	Name	Type	CP	Concentration	Standard	Status
☑	F10	Sample 70	Unknown				
☑	F11	Sample 71	Unknown	24,89			
☑	F12	Sample 72	Unknown	31,41			?
☑	G1	1629T	Unknown	30,32			
☑	G2	1629T	Unknown	30,52			
☑	G3	1629T	Unknown	30,50			
☑	G4	Sample 76	Unknown				
☑	G5	Sample 77	Unknown				
☑	G6	Sample 78	Unknown				
☑	G7	Sample 79	Unknown	18,65			
☑	G8	Sample 80	Unknown	20,68			
☑	G9	Sample 81	Unknown	31,99			
☑	G10	Sample 82	Unknown	20,02			
☑	G11	Sample 83	Unknown	18,45			
☑	G12	Sample 84	Unknown	17,33			?
☑	H1	1629N	Unknown	27,14			
☑	H2	1629N	Unknown	27,14			
☑	H3	1629N	Unknown	27,07			
☑	H4	Sample 88	Unknown	26,14			
☑	H5	Sample 89	Unknown	33,74			
☑	H6	Sample 90	Unknown	25,70			
☑	H7	Sample 91	Unknown	18,70			
☑	H8	Sample 92	Unknown	25,11			
☑	H9	Sample 93	Unknown	17,07			?
☑	H10	Sample 94	Unknown	27,00			
☑	H11	Sample 95	Unknown	25,97			
☑	H12	Sample 96	Unknown				

? - Detector Call uncertain

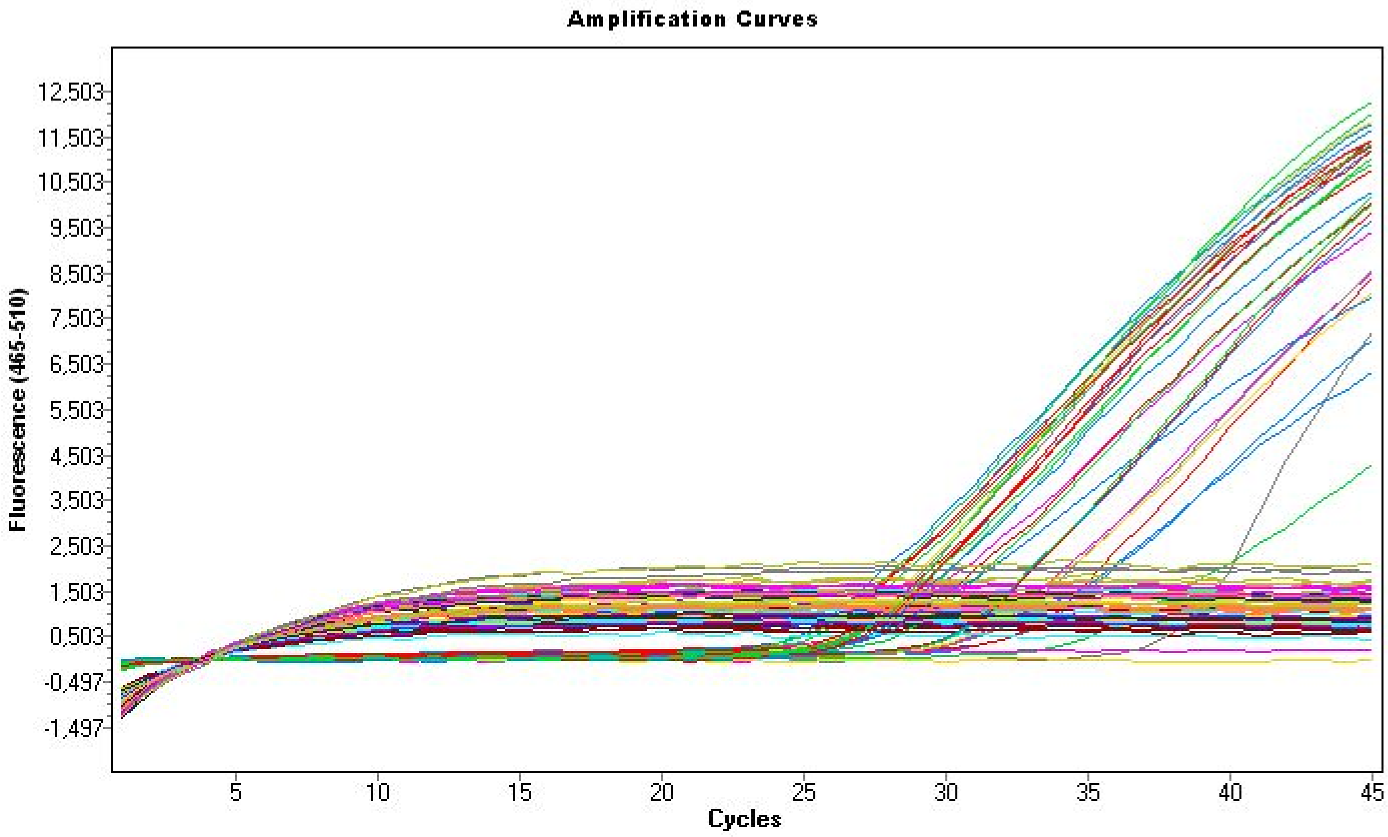

Tm Calling for All Samples (Tm Calling)

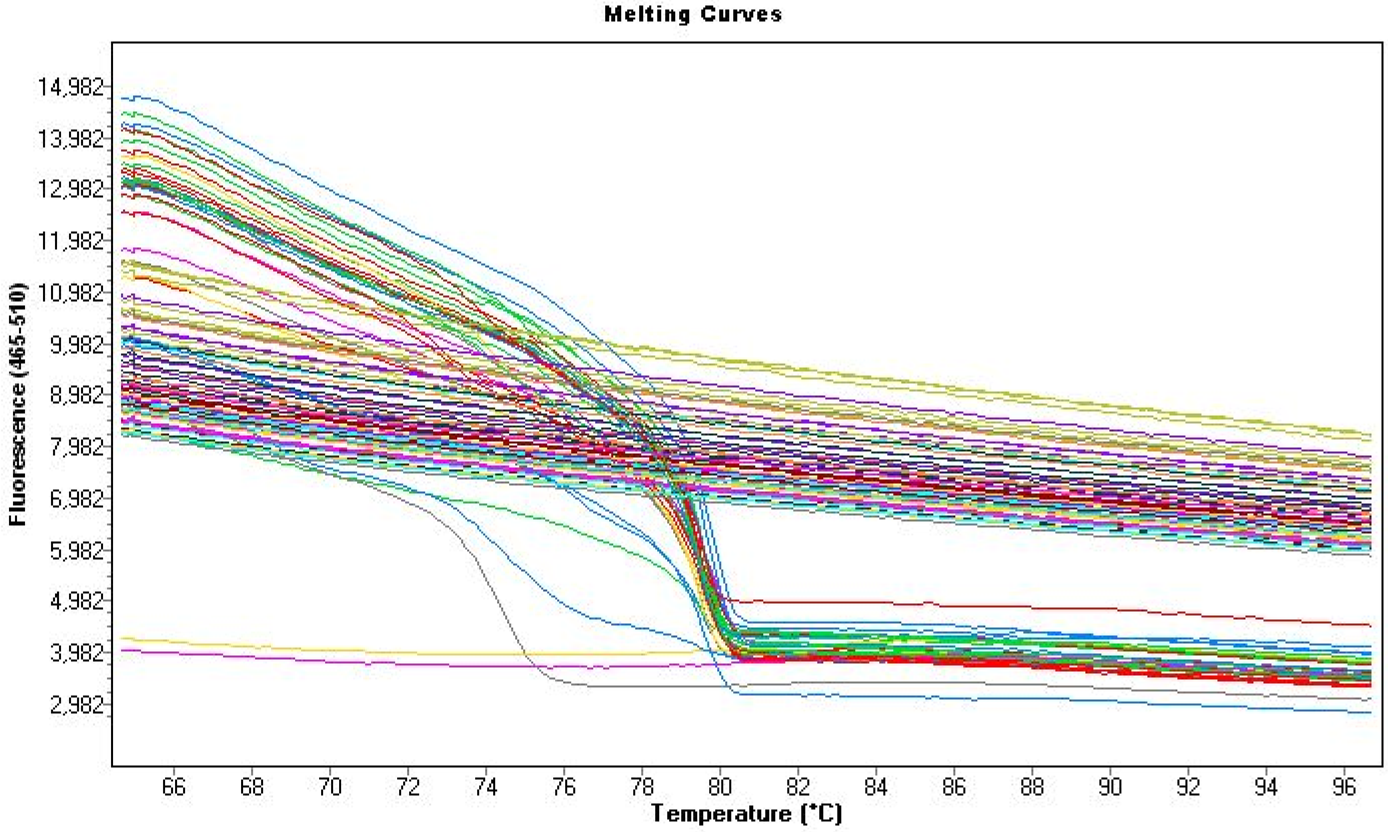

Melt Curve Genotyping for All Samples (Melt Curve Genotyping)

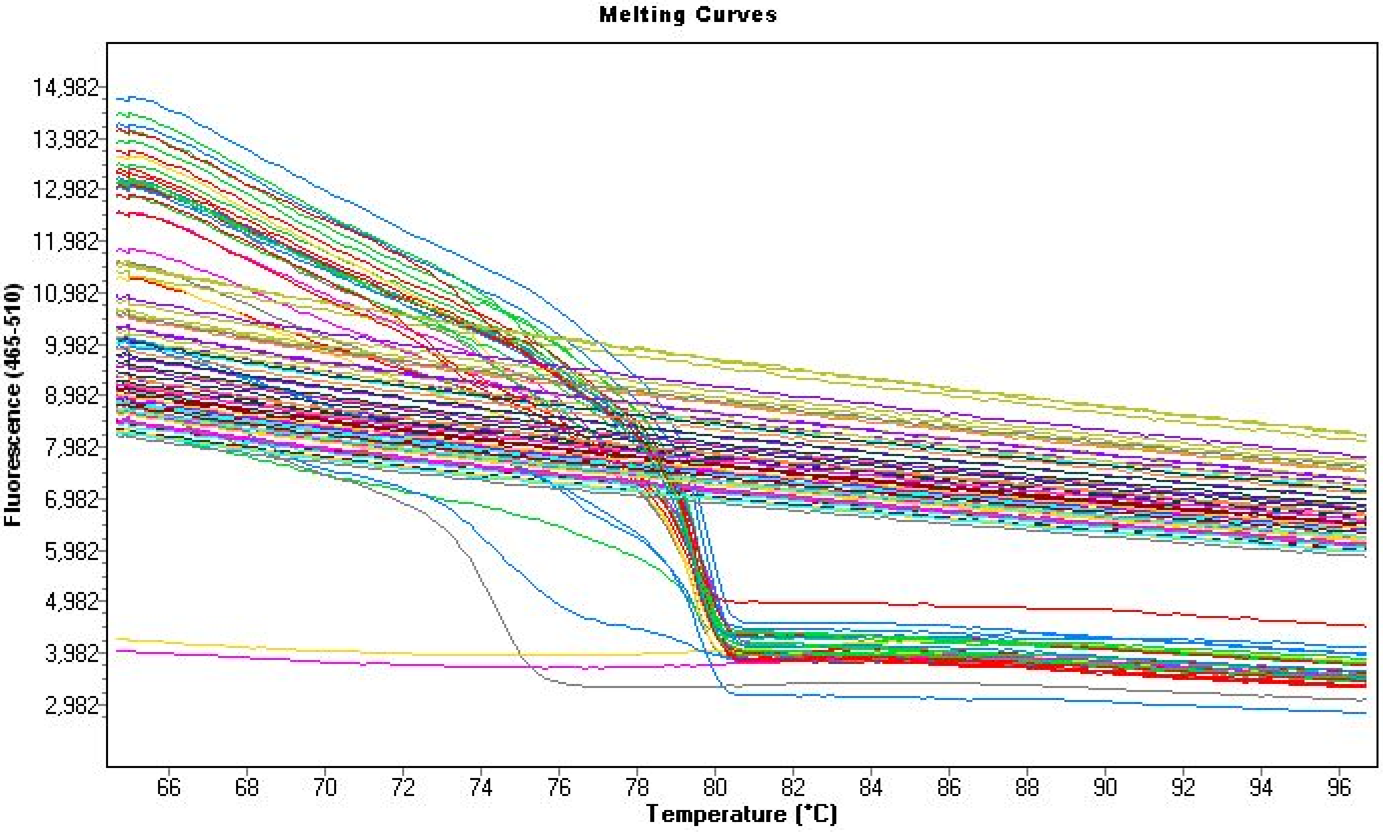

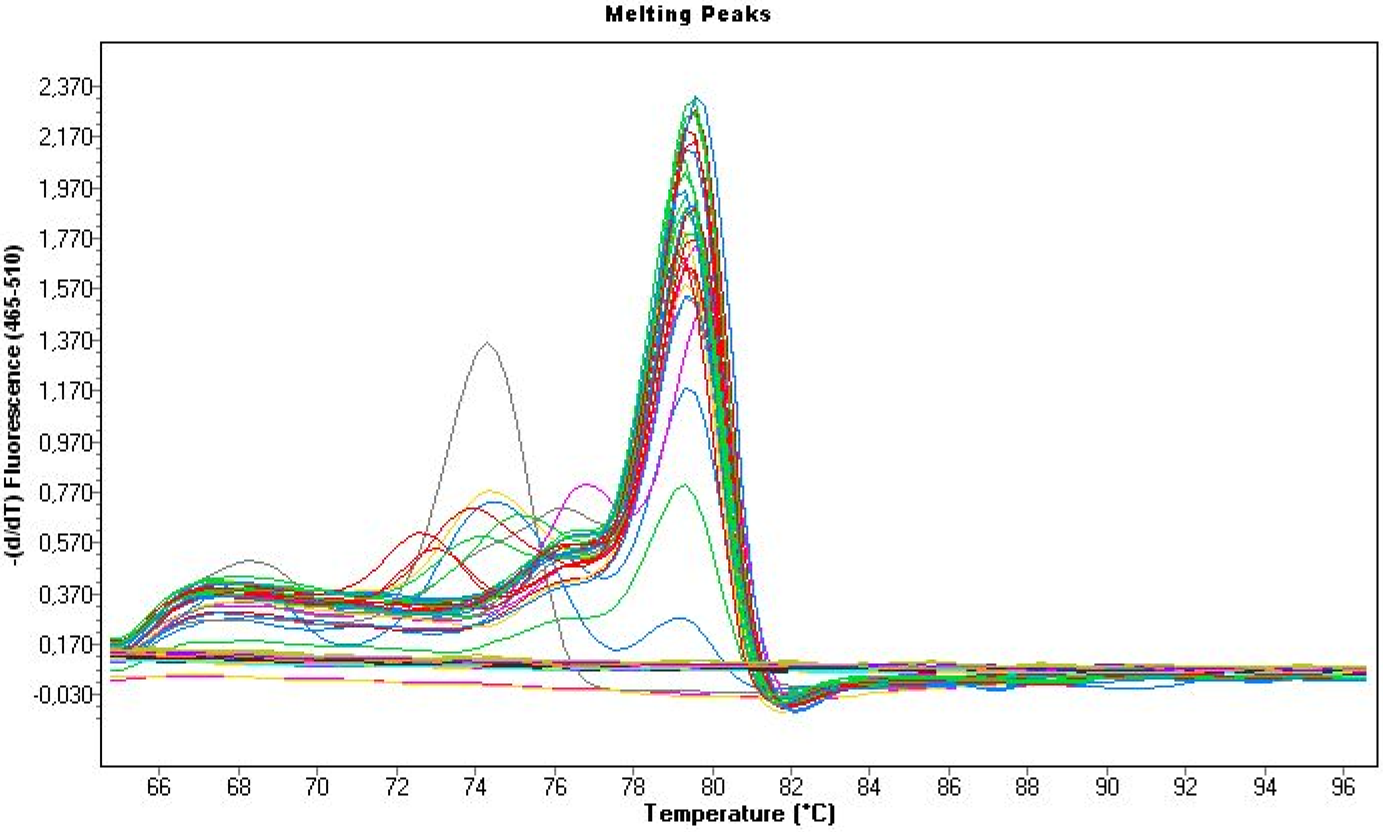

Supplement: Supplementary file 1 [file ijms-26-07889-s001.zip › ijms-3558049-supplementary/Manuscript data/Fig1 data/Data/wojtek_wyniki_26-04-2012.PDF]
