# Supplementary material for: MicroRNAs and Their Inhibition in Modulating SLC5A8 Expression in the Context of Papillary Thyroid Carcinoma
Source: Int J Mol Sci. 2025 Aug 15;26(16):7889. doi: 10.3390/ijms26167889 (PMC12386254; doi:10.3390/ijms26167889)
Supplement: Supplementary file 1 [file ijms-26-07889-s001.zip › ijms-3558049-supplementary/Manuscript data/Fig4 data/2013-10-03 miRy w PTC płytki 1-2.PDF]

Roche

Wojtek_2013-10-03 miRy w parach 1-2

Programs

Program Name	pre-incubation		
Cycles	1	Analysis Mode	None

Target (°C)	Acquisition Mode	Hold (hh:mm:ss)	Ramp Rate (°C/s)	Acquisitions (per °C)	Sec Target (°C)	Step size (°C)	Step Delay (cycles)
95	None	00:10:00	4,80		0	0	0

Program Name	amplification		
Cycles	45	Analysis Mode	Quantification

Target (°C)	Acquisition Mode	Hold (hh:mm:ss)	Ramp Rate (°C/s)	Acquisitions (per °C)	Sec Target (°C)	Step size (°C)	Step Delay (cycles)
95	None	00:00:10	4,80		0	0	0
60	Single	00:00:30	2,50		0	0	0
72	None	00:00:01	4,80		0	0	0

Program Name	cooling		
Cycles	1	Analysis Mode	None

Target (°C)	Acquisition Mode	Hold (hh:mm:ss)	Ramp Rate (°C/s)	Acquisitions (per °C)	Sec Target (°C)	Step size (°C)	Step Delay (cycles)
40	None	00:00:30	2,50		0	0	0

Abs Quant/2nd Derivative Max for All (Abs Quant/2nd Derivative Max)

Statistics

Samples	Mean Cp	Std Cp	Mean conc	Std conc
A1, A2, B1	25,75	0,16		
A3, A4, B3	25,02	0,34		
A5, A6, B5	24,45	0,36		
A7, A8, B7	25,94	0,40		
A10, A9, B9	27,49	0,31		
A11, A12, B11	26,40	0,19		
A13, A14, B13	26,86	0,33		
A15, A16, B15	25,58	0,26		
A17, A18, B17	25,99	0,50		
A19, A20, B19	28,40	0,45		
A21, A22, B21	27,82	0,36		
A23, A24, B23	27,67	0,26		
B2, C1, C2	24,95	0,39		
B4, C3, C4	26,04	0,32		

Statistics

Samples	Mean Cp	Std Cp	Mean conc	Std conc
B6, C5, C6	26,11	0,15		
B8, C7, C8	27,22	0,48		
B10, C10, C9	25,95	0,47		
B12, C11, C12	24,98	0,59		
B14, C13, C14	25,37	0,37		
B16, C15, C16	25,70	0,10		
B18, C17, C18	26,59	0,54		
B20, C19, C20	25,95	0,34		
B22, C21, C22	24,68	0,57		
B24, C23, C24	25,84	0,30		
D1, D2, E1	25,57	0,27		
D3, D4, E3	24,55	0,29		
D5, D6, E5	24,55	0,28		
D7, D8, E7	24,92	0,23		
D10, D9, E9	27,19	0,28		
D11, D12, E11	24,28	0,26		
D13, D14, E13	25,89	0,25		
D15, D16, E15	24,07	0,17		
D17, D18, E17	24,31	0,51		
D19, D20, E19	25,29	0,57		
D21, D22, E21	25,38	0,48		
D23, D24, E23	26,03	0,32		
E2, F1, F2	23,78	0,52		
E4, F3, F4	23,59	0,24		
E6, F5, F6	24,58	0,34		
E8, F7, F8	24,28	0,30		
E10, F10, F9	24,46	0,26		
E12, F11, F12	24,26	0,41		
E14, F13, F14	24,50	0,22		
E16, F15, F16	23,58	0,35		
E18, F17, F18	25,75	0,21		
E20, F19, F20	24,37	0,30		
E22, F21, F22	22,66	0,19		
E24, F23, F24	23,25	0,29		
G1, G2, H1	29,88	0,21		
G3, G4, H3	28,67	0,20		
G5, G6, H5	28,38	0,25		

Statistics

Samples	Mean Cp	Std Cp	Mean conc	Std conc
G7, G8, H7	30,27	0,56		
G10, G9, H9	30,36	0,28		
G11, G12, H11	29,72	0,26		
G13, G14, H13	30,03	0,28		
G15, G16, H15	29,85	0,14		
G17, G18, H17	28,01	0,38		
G19, G20, H19	31,16	0,72		
G21, G22, H21	30,71	0,45		
G23, G24, H23	30,94	0,17		
H2, I1, I2	28,49	0,15		
H4, I3, I4	28,44	0,26		
H6, I5, I6	29,43	0,11		
H8, I7, I8	29,77	0,38		
H10, I10, I9	27,91	0,55		
H12, I11, I12	29,20	0,27		
H14, I13, I14	29,27	0,26		
H16, I15, I16	28,90	0,68		
H18, I17, I18	29,93	0,79		
H20, I19, I20	29,33	0,49		
H22, I21, I22	27,96	0,40		
H24, I23, I24	28,51	0,38		
J1, J2, K1	34,85	0,20		
J3, J4, K3	35,43	0,14		
J5, J6, K5	31,62	0,29		
J7, J8, K7	35,01	0,11		
J10, J9, K9	34,56	0,22		
J11, J12, K11	35,95	0,15		
J13, J14, K13	34,86	0,27		
J15, J16, K15	35,26	0,44		
J17, J18, K17	33,74	0,41		
J19, J20, K19	36,05	0,19		
J21, J22, K21	35,04	0,36		
J23, J24, K23	36,49	0,40		
K2, L1, L2	33,41	0,33		
K4, L3, L4	34,38	0,17		
K6, L5, L6	35,68	0,42		
K8, L7, L8	36,07	0,41		

Statistics

Samples	Mean Cp	Std Cp	Mean conc	Std conc
K10, L10, L9	32,69	0,32		
K12, L11, L12	34,84	0,73		
K14, L13, L14	33,51	0,31		
K16, L15, L16	33,63	0,07		
K18, L17, L18	36,24	0,50		
K20, L19, L20	35,83	0,17		
K22, L21, L22	33,85	0,34		
K24, L23, L24	33,51	0,23		
M1, M2, N1	31,82	0,10		
M3, M4, N3	31,64	0,15		
M5, M6, N5	31,57	0,46		
M7, M8, N7	34,62	0,48		
M10, M9, N9	33,60	0,34		
M11, M12, N11	34,15	0,30		
M13, M14, N13	34,17	0,24		
M15, M16, N15	34,86	0,22		
M17, M18, N17	33,12	0,20		
M19, M20, N19	33,70	0,20		
M21, M22, N21	29,76	0,18		
M23, M24, N23	31,73	0,31		
N2, O1, O2	32,99	0,26		
N4, O3, O4	32,18	0,34		
N6, O5, O6	31,28	0,23		
N8, O7, O8	31,26	0,39		
N10, O10, O9	33,37	0,08		
N12, O11, O12	33,87	0,15		
N14, O13, O14	31,82	0,46		
N16, O15, O16	34,08	0,36		
N18, O17, O18	35,17	0,38		
N20, O19, O20	33,76	0,12		
N22, O21, O22	32,86	0,08		
N24, O23, O24	31,96	0,49		

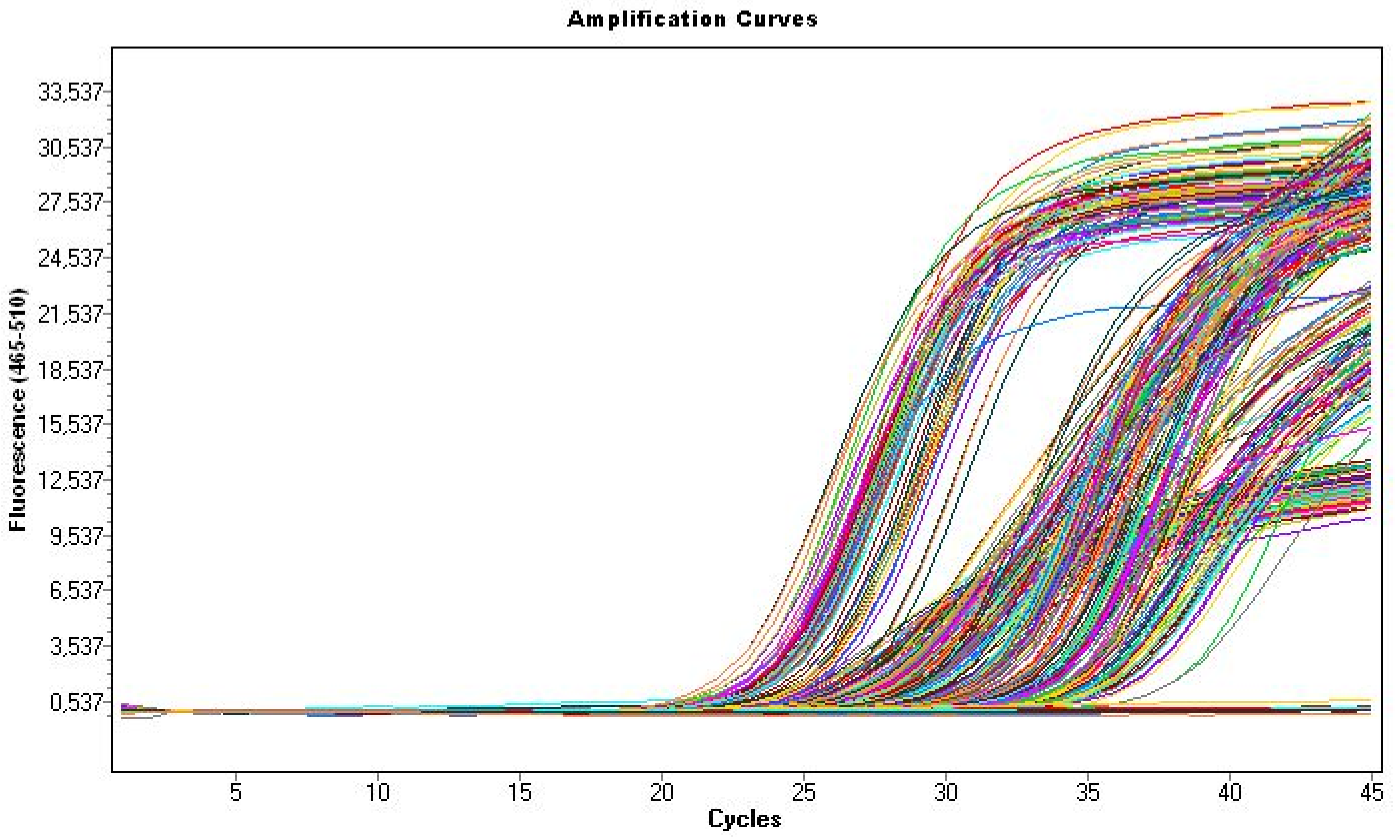
Amplification Curves
Fluorescence (465-510)
33,537
30,537
27,537
24,537
21,537
18,537
15,537
12,537
9,537
6,537
3,537
0,537
5
10
15
20
25
30
35
40
45
Cycles
