# Supplementary material for: MicroRNAs and Their Inhibition in Modulating SLC5A8 Expression in the Context of Papillary Thyroid Carcinoma
Source: Int J Mol Sci. 2025 Aug 15;26(16):7889. doi: 10.3390/ijms26167889 (PMC12386254; doi:10.3390/ijms26167889)
Supplement: Supplementary file 1 [file ijms-26-07889-s001.zip › ijms-3558049-supplementary/Manuscript data/Fig4 data/2013-10-07 miRy płytki 3-4.PDF]

Wojtek_2013-10-07 miRy plytki 3-4

Programs

Program Name	pre-incubation		
Cycles	1	Analysis Mode	None

Target (°C)	Acquisition Mode	Hold (hh:mm:ss)	Ramp Rate (°C/s)	Acquisitions (per °C)	Sec Target (°C)	Step size (°C)	Step Delay (cycles)
95	None	00:10:00	4,80		0	0	0

Program Name	amplification		
Cycles	45	Analysis Mode	Quantification

Target (°C)	Acquisition Mode	Hold (hh:mm:ss)	Ramp Rate (°C/s)	Acquisitions (per °C)	Sec Target (°C)	Step size (°C)	Step Delay (cycles)
95	None	00:00:10	4,80		0	0	0
60	Single	00:00:30	2,50		0	0	0
72	None	00:00:01	4,80		0	0	0

Program Name	cooling		
Cycles	1	Analysis Mode	None

Target (°C)	Acquisition Mode	Hold (hh:mm:ss)	Ramp Rate (°C/s)	Acquisitions (per °C)	Sec Target (°C)	Step size (°C)	Step Delay (cycles)
40	None	00:00:30	2,50		0	0	0

Abs Quant/2nd Derivative Max for All (Abs Quant/2nd Derivative Max)

Statistics

Samples	Mean Cp	Std Cp	Mean conc	Std conc
A1, A2, B1	27,86	0,23		
A3, A4, B3	26,44	0,19		
A5, A6, B5	26,64	0,10		
A7, A8, B7	26,71	0,14		
A10, A9, B9	26,91	0,25		
A11, A12, B11	26,31	0,46		
A13, A14, B13	27,15	0,17		
A15, A16, B15	26,87	0,03		
A17, A18, B17	26,14	0,12		
A19, A20, B19	27,39	0,19		
A21, A22, B21	27,05	0,05		
A23, A24, B23	27,90	0,12		
B2, C1, C2	25,42	0,28		
B4, C3, C4	26,10	0,40		

Statistics

Samples	Mean Cp	Std Cp	Mean conc	Std conc
B6, C5, C6	25,89	0,27		
B8, C7, C8	30,77	0,08		
B10, C10, C9	25,89	0,26		
B12, C11, C12	26,41	0,21		
B14, C13, C14	26,27	0,50		
B16, C15, C16	25,33	0,33		
B18, C17, C18	25,49	0,27		
B20, C19, C20	25,42	0,10		
B22, C21, C22	25,73	0,11		
B24, C23, C24	24,75	0,36		
D1, D2, E1	26,90	0,02		
D3, D4, E3	23,74	0,18		
D5, D6, E5	25,17	0,08		
D7, D8, E7	24,16	0,59		
D10, D9, E9	24,96	0,30		
D11, D12, E11	24,16	0,18		
D13, D14, E13	26,50	0,13		
D15, D16, E15	27,13	0,14		
D17, D18, E17	26,14	0,75		
D19, D20, E19	27,51	0,34		
D21, D22, E21	25,45	0,14		
D23, D24, E23	26,69	0,13		
E2, F1, F2	24,66	0,48		
E4, F3, F4	25,76	0,08		
E6, F5, F6	22,94	0,31		
E8, F7, F8	26,51	0,33		
E10, F10, F9	23,74	0,07		
E12, F11, F12	24,70	0,55		
E14, F13, F14	23,85	0,20		
E16, F15, F16	23,42	0,41		
E18, F17, F18	25,06	0,12		
E20, F19, F20	23,23	0,05		
E22, F21, F22	23,45	0,41		
E24, F23, F24	23,59	0,20		
G1, G2, H1	31,06	0,22		
G3, G4, H3	28,50	0,63		
G5, G6, H5	28,14	0,16		

Statistics

Samples	Mean Cp	Std Cp	Mean conc	Std conc
G7, G8, H7	28,83	0,22		
G10, G9, H9	28,44	0,22		
G11, G12, H11	29,27	0,26		
G13, G14, H13	30,39	0,21		
G15, G16, H15	31,23	0,03		
G17, G18, H17	29,41	0,32		
G19, G20, H19	31,21	0,12		
G21, G22, H21	29,58	0,25		
G23, G24, H23	32,12	0,10		
H2, I1, I2	27,52	0,40		
H4, I3, I4	28,45	0,19		
H6, I5, I6	26,84	0,26		
H8, I7, I8	31,31	0,07		
H10, I10, I9	26,85	0,08		
H12, I11, I12	28,84	0,50		
H14, I13, I14	27,19	0,33		
H16, I15, I16	28,33	0,33		
H18, I17, I18	27,00	0,10		
H20, I19, I20	26,98	0,15		
H22, I21, I22	27,80	0,37		
H24, I23, I24	27,90	0,23		
J1, J2, K1	37,70	0,10		
J3, J4, K3	34,96	0,21		
J5, J6, K5	34,62	0,17		
J7, J8, K7	34,78	0,29		
J10, J9, K9	34,38	0,09		
J11, J12, K11	34,25	0,60		
J13, J14, K13	36,72	0,37		
J15, J16, K15	37,87	1,13		
J17, J18, K17	34,12	0,15		
J19, J20, K19	34,65	0,34		
J21, J22, K21	33,81	0,34		
J23, J24, K23	35,71	0,48		
K2, L1, L2	35,87	0,40		
K4, L3, L4	38,02	1,32		
K6, L5, L6	35,46	0,42		
K8, L7, L8	40,00			

Statistics

Samples	Mean Cp	Std Cp	Mean conc	Std conc
K10, L10, L9	35,34	0,33		
K12, L11, L12	34,75	0,35		
K14, L13, L14	34,90	0,29		
K16, L15, L16	35,52	0,16		
K18, L17, L18	35,01	0,46		
K20, L19, L20	34,54	0,22		
K22, L21, L22	35,29	0,10		
K24, L23, L24	34,93	0,30		
M1, M2, N1	35,43	0,38		
M3, M4, N3	32,52	0,17		
M5, M6, N5	35,42	0,12		
M7, M8, N7	33,73	0,04		
M10, M9, N9	34,43	0,27		
M11, M12, N11	36,28	0,65		
M13, M14, N13	35,72	0,06		
M15, M16, N15	35,46	0,19		
M17, M18, N17	34,46	0,23		
M19, M20, N19	34,72	0,17		
M21, M22, N21	34,71	0,40		
M23, M24, N23	34,47	0,22		
N2, O1, O2	31,72	0,04		
N4, O3, O4				
N6, O5, O6	31,98	0,14		
N8, O7, O8	30,68	0,26		
N10, O10, O9	32,46	0,16		
N12, O11, O12	32,72	0,15		
N14, O13, O14	29,38	0,31		
N16, O15, O16	30,51	0,02		
N18, O17, O18	33,08	0,16		
N20, O19, O20	32,45	0,11		
N22, O21, O22	30,69	0,04		
N24, O23, O24	30,53	0,21		

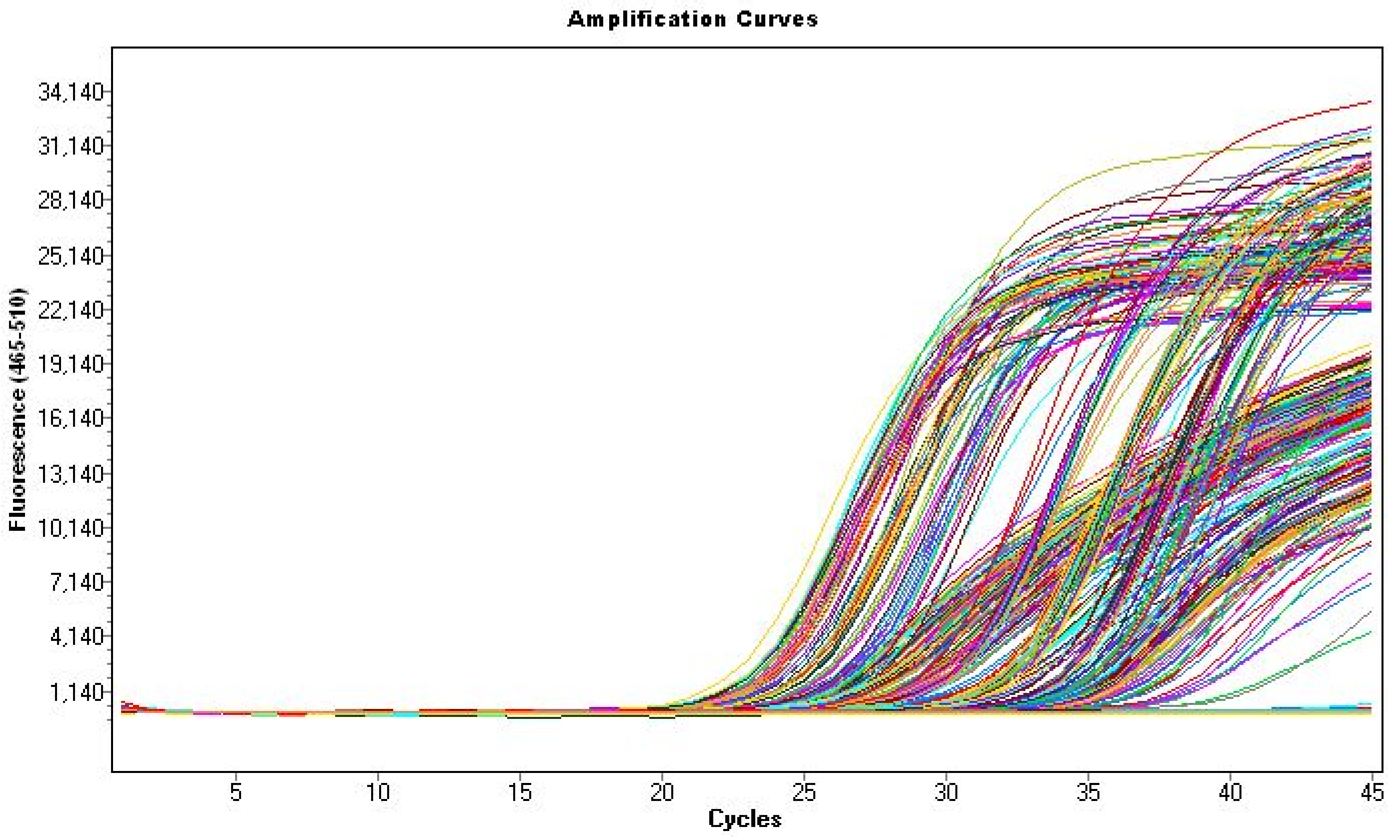
Amplification Curves
34,140
31,140
28,140
25,140
22,140
19,140
16,140
13,140
10,140
7,140
4,140
1,140
Fluorescence (465-510)
5
10
15
20
25
30
35
40
45
Cycles
