# Supplementary material for: MicroRNAs and Their Inhibition in Modulating SLC5A8 Expression in the Context of Papillary Thyroid Carcinoma
Source: Int J Mol Sci. 2025 Aug 15;26(16):7889. doi: 10.3390/ijms26167889 (PMC12386254; doi:10.3390/ijms26167889)

Roche

Wojtek_2013-10-15 miRy w parach 5

Programs

Program Name	pre-incubation		
Cycles	1	Analysis Mode	None

Target (°C)	Acquisition Mode	Hold (hh:mm:ss)	Ramp Rate (°C/s)	Acquisitions (per °C)	Sec Target (°C)	Step size (°C)	Step Delay (cycles)
95	None	00:10:00	4,80		0	0	0

Program Name	amplification		
Cycles	45	Analysis Mode	Quantification

Target (°C)	Acquisition Mode	Hold (hh:mm:ss)	Ramp Rate (°C/s)	Acquisitions (per °C)	Sec Target (°C)	Step size (°C)	Step Delay (cycles)
95	None	00:00:10	4,80		0	0	0
60	Single	00:00:30	2,50		0	0	0
72	None	00:00:01	4,80		0	0	0

Program Name	cooling		
Cycles	1	Analysis Mode	None

Target (°C)	Acquisition Mode	Hold (hh:mm:ss)	Ramp Rate (°C/s)	Acquisitions (per °C)	Sec Target (°C)	Step size (°C)	Step Delay (cycles)
40	None	00:00:30	2,50		0	0	0

Abs Quant/2nd Derivative Max for All (Abs Quant/2nd Derivative Max)

Statistics

Samples	Mean Cp	Std Cp	Mean conc	Std conc
A1, A2, B1	26,43	0,20		
A3, A4, B3	25,73	0,11		
A5, A6, B5	25,29	0,22		
A7, A8, B7	23,80	0,11		
A10, A9, B9	24,69	0,19		
A11, A12, B11	24,74	0,05		
A13, A14, B13	26,02	0,20		
A15, A16, B15	25,60	0,04		
A17, A18, B17	25,33	0,50		
A19, A20, B19	25,36	0,24		
A21, A22, B21	23,28	0,13		
A23, A24, B23	24,10	0,06		
B2, C1, C2	24,91	0,28		
B4, C3, C4	23,52	0,19		

Statistics

Samples	Mean Cp	Std Cp	Mean conc	Std conc
B6, C5, C6	25,38	0,27		
B8, C7, C8	34,95	3,50		
B10, C10, C9	24,30	0,22		
B12, C11, C12	24,25	0,23		
B14, C13, C14	24,76	0,17		
B16, C15, C16	24,66	0,20		
B18, C17, C18	23,26	0,18		
B20, C19, C20	23,23	0,15		
B22, C21, C22	23,81	0,22		
B24, C23, C24	23,95	0,24		
D1, D2, E1	29,02	0,18		
D3, D4, E3	28,04	0,21		
D5, D6, E5	28,81	0,19		
D7, D8, E7	27,82	0,24		
D10, D9, E9	28,81	0,17		
D11, D12, E11	28,45	0,33		
D13, D14, E13	28,18	0,17		
D15, D16, E15	29,05	0,14		
D17, D18, E17	27,41	0,47		
D19, D20, E19	28,15	0,25		
D21, D22, E21	27,13	0,67		
D23, D24, E23	28,64	0,26		
E2, F1, F2	34,53	0,20		
E4, F3, F4	33,80	0,15		
E6, F5, F6	35,04	0,18		
E8, F7, F8	34,87	0,24		
E10, F10, F9	33,22	0,54		
E12, F11, F12	33,10	0,11		
E14, F13, F14	34,94	0,19		
E16, F15, F16	34,66	0,26		
E18, F17, F18	34,81	0,74		
E20, F19, F20	34,50	0,57		
E22, F21, F22	34,81	0,65		
E24, F23, F24	35,18	0,64		
G1, G2, H1	33,70	0,06		
G3, G4, H3	31,35	0,16		
G5, G6, H5	34,17	0,27		

Statistics

Samples	Mean Cp	Std Cp	Mean conc	Std conc
G7, G8, H7	33,17	0,31		
G10, G9, H9	31,54	0,09		
G11, G12, H11	28,12	0,14		
G13, G14, H13	31,22	0,09		
G15, G16, H15	32,45	0,24		
G17, G18, H17	33,00	0,32		
G19, G20, H19	32,80	0,08		
G21, G22, H21	31,82	0,21		
G23, G24, H23	33,31	0,12		
I19, I20, I21				
I22, I23, I24				

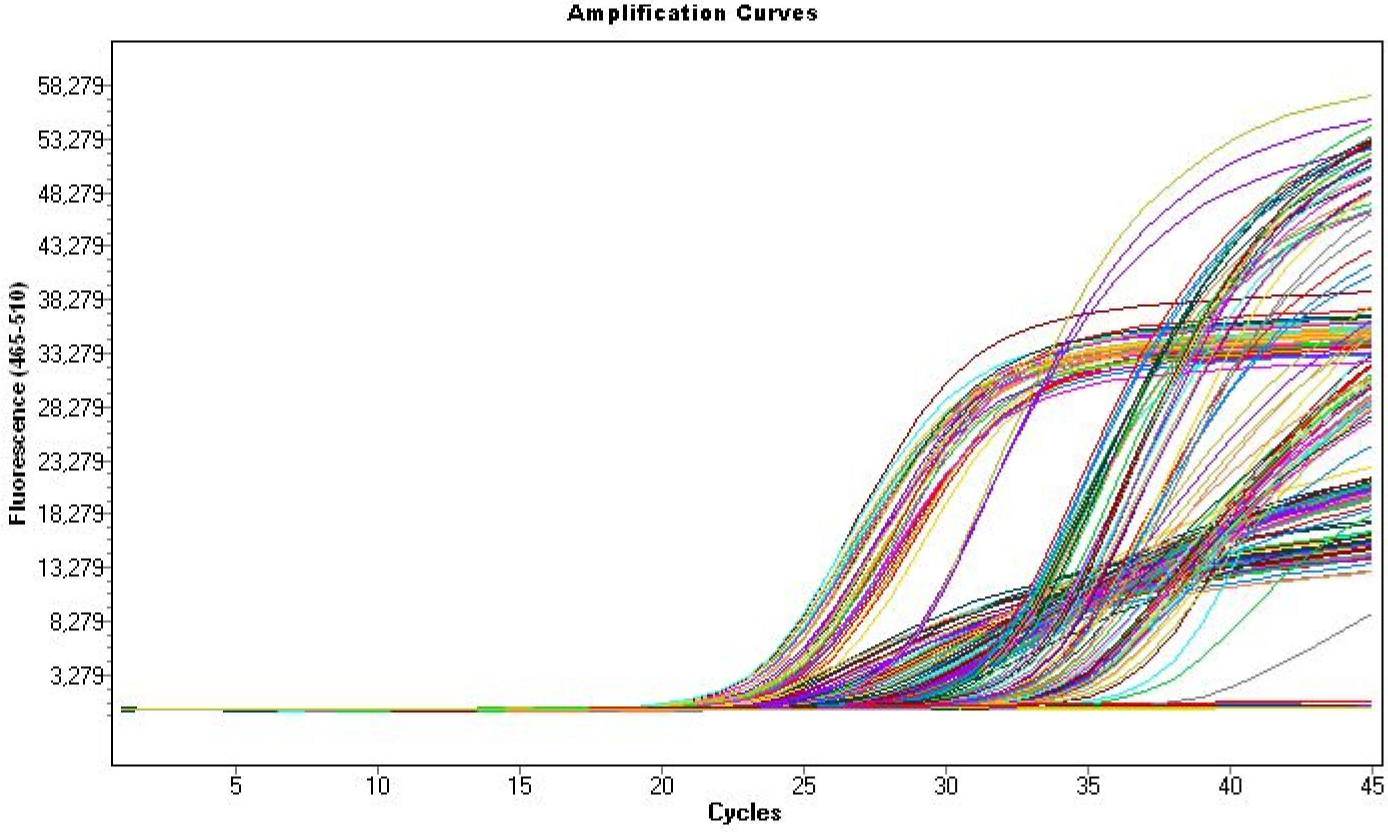

Supplement: Supplementary file 1 [file ijms-26-07889-s001.zip › ijms-3558049-supplementary/Manuscript data/Fig4 data/2013-10-15 miRy płytka 5.PDF]
