# Supplementary material for: MicroRNAs and Their Inhibition in Modulating SLC5A8 Expression in the Context of Papillary Thyroid Carcinoma
Source: Int J Mol Sci. 2025 Aug 15;26(16):7889. doi: 10.3390/ijms26167889 (PMC12386254; doi:10.3390/ijms26167889)

Wojtek_2013-10-24 miRy w parach 1534-1579

Programs

Program Name	pre-incubation		
Cycles	1	Analysis Mode	None

Target (°C)	Acquisition Mode	Hold (hh:mm:ss)	Ramp Rate (°C/s)	Acquisitions (per °C)	Sec Target (°C)	Step size (°C)	Step Delay (cycles)
95	None	00:10:00	4,80		0	0	0

Program Name	amplification		
Cycles	55	Analysis Mode	Quantification

Target (°C)	Acquisition Mode	Hold (hh:mm:ss)	Ramp Rate (°C/s)	Acquisitions (per °C)	Sec Target (°C)	Step size (°C)	Step Delay (cycles)
95	None	00:00:10	4,80		0	0	0
60	Single	00:00:30	2,50		0	0	0
72	None	00:00:01	4,80		0	0	0

Program Name	cooling		
Cycles	1	Analysis Mode	None

Target (°C)	Acquisition Mode	Hold (hh:mm:ss)	Ramp Rate (°C/s)	Acquisitions (per °C)	Sec Target (°C)	Step size (°C)	Step Delay (cycles)
40	None	00:00:30	2,50		0	0	0

Abs Quant/2nd Derivative Max for All (Abs Quant/2nd Derivative Max)

Statistics

Samples	Mean Cp	Std Cp	Mean conc	Std conc
A1, A2, A3	27,34	0,05		
A4, A5, A6	28,38	0,17		
B1, B2, B3	34,08	0,21		
B4, B5, B6	32,70	0,15		
C1, C2, C3	27,30	0,12		
C4, C5, C6	30,18	0,36		
D1, D2, D3	37,07	0,04		
D4, D5, D6	34,49	0,06		
E1, E2, E3	26,00	0,18		
E4, E5, E6	28,05	0,15		
F1, F2, F3	34,73	0,15		
F4, F5, F6	34,38	0,17		
G1, G2, G3	26,60	0,26		
G4, G5, G6	27,99	0,07		

Statistics

Samples	Mean Cp	Std Cp	Mean conc	Std conc
H1, H2, H3	36,10	0,22		
H4, H5, H6	35,73	0,12		
I1, I2, I3	26,37	0,22		
I4, I5, I6	27,75	0,04		
J1, J2, J3	34,97	0,26		
J4, J5, J6	34,44	0,17		
K1, K2, K3	25,61	0,12		
K4, K5, K6	27,96	0,08		
L1, L2, L3	35,92	0,45		
L4, L5, L6	34,09	0,23		
M1, M2, M3	26,68	0,09		
M4, M5, M6	29,75	0,06		
N1, N2, N3	37,03	0,70		
N4, N5, N6	35,80	0,30		
O1, O2, O3	27,21	1,37		
O4, O5, O6	29,34	0,18		
P1, P2, P3	36,41	0,45		
P4, P5, P6	35,39	0,14		

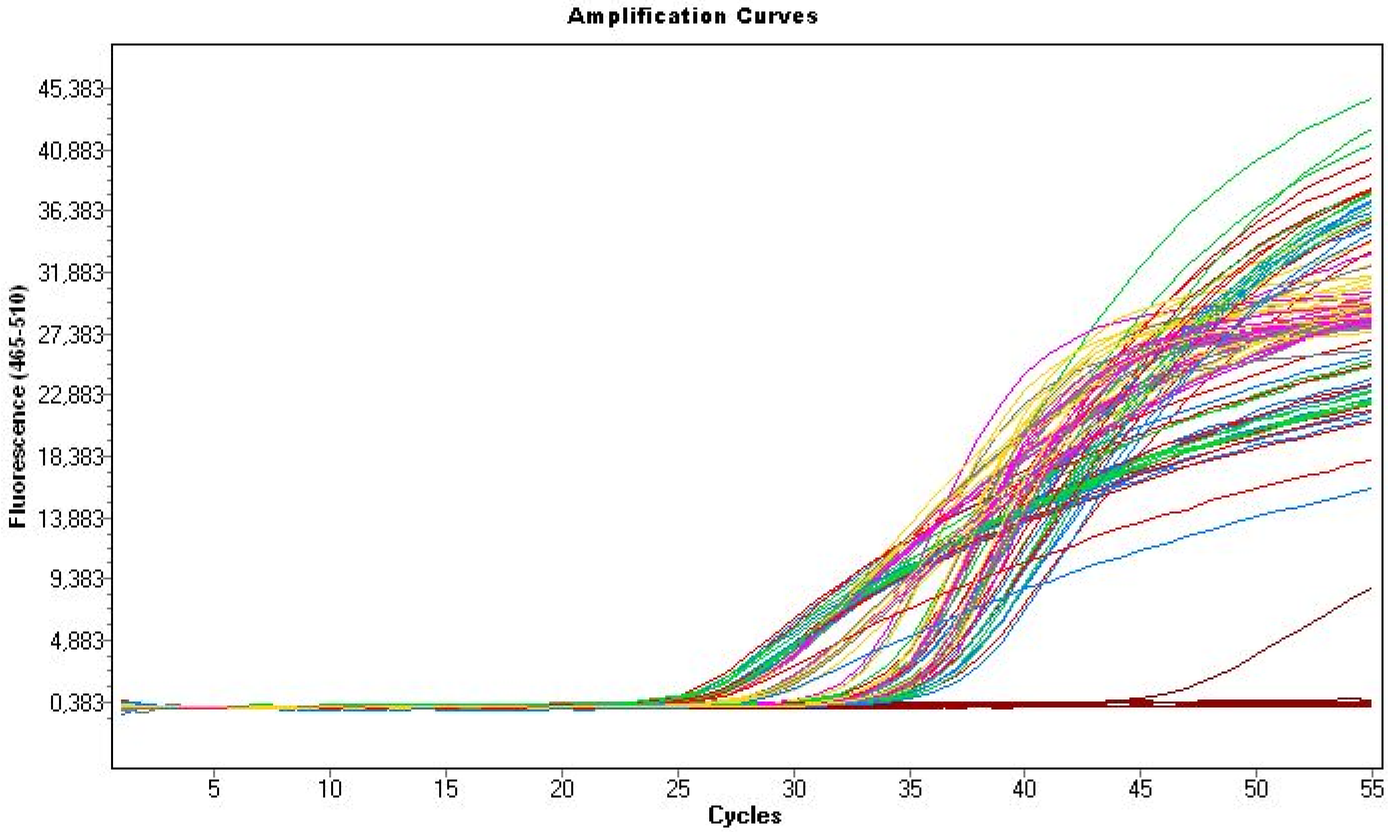

Supplement: Supplementary file 1 [file ijms-26-07889-s001.zip › ijms-3558049-supplementary/Manuscript data/Fig4 data/2013-10-24 miRy w parach 1534-1579.PDF]
