# Supplementary material for: MicroRNAs and Their Inhibition in Modulating SLC5A8 Expression in the Context of Papillary Thyroid Carcinoma
Source: Int J Mol Sci. 2025 Aug 15;26(16):7889. doi: 10.3390/ijms26167889 (PMC12386254; doi:10.3390/ijms26167889)

Roche

Wojtek_2013-10-31 miRy w parach 1507-10919

Programs

Program Name	pre-incubation		
Cycles	1	Analysis Mode	None

Target (°C)	Acquisition Mode	Hold (hh:mm:ss)	Ramp Rate (°C/s)	Acquisitions (per °C)	Sec Target (°C)	Step size (°C)	Step Delay (cycles)
95	None	00:10:00	4,80		0	0	0

Program Name	amplification		
Cycles	50	Analysis Mode	Quantification

Target (°C)	Acquisition Mode	Hold (hh:mm:ss)	Ramp Rate (°C/s)	Acquisitions (per °C)	Sec Target (°C)	Step size (°C)	Step Delay (cycles)
95	None	00:00:10	4,80		0	0	0
60	Single	00:00:30	2,50		0	0	0
72	None	00:00:01	4,80		0	0	0

Program Name	cooling		
Cycles	1	Analysis Mode	None

Target (°C)	Acquisition Mode	Hold (hh:mm:ss)	Ramp Rate (°C/s)	Acquisitions (per °C)	Sec Target (°C)	Step size (°C)	Step Delay (cycles)
40	None	00:00:30	2,50		0	0	0

Abs Quant/2nd Derivative Max for All (Abs Quant/2nd Derivative Max)

Statistics

Samples	Mean Cp	Std Cp	Mean conc	Std conc
A1, A2, A3	25,23	0,42		
A4, A5, A6	28,79	0,24		
A7, A8, A9	32,76	0,12		
A10, A11, A12	35,17	0,30		
B1, B2, B3	27,86	0,21		
B4, B5, B6	30,80	0,31		
B7, B8, B9	35,06	0,28		
B10, B11, B12	34,31	0,21		
C1, C2, C3	27,52	0,40		
C4, C5, C6	31,61	0,09		
C7, C8, C9	36,22	0,41		
C10, C11, C12	34,61	0,05		
D1, D2, D3	27,27	0,11		
D4, D5, D6	29,94	0,19		

Statistics

Samples	Mean Cp	Std Cp	Mean conc	Std conc
D7, D8, D9	35,64	0,42		
D10, D11, D12	35,75	0,45		
E1, E2, E3	26,92	0,45		
E4, E5, E6	29,88	0,19		
E7, E8, E9	35,27	0,75		
E10, E11, E12	34,86	0,32		
F1, F2, F3	28,13	0,17		
F4, F5, F6	32,07	0,28		
F7, F8, F9	37,96	0,40		
F10, F11, F12	34,37	0,16		
G1, G2, G3	27,84	0,33		
G4, G5, G6	31,22	0,33		
G7, G8, G9	36,36	0,19		
G10, G11, G12	35,97	0,20		
H1, H2, H3	26,66	0,36		
H4, H5, H6	31,91	0,32		
H7, H8, H9	36,56	0,18		
H10, H11, H12	35,50	0,22		
I1, I2, I3	26,03	0,42		
I4, I5, I6	29,98	0,04		
I7, I8, I9	34,54	0,26		
I10, I11, I12	34,65	0,34		
J1, J2, J3	26,67	0,19		
J4, J5, J6	34,22	0,27		
J7, J8, J9	39,44	0,93		
J10, J11, J12	36,72	0,26		
K1, K2, K3	25,28	0,47		
K4, K5, K6	29,46	0,31		
K7, K8, K9	34,48	0,48		
K10, K11, K12	34,83	0,40		
L1, L2, L3	26,38	0,36		
L4, L5, L6	30,39	0,14		
L7, L8, L9	34,57	0,34		
L10, L11, L12	32,70	0,19		
M1, M2, M3	28,24	0,20		
M4, M5, M6	32,34	0,24		
M7, M8, M9	36,93	0,44		

Statistics

Samples	Mean Cp	Std Cp	Mean conc	Std conc
M10, M11, M12	36,72	0,46		
N1, N2, N3	24,88	0,39		
N4, N5, N6	27,48	0,33		
N7, N8, N9	31,79	0,27		
N10, N11, N12	33,06	0,35		
O1, O2, O3	26,79	0,47		
O4, O5, O6	31,56	0,15		
O7, O8, O9	36,60	0,31		
O10, O11, O12	35,75	0,13		
P1, P2, P3	25,53	0,30		
P4, P5, P6	29,23	0,34		
P7, P8, P9	33,66	0,05		
P10, P11, P12	35,10	0,35		

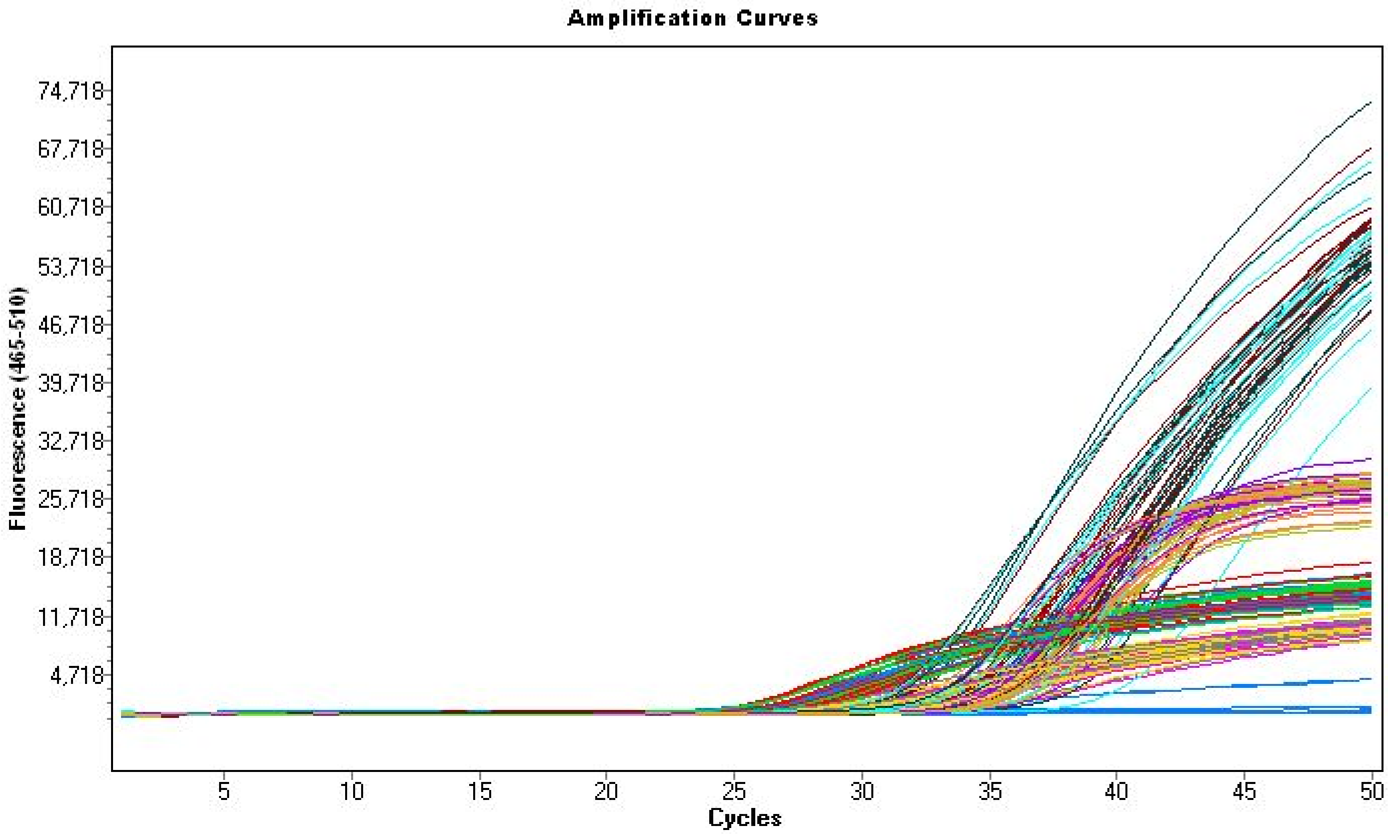

Supplement: Supplementary file 1 [file ijms-26-07889-s001.zip › ijms-3558049-supplementary/Manuscript data/Fig4 data/2013-10-31 miRy w parach 1507-10919.PDF]
