# Supplementary material for: MicroRNAs and Their Inhibition in Modulating SLC5A8 Expression in the Context of Papillary Thyroid Carcinoma
Source: Int J Mol Sci. 2025 Aug 15;26(16):7889. doi: 10.3390/ijms26167889 (PMC12386254; doi:10.3390/ijms26167889)
Supplement: Supplementary file 1 [file ijms-26-07889-s001.zip › ijms-3558049-supplementary/Manuscript data/Fig4 data/2013-11-13 miRy w parach 548-10001143.PDF]

LightCycler® 480 Software Report

Wojtek_2013-11-13 miRy w parach 548-10001142

Programs

Program Name	pre-incubation		
Cycles	1	Analysis Mode	None

Target (°C)	Acquisition Mode	Hold (hh:mm:ss)	Ramp Rate (°C/s)	Acquisitions (per °C)	Sec Target (°C)	Step size (°C)	Step Delay (cycles)
95	None	00:10:00	4,80		0	0	0

Program Name	amplification		
Cycles	50	Analysis Mode	Quantification

Target (°C)	Acquisition Mode	Hold (hh:mm:ss)	Ramp Rate (°C/s)	Acquisitions (per °C)	Sec Target (°C)	Step size (°C)	Step Delay (cycles)
95	None	00:00:10	4,80		0	0	0
60	Single	00:00:30	2,50		0	0	0
72	None	00:00:01	4,80		0	0	0

Program Name	cooling		
Cycles	1	Analysis Mode	None

Target (°C)	Acquisition Mode	Hold (hh:mm:ss)	Ramp Rate (°C/s)	Acquisitions (per °C)	Sec Target (°C)	Step size (°C)	Step Delay (cycles)
40	None	00:00:30	2,50		0	0	0

Abs Quant/2nd Derivative Max for All (Abs Quant/2nd Derivative Max)

Statistics

Samples	Mean Cp	Std Cp	Mean conc	Std conc
A1, A2, A3	25,80	0,33		
A4, A5, A6	27,35	0,28		
A7, A8, A9	33,95	0,21		
A10, A11, A12	33,15	0,17		
A13, A14, A15	26,49	0,53		
A16, A17, A18	30,96	0,75		
A19, A20, A21	39,91	1,38		
A22, A23, A24	36,48	0,61		
B1, B2, B3	26,09	0,16		
B4, B5, B6	27,57	0,23		
B7, B8, B9	34,46	0,27		
B10, B11, B12	34,10	0,18		
B13, B14, B15	26,25	0,52		
B16, B17, B18	30,55	1,81		

Statistics

Samples	Mean Cp	Std Cp	Mean conc	Std conc
B19, B20, B21	34,92	0,88		
B22, B23, B24	35,91	0,41		
C1, C2, C3	25,30	0,39		
C4, C5, C6	28,75	2,34		
C7, C8, C9	35,39	0,36		
C10, C11, C12	34,60	0,25		
C13, C14, C15	26,58	0,31		
C16, C17, C18	28,71	0,24		
C19, C20, C21	34,87	0,19		
C22, C23, C24	32,56	0,21		
D1, D2, D3	26,32	0,07		
D4, D5, D6	28,98	0,14		
D7, D8, D9	34,94	0,17		
D10, D11, D12	34,06	0,26		
D13, D14, D15	26,38	0,51		
D16, D17, D18	29,32	0,37		
D19, D20, D21	35,33	0,54		
D22, D23, D24	35,85	0,16		
E1, E2, E3	24,56	0,43		
E4, E5, E6	26,41	0,33		
E7, E8, E9	32,07	0,29		
E10, E11, E12	34,38	0,14		
E13, E14, E15	24,89	0,47		
E16, E17, E18	27,65	0,55		
E19, E20, E21	30,61	0,31		
E22, E23, E24	32,80	0,19		
F1, F2, F3	27,75	0,32		
F4, F5, F6	28,96	0,27		
F7, F8, F9	35,88	0,51		
F10, F11, F12	31,91	0,18		
F13, F14, F15	25,98	0,38		
F16, F17, F18	29,68	0,32		
F19, F20, F21	34,71	0,46		
F22, F23, F24	34,35	0,25		
G1, G2, G3	26,79	0,35		
G4, G5, G6	28,94	0,18		
G7, G8, G9	34,69	0,44		

Statistics

Samples	Mean Cp	Std Cp	Mean conc	Std conc
G10, G11, G12	33,93	0,23		
G13, G14, G15	26,49	0,61		
G16, G17, G18	29,20	0,47		
G19, G20, G21	35,60	0,22		
G22, G23, G24	33,79	0,51		
H1, H2, H3	25,61	0,26		
H4, H5, H6	27,39	0,26		
H7, H8, H9	33,59	0,23		
H10, H11, H12	33,77	0,14		
H13, H14, H15	25,65	0,58		
H16, H17, H18	29,99	0,25		
H19, H20, H21	36,05	0,19		
H22, H23, H24	35,46	0,25		
I1, I2, I3	25,01	0,33		
I4, I5, I6	27,98	0,44		
I7, I8, I9	32,29	0,46		
I10, I11, I12	32,60	0,31		
I13, I14, I15	26,02	0,42		
I16, I17, I18	28,33	0,21		
I19, I20, I21	33,51	0,10		
I22, I23, I24	35,40	0,41		
J1, J2, J3	26,60	0,15		
J4, J5, J6	27,47	0,22		
J7, J8, J9	34,33	0,29		
J10, J11, J12	32,37	0,23		
J13, J14, J15	26,72	0,42		
J16, J17, J18	29,61	0,39		
J19, J20, J21	33,87	0,33		
J22, J23, J24	34,19	0,34		
K1, K2, K3	26,00	0,31		
K4, K5, K6	28,84	0,20		
K7, K8, K9	35,04	0,40		
K10, K11, K12	35,07	0,35		
K13, K14, K15	26,35	0,42		
K16, K17, K18	28,22	0,35		
K19, K20, K21	34,40	0,19		
K22, K23, K24	35,17	0,21		

Statistics

Samples	Mean Cp	Std Cp	Mean conc	Std conc
L1, L2, L3	25,92	0,18		
L4, L5, L6	27,46	0,25		
L7, L8, L9	33,73	0,19		
L10, L11, L12	34,19	0,32		
L13, L14, L15	26,05	0,24		
L16, L17, L18	29,70	0,28		
L19, L20, L21	33,85	0,35		
L22, L23, L24	35,05	0,09		
M1, M2, M3	26,64	0,25		
M4, M5, M6	28,14	0,22		
M7, M8, M9	33,73	0,40		
M10, M11, M12	33,43	0,19		
M13, M14, M15	28,76	0,43		
M16, M17, M18	29,78	0,15		
M19, M20, M21	35,72	0,19		
M22, M23, M24	31,29	0,20		
N1, N2, N3	27,36	0,24		
N4, N5, N6	27,70	0,15		
N7, N8, N9	34,92	0,22		
N10, N11, N12	32,22	0,33		
N14, N15	30,69			
N17, N18				
N20, N21				
N23, N24				
O1, O2, O3	26,06	0,37		
O4, O5, O6	28,03	0,15		
O7, O8, O9	34,40	0,38		
O10, O11, O12	34,04	0,21		
O13, O14, O15	27,31	0,31		
O16, O17, O18	28,57	0,19		
O19, O20, O21	34,80	0,37		
O22, O23, O24	35,86	0,53		
P1, P2, P3	27,82	0,25		
P4, P5, P6	29,60	0,23		
P7, P8, P9	35,58	0,27		
P10, P11, P12	31,85	0,15		

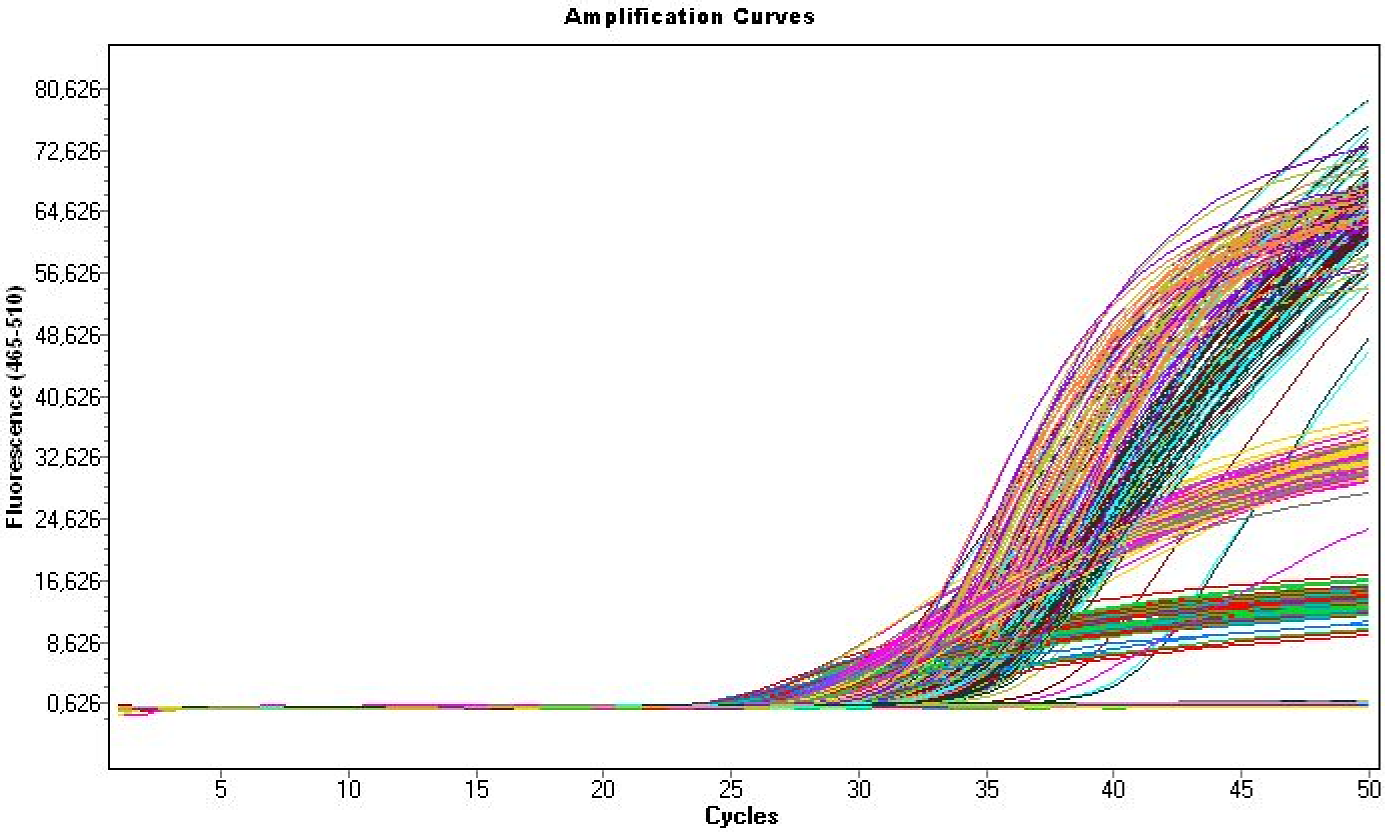
Amplification Curves
Fluorescence (465-510)
80,626
72,626
64,626
56,626
48,626
40,626
32,626
24,626
16,626
8,626
0,626
5
10
15
20
25
30
35
40
45
50
Cycles
