# Supplementary material for: MicroRNAs and Their Inhibition in Modulating SLC5A8 Expression in the Context of Papillary Thyroid Carcinoma
Source: Int J Mol Sci. 2025 Aug 15;26(16):7889. doi: 10.3390/ijms26167889 (PMC12386254; doi:10.3390/ijms26167889)

Roche

Wojtek_2013-11-14 miRy dorobki

Programs

Program Name	pre-incubation		
Cycles	1	Analysis Mode	None

Target (°C)	Acquisition Mode	Hold (hh:mm:ss)	Ramp Rate (°C/s)	Acquisitions (per °C)	Sec Target (°C)	Step size (°C)	Step Delay (cycles)
95	None	00:10:00	4,80		0	0	0

Program Name	amplification		
Cycles	50	Analysis Mode	Quantification

Target (°C)	Acquisition Mode	Hold (hh:mm:ss)	Ramp Rate (°C/s)	Acquisitions (per °C)	Sec Target (°C)	Step size (°C)	Step Delay (cycles)
95	None	00:00:10	4,80		0	0	0
60	Single	00:00:30	2,50		0	0	0
72	None	00:00:01	4,80		0	0	0

Program Name	cooling		
Cycles	1	Analysis Mode	None

Target (°C)	Acquisition Mode	Hold (hh:mm:ss)	Ramp Rate (°C/s)	Acquisitions (per °C)	Sec Target (°C)	Step size (°C)	Step Delay (cycles)
40	None	00:00:30	2,50		0	0	0

Abs Quant/2nd Derivative Max for All (Abs Quant/2nd Derivative Max)

Statistics

Samples	Mean Cp	Std Cp	Mean conc	Std conc
K16, L16, M16	27,47	0,44		
K17, L17, M17				
K18, L18, M18	41,95	2,95		
K19, L19, M19	30,75	0,26		
K20, L20, M20	35,42	0,23		
K21, L21, M21	32,25	0,23		
L22, M22				
L23, M23				
N16, O16, P16	27,84	0,10		
N17, O17, P17				
N18, O18, P18	35,18	0,63		
N19, O19, P19	33,56	0,24		
N20, O20, P20	35,62	0,26		
N21, O21, P21	33,70	0,07		

Statistics

Samples	Mean Cp	Std Cp	Mean conc	Std conc
O22, P22				

Amplification Curves

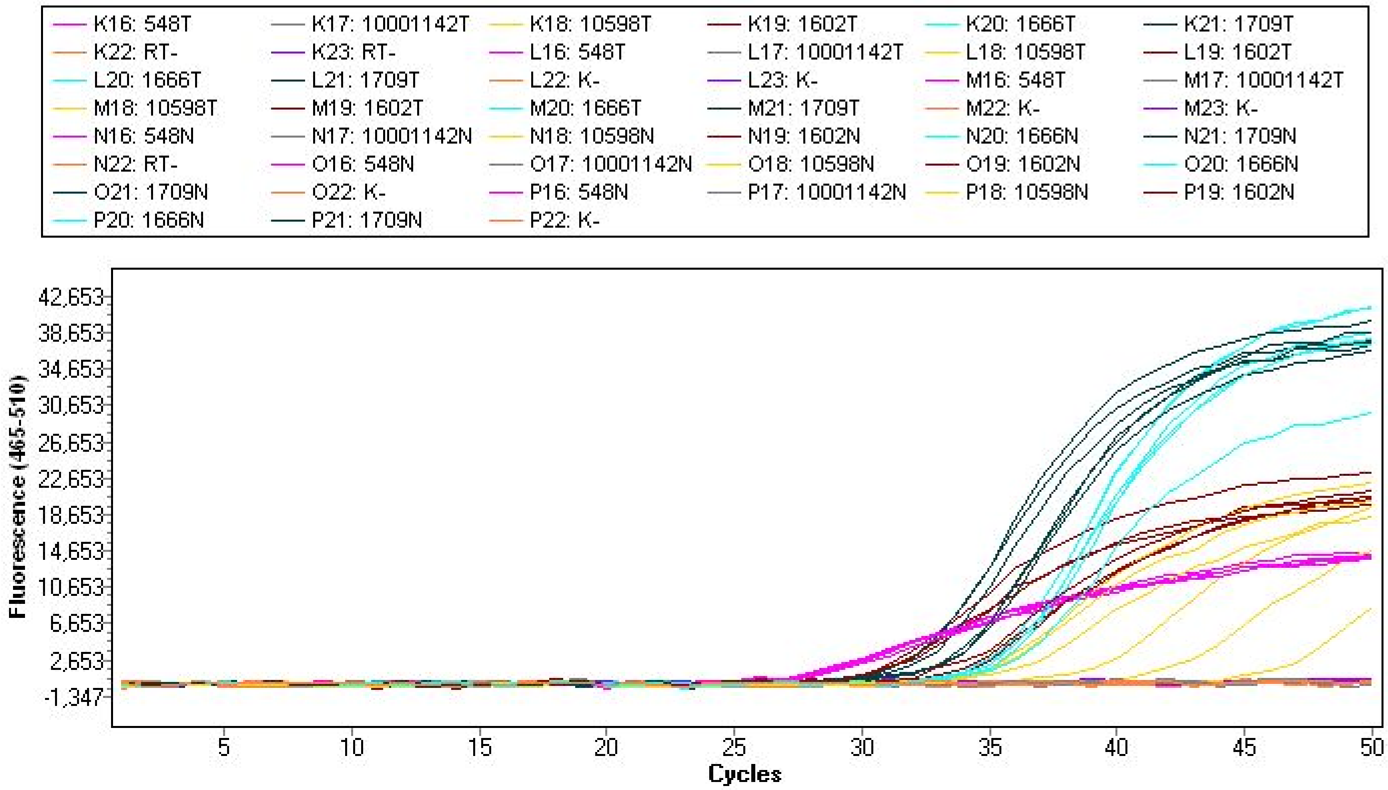

Supplement: Supplementary file 1 [file ijms-26-07889-s001.zip › ijms-3558049-supplementary/Manuscript data/Fig4 data/2013-11-14 miRy dorobki.PDF]
