# Supplementary material for: MicroRNAs and Their Inhibition in Modulating SLC5A8 Expression in the Context of Papillary Thyroid Carcinoma
Source: Int J Mol Sci. 2025 Aug 15;26(16):7889. doi: 10.3390/ijms26167889 (PMC12386254; doi:10.3390/ijms26167889)
Supplement: Supplementary file 1 [file ijms-26-07889-s001.zip › ijms-3558049-supplementary/Manuscript data/Fig4 data/2013-11-19 miRy dorobki.PDF]

Wojtek_2013-11-19 miRy dorobki

Programs

Program Name	pre-incubation		
Cycles	1	Analysis Mode	None

Target (°C)	Acquisition Mode	Hold (hh:mm:ss)	Ramp Rate (°C/s)	Acquisitions (per °C)	Sec Target (°C)	Step size (°C)	Step Delay (cycles)
95	None	00:10:00	4,80		0	0	0

Program Name	amplification		
Cycles	50	Analysis Mode	Quantification

Target (°C)	Acquisition Mode	Hold (hh:mm:ss)	Ramp Rate (°C/s)	Acquisitions (per °C)	Sec Target (°C)	Step size (°C)	Step Delay (cycles)
95	None	00:00:10	4,80		0	0	0
60	Single	00:00:30	2,50		0	0	0
72	None	00:00:01	4,80		0	0	0

Program Name	cooling		
Cycles	1	Analysis Mode	None

Target (°C)	Acquisition Mode	Hold (hh:mm:ss)	Ramp Rate (°C/s)	Acquisitions (per °C)	Sec Target (°C)	Step size (°C)	Step Delay (cycles)
40	None	00:00:30	2,50		0	0	0

Abs Quant/2nd Derivative Max for All (Abs Quant/2nd Derivative Max)

Statistics

Samples	Mean Cp	Std Cp	Mean conc	Std conc
A1, A2, A3	24,64	0,16		
A4, A5, A6	26,54	0,02		
A7, A8, A9	31,68	0,16		
A10, A11, A12	30,81	0,11		
B1, B2, B3	23,47	0,15		
B4, B5, B6	26,79	0,12		
B7, B8, B9	33,14	0,14		
B10, B11, B12	32,02	0,12		
C1, C2, C3	24,90	0,07		
C4, C5, C6	29,50	0,18		
C7, C8, C9	29,87	0,11		
C10, C11, C12	33,58	0,08		
D1, D2, D3	25,09	0,07		
D4, D5, D6	27,84	0,10		

Statistics

Samples	Mean Cp	Std Cp	Mean conc	Std conc
D7, D8, D9	32,04	0,09		
D10, D11, D12	34,33	0,42		
E1, E2, E3	30,53	0,12		
E4, E5, E6	33,78	0,23		
E7, E8, E9	35,38	0,49		
E10, E11, E12	37,57	0,44		
F1, F2, F3	29,06	0,13		
F4, F5, F6	33,36	0,32		
F7, F8, F9	37,55	0,71		
F10, F11, F12	37,58	0,21		
G1, G2, G3	24,15	0,18		
G4, G5, G6	29,76	0,58		
G7, G8, G9	37,65	0,49		
G10, G11, G12	34,85	0,28		
H1, H2, H3	24,59	0,18		
H4, H5, H6	27,85	0,20		
H7, H8, H9	33,07	0,26		
H10, H11, H12	30,90	0,15		
I1, I2, I3	25,05	0,12		
I4, I5, I6	29,13	0,13		
I7, I8, I9	33,70	0,26		
I10, I11, I12	34,94	0,18		
J1, J2, J3	25,57	0,05		
J4, J5, J6	29,20	0,15		
J7, J8, J9	34,76	0,57		
J10, J11, J12	35,35	0,18		
K2, K3	34,73			
K5, K6				
K8, K9				
K11, K12				

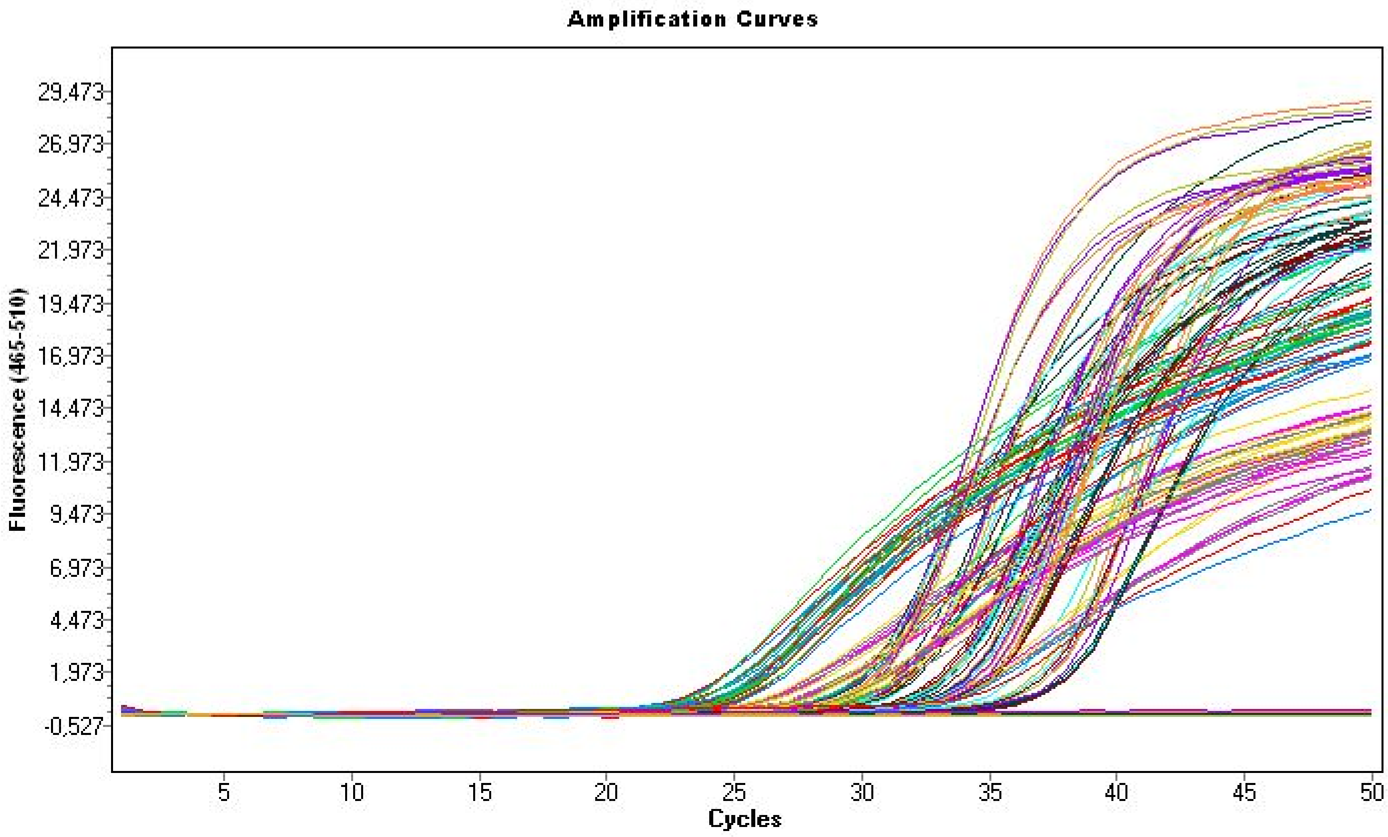
Amplification Curves
Fluorescence (465-510)
29,473
26,973
24,473
21,973
19,473
16,973
14,473
11,973
9,473
6,973
4,473
1,973
-0,527
5
10
15
20
25
30
35
40
45
50
Cycles
