# Supplementary material for: MicroRNAs and Their Inhibition in Modulating SLC5A8 Expression in the Context of Papillary Thyroid Carcinoma
Source: Int J Mol Sci. 2025 Aug 15;26(16):7889. doi: 10.3390/ijms26167889 (PMC12386254; doi:10.3390/ijms26167889)

Wojtek_2013-12-04 miRy dorobki

Programs

Program Name	pre-incubation		
Cycles	1	Analysis Mode	None

Target (°C)	Acquisition Mode	Hold (hh:mm:ss)	Ramp Rate (°C/s)	Acquisitions (per °C)	Sec Target (°C)	Step size (°C)	Step Delay (cycles)
95	None	00:10:00	4,80		0	0	0

Program Name	amplification		
Cycles	50	Analysis Mode	Quantification

Target (°C)	Acquisition Mode	Hold (hh:mm:ss)	Ramp Rate (°C/s)	Acquisitions (per °C)	Sec Target (°C)	Step size (°C)	Step Delay (cycles)
95	None	00:00:10	4,80		0	0	0
60	Single	00:00:30	2,50		0	0	0
72	None	00:00:01	4,80		0	0	0

Program Name	cooling		
Cycles	1	Analysis Mode	None

Target (°C)	Acquisition Mode	Hold (hh:mm:ss)	Ramp Rate (°C/s)	Acquisitions (per °C)	Sec Target (°C)	Step size (°C)	Step Delay (cycles)
40	None	00:00:30	2,50		0	0	0

Abs Quant/2nd Derivative Max for All (Abs Quant/2nd Derivative Max)

Statistics

Samples	Mean Cp	Std Cp	Mean conc	Std conc
A13, A14, A15	27,21	0,11		
A16, A17, A18	31,24	0,22		
A19, A20, A21	33,89	0,01		
A22, A23, A24	32,64	0,01		
B13, B14, B15	25,95	0,05		
B16, B17, B18	28,44	0,11		
B19, B20, B21	35,09	0,33		
B22, B23, B24	34,15	0,10		
C13, C14, C15	25,74	0,05		
C16, C17, C18	27,70	0,08		
C19, C20, C21	33,55	0,31		
C22, C23, C24	34,66	0,18		
D13, D14, D15	25,58	0,17		
D16, D17, D18	28,66	0,22		

Statistics

Samples	Mean Cp	Std Cp	Mean conc	Std conc
D19, D20, D21	33,88	0,15		
D22, D23, D24	33,82	0,13		
E13, E14, E15	26,60	0,06		
E16, E17, E18	28,47	0,15		
E19, E20, E21	33,99	0,05		
E22, E23, E24	34,50	0,37		
F13, F14, F15	26,13	0,16		
F16, F17, F18	28,45	0,10		
F19, F20, F21	33,99	0,18		
F22, F23, F24	33,05	0,12		
G13, G14, G15	26,62	0,20		
G16, G17, G18	27,31	0,04		
G19, G20, G21	34,47	0,30		
G22, G23, G24	32,20	0,12		
H13, H14, H15	28,17	0,36		
H16, H17, H18	30,24	0,09		
H19, H20, H21	35,11	0,24		
H22, H23, H24	33,71	0,02		
I14, I15	37,34			
I17, I18				
I20, I21				
I23, I24				

Amplification Curves

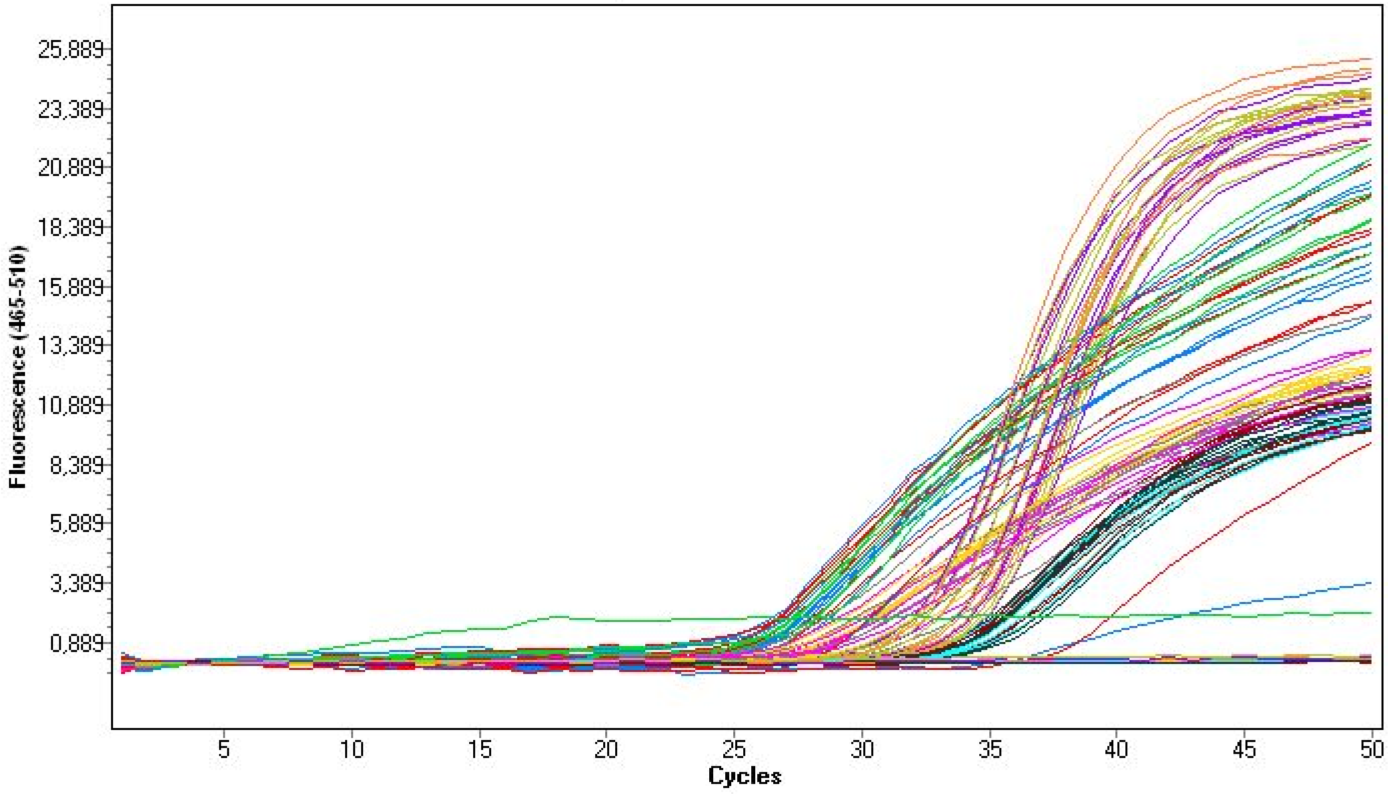

Supplement: Supplementary file 1 [file ijms-26-07889-s001.zip › ijms-3558049-supplementary/Manuscript data/Fig4 data/2013-12-04 miRy 538-1622.PDF]
