# Supplementary material for: MicroRNAs and Their Inhibition in Modulating SLC5A8 Expression in the Context of Papillary Thyroid Carcinoma
Source: Int J Mol Sci. 2025 Aug 15;26(16):7889. doi: 10.3390/ijms26167889 (PMC12386254; doi:10.3390/ijms26167889)
Supplement: Supplementary file 1 [file ijms-26-07889-s001.zip › ijms-3558049-supplementary/Manuscript data/Fig5A data/2019-07-10 HEK293 po V5-AIT.PDF]

Wojtek_2019-07-10 HEK293 po V5-AIT

Programs

Program Name	pre-incubation		
Cycles	1	Analysis Mode	None

Target (°C)	Acquisition Mode	Hold (hh:mm:ss)	Ramp Rate (°C/s)	Acquisitions (per °C)	Sec Target (°C)	Step size (°C)	Step Delay (cycles)
95	None	00:10:00	4.80		0	0	0

Program Name	amplification		
Cycles	55	Analysis Mode	Quantification

Target (°C)	Acquisition Mode	Hold (hh:mm:ss)	Ramp Rate (°C/s)	Acquisitions (per °C)	Sec Target (°C)	Step size (°C)	Step Delay (cycles)
95	None	00:00:10	4.80		0	0	0
58	None	00:00:15	2.50		0	0	0
72	Single	00:00:10	4.80		0	0	0

Program Name	melting curve		
Cycles	1	Analysis Mode	Melting Curves

Target (°C)	Acquisition Mode	Hold (hh:mm:ss)	Ramp Rate (°C/s)	Acquisitions (per °C)	Sec Target (°C)	Step size (°C)	Step Delay (cycles)
95	None	00:00:05	4.80		0	0	0
65	None	00:01:00	2.50		0	0	0
97	Continuous		0.11	5	0	0	0

Program Name	cooling		
Cycles	1	Analysis Mode	None

Target (°C)	Acquisition Mode	Hold (hh:mm:ss)	Ramp Rate (°C/s)	Acquisitions (per °C)	Sec Target (°C)	Step size (°C)	Step Delay (cycles)
40	None	00:00:30	2.50		0	0	0

Tm Calling for HPRT (Tm Calling)

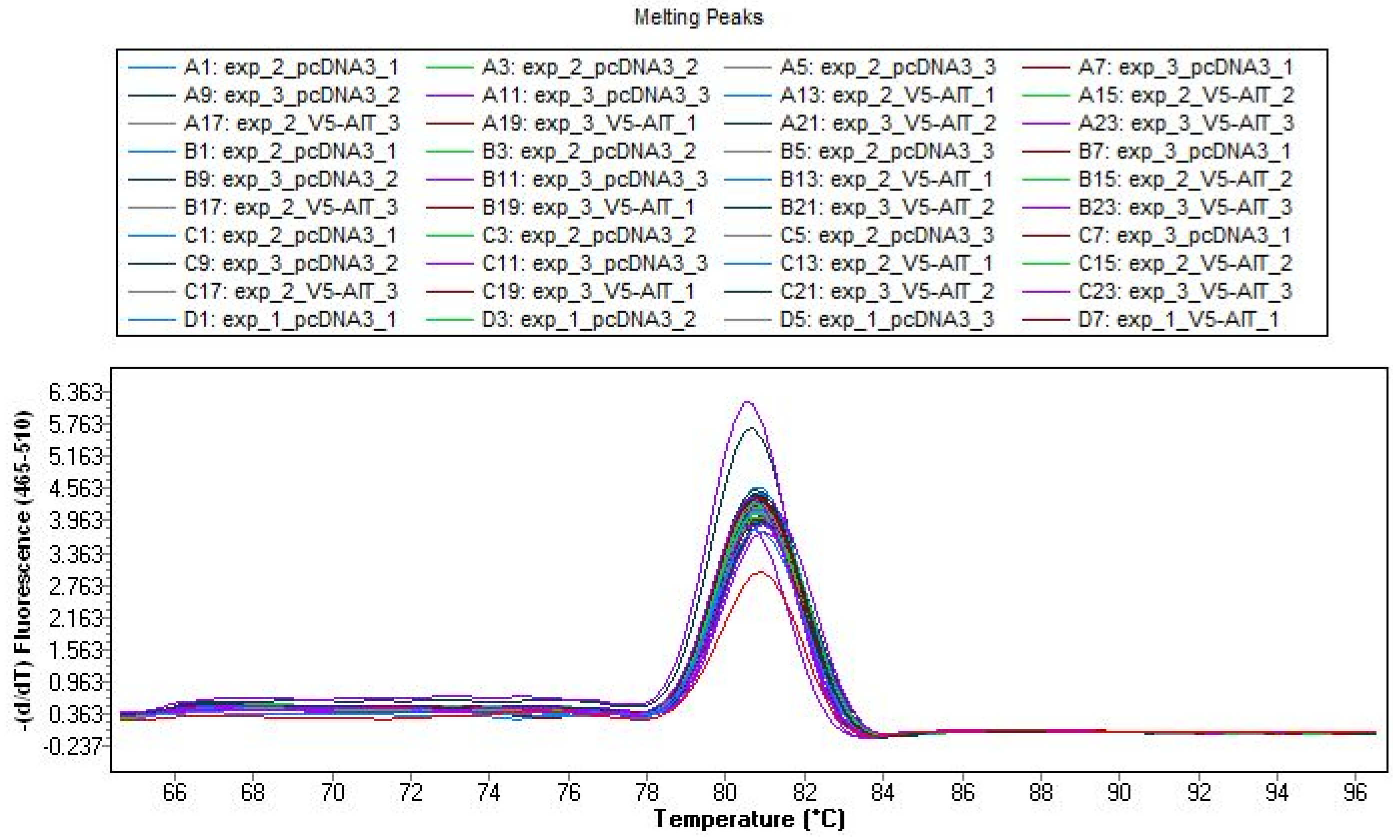

Tm Calling for BIRC5 (Tm Calling)

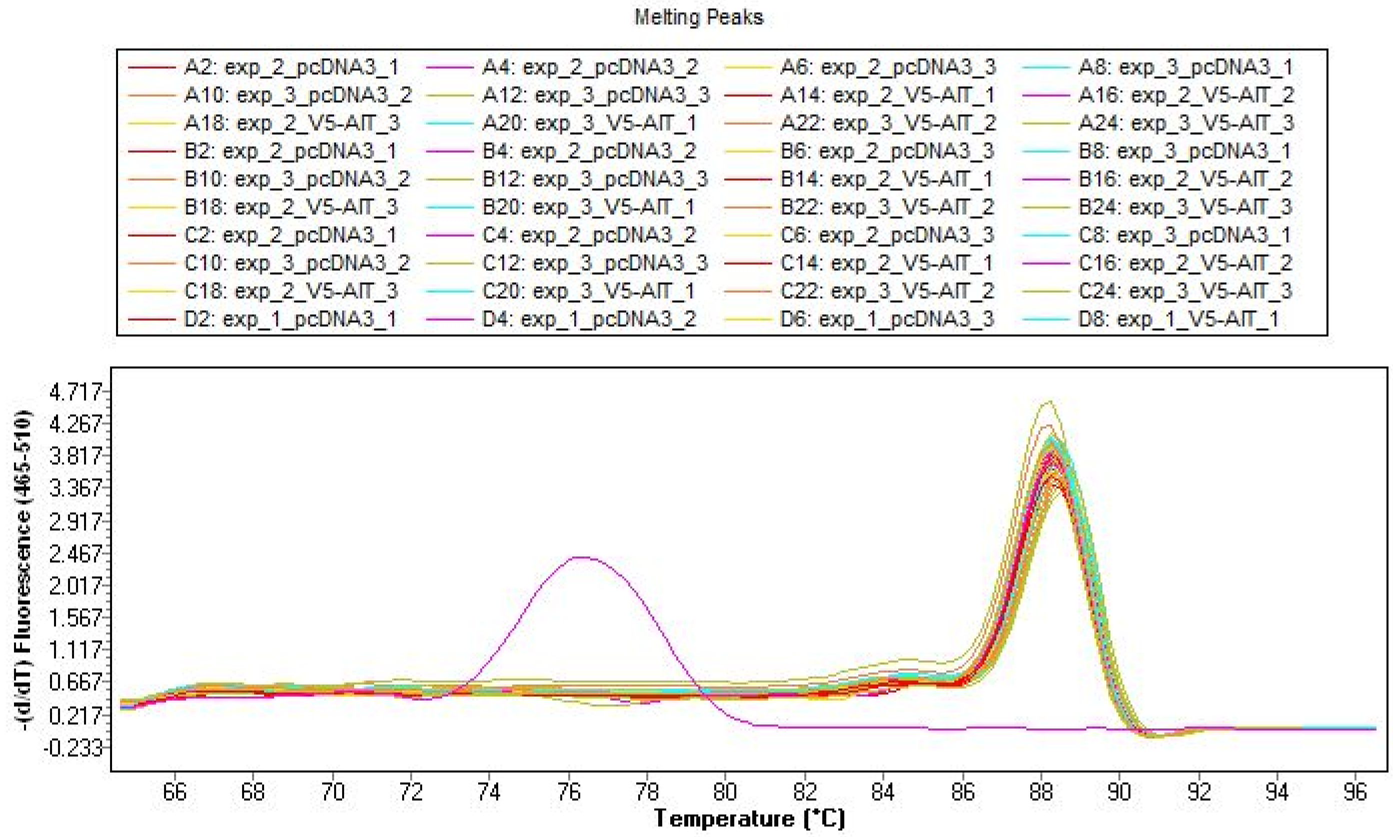

Tm Calling for AIT (Tm Calling)

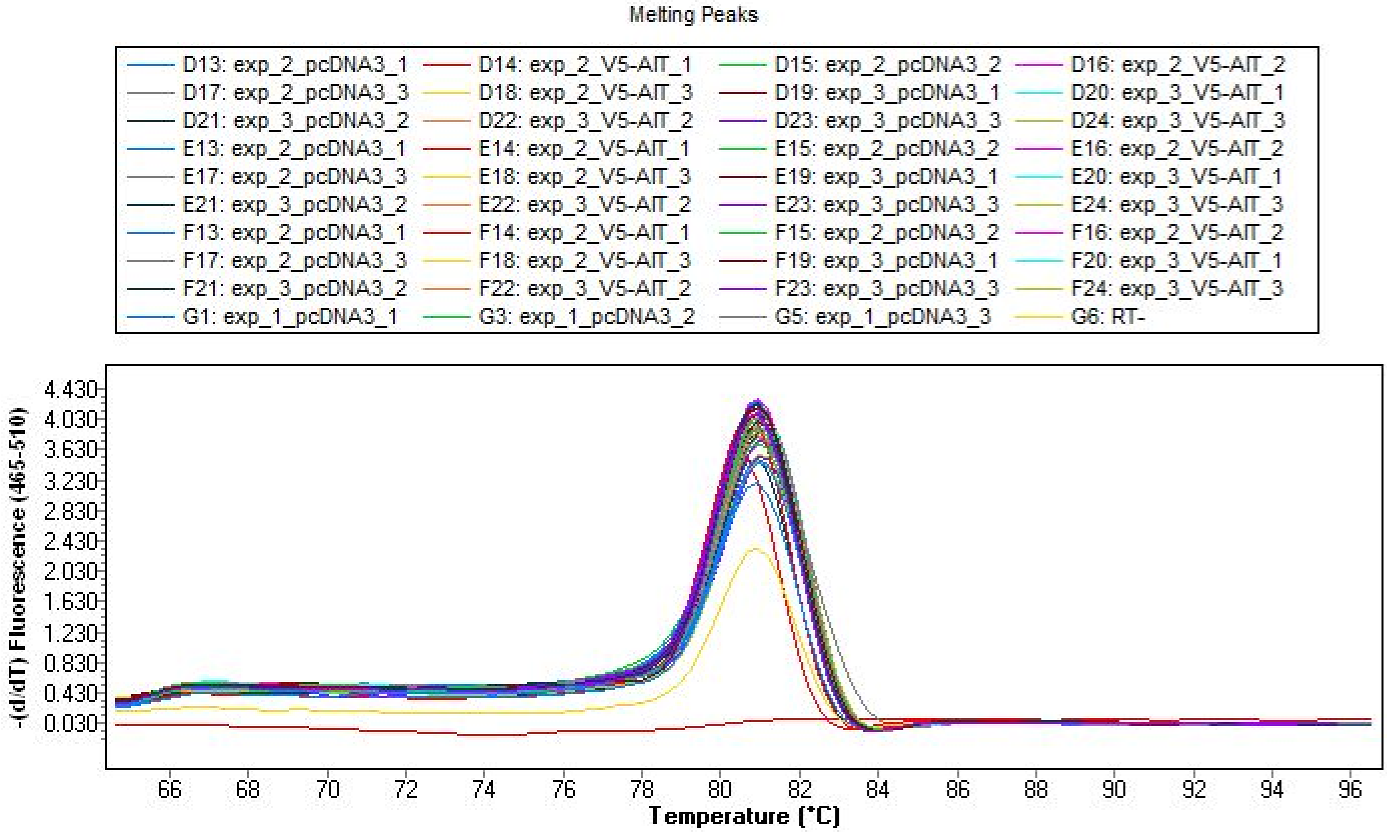

Abs Quant/2nd Derivative Max for HPRT (Abs Quant/2nd Derivative Max)

Results

Inc	Pos	Name	Type	CP	Concentration	Standard	Status
☑	A1	exp_2_pcDNA3_1	Unknown	25.09			
☑	A3	exp_2_pcDNA3_2	Unknown	24.72			
☑	A5	exp_2_pcDNA3_3	Unknown	25.69			
☑	A7	exp_3_pcDNA3_1	Unknown	25.07			
☑	A9	exp_3_pcDNA3_2	Unknown	24.63			
☑	A11	exp_3_pcDNA3_3	Unknown	24.82			
☑	A13	exp_2_V5-AIT_1	Unknown	24.98			
☑	A15	exp_2_V5-AIT_2	Unknown	25.22			
☑	A17	exp_2_V5-AIT_3	Unknown	24.91			
☑	A19	exp_3_V5-AIT_1	Unknown	24.46			
☑	A21	exp_3_V5-AIT_2	Unknown	25.62			
☑	A23	exp_3_V5-AIT_3	Unknown	24.66			
☑	B1	exp_2_pcDNA3_1	Unknown	25.19			
☑	B3	exp_2_pcDNA3_2	Unknown	24.74			
☑	B5	exp_2_pcDNA3_3	Unknown	25.87			

Results

Inc	Pos	Name	Type	CP	Concentration	Standard	Status
☑	B7	exp_3_pcDNA3_1	Unknown	25.11			
☑	B9	exp_3_pcDNA3_2	Unknown	24.69			
☑	B11	exp_3_pcDNA3_3	Unknown	24.93			
☑	B13	exp_2_V5-AIT_1	Unknown	25.12			
☑	B15	exp_2_V5-AIT_2	Unknown	25.29			
☑	B17	exp_2_V5-AIT_3	Unknown	24.87			
☑	B19	exp_3_V5-AIT_1	Unknown	24.48			
☑	B21	exp_3_V5-AIT_2	Unknown	25.67			
☑	B23	exp_3_V5-AIT_3	Unknown	24.68			
☑	C1	exp_2_pcDNA3_1	Unknown	25.06			
☑	C3	exp_2_pcDNA3_2	Unknown	24.84			
☑	C5	exp_2_pcDNA3_3	Unknown	25.87			
☑	C7	exp_3_pcDNA3_1	Unknown	25.01			
☑	C9	exp_3_pcDNA3_2	Unknown	24.63			
☑	C11	exp_3_pcDNA3_3	Unknown	24.76			
☑	C13	exp_2_V5-AIT_1	Unknown	25.09			
☑	C15	exp_2_V5-AIT_2	Unknown	25.18			
☑	C17	exp_2_V5-AIT_3	Unknown	24.81			
☑	C19	exp_3_V5-AIT_1	Unknown	24.56			
☑	C21	exp_3_V5-AIT_2	Unknown	25.61			
☑	C23	exp_3_V5-AIT_3	Unknown	24.70			
☑	D1	exp_1_pcDNA3_1	Unknown	24.83			
☑	D3	exp_1_pcDNA3_2	Unknown	25.57			
☑	D5	exp_1_pcDNA3_3	Unknown	25.05			
☑	D7	exp_1_V5-AIT_1	Unknown	24.93			
☑	D9	exp_1_V5-AIT_2	Unknown	25.07			
☑	D11	exp_1_V5-AIT_3	Unknown	23.75			
☑	E1	exp_1_pcDNA3_1	Unknown	24.84			
☑	E3	exp_1_pcDNA3_2	Unknown	25.50			
☑	E5	exp_1_pcDNA3_3	Unknown	25.01			
☑	E7	exp_1_V5-AIT_1	Unknown	24.94			
☑	E9	exp_1_V5-AIT_2	Unknown	24.98			
☑	E11	exp_1_V5-AIT_3	Unknown	23.89			
☑	F1	exp_1_pcDNA3_1	Unknown	24.88			
☑	F3	exp_1_pcDNA3_2	Unknown	25.56			
☑	F5	exp_1_pcDNA3_3	Unknown	25.08			
☑	F7	exp_1_V5-AIT_1	Unknown	24.84			

Results

Inc	Pos	Name	Type	CP	Concentration	Standard	Status
☑	F9	exp_1_V5-AIT_2	Unknown	25.00			
☑	F11	exp_1_V5-AIT_3	Unknown	23.86			
☑	G2	RT-	Negative Control	31.60			

Abs Quant/2nd Derivative Max for BIRC5 (Abs Quant/2nd Derivative Max)

Results

Inc	Pos	Name	Type	CP	Concentration	Standard	Status
☑	A2	exp_2_pcDNA3_1	Unknown	24.77			
☑	A4	exp_2_pcDNA3_2	Unknown	24.66			
☑	A6	exp_2_pcDNA3_3	Unknown	26.22			
☑	A8	exp_3_pcDNA3_1	Unknown	24.79			
☑	A10	exp_3_pcDNA3_2	Unknown	24.51			
☑	A12	exp_3_pcDNA3_3	Unknown	24.79			
☑	A14	exp_2_V5-AIT_1	Unknown	25.14			
☑	A16	exp_2_V5-AIT_2	Unknown	25.20			
☑	A18	exp_2_V5-AIT_3	Unknown	25.00			
☑	A20	exp_3_V5-AIT_1	Unknown	24.71			
☑	A22	exp_3_V5-AIT_2	Unknown	25.82			
☑	A24	exp_3_V5-AIT_3	Unknown	24.62			
☑	B2	exp_2_pcDNA3_1	Unknown	24.73			
☑	B4	exp_2_pcDNA3_2	Unknown	24.65			
☑	B6	exp_2_pcDNA3_3	Unknown	26.06			
☑	B8	exp_3_pcDNA3_1	Unknown	24.81			
☑	B10	exp_3_pcDNA3_2	Unknown	24.50			
☑	B12	exp_3_pcDNA3_3	Unknown	24.77			
☑	B14	exp_2_V5-AIT_1	Unknown	25.09			
☑	B16	exp_2_V5-AIT_2	Unknown	25.14			
☑	B18	exp_2_V5-AIT_3	Unknown	24.99			
☑	B20	exp_3_V5-AIT_1	Unknown	24.73			
☑	B22	exp_3_V5-AIT_2	Unknown	25.96			
☑	B24	exp_3_V5-AIT_3	Unknown	24.65			
☑	C2	exp_2_pcDNA3_1	Unknown	24.79			
☑	C4	exp_2_pcDNA3_2	Unknown	24.54			
☑	C6	exp_2_pcDNA3_3	Unknown	25.82			
☑	C8	exp_3_pcDNA3_1	Unknown	24.69			
☑	C10	exp_3_pcDNA3_2	Unknown	24.50			

Results

Inc	Pos	Name	Type	CP	Concentration	Standard	Status
☑	C12	exp_3_pcDNA3_3	Unknown	24.77			
☑	C14	exp_2_V5-AIT_1	Unknown	25.08			
☑	C16	exp_2_V5-AIT_2	Unknown	25.20			
☑	C18	exp_2_V5-AIT_3	Unknown	24.93			
☑	C20	exp_3_V5-AIT_1	Unknown	24.65			
☑	C22	exp_3_V5-AIT_2	Unknown	25.96			
☑	C24	exp_3_V5-AIT_3	Unknown	24.78			
☑	D2	exp_1_pcDNA3_1	Unknown	24.45			
☑	D4	exp_1_pcDNA3_2	Unknown	25.28			
☑	D6	exp_1_pcDNA3_3	Unknown	24.82			
☑	D8	exp_1_V5-AIT_1	Unknown	24.66			
☑	D10	exp_1_V5-AIT_2	Unknown	24.73			
☑	D12	exp_1_V5-AIT_3	Unknown	23.96			
☑	E2	exp_1_pcDNA3_1	Unknown	24.57			
☑	E4	exp_1_pcDNA3_2	Unknown	25.24			
☑	E6	exp_1_pcDNA3_3	Unknown	24.88			
☑	E8	exp_1_V5-AIT_1	Unknown	24.69			
☑	E10	exp_1_V5-AIT_2	Unknown	24.62			
☑	E12	exp_1_V5-AIT_3	Unknown	23.93			
☑	F2	exp_1_pcDNA3_1	Unknown	24.58			
☑	F4	exp_1_pcDNA3_2	Unknown	25.16			
☑	F6	exp_1_pcDNA3_3	Unknown	24.88			
☑	F8	exp_1_V5-AIT_1	Unknown	24.69			
☑	F10	exp_1_V5-AIT_2	Unknown	24.62			
☑	F12	exp_1_V5-AIT_3	Unknown	23.98			
☑	G4	RT-	Negative Control	29.04			

Abs Quant/2nd Derivative Max for AIT (Abs Quant/2nd Derivative Max)

Results

Inc	Pos	Name	Type	CP	Concentration	Standard	Status
☑	D13	exp_2_pcDNA3_1	Unknown	36.03			
☑	D14	exp_2_V5-AIT_1	Unknown				
☑	D15	exp_2_pcDNA3_2	Unknown	38.33			
☑	D16	exp_2_V5-AIT_2	Unknown	19.56			
☑	D17	exp_2_pcDNA3_3	Unknown	37.15			
☑	D18	exp_2_V5-AIT_3	Unknown	19.24			

Results

Inc	Pos	Name	Type	CP	Concentration	Standard	Status
☑	D19	exp_3_pcDNA3_1	Unknown	35.75			
☑	D20	exp_3_V5-AIT_1	Unknown	19.33			
☑	D21	exp_3_pcDNA3_2	Unknown	35.14			
☑	D22	exp_3_V5-AIT_2	Unknown	19.77			
☑	D23	exp_3_pcDNA3_3	Unknown	36.01			
☑	D24	exp_3_V5-AIT_3	Unknown	19.46			
☑	E13	exp_2_pcDNA3_1	Unknown	37.14			
☑	E14	exp_2_V5-AIT_1	Unknown	18.21			
☑	E15	exp_2_pcDNA3_2	Unknown	37.18			
☑	E16	exp_2_V5-AIT_2	Unknown	19.46			
☑	E17	exp_2_pcDNA3_3	Unknown	37.35			
☑	E18	exp_2_V5-AIT_3	Unknown	19.25			
☑	E19	exp_3_pcDNA3_1	Unknown	34.98			
☑	E20	exp_3_V5-AIT_1	Unknown	19.31			
☑	E21	exp_3_pcDNA3_2	Unknown	35.50			
☑	E22	exp_3_V5-AIT_2	Unknown	19.77			
☑	E23	exp_3_pcDNA3_3	Unknown	36.03			
☑	E24	exp_3_V5-AIT_3	Unknown	19.43			
☑	F13	exp_2_pcDNA3_1	Unknown	35.12			
☑	F14	exp_2_V5-AIT_1	Unknown	19.10			
☑	F15	exp_2_pcDNA3_2	Unknown	37.15			
☑	F16	exp_2_V5-AIT_2	Unknown	19.48			
☑	F17	exp_2_pcDNA3_3	Unknown	36.52			
☑	F18	exp_2_V5-AIT_3	Unknown	19.14			
☑	F19	exp_3_pcDNA3_1	Unknown	37.74			
☑	F20	exp_3_V5-AIT_1	Unknown	19.20			
☑	F21	exp_3_pcDNA3_2	Unknown	35.53			
☑	F22	exp_3_V5-AIT_2	Unknown	19.75			
☑	F23	exp_3_pcDNA3_3	Unknown	37.48			
☑	F24	exp_3_V5-AIT_3	Unknown	19.35			
☑	G1	exp_1_pcDNA3_1	Unknown	35.74			
☑	G3	exp_1_pcDNA3_2	Unknown	26.97			
☑	G5	exp_1_pcDNA3_3	Unknown	32.82			
☑	G6	RT-	Negative Control	34.48			
☑	G7	exp_1_V5-AIT_1	Unknown	19.92			
☑	G9	exp_1_V5-AIT_2	Unknown	19.81			
☑	G11	exp_1_V5-AIT_3	Unknown	19.92			

Results

Inc	Pos	Name	Type	CP	Concentration	Standard	Status
☑	H1	exp_1_pcDNA3_1	Unknown	35.78			
☑	H3	exp_1_pcDNA3_2	Unknown	26.73			
☑	H5	exp_1_pcDNA3_3	Unknown	32.94			
☑	H7	exp_1_V5-AIT_1	Unknown	19.85			
☑	H9	exp_1_V5-AIT_2	Unknown	19.74			
☑	H11	exp_1_V5-AIT_3	Unknown	19.86			
☑	I1	exp_1_pcDNA3_1	Unknown	35.46			
☑	I3	exp_1_pcDNA3_2	Unknown	26.52			
☑	I5	exp_1_pcDNA3_3	Unknown	32.13			
☑	I7	exp_1_V5-AIT_1	Unknown	19.78			
☑	I9	exp_1_V5-AIT_2	Unknown	19.65			
☑	I11	exp_1_V5-AIT_3	Unknown	19.77			
