# Supplementary material for: MicroRNAs and Their Inhibition in Modulating SLC5A8 Expression in the Context of Papillary Thyroid Carcinoma
Source: Int J Mol Sci. 2025 Aug 15;26(16):7889. doi: 10.3390/ijms26167889 (PMC12386254; doi:10.3390/ijms26167889)
Supplement: Supplementary file 1 [file ijms-26-07889-s001.zip › ijms-3558049-supplementary/Manuscript data/Fig5B data/Exp_1/2014-10-07 miRy po transfekcji plazmidem.PDF]

Roche

Wojtek_2014-10-07 miRy po transfekcji plazmidem 400ng

Programs

Program Name	pre-incubation		
Cycles	1	Analysis Mode	None

Target (°C)	Acquisition Mode	Hold (hh:mm:ss)	Ramp Rate (°C/s)	Acquisitions (per °C)	Sec Target (°C)	Step size (°C)	Step Delay (cycles)
95	None	00:10:00	4.80		0	0	0

Program Name	amplification		
Cycles	60	Analysis Mode	Quantification

Target (°C)	Acquisition Mode	Hold (hh:mm:ss)	Ramp Rate (°C/s)	Acquisitions (per °C)	Sec Target (°C)	Step size (°C)	Step Delay (cycles)
95	None	00:00:10	4.80		0	0	0
60	Single	00:00:30	2.50		0	0	0
72	None	00:00:01	4.80		0	0	0

Program Name	cooling		
Cycles	1	Analysis Mode	None

Target (°C)	Acquisition Mode	Hold (hh:mm:ss)	Ramp Rate (°C/s)	Acquisitions (per °C)	Sec Target (°C)	Step size (°C)	Step Delay (cycles)
40	None	00:00:30	2.50		0	0	0

Abs Quant/2nd Derivative Max for All (Abs Quant/2nd Derivative Max)

Statistics

Samples	Mean Cp	Std Cp	Mean conc	Std conc
H21, H22, H23	36.19	0.56		
I21, I22, I23	36.08	0.20		
J22, K22, L22	31.45	0.13		
J23, K23, L23	31.74	0.23		
J24, K24, L24	32.13	0.03		
K21, L21				
M1, N1, O1	27.54	0.20		
M2, N2, O2	33.37	0.11		
M3, N3, O3	27.60	0.11		
M4, N4, O4	33.50	0.22		
M5, N5, O5	28.16	0.11		
M6, N6, O6	33.95	0.65		
M7, N7, O7	27.65	0.20		

Statistics

Samples	Mean Cp	Std Cp	Mean conc	Std conc
M8, N8, O8	28.32	0.16		
M9, N9, O9	27.31	0.28		
M10, N10, O10	28.18	0.13		
M11, N11, O11	27.88	0.27		
M12, N12, O12	28.91	0.21		
M13, N13, O13	28.51	0.18		
M14, N14, O14	26.51	0.10		
M15, N15, O15	28.38	0.32		
M16, N16, O16	26.32	0.12		
M17, N17, O17	28.07	0.41		
M18, N18, O18	26.54	0.08		
M19, N19, O19	28.62	0.32		
M20, N20, O20	31.50	0.19		
M21, N21, O21	28.33	0.23		
M22, N22, O22	31.15	0.09		
M23, N23, O23	28.77	0.20		
M24, N24, O24	31.22	0.04		
P2, P3				
P5, P6				
P7, P8, P9				
P10, P11, P12	42.72	10.65		

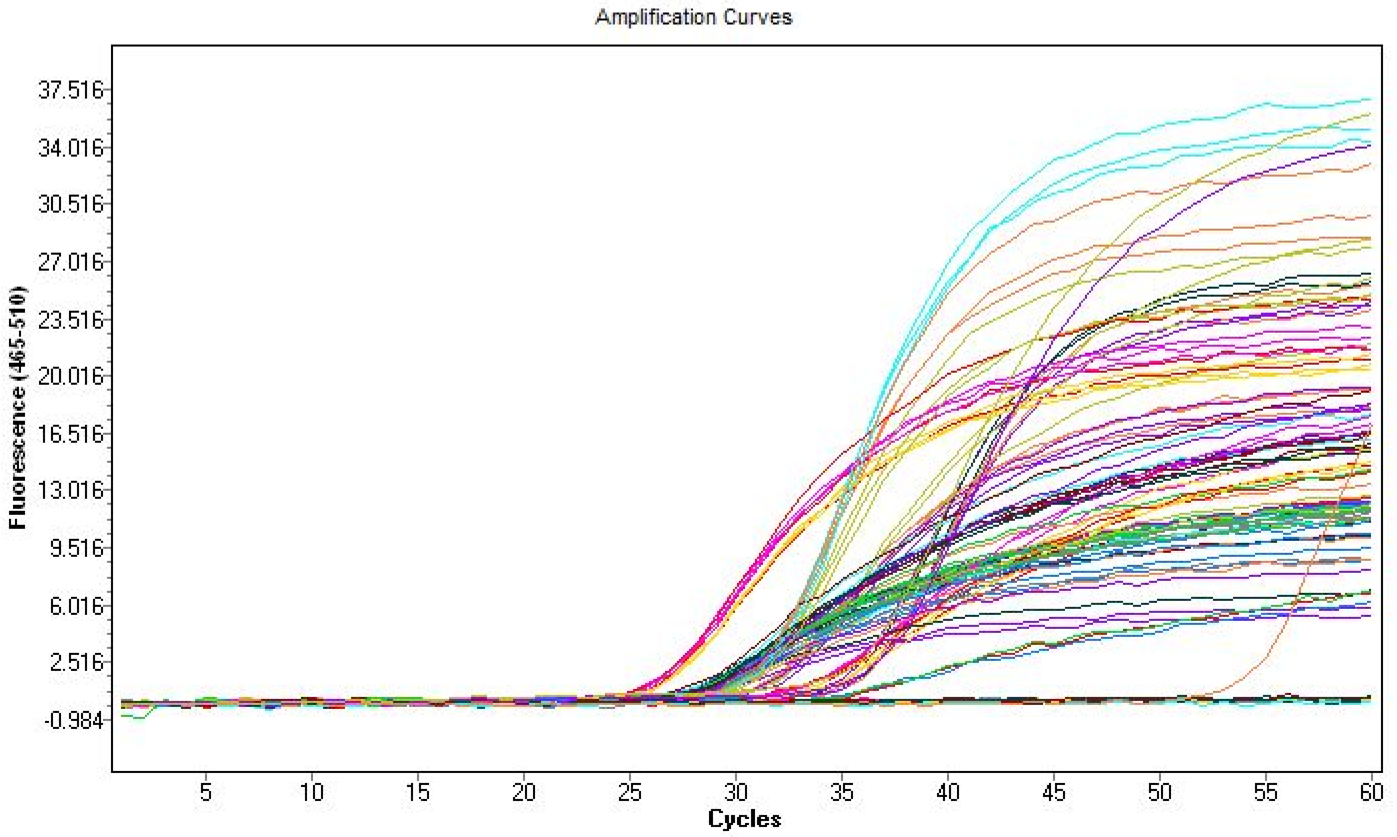
Amplification Curves
Fluorescence (465-510)
37.516
34.016
30.516
27.016
23.516
20.016
16.516
13.016
9.516
6.016
2.516
-0.984
5
10
15
20
25
30
35
40
45
50
55
60
Cycles
