# Supplementary material for: MicroRNAs and Their Inhibition in Modulating SLC5A8 Expression in the Context of Papillary Thyroid Carcinoma
Source: Int J Mol Sci. 2025 Aug 15;26(16):7889. doi: 10.3390/ijms26167889 (PMC12386254; doi:10.3390/ijms26167889)

Roche

Wojtek_2014-10-14 miRy po transfekcji plazmidem 400ng

Programs

Program Name	pre-incubation		
Cycles	1	Analysis Mode	None

Target (°C)	Acquisition Mode	Hold (hh:mm:ss)	Ramp Rate (°C/s)	Acquisitions (per °C)	Sec Target (°C)	Step size (°C)	Step Delay (cycles)
95	None	00:10:00	4.80		0	0	0

Program Name	amplification		
Cycles	50	Analysis Mode	Quantification

Target (°C)	Acquisition Mode	Hold (hh:mm:ss)	Ramp Rate (°C/s)	Acquisitions (per °C)	Sec Target (°C)	Step size (°C)	Step Delay (cycles)
95	None	00:00:10	4.80		0	0	0
60	Single	00:00:30	2.50		0	0	0
72	None	00:00:01	4.80		0	0	0

Program Name	cooling		
Cycles	1	Analysis Mode	None

Target (°C)	Acquisition Mode	Hold (hh:mm:ss)	Ramp Rate (°C/s)	Acquisitions (per °C)	Sec Target (°C)	Step size (°C)	Step Delay (cycles)
40	None	00:00:30	2.50		0	0	0

Abs Quant/2nd Derivative Max for All (Abs Quant/2nd Derivative Max)

Statistics

Samples	Mean Cp	Std Cp	Mean conc	Std conc
A1, B1, C1	28.05	0.16		
A2, B2, C2	35.35	0.34		
A3, B3, C3	27.68	0.10		
A4, B4, C4	35.29	0.31		
A5, B5, C5	27.39	0.11		
A6, B6, C6	35.16	0.27		
A7, B7, C7	27.45	0.18		
A8, B8, C8	30.11	0.13		
A9, B9, C9	27.64	0.14		
A10, B10, C10	30.13	0.14		
A11, B11, C11	27.38	0.10		
A12, B12, C12	29.86	0.05		
A13, B13, C13	27.41	0.34		

Statistics

Samples	Mean Cp	Std Cp	Mean conc	Std conc
A14, B14, C14	25.55	0.02		
A15, B15, C15	27.57	0.03		
A16, B16, C16	25.69	0.01		
A17, B17, C17	27.53	0.04		
A18, B18, C18	25.52	0.04		
A19, B19, C19	27.75	0.03		
A20, B20, C20	30.83	0.10		
A21, B21, C21	28.00	0.09		
A22, B22, C22	31.22	0.26		
A23, B23, C23	27.89	0.10		
A24, B24, C24	30.91	0.17		
D1, E1, F1	31.29	0.05		
D2, E2, F2	39.39	0.62		
D3, E3, F3	31.24	0.42		
D4, E4, F4	39.95	0.53		
D5, E5, F5	30.90	0.04		
D6, E6, F6	39.48	1.78		
E7, F7	36.68	0.68		
E8, F8				
E9, F9				
E10, F10				

Amplification Curves

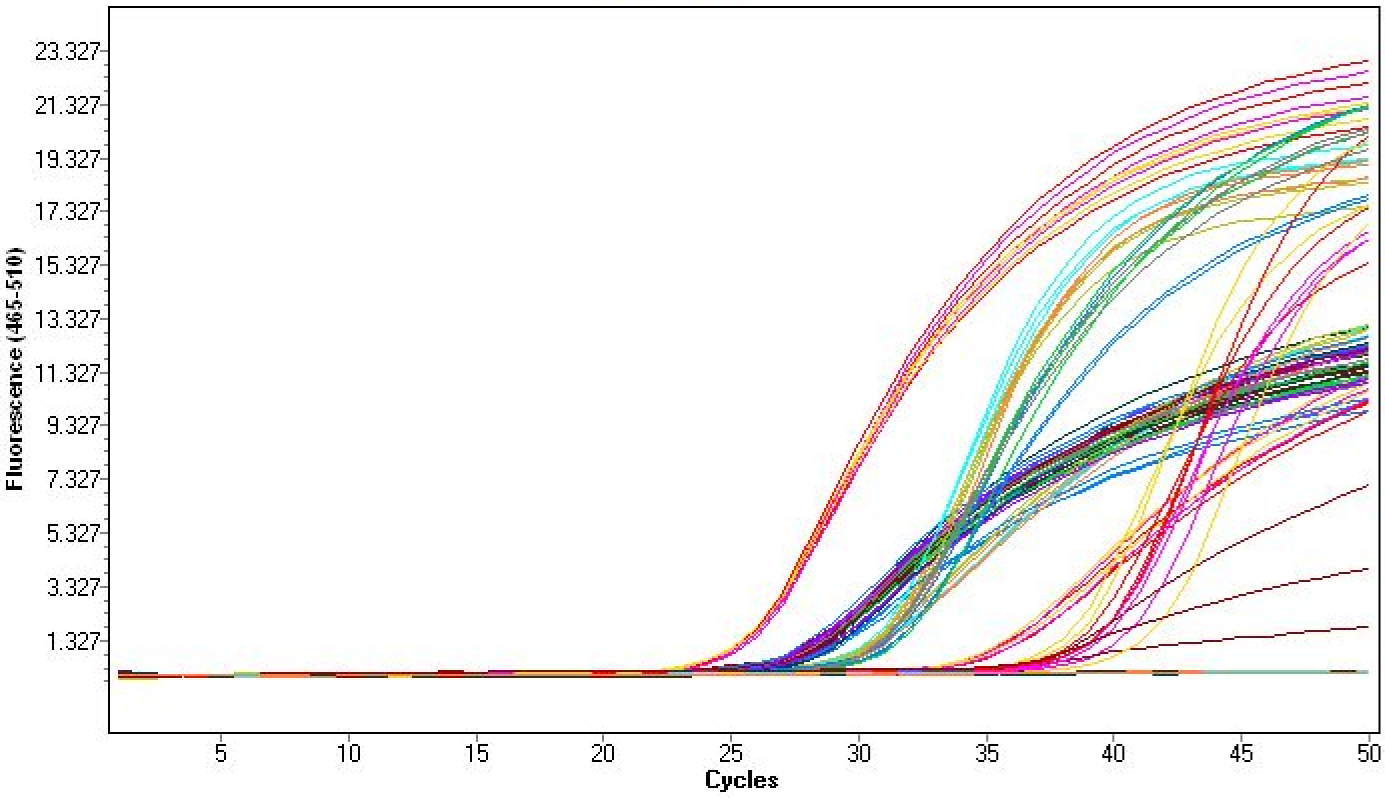

Supplement: Supplementary file 1 [file ijms-26-07889-s001.zip › ijms-3558049-supplementary/Manuscript data/Fig5B data/Exp_2/2014-10-14 miRy po transfekcji plazmidem.PDF]
