# Supplementary material for: MicroRNAs and Their Inhibition in Modulating SLC5A8 Expression in the Context of Papillary Thyroid Carcinoma
Source: Int J Mol Sci. 2025 Aug 15;26(16):7889. doi: 10.3390/ijms26167889 (PMC12386254; doi:10.3390/ijms26167889)
Supplement: Supplementary file 1 [file ijms-26-07889-s001.zip › ijms-3558049-supplementary/Manuscript data/Fig5B data/Exp_3/2014-10-22 miRy po transfekcji plazmidem 400ng.PDF]

Roche

Wojtek_2014-10-22 miRy po transfekcji plazmidem 400ng

Programs

Program Name	pre-incubation		
Cycles	1	Analysis Mode	None

Target (°C)	Acquisition Mode	Hold (hh:mm:ss)	Ramp Rate (°C/s)	Acquisitions (per °C)	Sec Target (°C)	Step size (°C)	Step Delay (cycles)
95	None	00:10:00	4.80		0	0	0

Program Name	amplification		
Cycles	50	Analysis Mode	Quantification

Target (°C)	Acquisition Mode	Hold (hh:mm:ss)	Ramp Rate (°C/s)	Acquisitions (per °C)	Sec Target (°C)	Step size (°C)	Step Delay (cycles)
95	None	00:00:10	4.80		0	0	0
60	Single	00:00:30	2.50		0	0	0
72	None	00:00:01	4.80		0	0	0

Program Name	cooling		
Cycles	1	Analysis Mode	None

Target (°C)	Acquisition Mode	Hold (hh:mm:ss)	Ramp Rate (°C/s)	Acquisitions (per °C)	Sec Target (°C)	Step size (°C)	Step Delay (cycles)
40	None	00:00:30	2.50		0	0	0

Abs Quant/2nd Derivative Max for All (Abs Quant/2nd Derivative Max)

Statistics

Samples	Mean Cp	Std Cp	Mean conc	Std conc
D11, E11, F11	32.33	0.65		
D12, E12, F12	37.91	0.31		
D13, E13, F13	31.76	0.11		
D14, E14, F14	37.19	0.17		
D15, E15, F15	32.11	0.08		
D16, E16, F16	38.80	1.01		
E17, F17	36.45	0.40		
E18, F18				
E19, F19				
E20, F20				
G1, H1, I1	27.03	0.12		
G2, H2, I2	33.06	0.02		
G3, H3, I3	26.21	0.15		

Statistics

Samples	Mean Cp	Std Cp	Mean conc	Std conc
G4, H4, I4	32.69	0.11		
G5, H5, I5	27.13	0.14		
G6, H6, I6	33.25	0.11		
G7, H7, I7	26.29	0.20		
G8, H8, I8	27.61	0.25		
G9, H9, I9	26.61	0.08		
G10, H10, I10	27.68	0.11		
G11, H11, I11	27.20	0.43		
G12, H12, I12	28.23	0.22		
G13, H13, I13	26.99	0.12		
G14, H14, I14	26.76	0.10		
G15, H15, I15	26.05	0.07		
G16, H16, I16	25.84	0.07		
G17, H17, I17	27.55	0.22		
G18, H18, I18	26.98	0.14		
G19, H19, I19	27.25	1.30		
G20, H20, I20	30.13	0.18		
G21, H21, I21	27.66	0.86		
G22, H22, I22	30.68	0.29		
G23, H23, I23	27.38	0.24		
G24, H24, I24	31.28	0.19		

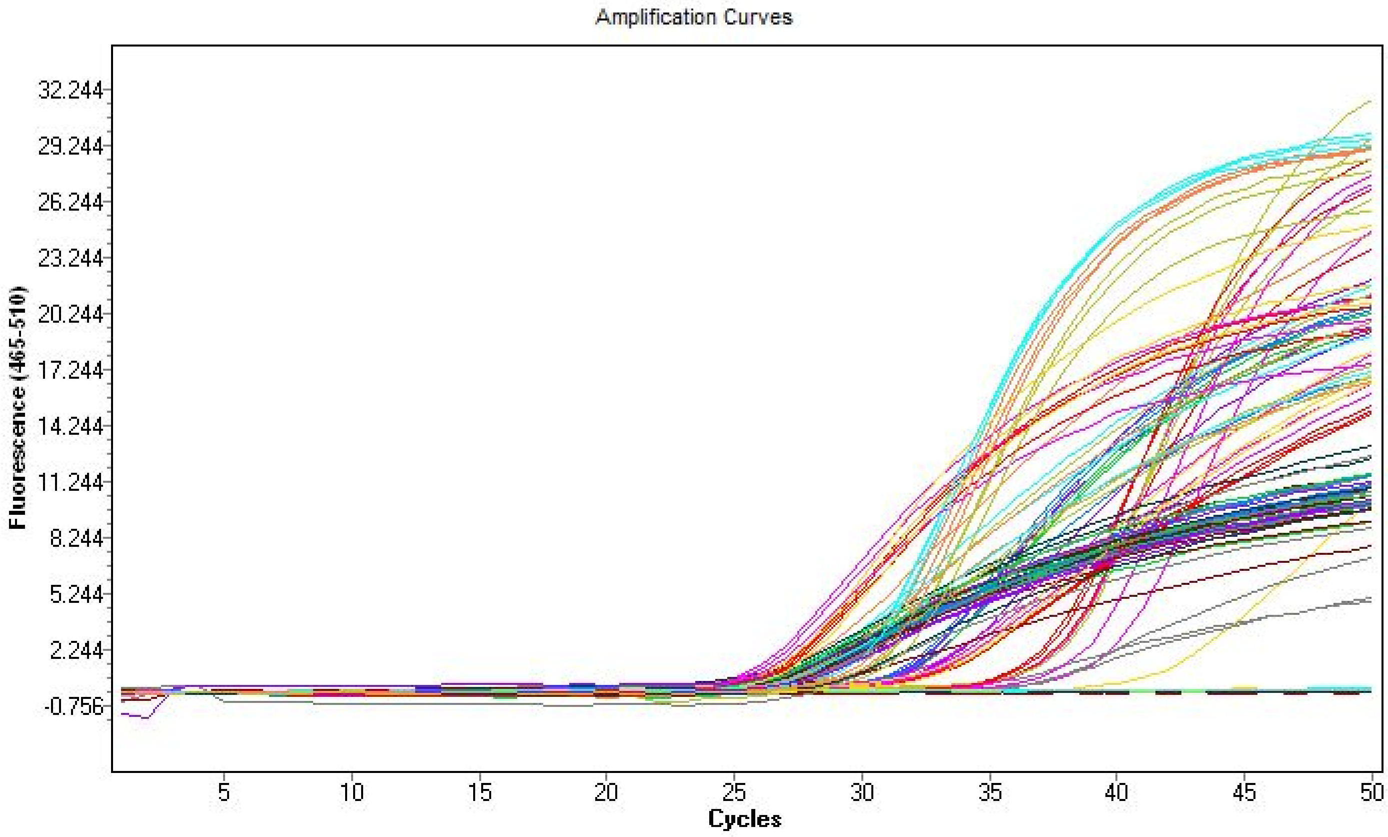
Amplification Curves
Fluorescence (465-510)
32.244
29.244
26.244
23.244
20.244
17.244
14.244
11.244
8.244
5.244
2.244
-0.756
5
10
15
20
25
30
35
40
45
50
Cycles
