# Supplementary figures and images for: MicroRNAs and Their Inhibition in Modulating SLC5A8 Expression in the Context of Papillary Thyroid Carcinoma
Source: Int J Mol Sci. 2025 Aug 15;26(16):7889. doi: 10.3390/ijms26167889 (PMC12386254; doi:10.3390/ijms26167889)

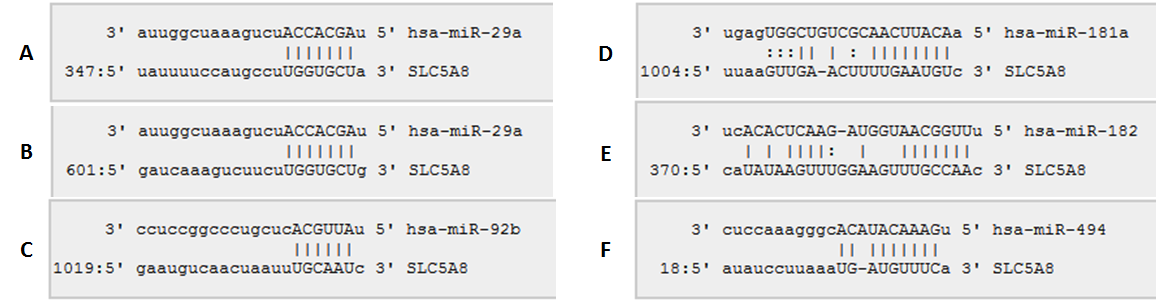

Supplement: Supplementary file 1 [file ijms-26-07889-s001.zip › ijms-3558049-supplementary/Manuscript data/Fig2.tif]
