# Supplementary material for: MicroRNAs and Their Inhibition in Modulating SLC5A8 Expression in the Context of Papillary Thyroid Carcinoma
Source: Int J Mol Sci. 2025 Aug 15;26(16):7889. doi: 10.3390/ijms26167889 (PMC12386254; doi:10.3390/ijms26167889)

Wojtek_2014-10-22_HPRT AIT po transfekcji plazmidem + BIRC5 PEN

Programs

Program Name	pre-incubation		
Cycles	1	Analysis Mode	None

Target (°C)	Acquisition Mode	Hold (hh:mm:ss)	Ramp Rate (°C/s)	Acquisitions (per °C)	Sec Target (°C)	Step size (°C)	Step Delay (cycles)
95	None	00:10:00	4.80		0	0	0

Program Name	amplification		
Cycles	50	Analysis Mode	Quantification

Target (°C)	Acquisition Mode	Hold (hh:mm:ss)	Ramp Rate (°C/s)	Acquisitions (per °C)	Sec Target (°C)	Step size (°C)	Step Delay (cycles)
95	None	00:00:10	4.80		0	0	0
58	None	00:00:05	2.50		0	0	0
72	Single	00:00:08	4.80		0	0	0

Program Name	melting curve		
Cycles	1	Analysis Mode	Melting Curves

Target (°C)	Acquisition Mode	Hold (hh:mm:ss)	Ramp Rate (°C/s)	Acquisitions (per °C)	Sec Target (°C)	Step size (°C)	Step Delay (cycles)
95	None	00:00:05	4.80		0	0	0
65	None	00:01:00	2.50		0	0	0
97	Continuous		0.11	5	0	0	0

Program Name	cooling		
Cycles	1	Analysis Mode	None

Target (°C)	Acquisition Mode	Hold (hh:mm:ss)	Ramp Rate (°C/s)	Acquisitions (per °C)	Sec Target (°C)	Step size (°C)	Step Delay (cycles)
40	None	00:00:30	2.50		0	0	0

Tm Calling for All (Tm Calling)

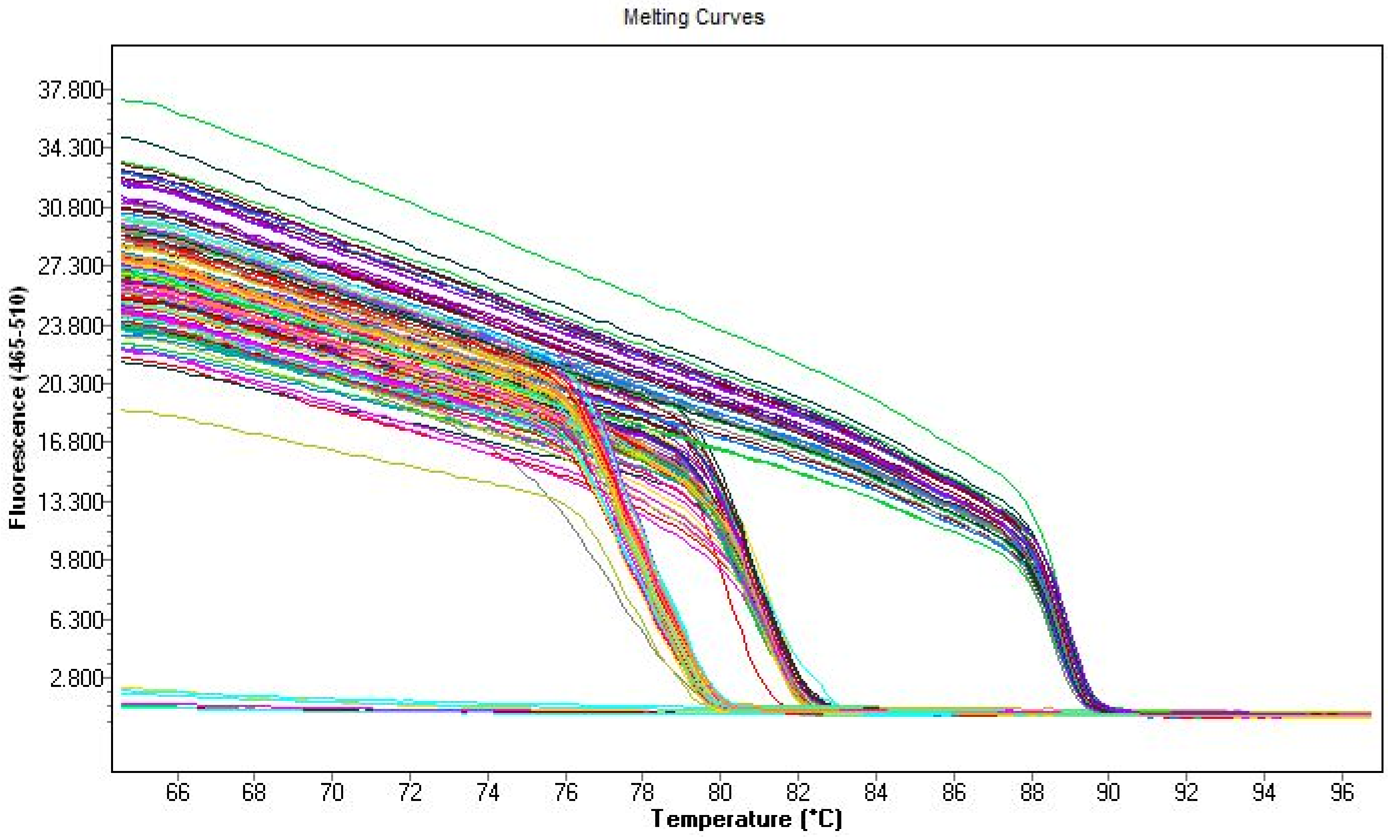

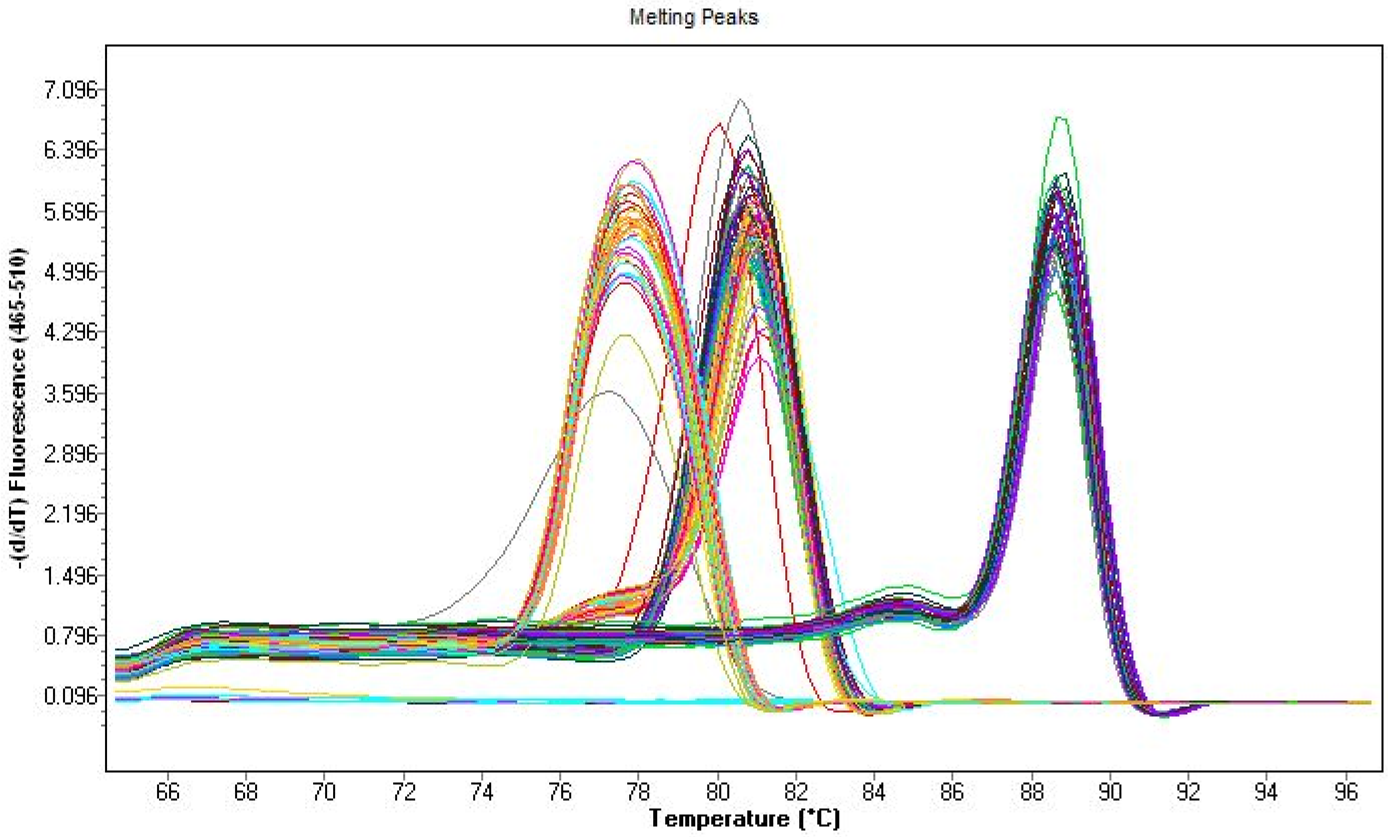

Abs Quant/2nd Derivative Max for All (Abs Quant/2nd Derivative Max)

Statistics

Samples	Mean Cp	Std Cp	Mean conc	Std conc
H1, I1, J1	21.29	0.17		
H2, I2, J2	40.31	0.76		
H3, I3, J3	21.49	0.10		
H4, I4, J4	42.58	2.12		
H5, I5, J5	21.26	0.09		
H6, I6, J6	41.11	1.18		
H7, I7, J7	21.91	0.22		
H8, I8, J8	42.04	0.28		
H9, I9, J9	21.68	0.08		
H10, I10, J10	42.04	1.43		
H11, I11, J11	22.14	0.03		
H12, I12, J12	42.23	0.85		
H13, I13, J13	22.67	0.04		
H14, I14, J14	42.82	1.36		
H15, I15, J15	22.69	0.09		
H16, I16, J16	43.95	1.24		
H17, I17, J17	22.31	0.22		
H18, I18, J18	42.20	0.76		
H19, I19, J19	22.49	0.18		
H20, I20, J20	42.39	0.86		
H21, I21, J21	21.69	0.17		
H22, I22, J22	41.21	0.81		
H23, I23, J23	21.76	0.15		
H24, I24, J24	42.18	2.61		
K1, L1, M1	22.89	0.16		
K2, L2, M2	33.05	0.25		
K3, L3, M3	23.12	0.11		
K4, L4, M4	33.51	0.23		
K5, L5, M5	23.20	0.19		
K6, L6, M6	33.62	0.14		
K7, L7, M7	24.37	0.13		
K8, L8, M8	34.65	0.13		
K9, L9, M9	24.05	0.06		
K10, L10, M10	34.03	0.03		
K11, L11, M11	24.53	0.20		
K12, L12, M12	34.55	0.09		
K13, L13, M13	24.82	0.12		

Statistics

Samples	Mean Cp	Std Cp	Mean conc	Std conc
K14, L14, M14	35.42	1.04		
K15, L15, M15	24.65	0.16		
K16, L16, M16	34.95	0.54		
K17, L17, M17	24.23	0.25		
K18, L18, M18	34.68	0.20		
K19, L19, M19	24.33	0.27		
K20, L20, M20	34.07	0.21		
K21, L21, M21	23.69	0.19		
K22, L22, M22	33.34	0.14		
K23, L23, M23	23.62	0.14		
K24, L24, M24	34.03	0.40		

Amplification Curves

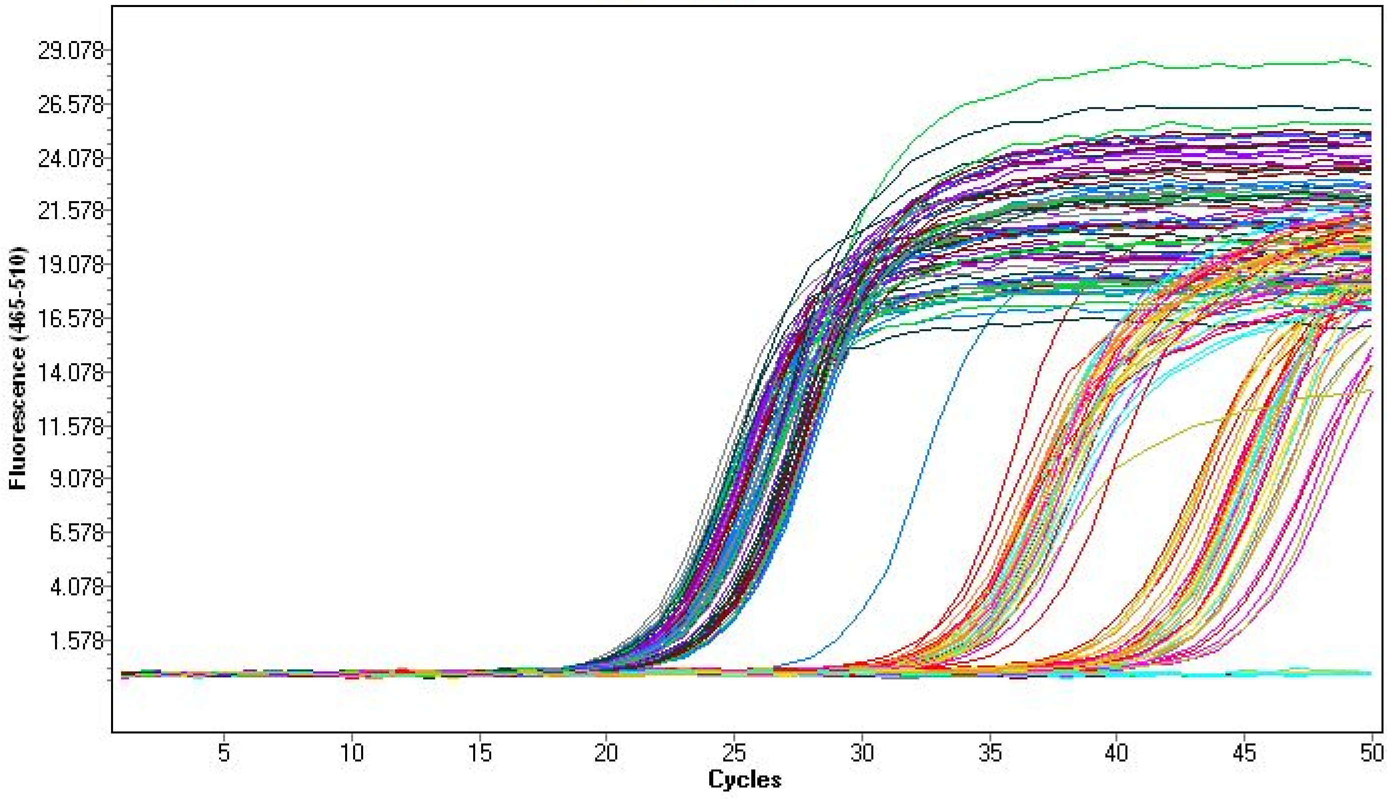

Supplement: Supplementary file 1 [file ijms-26-07889-s001.zip › ijms-3558049-supplementary/Manuscript data/Fig6A data/Exp_1/2014-10-22 HPRT AIT po transfekcji plazmidem+BIRC5+PEN.PDF]
