# Supplementary material for: MicroRNAs and Their Inhibition in Modulating SLC5A8 Expression in the Context of Papillary Thyroid Carcinoma
Source: Int J Mol Sci. 2025 Aug 15;26(16):7889. doi: 10.3390/ijms26167889 (PMC12386254; doi:10.3390/ijms26167889)

Wojtek_2014-11-10_HPRT AIT po transfekcji plazmidem + BIRC5 PEN

Programs

Program Name	pre-incubation		
Cycles	1	Analysis Mode	None

Target (°C)	Acquisition Mode	Hold (hh:mm:ss)	Ramp Rate (°C/s)	Acquisitions (per °C)	Sec Target (°C)	Step size (°C)	Step Delay (cycles)
95	None	00:10:00	4.80		0	0	0

Program Name	amplification		
Cycles	60	Analysis Mode	Quantification

Target (°C)	Acquisition Mode	Hold (hh:mm:ss)	Ramp Rate (°C/s)	Acquisitions (per °C)	Sec Target (°C)	Step size (°C)	Step Delay (cycles)
95	None	00:00:10	4.80		0	0	0
58	None	00:00:05	2.50		0	0	0
72	Single	00:00:08	4.80		0	0	0

Program Name	melting curve		
Cycles	1	Analysis Mode	Melting Curves

Target (°C)	Acquisition Mode	Hold (hh:mm:ss)	Ramp Rate (°C/s)	Acquisitions (per °C)	Sec Target (°C)	Step size (°C)	Step Delay (cycles)
95	None	00:00:05	4.80		0	0	0
65	None	00:01:00	2.50		0	0	0
97	Continuous		0.11	5	0	0	0

Program Name	cooling		
Cycles	1	Analysis Mode	None

Target (°C)	Acquisition Mode	Hold (hh:mm:ss)	Ramp Rate (°C/s)	Acquisitions (per °C)	Sec Target (°C)	Step size (°C)	Step Delay (cycles)
40	None	00:00:30	2.50		0	0	0

Tm Calling for All (Tm Calling)

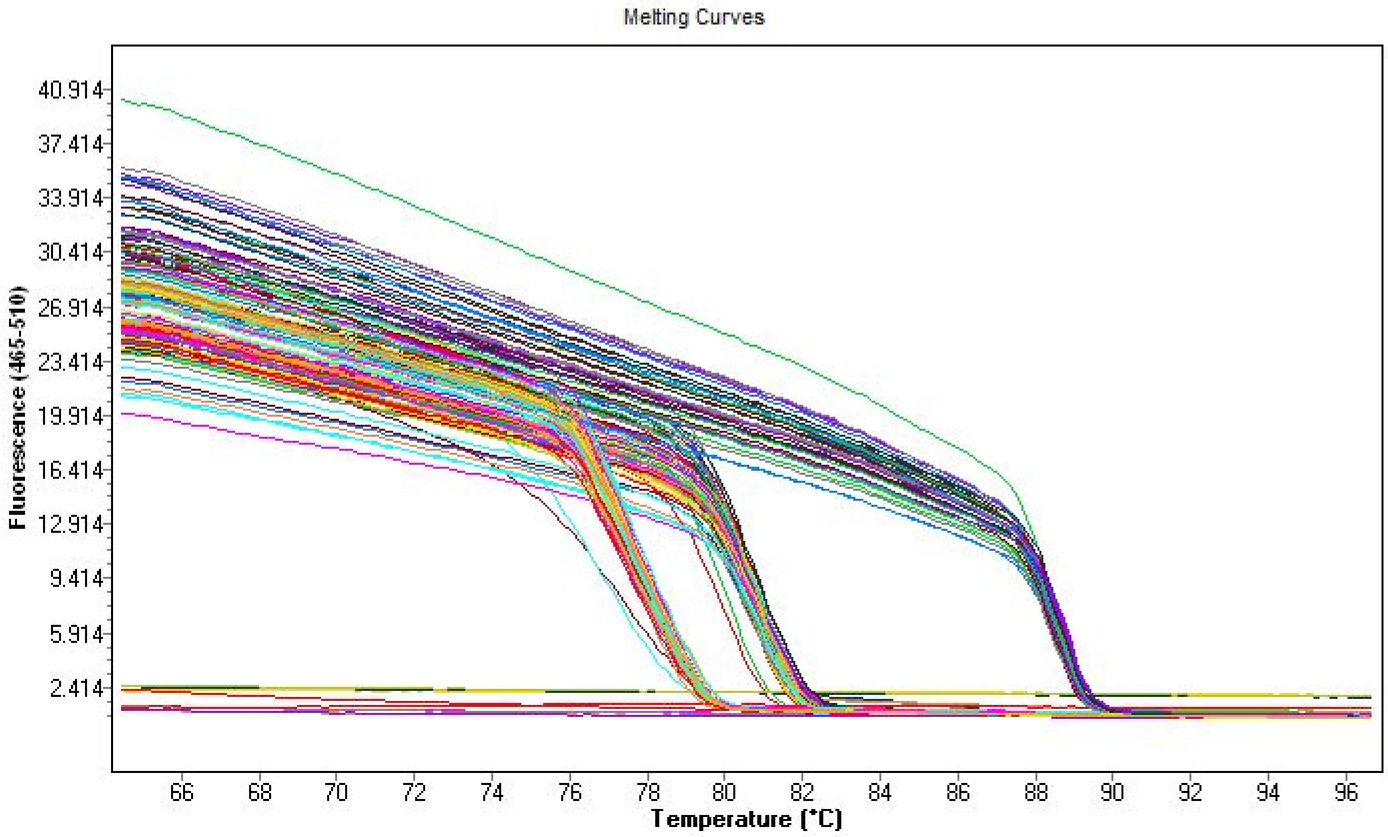

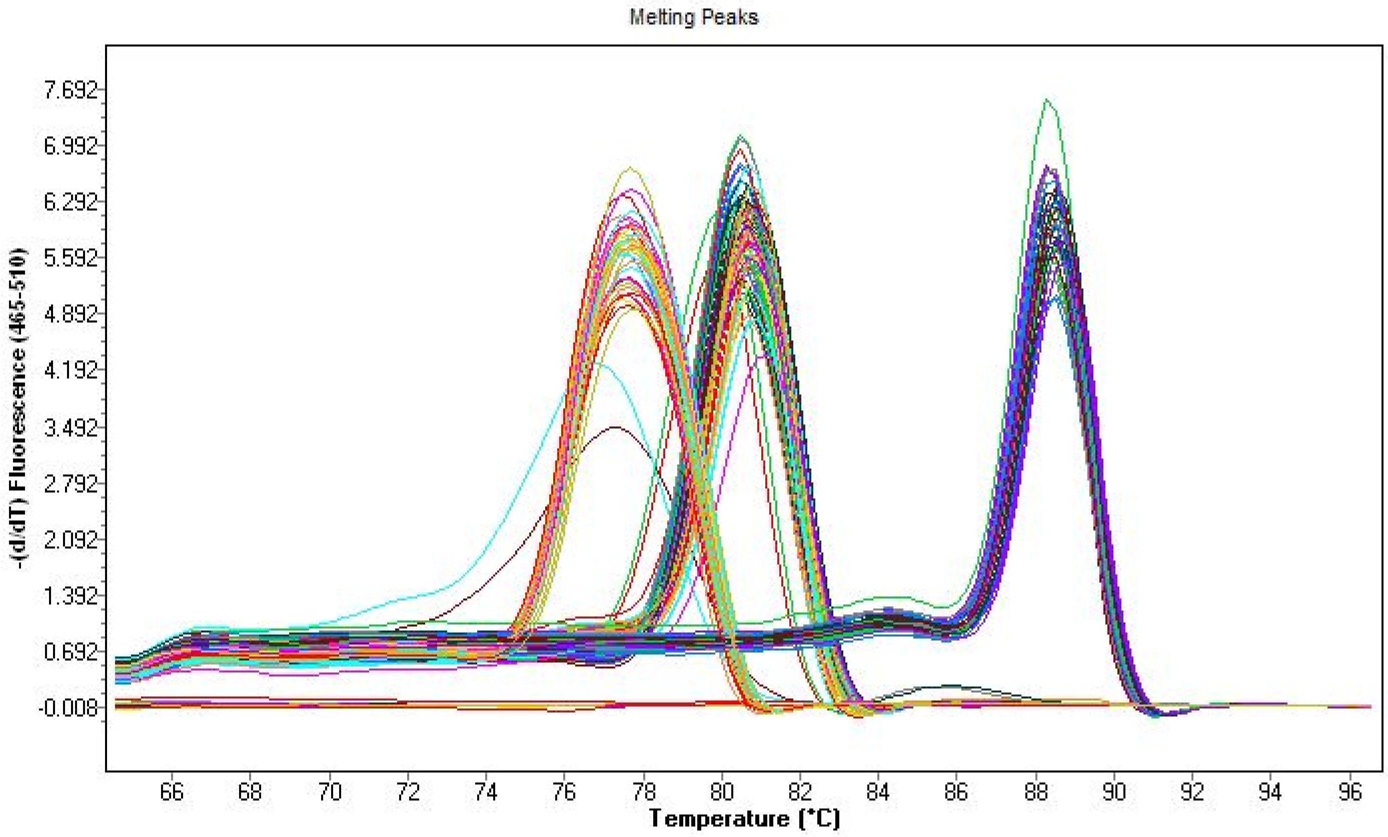

Abs Quant/2nd Derivative Max for All (Abs Quant/2nd Derivative Max)

Statistics

Samples	Mean Cp	Std Cp	Mean conc	Std conc
G14, G15	32.53	0.10		
G17, G18				
G20, G21	37.42			
G23, G24				
H1, I1, J1	21.60	0.05		
H2, I2, J2	41.96	1.78		
H3, I3, J3	21.50	0.04		
H4, I4, J4	40.97	0.45		
H5, I5, J5	21.65	0.02		
H6, I6, J6	41.63	0.59		
H7, I7, J7	21.85	0.11		
H8, I8, J8	42.92	0.99		
H9, I9, J9	21.69	0.08		
H10, I10, J10	41.82	0.70		
H11, I11, J11	21.65	0.06		
H12, I12, J12	42.15	1.45		
H13, I13, J13	21.68	0.06		
H14, I14, J14	42.12	0.63		
H15, I15, J15	21.76	0.11		
H16, I16, J16	43.70	1.72		
H17, I17, J17	21.77	0.16		
H18, I18, J18	41.91	0.35		
H19, I19, J19	21.74	0.08		
H20, I20, J20	43.68	1.75		
H21, I21, J21	21.58	0.13		
H22, I22, J22	40.96	0.14		
H23, I23, J23	21.50	0.01		
H24, I24, J24	41.39	0.24		
K1, L1, M1	22.23	0.28		
K2, L2, M2	32.62	0.19		
K3, L3, M3	21.95	0.17		
K4, L4, M4	32.79	0.17		
K5, L5, M5	22.41	0.17		
K6, L6, M6	32.96	0.09		
K7, L7, M7	22.52	0.08		
K8, L8, M8	33.32	0.18		
K9, L9, M9	22.53	0.12		

Statistics

Samples	Mean Cp	Std Cp	Mean conc	Std conc
K10, L10, M10	33.12	0.30		
K11, L11, M11	22.41	0.22		
K12, L12, M12	32.96	0.41		
K13, L13, M13	22.46	0.19		
K14, L14, M14	33.22	0.37		
K15, L15, M15	22.60	0.10		
K16, L16, M16	32.77	0.37		
K17, L17, M17	22.72	0.09		
K18, L18, M18	33.00	0.17		
K19, L19, M19	22.32	0.09		
K20, L20, M20	32.77	0.18		
K21, L21, M21	22.11	0.03		
K22, L22, M22	32.82	0.17		
K23, L23, M23	21.86	0.07		
K24, L24, M24	32.36	0.19		

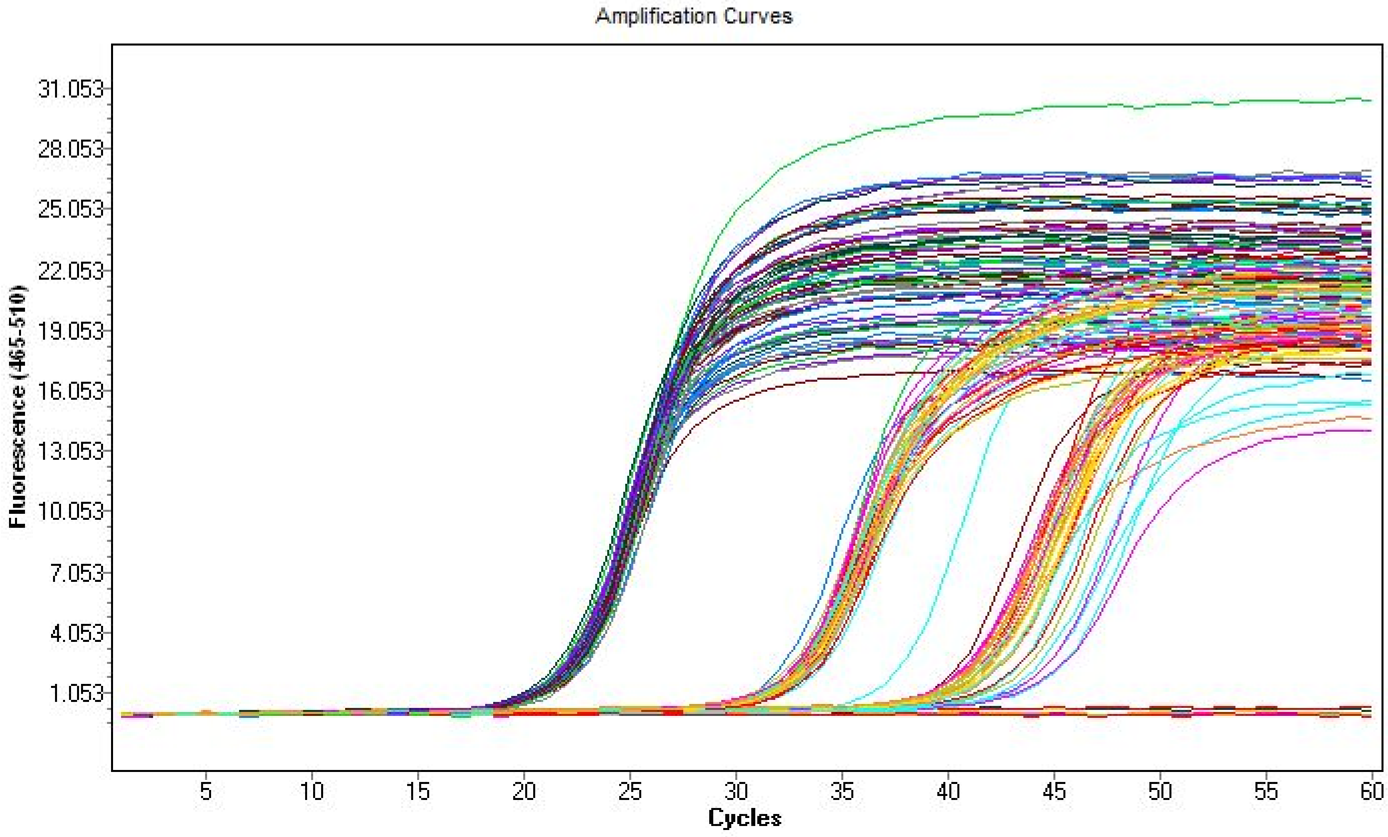

Supplement: Supplementary file 1 [file ijms-26-07889-s001.zip › ijms-3558049-supplementary/Manuscript data/Fig6A data/Exp_2/2014-11-10 HPRT AIT po transfekcji plazmidem+BIRC5+PEN.PDF]
