# Supplementary material for: MicroRNAs and Their Inhibition in Modulating SLC5A8 Expression in the Context of Papillary Thyroid Carcinoma
Source: Int J Mol Sci. 2025 Aug 15;26(16):7889. doi: 10.3390/ijms26167889 (PMC12386254; doi:10.3390/ijms26167889)

Wojtek_2014-11-26_HPRT AIT po transfekcji plazmidem + BIRC5 PEN + Marta PEN

Programs

Program Name	pre-incubation		
Cycles	1	Analysis Mode	None

Target (°C)	Acquisition Mode	Hold (hh:mm:ss)	Ramp Rate (°C/s)	Acquisitions (per °C)	Sec Target (°C)	Step size (°C)	Step Delay (cycles)
95	None	00:10:00	4.80		0	0	0

Program Name	amplification		
Cycles	50	Analysis Mode	Quantification

Target (°C)	Acquisition Mode	Hold (hh:mm:ss)	Ramp Rate (°C/s)	Acquisitions (per °C)	Sec Target (°C)	Step size (°C)	Step Delay (cycles)
95	None	00:00:10	4.80		0	0	0
58	None	00:00:05	2.50		0	0	0
72	Single	00:00:08	4.80		0	0	0

Program Name	melting curve		
Cycles	1	Analysis Mode	Melting Curves

Target (°C)	Acquisition Mode	Hold (hh:mm:ss)	Ramp Rate (°C/s)	Acquisitions (per °C)	Sec Target (°C)	Step size (°C)	Step Delay (cycles)
95	None	00:00:05	4.80		0	0	0
65	None	00:01:00	2.50		0	0	0
97	Continuous		0.11	5	0	0	0

Program Name	cooling		
Cycles	1	Analysis Mode	None

Target (°C)	Acquisition Mode	Hold (hh:mm:ss)	Ramp Rate (°C/s)	Acquisitions (per °C)	Sec Target (°C)	Step size (°C)	Step Delay (cycles)
40	None	00:00:30	2.50		0	0	0

Tm Calling for All (Tm Calling)

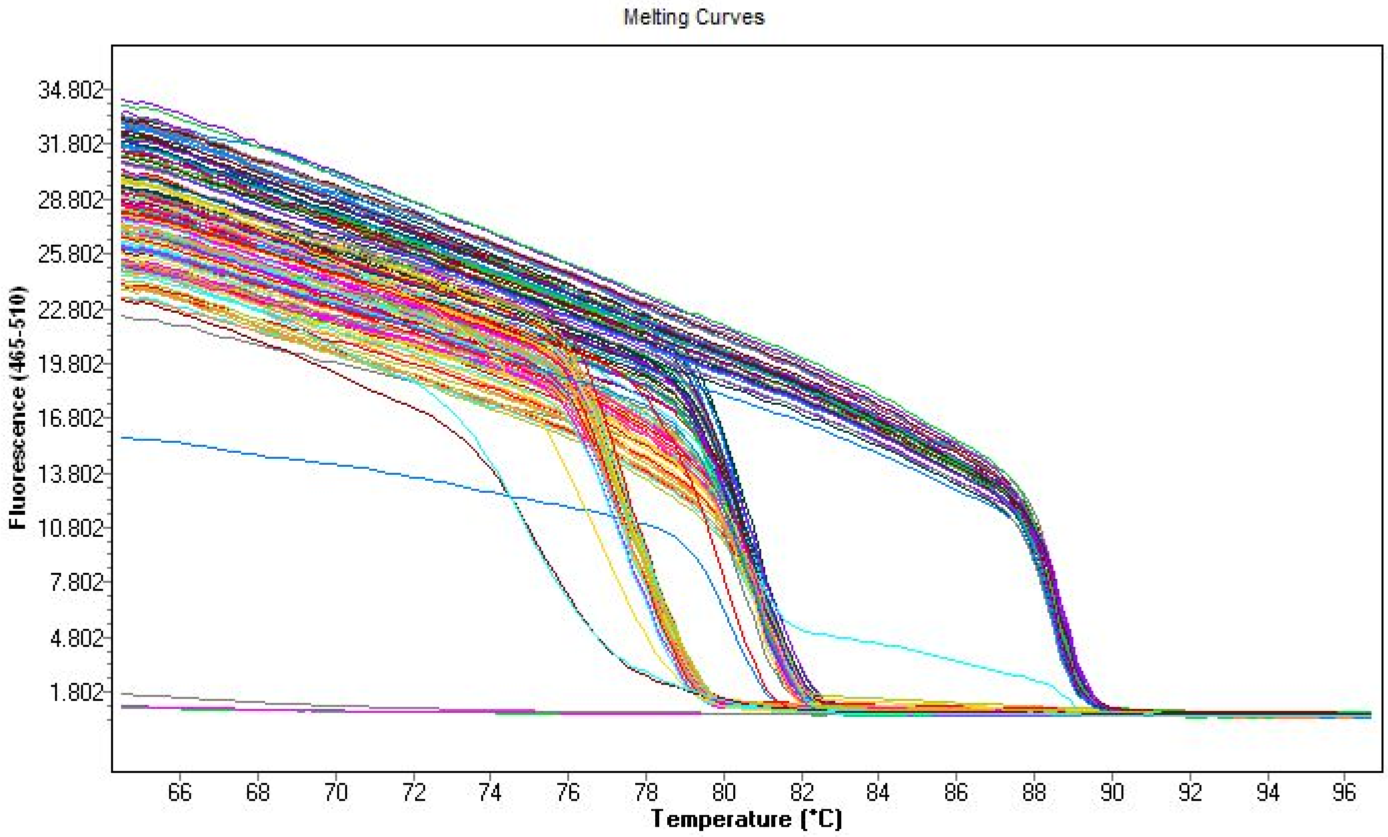

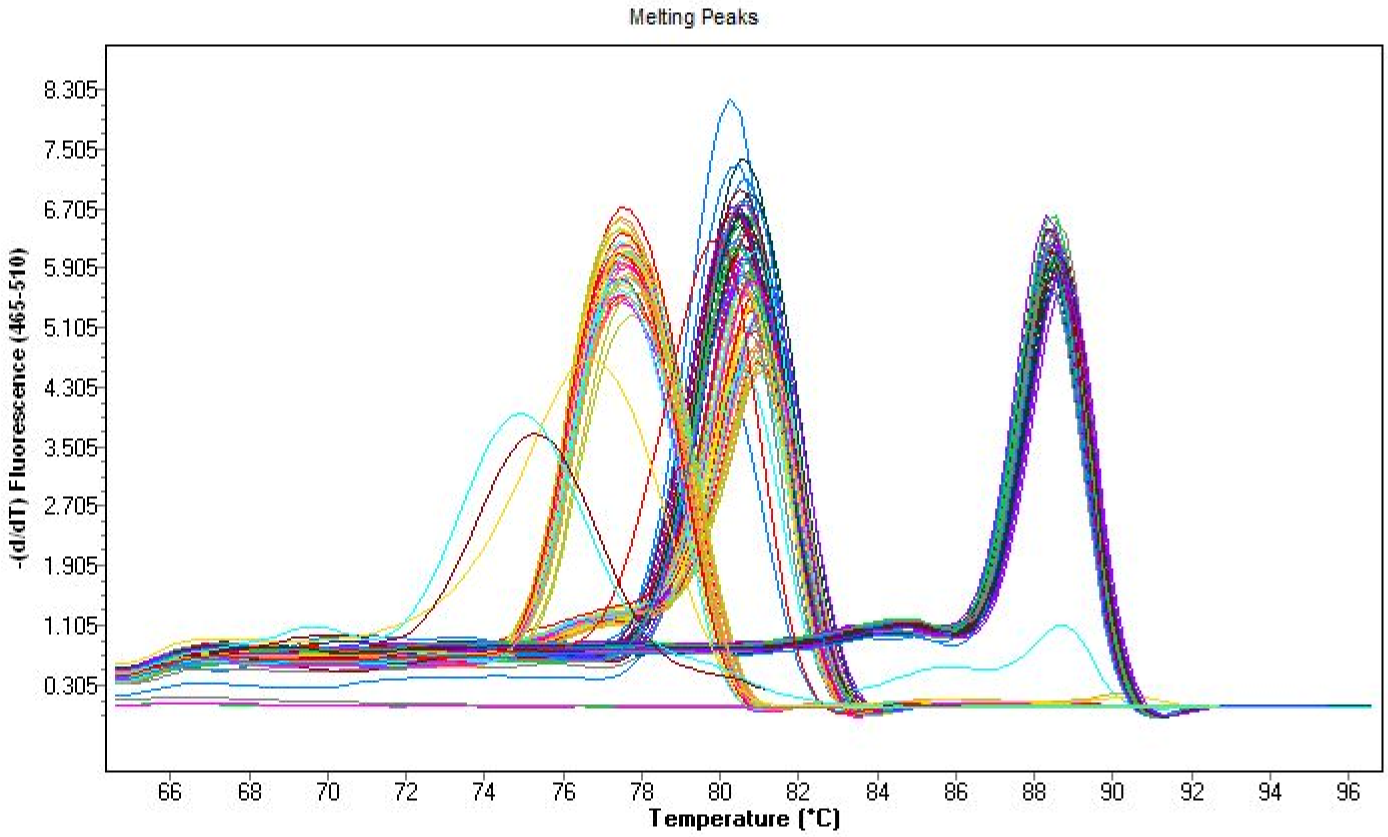

Abs Quant/2nd Derivative Max for All (Abs Quant/2nd Derivative Max)

Statistics

Samples	Mean Cp	Std Cp	Mean conc	Std conc
A1, B1, C1	21.59	0.32		
A2, B2, C2	41.33	0.78		
A3, B3, C3	21.61	0.14		
A4, B4, C4	41.46	0.45		
A5, B5, C5	21.75	0.17		
A6, B6, C6	41.49	0.33		
A7, B7, C7	21.83	0.10		
A8, B8, C8	42.21	0.38		
A9, B9, C9	21.81	0.14		
A10, B10, C10	41.99	0.93		
A11, B11, C11	21.71	0.14		
A12, B12, C12	41.51	0.11		
A13, B13, C13	21.91	0.19		
A14, B14, C14	42.05	1.52		
A15, B15, C15	21.59	0.10		
A16, B16, C16	41.75	0.84		
A17, B17, C17	21.75	0.07		
A18, B18, C18	41.59	0.87		
A19, B19, C19	21.99	0.27		
A20, B20, C20	41.60	0.89		
A21, B21, C21	22.04	0.10		
A22, B22, C22	43.21	0.91		
A23, B23, C23	21.95	0.16		
A24, B24, C24	43.80	1.13		
D1, E1, F1	21.95	0.08		
D2, E2, F2	32.53	0.26		
D3, E3, F3	21.88	0.10		
D4, E4, F4	32.79	0.20		
D5, E5, F5	22.32	0.11		
D6, E6, F6	33.47	0.16		
D7, E7, F7	22.23	0.07		
D8, E8, F8	33.38	0.43		
D9, E9, F9	22.32	0.22		
D10, E10, F10	33.22	0.04		
D11, E11, F11	22.01	0.10		
D12, E12, F12	32.74	0.24		
D13, E13, F13	22.13	0.18		

Statistics

Samples	Mean Cp	Std Cp	Mean conc	Std conc
D14, E14, F14	33.09	0.04		
D15, E15, F15	21.73	0.10		
D16, E16, F16	32.66	0.16		
D17, E17, F17	22.03	0.21		
D18, E18, F18	32.89	0.28		
D19, E19, F19	22.49	0.09		
D20, E20, F20	33.15	0.15		
D21, E21, F21	22.47	0.14		
D22, E22, F22	33.11	0.06		
D23, E23, F23	22.59	0.10		
D24, E24, F24	33.04	0.18		

Amplification Curves

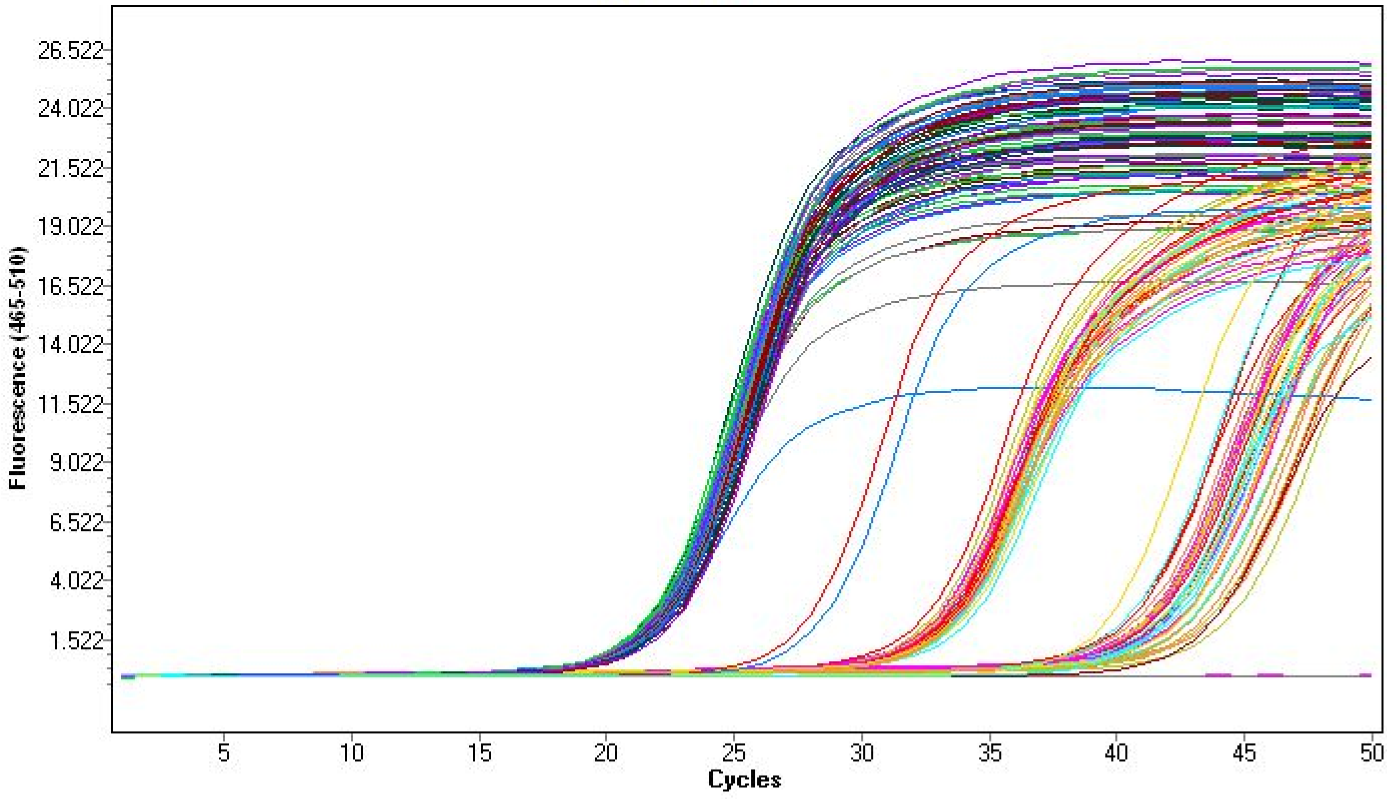

Supplement: Supplementary file 1 [file ijms-26-07889-s001.zip › ijms-3558049-supplementary/Manuscript data/Fig6A data/Exp_3/2014-11-26 HPRT AIT po transfekcji plazmidem+BIRC5+PEN.PDF]
