# Supplementary material for: MicroRNAs and Their Inhibition in Modulating SLC5A8 Expression in the Context of Papillary Thyroid Carcinoma
Source: Int J Mol Sci. 2025 Aug 15;26(16):7889. doi: 10.3390/ijms26167889 (PMC12386254; doi:10.3390/ijms26167889)
Supplement: Supplementary file 1 [file ijms-26-07889-s001.zip › ijms-3558049-supplementary/Manuscript data/Fig6C data/Sponge-mix/Exp_1/2018-09-19 HEK293 sponge-mix.PDF]

LightCycler® 480 Software Report

Roche

2018-09-19_HEK293_s-ctrl+s_mix

Programs

Program Name	pre-incubation		
Cycles	1	Analysis Mode	None

Target (°C)	Acquisition Mode	Hold (hh:mm:ss)	Ramp Rate (°C/s)	Acquisitions (per °C)	Sec Target (°C)	Step size (°C)	Step Delay (cycles)
95	None	00:10:00	4.80		0	0	0

Program Name	amplification		
Cycles	55	Analysis Mode	Quantification

Target (°C)	Acquisition Mode	Hold (hh:mm:ss)	Ramp Rate (°C/s)	Acquisitions (per °C)	Sec Target (°C)	Step size (°C)	Step Delay (cycles)
95	None	00:00:10	4.80		0	0	0
58	None	00:00:15	2.50		0	0	0
72	Single	00:00:10	4.80		0	0	0

Program Name	melting curve		
Cycles	1	Analysis Mode	Melting Curves

Target (°C)	Acquisition Mode	Hold (hh:mm:ss)	Ramp Rate (°C/s)	Acquisitions (per °C)	Sec Target (°C)	Step size (°C)	Step Delay (cycles)
95	None	00:00:05	4.80		0	0	0
65	None	00:01:00	2.50		0	0	0
97	Continuous		0.11	5	0	0	0

Program Name	cooling		
Cycles	1	Analysis Mode	None

Target (°C)	Acquisition Mode	Hold (hh:mm:ss)	Ramp Rate (°C/s)	Acquisitions (per °C)	Sec Target (°C)	Step size (°C)	Step Delay (cycles)
40	None	00:00:30	2.50		0	0	0

Tm Calling for HPRT (Tm Calling)

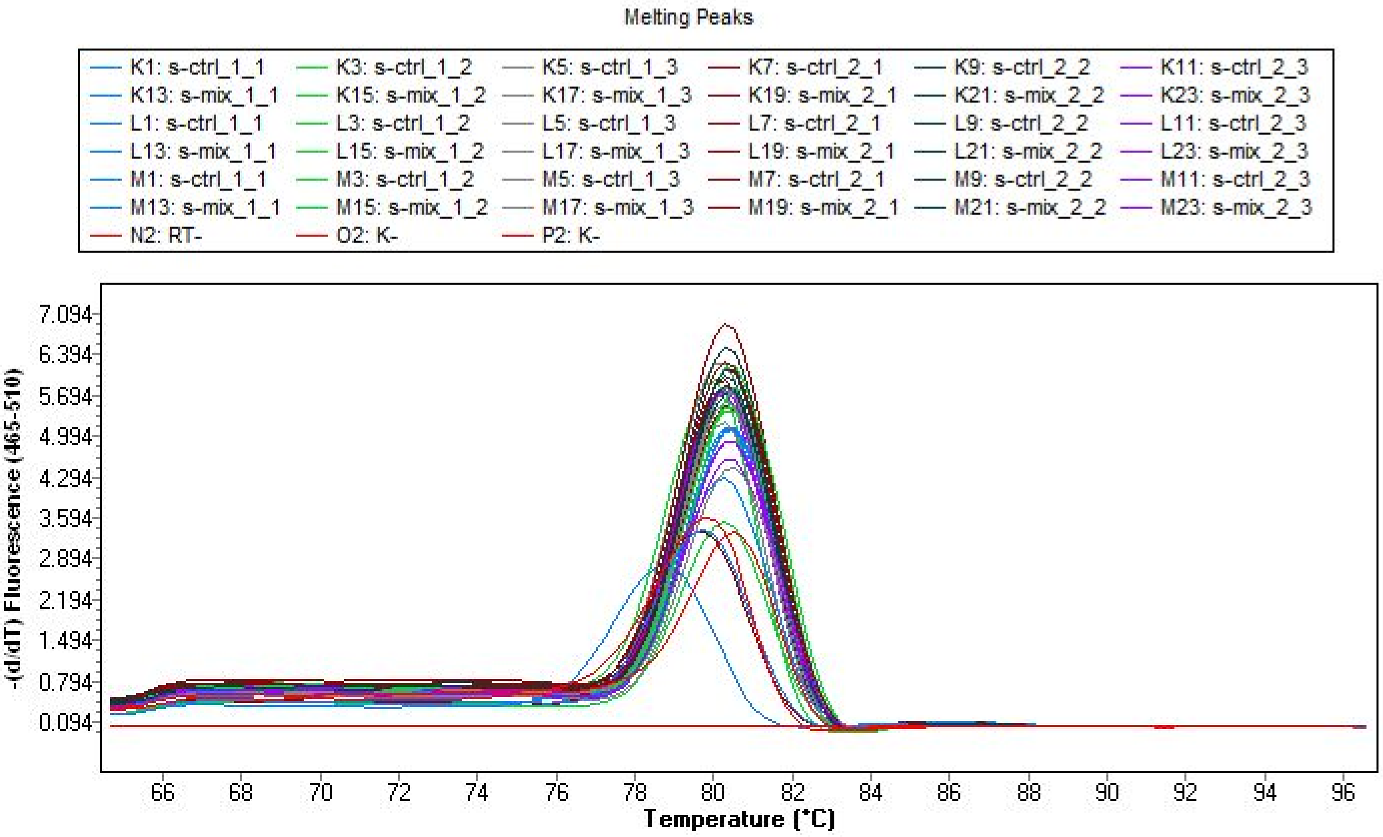

Tm Calling for NIS (Tm Calling)

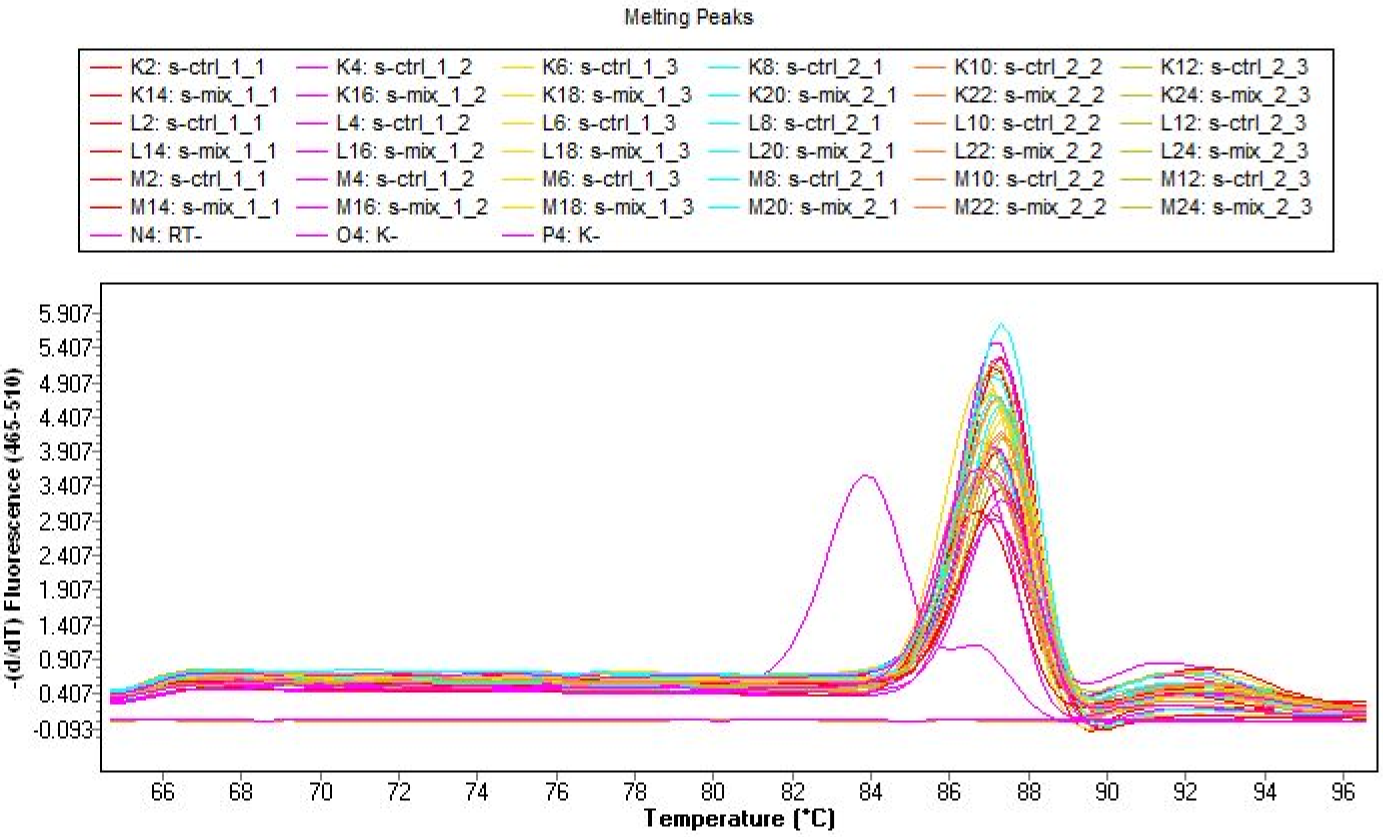

Tm Calling for Pen (Tm Calling)

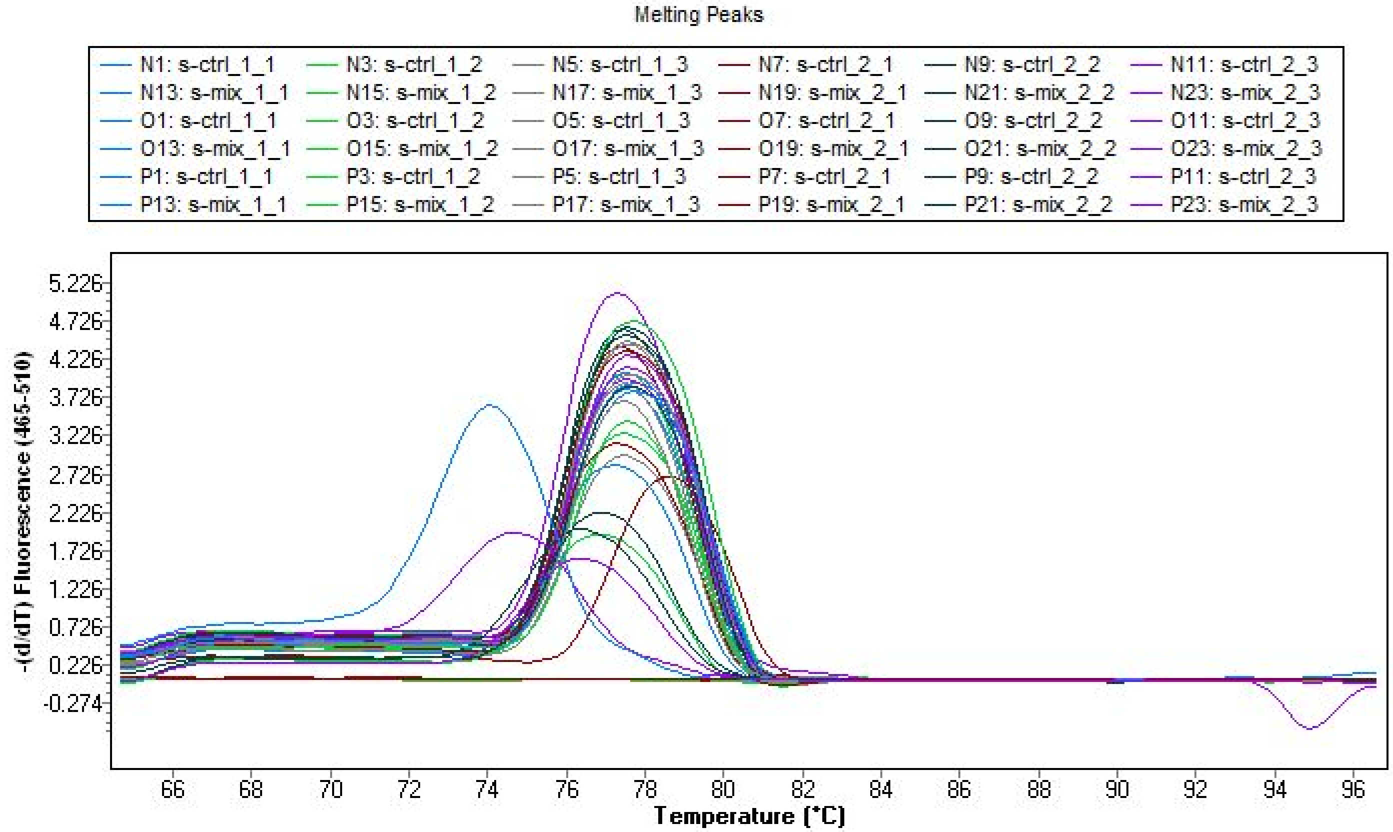

Abs Quant/2nd Derivative Max for HPRT (Abs Quant/2nd Derivative Max)

Results

Inc	Pos	Name	Type	CP	Concentration	Standard	Status
☑	K1	s-ctrl_1_1	Unknown	22.19			
☑	K3	s-ctrl_1_2	Unknown	22.76			
☑	K5	s-ctrl_1_3	Unknown	22.34			
☑	K7	s-ctrl_2_1	Unknown	23.66			
☑	K9	s-ctrl_2_2	Unknown	23.97			
☑	K11	s-ctrl_2_3	Unknown	23.09			
☑	K13	s-mix_1_1	Unknown	23.18			
☑	K15	s-mix_1_2	Unknown	30.84			
☑	K17	s-mix_1_3	Unknown	23.13			
☑	K19	s-mix_2_1	Unknown	22.45			
☑	K21	s-mix_2_2	Unknown	22.27			
☑	K23	s-mix_2_3	Unknown	23.10			
☑	L1	s-ctrl_1_1	Unknown	22.24			
☑	L3	s-ctrl_1_2	Unknown	22.87			
☑	L5	s-ctrl_1_3	Unknown	22.50			

Results

Inc	Pos	Name	Type	CP	Concentration	Standard	Status
☑	L7	s-ctrl_2_1	Unknown	23.75			
☑	L9	s-ctrl_2_2	Unknown	24.12			
☑	L11	s-ctrl_2_3	Unknown	23.20			
☑	L13	s-mix_1_1	Unknown	21.86			
☑	L15	s-mix_1_2	Unknown	29.84			
☑	L17	s-mix_1_3	Unknown	22.17			
☑	L19	s-mix_2_1	Unknown	22.67			
☑	L21	s-mix_2_2	Unknown	22.33			
☑	L23	s-mix_2_3	Unknown	23.07			
☑	M1	s-ctrl_1_1	Unknown	22.22			
☑	M3	s-ctrl_1_2	Unknown	22.81			
☑	M5	s-ctrl_1_3	Unknown	22.51			
☑	M7	s-ctrl_2_1	Unknown	23.72			
☑	M9	s-ctrl_2_2	Unknown	24.00			
☑	M11	s-ctrl_2_3	Unknown	23.17			
☑	M13	s-mix_1_1	Unknown	21.69			
☑	M15	s-mix_1_2	Unknown	29.57			
☑	M17	s-mix_1_3	Unknown	22.08			
☑	M19	s-mix_2_1	Unknown	22.27			
☑	M21	s-mix_2_2	Unknown	22.26			
☑	M23	s-mix_2_3	Unknown	23.08			
☑	N2	RT-	Unknown	32.73			
☑	O2	K-	Unknown	33.30			
☑	P2	K-	Unknown				

Amplification Curves

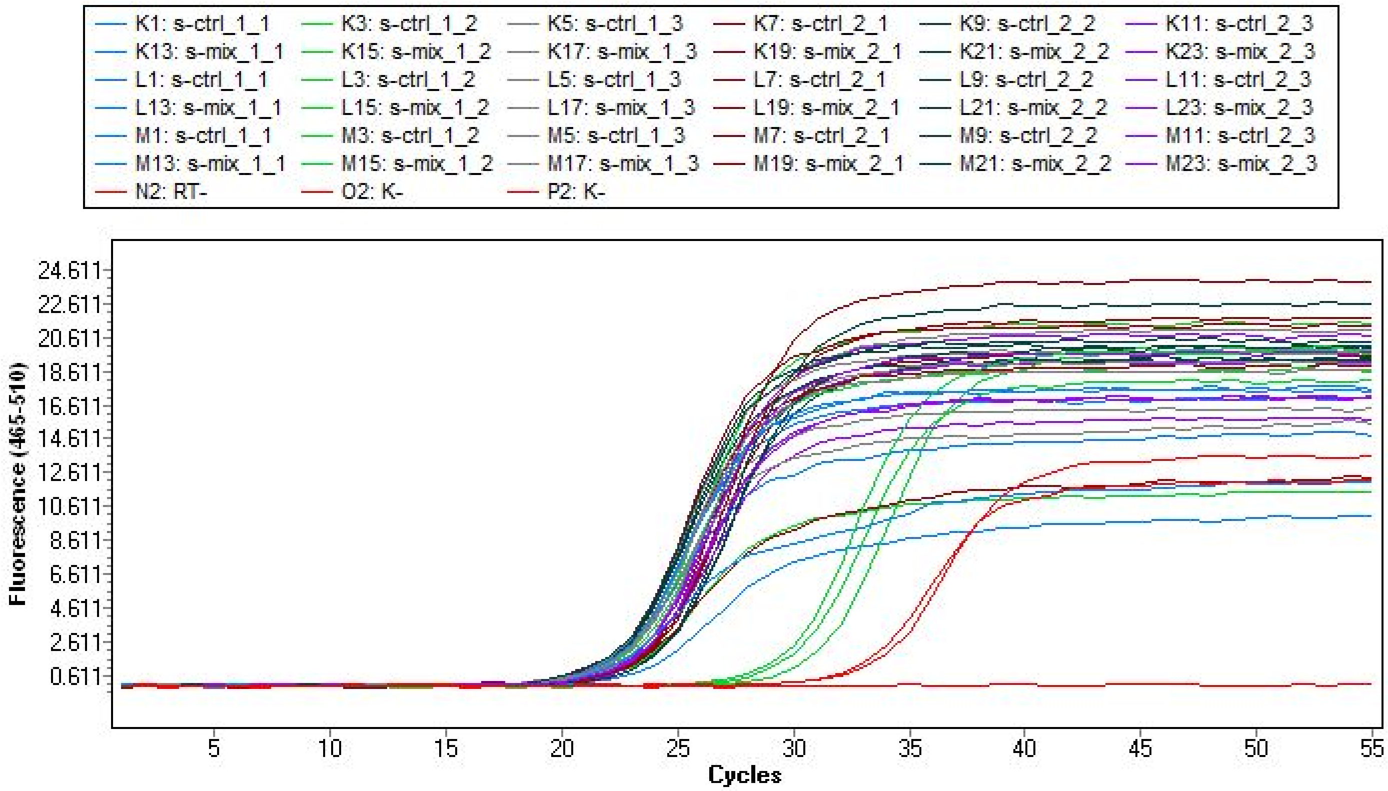

Abs Quant/2nd Derivative Max for NIS (Abs Quant/2nd Derivative Max)

Results

Inc	Pos	Name	Type	CP	Concentration	Standard	Status
☑	K2	s-ctrl_1_1	Unknown	26.86			
☑	K4	s-ctrl_1_2	Unknown	25.93			
☑	K6	s-ctrl_1_3	Unknown	26.89			
☑	K8	s-ctrl_2_1	Unknown	26.51			
☑	K10	s-ctrl_2_2	Unknown				
☑	K12	s-ctrl_2_3	Unknown	26.98			
☑	K14	s-mix_1_1	Unknown	27.20			
☑	K16	s-mix_1_2	Unknown	27.14			
☑	K18	s-mix_1_3	Unknown	27.02			
☑	K20	s-mix_2_1	Unknown	27.01			
☑	K22	s-mix_2_2	Unknown	27.21			
☑	K24	s-mix_2_3	Unknown	27.01			
☑	L2	s-ctrl_1_1	Unknown	26.91			
☑	L4	s-ctrl_1_2	Unknown	26.61			
☑	L6	s-ctrl_1_3	Unknown	26.98			
☑	L8	s-ctrl_2_1	Unknown	26.77			
☑	L10	s-ctrl_2_2	Unknown	22.07			

Results

Inc	Pos	Name	Type	CP	Concentration	Standard	Status
☑	L12	s-ctrl_2_3	Unknown	27.19			
☑	L14	s-mix_1_1	Unknown	27.13			
☑	L16	s-mix_1_2	Unknown	27.50			
☑	L18	s-mix_1_3	Unknown	27.01			
☑	L20	s-mix_2_1	Unknown	27.03			
☑	L22	s-mix_2_2	Unknown	27.04			
☑	L24	s-mix_2_3	Unknown	27.04			
☑	M2	s-ctrl_1_1	Unknown	26.88			
☑	M4	s-ctrl_1_2	Unknown	26.20			
☑	M6	s-ctrl_1_3	Unknown	26.93			
☑	M8	s-ctrl_2_1	Unknown	26.83			
☑	M10	s-ctrl_2_2	Unknown	22.45			
☑	M12	s-ctrl_2_3	Unknown	27.05			
☑	M14	s-mix_1_1	Unknown	22.66			
☑	M16	s-mix_1_2	Unknown	27.22			
☑	M18	s-mix_1_3	Unknown	26.99			
☑	M20	s-mix_2_1	Unknown	27.00			
☑	M22	s-mix_2_2	Unknown	27.10			
☑	M24	s-mix_2_3	Unknown	21.48			
☑	N4	RT-	Unknown	26.77			
☑	O4	K-	Unknown	26.80			
☑	P4	K-	Unknown				

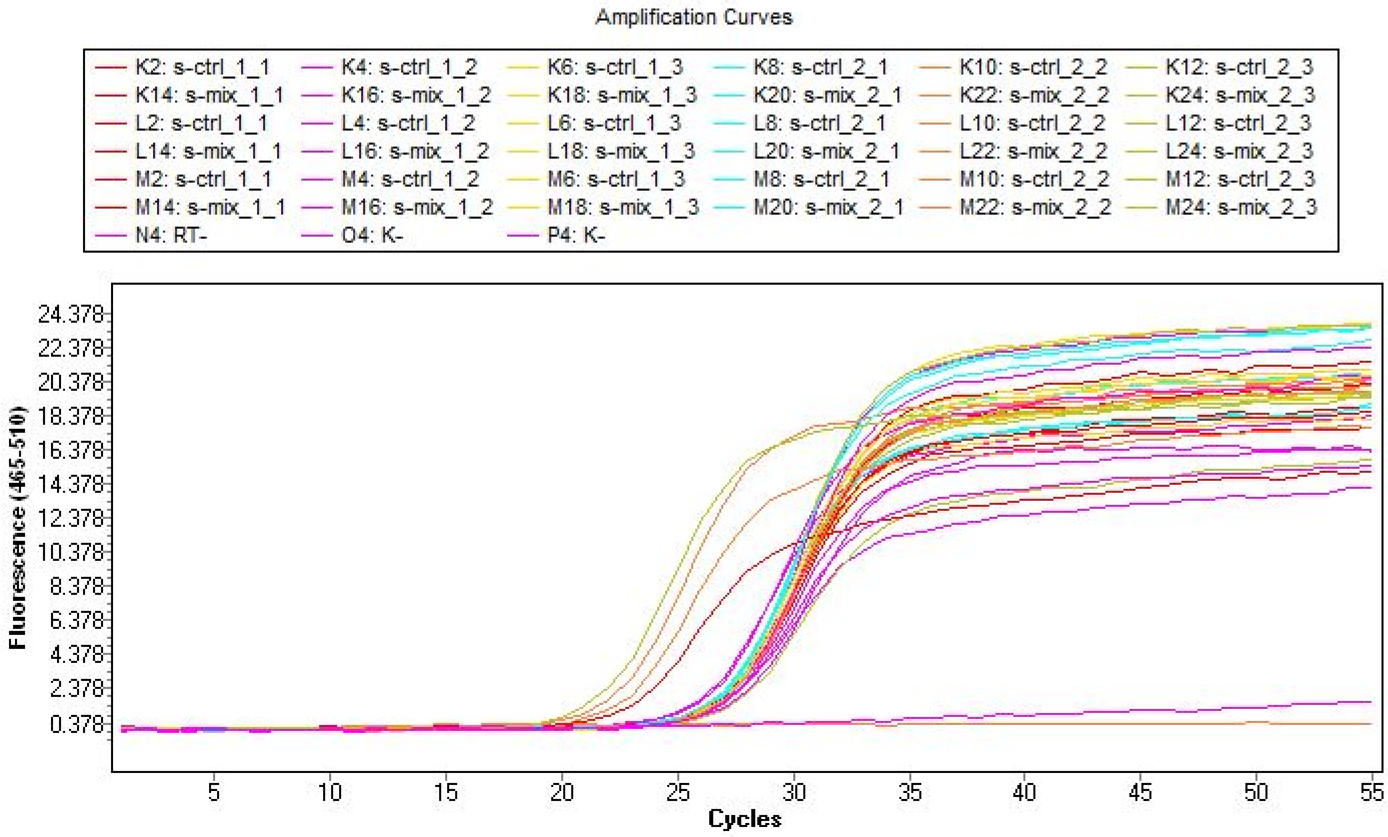

Abs Quant/2nd Derivative Max for Pen (Abs Quant/2nd Derivative Max)

Results

Inc	Pos	Name	Type	CP	Concentration	Standard	Status
☑	N1	s-ctrl_1_1	Unknown	31.26			
☑	N3	s-ctrl_1_2	Unknown	32.17			
☑	N5	s-ctrl_1_3	Unknown	31.89			
☑	N7	s-ctrl_2_1	Unknown	33.18			
☑	N9	s-ctrl_2_2	Unknown	34.07			
☑	N11	s-ctrl_2_3	Unknown	32.69			
☑	N13	s-mix_1_1	Unknown	30.98			
☑	N15	s-mix_1_2	Unknown				
☑	N17	s-mix_1_3	Unknown	32.12			
☑	N19	s-mix_2_1	Unknown	30.92			
☑	N21	s-mix_2_2	Unknown	31.47			
☑	N23	s-mix_2_3	Unknown	32.00			
☑	O1	s-ctrl_1_1	Unknown	31.53			
☑	O3	s-ctrl_1_2	Unknown	32.63			
☑	O5	s-ctrl_1_3	Unknown	32.21			
☑	O7	s-ctrl_2_1	Unknown	33.45			
☑	O9	s-ctrl_2_2	Unknown	33.75			

Results

Inc	Pos	Name	Type	CP	Concentration	Standard	Status
☑	O11	s-ctrl_2_3	Unknown	33.30			
☑	O13	s-mix_1_1	Unknown	31.03			
☑	O15	s-mix_1_2	Unknown	36.61			
☑	O17	s-mix_1_3	Unknown	32.11			
☑	O19	s-mix_2_1	Unknown				
☑	O21	s-mix_2_2	Unknown	31.27			
☑	O23	s-mix_2_3	Unknown	32.68			
☑	P1	s-ctrl_1_1	Unknown	30.97			
☑	P3	s-ctrl_1_2	Unknown	32.73			
☑	P5	s-ctrl_1_3	Unknown	31.35			
☑	P7	s-ctrl_2_1	Unknown	33.05			
☑	P9	s-ctrl_2_2	Unknown	33.67			
☑	P11	s-ctrl_2_3	Unknown	33.88			
☑	P13	s-mix_1_1	Unknown				
☑	P15	s-mix_1_2	Unknown	36.71			
☑	P17	s-mix_1_3	Unknown	31.98			
☑	P19	s-mix_2_1	Unknown				
☑	P21	s-mix_2_2	Unknown	31.75			
☑	P23	s-mix_2_3	Unknown	31.84			

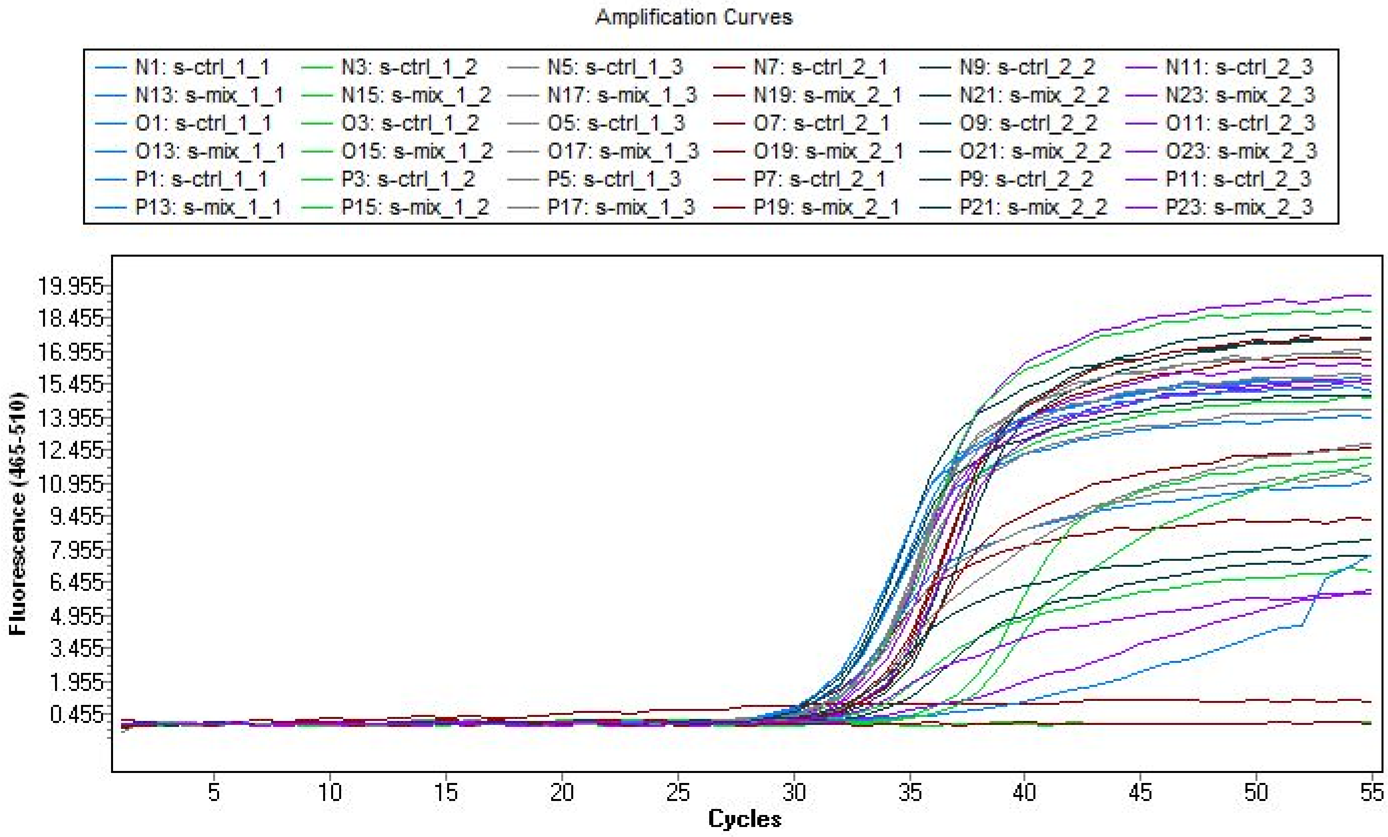
Amplification Curves
N1: s-ctrl_1_1
N3: s-ctrl_1_2
N5: s-ctrl_1_3
N7: s-ctrl_2_1
N9: s-ctrl_2_2
N11: s-ctrl_2_3
N13: s-mix_1_1
N15: s-mix_1_2
N17: s-mix_1_3
N19: s-mix_2_1
N21: s-mix_2_2
N23: s-mix_2_3
O1: s-ctrl_1_1
O3: s-ctrl_1_2
O5: s-ctrl_1_3
O7: s-ctrl_2_1
O9: s-ctrl_2_2
O11: s-ctrl_2_3
O13: s-mix_1_1
O15: s-mix_1_2
O17: s-mix_1_3
O19: s-mix_2_1
O21: s-mix_2_2
O23: s-mix_2_3
P1: s-ctrl_1_1
P3: s-ctrl_1_2
P5: s-ctrl_1_3
P7: s-ctrl_2_1
P9: s-ctrl_2_2
P11: s-ctrl_2_3
P13: s-mix_1_1
P15: s-mix_1_2
P17: s-mix_1_3
P19: s-mix_2_1
P21: s-mix_2_2
P23: s-mix_2_3
Fluorescence (465-510)
19.955
18.455
16.955
15.455
13.955
12.455
10.955
9.455
7.955
6.455
4.955
3.455
1.955
0.455
Cycles
5
10
15
20
25
30
35
40
45
50
55
