# Supplementary material for: MicroRNAs and Their Inhibition in Modulating SLC5A8 Expression in the Context of Papillary Thyroid Carcinoma
Source: Int J Mol Sci. 2025 Aug 15;26(16):7889. doi: 10.3390/ijms26167889 (PMC12386254; doi:10.3390/ijms26167889)
Supplement: Supplementary file 1 [file ijms-26-07889-s001.zip › ijms-3558049-supplementary/Manuscript data/Fig6C data/Sponge-mix/Exp_2/2019-01-08 HEK293 po sponge-mix i V5-AIT.PDF]

Roche

Wojtek_2019-01-08 HEK293 po sponge-mix i V5-AIT

Programs

Program Name	pre-incubation		
Cycles	1	Analysis Mode	None

Target (°C)	Acquisition Mode	Hold (hh:mm:ss)	Ramp Rate (°C/s)	Acquisitions (per °C)	Sec Target (°C)	Step size (°C)	Step Delay (cycles)
95	None	00:10:00	4.80		0	0	0

Program Name	amplification		
Cycles	55	Analysis Mode	Quantification

Target (°C)	Acquisition Mode	Hold (hh:mm:ss)	Ramp Rate (°C/s)	Acquisitions (per °C)	Sec Target (°C)	Step size (°C)	Step Delay (cycles)
95	None	00:00:10	4.80		0	0	0
58	None	00:00:15	2.50		0	0	0
72	Single	00:00:10	4.80		0	0	0

Program Name	melting curve		
Cycles	1	Analysis Mode	Melting Curves

Target (°C)	Acquisition Mode	Hold (hh:mm:ss)	Ramp Rate (°C/s)	Acquisitions (per °C)	Sec Target (°C)	Step size (°C)	Step Delay (cycles)
95	None	00:00:05	4.80		0	0	0
65	None	00:01:00	2.50		0	0	0
97	Continuous		0.11	5	0	0	0

Program Name	cooling		
Cycles	1	Analysis Mode	None

Target (°C)	Acquisition Mode	Hold (hh:mm:ss)	Ramp Rate (°C/s)	Acquisitions (per °C)	Sec Target (°C)	Step size (°C)	Step Delay (cycles)
40	None	00:00:30	2.50		0	0	0

Tm Calling for HPRT (Tm Calling)

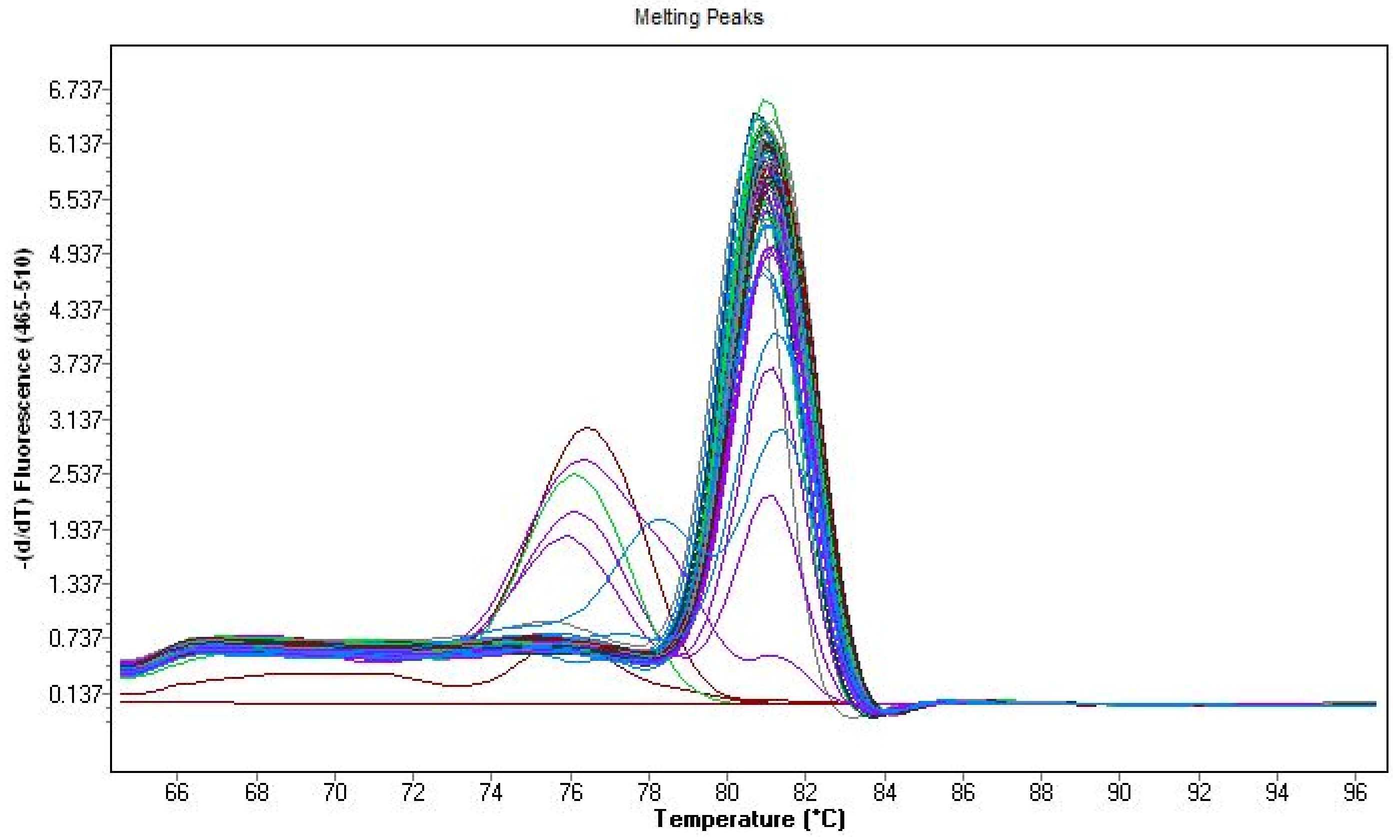

Tm Calling for AIT (Tm Calling)

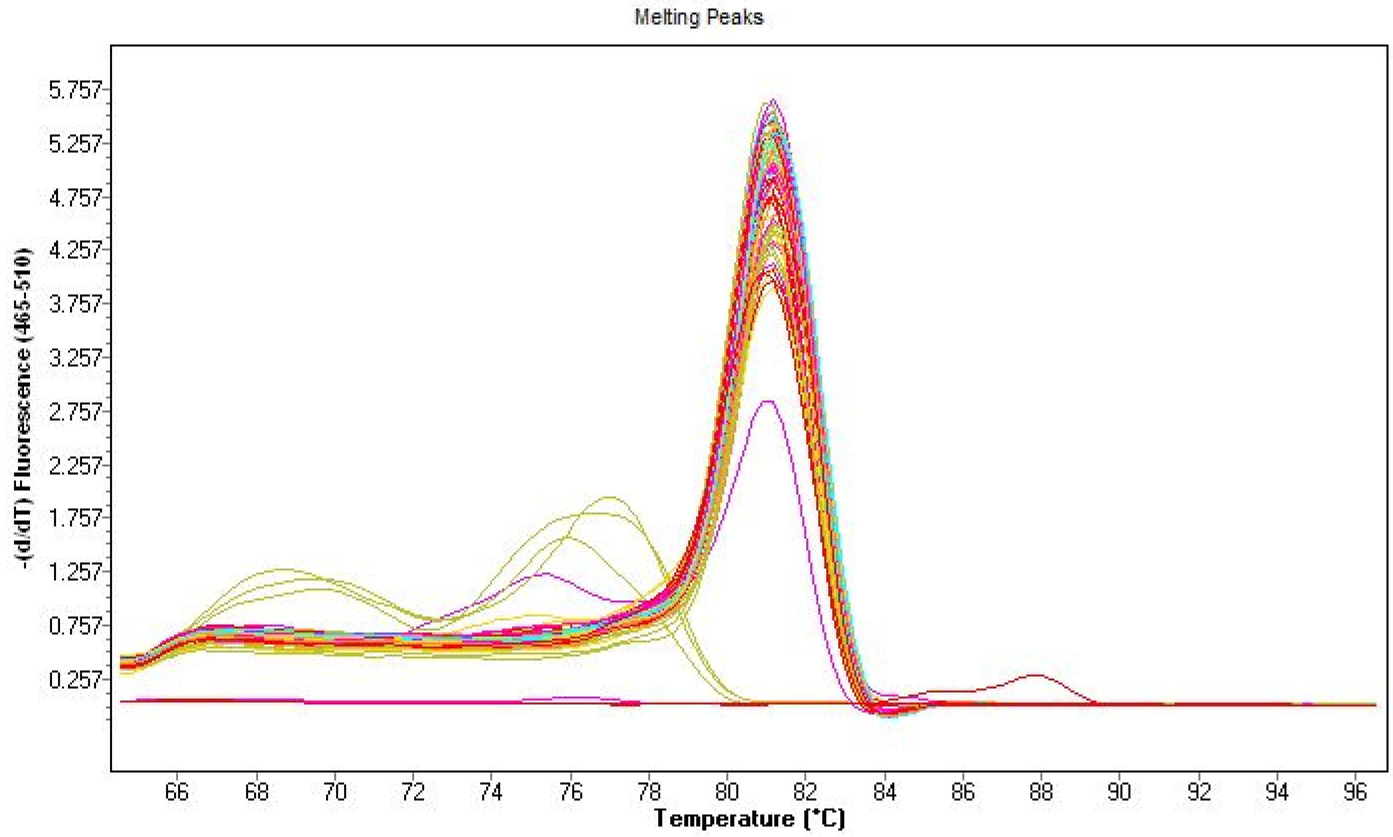

Tm Calling for BIRC5 (Tm Calling)

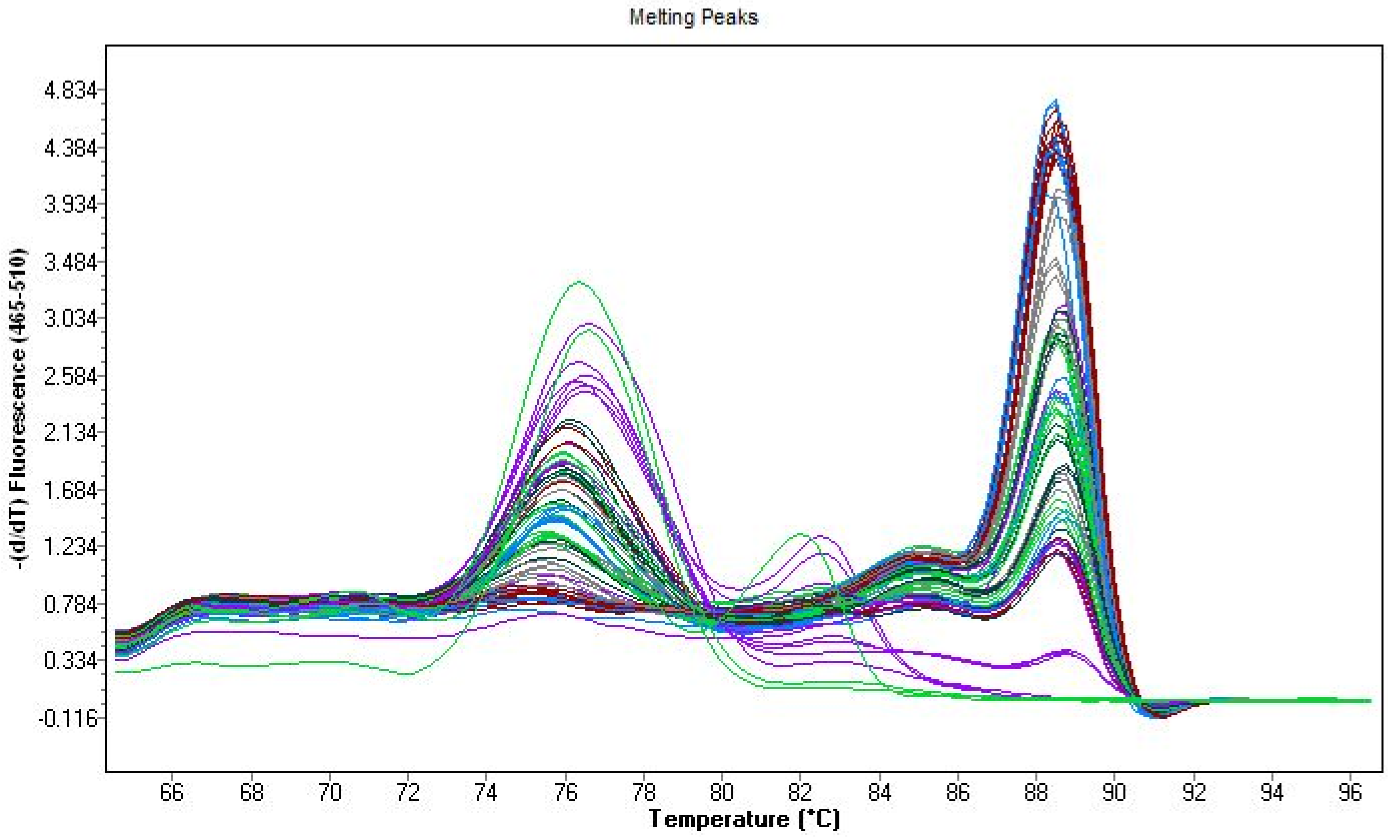

Tm Calling for Pendryna (Tm Calling)

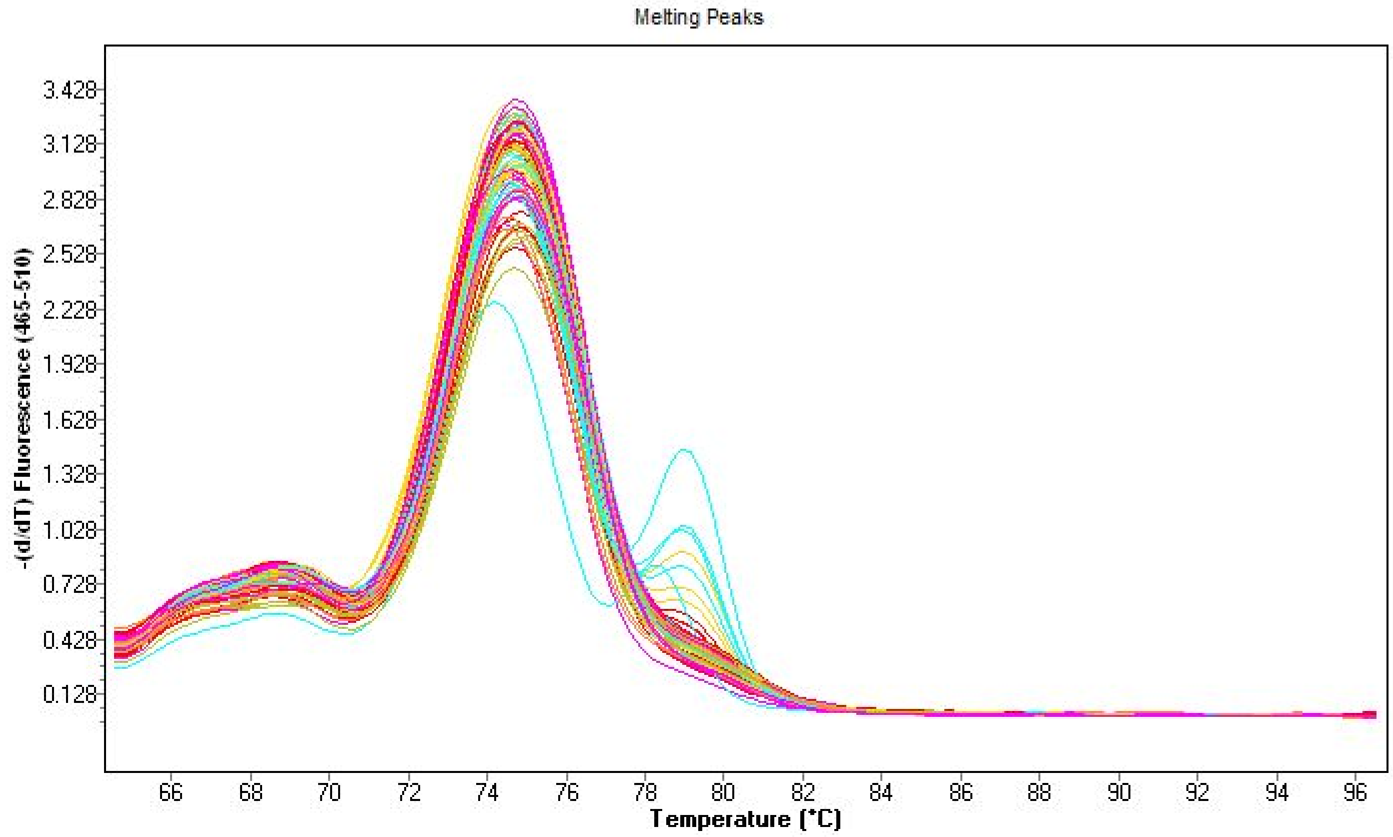

Abs Quant/2nd Derivative Max for S_HPRT (Abs Quant/2nd Derivative Max)

Results

Inc	Pos	Name	Type	CP	Concentration	Standard	Status
☑	A1	s-ctrl_1	Unknown	25.10			
☑	A3	s-ctrl_2	Unknown	24.69			
☑	A5	s-ctrl_3	Unknown	23.71			
☑	A7	s-ctrl_4	Unknown	41.22			
☑	A9	s-ctrl_5	Unknown	24.85			
☑	A11	s-ctrl_6	Unknown	31.28			
☑	A13	s-mix_1	Unknown	24.15			
☑	A15	s-mix_2	Unknown	24.07			
☑	A17	s-mix_3	Unknown	23.69			
☑	A19	s-mix_4	Unknown	23.17			
☑	A21	s-mix_5	Unknown	23.72			
☑	A23	s-mix_6	Unknown	24.27			
☑	B1	s-ctrl_1	Unknown	25.10			
☑	B3	s-ctrl_2	Unknown	24.68			
☑	B5	s-ctrl_3	Unknown	23.67			
☑	B7	s-ctrl_4	Unknown	28.85			
☑	B9	s-ctrl_5	Unknown	24.85			

Results

Inc	Pos	Name	Type	CP	Concentration	Standard	Status
☑	B11	s-ctrl_6	Unknown	32.03			
☑	B13	s-mix_1	Unknown	24.12			
☑	B15	s-mix_2	Unknown	24.09			
☑	B17	s-mix_3	Unknown	23.78			
☑	B19	s-mix_4	Unknown	23.27			
☑	B21	s-mix_5	Unknown	23.65			
☑	B23	s-mix_6	Unknown	24.54			
☑	C1	s-ctrl_1	Unknown	25.17			
☑	C3	s-ctrl_2	Unknown	35.63			
☑	C5	s-ctrl_3	Unknown	23.76			
☑	C7	s-ctrl_4	Unknown				
☑	C9	s-ctrl_5	Unknown	25.22			
☑	C11	s-ctrl_6	Unknown	31.49			
☑	C13	s-mix_1	Unknown	24.20			
☑	C15	s-mix_2	Unknown	24.03			
☑	C17	s-mix_3	Unknown	23.74			
☑	C19	s-mix_4	Unknown	23.22			
☑	C21	s-mix_5	Unknown	23.66			
☑	C23	s-mix_6	Unknown	24.49			
☑	M1	RT-	Unknown	32.70			
☑	N1	K-	Unknown	34.34			
☑	O1	K-	Unknown	32.60			

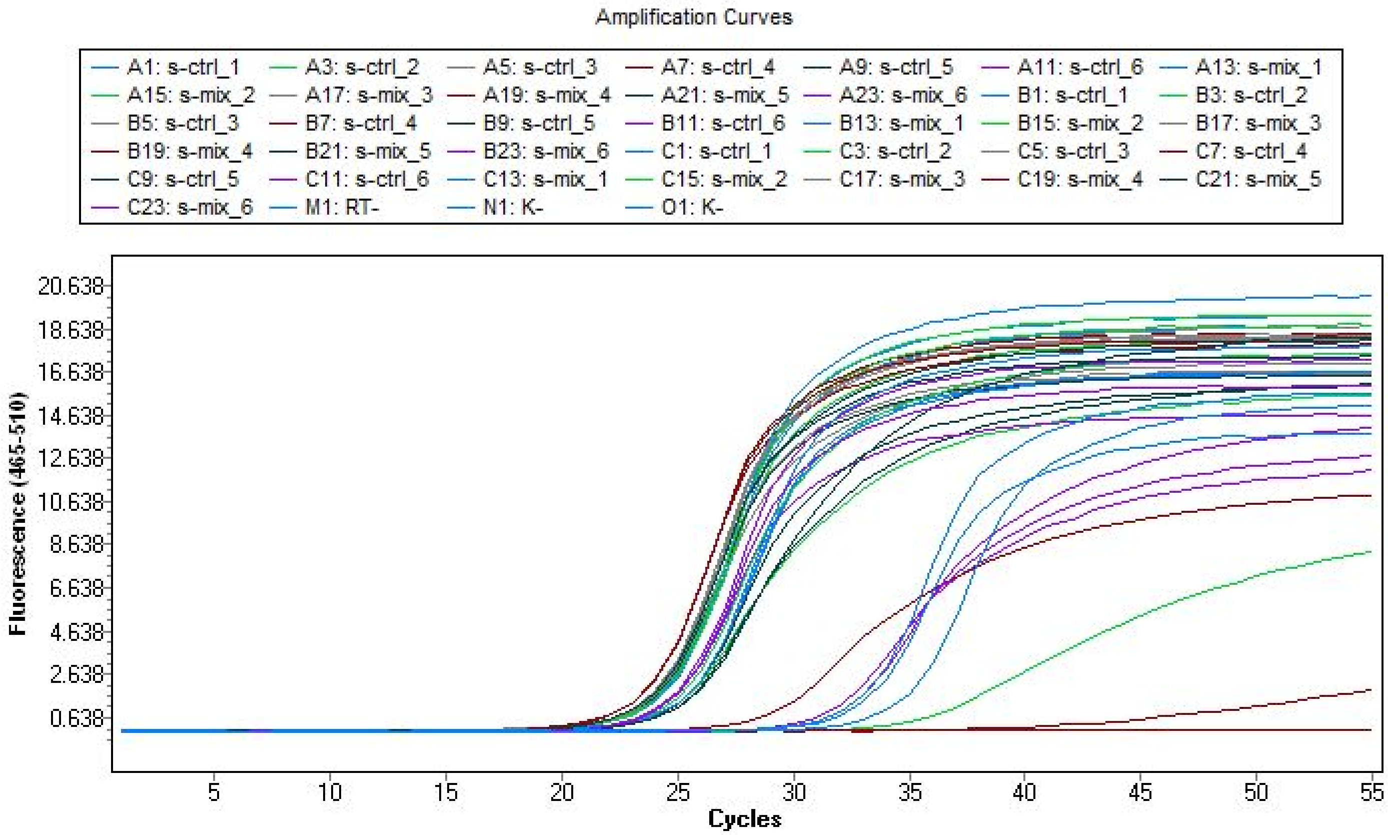

Abs Quant/2nd Derivative Max for S_AIT (Abs Quant/2nd Derivative Max)

Results

Inc	Pos	Name	Type	CP	Concentration	Standard	Status
☑	A2	s-ctrl_1	Unknown	32.53			
☑	A4	s-ctrl_2	Unknown	32.82			
☑	A6	s-ctrl_3	Unknown	31.96			
☑	A8	s-ctrl_4	Unknown	23.91			
☑	A10	s-ctrl_5	Unknown	25.14			
☑	A12	s-ctrl_6	Unknown	34.70			
☑	A14	s-mix_1	Unknown	30.19			
☑	A16	s-mix_2	Unknown	32.19			
☑	A18	s-mix_3	Unknown	32.03			
☑	A20	s-mix_4	Unknown	27.48			
☑	A22	s-mix_5	Unknown	28.84			
☑	A24	s-mix_6	Unknown	26.85			
☑	B2	s-ctrl_1	Unknown	33.61			
☑	B4	s-ctrl_2	Unknown	33.65			
☑	B6	s-ctrl_3	Unknown	32.46			
☑	B8	s-ctrl_4	Unknown	24.08			
☑	B10	s-ctrl_5	Unknown	25.19			

Results

Inc	Pos	Name	Type	CP	Concentration	Standard	Status
☑	B12	s-ctrl_6	Unknown	34.00			
☑	B14	s-mix_1	Unknown	30.61			
☑	B16	s-mix_2	Unknown	32.11			
☑	B18	s-mix_3	Unknown	32.18			
☑	B20	s-mix_4	Unknown	27.29			
☑	B22	s-mix_5	Unknown	29.22			
☑	B24	s-mix_6	Unknown	26.84			
☑	C2	s-ctrl_1	Unknown	32.79			
☑	C4	s-ctrl_2	Unknown				
☑	C6	s-ctrl_3	Unknown	32.49			
☑	C8	s-ctrl_4	Unknown	24.06			
☑	C10	s-ctrl_5	Unknown	25.09			
☑	C12	s-ctrl_6	Unknown	32.90			
☑	C14	s-mix_1	Unknown	30.71			
☑	C16	s-mix_2	Unknown	31.53			
☑	C18	s-mix_3	Unknown	32.25			
☑	C20	s-mix_4	Unknown	27.50			
☑	C22	s-mix_5	Unknown	29.20			
☑	C24	s-mix_6	Unknown	26.98			
☑	M2	RT-	Unknown				
☑	N2	K-	Unknown	46.23			
☑	O2	K-	Unknown				

Amplification Curves

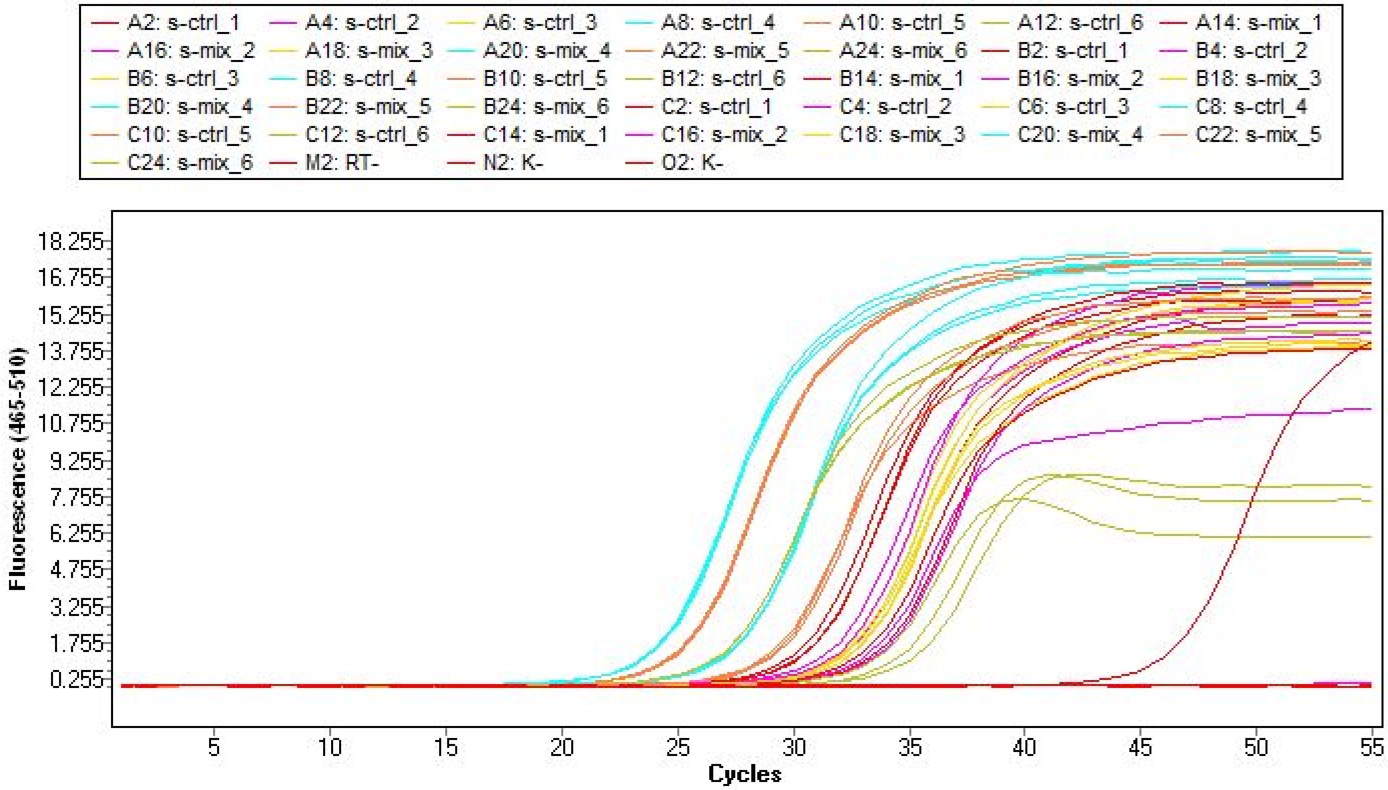

Abs Quant/2nd Derivative Max for S_BIRC5 (Abs Quant/2nd Derivative Max)

Results

Inc	Pos	Name	Type	CP	Concentration	Standard	Status
☑	D1	s-ctrl_1	Unknown	23.54			
☑	D3	s-ctrl_2	Unknown	22.92			
☑	D5	s-ctrl_3	Unknown	22.81			
☑	D7	s-ctrl_4	Unknown	25.18			
☑	D9	s-ctrl_5	Unknown	22.84			
☑	D11	s-ctrl_6	Unknown	23.70			
☑	D13	s-mix_1	Unknown	22.91			
☑	D15	s-mix_2	Unknown	22.29			
☑	D17	s-mix_3	Unknown	22.27			
☑	D19	s-mix_4	Unknown	23.45			
☑	D21	s-mix_5	Unknown	22.95			
☑	D23	s-mix_6	Unknown	23.88			
☑	E1	s-ctrl_1	Unknown	23.49			
☑	E3	s-ctrl_2	Unknown	22.97			
☑	E5	s-ctrl_3	Unknown	22.80			
☑	E7	s-ctrl_4	Unknown	25.17			
☑	E9	s-ctrl_5	Unknown	22.82			

Results

Inc	Pos	Name	Type	CP	Concentration	Standard	Status
☑	E11	s-ctrl_6	Unknown	23.65			
☑	E13	s-mix_1	Unknown	23.00			
☑	E15	s-mix_2	Unknown	22.31			
☑	E17	s-mix_3	Unknown	22.27			
☑	E19	s-mix_4	Unknown	23.46			
☑	E21	s-mix_5	Unknown	22.97			
☑	E23	s-mix_6	Unknown	23.95			
☑	F1	s-ctrl_1	Unknown	23.50			
☑	F3	s-ctrl_2	Unknown	22.95			
☑	F5	s-ctrl_3	Unknown	22.83			
☑	F7	s-ctrl_4	Unknown	25.14			
☑	F9	s-ctrl_5	Unknown	22.84			
☑	F11	s-ctrl_6	Unknown	23.65			
☑	F13	s-mix_1	Unknown	22.99			
☑	F15	s-mix_2	Unknown	22.25			
☑	F17	s-mix_3	Unknown	22.23			
☑	F19	s-mix_4	Unknown	23.47			
☑	F21	s-mix_5	Unknown	22.95			
☑	F23	s-mix_6	Unknown	23.76			
☑	M3	RT-	Unknown	29.62			
☑	N3	K-	Unknown	29.50			
☑	O3	K-	Unknown	29.68			

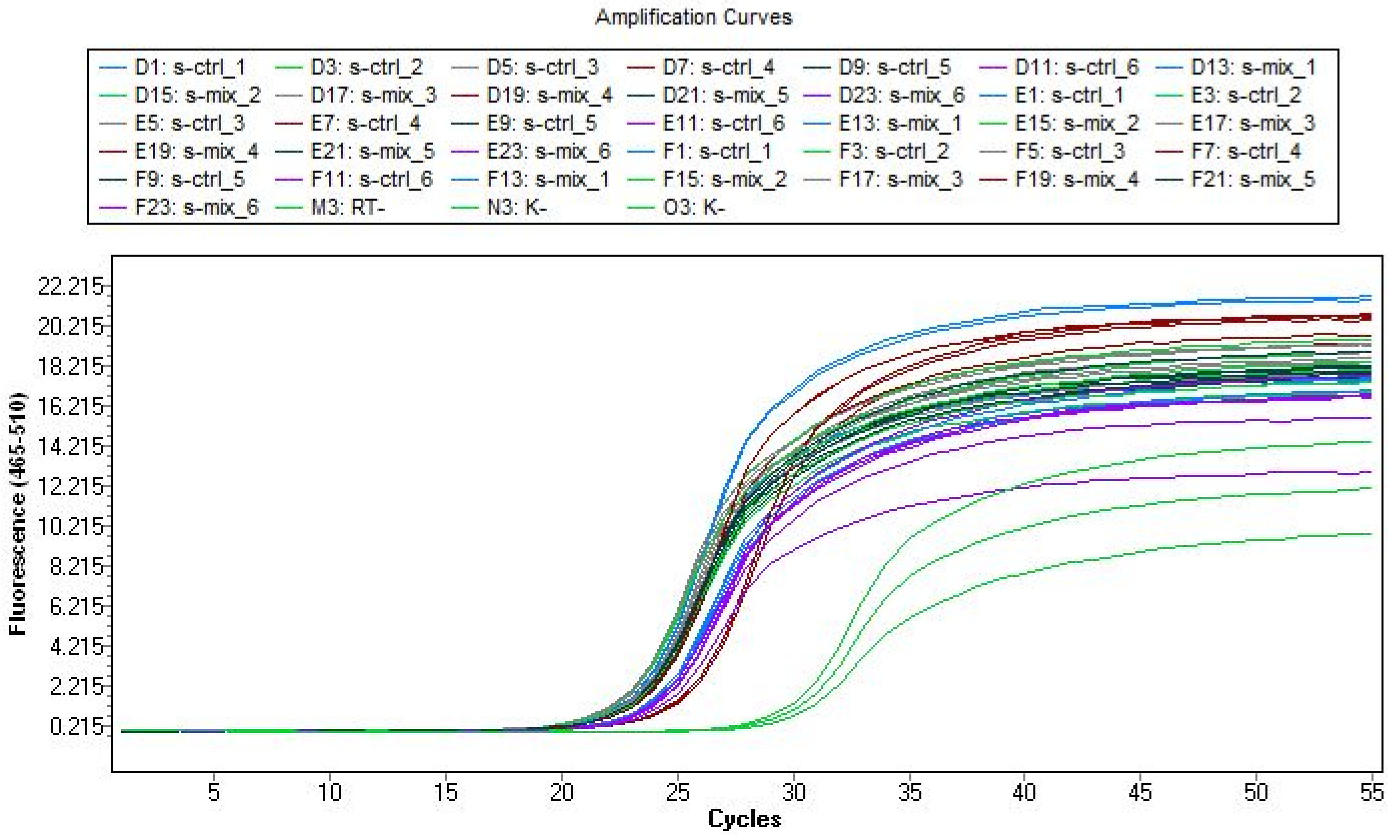

Abs Quant/2nd Derivative Max for S_Pendrin (Abs Quant/2nd Derivative Max)

Results

Inc	Pos	Name	Type	CP	Concentration	Standard	Status
☑	D2	s-ctrl_1	Unknown	26.26			
☑	D4	s-ctrl_2	Unknown	28.28			
☑	D6	s-ctrl_3	Unknown	25.11			
☑	D8	s-ctrl_4	Unknown	26.24			
☑	D10	s-ctrl_5	Unknown	26.73			
☑	D12	s-ctrl_6	Unknown	27.25			
☑	D14	s-mix_1	Unknown	29.83			
☑	D16	s-mix_2	Unknown	25.64			
☑	D18	s-mix_3	Unknown	29.53			
☑	D20	s-mix_4	Unknown	24.80			
☑	D22	s-mix_5	Unknown	26.08			
☑	D24	s-mix_6	Unknown	23.95			
☑	E2	s-ctrl_1	Unknown	26.52			
☑	E4	s-ctrl_2	Unknown	27.81			
☑	E6	s-ctrl_3	Unknown	25.19			
☑	E8	s-ctrl_4	Unknown	26.32			
☑	E10	s-ctrl_5	Unknown	26.92			

Results

Inc	Pos	Name	Type	CP	Concentration	Standard	Status
☑	E12	s-ctrl_6	Unknown	27.25			
☑	E14	s-mix_1	Unknown	29.86			
☑	E16	s-mix_2	Unknown	25.68			
☑	E18	s-mix_3	Unknown	29.36			
☑	E20	s-mix_4	Unknown	24.72			
☑	E22	s-mix_5	Unknown	26.18			
☑	E24	s-mix_6	Unknown	24.02			
☑	F2	s-ctrl_1	Unknown	26.31			
☑	F4	s-ctrl_2	Unknown	27.75			
☑	F6	s-ctrl_3	Unknown	25.11			
☑	F8	s-ctrl_4	Unknown	26.13			
☑	F10	s-ctrl_5	Unknown	26.84			
☑	F12	s-ctrl_6	Unknown	26.91			
☑	F14	s-mix_1	Unknown	29.50			
☑	F16	s-mix_2	Unknown	25.60			
☑	F18	s-mix_3	Unknown	29.03			
☑	F20	s-mix_4	Unknown	24.80			
☑	F22	s-mix_5	Unknown	26.20			
☑	F24	s-mix_6	Unknown	23.95			
☑	M4	RT-	Unknown	32.94			
☑	N4	K-	Unknown	32.84			

Amplification Curves

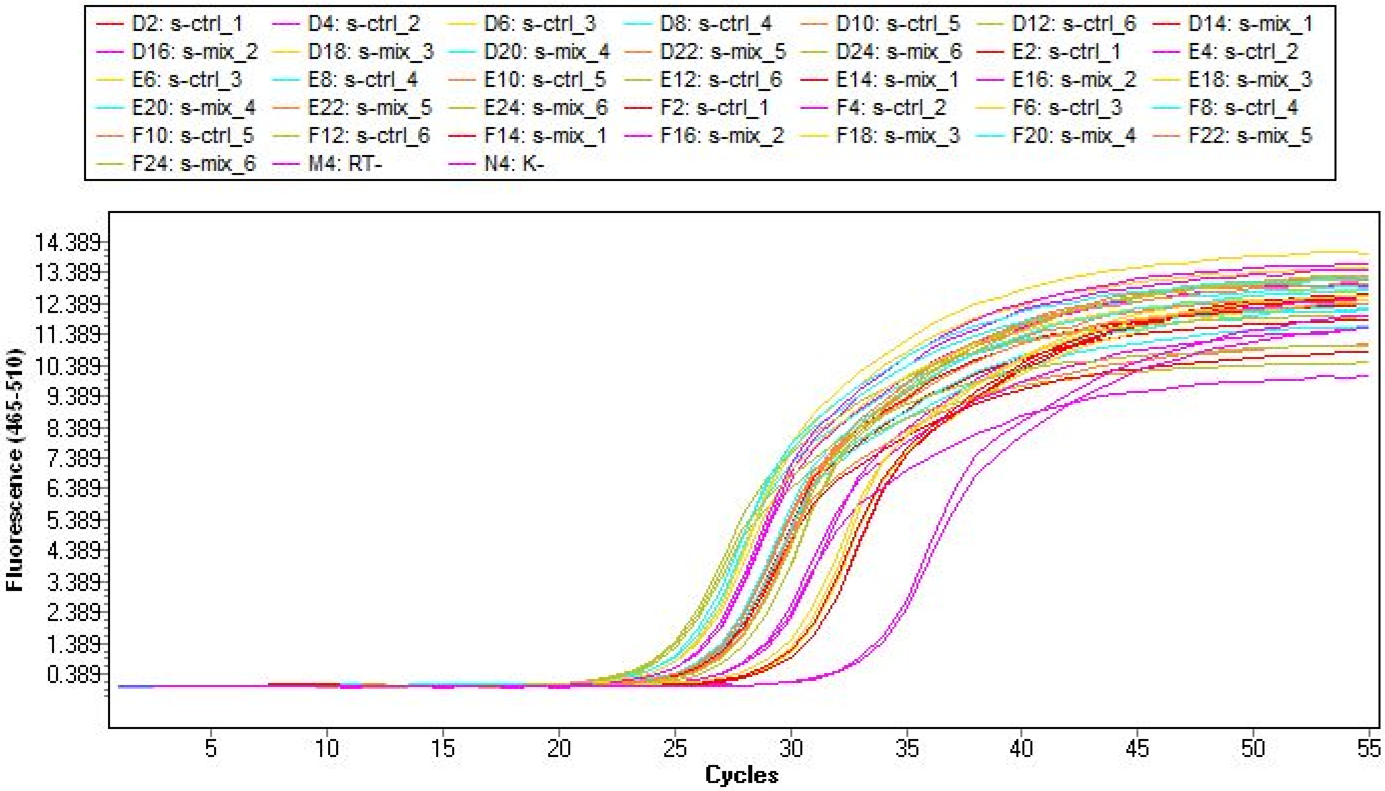

Abs Quant/2nd Derivative Max for A_HPRT (Abs Quant/2nd Derivative Max)

Results

Inc	Pos	Name	Type	CP	Concentration	Standard	Status
☑	G1	V5-AIT_1	Unknown	24.21			
☑	G3	V5-AIT_2	Unknown	24.30			
☑	G5	V5-AIT_3	Unknown	23.67			
☑	G7	V5-AIT_4	Unknown	25.02			
☑	G9	V5-AIT_5	Unknown	25.29			
☑	G11	V5-AIT_6	Unknown	25.18			
☑	G13	pcDNA3_1	Unknown	24.19			
☑	G15	pcDNA3_2	Unknown	24.71			
☑	G17	pcDNA3_3	Unknown	23.67			
☑	G19	pcDNA3_4	Unknown	24.31			
☑	G21	pcDNA3_5	Unknown	25.54			
☑	G23	pcDNA3_6	Unknown	27.05			
☑	H1	V5-AIT_1	Unknown	24.14			
☑	H3	V5-AIT_2	Unknown	24.29			
☑	H5	V5-AIT_3	Unknown	23.67			
☑	H7	V5-AIT_4	Unknown	24.97			
☑	H9	V5-AIT_5	Unknown	25.29			

Results

Inc	Pos	Name	Type	CP	Concentration	Standard	Status
☑	H11	V5-AIT_6	Unknown	25.01			
☑	H13	pcDNA3_1	Unknown	24.30			
☑	H15	pcDNA3_2	Unknown	24.69			
☑	H17	pcDNA3_3	Unknown	23.75			
☑	H19	pcDNA3_4	Unknown	24.34			
☑	H21	pcDNA3_5	Unknown	25.54			
☑	H23	pcDNA3_6	Unknown	27.17			
☑	I1	V5-AIT_1	Unknown	24.23			
☑	I3	V5-AIT_2	Unknown	24.29			
☑	I5	V5-AIT_3	Unknown	23.66			
☑	I7	V5-AIT_4	Unknown	25.09			
☑	I9	V5-AIT_5	Unknown	25.31			
☑	I11	V5-AIT_6	Unknown	25.12			
☑	I13	pcDNA3_1	Unknown	24.33			
☑	I15	pcDNA3_2	Unknown	24.70			
☑	I17	pcDNA3_3	Unknown	23.73			
☑	I19	pcDNA3_4	Unknown	24.33			
☑	I21	pcDNA3_5	Unknown	25.48			
☑	I23	pcDNA3_6	Unknown	27.08			
☑	M1	RT-	Unknown	32.70			
☑	N1	K-	Unknown	34.34			
☑	O1	K-	Unknown	32.60			

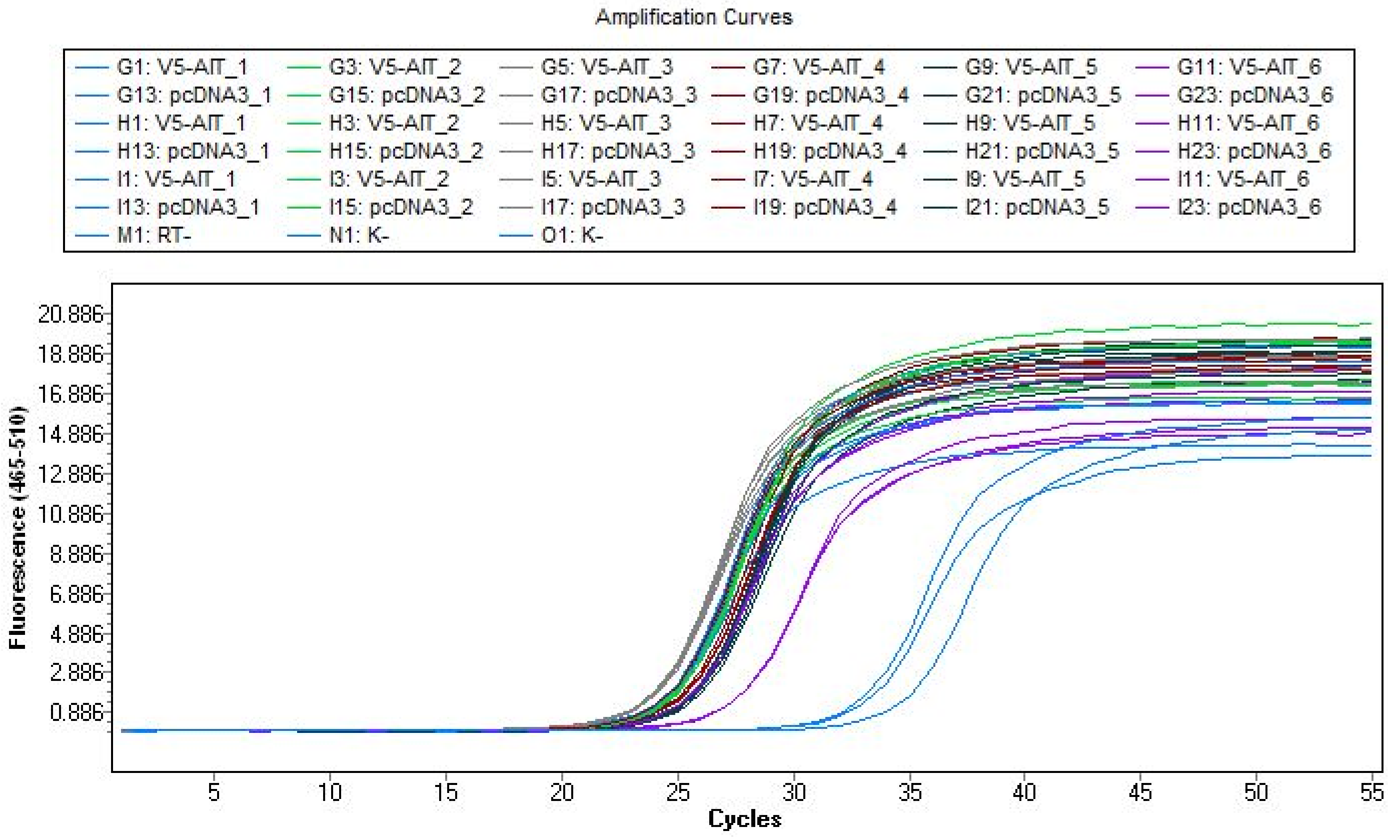

Abs Quant/2nd Derivative Max for A_AIT (Abs Quant/2nd Derivative Max)

Results

Inc	Pos	Name	Type	CP	Concentration	Standard	Status
☑	G2	V5-AIT_1	Unknown	14.29			
☑	G4	V5-AIT_2	Unknown	12.97			
☑	G6	V5-AIT_3	Unknown	14.09			
☑	G8	V5-AIT_4	Unknown	12.98			
☑	G10	V5-AIT_5	Unknown	13.12			
☑	G12	V5-AIT_6	Unknown	13.29			
☑	G14	pcDNA3_1	Unknown	20.59			
☑	G16	pcDNA3_2	Unknown	22.83			
☑	G18	pcDNA3_3	Unknown	21.12			
☑	G20	pcDNA3_4	Unknown	18.66			
☑	G22	pcDNA3_5	Unknown	21.24			
☑	G24	pcDNA3_6	Unknown	21.21			
☑	H2	V5-AIT_1	Unknown	14.34			
☑	H4	V5-AIT_2	Unknown	12.99			
☑	H6	V5-AIT_3	Unknown	14.17			
☑	H8	V5-AIT_4	Unknown	12.86			
☑	H10	V5-AIT_5	Unknown	13.16			

Results

Inc	Pos	Name	Type	CP	Concentration	Standard	Status
☑	H12	V5-AIT_6	Unknown	13.34			
☑	H14	pcDNA3_1	Unknown	20.77			
☑	H16	pcDNA3_2	Unknown	22.86			
☑	H18	pcDNA3_3	Unknown	21.17			
☑	H20	pcDNA3_4	Unknown	18.66			
☑	H22	pcDNA3_5	Unknown	21.36			
☑	H24	pcDNA3_6	Unknown	21.47			
☑	I2	V5-AIT_1	Unknown	14.32			
☑	I4	V5-AIT_2	Unknown	12.97			
☑	I6	V5-AIT_3	Unknown	14.18			
☑	I8	V5-AIT_4	Unknown	12.91			
☑	I10	V5-AIT_5	Unknown	13.13			
☑	I12	V5-AIT_6	Unknown	13.31			
☑	I14	pcDNA3_1	Unknown	20.74			
☑	I16	pcDNA3_2	Unknown	22.94			
☑	I18	pcDNA3_3	Unknown	21.42			
☑	I20	pcDNA3_4	Unknown	18.70			
☑	I22	pcDNA3_5	Unknown	21.32			
☑	I24	pcDNA3_6	Unknown	21.18			
☑	M2	RT-	Unknown				
☑	N2	K-	Unknown	46.23			
☑	O2	K-	Unknown				

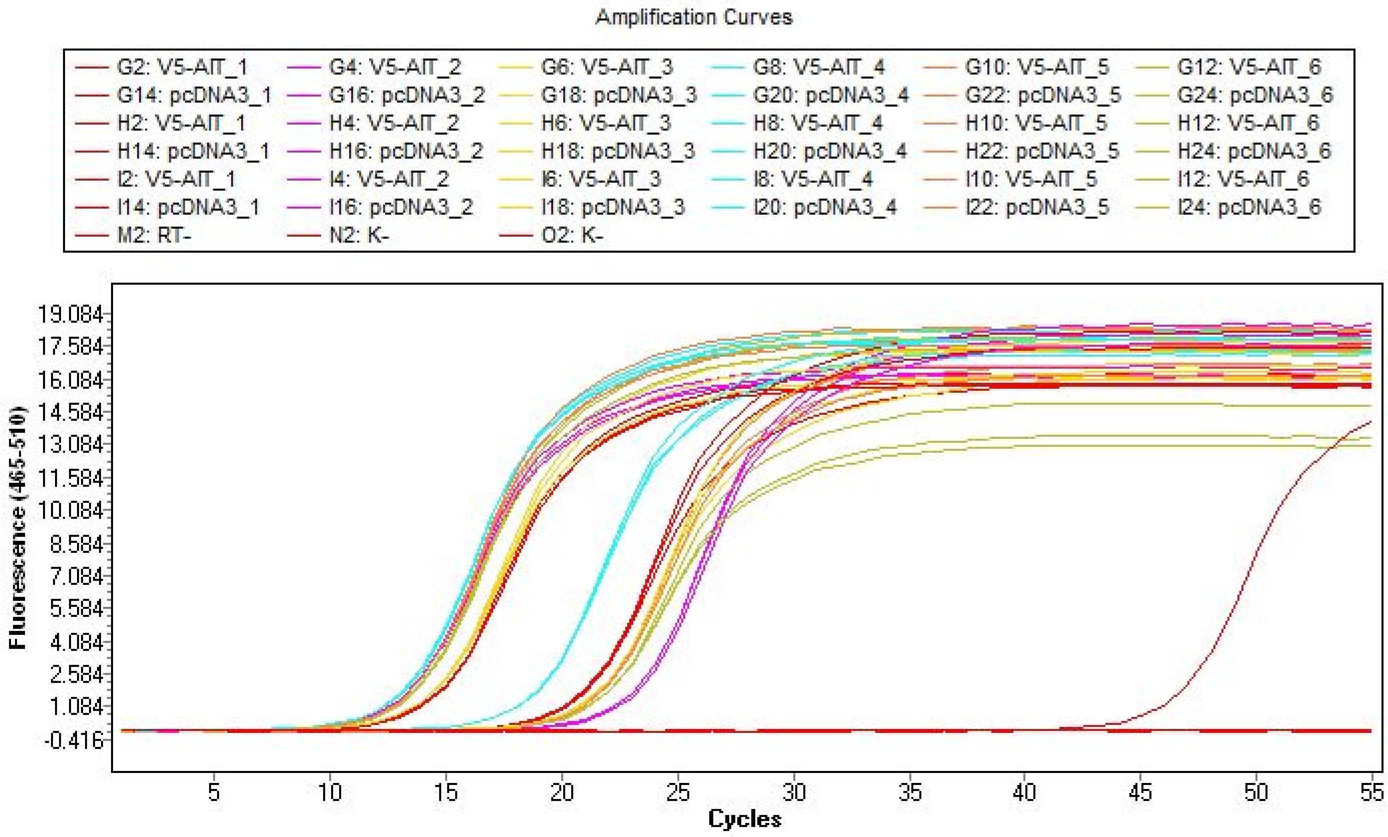

Abs Quant/2nd Derivative Max for A_BIRC5 (Abs Quant/2nd Derivative Max)

Results

Inc	Pos	Name	Type	CP	Concentration	Standard	Status
☑	J1	V5-AIT_1	Unknown	21.67			
☑	J3	V5-AIT_2	Unknown	22.82			
☑	J5	V5-AIT_3	Unknown	21.87			
☑	J7	V5-AIT_4	Unknown	21.83			
☑	J9	V5-AIT_5	Unknown	22.17			
☑	J11	V5-AIT_6	Unknown	21.99			
☑	J13	pcDNA3_1	Unknown	24.27			
☑	J15	pcDNA3_2	Unknown	22.01			
☑	J17	pcDNA3_3	Unknown	23.93			
☑	J19	pcDNA3_4	Unknown	24.89			
☑	J21	pcDNA3_5	Unknown	23.11			
☑	J23	pcDNA3_6	Unknown	24.19			
☑	K1	V5-AIT_1	Unknown	21.77			
☑	K3	V5-AIT_2	Unknown	22.61			
☑	K5	V5-AIT_3	Unknown	21.78			
☑	K7	V5-AIT_4	Unknown	21.85			
☑	K9	V5-AIT_5	Unknown	22.09			

Results

Inc	Pos	Name	Type	CP	Concentration	Standard	Status
☑	K11	V5-AIT_6	Unknown	22.21			
☑	K13	pcDNA3_1	Unknown	24.36			
☑	K15	pcDNA3_2	Unknown	22.04			
☑	K17	pcDNA3_3	Unknown	23.85			
☑	K19	pcDNA3_4	Unknown	24.84			
☑	K21	pcDNA3_5	Unknown	23.25			
☑	K23	pcDNA3_6	Unknown	24.22			
☑	L1	V5-AIT_1	Unknown	21.73			
☑	L3	V5-AIT_2	Unknown	22.62			
☑	L5	V5-AIT_3	Unknown	21.77			
☑	L7	V5-AIT_4	Unknown	21.79			
☑	L9	V5-AIT_5	Unknown	22.06			
☑	L11	V5-AIT_6	Unknown	22.14			
☑	L13	pcDNA3_1	Unknown	24.44			
☑	L15	pcDNA3_2	Unknown	22.07			
☑	L17	pcDNA3_3	Unknown	23.91			
☑	L19	pcDNA3_4	Unknown	24.89			
☑	L21	pcDNA3_5	Unknown	23.28			
☑	L23	pcDNA3_6	Unknown	24.21			
☑	M3	RT-	Unknown	29.62			
☑	N3	K-	Unknown	29.50			
☑	O3	K-	Unknown	29.68			

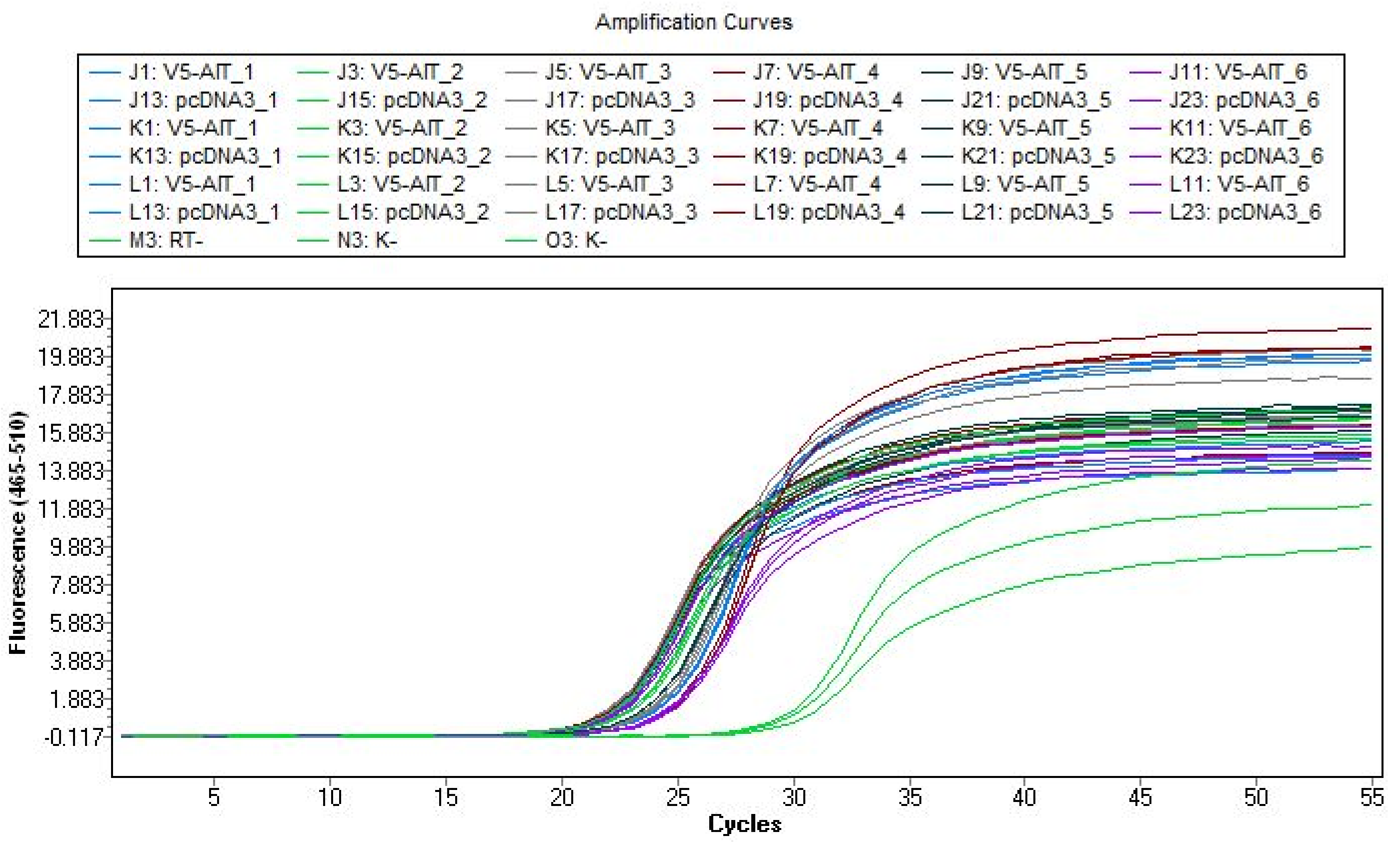

Abs Quant/2nd Derivative Max for A_Pendrin (Abs Quant/2nd Derivative Max)

Results

Inc	Pos	Name	Type	CP	Concentration	Standard	Status
☑	J2	V5-AIT_1	Unknown	26.47			
☑	J4	V5-AIT_2	Unknown	25.88			
☑	J6	V5-AIT_3	Unknown	25.04			
☑	J8	V5-AIT_4	Unknown	29.59			
☑	J10	V5-AIT_5	Unknown	27.09			
☑	J12	V5-AIT_6	Unknown	25.93			
☑	J14	pcDNA3_1	Unknown	24.78			
☑	J16	pcDNA3_2	Unknown	27.15			
☑	J18	pcDNA3_3	Unknown	25.47			
☑	J20	pcDNA3_4	Unknown	30.23			
☑	J22	pcDNA3_5	Unknown	26.29			
☑	J24	pcDNA3_6	Unknown	24.96			
☑	K2	V5-AIT_1	Unknown	26.51			
☑	K4	V5-AIT_2	Unknown	25.84			
☑	K6	V5-AIT_3	Unknown	25.14			
☑	K8	V5-AIT_4	Unknown	31.33			
☑	K10	V5-AIT_5	Unknown	27.06			

Results

Inc	Pos	Name	Type	CP	Concentration	Standard	Status
☑	K12	V5-AIT_6	Unknown	26.00			
☑	K14	pcDNA3_1	Unknown	24.76			
☑	K16	pcDNA3_2	Unknown	27.21			
☑	K18	pcDNA3_3	Unknown	25.67			
☑	K20	pcDNA3_4	Unknown	30.50			
☑	K22	pcDNA3_5	Unknown	26.33			
☑	K24	pcDNA3_6	Unknown	25.08			
☑	L2	V5-AIT_1	Unknown	26.54			
☑	L4	V5-AIT_2	Unknown	25.78			
☑	L6	V5-AIT_3	Unknown	25.02			
☑	L8	V5-AIT_4	Unknown	31.69			
☑	L10	V5-AIT_5	Unknown	26.23			
☑	L12	V5-AIT_6	Unknown	25.59			
☑	L14	pcDNA3_1	Unknown	24.62			
☑	L16	pcDNA3_2	Unknown	26.94			
☑	L18	pcDNA3_3	Unknown	25.62			
☑	L20	pcDNA3_4	Unknown	29.91			
☑	L22	pcDNA3_5	Unknown	25.95			
☑	L24	pcDNA3_6	Unknown	24.73			
☑	M4	RT-	Unknown	32.94			
☑	N4	K-	Unknown	32.84			

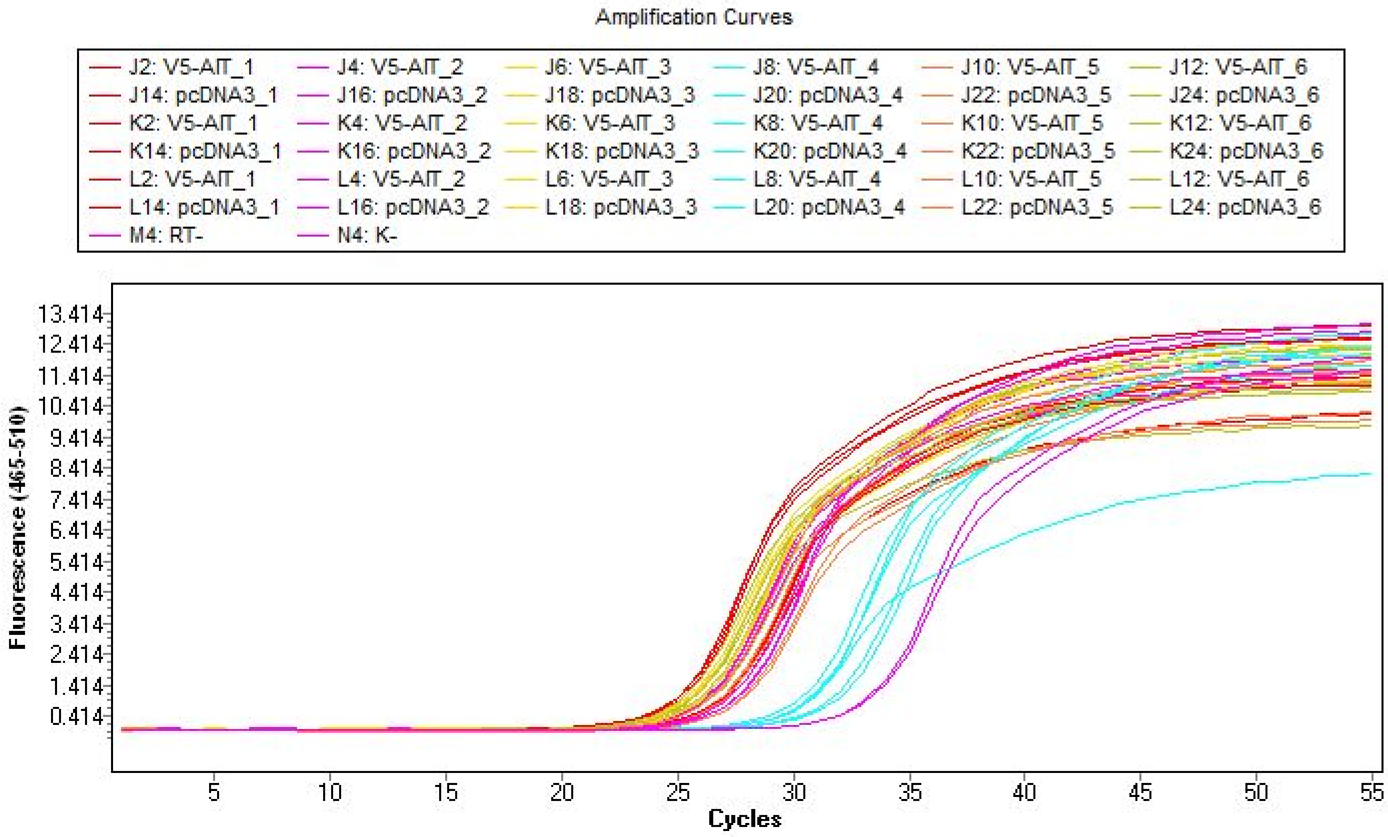
Amplification Curves
J2: V5-AIT_1
J4: V5-AIT_2
J6: V5-AIT_3
J8: V5-AIT_4
J10: V5-AIT_5
J12: V5-AIT_6
J14: pcDNA3_1
J16: pcDNA3_2
J18: pcDNA3_3
J20: pcDNA3_4
J22: pcDNA3_5
J24: pcDNA3_6
K2: V5-AIT_1
K4: V5-AIT_2
K6: V5-AIT_3
K8: V5-AIT_4
K10: V5-AIT_5
K12: V5-AIT_6
K14: pcDNA3_1
K16: pcDNA3_2
K18: pcDNA3_3
K20: pcDNA3_4
K22: pcDNA3_5
K24: pcDNA3_6
L2: V5-AIT_1
L4: V5-AIT_2
L6: V5-AIT_3
L8: V5-AIT_4
L10: V5-AIT_5
L12: V5-AIT_6
L14: pcDNA3_1
L16: pcDNA3_2
L18: pcDNA3_3
L20: pcDNA3_4
L22: pcDNA3_5
L24: pcDNA3_6
M4: RT-
N4: K-
Fluorescence (465-510)
13.414
12.414
11.414
10.414
9.414
8.414
7.414
6.414
5.414
4.414
3.414
2.414
1.414
0.414
5
10
15
20
25
30
35
40
45
50
55
Cycles
