# Supplementary material for: MicroRNAs and Their Inhibition in Modulating SLC5A8 Expression in the Context of Papillary Thyroid Carcinoma
Source: Int J Mol Sci. 2025 Aug 15;26(16):7889. doi: 10.3390/ijms26167889 (PMC12386254; doi:10.3390/ijms26167889)
Supplement: Supplementary file 1 [file ijms-26-07889-s001.zip › ijms-3558049-supplementary/Manuscript data/Fig6C data/Sponge-mix/Exp_3/2019-01-16 HEK293 po sponge-mix.pdf]

Roche

Wojtek_2019-01-16 HEK293 po sponge-mix

Programs

Program Name	pre-incubation		
Cycles	1	Analysis Mode	None

Target (°C)	Acquisition Mode	Hold (hh:mm:ss)	Ramp Rate (°C/s)	Acquisitions (per °C)	Sec Target (°C)	Step size (°C)	Step Delay (cycles)
95	None	00:10:00	4.80		0	0	0

Program Name	amplification		
Cycles	55	Analysis Mode	Quantification

Target (°C)	Acquisition Mode	Hold (hh:mm:ss)	Ramp Rate (°C/s)	Acquisitions (per °C)	Sec Target (°C)	Step size (°C)	Step Delay (cycles)
95	None	00:00:10	4.80		0	0	0
58	None	00:00:15	2.50		0	0	0
72	Single	00:00:10	4.80		0	0	0

Program Name	melting curve		
Cycles	1	Analysis Mode	Melting Curves

Target (°C)	Acquisition Mode	Hold (hh:mm:ss)	Ramp Rate (°C/s)	Acquisitions (per °C)	Sec Target (°C)	Step size (°C)	Step Delay (cycles)
95	None	00:00:05	4.80		0	0	0
65	None	00:01:00	2.50		0	0	0
97	Continuous		0.11	5	0	0	0

Program Name	cooling		
Cycles	1	Analysis Mode	None

Target (°C)	Acquisition Mode	Hold (hh:mm:ss)	Ramp Rate (°C/s)	Acquisitions (per °C)	Sec Target (°C)	Step size (°C)	Step Delay (cycles)
40	None	00:00:30	2.50		0	0	0

Tm Calling for HPRT (Tm Calling)

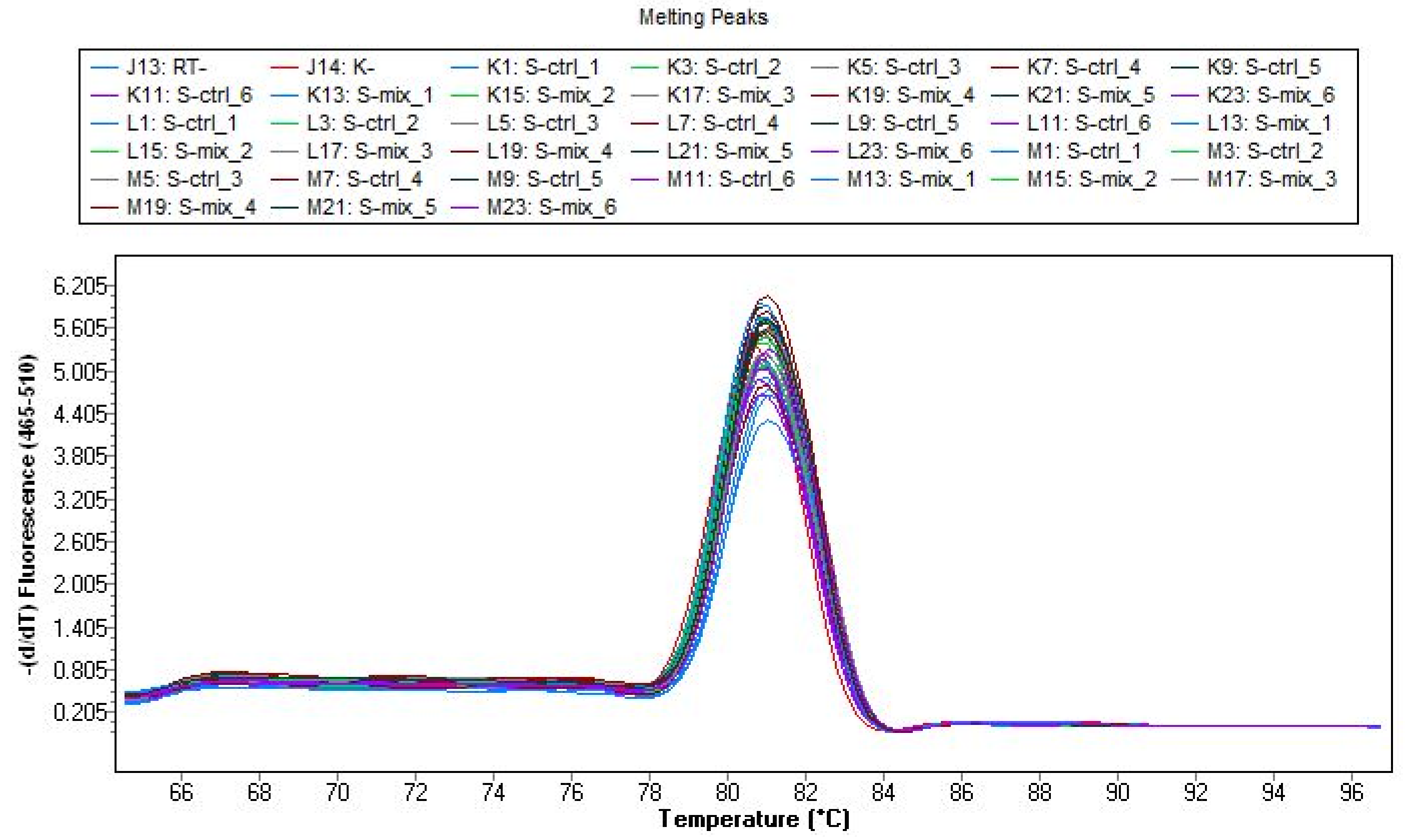

Tm Calling for AIT (Tm Calling)

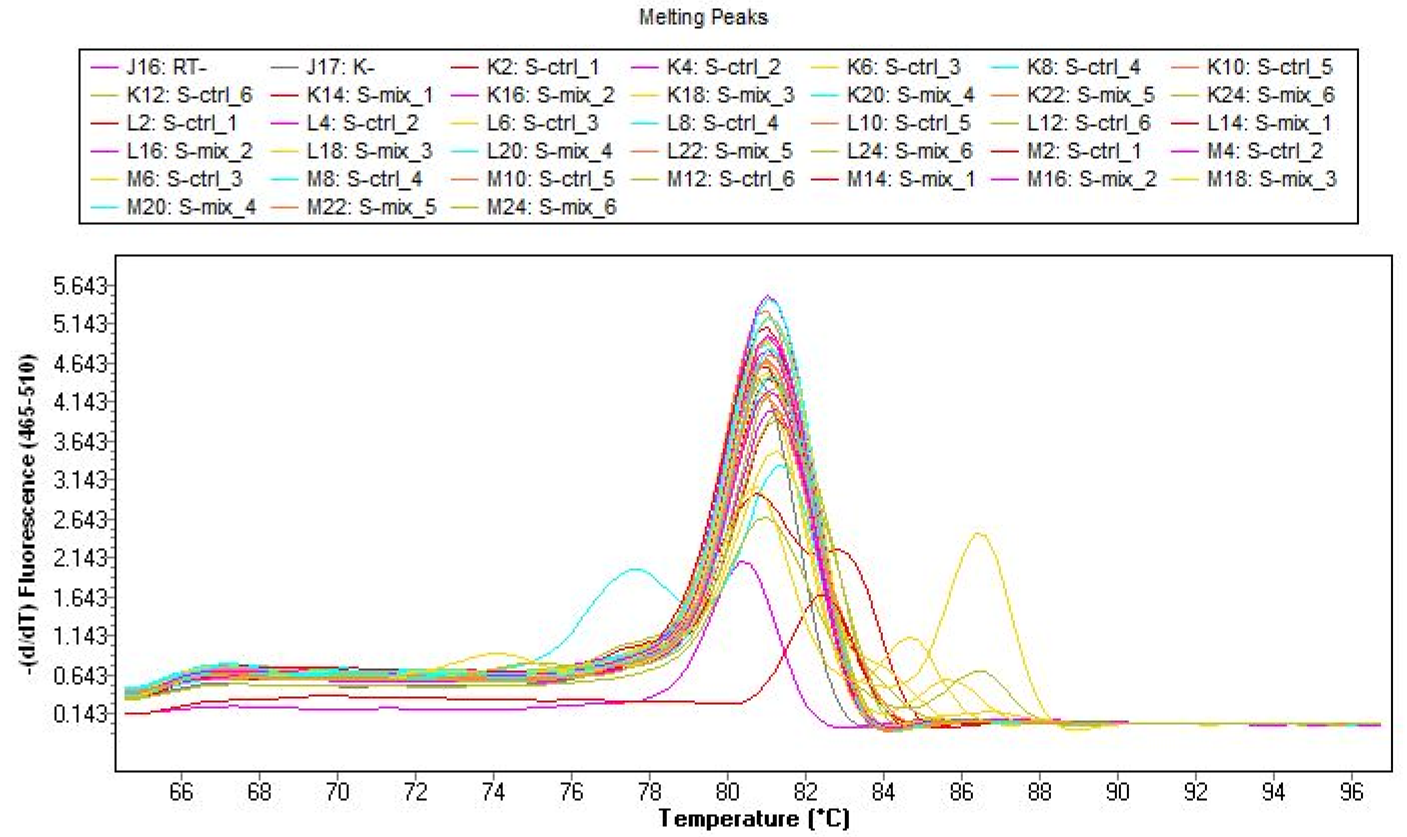

Tm Calling for BIRC5 (Tm Calling)

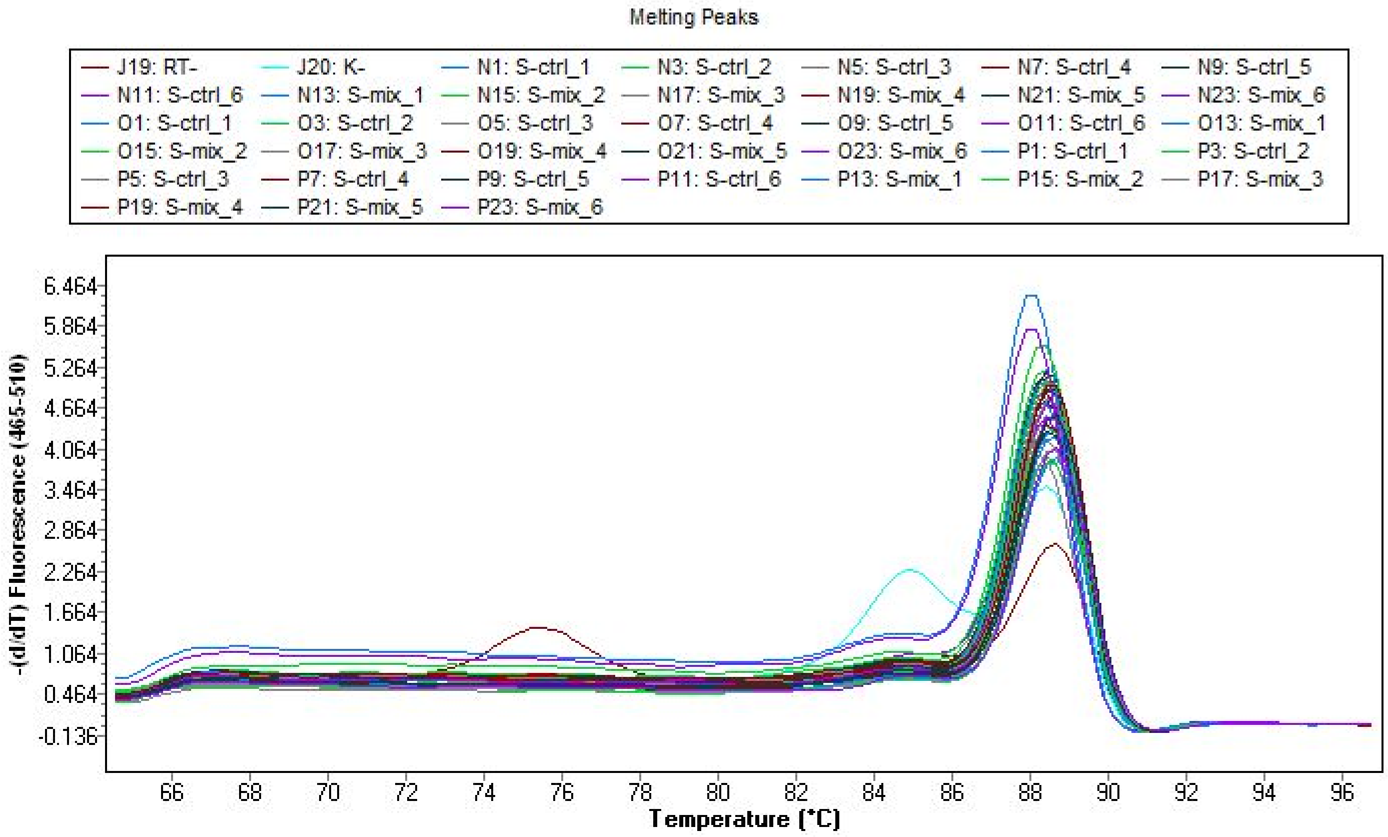

Abs Quant/2nd Derivative Max for HPRT (Abs Quant/2nd Derivative Max)

Results

Inc	Pos	Name	Type	CP	Concentration	Standard	Status
☑	J13	RT-	Negative Control	31.18			
☑	J14	K-	Negative Control	29.42			
☑	K1	S-ctrl_1	Unknown	22.71			
☑	K3	S-ctrl_2	Unknown	22.71			
☑	K5	S-ctrl_3	Unknown	23.70			
☑	K7	S-ctrl_4	Unknown	23.37			
☑	K9	S-ctrl_5	Unknown	23.85			
☑	K11	S-ctrl_6	Unknown	23.86			
☑	K13	S-mix_1	Unknown	23.53			
☑	K15	S-mix_2	Unknown	23.53			
☑	K17	S-mix_3	Unknown	23.56			
☑	K19	S-mix_4	Unknown	23.54			
☑	K21	S-mix_5	Unknown	23.35			
☑	K23	S-mix_6	Unknown	23.62			
☑	L1	S-ctrl_1	Unknown	22.77			

Results

Inc	Pos	Name	Type	CP	Concentration	Standard	Status
☑	L3	S-ctrl_2	Unknown	22.79			
☑	L5	S-ctrl_3	Unknown	23.61			
☑	L7	S-ctrl_4	Unknown	23.22			
☑	L9	S-ctrl_5	Unknown	23.67			
☑	L11	S-ctrl_6	Unknown	23.92			
☑	L13	S-mix_1	Unknown	23.35			
☑	L15	S-mix_2	Unknown	23.03			
☑	L17	S-mix_3	Unknown	23.50			
☑	L19	S-mix_4	Unknown	23.63			
☑	L21	S-mix_5	Unknown	23.25			
☑	L23	S-mix_6	Unknown	23.50			
☑	M1	S-ctrl_1	Unknown	21.96			
☑	M3	S-ctrl_2	Unknown	22.28			
☑	M5	S-ctrl_3	Unknown	23.15			
☑	M7	S-ctrl_4	Unknown	22.81			
☑	M9	S-ctrl_5	Unknown	23.45			
☑	M11	S-ctrl_6	Unknown	23.75			
☑	M13	S-mix_1	Unknown	22.96			
☑	M15	S-mix_2	Unknown	22.90			
☑	M17	S-mix_3	Unknown	23.24			
☑	M19	S-mix_4	Unknown	23.14			
☑	M21	S-mix_5	Unknown	23.04			
☑	M23	S-mix_6	Unknown	23.29			

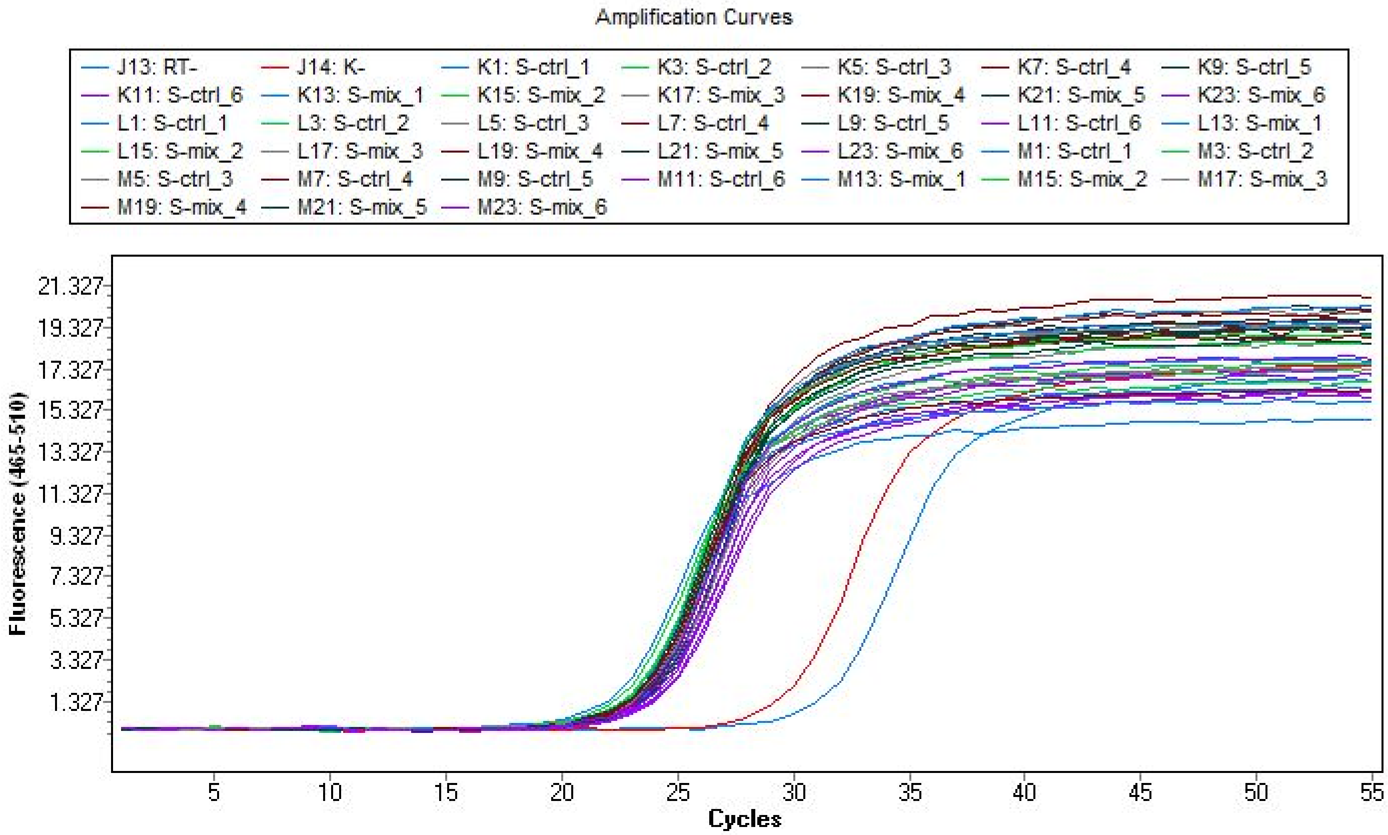

Abs Quant/2nd Derivative Max for AIT (Abs Quant/2nd Derivative Max)

Results

Inc	Pos	Name	Type	CP	Concentration	Standard	Status
☑	J16	RT-	Negative Control	37.26			
☑	J17	K-	Negative Control	38.04			
☑	K2	S-ctrl_1	Unknown	34.90			
☑	K4	S-ctrl_2	Unknown	31.64			
☑	K6	S-ctrl_3	Unknown	35.87			
☑	K8	S-ctrl_4	Unknown	35.55			
☑	K10	S-ctrl_5	Unknown	35.80			
☑	K12	S-ctrl_6	Unknown	36.78			
☑	K14	S-mix_1	Unknown	35.66			
☑	K16	S-mix_2	Unknown	34.96			
☑	K18	S-mix_3	Unknown	36.86			
☑	K20	S-mix_4	Unknown	35.64			
☑	K22	S-mix_5	Unknown	35.50			
☑	K24	S-mix_6	Unknown	35.33			
☑	L2	S-ctrl_1	Unknown	35.65			
☑	L4	S-ctrl_2	Unknown	33.41			

> - Late Cp call (last five cycles) has higher uncertainty

Results

Inc	Pos	Name	Type	CP	Concentration	Standard	Status
☑	L6	S-ctrl_3	Unknown	36.97			
☑	L8	S-ctrl_4	Unknown	36.23			
☑	L10	S-ctrl_5	Unknown	35.48			
☑	L12	S-ctrl_6	Unknown	36.31			
☑	L14	S-mix_1	Unknown	34.80			
☑	L16	S-mix_2	Unknown	36.55			
☑	L18	S-mix_3	Unknown	36.08			
☑	L20	S-mix_4	Unknown	33.58			
☑	L22	S-mix_5	Unknown	34.57			
☑	L24	S-mix_6	Unknown	35.21			
☑	M2	S-ctrl_1	Unknown	34.70			
☑	M4	S-ctrl_2	Unknown	32.00			
☑	M6	S-ctrl_3	Unknown	36.11			
☑	M8	S-ctrl_4	Unknown	35.56			
☑	M10	S-ctrl_5	Unknown	35.15			
☑	M12	S-ctrl_6	Unknown	38.16			
☑	M14	S-mix_1	Unknown	50.00			>
☑	M16	S-mix_2	Unknown	35.42			
☑	M18	S-mix_3	Unknown	36.07			
☑	M20	S-mix_4	Unknown	33.30			
☑	M22	S-mix_5	Unknown	34.11			
☑	M24	S-mix_6	Unknown	34.98			

> - Late Cp call (last five cycles) has higher uncertainty

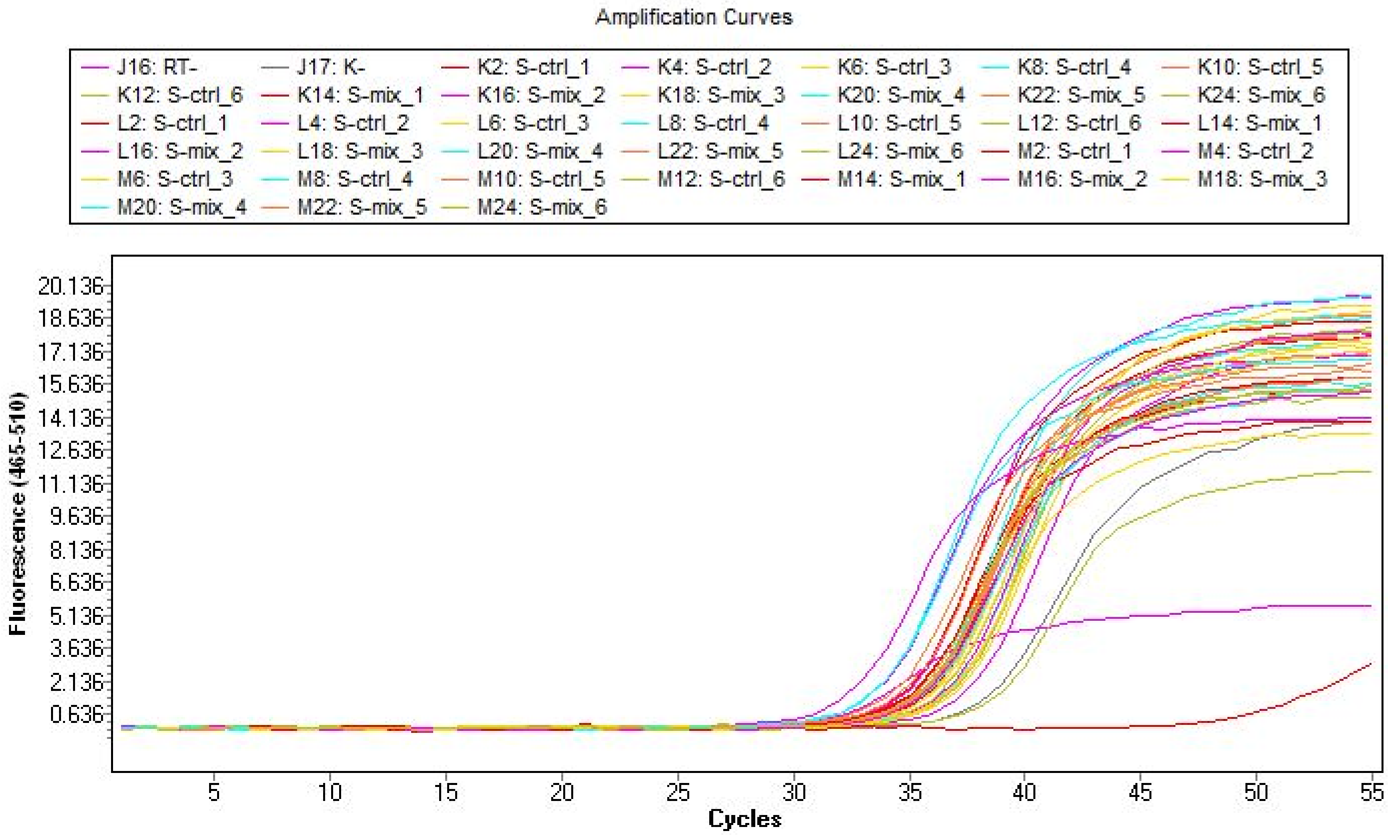

Abs Quant/2nd Derivative Max for BIRC5 (Abs Quant/2nd Derivative Max)

Results

Inc	Pos	Name	Type	CP	Concentration	Standard	Status
☑	J19	RT-	Negative Control	33.06			
☑	J20	K-	Negative Control	30.10			
☑	N1	S-ctrl_1	Unknown	22.84			
☑	N3	S-ctrl_2	Unknown	22.61			
☑	N5	S-ctrl_3	Unknown	23.98			
☑	N7	S-ctrl_4	Unknown	24.05			
☑	N9	S-ctrl_5	Unknown	25.03			
☑	N11	S-ctrl_6	Unknown	25.61			
☑	N13	S-mix_1	Unknown	25.10			
☑	N15	S-mix_2	Unknown	25.77			
☑	N17	S-mix_3	Unknown	24.59			
☑	N19	S-mix_4	Unknown	25.71			
☑	N21	S-mix_5	Unknown	24.82			
☑	N23	S-mix_6	Unknown	24.60			
☑	O1	S-ctrl_1	Unknown	23.13			
☑	O3	S-ctrl_2	Unknown	22.95			
☑	O5	S-ctrl_3	Unknown	23.76			

Results

Inc	Pos	Name	Type	CP	Concentration	Standard	Status
☑	O7	S-ctrl_4	Unknown	24.05			
☑	O9	S-ctrl_5	Unknown	23.56			
☑	O11	S-ctrl_6	Unknown	24.04			
☑	O13	S-mix_1	Unknown	23.91			
☑	O15	S-mix_2	Unknown	23.99			
☑	O17	S-mix_3	Unknown	23.26			
☑	O19	S-mix_4	Unknown	23.75			
☑	O21	S-mix_5	Unknown	23.49			
☑	O23	S-mix_6	Unknown	23.99			
☑	P1	S-ctrl_1	Unknown	21.82			
☑	P3	S-ctrl_2	Unknown	21.17			
☑	P5	S-ctrl_3	Unknown	22.23			
☑	P7	S-ctrl_4	Unknown	22.26			
☑	P9	S-ctrl_5	Unknown	22.09			
☑	P11	S-ctrl_6	Unknown	22.91			
☑	P13	S-mix_1	Unknown	22.52			
☑	P15	S-mix_2	Unknown	22.16			
☑	P17	S-mix_3	Unknown	22.24			
☑	P19	S-mix_4	Unknown	22.20			
☑	P21	S-mix_5	Unknown	21.96			
☑	P23	S-mix_6	Unknown	22.44			

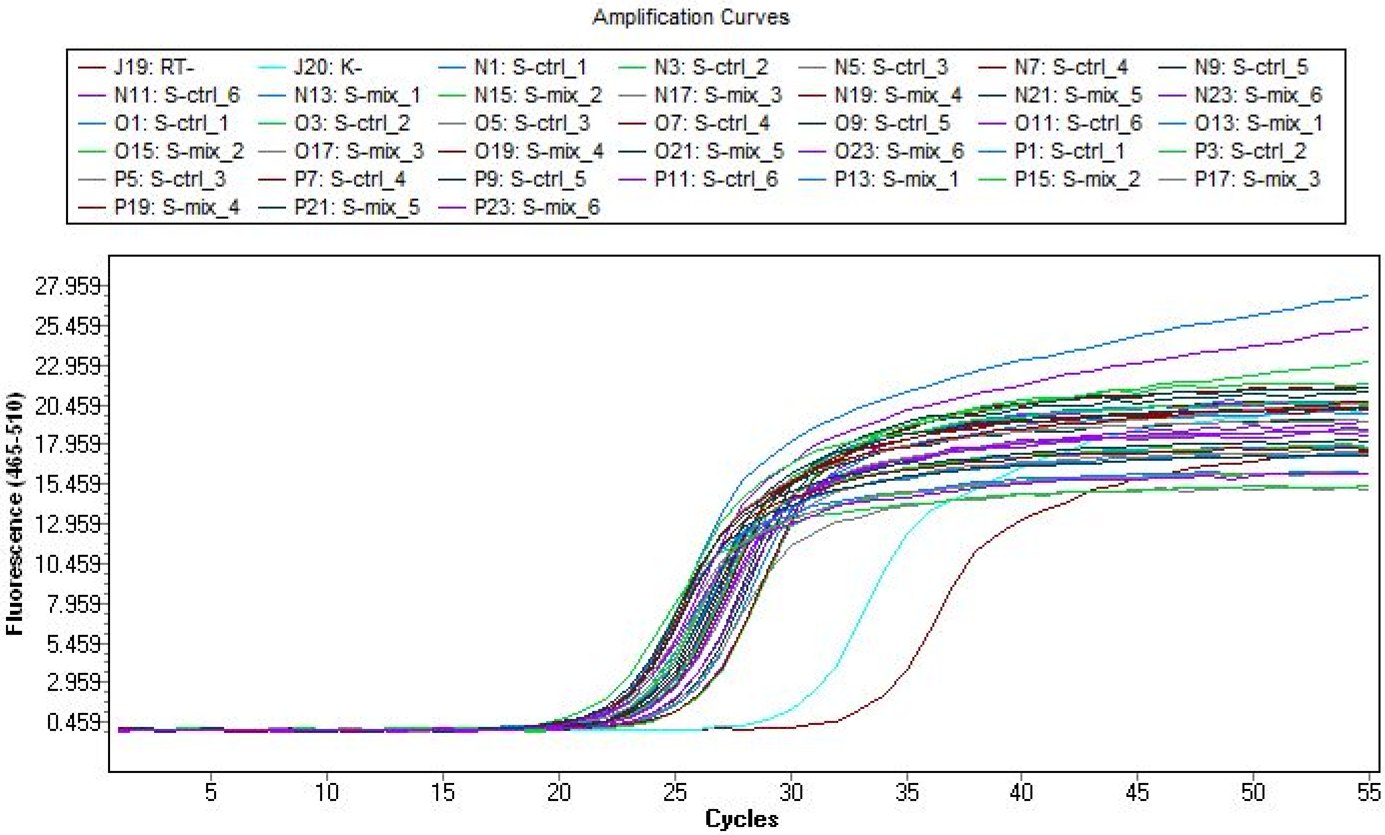
Amplification Curves
J19: RT-
J20: K-
N1: S-ctrl_1
N3: S-ctrl_2
N5: S-ctrl_3
N7: S-ctrl_4
N9: S-ctrl_5
N11: S-ctrl_6
N13: S-mix_1
N15: S-mix_2
N17: S-mix_3
N19: S-mix_4
N21: S-mix_5
N23: S-mix_6
O1: S-ctrl_1
O3: S-ctrl_2
O5: S-ctrl_3
O7: S-ctrl_4
O9: S-ctrl_5
O11: S-ctrl_6
O13: S-mix_1
O15: S-mix_2
O17: S-mix_3
O19: S-mix_4
O21: S-mix_5
O23: S-mix_6
P1: S-ctrl_1
P3: S-ctrl_2
P5: S-ctrl_3
P7: S-ctrl_4
P9: S-ctrl_5
P11: S-ctrl_6
P13: S-mix_1
P15: S-mix_2
P17: S-mix_3
P19: S-mix_4
P21: S-mix_5
P23: S-mix_6
27.959
25.459
22.959
20.459
17.959
15.459
12.959
10.459
7.959
5.459
2.959
0.459
Fluorescence (465-510)
5
10
15
20
25
30
35
40
45
50
55
Cycles
