# Supplementary material for: MicroRNAs and Their Inhibition in Modulating SLC5A8 Expression in the Context of Papillary Thyroid Carcinoma
Source: Int J Mol Sci. 2025 Aug 15;26(16):7889. doi: 10.3390/ijms26167889 (PMC12386254; doi:10.3390/ijms26167889)
Supplement: Supplementary file 1 [file ijms-26-07889-s001.zip › ijms-3558049-supplementary/Manuscript data/Fig6C data/Sponge-single/Exp_1/2017-10-31 AIT w HEK po transfekcji sponge 100ng.xlsx.PDF]

Wojtek_2017-10-31 AIT po transfekcji HEK sponge 100ng

Programs

Program Name	pre-incubation		
Cycles	1	Analysis Mode	None

Target (°C)	Acquisition Mode	Hold (hh:mm:ss)	Ramp Rate (°C/s)	Acquisitions (per °C)	Sec Target (°C)	Step size (°C)	Step Delay (cycles)
95	None	00:10:00	4.80		0	0	0

Program Name	amplification		
Cycles	55	Analysis Mode	Quantification

Target (°C)	Acquisition Mode	Hold (hh:mm:ss)	Ramp Rate (°C/s)	Acquisitions (per °C)	Sec Target (°C)	Step size (°C)	Step Delay (cycles)
95	None	00:00:10	4.80		0	0	0
58	None	00:00:15	2.50		0	0	0
72	Single	00:00:10	4.80		0	0	0

Program Name	melting curve		
Cycles	1	Analysis Mode	Melting Curves

Target (°C)	Acquisition Mode	Hold (hh:mm:ss)	Ramp Rate (°C/s)	Acquisitions (per °C)	Sec Target (°C)	Step size (°C)	Step Delay (cycles)
95	None	00:00:05	4.80		0	0	0
65	None	00:01:00	2.50		0	0	0
97	Continuous		0.11	5	0	0	0

Program Name	cooling		
Cycles	1	Analysis Mode	None

Target (°C)	Acquisition Mode	Hold (hh:mm:ss)	Ramp Rate (°C/s)	Acquisitions (per °C)	Sec Target (°C)	Step size (°C)	Step Delay (cycles)
40	None	00:00:30	2.50		0	0	0

Abs Quant/2nd Derivative Max for HPRT (Abs Quant/2nd Derivative Max)

Results

Inc	Pos	Name	Type	CP	Concentration	Standard	Status
☑	J1	S-ctrl_100ng_1	Unknown	20.96			
☑	J3	S-ctrl_100ng_2	Unknown	20.58			
☑	J5	S-ctrl_100ng_3	Unknown	20.48			
☑	J7	S-181_100ng_1	Unknown	20.70			
☑	J9	S-181_100ng_2	Unknown	20.53			

Results

Inc	Pos	Name	Type	CP	Concentration	Standard	Status
☑	J11	S-181_100ng_3	Unknown	20.44			
☑	J13	S-182_100ng_1	Unknown	20.71			
☑	J15	S-182_100ng_2	Unknown	20.94			
☑	J17	S-182_100ng_3	Unknown	20.80			
☑	J19	S-494_100ng_1	Unknown	20.88			
☑	J21	S-494_100ng_2	Unknown	20.82			
☑	J23	S-494_100ng_3	Unknown	20.81			
☑	K1	S-ctrl_100ng_1	Unknown	20.97			
☑	K3	S-ctrl_100ng_2	Unknown	20.64			
☑	K5	S-ctrl_100ng_3	Unknown	20.60			
☑	K7	S-181_100ng_1	Unknown	20.72			
☑	K9	S-181_100ng_2	Unknown	20.59			
☑	K11	S-181_100ng_3	Unknown	20.52			
☑	K13	S-182_100ng_1	Unknown	20.76			
☑	K15	S-182_100ng_2	Unknown	21.00			
☑	K17	S-182_100ng_3	Unknown	20.85			
☑	K19	S-494_100ng_1	Unknown	20.93			
☑	K21	S-494_100ng_2	Unknown	20.84			
☑	K23	S-494_100ng_3	Unknown	20.80			
☑	L1	S-ctrl_100ng_1	Unknown	20.93			
☑	L3	S-ctrl_100ng_2	Unknown	20.66			
☑	L5	S-ctrl_100ng_3	Unknown	20.49			
☑	L7	S-181_100ng_1	Unknown	20.76			
☑	L9	S-181_100ng_2	Unknown	20.64			
☑	L11	S-181_100ng_3	Unknown	20.57			
☑	L13	S-182_100ng_1	Unknown	20.84			
☑	L15	S-182_100ng_2	Unknown	21.04			
☑	L17	S-182_100ng_3	Unknown	20.90			
☑	L19	S-494_100ng_1	Unknown	21.00			
☑	L21	S-494_100ng_2	Unknown	20.87			
☑	L23	S-494_100ng_3	Unknown	20.85			
☑	M1	RT-	Negative Control				
☑	M2	K-	Negative Control	37.40			

Amplification Curves

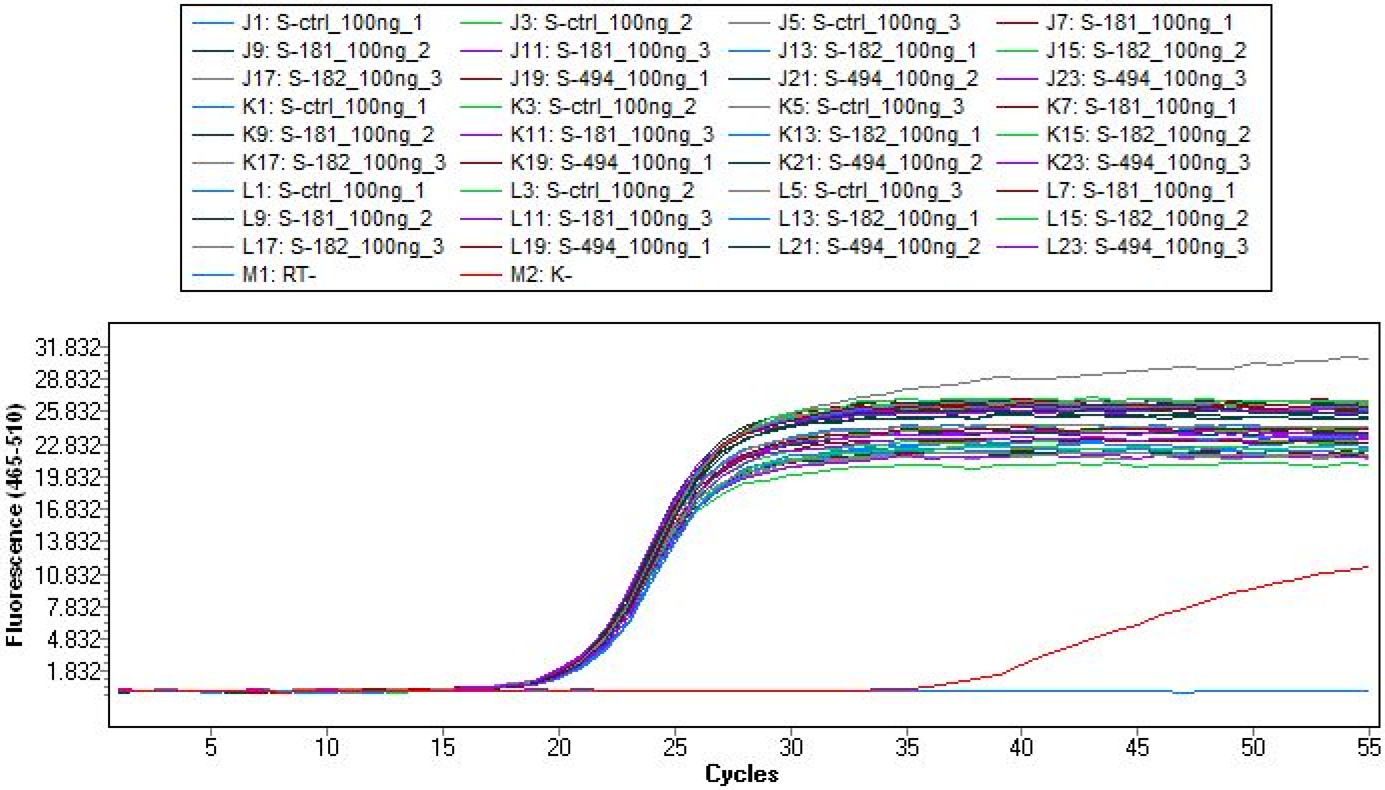

Abs Quant/2nd Derivative Max for AIT (Abs Quant/2nd Derivative Max)

Results

Inc	Pos	Name	Type	CP	Concentration	Standard	Status
☑	J2	S-ctrl_100ng_1	Unknown	36.79			
☑	J4	S-ctrl_100ng_2	Unknown	34.47			
☑	J6	S-ctrl_100ng_3	Unknown	34.91			
☑	J8	S-181_100ng_1	Unknown	35.12			
☑	J10	S-181_100ng_2	Unknown	35.63			
☑	J12	S-181_100ng_3	Unknown	35.55			
☑	J14	S-182_100ng_1	Unknown	34.59			
☑	J16	S-182_100ng_2	Unknown	35.62			
☑	J18	S-182_100ng_3	Unknown	36.09			
☑	J20	S-494_100ng_1	Unknown	35.99			
☑	J22	S-494_100ng_2	Unknown	35.46			
☑	J24	S-494_100ng_3	Unknown	35.47			
☑	K2	S-ctrl_100ng_1	Unknown	36.95			
☑	K4	S-ctrl_100ng_2	Unknown	35.59			
☑	K6	S-ctrl_100ng_3	Unknown	34.19			
☑	K8	S-181_100ng_1	Unknown	35.30			

Results

Inc	Pos	Name	Type	CP	Concentration	Standard	Status
☑	K10	S-181_100ng_2	Unknown	34.89			
☑	K12	S-181_100ng_3	Unknown	35.66			
☑	K14	S-182_100ng_1	Unknown	35.98			
☑	K16	S-182_100ng_2	Unknown	34.76			
☑	K18	S-182_100ng_3	Unknown	35.46			
☑	K20	S-494_100ng_1	Unknown	35.71			
☑	K22	S-494_100ng_2	Unknown	36.56			
☑	K24	S-494_100ng_3	Unknown	35.67			
☑	L2	S-ctrl_100ng_1	Unknown	35.26			
☑	L4	S-ctrl_100ng_2	Unknown	35.49			
☑	L6	S-ctrl_100ng_3	Unknown	35.22			
☑	L8	S-181_100ng_1	Unknown	34.46			
☑	L10	S-181_100ng_2	Unknown	35.43			
☑	L12	S-181_100ng_3	Unknown	35.47			
☑	L14	S-182_100ng_1	Unknown	34.69			
☑	L16	S-182_100ng_2	Unknown	35.27			
☑	L18	S-182_100ng_3	Unknown	35.66			
☑	L20	S-494_100ng_1	Unknown	37.00			
☑	L22	S-494_100ng_2	Unknown	35.18			
☑	L24	S-494_100ng_3	Unknown	35.54			
☑	M3	RT-	Negative Control				
☑	M4	K-	Negative Control				

Amplification Curves

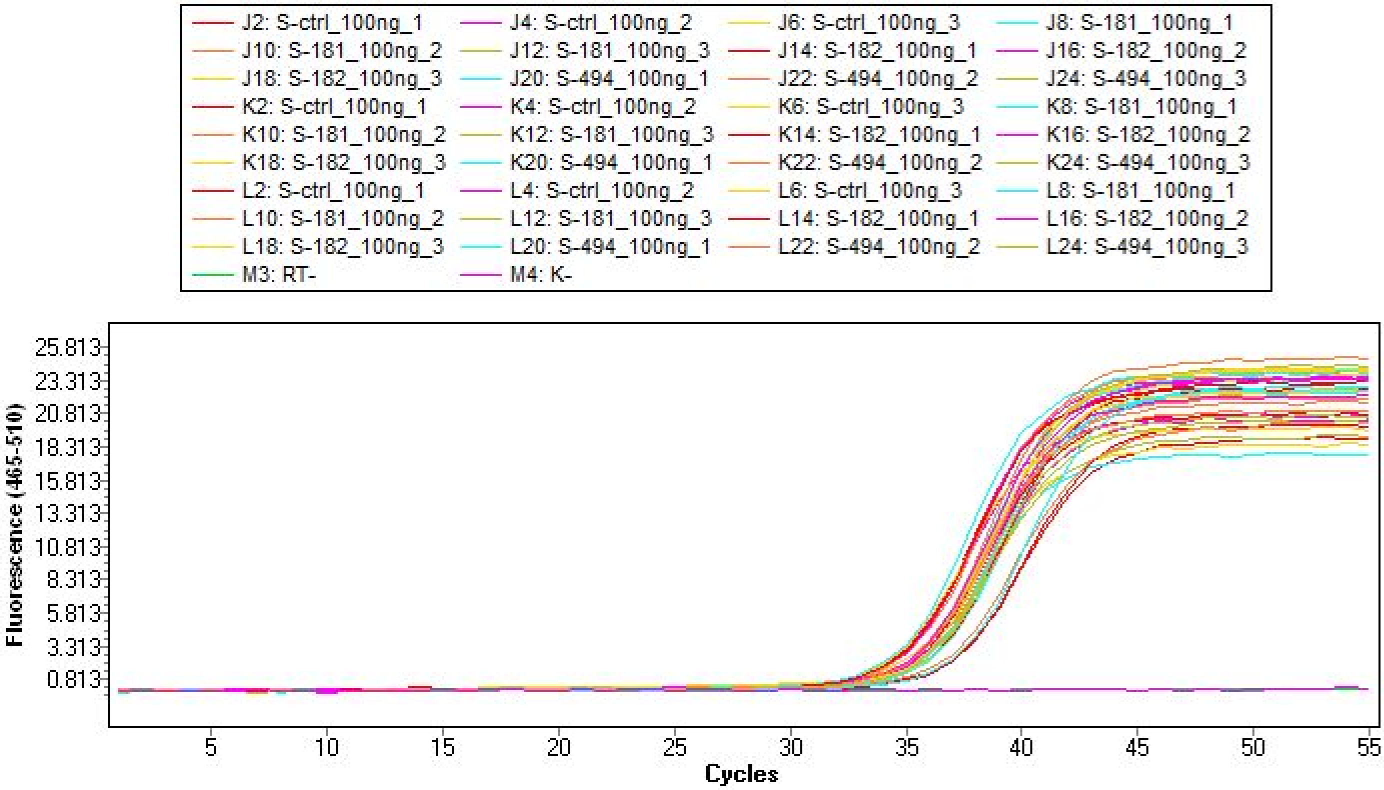

Tm Calling for HPRT (Tm Calling)

Results

Inc	Pos	Sample Name	Peak 1 Tm	Peak 1 Area	Peak 1 Width	Peak 1 Height	Peak 2 Tm	Peak 2 Area	Peak 2 Width	Peak 2 Height	Status
☑	J1	S-ctrl_100ng_1	80.65	19.98	2.62	7.64					
☑	J3	S-ctrl_100ng_2	80.71	17.77	2.65	6.70					
☑	J5	S-ctrl_100ng_3	80.76	18.27	2.61	7.01					
☑	J7	S-181_100ng_1	80.70	18.45	2.62	7.05					
☑	J9	S-181_100ng_2	80.69	18.59	2.62	7.09					
☑	J11	S-181_100ng_3	80.68	20.15	2.68	7.53					
☑	J13	S-182_100ng_1	80.69	20.40	2.66	7.67					
☑	J15	S-182_100ng_2	80.66	20.31	2.71	7.50					
☑	J17	S-182_100ng_3	80.80	20.46	2.67	7.67					
☑	J19	S-494_100ng_1	80.81	20.34	2.69	7.56					
☑	J21	S-494_100ng_2	80.83	19.96	2.70	7.40					
☑	J23	S-494_100ng_3	80.90	18.28	2.70	6.76					
☑	K1	S-ctrl_100ng_1	80.87	19.01	2.72	6.98					
☑	K3	S-ctrl_100ng_2	80.79	19.55	2.74	7.14					
☑	K5	S-ctrl_100ng_3	80.38	25.73	2.49	10.34					

Results

Inc	Pos	Sample Name	Peak 1 Tm	Peak 1 Area	Peak 1 Width	Peak 1 Height	Peak 2 Tm	Peak 2 Area	Peak 2 Width	Peak 2 Height	Status
☑	K7	S-181_100ng_1	80.79	19.00	2.74	6.93					
☑	K9	S-181_100ng_2	80.82	21.19	2.76	7.68					
☑	K11	S-181_100ng_3	80.73	21.81	2.76	7.90					
☑	K13	S-182_100ng_1	80.72	21.85	2.73	8.01					
☑	K15	S-182_100ng_2	80.70	22.03	2.75	8.02					
☑	K17	S-182_100ng_3	80.85	21.86	2.76	7.93					
☑	K19	S-494_100ng_1	80.83	22.38	2.76	8.12					
☑	K21	S-494_100ng_2	80.85	21.14	2.75	7.68					
☑	K23	S-494_100ng_3	80.95	19.63	2.74	7.16					
☑	L1	S-ctrl_100ng_1	80.92	18.49	2.79	6.64					
☑	L3	S-ctrl_100ng_2	80.80	18.85	2.74	6.89					
☑	L5	S-ctrl_100ng_3	80.83	18.31	2.73	6.71					
☑	L7	S-181_100ng_1	80.82	21.96	2.78	7.89					
☑	L9	S-181_100ng_2	80.86	22.40	2.81	7.98					
☑	L11	S-181_100ng_3	80.77	21.93	2.79	7.85					
☑	L13	S-182_100ng_1	80.74	22.20	2.78	8.00					
☑	L15	S-182_100ng_2	80.73	22.37	2.78	8.05					
☑	L17	S-182_100ng_3	80.85	22.49	2.79	8.07					
☑	L19	S-494_100ng_1	80.81	22.27	2.79	7.97					
☑	L21	S-494_100ng_2	80.86	21.59	2.78	7.76					
☑	L23	S-494_100ng_3	80.92	19.50	2.74	7.12					
☑	M1	RT-									
☑	M2	K-	79.60	10.49	2.36	4.44					

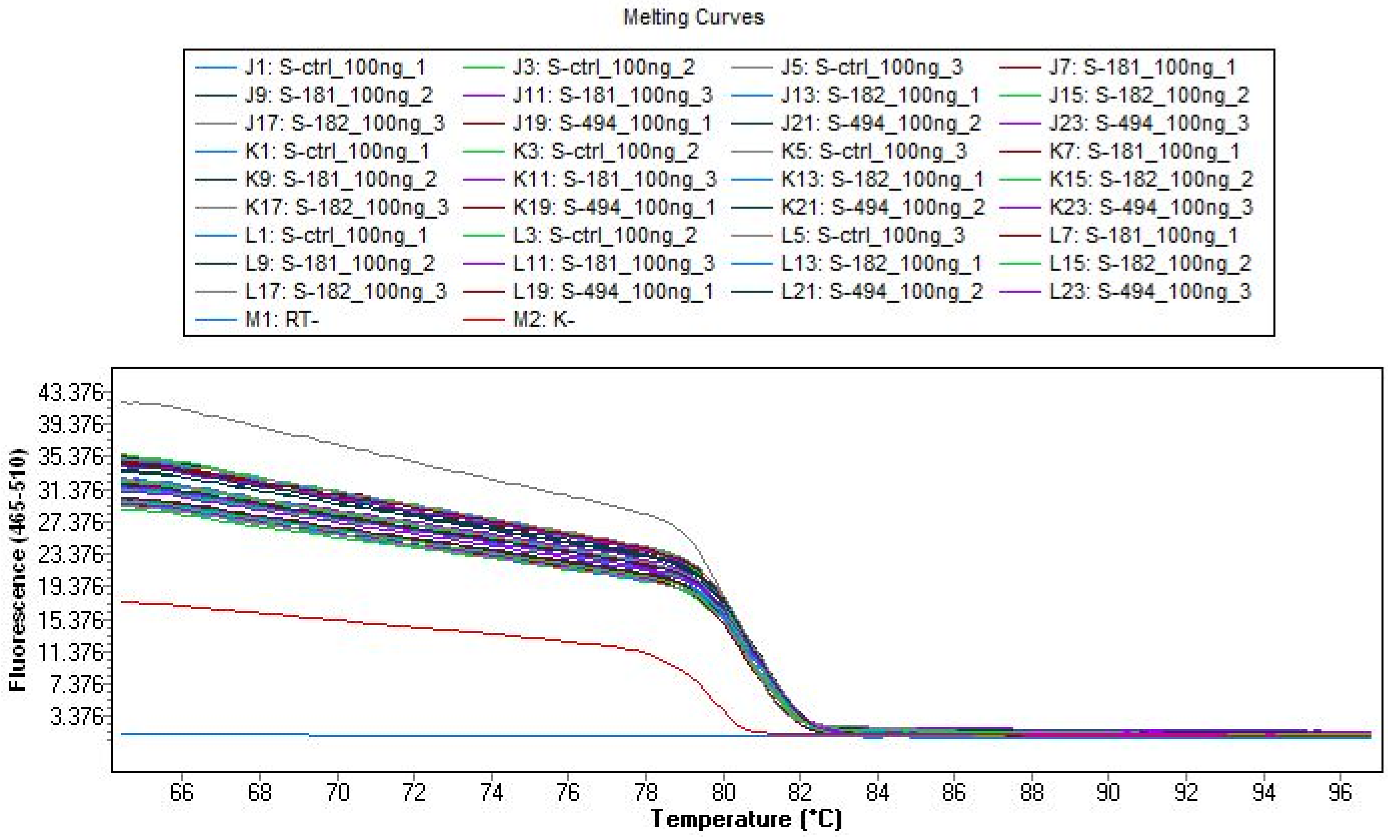

Tm Calling for AIT (Tm Calling)

Results

			Peak 1				Peak 2				
Inc	Pos	Sample Name	Tm	Area	Width	Height	Tm	Area	Width	Height	Status
☑	J2	S-ctrl_100ng_1	80.91	14.72	2.90	5.07					
☑	J4	S-ctrl_100ng_2	80.81	16.35	2.58	6.34					
☑	J6	S-ctrl_100ng_3	80.74	14.40	2.45	5.87					
☑	J8	S-181_100ng_1	80.78	14.07	2.55	5.51					
☑	J10	S-181_100ng_2	80.79	17.44	2.68	6.51					
☑	J12	S-181_100ng_3	80.73	17.79	2.66	6.69					
☑	J14	S-182_100ng_1	80.86	17.93	2.63	6.82					
☑	J16	S-182_100ng_2	80.90	18.82	2.63	7.16					
☑	J18	S-182_100ng_3	81.01	18.43	2.63	7.01					
☑	J20	S-494_100ng_1	80.95	17.85	2.82	6.34					
☑	J22	S-494_100ng_2	80.93	16.55	2.62	6.31					
☑	J24	S-494_100ng_3	81.01	14.95	2.58	5.80					
☑	K2	S-ctrl_100ng_1	81.17	16.16	3.10	5.21					
☑	K4	S-ctrl_100ng_2	80.85	15.99	2.65	6.04					
☑	K6	S-ctrl_100ng_3	80.83	15.59	2.69	5.79					

Results

Inc	Pos	Sample Name	Peak 1 Tm	Peak 1 Area	Peak 1 Width	Peak 1 Height	Peak 2 Tm	Peak 2 Area	Peak 2 Width	Peak 2 Height	Status
☑	K8	S-181_100ng_1	80.88	18.55	2.70	6.87					
☑	K10	S-181_100ng_2	80.84	19.10	2.80	6.82					
☑	K12	S-181_100ng_3	80.69	16.73	2.71	6.18					
☑	K14	S-182_100ng_1	80.84	18.35	2.62	7.01					
☑	K16	S-182_100ng_2	80.85	18.60	2.85	6.53					
☑	K18	S-182_100ng_3	80.99	18.93	2.62	7.23					
☑	K20	S-494_100ng_1	80.88	17.89	2.60	6.88					
☑	K22	S-494_100ng_2	80.96	15.99	2.95	5.42					
☑	K24	S-494_100ng_3	81.09	15.87	2.61	6.07					
☑	L2	S-ctrl_100ng_1	81.00	16.41	2.76	5.94					
☑	L4	S-ctrl_100ng_2	80.82	16.77	2.71	6.19					
☑	L6	S-ctrl_100ng_3	80.90	17.92	2.64	6.80					
☑	L8	S-181_100ng_1	80.93	19.03	2.69	7.08					
☑	L10	S-181_100ng_2	80.92	19.45	2.68	7.27					
☑	L12	S-181_100ng_3	80.83	19.31	2.64	7.32					
☑	L14	S-182_100ng_1	80.82	17.33	2.66	6.51					
☑	L16	S-182_100ng_2	80.90	18.55	2.69	6.89					
☑	L18	S-182_100ng_3	80.69	16.62	2.96	5.60					
☑	L20	S-494_100ng_1	80.91	16.65	2.77	6.00					
☑	L22	S-494_100ng_2	80.98	16.49	2.66	6.19					
☑	L24	S-494_100ng_3	81.11	16.23	2.63	6.18					
☑	M3	RT-									
☑	M4	K-									

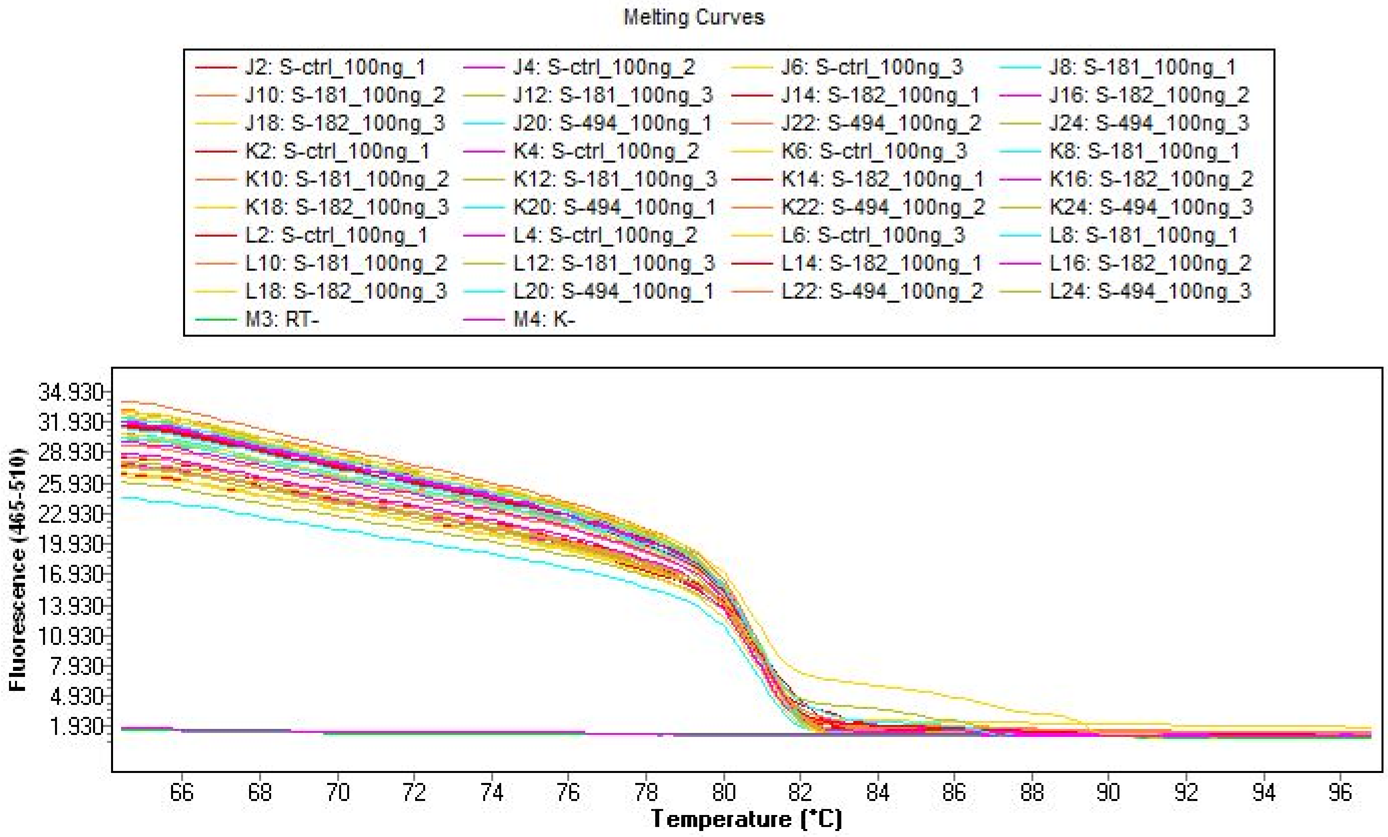
Melting Curves
J2: S-ctrl_100ng_1
J4: S-ctrl_100ng_2
J6: S-ctrl_100ng_3
J8: S-181_100ng_1
J10: S-181_100ng_2
J12: S-181_100ng_3
J14: S-182_100ng_1
J16: S-182_100ng_2
J18: S-182_100ng_3
J20: S-494_100ng_1
J22: S-494_100ng_2
J24: S-494_100ng_3
K2: S-ctrl_100ng_1
K4: S-ctrl_100ng_2
K6: S-ctrl_100ng_3
K8: S-181_100ng_1
K10: S-181_100ng_2
K12: S-181_100ng_3
K14: S-182_100ng_1
K16: S-182_100ng_2
K18: S-182_100ng_3
K20: S-494_100ng_1
K22: S-494_100ng_2
K24: S-494_100ng_3
L2: S-ctrl_100ng_1
L4: S-ctrl_100ng_2
L6: S-ctrl_100ng_3
L8: S-181_100ng_1
L10: S-181_100ng_2
L12: S-181_100ng_3
L14: S-182_100ng_1
L16: S-182_100ng_2
L18: S-182_100ng_3
L20: S-494_100ng_1
L22: S-494_100ng_2
L24: S-494_100ng_3
M3: RT-
M4: K-
Fluorescence (465-510)
34.930
31.930
28.930
25.930
22.930
19.930
16.930
13.930
10.930
7.930
4.930
1.930
66
68
70
72
74
76
78
80
82
84
86
88
90
92
94
96
Temperature (°C)
