# Supplementary material for: MicroRNAs and Their Inhibition in Modulating SLC5A8 Expression in the Context of Papillary Thyroid Carcinoma
Source: Int J Mol Sci. 2025 Aug 15;26(16):7889. doi: 10.3390/ijms26167889 (PMC12386254; doi:10.3390/ijms26167889)
Supplement: Supplementary file 1 [file ijms-26-07889-s001.zip › ijms-3558049-supplementary/Manuscript data/Fig6C data/Sponge-single/Exp_2/2017-12-06 AIT po transfekcji HEK sponge 100 i 50ng.PDF]

Roche

# Wojtek_2017-12-06 AIT po transfekcji HEK sponge 100 i 50ng

## Programs

| Program Name | pre-incubation | | |
|---|---|---|---|
| Cycles | 1 | Analysis Mode | None |

| Target (°C) | Acquisition Mode | Hold (hh:mm:ss) | Ramp Rate (°C/s) | Acquisitions (per °C) | Sec Target (°C) | Step size (°C) | Step Delay (cycles) |
|---|---|---|---|---|---|---|---|
| 95 | None | 00:10:00 | 4.80 | | 0 | 0 | 0 |

| Program Name | amplification | | |
|---|---|---|---|
| Cycles | 55 | Analysis Mode | Quantification |

| Target (°C) | Acquisition Mode | Hold (hh:mm:ss) | Ramp Rate (°C/s) | Acquisitions (per °C) | Sec Target (°C) | Step size (°C) | Step Delay (cycles) |
|---|---|---|---|---|---|---|---|
| 95 | None | 00:00:10 | 4.80 | | 0 | 0 | 0 |
| 58 | None | 00:00:15 | 2.50 | | 0 | 0 | 0 |
| 72 | Single | 00:00:10 | 4.80 | | 0 | 0 | 0 |

| Program Name | melting curve | | |
|---|---|---|---|
| Cycles | 1 | Analysis Mode | Melting Curves |

| Target (°C) | Acquisition Mode | Hold (hh:mm:ss) | Ramp Rate (°C/s) | Acquisitions (per °C) | Sec Target (°C) | Step size (°C) | Step Delay (cycles) |
|---|---|---|---|---|---|---|---|
| 95 | None | 00:00:05 | 4.80 | | 0 | 0 | 0 |
| 65 | None | 00:01:00 | 2.50 | | 0 | 0 | 0 |
| 97 | Continuous | | 0.11 | 5 | 0 | 0 | 0 |

| Program Name | cooling | | |
|---|---|---|---|
| Cycles | 1 | Analysis Mode | None |

| Target (°C) | Acquisition Mode | Hold (hh:mm:ss) | Ramp Rate (°C/s) | Acquisitions (per °C) | Sec Target (°C) | Step size (°C) | Step Delay (cycles) |
|---|---|---|---|---|---|---|---|
| 40 | None | 00:00:30 | 2.50 | | 0 | 0 | 0 |

# Abs Quant/2nd Derivative Max for HPRT (Abs Quant/2nd Derivative Max)

## Results

| Inc | Pos | Name | Type | CP | Concentration | Standard | Status |
|---|---|---|---|---|---|---|---|
| ☑ | J19 | RT- | Negative Control | 35.83 | | | |
| ☑ | J20 | K- | Negative Control | 35.27 | | | |
| ☑ | J21 | K- | Negative Control | | | | |
| ☑ | K1 | S-ctrl_100ng_1 | Unknown | 27.13 | | | |
| ☑ | K3 | S-ctrl_100ng_2 | Unknown | 24.44 | | | |

## Results

| Inc | Pos | Name | Type | CP | Concentration | Standard | Status |
|---|---|---|---|---|---|---|---|
| ☑ | K5 | S-ctrl_100ng_3 | Unknown | 24.76 | | | |
| ☑ | K7 | S-181_100ng_1 | Unknown | 25.60 | | | |
| ☑ | K9 | S-181_100ng_2 | Unknown | 25.23 | | | |
| ☑ | K11 | S-181_100ng_3 | Unknown | 25.86 | | | |
| ☑ | K13 | S-182_100ng_1 | Unknown | 24.11 | | | |
| ☑ | K15 | S-182_100ng_2 | Unknown | 24.24 | | | |
| ☑ | K17 | S-182_100ng_3 | Unknown | 24.67 | | | |
| ☑ | K19 | S-494_100ng_1 | Unknown | 24.60 | | | |
| ☑ | K21 | S-494_100ng_2 | Unknown | 25.69 | | | |
| ☑ | K23 | S-494_100ng_3 | Unknown | 25.02 | | | |
| ☑ | L1 | S-ctrl_100ng_1 | Unknown | 27.09 | | | |
| ☑ | L3 | S-ctrl_100ng_2 | Unknown | 24.85 | | | |
| ☑ | L5 | S-ctrl_100ng_3 | Unknown | 23.71 | | | |
| ☑ | L7 | S-181_100ng_1 | Unknown | 25.13 | | | |
| ☑ | L9 | S-181_100ng_2 | Unknown | 24.70 | | | |
| ☑ | L11 | S-181_100ng_3 | Unknown | 24.57 | | | |
| ☑ | L13 | S-182_100ng_1 | Unknown | 24.07 | | | |
| ☑ | L15 | S-182_100ng_2 | Unknown | 23.89 | | | |
| ☑ | L17 | S-182_100ng_3 | Unknown | 22.80 | | | |
| ☑ | L19 | S-494_100ng_1 | Unknown | 24.01 | | | |
| ☑ | L21 | S-494_100ng_2 | Unknown | 25.21 | | | |
| ☑ | L23 | S-494_100ng_3 | Unknown | 23.25 | | | |
| ☑ | M1 | S-ctrl_100ng_1 | Unknown | 24.15 | | | |
| ☑ | M3 | S-ctrl_100ng_2 | Unknown | 20.42 | | | |
| ☑ | M5 | S-ctrl_100ng_3 | Unknown | 20.10 | | | |
| ☑ | M7 | S-181_100ng_1 | Unknown | 21.06 | | | |
| ☑ | M9 | S-181_100ng_2 | Unknown | 20.43 | | | |
| ☑ | M11 | S-181_100ng_3 | Unknown | 21.03 | | | |
| ☑ | M13 | S-182_100ng_1 | Unknown | 20.47 | | | |
| ☑ | M15 | S-182_100ng_2 | Unknown | 19.98 | | | |
| ☑ | M17 | S-182_100ng_3 | Unknown | 20.78 | | | |
| ☑ | M19 | S-494_100ng_1 | Unknown | 21.08 | | | |
| ☑ | M21 | S-494_100ng_2 | Unknown | 21.77 | | | |
| ☑ | M23 | S-494_100ng_3 | Unknown | 20.68 | | | |
| ☑ | N1 | S-ctrl_50ng_1 | Unknown | 25.98 | | | |
| ☑ | N3 | S-ctrl_50ng_2 | Unknown | 24.81 | | | |

**Results**

| Inc | Pos | Name | Type | CP | Concentration | Standard | Status |
|---|---|---|---|---|---|---|---|
| ☑ | N5 | S-ctrl_50ng_3 | Unknown | 24.94 | | | |
| ☑ | N7 | S-181_50ng_1 | Unknown | 24.72 | | | |
| ☑ | N9 | S-181_50ng_2 | Unknown | 24.68 | | | |
| ☑ | N11 | S-181_50ng_3 | Unknown | 25.10 | | | |
| ☑ | N13 | S-182_50ng_1 | Unknown | 25.85 | | | |
| ☑ | N15 | S-182_50ng_2 | Unknown | 24.82 | | | |
| ☑ | N17 | S-182_50ng_3 | Unknown | 24.25 | | | |
| ☑ | N19 | S-494_50ng_1 | Unknown | 24.42 | | | |
| ☑ | N21 | S-494_50ng_2 | Unknown | 24.67 | | | |
| ☑ | N23 | S-494_50ng_3 | Unknown | 26.20 | | | |
| ☑ | O1 | S-ctrl_50ng_1 | Unknown | 25.90 | | | |
| ☑ | O3 | S-ctrl_50ng_2 | Unknown | 24.83 | | | |
| ☑ | O5 | S-ctrl_50ng_3 | Unknown | 23.65 | | | |
| ☑ | O7 | S-181_50ng_1 | Unknown | 25.46 | | | |
| ☑ | O9 | S-181_50ng_2 | Unknown | 24.96 | | | |
| ☑ | O11 | S-181_50ng_3 | Unknown | 21.62 | | | |
| ☑ | O13 | S-182_50ng_1 | Unknown | 24.91 | | | |
| ☑ | O15 | S-182_50ng_2 | Unknown | 24.46 | | | |
| ☑ | O17 | S-182_50ng_3 | Unknown | 22.51 | | | |
| ☑ | O19 | S-494_50ng_1 | Unknown | 23.90 | | | |
| ☑ | O21 | S-494_50ng_2 | Unknown | 24.33 | | | |
| ☑ | O23 | S-494_50ng_3 | Unknown | 25.45 | | | |
| ☑ | P1 | S-ctrl_50ng_1 | Unknown | 23.48 | | | |
| ☑ | P3 | S-ctrl_50ng_2 | Unknown | 21.02 | | | |
| ☑ | P5 | S-ctrl_50ng_3 | Unknown | 21.87 | | | |
| ☑ | P7 | S-181_50ng_1 | Unknown | 20.99 | | | |
| ☑ | P9 | S-181_50ng_2 | Unknown | 20.18 | | | |
| ☑ | P11 | S-181_50ng_3 | Unknown | 20.90 | | | |
| ☑ | P13 | S-182_50ng_1 | Unknown | 21.70 | | | |
| ☑ | P15 | S-182_50ng_2 | Unknown | 20.14 | | | |
| ☑ | P17 | S-182_50ng_3 | Unknown | 20.15 | | | |
| ☑ | P19 | S-494_50ng_1 | Unknown | 21.02 | | | |
| ☑ | P21 | S-494_50ng_2 | Unknown | 21.26 | | | |
| ☑ | P23 | S-494_50ng_3 | Unknown | 22.88 | | | |

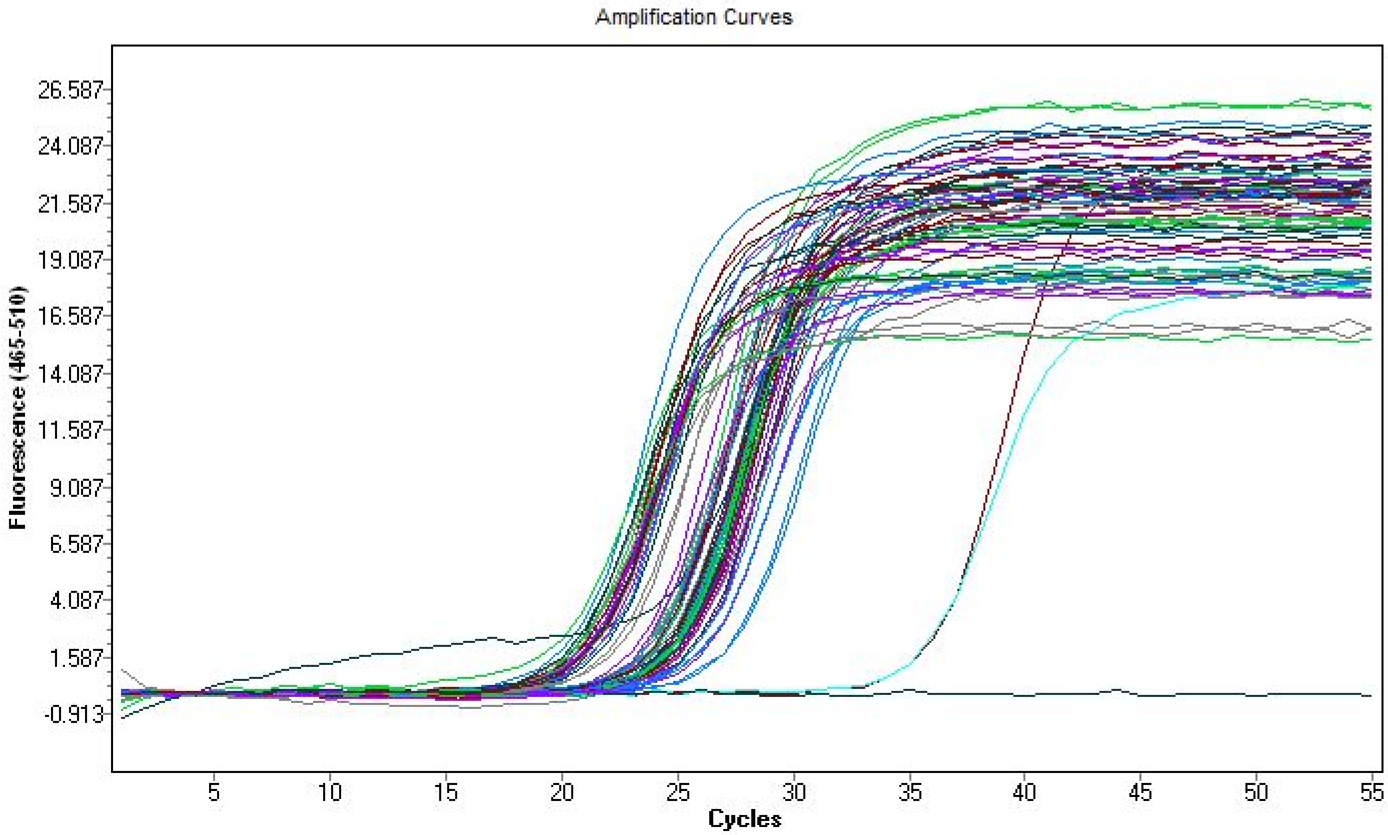


## Abs Quant/2nd Derivative Max for AIT (Abs Quant/2nd Derivative Max)

**Results**

| Inc | Pos | Name | Type | CP | Concentration | Standard | Status |
|---|---|---|---|---|---|---|---|
| ☑ | J22 | RT- | Negative Control | 40.46 | | | |
| ☑ | J23 | K- | Negative Control | | | | |
| ☑ | J24 | K- | Negative Control | 36.07 | | | |
| ☑ | K2 | S-ctrl_100ng_1 | Unknown | 37.13 | | | |
| ☑ | K4 | S-ctrl_100ng_2 | Unknown | 35.05 | | | |
| ☑ | K6 | S-ctrl_100ng_3 | Unknown | 35.08 | | | |
| ☑ | K8 | S-181_100ng_1 | Unknown | 34.03 | | | |
| ☑ | K10 | S-181_100ng_2 | Unknown | 26.83 | | | |
| ☑ | K12 | S-181_100ng_3 | Unknown | 27.92 | | | |
| ☑ | K14 | S-182_100ng_1 | Unknown | 30.43 | | | |
| ☑ | K16 | S-182_100ng_2 | Unknown | 31.23 | | | |
| ☑ | K18 | S-182_100ng_3 | Unknown | 30.75 | | | |
| ☑ | K20 | S-494_100ng_1 | Unknown | 33.23 | | | |
| ☑ | K22 | S-494_100ng_2 | Unknown | 27.59 | | | |
| ☑ | K24 | S-494_100ng_3 | Unknown | 31.49 | | | |
| ☑ | L2 | S-ctrl_100ng_1 | Unknown | 36.88 | | | |

**Results**

| Inc | Pos | Name | Type | CP | Concentration | Standard | Status |
|---|---|---|---|---|---|---|---|
| ☑ | L4 | S-ctrl_100ng_2 | Unknown | 34.65 | | | |
| ☑ | L6 | S-ctrl_100ng_3 | Unknown | 34.95 | | | |
| ☑ | L8 | S-181_100ng_1 | Unknown | 35.54 | | | |
| ☑ | L10 | S-181_100ng_2 | Unknown | 26.79 | | | |
| ☑ | L12 | S-181_100ng_3 | Unknown | 27.23 | | | |
| ☑ | L14 | S-182_100ng_1 | Unknown | 30.44 | | | |
| ☑ | L16 | S-182_100ng_2 | Unknown | 29.03 | | | |
| ☑ | L18 | S-182_100ng_3 | Unknown | 28.09 | | | |
| ☑ | L20 | S-494_100ng_1 | Unknown | 32.87 | | | |
| ☑ | L22 | S-494_100ng_2 | Unknown | 27.67 | | | |
| ☑ | L24 | S-494_100ng_3 | Unknown | 27.90 | | | |
| ☑ | M2 | S-ctrl_100ng_1 | Unknown | 45.82 | | | |
| ☑ | M4 | S-ctrl_100ng_2 | Unknown | 33.97 | | | |
| ☑ | M6 | S-ctrl_100ng_3 | Unknown | 36.47 | | | |
| ☑ | M8 | S-181_100ng_1 | Unknown | 34.96 | | | |
| ☑ | M10 | S-181_100ng_2 | Unknown | 27.63 | | | |
| ☑ | M12 | S-181_100ng_3 | Unknown | 28.87 | | | |
| ☑ | M14 | S-182_100ng_1 | Unknown | 29.96 | | | |
| ☑ | M16 | S-182_100ng_2 | Unknown | 28.50 | | | |
| ☑ | M18 | S-182_100ng_3 | Unknown | 27.72 | | | |
| ☑ | M20 | S-494_100ng_1 | Unknown | 32.46 | | | |
| ☑ | M22 | S-494_100ng_2 | Unknown | 27.85 | | | |
| ☑ | M24 | S-494_100ng_3 | Unknown | 28.86 | | | |
| ☑ | N2 | S-ctrl_50ng_1 | Unknown | 46.13 | | | |
| ☑ | N4 | S-ctrl_50ng_2 | Unknown | 37.20 | | | |
| ☑ | N6 | S-ctrl_50ng_3 | Unknown | 36.56 | | | |
| ☑ | N8 | S-181_50ng_1 | Unknown | 36.51 | | | |
| ☑ | N10 | S-181_50ng_2 | Unknown | 32.67 | | | |
| ☑ | N12 | S-181_50ng_3 | Unknown | 38.48 | | | |
| ☑ | N14 | S-182_50ng_1 | Unknown | 37.80 | | | |
| ☑ | N16 | S-182_50ng_2 | Unknown | 35.41 | | | |
| ☑ | N18 | S-182_50ng_3 | Unknown | 37.84 | | | |
| ☑ | N20 | S-494_50ng_1 | Unknown | 35.93 | | | |
| ☑ | N22 | S-494_50ng_2 | Unknown | 36.95 | | | |
| ☑ | N24 | S-494_50ng_3 | Unknown | 37.64 | | | |
| ☑ | O2 | S-ctrl_50ng_1 | Unknown | | | | |

**Results**

| Inc | Pos | Name | Type | CP | Concentration | Standard | Status |
|---|---|---|---|---|---|---|---|
| ☑ | O4 | S-ctrl_50ng_2 | Unknown | 36.11 | | | |
| ☑ | O6 | S-ctrl_50ng_3 | Unknown | 34.61 | | | |
| ☑ | O8 | S-181_50ng_1 | Unknown | 37.16 | | | |
| ☑ | O10 | S-181_50ng_2 | Unknown | 33.78 | | | |
| ☑ | O12 | S-181_50ng_3 | Unknown | 35.14 | | | |
| ☑ | O14 | S-182_50ng_1 | Unknown | 38.23 | | | |
| ☑ | O16 | S-182_50ng_2 | Unknown | 37.07 | | | |
| ☑ | O18 | S-182_50ng_3 | Unknown | 35.08 | | | |
| ☑ | O20 | S-494_50ng_1 | Unknown | 38.73 | | | |
| ☑ | O22 | S-494_50ng_2 | Unknown | 36.04 | | | |
| ☑ | O24 | S-494_50ng_3 | Unknown | 36.71 | | | |
| ☑ | P2 | S-ctrl_50ng_1 | Unknown | 44.55 | | | |
| ☑ | P4 | S-ctrl_50ng_2 | Unknown | 38.27 | | | |
| ☑ | P6 | S-ctrl_50ng_3 | Unknown | 43.04 | | | |
| ☑ | P8 | S-181_50ng_1 | Unknown | 37.72 | | | |
| ☑ | P10 | S-181_50ng_2 | Unknown | 33.29 | | | |
| ☑ | P12 | S-181_50ng_3 | Unknown | | | | |
| ☑ | P14 | S-182_50ng_1 | Unknown | 40.91 | | | |
| ☑ | P16 | S-182_50ng_2 | Unknown | 38.54 | | | |
| ☑ | P18 | S-182_50ng_3 | Unknown | 41.22 | | | |
| ☑ | P20 | S-494_50ng_1 | Unknown | 40.66 | | | |
| ☑ | P22 | S-494_50ng_2 | Unknown | 40.15 | | | |
| ☑ | P24 | S-494_50ng_3 | Unknown | | | | |

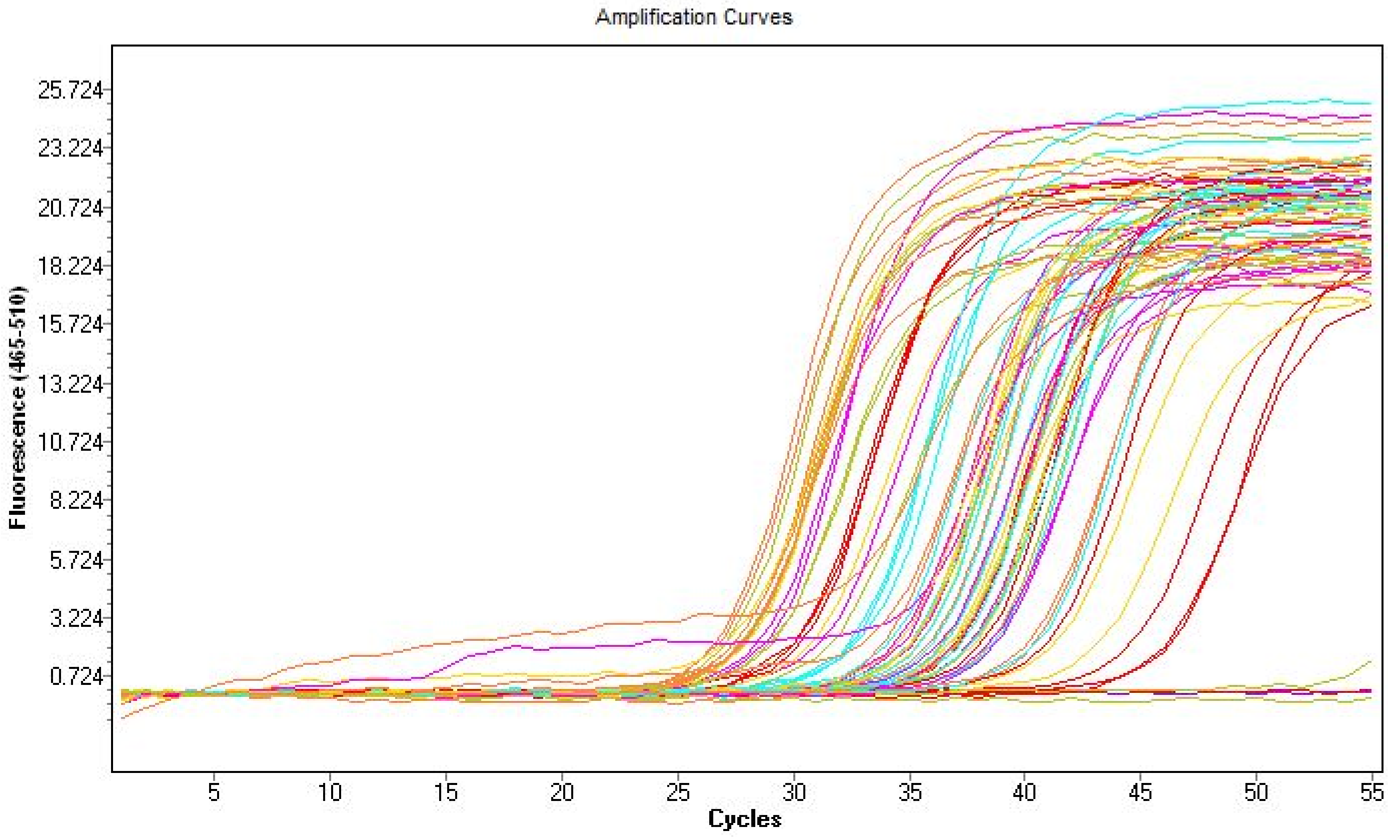


## Tm Calling for HPRT (Tm Calling)

### Results

| Inc | Pos | Sample Name | Peak 1 | | | | Peak 2 | | | | Status |
|---|---|---|---|---|---|---|---|---|---|---|---|
| | | | Tm | Area | Width | Height | Tm | Area | Width | Height | |
| ☑ | J19 | RT- | 80.92 | 20.02 | 2.76 | 7.26 | | | | | |
| ☑ | J20 | K- | 79.74 | 15.38 | 2.53 | 6.07 | | | | | |
| ☑ | J21 | K- | | | | | | | | | |
| ☑ | K1 | S-ctrl_100ng_1 | 80.01 | 17.27 | 2.70 | 6.38 | | | | | |
| ☑ | K3 | S-ctrl_100ng_2 | 79.92 | 17.51 | 2.69 | 6.50 | | | | | |
| ☑ | K5 | S-ctrl_100ng_3 | 79.86 | 17.50 | 2.58 | 6.78 | | | | | |
| ☑ | K7 | S-181_100ng_1 | 79.88 | 18.38 | 2.69 | 6.83 | | | | | |
| ☑ | K9 | S-181_100ng_2 | 79.91 | 20.02 | 2.70 | 7.41 | | | | | |
| ☑ | K11 | S-181_100ng_3 | 79.90 | 20.80 | 2.70 | 7.70 | | | | | |
| ☑ | K13 | S-182_100ng_1 | 79.88 | 20.33 | 2.71 | 7.50 | | | | | |
| ☑ | K15 | S-182_100ng_2 | 79.94 | 21.84 | 2.68 | 8.13 | | | | | |
| ☑ | K17 | S-182_100ng_3 | 79.98 | 19.92 | 2.70 | 7.38 | | | | | |
| ☑ | K19 | S-494_100ng_1 | 79.98 | 20.45 | 2.71 | 7.54 | | | | | |
| ☑ | K21 | S-494_100ng_2 | 80.01 | 19.61 | 2.70 | 7.26 | | | | | |
| ☑ | K23 | S-494_100ng_3 | 80.09 | 18.80 | 2.70 | 6.96 | | | | | |

**Results**

| Inc | Pos | Sample Name | Peak 1 Tm | Area | Width | Height | Peak 2 Tm | Area | Width | Height | Status |
|---|---|---|---|---|---|---|---|---|---|---|---|
| ☑ | L1 | S-ctrl_100ng_1 | 80.40 | 18.06 | 2.79 | 6.47 | | | | | |
| ☑ | L3 | S-ctrl_100ng_2 | 80.28 | 18.12 | 2.79 | 6.50 | | | | | |
| ☑ | L5 | S-ctrl_100ng_3 | 80.42 | 17.73 | 2.72 | 6.51 | | | | | |
| ☑ | L7 | S-181_100ng_1 | 80.21 | 19.82 | 2.78 | 7.14 | | | | | |
| ☑ | L9 | S-181_100ng_2 | 80.36 | 19.12 | 2.81 | 6.80 | | | | | |
| ☑ | L11 | S-181_100ng_3 | 80.58 | 19.23 | 2.80 | 6.86 | | | | | |
| ☑ | L13 | S-182_100ng_1 | 80.18 | 21.31 | 2.76 | 7.72 | | | | | |
| ☑ | L15 | S-182_100ng_2 | 80.32 | 21.66 | 2.79 | 7.77 | | | | | |
| ☑ | L17 | S-182_100ng_3 | 80.64 | 15.51 | 2.80 | 5.54 | | | | | |
| ☑ | L19 | S-494_100ng_1 | 80.33 | 20.69 | 2.81 | 7.37 | | | | | |
| ☑ | L21 | S-494_100ng_2 | 80.44 | 20.56 | 2.78 | 7.40 | | | | | |
| ☑ | L23 | S-494_100ng_3 | 80.82 | 19.62 | 2.83 | 6.93 | | | | | |
| ☑ | M1 | S-ctrl_100ng_1 | 81.38 | 15.27 | 3.11 | 4.91 | | | | | |
| ☑ | M3 | S-ctrl_100ng_2 | 81.31 | 15.69 | 2.84 | 5.52 | | | | | |
| ☑ | M5 | S-ctrl_100ng_3 | 81.30 | 14.95 | 2.77 | 5.40 | | | | | |
| ☑ | M7 | S-181_100ng_1 | 81.20 | 17.02 | 2.86 | 5.94 | | | | | |
| ☑ | M9 | S-181_100ng_2 | 81.31 | 17.33 | 2.88 | 6.02 | | | | | |
| ☑ | M11 | S-181_100ng_3 | 81.36 | 16.72 | 2.88 | 5.81 | | | | | |
| ☑ | M13 | S-182_100ng_1 | 81.16 | 19.12 | 2.83 | 6.75 | | | | | |
| ☑ | M15 | S-182_100ng_2 | 81.27 | 16.48 | 2.80 | 5.89 | | | | | |
| ☑ | M17 | S-182_100ng_3 | 81.47 | 13.75 | 2.83 | 4.86 | | | | | |
| ☑ | M19 | S-494_100ng_1 | 81.27 | 18.67 | 2.87 | 6.49 | | | | | |
| ☑ | M21 | S-494_100ng_2 | 81.38 | 17.15 | 2.87 | 5.97 | | | | | |
| ☑ | M23 | S-494_100ng_3 | 81.55 | 14.97 | 2.81 | 5.32 | | | | | |
| ☑ | N1 | S-ctrl_50ng_1 | 80.07 | 15.91 | 2.69 | 5.91 | | | | | |
| ☑ | N3 | S-ctrl_50ng_2 | 79.99 | 17.57 | 2.70 | 6.51 | | | | | |
| ☑ | N5 | S-ctrl_50ng_3 | 79.86 | 15.71 | 2.61 | 6.02 | | | | | |
| ☑ | N7 | S-181_50ng_1 | 79.91 | 17.96 | 2.68 | 6.70 | | | | | |
| ☑ | N9 | S-181_50ng_2 | 79.84 | 17.47 | 2.66 | 6.56 | | | | | |
| ☑ | N11 | S-181_50ng_3 | 79.88 | 18.76 | 2.69 | 6.97 | | | | | |
| ☑ | N13 | S-182_50ng_1 | 79.82 | 18.95 | 2.70 | 7.03 | | | | | |
| ☑ | N15 | S-182_50ng_2 | 79.89 | 19.13 | 2.68 | 7.15 | | | | | |
| ☑ | N17 | S-182_50ng_3 | 79.98 | 18.59 | 2.70 | 6.90 | | | | | |
| ☑ | N19 | S-494_50ng_1 | 79.94 | 17.64 | 2.63 | 6.72 | | | | | |
| ☑ | N21 | S-494_50ng_2 | 80.01 | 18.50 | 2.71 | 6.84 | | | | | |

**Results**

| Inc | Pos | Sample Name | Peak 1 | | | | Peak 2 | | | | Status |
|---|---|---|---|---|---|---|---|---|---|---|---|
| | | | Tm | Area | Width | Height | Tm | Area | Width | Height | |
| ☑ | N23 | S-494_50ng_3 | 80.09 | 17.89 | 2.71 | 6.60 | | | | | |
| ☑ | O1 | S-ctrl_50ng_1 | 80.30 | 15.38 | 2.77 | 5.54 | | | | | |
| ☑ | O3 | S-ctrl_50ng_2 | 80.36 | 18.62 | 2.79 | 6.67 | | | | | |
| ☑ | O5 | S-ctrl_50ng_3 | 80.69 | 17.86 | 2.79 | 6.39 | | | | | |
| ☑ | O7 | S-181_50ng_1 | 80.22 | 20.35 | 2.74 | 7.43 | | | | | |
| ☑ | O9 | S-181_50ng_2 | 80.28 | 19.01 | 2.76 | 6.89 | | | | | |
| ☑ | O11 | S-181_50ng_3 | 81.01 | 18.24 | 2.82 | 6.47 | | | | | |
| ☑ | O13 | S-182_50ng_1 | 80.12 | 20.60 | 2.76 | 7.47 | | | | | |
| ☑ | O15 | S-182_50ng_2 | 80.15 | 18.68 | 2.72 | 6.86 | | | | | |
| ☑ | O17 | S-182_50ng_3 | 80.68 | 18.84 | 2.79 | 6.75 | | | | | |
| ☑ | O19 | S-494_50ng_1 | 80.40 | 18.60 | 2.75 | 6.77 | | | | | |
| ☑ | O21 | S-494_50ng_2 | 80.29 | 18.57 | 2.70 | 6.89 | | | | | |
| ☑ | O23 | S-494_50ng_3 | 80.67 | 18.71 | 2.80 | 6.68 | | | | | |
| ☑ | P1 | S-ctrl_50ng_1 | 81.46 | 14.81 | 3.03 | 4.89 | | | | | |
| ☑ | P3 | S-ctrl_50ng_2 | 81.51 | 15.44 | 2.84 | 5.43 | | | | | |
| ☑ | P5 | S-ctrl_50ng_3 | 81.70 | 15.39 | 2.84 | 5.43 | | | | | |
| ☑ | P7 | S-181_50ng_1 | 81.33 | 18.18 | 2.85 | 6.38 | | | | | |
| ☑ | P9 | S-181_50ng_2 | 81.36 | 16.29 | 2.83 | 5.76 | | | | | |
| ☑ | P11 | S-181_50ng_3 | 81.44 | 16.66 | 2.82 | 5.91 | | | | | |
| ☑ | P13 | S-182_50ng_1 | 81.24 | 18.06 | 2.83 | 6.38 | | | | | |
| ☑ | P15 | S-182_50ng_2 | 81.26 | 15.94 | 2.72 | 5.86 | | | | | |
| ☑ | P17 | S-182_50ng_3 | 81.51 | 14.38 | 2.77 | 5.19 | | | | | |
| ☑ | P19 | S-494_50ng_1 | 81.57 | 16.37 | 2.84 | 5.77 | | | | | |
| ☑ | P21 | S-494_50ng_2 | 81.43 | 18.21 | 2.80 | 6.50 | | | | | |
| ☑ | P23 | S-494_50ng_3 | 81.59 | 15.02 | 2.92 | 5.14 | | | | | |

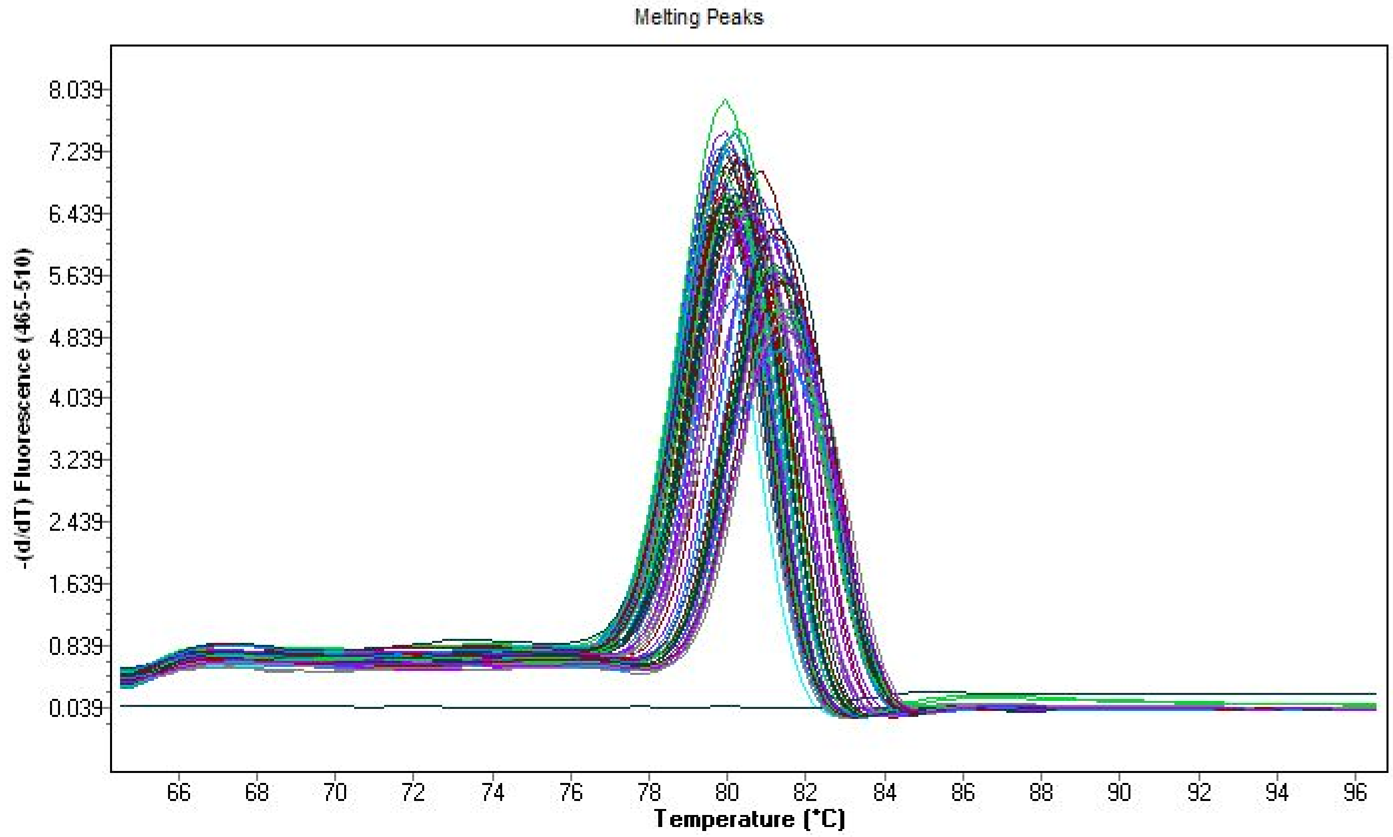


## Tm Calling for AIT (Tm Calling)

### Results

| Inc | Pos | Sample Name | Peak 1 Tm | Peak 1 Area | Peak 1 Width | Peak 1 Height | Peak 2 Tm | Peak 2 Area | Peak 2 Width | Peak 2 Height | Status |
|---|---|---|---|---|---|---|---|---|---|---|---|
| ☑ | J22 | RT- | 81.02 | 16.53 | 2.68 | 6.17 | | | | | |
| ☑ | J23 | K- | | | | | | | | | |
| ☑ | J24 | K- | 80.18 | 13.77 | 2.29 | 6.01 | | | | | |
| ☑ | K2 | S-ctrl_100ng_1 | 80.44 | 17.09 | 2.91 | 5.88 | | | | | |
| ☑ | K4 | S-ctrl_100ng_2 | 80.15 | 15.39 | 2.32 | 6.64 | | | | | |
| ☑ | K6 | S-ctrl_100ng_3 | 80.10 | 13.81 | 2.31 | 5.98 | | | | | |
| ☑ | K8 | S-181_100ng_1 | 80.13 | 15.62 | 2.30 | 6.79 | | | | | |
| ☑ | K10 | S-181_100ng_2 | 80.13 | 17.13 | 2.33 | 7.36 | | | | | |
| ☑ | K12 | S-181_100ng_3 | 80.07 | 16.57 | 2.34 | 7.09 | | | | | |
| ☑ | K14 | S-182_100ng_1 | 81.35 | 17.83 | 2.81 | 6.36 | | | | | |
| ☑ | K16 | S-182_100ng_2 | 81.50 | 16.41 | 2.82 | 5.82 | | | | | |
| ☑ | K18 | S-182_100ng_3 | 81.55 | 16.09 | 2.84 | 5.66 | | | | | |
| ☑ | K20 | S-494_100ng_1 | 81.47 | 18.57 | 2.80 | 6.62 | | | | | |
| ☑ | K22 | S-494_100ng_2 | 81.47 | 15.05 | 2.80 | 5.37 | | | | | |
| ☑ | K24 | S-494_100ng_3 | 81.63 | 14.28 | 2.78 | 5.14 | | | | | |

**Results**

| Inc | Pos | Sample Name | Peak 1 Tm | Peak 1 Area | Peak 1 Width | Peak 1 Height | Peak 2 Tm | Peak 2 Area | Peak 2 Width | Peak 2 Height | Status |
|---|---|---|---|---|---|---|---|---|---|---|---|
| ☑ | L2 | S-ctrl_100ng_1 | 80.65 | 15.97 | 3.35 | 4.77 | | | | | |
| ☑ | L4 | S-ctrl_100ng_2 | 80.59 | 16.02 | 2.44 | 6.56 | | | | | |
| ☑ | L6 | S-ctrl_100ng_3 | 80.63 | 15.41 | 2.43 | 6.35 | | | | | |
| ☑ | L8 | S-181_100ng_1 | 80.47 | 16.25 | 2.41 | 6.73 | | | | | |
| ☑ | L10 | S-181_100ng_2 | 80.62 | 18.11 | 2.46 | 7.36 | | | | | |
| ☑ | L12 | S-181_100ng_3 | 80.72 | 17.86 | 2.48 | 7.21 | | | | | |
| ☑ | L14 | S-182_100ng_1 | 80.46 | 16.41 | 2.43 | 6.76 | | | | | |
| ☑ | L16 | S-182_100ng_2 | 80.56 | 18.49 | 2.44 | 7.59 | | | | | |
| ☑ | L18 | S-182_100ng_3 | 80.76 | 17.23 | 2.49 | 6.92 | | | | | |
| ☑ | L20 | S-494_100ng_1 | 80.55 | 18.72 | 2.44 | 7.67 | | | | | |
| ☑ | L22 | S-494_100ng_2 | 80.74 | 16.58 | 2.48 | 6.69 | | | | | |
| ☑ | L24 | S-494_100ng_3 | 81.04 | 15.75 | 2.51 | 6.27 | | | | | |
| ☑ | M2 | S-ctrl_100ng_1 | 81.49 | 13.63 | 3.07 | 4.44 | | | | | |
| ☑ | M4 | S-ctrl_100ng_2 | 81.41 | 14.07 | 2.81 | 5.01 | | | | | |
| ☑ | M6 | S-ctrl_100ng_3 | 81.39 | 13.62 | 2.80 | 4.86 | | | | | |
| ☑ | M8 | S-181_100ng_1 | 81.35 | 16.02 | 2.85 | 5.62 | | | | | |
| ☑ | M10 | S-181_100ng_2 | 81.42 | 16.96 | 2.81 | 6.04 | | | | | |
| ☑ | M12 | S-181_100ng_3 | 81.36 | 15.30 | 2.82 | 5.43 | | | | | |
| ☑ | M14 | S-182_100ng_1 | 80.09 | 16.06 | 2.33 | 6.89 | | | | | |
| ☑ | M16 | S-182_100ng_2 | 80.18 | 16.29 | 2.31 | 7.06 | | | | | |
| ☑ | M18 | S-182_100ng_3 | 80.20 | 16.21 | 2.32 | 6.98 | | | | | |
| ☑ | M20 | S-494_100ng_1 | 80.23 | 15.71 | 2.33 | 6.74 | | | | | |
| ☑ | M22 | S-494_100ng_2 | 80.26 | 15.28 | 2.35 | 6.51 | | | | | |
| ☑ | M24 | S-494_100ng_3 | 80.35 | 14.21 | 2.34 | 6.09 | | | | | |
| ☑ | N2 | S-ctrl_50ng_1 | 82.53 | 12.68 | 2.09 | 6.06 | | | | | |
| ☑ | N4 | S-ctrl_50ng_2 | 80.19 | 13.47 | 2.28 | 5.90 | | | | | |
| ☑ | N6 | S-ctrl_50ng_3 | 80.22 | 14.40 | 2.43 | 5.92 | | | | | |
| ☑ | N8 | S-181_50ng_1 | 80.20 | 15.37 | 2.32 | 6.63 | | | | | |
| ☑ | N10 | S-181_50ng_2 | 79.96 | 12.56 | 2.35 | 5.34 | | | | | |
| ☑ | N12 | S-181_50ng_3 | 80.16 | 15.46 | 2.30 | 6.72 | | | | | |
| ☑ | N14 | S-182_50ng_1 | 80.17 | 15.71 | 2.36 | 6.65 | | | | | |
| ☑ | N16 | S-182_50ng_2 | 80.39 | 15.45 | 4.04 | 3.82 | | | | | |
| ☑ | N18 | S-182_50ng_3 | 80.43 | 16.84 | 2.88 | 5.84 | | | | | |
| ☑ | N20 | S-494_50ng_1 | 80.28 | 15.97 | 2.33 | 6.86 | | | | | |
| ☑ | N22 | S-494_50ng_2 | 80.36 | 14.89 | 2.36 | 6.32 | | | | | |

**Results**

| Inc | Pos | Sample Name | Peak 1 | | | | Peak 2 | | | | Status |
|---|---|---|---|---|---|---|---|---|---|---|---|
| | | | Tm | Area | Width | Height | Tm | Area | Width | Height | |
| ☑ | N24 | S-494_50ng_3 | 80.71 | 14.96 | 2.42 | 6.19 | | | | | |
| ☑ | O2 | S-ctrl_50ng_1 | | | | | | | | | |
| ☑ | O4 | S-ctrl_50ng_2 | 80.51 | 13.42 | 2.46 | 5.45 | | | | | |
| ☑ | O6 | S-ctrl_50ng_3 | 80.93 | 15.17 | 2.48 | 6.12 | | | | | |
| ☑ | O8 | S-181_50ng_1 | 80.53 | 15.59 | 2.42 | 6.45 | | | | | |
| ☑ | O10 | S-181_50ng_2 | 80.44 | 15.25 | 2.68 | 5.68 | | | | | |
| ☑ | O12 | S-181_50ng_3 | 81.00 | 17.07 | 2.65 | 6.44 | | | | | |
| ☑ | O14 | S-182_50ng_1 | 80.45 | 16.77 | 2.41 | 6.96 | | | | | |
| ☑ | O16 | S-182_50ng_2 | 80.40 | 14.55 | 2.38 | 6.12 | | | | | |
| ☑ | O18 | S-182_50ng_3 | 80.95 | 16.61 | 2.49 | 6.68 | | | | | |
| ☑ | O20 | S-494_50ng_1 | 80.53 | 16.71 | 2.41 | 6.92 | | | | | |
| ☑ | O22 | S-494_50ng_2 | 80.46 | 13.06 | 2.40 | 5.45 | 85.43 | 4.36 | 2.35 | 1.86 | |
| ☑ | O24 | S-494_50ng_3 | 80.97 | 13.87 | 2.61 | 5.32 | | | | | |
| ☑ | P2 | S-ctrl_50ng_1 | 81.52 | 14.13 | 2.93 | 4.83 | | | | | |
| ☑ | P4 | S-ctrl_50ng_2 | 81.58 | 14.58 | 2.79 | 5.22 | | | | | |
| ☑ | P6 | S-ctrl_50ng_3 | 81.61 | 14.15 | 2.84 | 4.98 | | | | | |
| ☑ | P8 | S-181_50ng_1 | 81.42 | 15.13 | 2.72 | 5.57 | | | | | |
| ☑ | P10 | S-181_50ng_2 | 81.34 | 14.92 | 2.80 | 5.34 | | | | | |
| ☑ | P12 | S-181_50ng_3 | 71.99 | 0.75 | 6.11 | 0.12 | 83.65 | 2.09 | 4.88 | 0.43 | |
| ☑ | P14 | S-182_50ng_1 | 81.16 | 14.36 | 2.75 | 5.23 | | | | | |
| ☑ | P16 | S-182_50ng_2 | 81.40 | 15.47 | 2.69 | 5.74 | | | | | |
| ☑ | P18 | S-182_50ng_3 | 81.62 | 14.71 | 2.75 | 5.34 | | | | | |
| ☑ | P20 | S-494_50ng_1 | 81.42 | 16.73 | 2.77 | 6.05 | | | | | |
| ☑ | P22 | S-494_50ng_2 | 81.60 | 15.52 | 2.79 | 5.57 | | | | | |
| ☑ | P24 | S-494_50ng_3 | | | | | | | | | |

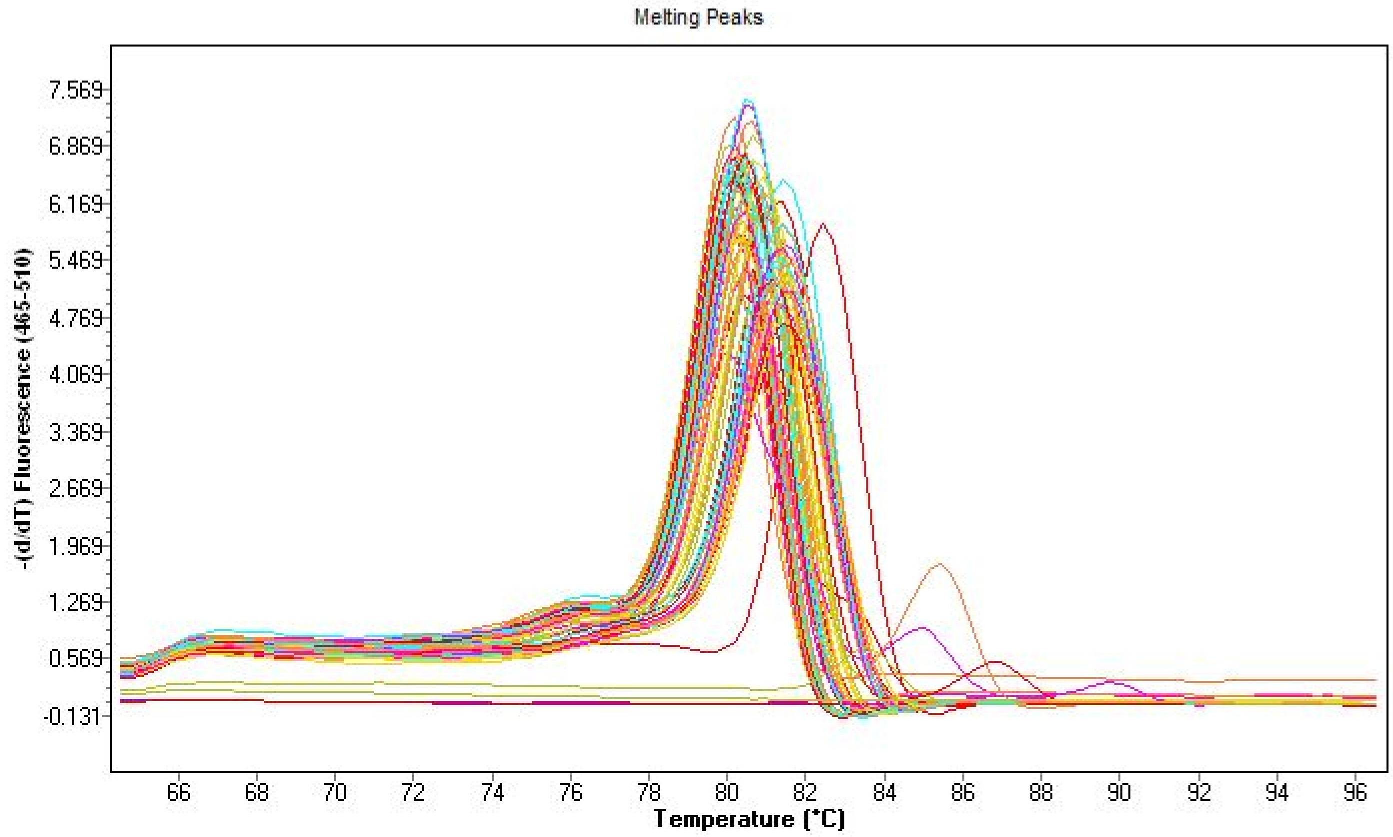

Melting Peaks
7.569
6.869
6.169
5.469
4.769
4.069
3.369
2.669
1.969
1.269
0.569
-0.131
-(d/dT) Fluorescence (465-510)
66
68
70
72
74
76
78
80
82
84
86
88
90
92
94
96
Temperature (°C)
