# Supplementary material for: MicroRNAs and Their Inhibition in Modulating SLC5A8 Expression in the Context of Papillary Thyroid Carcinoma
Source: Int J Mol Sci. 2025 Aug 15;26(16):7889. doi: 10.3390/ijms26167889 (PMC12386254; doi:10.3390/ijms26167889)
Supplement: Supplementary file 1 [file ijms-26-07889-s001.zip › ijms-3558049-supplementary/Manuscript data/Fig6C data/Sponge-single/Exp_3/2017-12-20 AIT po transfekcji HEK sponge 100ng.PDF]

Roche

Wojtek_2017-12-20 AIT po transfekcji HEK sponge 100ng

Programs

Program Name	pre-incubation		
Cycles	1	Analysis Mode	None

Target (°C)	Acquisition Mode	Hold (hh:mm:ss)	Ramp Rate (°C/s)	Acquisitions (per °C)	Sec Target (°C)	Step size (°C)	Step Delay (cycles)
95	None	00:10:00	4.80		0	0	0

Program Name	amplification		
Cycles	55	Analysis Mode	Quantification

Target (°C)	Acquisition Mode	Hold (hh:mm:ss)	Ramp Rate (°C/s)	Acquisitions (per °C)	Sec Target (°C)	Step size (°C)	Step Delay (cycles)
95	None	00:00:10	4.80		0	0	0
58	None	00:00:15	2.50		0	0	0
72	Single	00:00:10	4.80		0	0	0

Program Name	melting curve		
Cycles	1	Analysis Mode	Melting Curves

Target (°C)	Acquisition Mode	Hold (hh:mm:ss)	Ramp Rate (°C/s)	Acquisitions (per °C)	Sec Target (°C)	Step size (°C)	Step Delay (cycles)
95	None	00:00:05	4.80		0	0	0
65	None	00:01:00	2.50		0	0	0
97	Continuous		0.11	5	0	0	0

Program Name	cooling		
Cycles	1	Analysis Mode	None

Target (°C)	Acquisition Mode	Hold (hh:mm:ss)	Ramp Rate (°C/s)	Acquisitions (per °C)	Sec Target (°C)	Step size (°C)	Step Delay (cycles)
40	None	00:00:30	2.50		0	0	0

Tm Calling for All (Tm Calling)

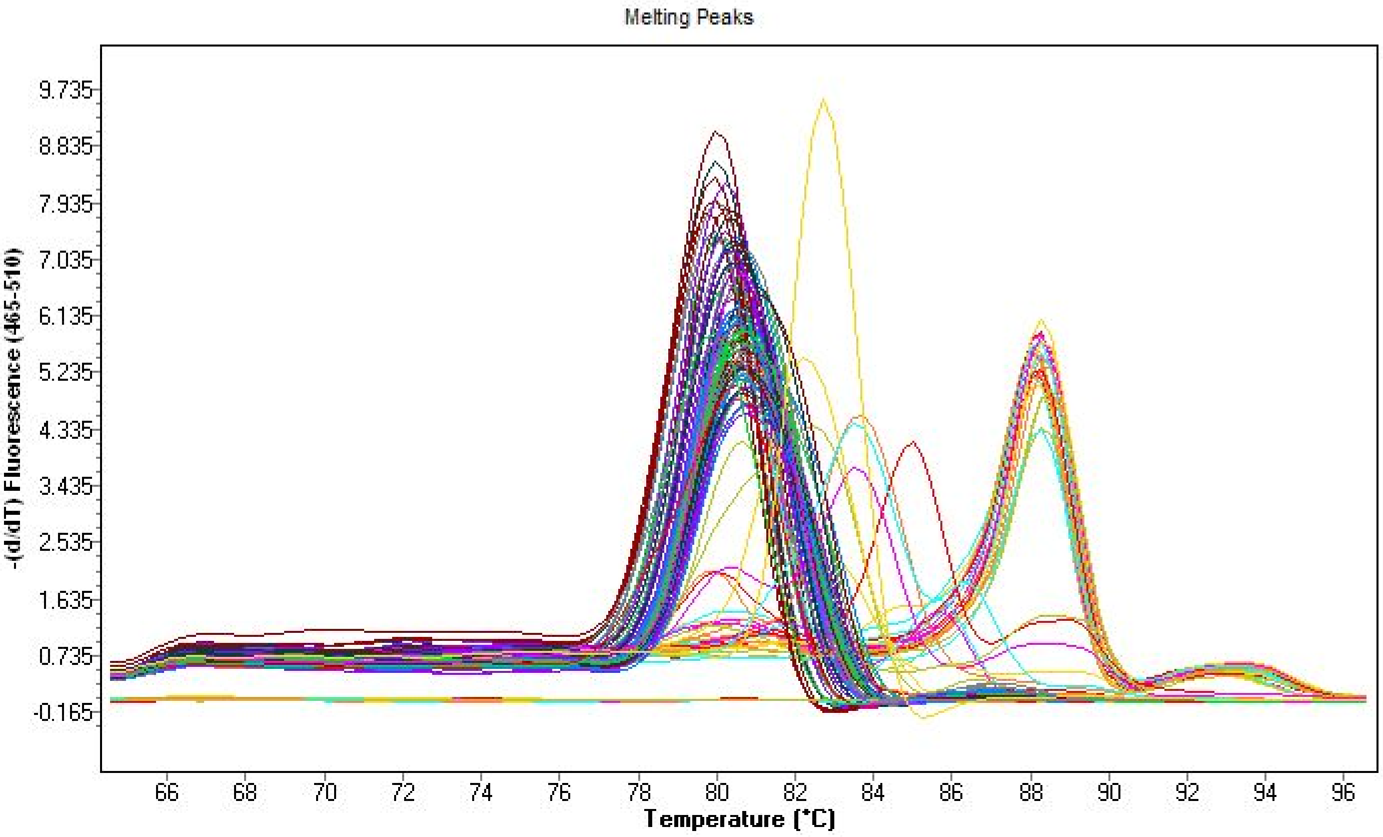

Tm Calling for HPRT_10/12 (Tm Calling)

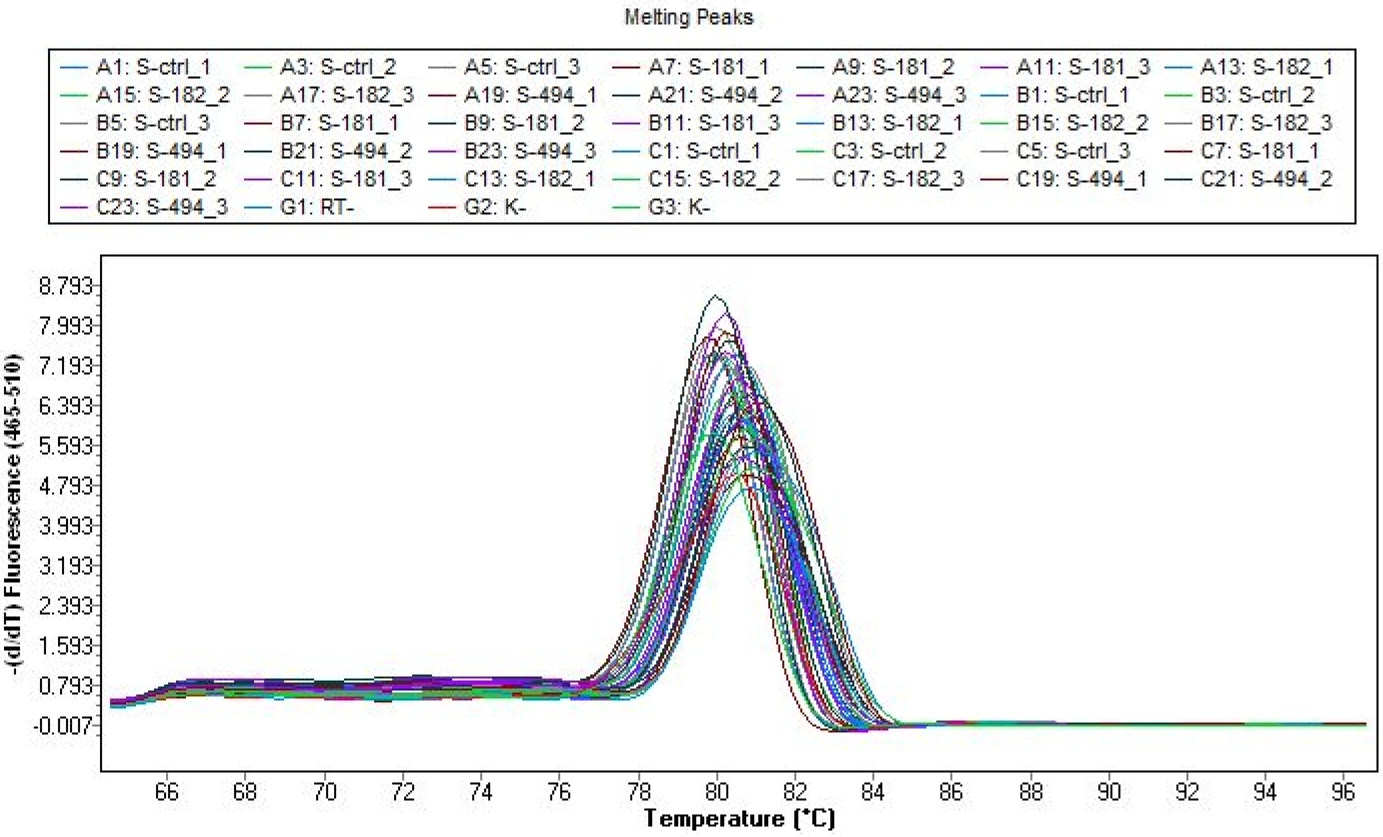

Tm Calling for AIT_10/12 (Tm Calling)

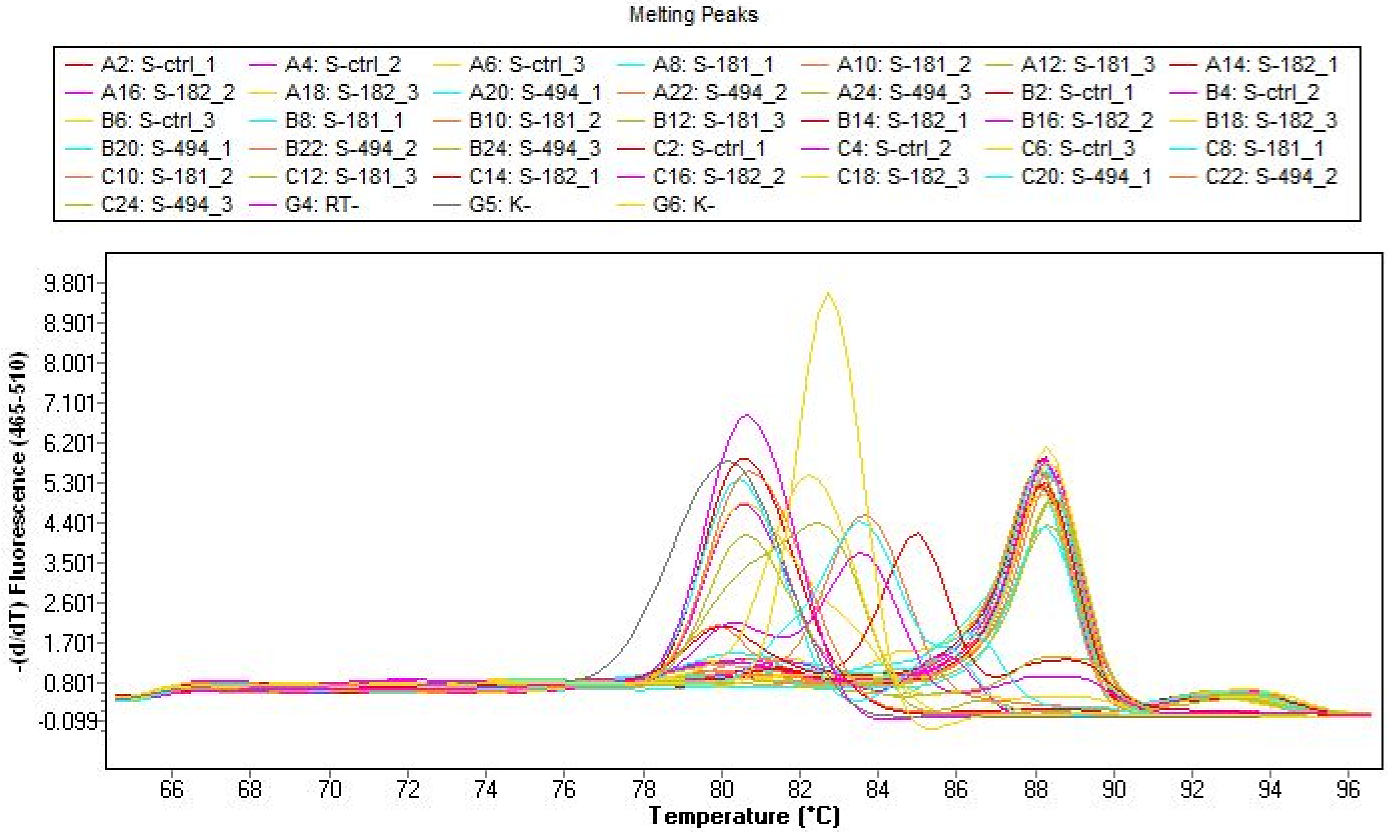

Tm Calling for HPRT_12/12 (Tm Calling)

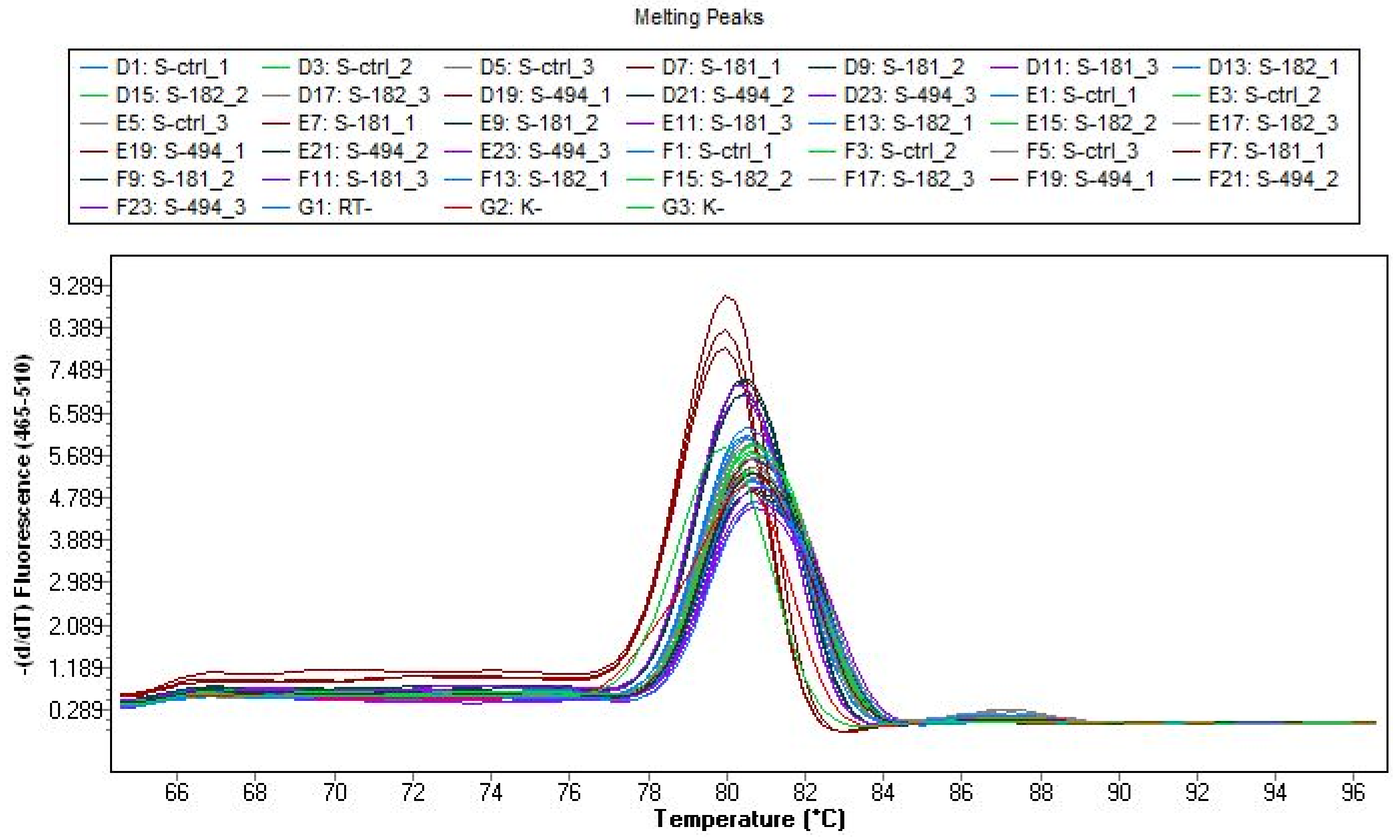

Tm Calling for AIT_12/12 (Tm Calling)

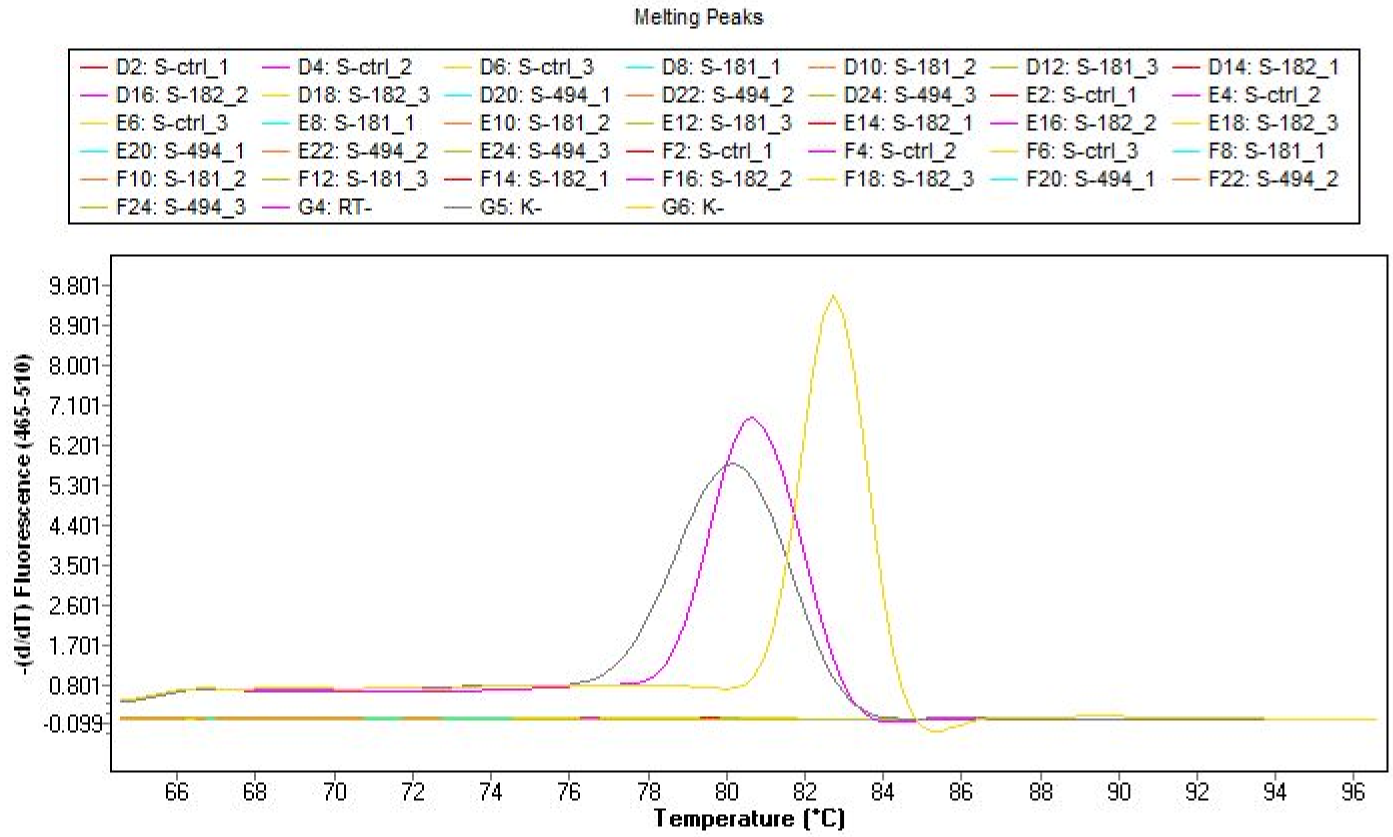

Abs Quant/2nd Derivative Max for HPRT_10/12 (Abs Quant/2nd Derivative Max)

Results

Inc	Pos	Name	Type	CP	Concentration	Standard	Status
☑	A1	S-ctrl_1	Unknown	26.42			
☑	A3	S-ctrl_2	Unknown	24.22			
☑	A5	S-ctrl_3	Unknown	25.80			
☑	A7	S-181_1	Unknown	25.30			
☑	A9	S-181_2	Unknown	24.58			
☑	A11	S-181_3	Unknown	26.03			
☑	A13	S-182_1	Unknown	27.09			
☑	A15	S-182_2	Unknown	26.79			
☑	A17	S-182_3	Unknown	27.79			
☑	A19	S-494_1	Unknown	29.03			
☑	A21	S-494_2	Unknown	28.72			
☑	A23	S-494_3	Unknown	26.86			
☑	B1	S-ctrl_1	Unknown	26.25			
☑	B3	S-ctrl_2	Unknown	23.86			
☑	B5	S-ctrl_3	Unknown	24.86			
☑	B7	S-181_1	Unknown	25.11			
☑	B9	S-181_2	Unknown	24.57			
☑	B11	S-181_3	Unknown	24.55			
☑	B13	S-182_1	Unknown	25.30			
☑	B15	S-182_2	Unknown	26.09			
☑	B17	S-182_3	Unknown	26.72			
☑	B19	S-494_1	Unknown	27.12			
☑	B21	S-494_2	Unknown	27.44			
☑	B23	S-494_3	Unknown	26.78			
☑	C1	S-ctrl_1	Unknown	25.89			
☑	C3	S-ctrl_2	Unknown	23.48			
☑	C5	S-ctrl_3	Unknown	24.29			
☑	C7	S-181_1	Unknown	24.51			
☑	C9	S-181_2	Unknown	23.62			
☑	C11	S-181_3	Unknown	23.77			
☑	C13	S-182_1	Unknown	30.72			
☑	C15	S-182_2	Unknown	22.77			
☑	C17	S-182_3	Unknown	24.29			
☑	C19	S-494_1	Unknown	33.30			
☑	C21	S-494_2	Unknown	27.97			

Results

Inc	Pos	Name	Type	CP	Concentration	Standard	Status
☑	C23	S-494_3	Unknown	26.47			
☑	G1	RT-	Negative Control	33.87			
☑	G2	K-	Negative Control	35.88			
☑	G3	K-	Negative Control	37.78			

Abs Quant/2nd Derivative Max for AIT_10/12 (Abs Quant/2nd Derivative Max)

Results

Inc	Pos	Name	Type	CP	Concentration	Standard	Status
☑	A2	S-ctrl_1	Unknown	32.85			
☑	A4	S-ctrl_2	Unknown	33.02			
☑	A6	S-ctrl_3	Unknown	33.43			
☑	A8	S-181_1	Unknown	31.03			
☑	A10	S-181_2	Unknown	30.78			
☑	A12	S-181_3	Unknown	32.51			
☑	A14	S-182_1	Unknown	31.98			
☑	A16	S-182_2	Unknown	29.57			
☑	A18	S-182_3	Unknown	32.60			
☑	A20	S-494_1	Unknown	32.25			
☑	A22	S-494_2	Unknown	31.61			
☑	A24	S-494_3	Unknown	31.23			
☑	B2	S-ctrl_1	Unknown	31.66			
☑	B4	S-ctrl_2	Unknown	31.88			
☑	B6	S-ctrl_3	Unknown	32.47			
☑	B8	S-181_1	Unknown	31.31			
☑	B10	S-181_2	Unknown	30.03			
☑	B12	S-181_3	Unknown	29.93			
☑	B14	S-182_1	Unknown	30.95			
☑	B16	S-182_2	Unknown	28.89			
☑	B18	S-182_3	Unknown	31.15			
☑	B20	S-494_1	Unknown	31.64			
☑	B22	S-494_2	Unknown	31.13			
☑	B24	S-494_3	Unknown	29.94			
☑	C2	S-ctrl_1	Unknown	30.61			
☑	C4	S-ctrl_2	Unknown	29.50			
☑	C6	S-ctrl_3	Unknown	30.46			
☑	C8	S-181_1	Unknown	28.49			

Results

Inc	Pos	Name	Type	CP	Concentration	Standard	Status
☑	C10	S-181_2	Unknown	28.14			
☑	C12	S-181_3	Unknown	28.93			
☑	C14	S-182_1	Unknown	29.69			
☑	C16	S-182_2	Unknown	28.13			
☑	C18	S-182_3	Unknown	30.72			
☑	C20	S-494_1	Unknown	30.90			
☑	C22	S-494_2	Unknown	29.90			
☑	C24	S-494_3	Unknown	30.80			
☑	G4	RT-	Negative Control	35.46			
☑	G5	K-	Negative Control	40.93			
☑	G6	K-	Negative Control	38.42			

Abs Quant/2nd Derivative Max for HPRT_12/12 (Abs Quant/2nd Derivative Max)

Results

Inc	Pos	Name	Type	CP	Concentration	Standard	Status
☑	D1	S-ctrl_1	Unknown	23.06			
☑	D3	S-ctrl_2	Unknown	22.74			
☑	D5	S-ctrl_3	Unknown	26.72			
☑	D7	S-181_1	Unknown	30.55			
☑	D9	S-181_2	Unknown	25.59			
☑	D11	S-181_3	Unknown	25.61			
☑	D13	S-182_1	Unknown	24.42			
☑	D15	S-182_2	Unknown	24.74			
☑	D17	S-182_3	Unknown	23.68			
☑	D19	S-494_1	Unknown	26.02			
☑	D21	S-494_2	Unknown	27.65			
☑	D23	S-494_3	Unknown	26.50			
☑	E1	S-ctrl_1	Unknown	23.02			
☑	E3	S-ctrl_2	Unknown	22.72			
☑	E5	S-ctrl_3	Unknown	26.82			
☑	E7	S-181_1	Unknown	30.48			
☑	E9	S-181_2	Unknown	25.59			
☑	E11	S-181_3	Unknown	25.80			
☑	E13	S-182_1	Unknown	24.41			
☑	E15	S-182_2	Unknown	24.79			
☑	E17	S-182_3	Unknown	23.72			

Results

Inc	Pos	Name	Type	CP	Concentration	Standard	Status
☑	E19	S-494_1	Unknown	26.11			
☑	E21	S-494_2	Unknown	27.64			
☑	E23	S-494_3	Unknown	26.64			
☑	F1	S-ctrl_1	Unknown	23.06			
☑	F3	S-ctrl_2	Unknown	22.77			
☑	F5	S-ctrl_3	Unknown	26.72			
☑	F7	S-181_1	Unknown	30.56			
☑	F9	S-181_2	Unknown	25.63			
☑	F11	S-181_3	Unknown	25.81			
☑	F13	S-182_1	Unknown	24.31			
☑	F15	S-182_2	Unknown	24.89			
☑	F17	S-182_3	Unknown	23.75			
☑	F19	S-494_1	Unknown	26.04			
☑	F21	S-494_2	Unknown	27.63			
☑	F23	S-494_3	Unknown	26.68			
☑	G1	RT-	Negative Control	33.87			
☑	G2	K-	Negative Control	35.88			
☑	G3	K-	Negative Control	37.78			

Abs Quant/2nd Derivative Max for AIT_12/12 (Abs Quant/2nd Derivative Max)

Results

Inc	Pos	Name	Type	CP	Concentration	Standard	Status
☑	D2	S-ctrl_1	Unknown				
☑	D4	S-ctrl_2	Unknown				
☑	D6	S-ctrl_3	Unknown				
☑	D8	S-181_1	Unknown				
☑	D10	S-181_2	Unknown				
☑	D12	S-181_3	Unknown				
☑	D14	S-182_1	Unknown				
☑	D16	S-182_2	Unknown				
☑	D18	S-182_3	Unknown				
☑	D20	S-494_1	Unknown				
☑	D22	S-494_2	Unknown				
☑	D24	S-494_3	Unknown				
☑	E2	S-ctrl_1	Unknown				
☑	E4	S-ctrl_2	Unknown				

Results

Inc	Pos	Name	Type	CP	Concentration	Standard	Status
☑	E6	S-ctrl_3	Unknown				
☑	E8	S-181_1	Unknown				
☑	E10	S-181_2	Unknown				
☑	E12	S-181_3	Unknown				
☑	E14	S-182_1	Unknown				
☑	E16	S-182_2	Unknown				
☑	E18	S-182_3	Unknown				
☑	E20	S-494_1	Unknown				
☑	E22	S-494_2	Unknown				
☑	E24	S-494_3	Unknown				
☑	F2	S-ctrl_1	Unknown				
☑	F4	S-ctrl_2	Unknown				
☑	F6	S-ctrl_3	Unknown				
☑	F8	S-181_1	Unknown				
☑	F10	S-181_2	Unknown				
☑	F12	S-181_3	Unknown				
☑	F14	S-182_1	Unknown				
☑	F16	S-182_2	Unknown				
☑	F18	S-182_3	Unknown				
☑	F20	S-494_1	Unknown				
☑	F22	S-494_2	Unknown				
☑	F24	S-494_3	Unknown				
☑	G4	RT-	Negative Control	35.46			
☑	G5	K-	Negative Control	40.93			
☑	G6	K-	Negative Control	38.42			
