# Supplementary material for: MicroRNAs and Their Inhibition in Modulating SLC5A8 Expression in the Context of Papillary Thyroid Carcinoma
Source: Int J Mol Sci. 2025 Aug 15;26(16):7889. doi: 10.3390/ijms26167889 (PMC12386254; doi:10.3390/ijms26167889)
Supplement: Supplementary file 1 [file ijms-26-07889-s001.zip › ijms-3558049-supplementary/Manuscript data/RNA-Seq/gierlikowski_210714_rnaseq_libSizes.pdf]

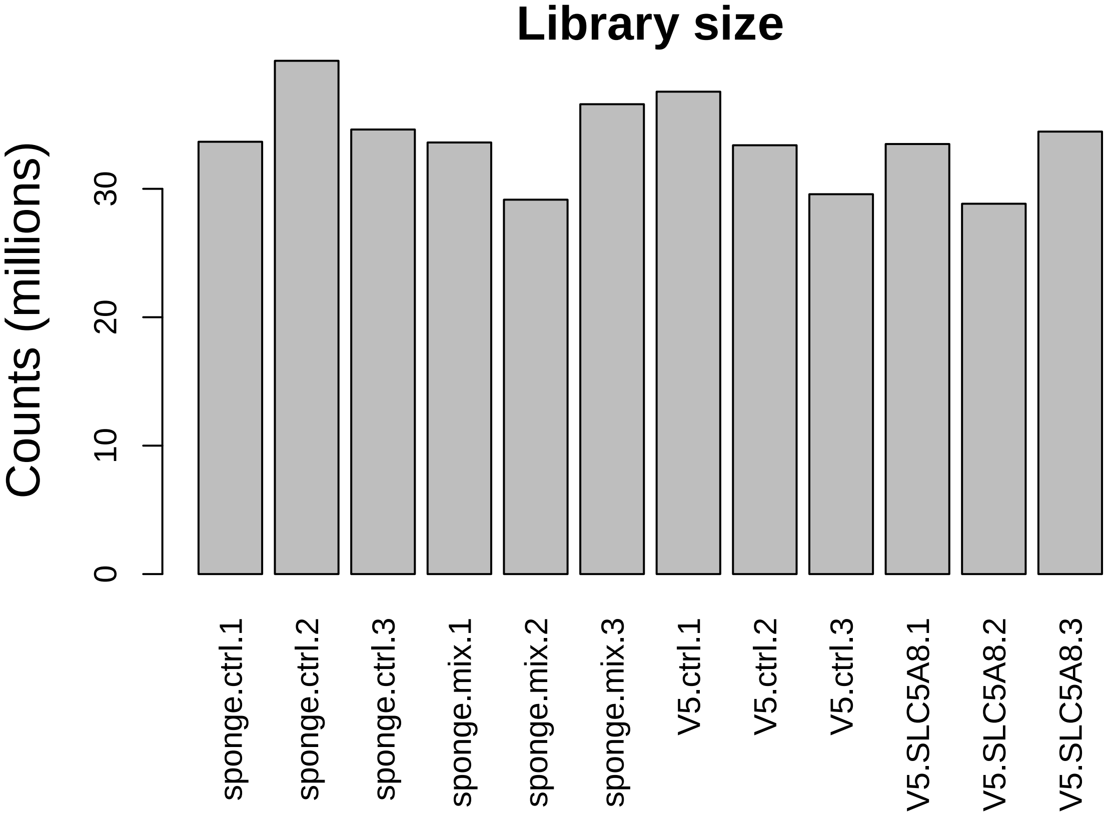

Library size
Counts (millions)
0
10
20
30
sponge.ctrl.1
sponge.ctrl.2
sponge.ctrl.3
sponge.mix.1
sponge.mix.2
sponge.mix.3
V5.ctrl.1
V5.ctrl.2
V5.ctrl.3
V5.SLC5A8.1
V5.SLC5A8.2
V5.SLC5A8.3
