# Supplementary material for: MicroRNAs and Their Inhibition in Modulating SLC5A8 Expression in the Context of Papillary Thyroid Carcinoma
Source: Int J Mol Sci. 2025 Aug 15;26(16):7889. doi: 10.3390/ijms26167889 (PMC12386254; doi:10.3390/ijms26167889)
Supplement: Supplementary file 1 [file ijms-26-07889-s001.zip › ijms-3558049-supplementary/Manuscript data/RNA-Seq/gierlikowski_210714_rnaseq_PCA_genesAll_samplesFiltered.pdf]

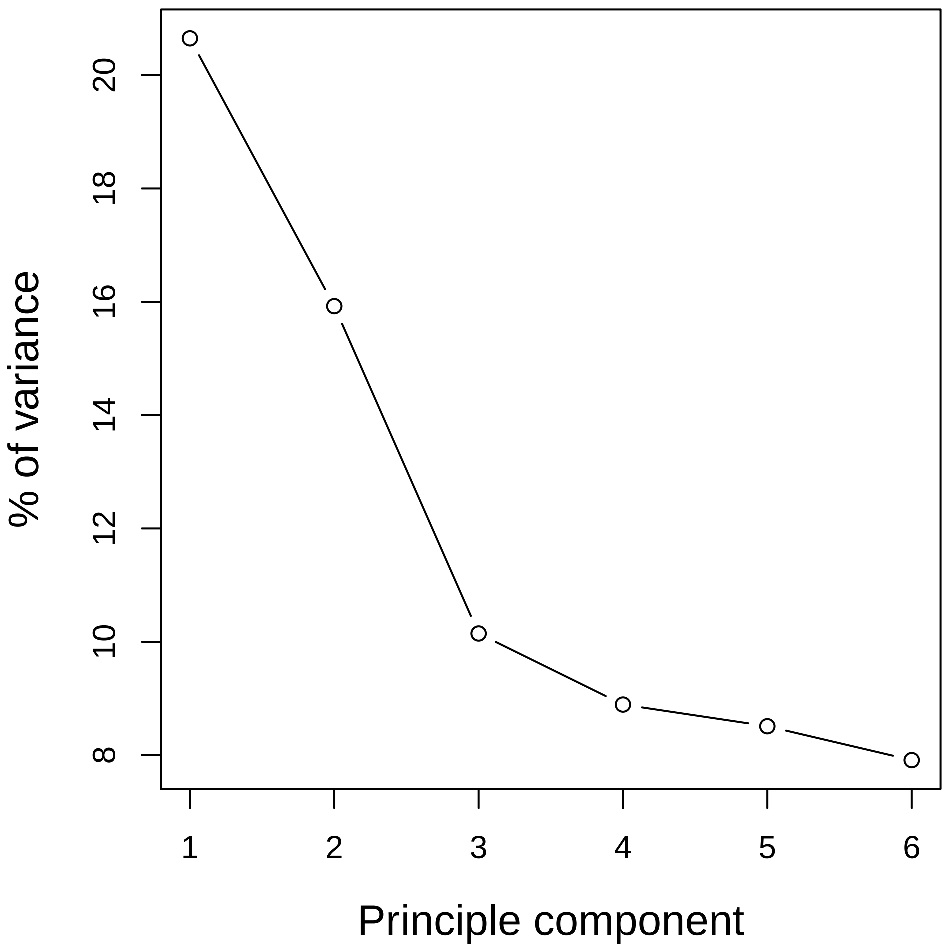

% of variance
20
18
16
14
12
10
8
1
2
3
4
5
6
Principle component

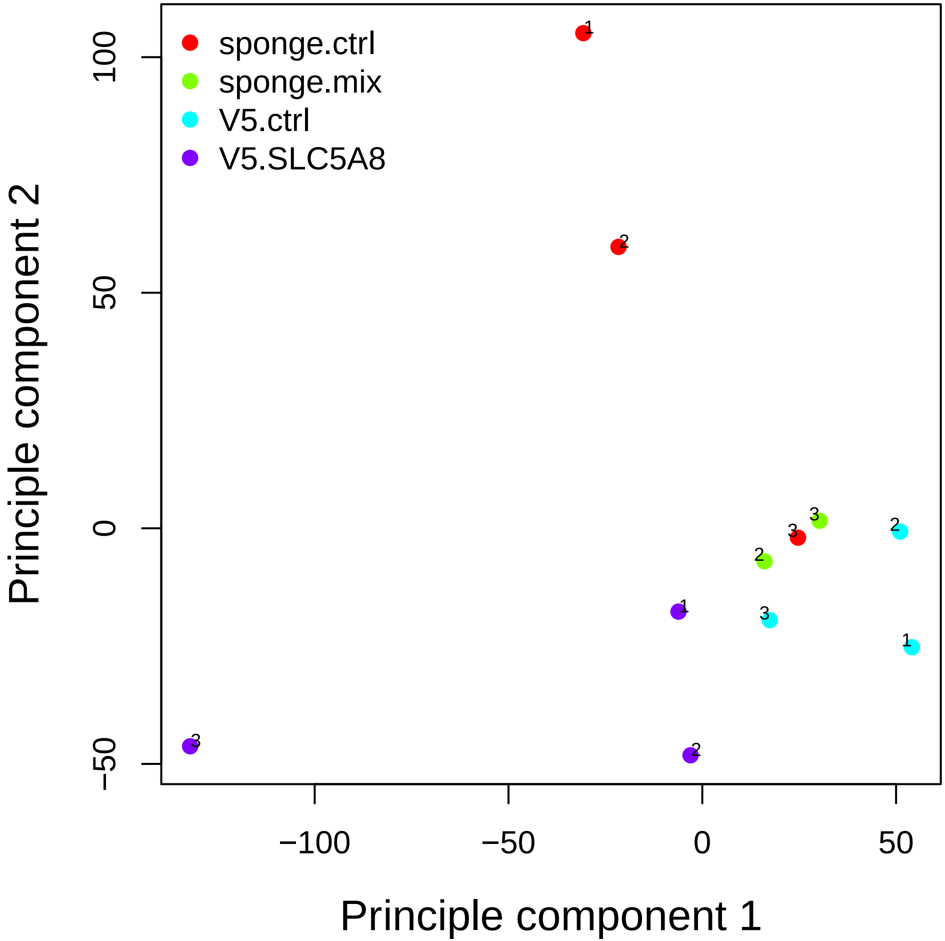
sponge.ctrl
sponge.mix
V5.ctrl
V5.SLC5A8
Principle component 1
Principle component 2
−100
−50
0
50
100
50
0
−50
1
2
3
1
2
3
1
2
3
1
2
3

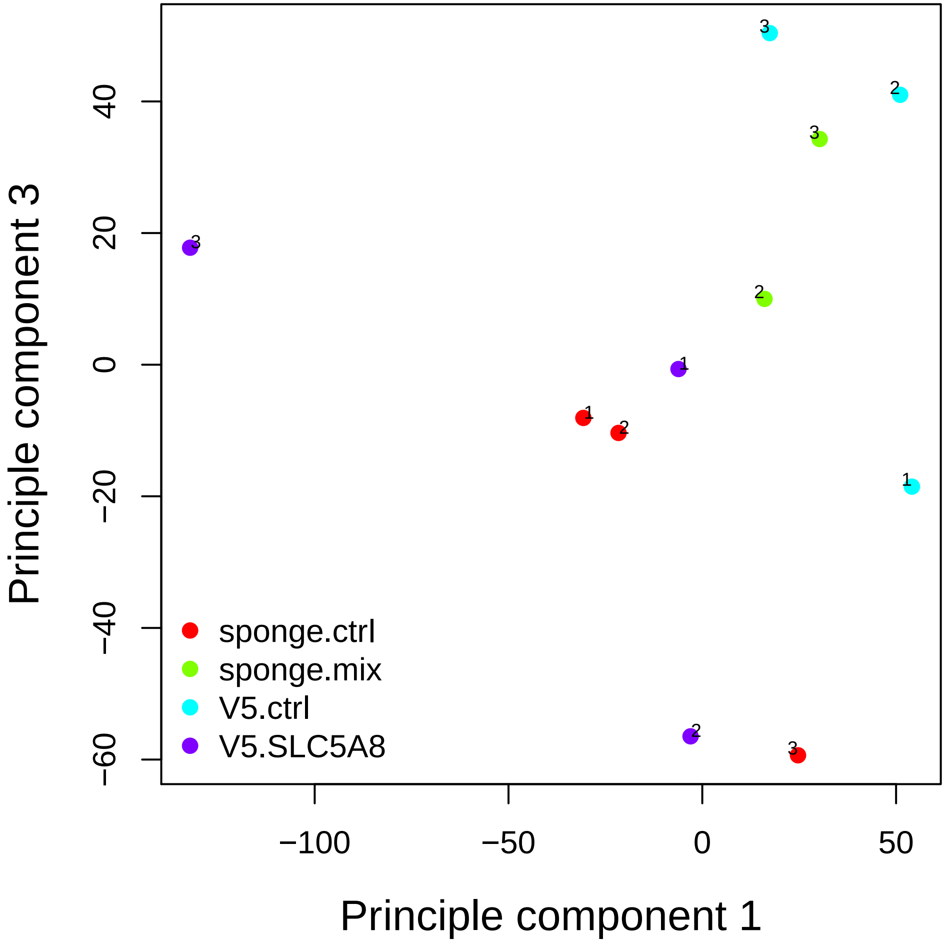

Principle component 3
Principle component 1
40
20
0
−20
−40
−60
−100
−50
0
50
sponge.ctrl
sponge.mix
V5.ctrl
V5.SLC5A8
1
2
3

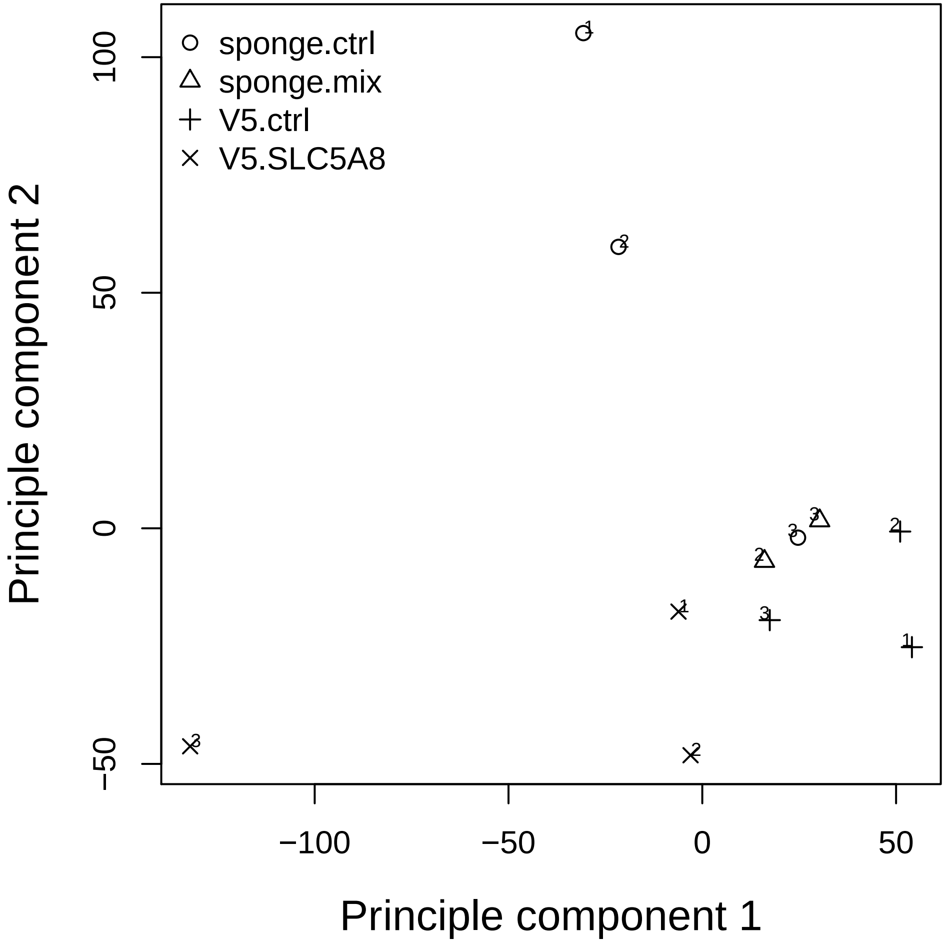
sponge.ctrl
sponge.mix
V5.ctrl
V5.SLC5A8
100
50
0
−50
−100
−50
0
50
Principle component 1
Principle component 2

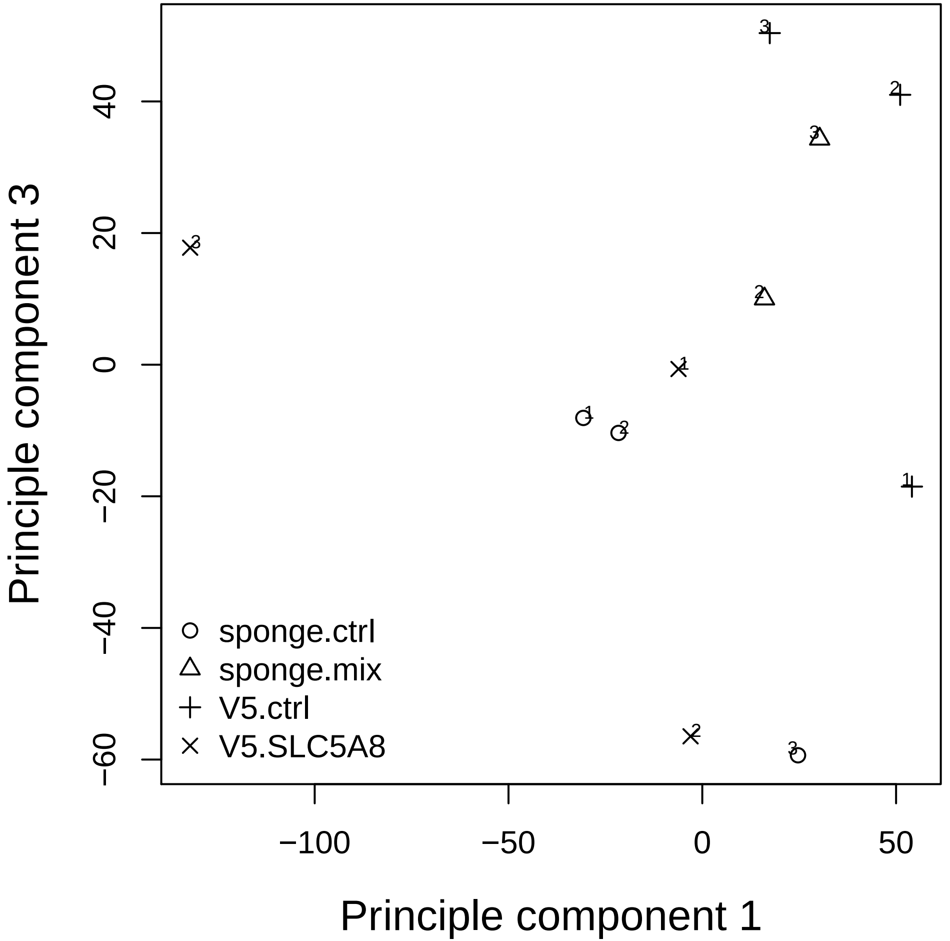

Principle component 3
Principle component 1
40
20
0
−20
−40
−60
−100
−50
0
50
sponge.ctrl
sponge.mix
V5.ctrl
V5.SLC5A8
1
2
3
1
2
3
1
2
3
2
3
