# Supplementary material for: MicroRNAs and Their Inhibition in Modulating SLC5A8 Expression in the Context of Papillary Thyroid Carcinoma
Source: Int J Mol Sci. 2025 Aug 15;26(16):7889. doi: 10.3390/ijms26167889 (PMC12386254; doi:10.3390/ijms26167889)
Supplement: Supplementary file 1 [file ijms-26-07889-s001.zip › ijms-3558049-supplementary/Manuscript data/RNA-Seq/volcano_plots/volcano_sponge.mix_vs_sponge.ctrl.pdf]

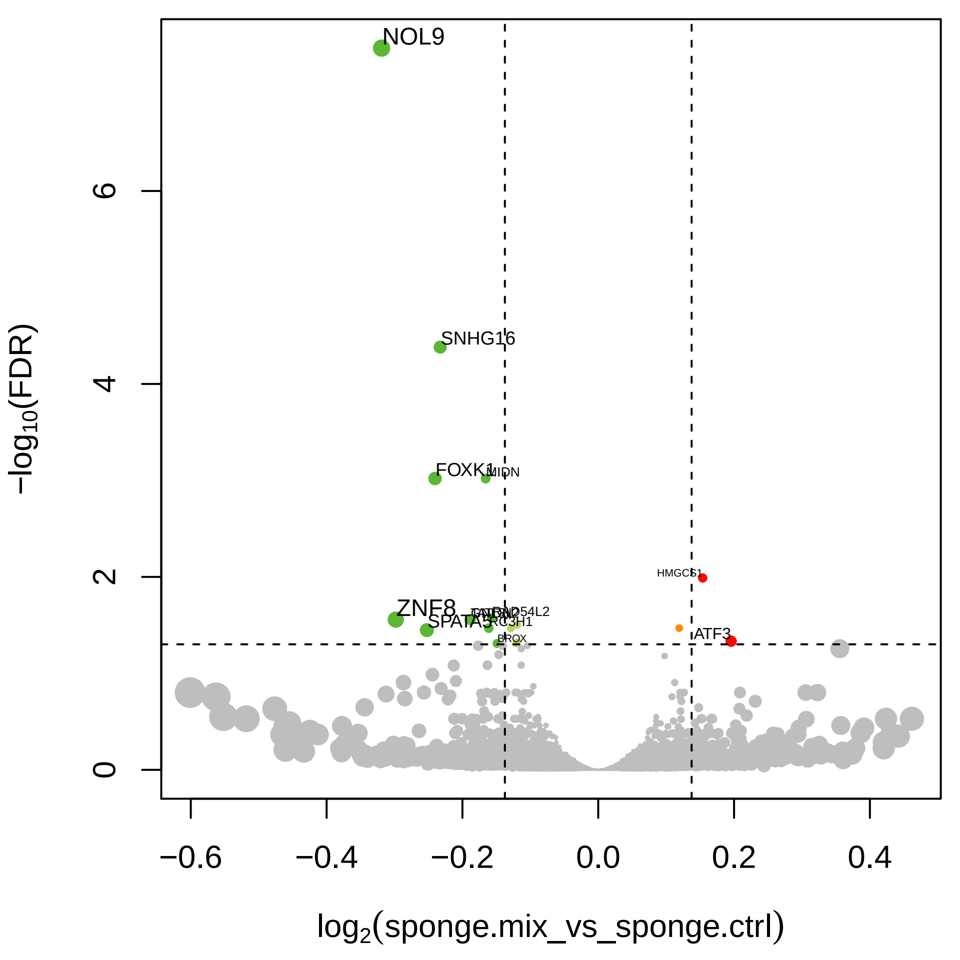

NOL9
SNHG16
FOXK1
MIDN
HMGCS1
ZNF8
SPATA5
ATF3
BROX
0
2
4
6
−0.6
−0.4
−0.2
0.0
0.2
0.4
$-\log_{10}(FDR)$
$\log_2(sponge.mix\_vs\_sponge.ctrl)$
