# Supplementary material for: MicroRNAs and Their Inhibition in Modulating SLC5A8 Expression in the Context of Papillary Thyroid Carcinoma
Source: Int J Mol Sci. 2025 Aug 15;26(16):7889. doi: 10.3390/ijms26167889 (PMC12386254; doi:10.3390/ijms26167889)
Supplement: Supplementary file 1 [file ijms-26-07889-s001.zip › ijms-3558049-supplementary/Manuscript data/RNA-Seq/volcano_plots/volcano_V5.SLC5A8_vs_V5.ctrl.pdf]

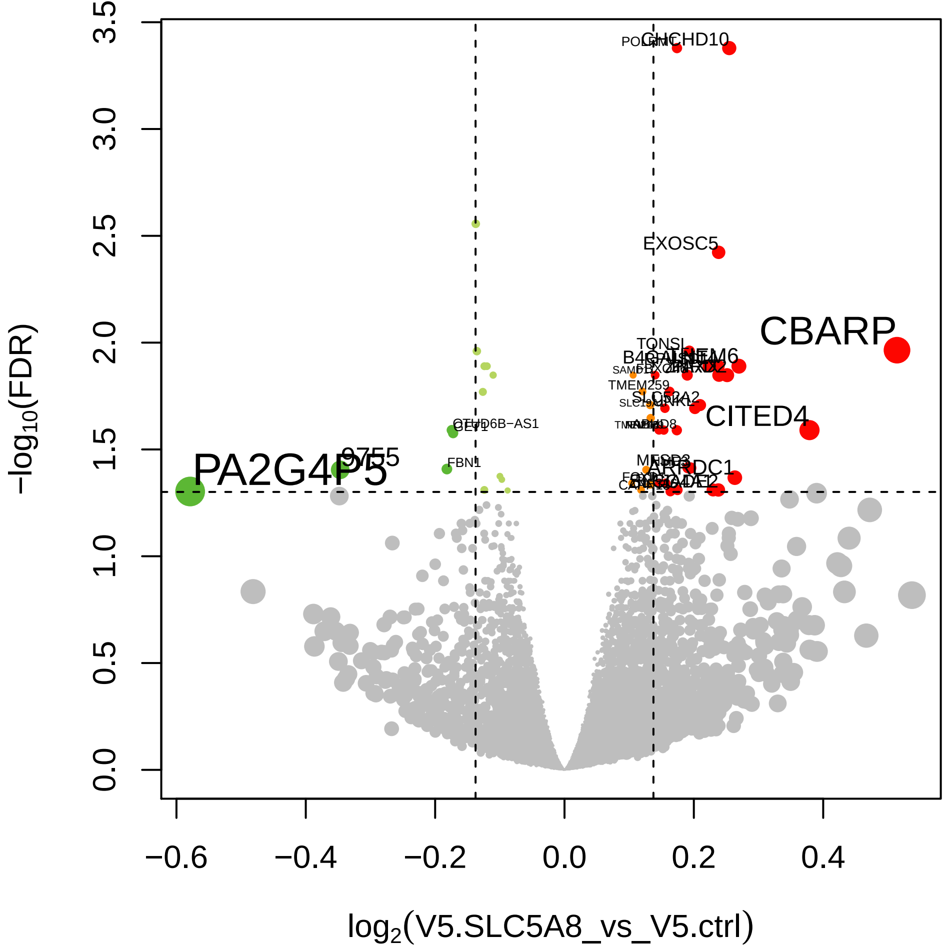

CHCHD10
POLRMT
EXOSC5
CBARP
TONSL
B4GALNT4
TMEM6
SAMD1
FBXO2
TMEM259
SLC52A2
SLC19A1
CITED4
TMEM41
OTUD6B-AS1
GET1
PA2G4P5
9755
FBN1
MFSD3
ARRDC1
−log10(FDR)
log2(V5.SLC5A8_vs_V5.ctrl)
3.5
3.0
2.5
2.0
1.5
1.0
0.5
0.0
−0.6
−0.4
−0.2
0.0
0.2
0.4
