# Supplementary material for: MicroRNAs and Their Inhibition in Modulating SLC5A8 Expression in the Context of Papillary Thyroid Carcinoma
Source: Int J Mol Sci. 2025 Aug 15;26(16):7889. doi: 10.3390/ijms26167889 (PMC12386254; doi:10.3390/ijms26167889)
Supplement: Supplementary file 1 [file ijms-26-07889-s001.zip › ijms-3558049-supplementary/Manuscript data/TableS1.pdf]

Gene	Forward primer (F)	Reverse primer (R)	Product length [bp]
SLC5A8	GATGCCTATGATGGTGGAAG	GAGAGACAGTTTTGCCTGGA	183
HPRT	CAGAGGGCTACAATGTGATG	TGGCGTCGTGATTAGTGATG	180
pre-miR -ctrl sequence	TCCCTCAGATACCGAGTGCAAGTGGGAAGTGATTCAGGTGATCCCCCCGCCGGTATTTCCCTCATGGATATACCGGCGGGGGGATTTGCCCAGAAATCCCAACTACCCTTATGAGTT		135
pre-miR-ctrl	CCCGGTACC-TCCCTCAGATACCGAGTGCAAGTG	CCCGGTACC-AACTCATAAGGGTAGTTGGG	
hsa-mir-181a-2	CGGAAGCTT-CACTGCTCACTGGTTCTTG	CCCGATATC-GTAGTGTCGTCTGTAAGG	162
hsa-mir-182	CGCAAGCTT-TGCCTGGACCATCCTAAC	CCCGATATC-TTCCTCGCGGAGAACAG	240
hsa-mir-494	CGCAAGCTT-ACATCGGCGAAGATTCGG	GCCGATATC-GAAGGCTGCATCAGGAAC	192
sponge	CCC CTCGAG-GCACAAGGAGGTGAGAAG	CCC CTGCAG-GTGGGATGGGAAGCAAAC	approx. 100
sponge-ctrl sequence	CACAAGGAGGTGAGAAGctgggcaaatcccccCGCCGGTactgggcaaatcccccCGCCGGTaGTTTGCTTCCCATCCCA		
sponge-181a-5p sequence	CACAAGGAGGTGAGAAGactcaccgacagcgtTGAATGTtactcaccgacagcgtTGAATGTtGTTTGCTTCCCATCCCA		
sponge-182-5p	ACAAGGAGGTGAGAAGagtgtgagttctaccaTTGCCAAaagtgtgagttctaccaTTGCCAAaGTTTGCTTCCCATCCC		

sequence		
sponge-494-3p sequence	GCACAAGGAGGTGAGAAGgaggtttcccgtgtATGTTTCagaggtttcccgtgtATGTTTCaGTTTGCTTCCCATCCCAC	
sponge-mix sequence	GCACAAGGAGGTGAGAAGactcaccgacacgcTGAATGTtgcatactcaccgacacgcTGAATGTtcgatgaggtttcccacaATGTTTCagactgaggtttcccacaATGTTTCagactagtgtgagttcttggTTGCCAAagcatagtgtgagttcttggTTGCCAAaGTTTGCTTCCCATCCCAC	206
